# The apricot (*Prunus armeniaca* L.) genome elucidates Rosaceae evolution and beta-carotenoid synthesis

**DOI:** 10.1038/s41438-019-0215-6

**Published:** 2019-11-18

**Authors:** Fengchao Jiang, Junhuan Zhang, Sen Wang, Li Yang, Yingfeng Luo, Shenghan Gao, Meiling Zhang, Shuangyang Wu, Songnian Hu, Haoyuan Sun, Yuzhu Wang

**Affiliations:** 1 Beijing Academy of Forestry and Pomology Sciences, 100093 Beijing, PR China; 2 Apricot Engineering and Technology Research Center, National Forestry and Grassland Administration, 100093 Beijing, PR China; 3 CAS Key Laboratory of Genome Sciences and Information, Beijing Institute of Genomics, Chinese Academy of Sciences, 100101 Beijing, China

**Keywords:** Genome, Transcriptomics, Evolution

## Abstract

Apricots, scientifically known as *Prunus armeniaca* L, are drupes that resemble and are closely related to peaches or plums. As one of the top consumed fruits, apricots are widely grown worldwide except in Antarctica. A high-quality reference genome for apricot is still unavailable, which has become a handicap that has dramatically limited the elucidation of the associations of phenotypes with the genetic background, evolutionary diversity, and population diversity in apricot. DNA from *P. armeniaca* was used to generate a standard, size-selected library with an average DNA fragment size of ~20 kb. The library was run on Sequel SMRT Cells, generating a total of 16.54 Gb of PacBio subreads (N50 = 13.55 kb). The high-quality *P. armeniaca* reference genome presented here was assembled using long-read single-molecule sequencing at approximately 70× coverage and 171× Illumina reads (40.46 Gb), combined with a genetic map for chromosome scaffolding. The assembled genome size was 221.9 Mb, with a contig NG50 size of 1.02 Mb. Scaffolds covering 92.88% of the assembled genome were anchored on eight chromosomes. Benchmarking Universal Single-Copy Orthologs analysis showed 98.0% complete genes. We predicted 30,436 protein-coding genes, and 38.28% of the genome was predicted to be repetitive. We found 981 contracted gene families, 1324 expanded gene families and 2300 apricot-specific genes. The differentially expressed gene (DEG) analysis indicated that a change in the expression of the 9-*cis*-epoxycarotenoid dioxygenase (*NCED*) gene but not lycopene beta-cyclase (*Lcy*B) gene results in a low β-carotenoid content in the white cultivar “Dabaixing”. This complete and highly contiguous *P. armeniaca* reference genome will be of help for future studies of resistance to *plum pox virus* (PPV) and the identification and characterization of important agronomic genes and breeding strategies in apricot.

## Introduction

The Rosaceae family provides most of the world’s well-known temperate fruit crops classified as pome and stone fruits according to their fruit morphology. With the global tendency of consumers’ purchasing preferences to shift from large-scale commodity fruit crops (e.g., apples, citrus, and pears) to smaller unique fruit crops with increased nutritional value and a pleasing flavor, the rapid development of stone fruit crops (e.g., apricots, cherries, peaches and plums) has come to the forefront^1–3^.

Apricot (*Prunus armeniaca* L.), which is now generally accepted as a fruit of Chinese origin with a growing history of more than 3000 years in China^4,5^, has been widely grown throughout the world except for Antarctica due to its early harvesting season, unique aroma, delicious taste, high nutritional value and multiple uses. The rich diversity of apricot germplasms indicates that apricots can be grown even more widely and provide a higher proportion of the world’s fruit production. Since the early 2000s, the fruit production and harvested orchard area of apricot have both increased on a worldwide basis, with a 7.9 million Mt fruit tonnage and 536,072 ha orchard area being recorded in 2017, respectively. Compared with 2002, the world production and harvested area in 2017 were increased by 196.5% and 32.2%, respectively. The production of apricot and other *Prunus* species in Europe is currently subjected to severe damaged from sharka disease caused by the *plum pox virus* (PPV)^5,6^. PPV is the most important viral disease affecting a number of *Prunus* species, including apricot^7^. Therefore, it is necessary to construct an apricot genome to localize PPV resistance-related genes to guide the PPV resistance breeding of apricots.

Apricot fruits are enriched in β-carotene, which represents 60−70% of the total carotenoid content^8,9^ and gives the fruit its characteristic orange color^10^. β-carotene is the main precursor of vitamin A, one of the most important functional ingredients in apricots. Vitamin A is an essential nutrient for humans because it cannot be synthesized within the body. Thus, as a good source of β-carotene, apricots are highly beneficial for human health^11,12^. In addition to its nutritional characteristics, apricot fruit also presents some pharmacological significance due to its high antioxidant content. Apricot and/or β-carotene treatment is believed to be effective for preventing the impairments caused by oxidative stress, methotrexate-induced intestinal damage and nephrotoxicity^13,14^. Therefore, the generation of new apricot genotypes with higher levels of β-carotene in the fruit is a promising breeding objective. Understanding the regulation of β-carotenoid metabolism and biosynthesis will allow breeders to develop more effective methodologies for increasing β-carotenoid content and consequently achieving the breeding goal.

Over the last 20 years, genomics research in Rosaceae, and especially in *Prunus*, has made great advances^15^. Due to the small size and simplicity of stone fruit tree genomes, they are considered ideal candidates for the promotion of association genetics approaches based on whole-genome sequence genotyping and genome-wide selection. Now that peach^16^, mume^17^, cherry^18^ and almond^19^ genome sequences are available, genome-level comparative analysis between multigenome sequences within a family has become possible, which provide valuable evolutionary insights and allow the transfer of knowledge between species. In this study, we aimed to construct a complementary apricot genome cv. “Chuanzhihong” using third-generation PacBio technology combined with second-generation Illumina data to understand Rosaceae evolution, particularly the evolution of genes contributing to combating PPV in *Prunus* and to beta-carotenoid synthesis. A high-quality apricot reference genome sequence will afford great opportunities for further research on germplasm diversity, evolution and breeding.

## Methods

### Plant materials

The *P. armeniaca* L plant (“Chuanzhihong”) is native to Hebei Province and has a cultivation history of more than 300 years. It is known as “Chuanzhihong” because of its red color and fruitfulness. “Chuanzhihong” fruit with good comprehensive cultivation characteristics of red color, high yield, disease resistance, and late maturation is a major variety in the northern region. The fruit of “Chuanzhihong” presents good commodity value because it can be stored for more than 10 days under normal conditions and is suitable for long-distance transportation. Leaves of “Chuanzhihong” at an early-to-mid developmental stage were collected for genome sequencing in the Yanqing District (40°35′N and 116°11′E), in the north of Beijing, China, and were immediately frozen in liquid nitrogen and stored at −80 °C until DNA was extracted.

Two Chinese apricot varieties, “Chuanzhihong” (yellow-fleshed fruit) and “Dabaixing” (white-fleshed fruit), from the garden of the Beijing Academy of Forestry and Pomology Sciences, Beijing, China, were selected for RNA sequencing to analyze the biosynthesis of β-carotene. Samples were collected at four developmental stages: green fruit with a soft kernel (G1), green fruit with a hard kernel (G2), color-returning fruit (CT) and fully ripe fruit (FR). Three biological replicates were performed, and ten almost identical fruits were sampled at every stage for each replicate. After the peel was removed, the pulp was quickly cut into small pieces and frozen with liquid nitrogen, then stored at −80 °C until RNA was extracted. The stamen and seed tissues of “Chuanzhihong” from the garden were used for RNA sequencing. All RNA data were used for transcriptome-based gene prediction.

### DNA and RNA sequencing

#### DNA sequencing

For PacBio sequencing, a DNA Template Prep Kit 1.0 was used to generate single-molecule real-time (SMRT)bell genomic libraries. DNA fragments of ~20 kb were obtained using a Covaris g-Tube, and the distribution of the fragments was assessed using a Bioanalyzer 2100 12 K DNA Chip assay. The quality and quantity of the SMRT Bell template (>10 kb) were checked by using an Agilent Bioanalyzer and a Qubit fluorometer, respectively. According to the manufacturer’s instructions, the PacBio Binding Kit 2.0 was used to generate a ready-to-sequence SMRTbell-Polymerase Complex. The genomic DNA was sequenced using SMRT Cells v3.0 with a yield of 16.54 Gb of subreads (Table S1). For short-read sequencing, 150, 180 and 500 bp insert libraries were constructed according to the manufacturer’s instructions, and 40.46 Gb of Illumina sequences was generated using the Illumina HiSeq 2000 platform (Table S2).

#### RNA sequencing

RNA from G1, G2, CT and FR fruits (two cultivars “Chuanzhihong” and “Dabaixing”) was extracted using the RNAprep Pure Plant Kit (Polysaccharides & Polyphenolics-rich). The samples were processed using an RNA library preparation kit and then sequenced on the Illumina HiSeq 2000 platform. The RNA from the stamen and seed tissues of “Chuanzhihong” was extracted using the NEBNext Poly (A) mRNA Magnetic Isolation Module, and libraries were prepared using the NEBNext mRNA Library Prep Master Mix Set. Then, paired-end sequencing with a read length of 100 bp was conducted on the HiSeq 2500 platform.

### Genome size estimation and heterozygosity

The genome size of *P. armeniaca* was estimated using GenomeScope^20^. The quality of the Illumina reads was estimated using the FastQC program^21^. Adapter sequences, PCR duplicates, contaminants, and low-quality sequences (Phred score < 30) were removed using fastp^22^. The analysis of optimal kmer size was performed by using KmerGenie^23^ with chloroplast and mitochondrial sequences being removed from the high-quality clean reads. Then, the best k-mer was used for kmer count analysis with Jellyfish^24^. After converting the k-mer counts into a histogram format, the k-mer distribution was analyzed to estimate genome size and heterozygosity.

### De novo genome assembly

We first estimated the error rate of the long reads obtained from the PacBio platform using Illumina paired-end reads. We applied the Canu^25^ pipeline to assemble the long reads and super-reads obtained from MaSuRCA^26^ into contigs with the following flow parameters: genomeSize = 300 m, corOutCoverage = 100, minReadLength = 1000, minOverlapLength = 1000, ErrorRate = 0.064 and batOptions. One copy of the contigs from heterozygous regions was retained by using Purge_Haplotigs^27^. We then further mapped the Illumina paired-end reads to the filtered contigs using bwa-mem^28^ and polished the contigs with Pilon^29^. We constructed the linkage map (10.1139/CJPS-2018-0177) and organized the contigs into pseudochromosomes with JCVI allmaps^30^ and SLAF markers.

### Transcriptome assembly

The quality control (base correction, adapter trimming and read filtering) of the RNA-seq data from leaf, fruit, stamen and seed tissues (28 libraries in total) was performed by using the software fastp with the default parameters, after which two approaches were used to reconstruct transcripts: de novo assembly and reference-guided assembly. In the de novo assembly, Trinity^31^ was used to reconstruct transcripts from the RNA-seq reads. In the genome-referenced assembly, high-quality RNA-seq reads were mapped to the genomes using Hisat2^32^, and transcripts were built by using Stringtie^33^ with the default settings. CD-HIT^34^ was used to cluster highly similar transcripts for de novo assembly with the default parameters.

### Evaluation of the assembled genome

We first mapped the Illumina paired-end reads to the genome using bwa-mem with the default parameters. Second, RNA-seq data from leaf, fruit, stamen and seed tissues were aligned using Hisat2 with the default settings. Finally, we used BUSCO^35^ to examine the single-copy orthologs (1375, Species: Arabidopsis) with OrthoDB^36^ v10.

### Repeat element identification

The library of species-specific repeats was constructed using RepeatModeler (http://www.repeatmasker.org) with the default parameters, and RepeatMasker^37^ was used to identify repeat elements with the specific library and the default library from the RepeatMasker database (http://www.Repeatmasker.org). Long terminal repeat (LTR) retrotransposons were detected with LTR-Finder^38^ and Inpactor^39^. The phylogenetic tree was constructed to estimate the insertion times of LTR retrotransposons using the Inpactor pipeline.

### Noncoding RNA prediction

The tRNA genes were detected with tRNAscan-SE^40^ using general eukaryote parameters. Ribosomal RNA (rRNA) genes were identified with the program RNAammer^41^. For miRNA prediction, we aligned the mature miRNA sequences in miRBase (www.mirbase.org) against the *P. armeniaca* genome with an e-value < 1e-5 and identity > 95%. The candidate sequences were extracted from the aligned regions with an extension of 90 nucleotides flanking each side and were used to predict RNA secondary structures by using RNAfold^42^ with the default parameters. According to the RNAfold analysis, the candidate miRNAs were selected using the following criteria: (1) candidate sequences were located in one of the hairpin precursor arms, (2) the minimum free energy for the hairpin structures was −20 kcal/mol, and (3) the hairpins were located in intergenic regions or introns. snRNAs and other ncRNAs were predicted using Infernal^43^ with the Rfam database (http://rfam.xfam.org/).

### Gene prediction

Transcriptome-based, homology-based and ab initio prediction methods were applied to predict gene models. For transcriptome-based prediction, the nonredundant and full-length transcripts from the de novo assembly were aligned to the genome to resolve gene structures using PASA^44^. The transcripts from the genome-referenced assembly were applied to obtain reliable transcripts with the longest open reading frames using TransDecoder. For homology-based prediction, the protein sequences of Amygdaloideae genomes were aligned to the genome by SPLAN^45^ with the default settings. The alignments were extended by 1 kb on each side of the hits to identify start and (or) stop codons. For ab initio gene prediction, the training sets (the transcripts obtained from transcriptome-based prediction with complete 5′UTRs and 3′UTR) were used to generate a hidden Markov model (HMM) for ab initio gene prediction. Augustus^46^ and SNAP^47^ were employed to predict gene models in the repeat-masked genome. We used the gene models obtained from the three approaches to generate consensus gene models with EVidenceModeler^44^. We polished the gene models using full-length transcripts according to the following steps: (1) We rectified the potential mistakes caused by frame-shift problems. (2) Priority was given in the following order: SPALN (Conserved), PASA (Expressed), and EVM (Predicted). (3) If genes overlapped on the same strand, the longer one was retained. (4) Gene overlaps with different strand orientations were allowed. (5) Gene nesting in another gene’s intron was allowed. Finally, we identified the UTRs and alternative splicing of the models with PASA.

### Gene functions

Gene functions were assigned by searching the predicted proteins against public databases by using BLASTP^48^ with e-value < 1e-5, including the UniProt and the KEGG (Kyoto Encyclopedia of Genes and Genomes) databases. We aligned the proteins against the InterPro database using InterProScan^49^ to identify protein domains and transmembrane helices and to assign gene ontology (GO) terms. Transcription factor (TF) identification was carried out using an online web resource (http://planttfdb.cbi.pku.edu.cn/prediction.php).

### Gene families and synteny

We collected the protein sequences from *Prunus armeniaca* L. and 13 other species (*Prunus persica* (L) Batsch, *Prunus avium* (L) L., *Prunus mume* (mei), *Prunus dulcis* Miller., *Malus domestica* Borkh., *Fragaria vesca* L., *Rosa chinensis* Jacq., *Vitis vinifera* L., *Pyrus bretschneideri* Rehder., *Populus trichocarpa* Torr., *Oryza sativa* L.*, Arabidopsis thaliana* (L.) Heynh. and *Amborella trichopoda* Baill.) to analyze gene families. An all-to-all BLASTP analysis of proteins with a length ≥30 aa was performed with an e-value < 1e-5. According to the BLASTP results, paralogous and orthologous genes were identified by using the software OrthoFinder^50^ with an inflation of 1.5. The all-to-all BLASTP results between *P. armeniaca* and *P. persica*, *P. armeniaca* and *P. dulcis*, and *P. armeniaca* and *P. avium* were extracted, and the orthologous gene blocks on the chromosomes or pseudomolecules were identified using the software MCscanX with the default parameters^51^.

### Phylogenetic reconstructions and divergence time estimation

Phylogenetic construction was performed based on 269 single-copy genes extracted from the gene family analysis. We utilized MAFFT^52^ to construct protein alignments for each single-copy gene family and removed gaps from the alignments using the program trimAL^53^. The protein alignments with a length ≥30 aa were concatenated for subsequent analyses. The best substitution model for the alignment was estimated by using ModelFinder^54^ with the default settings. The maximum likelihood tree was constructed using IQ-TREE^55^ with a Best-fit model of JTT + F + R3 and 1000 bootstrap replicates.

The divergence time of each node in the phylogenetic tree was estimated based on the JC69 model in the MCMCTree program from the PAML package^56^. The use data parameter was set to 1 for the calculation of the likelihood function in a normal way. For the clock parameter, the correlated rates were used following a log-normal distribution. In total, the MCMCTree was run for 1,000,000 generations, with a burn-in 10,000 iterations to a stable state. Three reported divergence times were used as a calibration. The divergence times between the Amygdaleae and Maleae^57^ (48.4 Ma), *P. trichocarpa* and *A. thalian*^58^ (100−120 Ma) and monocots and eudicots^59^ (~240 Ma) were used as calibrators.

### Gene family expansion and contraction analysis

The gene family count profile used as the input file for CAFE^60^ was obtained with the program OrthoFinder. The phylogenetic tree generated by IQ-TREE was converted to an ultrametric tree using r8s^61^. The *λ* value was estimated with the CAFE program to identify the expansion or contraction of gene families based on a stochastic birth and death process model. We only considered gene families that were significantly expanded or contracted with *p* values smaller than 0.01. We considered both expansion and contraction compared to the RCAs (recent common ancestors) of species. We considered a gene family to be unchanged if the species and its RCA exhibited the same gene copy.

### Synonymous substitutions per synonymous site (*K*_s_) distribution

Orthologous genes and paralogous genes among *P. armeniaca*, *P. persica*, *P. avium*, *P. mume, M. domestica* and *P. bretschneideri* were extracted from the gene families. Protein alignments were constructed by using MAFFT, and the corresponding CDS (nucleotide sequences) alignments were converted. Then, the *K*_s_ value for each pair of orthologous genes and paralogous genes was calculated using codeml (CodonFreq = 2, runmodel = −2) in the PAML package.

### RNA-seq and WGCNA analysis

In genome-referenced mapping, the high-quality reads from RNA-seq data (“Chuanzhihong” and “Dabaixing”, G1, G2, CT and FR) were aligned to the genome by using Hisat2 and transcripts of each sample were built by using Stringtie with the default parameters. The fragments per kilobase of transcript per million fragments mapped (FPKM) for each gene were calculated using Stringtie with the -G parameter employing the gff3 genome as the reference. The differentially expressed genes (DEGs) were analyzed using the edgeR R package (FDR < 0.05, logFC ≥ 1)^62^. The expression levels of the genes involved in carotenoid metabolism and genes encoding transcription factors were used to construct the correlation network by using the WGCNA R package^63^.

## Results

### Genome assembly

**Fig. 1:**
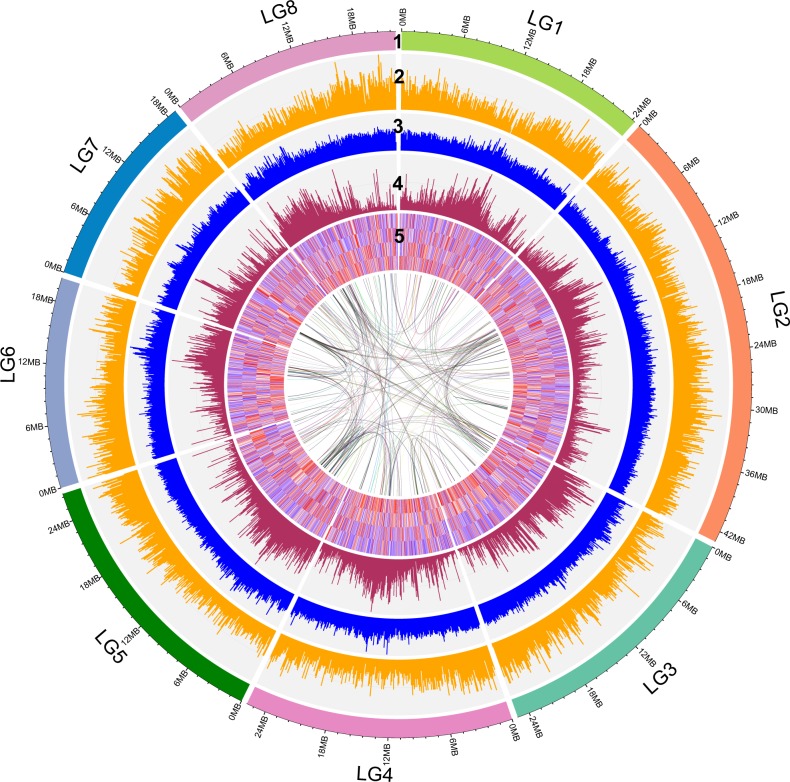
Genetic structure and variant density of apricot. (1) Pseudomolecules; (2) gene density; (3) GC content (per 100 Kb); (4) repeat density (per 100 Kb); (5) heatmap of the G1, G2, CT and FR stages.

The genome of apricot (*P. armeniaca* L) (2*n* = 16) is small but highly heterozygous. The genome size and fraction of heterozygosity in *P. armeniaca* were estimated to be 220.36−220.56 Mb and 0.900−0.902%, respectively, according to evaluation with GenomeScope (best k-mer = 61, obtained with KmerGenie). After the purging of haplotigs, we obtained a haplotype assembly with 444 contigs, and its size was 221.9 Mb, with a contig NG50 size of 1.02 Mb (Table 1). A total of 92.88% of the assembly was anchored to eight linkage groups using linkage maps, and the pseudomolecules ranged in size from 18.6 to 43.0 Mb (Fig. 1, Table S3). The Illumina reads were remapped to the genome, and single nucleotide polymorphisms were called to estimate the level of heterozygosity (0.96%). It can be seen from Fig. 1 that the gene density and GC content were uniformly distributed on eight chromosomes, but the repetition density was not uniformly distributed either in the whole genome or on each chromosome.

**Table 1:** Genome features of *P. armeniaca.*

	Assembly	Pseudomolecules
Size (bp)	221,901,797	206,096,285
Number	444	8
NG50 (bp)	1,020,063	25,125,992
N50 (bp)	1,018,044	25,125,992
GC content (%)	37.6%	37.42%
Maximum size (bp)	5,999,228	42,984,470
Minimum size (bp)	1159	18,857,615
Mean size (bp)	499,724	25,762,035

### Assembly validation

To assess the quality of the apricot genome, we aligned the Illumina clean data to the apricot genome and obtained a mapping ratio of 99.36%. We also quantified the coverage by the PacBio data, which was 99.87%. In addition, we aligned resequenced *Prunus sibirica* Illumina paired-end reads (SRR5046735) to the assembled genome and found that 98.69% of the reads could be mapped^58^. The alignment rates of the RNA-seq reads from three different tissues (flower, fruit and seed) were approximately 91.45 and 96.45% (Table S4). The BUSCO analysis showed that 98.0% of the complete genes could be detected in the assembly (Table S5).

### Repeat annotation

Among the predicted repeats in the apricot genome, long terminal repeats (LTR) comprised the largest proportion (13.43%) (Table S6). Unclassified elements ranked second, accounting for 12.17% of the genome. The DNA class repeat elements comprised 9.50% of the genome. Altogether, 38.28% of the genome was predicted to be repetitive. The phylogenetic tree of LTR retrotransposons showed that the repeat elements were clustered into four groups: *Gypsy*, *Copia*, retrovirus and others (Fig. S3). The mean divergence times of *Gypsy* and *Copia* were 0.97 and 0.88 Mya (million years ago), and both groups exhibited recent active transposition events (Fig. S4). Altogether, 38.28% of the genome was predicted to be repetitive, which is comparable with the repeat content observed in mume (45.0%) and sweet cherry (43.8%) but higher than that observed in peach (29.6%).

### Gene prediction and functional annotation

A total of 30,436 protein-coding genes were predicted, with an average transcript length of 1641 bp, by using a combination of homology-based, ab initio and transcriptome-based prediction methods (Table S7). The average gene density of apricot was 137 genes per Mb, which is higher than in peach (122 genes per Mb), mume (132 genes per Mb), sweet cherry (87 genes per Mb) and almond (112 genes per Mb). We identified 905 ribosomal RNAs (5S, 5.8S, 18S and 28S), 488 transfer RNAs, 353 small nuclear RNAs and 278 microRNAs (Table S8). The proportions of all gene models annotated to the Nr, Pfam^59^, KEGG, GO, UniProt and Trans Membrane prediction (TMHMM) databases were 99.17%, 86.07%, 43.40%, 54.59%, 71.53% and 23.51%, respectively (Table S9). We also detected 1363 transcription factors in the apricot genome (Table S10).

### Genome evolution

**Fig. 2:**
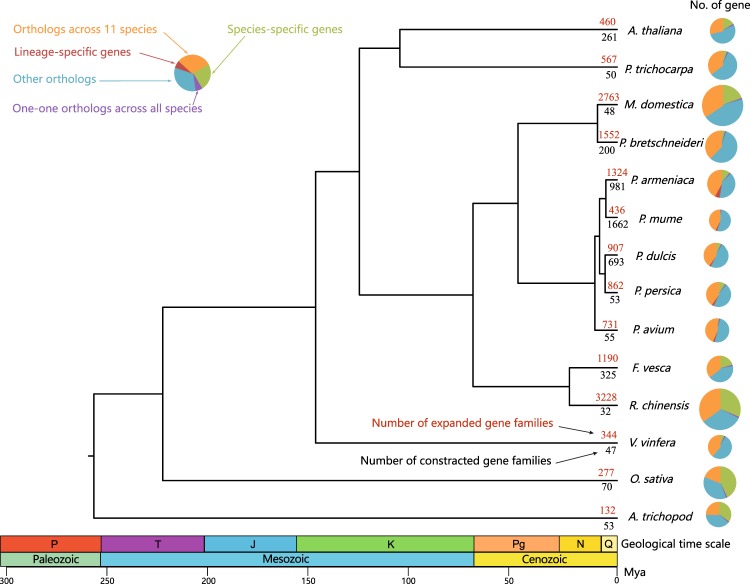
**Phylogenetic tree and gene family changes of apricot and related species**.

The phylogenetic tree of apricot and related species was constructed using the maximum likelihood method, and the divergence time among branches of the tree was estimated (Fig. 2, Figs. S5, S6). The phylogenetic tree indicated that apricot was more closely related to *P. mume* (Japanese apricot) and that the ancestor of the two species split ~5.53 million years ago. The estimated divergence time of the ancestor of sweet cherry was relatively distant in the four *Prunus* species, at 10.92 million years (Fig. 2, Fig. S6).

A collinear analysis of the three closely related *Prunus* species (*P. armeniaca*, *P. persica* and *P. dulcis*) was performed, and the results showed that the three species exhibited high collinearity. A total of 16,780 and 13,094 apricot genes were located in collinear blocks between apricot and peach and between apricot and almond, respectively (Fig. S7). Functional annotation showed that these collinear genes were mainly involved in the basic needs of organisms, including energy and other types of metabolism (Fig. S8).

Gene family analysis showed that during the evolution of apricot, 1324 gene families expanded, while 981 families contracted and produced 2300 apricot-specific genes (Fig. 2, Table S11). The genes from the expanded families were mainly enriched in phenylpropanoid biosynthesis (*p* = 0.0018) and flavonoid biosynthesis (*p* = 0.0019) (Fig. S8, Table S12). In addition, the citrate synthase family was expanded, with three copies in the apricot genome (the additional copy came from the recent species-specific tandem duplication event) and two in other species in the *Prunus* genus. Gene expression analysis indicated that the three citrate synthase-encoding genes were highly expressed during fruit development. The functions of the apricot-specific genes were enriched in transport (sulfate transport (*p* = 6.0404e-9), anion transport (*p* = 6.0985e-6), transmembrane transport (*p* = 5.1224e-5), and oxidation reduction (*p* = 1.7853e-5)). A total of 361 apricot-specific genes were transcriptionally active during at least one of the four fruit developmental stages, indicating that these genes may play an important role in the growth and development of apricot (Fig. S8, Table S13).

Although apricot has not experienced whole-genome duplication events, as observed in apple and pear (Fig. S10), there were many large segmental duplication regions in the apricot genome. We identified 290 gene blocks in the apricot genome, involving a total of 2794 genes. These genes were mainly enriched in pathways of plant−pathogen interactions (*p* = 0.00029) and phenylpropanoid biosynthesis (*p* = 0.0011) (Fig. S8, Table S14).

### MATHd evolution of PPV in *Prunus*

**Fig. 3:**
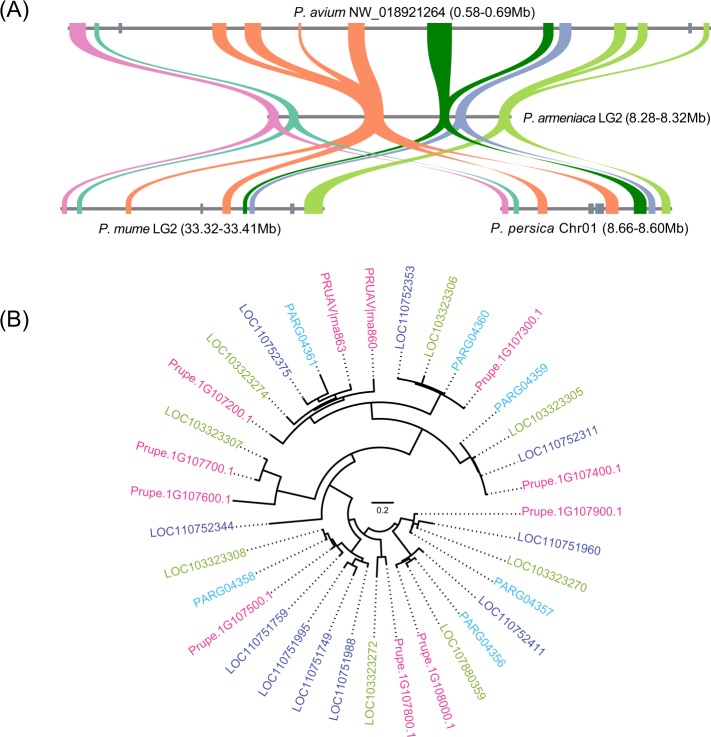
MATHd proteins within apricot, *P. mume*, *P. persica* and *P. avium*. **a** Orthologous regions; **b** the phylogenetic tree of MATH proteins from apricot (blue), *P. mume* (green), *P. persica* (pink) and *P. avium* (dark blue).

The comparative analysis of MATHd-orthologous regions within related *Prunus* species (*P. armeniaca*, *P. avium, P. mume* and *P. persica*) was performed to detail the evolutionary history of these important gene clusters (Fig. 3a). These species exhibited different copy numbers within these regions; *P. armeniaca* exhibited 6, *P. mume* 7, *P. persica* 7 and *P. avium* 12. Phylogenetic tree analysis suggested that species-specific tandem duplication and perhaps gene loss events contributed to the architectural composition of these orthologous syntenic regions (Fig. 3b).

### Carotenoid metabolism

**Fig. 4:**
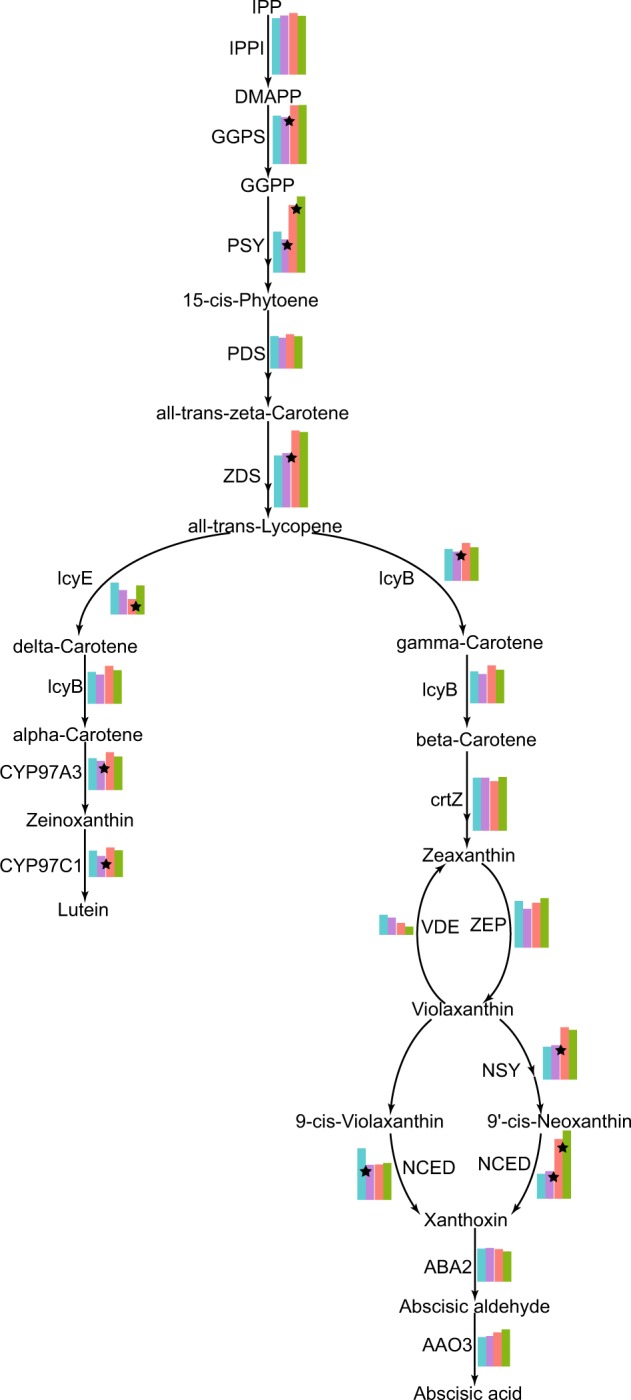
Carotenoid metabolism pathway of apricot. The expression levels of genes are shown in a bar chart with different colors corresponding to different stages. A black asterisk indicates a significant change (*p* ≤ 0.05) between two adjacent stages.

**Fig. 5:**
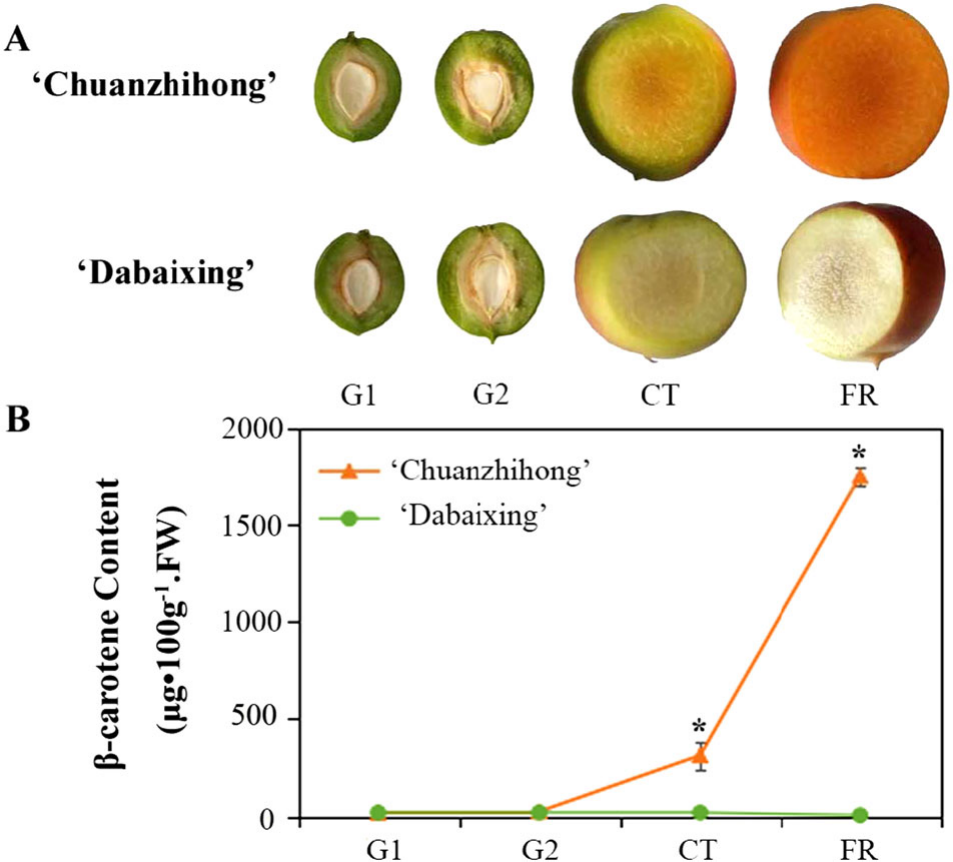
**Changes in the color and beta-carotenoid content of apricot fruits in different developmental stages**.

Carotenoids play an important role in plant photosynthesis and lipid peroxidation and impact the color traits of plant fruits. We analyzed the dynamic changes in gene expression levels in four stages of apricot pulp (G1, G2, CT and FR) (Fig. 4). Differentially expressed gene analysis showed that 2532, 4708, and 2033 genes differed between G1 and G2, G2 and CT, and CT and FR, respectively, and 1, 9, and 3 genes were involved in carotenoid metabolism (Fig. 4, Fig. S11, Table S15A, B, C). During the G2 to CT phase of fruit ripening, the expression levels of the genes changed more significantly, especially those of genes related to beta-carotene synthesis. The gene encoding the enzyme *LcyB* (lycopene beta-cyclase), which is the key enzyme in the synthesis of carotene, was significantly upregulated, indicating that beta-carotene is rapidly synthesized during the CT phase. The genes encoding PSY (15-*cis*-phytoene synthase) and ZDS (zeta-carotene desaturase), which play an important role in the synthesis of the precursors of beta-carotene, were also upregulated between G2 and CT. Although the carotene content increased, there was no significant change in beta-carotene synthesis-related gene expression between CT and FR (Fig. 5), which revealed that high expression of the *LcyB* gene results in the accumulation of carotenoids.

We further explored the transcription factors involved in the regulation of beta-carotene synthesis through coexpression network analysis as described in WGCNA (Figs. S12, 13). We identified 95 transcription factors involved in the regulation of carotenoid metabolic pathways, 12 of which were related to the *LcyB* gene (Table S16). Among the 11 transcription factors, 9 were involved in positive regulation, and 3 were involved in negative regulation (Table S17).

The most important difference in the color of the white and yellow cultivars was the beta-carotene content, but DEG analysis showed that no obvious changes in the genes related to beta-carotene synthesis (Fig. S14). We found that the expression of the gene encoding the enzyme NCED (9-*cis*-epoxycarotenoid dioxygenase), which catalyzes the synthesis of 9-*cis*-neoxanthin-synthesized xanthoxin, was very active, and its expression level was significantly different between the two cultivars “Chuanzhihong” and “Dabaixing” (Fig. S14). In the white cultivar, the synthesis and decomposition of carotene were balanced, and the newly synthesized carotenoids were converted into xanthoxin, the precursor of abcisate, through enzyme catalysis, especially by *NCED*, halting the accumulation of carotene.

## Discussion

Fruit trees usually exhibit high heterogeneity, which makes it more difficult to obtain high-quality complete genomes, and this effect is particularly evident in the released genomes of stone fruit trees. In the assembly of the apricot genome, to overcome the problem caused by heterozygosity, we first assembled the Illumina data into super-reads by using MaSuRCA, and super-reads and corrected PacBio subreads were then assembled into contigs constituting the diploid genome. After purging haplotigs, the apricot genome N50 size was 1,018,044 bp, and the contig N50 sizes of peach, cherry, mume and almond were 294, 276, 31,772 and 77,040 bp, respectively, which suggested that the *P. armeniaca* reference genome was the most contiguous among the sequenced stone fruit trees^16–19^. The BUSCO analysis showed that 98.0% complete genes were detected in the assembly, which indicated that the genome quality of apricot was better than those of the other published stone fruit genomes^16–19^. In brief, these results indicate that the genome of apricot is relatively accurate and complete among the available genomes of stone fruit trees.

*Plum pox virus*, also known as sharka, is a linear single-stranded RNA virus that affects *Prunus* species. The selection of PPV-resistant genetic resources in *Prunus* germplasms is an important approach for resistance breeding that is currently effective against PPV; with the breakthroughs regarding anti-PPV genes, biotechnological strategies have been applied or may be exploited to confer PPV resistance^64,65^. Recent studies have shown that apricot resistance to PPV is associated with the pseudogenization/downregulation of two tandemly duplicated MATHd genes^66^. By comparing the changes in MATHd orthologues in *Prunus*, we found that the associated regions were vertically inherited from the ancestor of *Prunus* species and that at least two tandemly arrayed copies have been retained in each species; the loss of the MATHd genes may result in susceptibility to PPV. Considering these results together, it will be interesting to test the roles of these genes in PPV infection through molecular studies, genetic association studies and molecular breeding within other *Prunus* species.

The balance of the biosynthesis and decomposition of β-carotene may contribute to the color of apricot fruit. First, β-carotene, as one of the metabolites important for fruit quality, contributes to the yellow color of apricot fruit^67,68^. Among fruit pigments, anthocyanins are the main typical pigments contributing to the red, pink or violet coloration of some fruit, such as apple, tomato, purple sweet potato, and strawberry^69–76^. However, in apricot fruit, β-carotene is detectable and shows significantly higher levels in the yellow-flesh of “Chuanzhihong” than in the white-flesh of “Dabaixing”. These results showed that β-carotene is the main pigment of apricot with yellow flesh (Fig. 5), which is supported by studies from Curl^77^ and Roussos et al.^10^ There are also many other fruit species with high levels of β-carotene, such as citrus, carrot, mango, papaya, and tomato^73,78^.

Moreover, the expression patterns of β-carotene synthesis genes in plants are tissue- and stage-dependent. *Psy, pds, zds, lcy-e, crt-b, zep* and *necd3* are all expressed in coffee leaves, flowers and shoots, but the transcript levels differ among the three tissues^79^. For tomato, citrus, watermelon and other fruit-type crops, the genes related to β-carotene biosynthesis appear to show the highest transcript levels, and rapid β-carotene accumulation is mainly observed in the nearly mature stage^80–82^. Lycopene and β-carotene rapidly accumulate in the flesh of Cara citrus fruit during the two stages of fruit enlargement and fruit ripening. The present study indicated that the G2 to CT stages may be the corresponding key period in apricot. As described in the literature, the color change from green to red or yellow is very important in fruit development as anthocyanins or carotenoids accumulate, in addition to sugar, ABA and ETH, to promote fruit ripening^83–85^. A similar result was observed in this study (Fig. 5). During the G2 to CT phase of fruit ripening, the expression levels of genes changed more significantly, especially those of genes related to β-carotene synthesis. Multiple metabolic pathways are involved in this stage to affect fruit development and ripening, including β-carotene biosynthesis. The β-carotene content of “Chuanzhihong” rapidly increased to a high level beginning in the CT stage and reached its highest level at the FR stage, which indicates maturity of the fruit.

Furthermore, all the DEGs related to the β-carotene biosynthesis pathway were analyzed. In apricot, *lcy-b* may make an important contribution to yellow flesh development in the ripening fruit of “Chaunzhihong”. As shown in Fig. S10, *lcy-b* was significantly upregulated, indicating that β-carotene was undergoing rapid synthesis during the CT phase, which is similar to the change trend of flesh color. In contrast, among the other three important genes (*psy, pds, zds*) for β-carotene biosynthesis, the gene expression levels were not constant with the accumulation of β-carotene. All of the previous studies indicated that the gene expression changes controlling β-carotene biosynthesis in plants among different species or different varieties are very complex. However, it is interesting that, in the white cultivar “Dabaixing”, the transcript level of the *NCED* gene is much higher during fruit development, especially in the last two stages of CT and FR, which is contrary to what is observed in the yellower cultivar “Chuanzhihong” (Fig. S13). The newly synthesized carotenoids are rapidly converted into xanthoxin, the precursor of abcisate, through enzyme catalysis, especially that by *NCED*, halting the accumulation of carotene. Thus, the balance of the biosynthesis and decomposition of β-carotene may contribute to the color of apricot fruit, providing the first report of the likely mechanism of fruit color development in apricot. Further research with respect to the characterization and functional identification of *lcy-b* and *NCED* as well as the possible transcriptional regulation mechanism of β-carotene in apricot will be carried out, which is valuable for basic research and the future breeding of new apricot varieties rich in β-carotene.

## Conclusions

In this study, we first report the sequencing, assembly and annotation of the apricot (*Prunus armeniaca* L) genome, along with the significant evolutionary features of the main *Prunus* species. The MATHd genes were shown to be vertically inherited from the ancestor of the *Prunus* species and retained at least two tandemly arrayed copies in apricot, cherry, mume and peach species. The *NCED* (9-*cis*-epoxycarotenoid dioxygenase) gene, not *Lcy*B (lycopene beta-cyclase), results in a low β-carotenoid content in the white cultivar “Dabaixing”. The chromosome-scale assembly of apricot will provide more important gene resources for future studies on stone fruit crops, which is also valuable for efficiently screening functional genes related to agronomic traits as well as for GWAS (genome-wide association study) analysis and fine QTL mapping. Taken together, the results of this study indicate that it is feasible to use this genome as a tool for improving breeding strategies.

## Supplementary information

Supplementary Figures

Supplementary Tables

## Data Availability

The data that support the findings of this study have been deposited in the CNSA (https://db.cngb.org/cnsa/) of CNGBdb with accession number CNP0000755. The genome assembly data have been deposited in genome database for Rosaceae (www.rosaceae.org).
