# Supplementary material for: The apricot (Prunus armeniaca L.) genome elucidates Rosaceae evolution and beta-carotenoid synthesis
Source: Hortic Res. 2019 Nov 18;6:128. doi: 10.1038/s41438-019-0215-6 (PMC6861294; doi:10.1038/s41438-019-0215-6)
Supplement: Supplementary file 1 — Supplementary Figures [file 41438_2019_215_MOESM1_ESM.pdf]

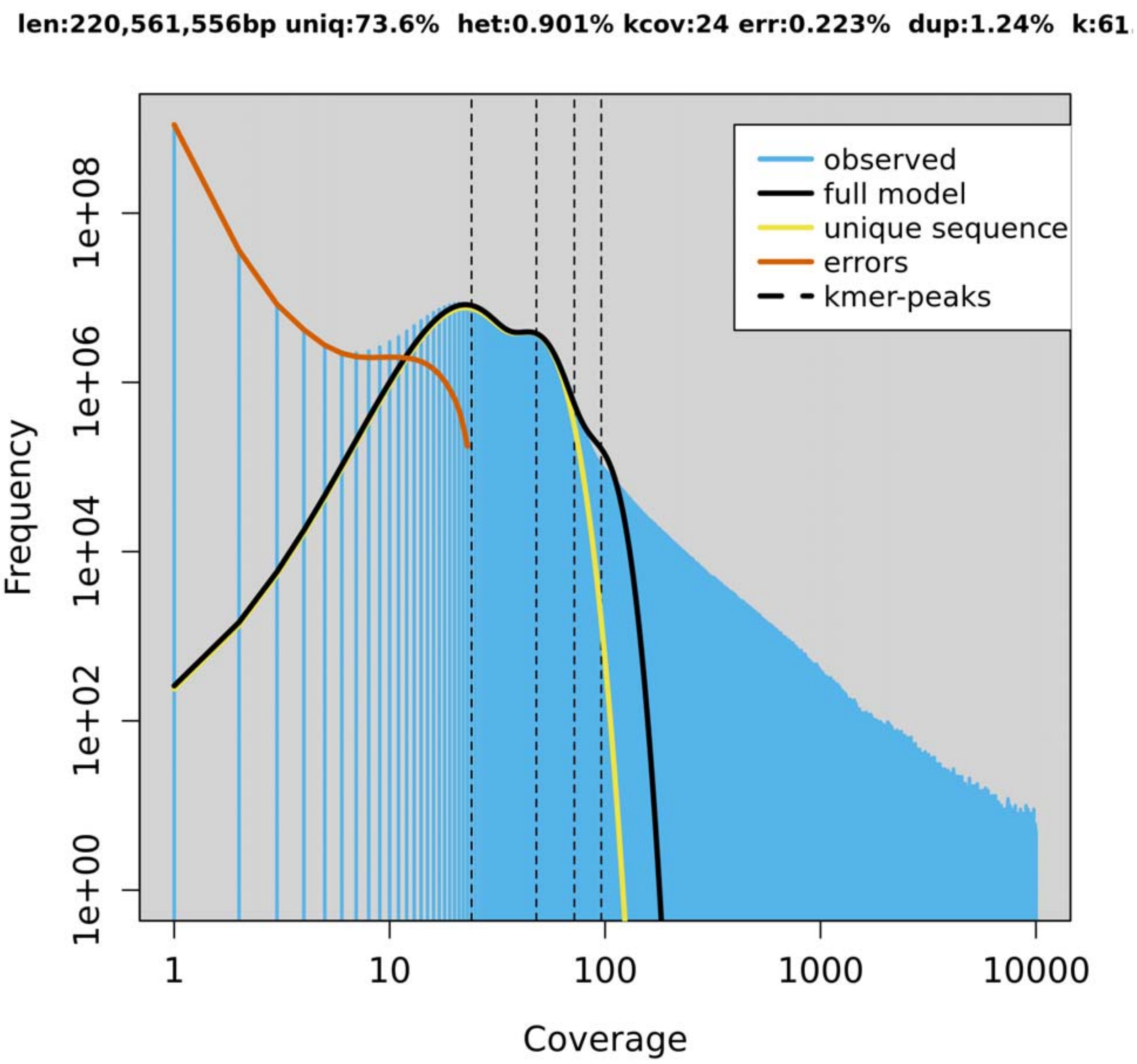

Fig. S1: Genome size estimation generated by Genomescope, providing a k-mer analysis (k-mer=61) to estimate haploid genome size, fraction of heterozygosity.

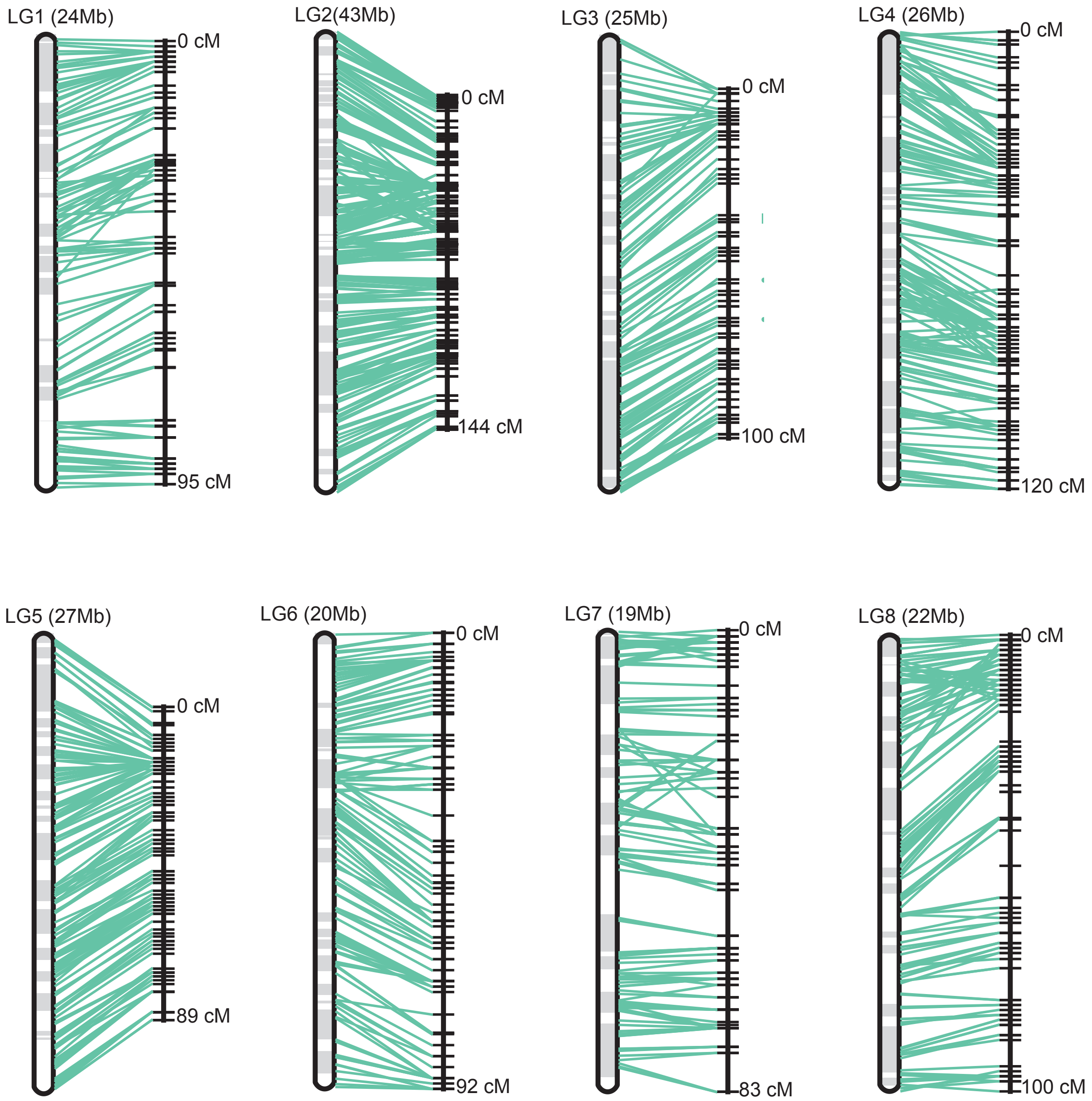

Fig. S2**:** Anchoring the assembled contigs of apricot genome to the linkage groups of the genetic map.

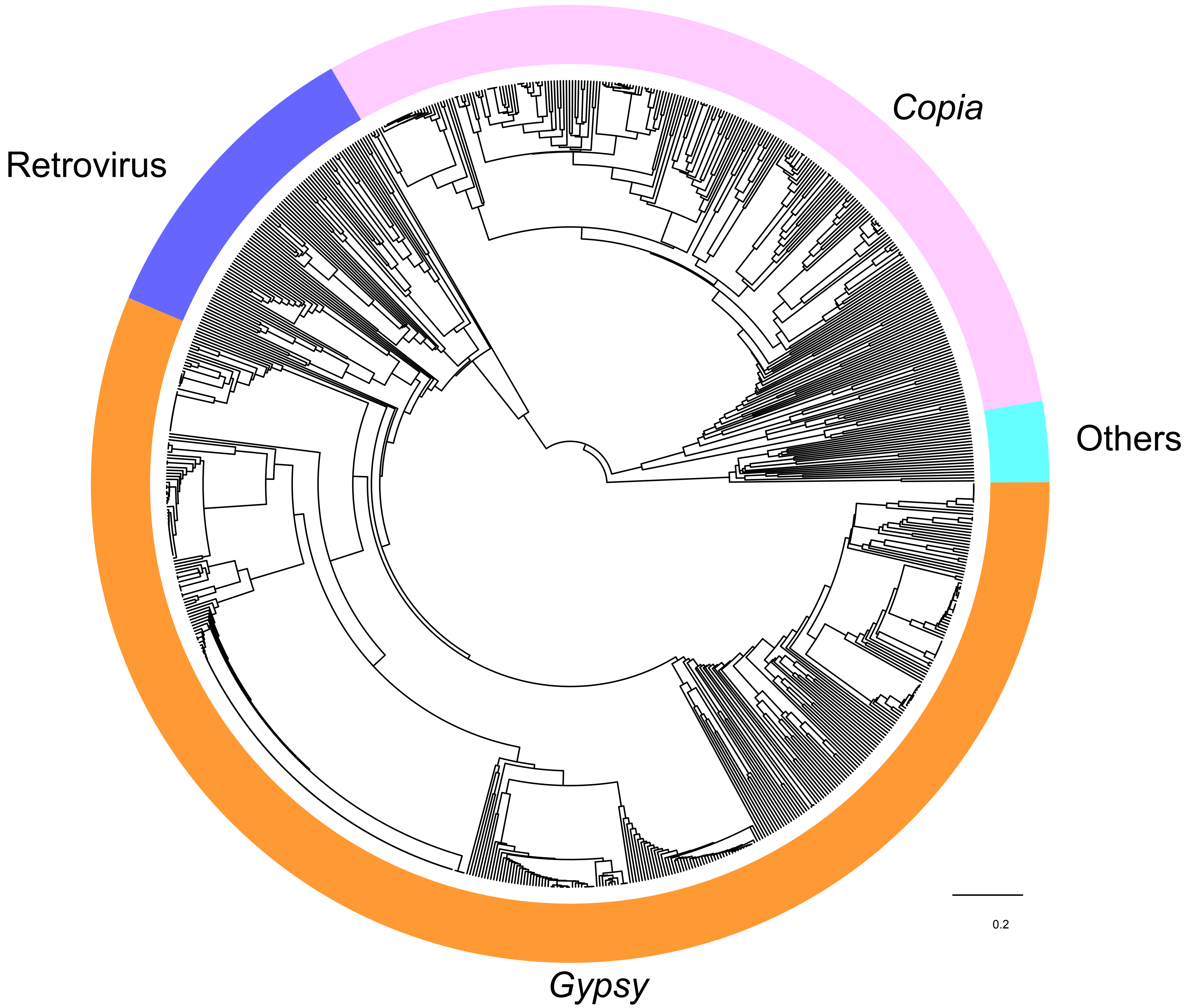

Fig. S3: The phylogenetic tree of LTR retrotransposons.

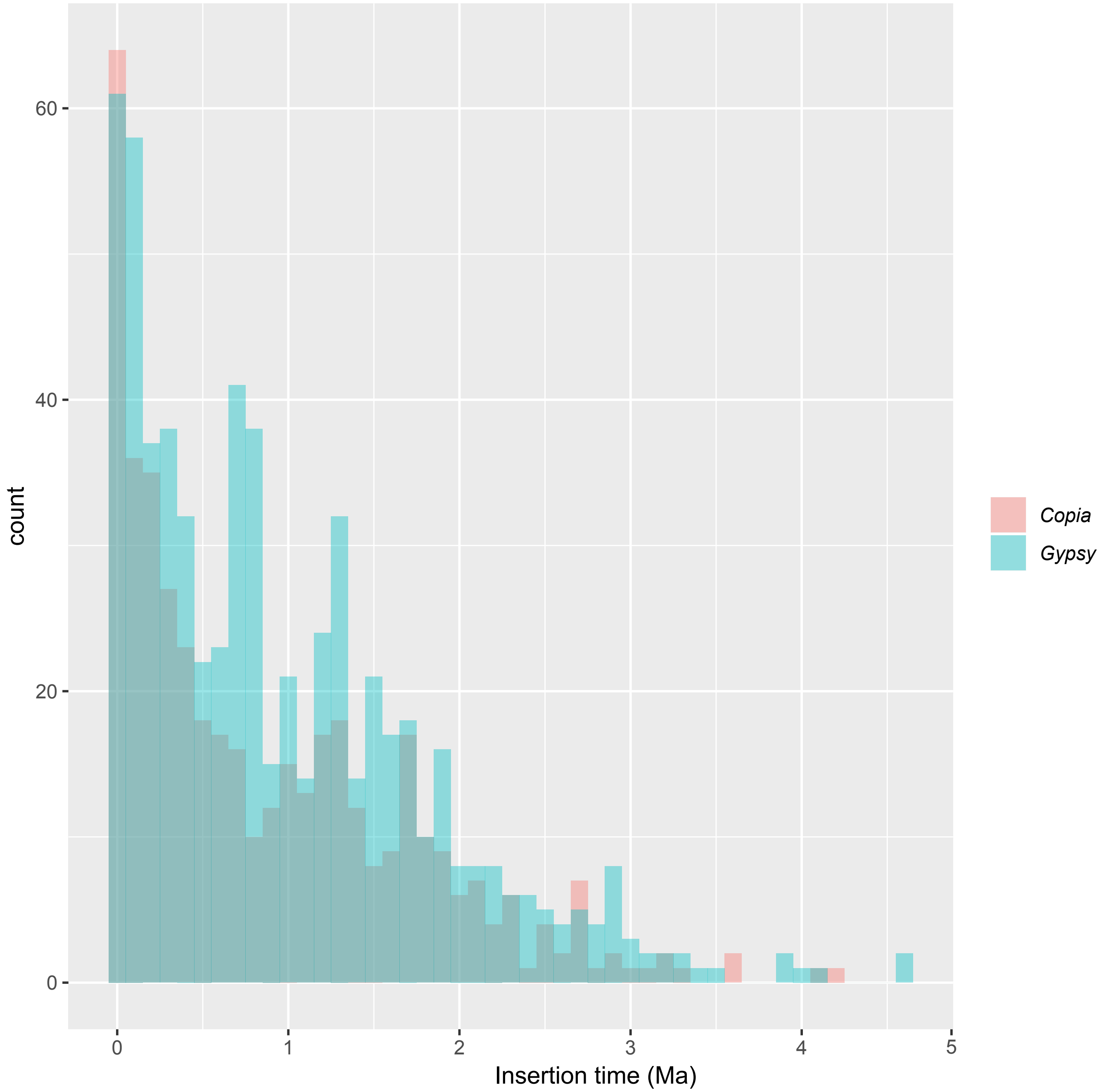

Fig. S4: Insertion times of Gypsy and Copia in apricot.

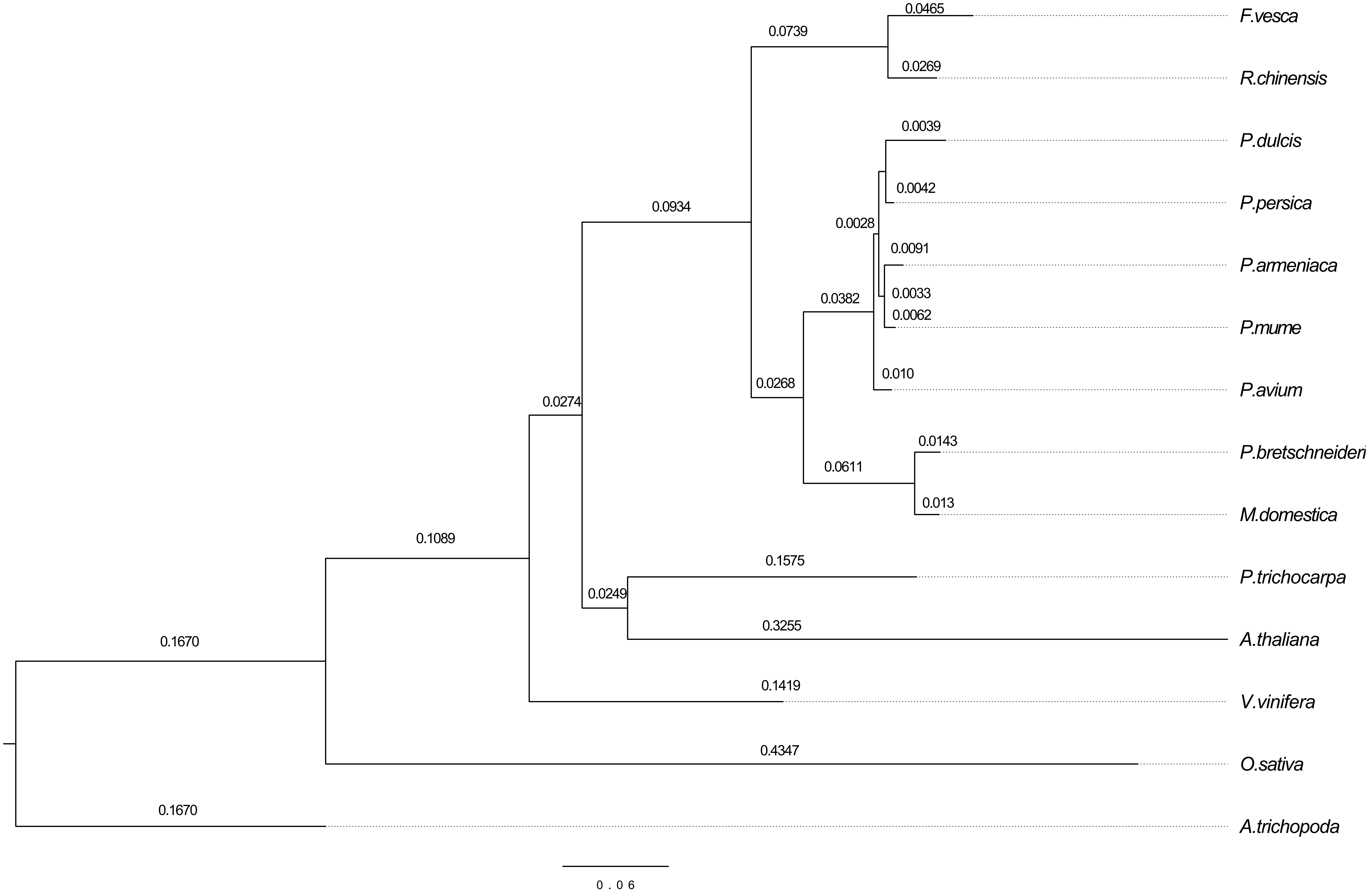

Fig. S5: The phylogenetic tree of apricot and related species (Number indicates branch length).

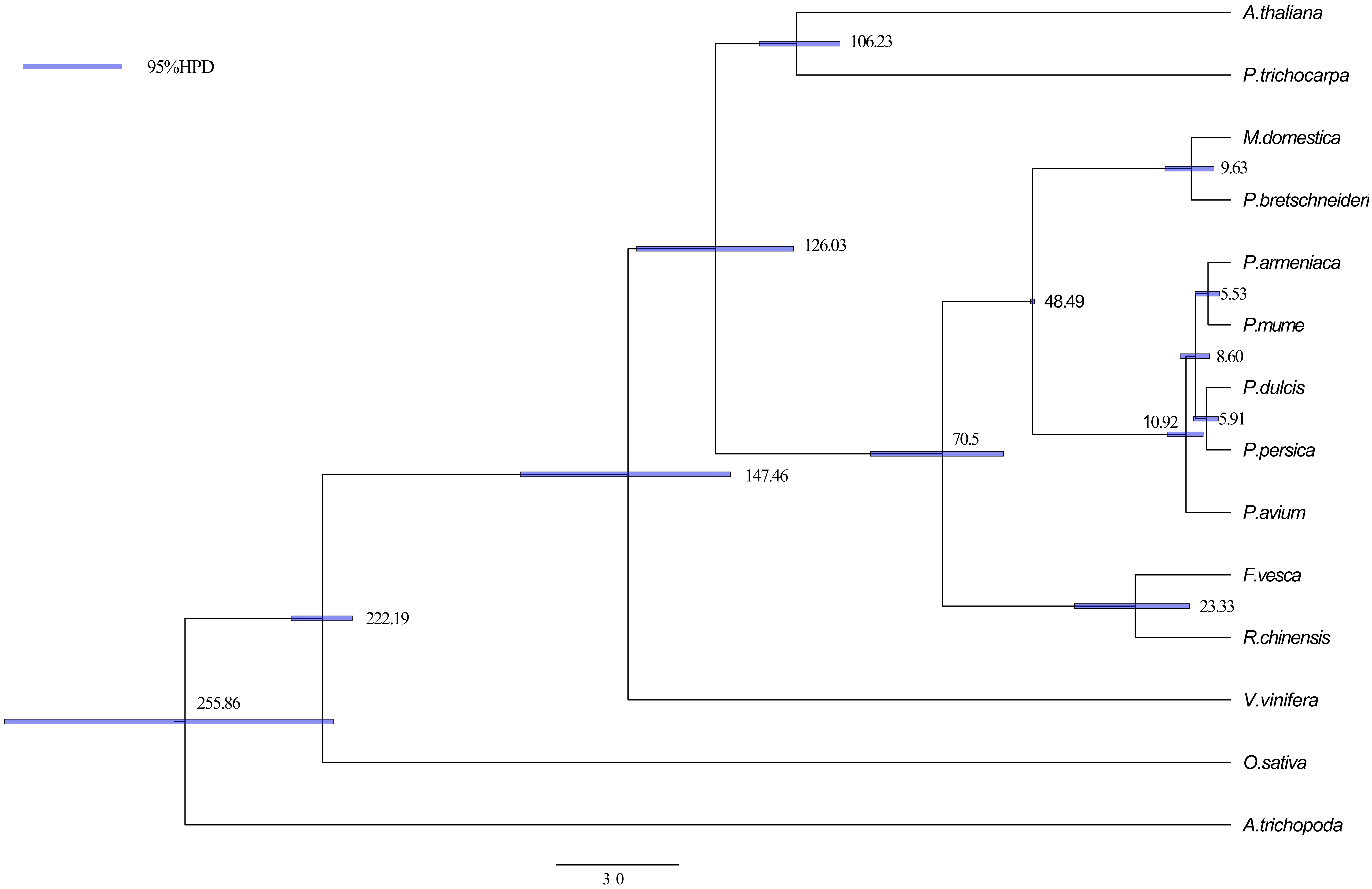

Fig. S6: Divergence time of apricot and related species.

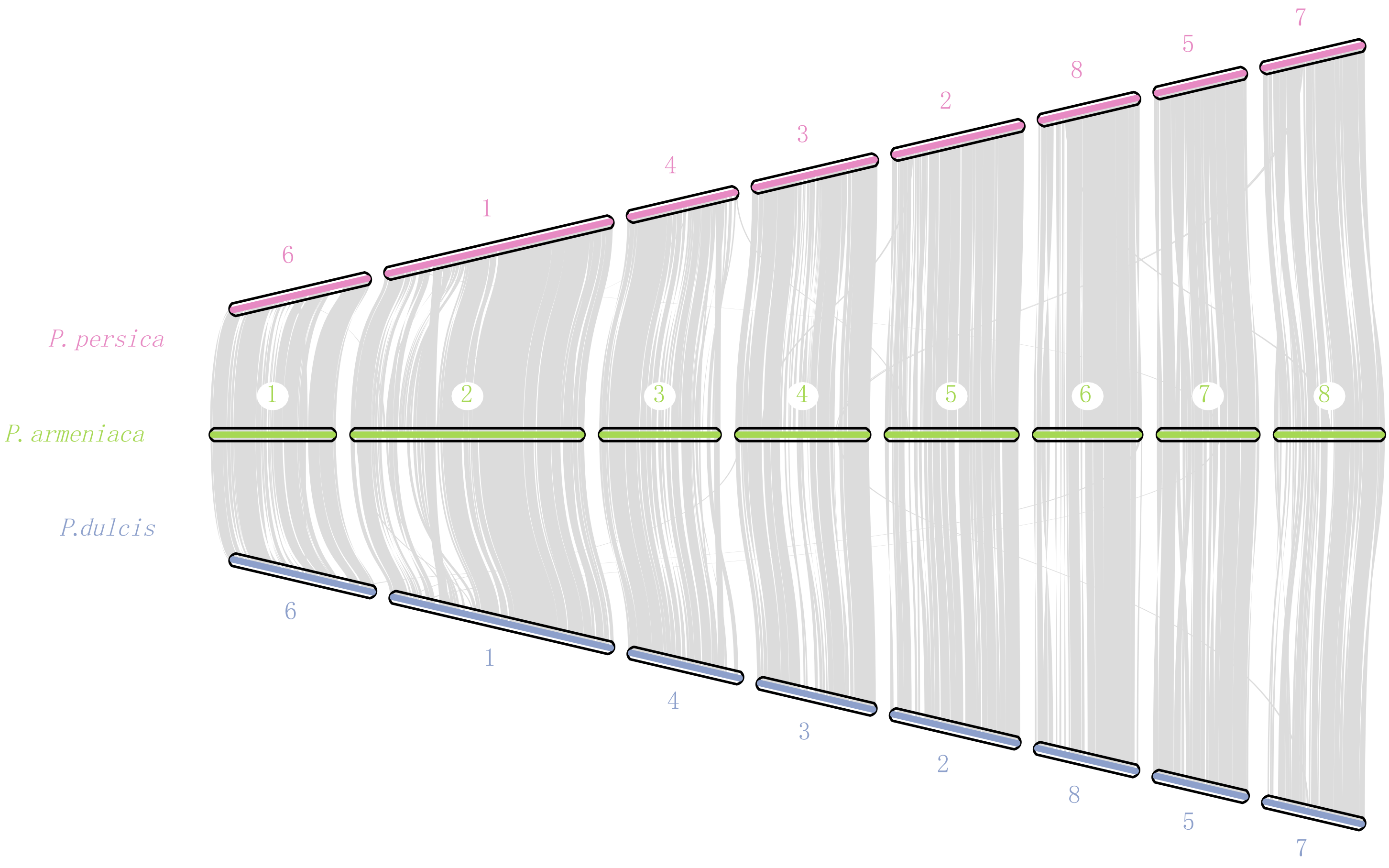

Fig. S7: Collinear genes located in the chromosomes or pseudomolecules of *P .armeniaca*, *P. persica* and *P. dulcis*.

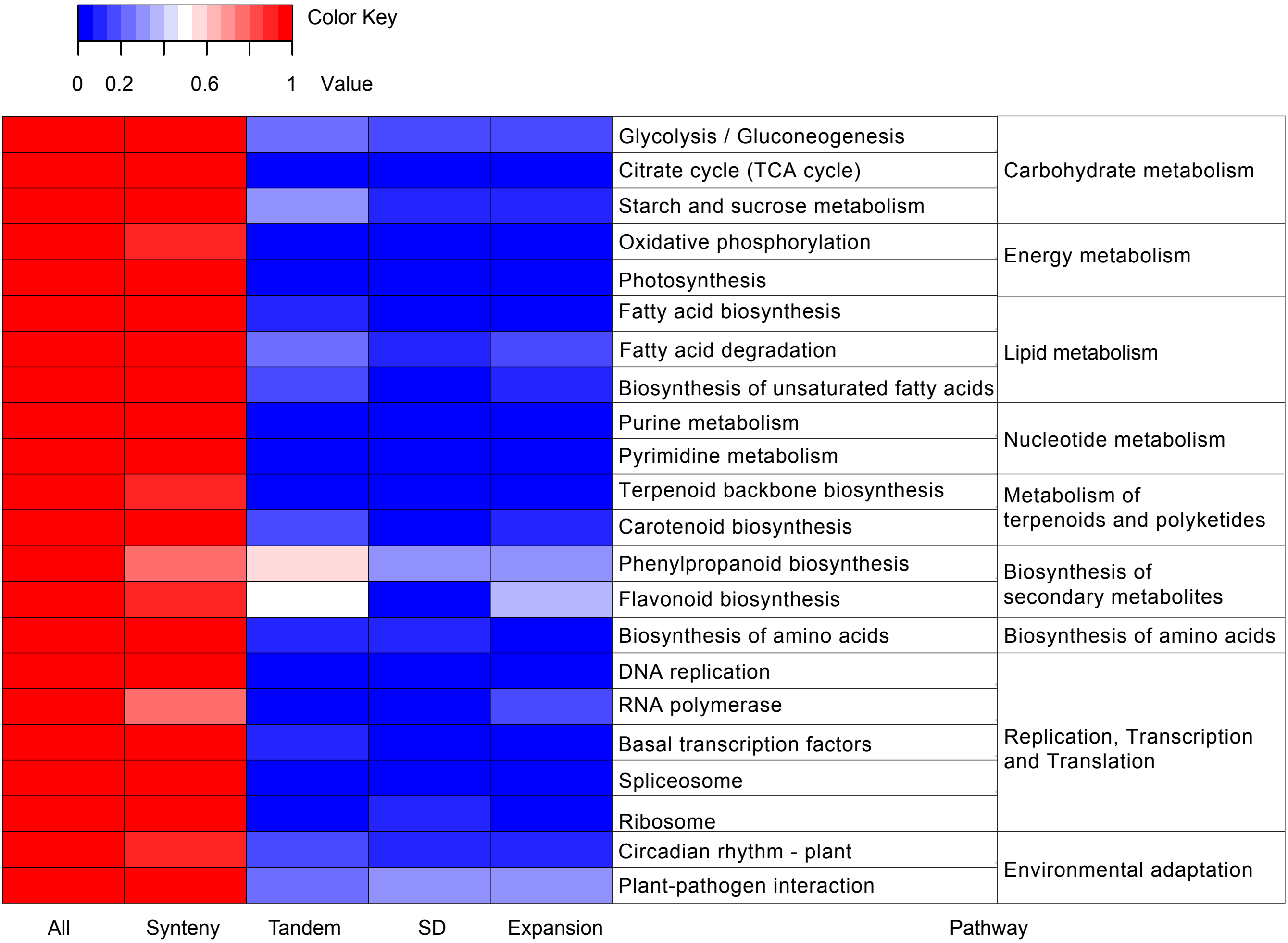

Fig. 8. Heatmap of gene annotation category in different KEGG pathway.
All: total genes; Synteny: genes located in collinearity regions; Tandem: tandem genes;
SD: genes located in segmental duplication (SD) regions;
Expansion: genes from expansion families.
The value was measured using “ALL” gene number as background.

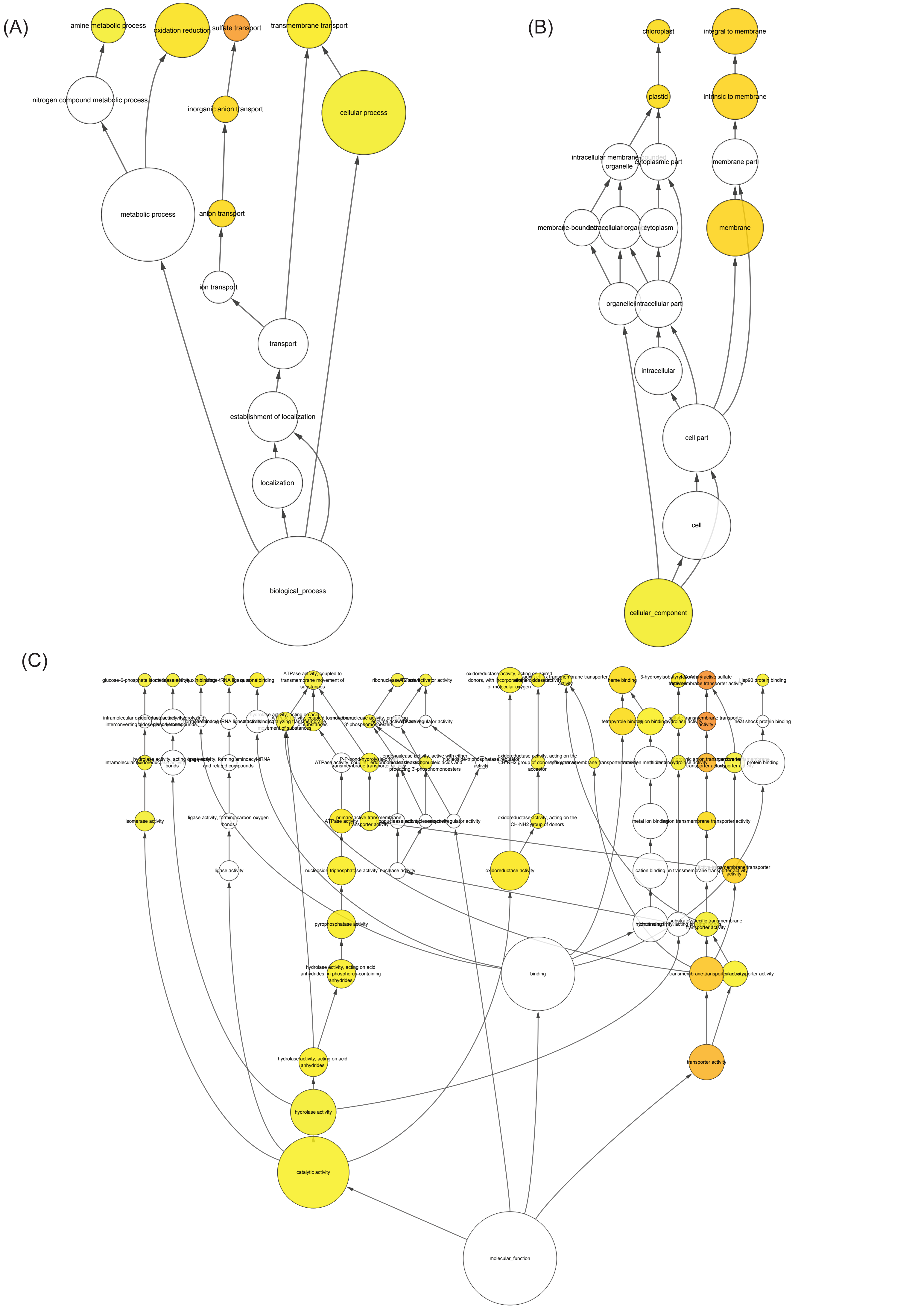

Fig. S9: GO enrichment of apricot specific genes compared with related species at (A) Biological Process, (B) Cellular Component, and (C) Molecular Function.

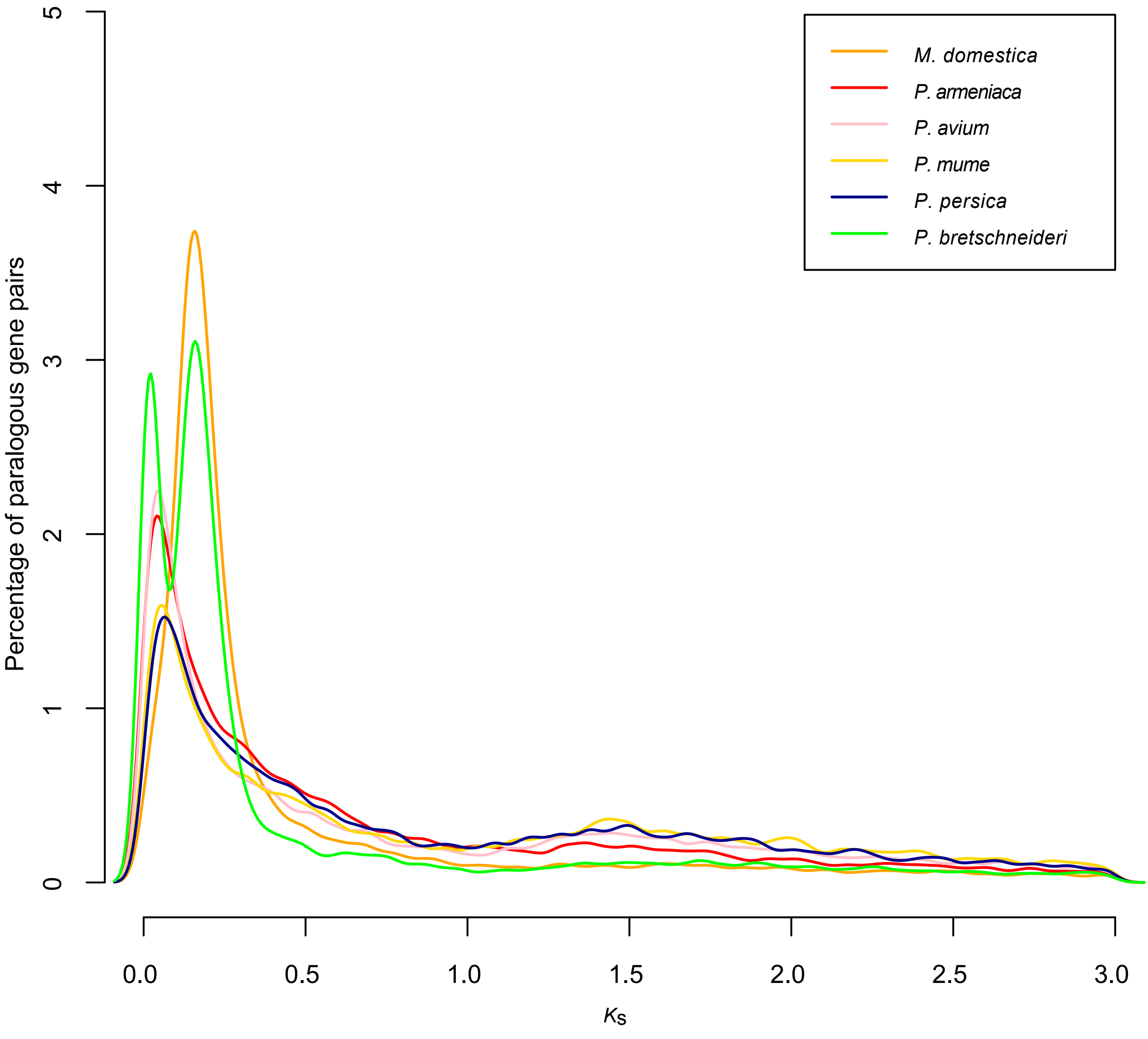

Fig. S10: Distribution of Ks values in paralogous genes *P. armeniaca*, *P. persica*, *P. mume*, *P. avium*, *M. domestica* and *P. bretschneideri*.

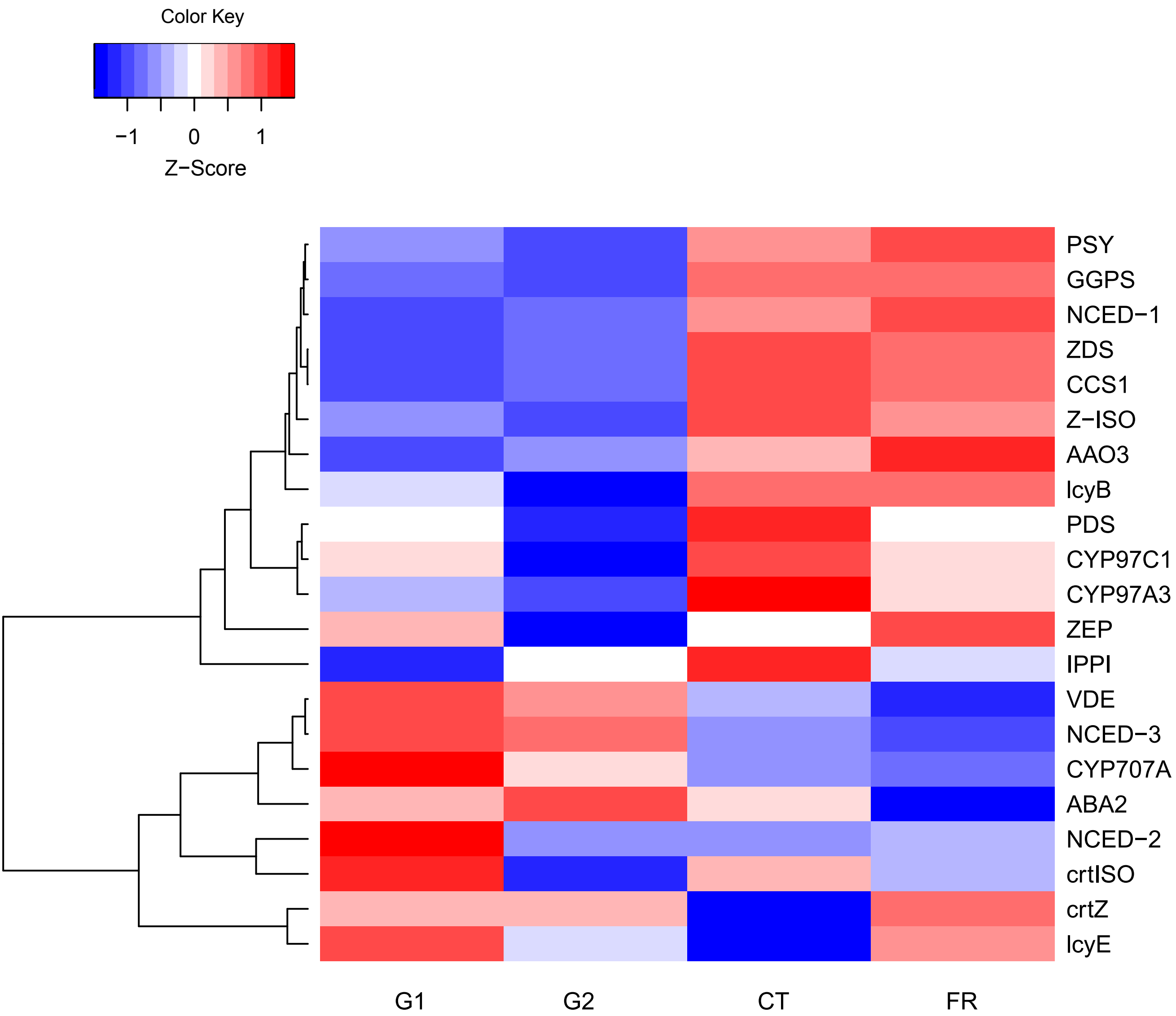

Fig. S11: The heatmap of four developmental stages of apricot.

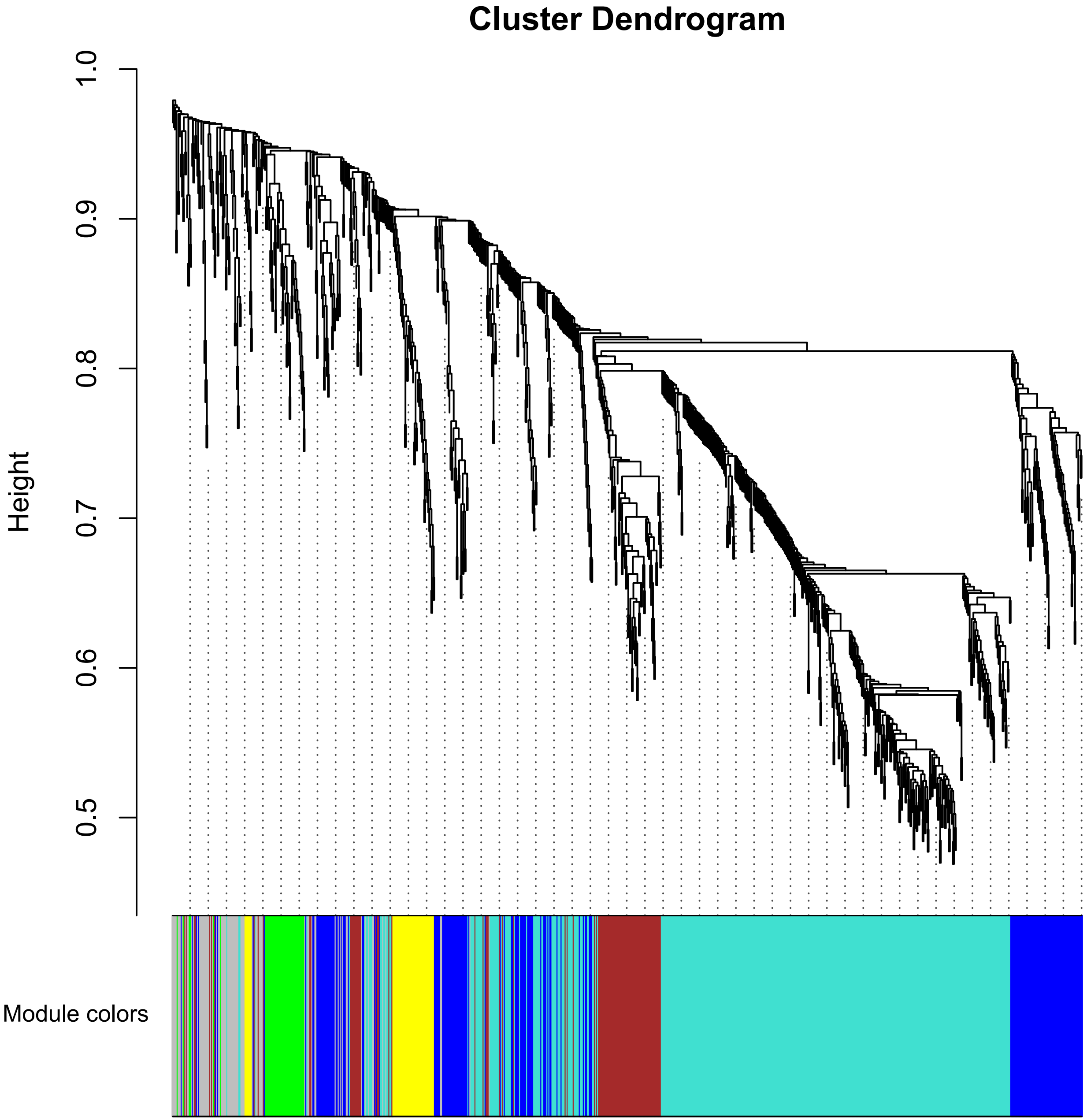

Fig. S12: Co-expression network of the genes involved in carotenoid metabolism and transcription factors. Hierarchical cluster tree showed that genes were comprised of 6 co-expression modules. Carotenoid metabolism related genes were in the "blue" and "turquoise" modules.

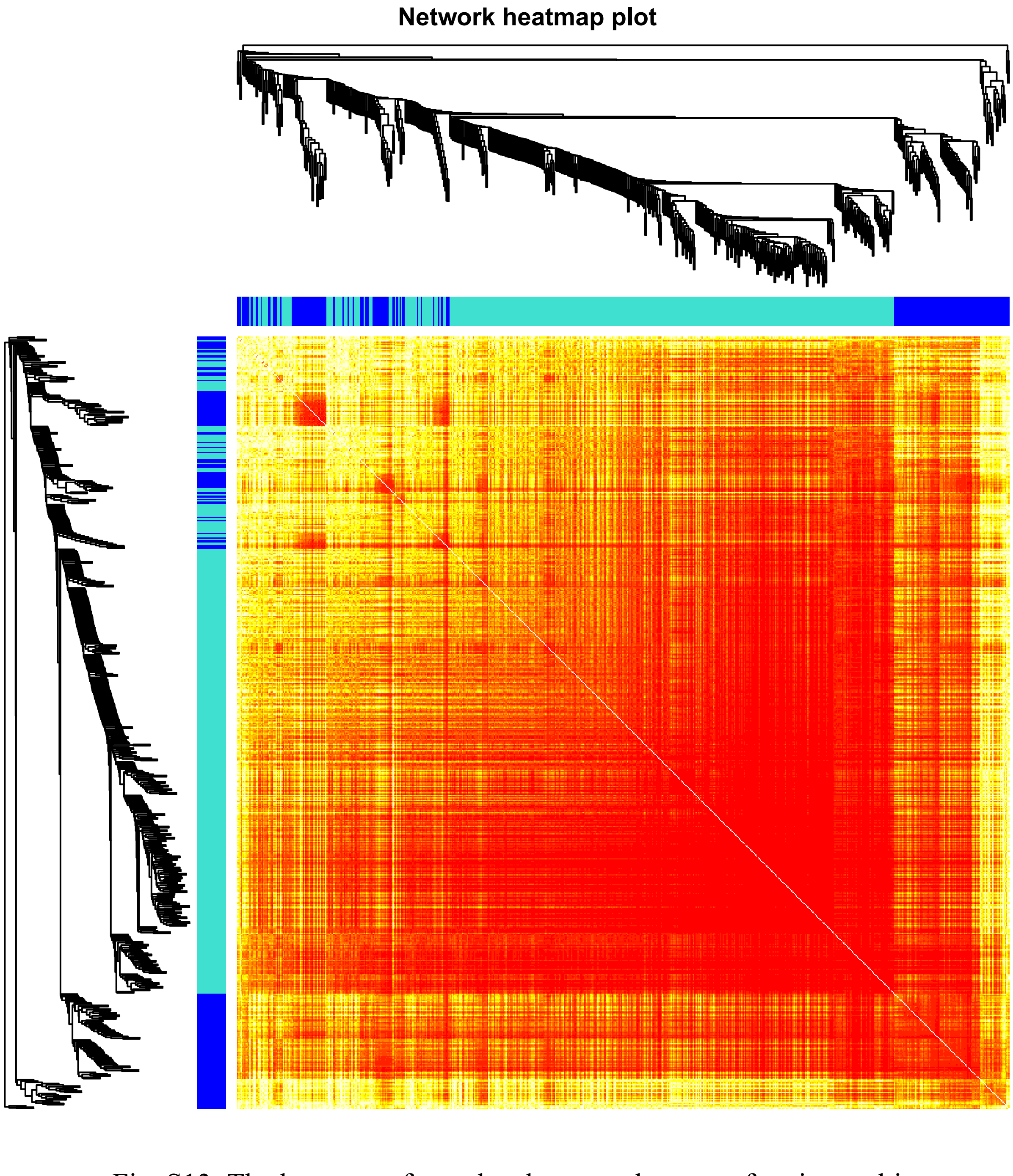

Fig. S13: The heatmap of two developmental stages of apricot cultivars ‘Chuanzhihong’ and ‘Dabaixing’.

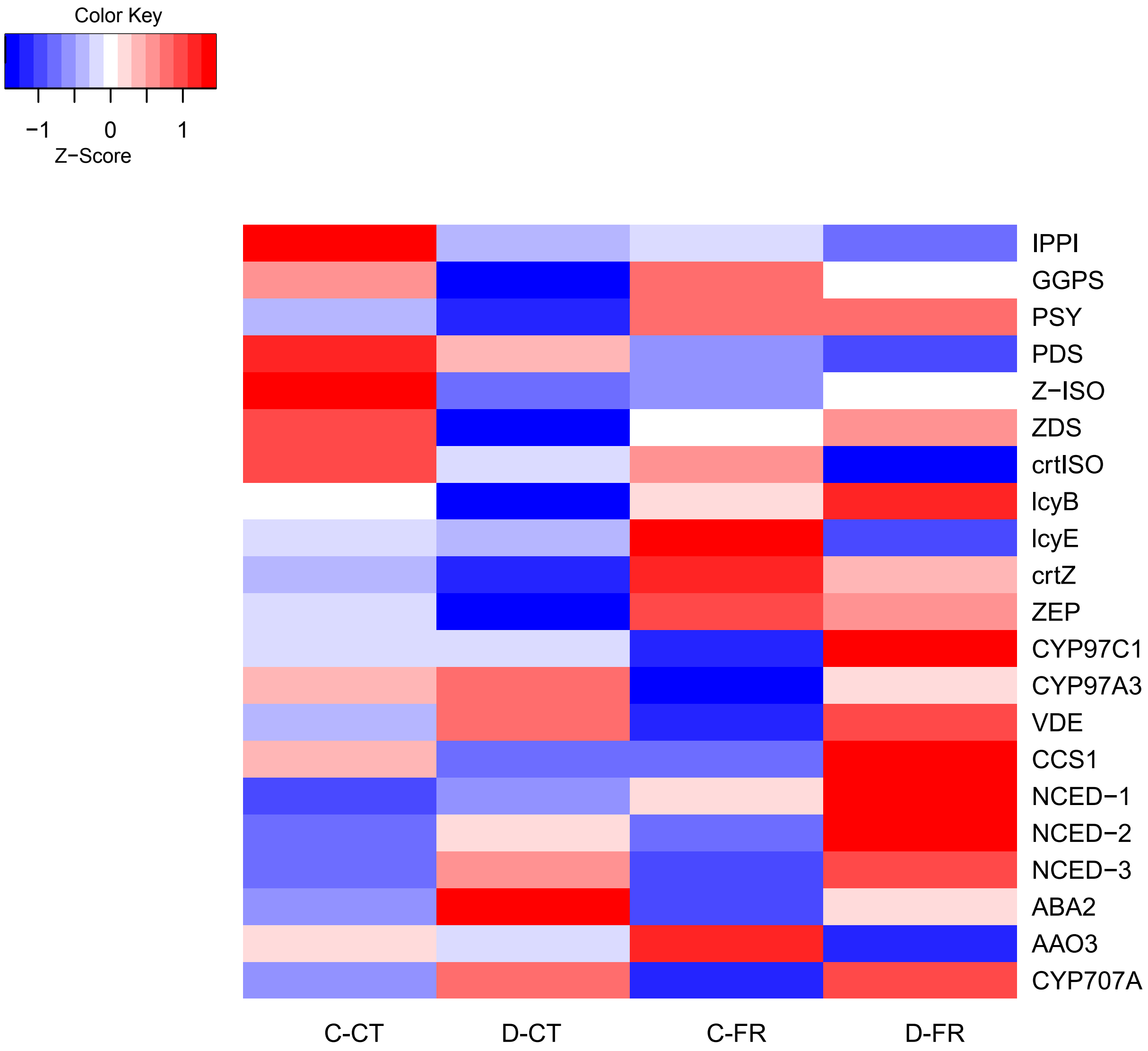

Fig. S14: Heatmap of correlation matrix with module “blue” and “turquoise” in WGCNA.
