# Supplementary material for: The apricot (Prunus armeniaca L.) genome elucidates Rosaceae evolution and beta-carotenoid synthesis
Source: Hortic Res. 2019 Nov 18;6:128. doi: 10.1038/s41438-019-0215-6 (PMC6861294; doi:10.1038/s41438-019-0215-6)
Supplement: Supplementary file 2 — Supplementary Tables [file 41438_2019_215_MOESM2_ESM.pdf]

Table S2. Statistics of Illimina pair-end data of apricot

Insert size (bp)	Read length (bp)	Raw data		Clean data	
		Data (Gb）	Depth (X)	Data (Gb	Depth (X)
150	101	22.47	95	16.75	71
180	101	11.28	48	9.49	40
500	101	6.71	28	5.27	22

Table S3. Summary of *P. armeniaca* pseudomolecules.

LG	Genetics Map	Length (bp)
LG1	95	24,299,982
LG2	144	43,032,007
LG3	100	25,156,918
LG4	120	25,887,085
LG5	89	26,717,257
LG6	92	20,273,904
LG7	83	18,878,021
LG8	100	22,114,339
Total	823	206,359,513

Table S4. The mapping rate of RNA-seq data

Names	Read numbe	Read base	Min len	Avg len	Max len	Mapping rate
CT_C_1	56344490	8451673500	150	150	150	0.9406
CT_C_2	50312412	7546861800	150	150	150	0.9427
CT_C_3	51617510	7742626500	150	150	150	0.9369
CT_D_1	57639448	8645917200	150	150	150	0.9271
CT_D_2	57379366	8606904900	150	150	150	0.9356
CT_D_3	50159744	7523961600	150	150	150	0.9321
FR_C_1	51517198	7727579700	150	150	150	0.9445
FR_C_2	58998280	8849742000	150	150	150	0.9389
FR_C_3	49397262	7409589300	150	150	150	0.9213
FR_D_1	56794676	8519201400	150	150	150	0.9249
FR_D_2	51134496	7670174400	150	150	150	0.9349
FR_D_3	53338330	8000749500	150	150	150	0.9211
G1_C_1	50293814	7544072100	150	150	150	0.9368
G1_C_2	56690592	8503588800	150	150	150	0.9363
G1_C_3	50469862	7570479300	150	150	150	0.9352
G1_D_1	50144268	7521640200	150	150	150	0.9236
G1_D_2	54046046	8106906900	150	150	150	0.9213
G1_D_3	62364486	9354672900	150	150	150	0.9285
G2_C_1	51678408	7751761200	150	150	150	0.9145
G2_C_2	56708484	8506272600	150	150	150	0.9321
G2_C_3	56912328	8536849200	150	150	150	0.9273
G2_D_1	47696100	7154415000	150	150	150	0.9308
G2_D_2	54326782	8149017300	150	150	150	0.9335
G2_D_3	50530132	7579519800	150	150	150	0.9261
bitter	43514792	4382602290	33	100.7	101	0.9454
sweet	43171460	4347730671	33	100.7	101	0.9393
abortion	46448592	4680461925	32	100.8	101	0.9645
norma	45305288	4557297068	32	100.8	101	0.9443

Table S5. The result of BUSCO

classification	counts
Complete BUSCOs (C)	1,347
Complete and single-copy BUSCOs (S)	1,305
Complete and duplicated BUSCOs (D)	42
Fragmented BUSCOs (F)	6
Missing BUSCOs (M)	22
Total BUSCO groups searched	1,375

Table S6. Summary of repeat elements identified in

**Class**	***Prunus armeniaca***		
	**Count**	**Length (bp)**	**Ratio**
LTR	42,697	29,803,110	13.43%
LINE	2,860	300,311	0.14%
SINE	4,954	2,103,384	0.95%
DNA element	59,053	21,077,231	9.50%
Simple Repeat	78,465	3,303,735	1.49%
Unclassified	92,403	27,011,946	12.17%
Total repeat	55,013	84,942,023	38.28%

Table S7. Statistics of gene annotation

	P. armeniaca
Genome contig number	182
Genome size (bp)	221,901,797
Percent of genome of genes (%)	56.31
Gene number	30436
Gene total length	124,959,081
Gene average length	4,105
Gene density	137/Mb
Average coding sequence length	1,641
All exons	316,496
Average exon length (bp)	270
Exon GC content (%)	43.45
All introns	264,398
Average intron length (bp)	530
Intron GC content (%)	35.06

Table S8. Non-coding RNAs in the *P. armeniaca* genome

Kind	*P. armeniaca*
**Ribosomal RNAs**	
5_8S_rRNA	101
5S_rRNA	624
18S_rRNA	95
28S_RNA	85
**tRNAs**	
tRNA	488
**Small nucleolar RNA (snRNA)**	
snoRNA	353
**spliceosomal RNA**	
U1	27
U2	29
U3	21
U4	11
U5	18
U6	28
U11	2
U12	1
**RNA component of signal-recognition particle (SRP)**	
Plant_SRP	7
**nuclear ribonuclease P (RNase P)**	
RNase_MRP	1
**MicroRNAs**	
miRNA	278

Table S9. Function annotation of *P. armeniaca*

	Gene Numbe	Percent (%)
Nr	30,184	99.17%
Pfam	26,198	86.07%
KEGG	13,211	43.40%
GO	16,615	54.59%
Uniprot	21,772	71.53%
TMHMM	7,156	23.51%
Total genes	30,436	100.00%

Table S10 Transcription factors of *P. armeniaca*

Gene ID	Calss	Function
PARG00096	RAV	RAV family protein
PARG00093	GATA	GATA transcription factor 29
PARG00125	EIL	ETHYLENE-INSENSITIVE3-like 3
PARG00171	bHLH	bHLH family protein
PARG00312	WRKY	WRKY DNA-binding protein 2
PARG00318	GRAS	GRAS family protein
PARG00355	bZIP	bZIP family protein
PARG00378	LBD	LOB domain-containing protein 13
PARG00404	WRKY	WRKY DNA-binding protein 33
PARG00429	LBD	LBD family protein
PARG00484	NAC	NAC domain containing protein 83
PARG00527	bZIP	bZIP family protein
PARG00654	GRAS	GRAS family protein
PARG00657	GRAS	SCARECROW-like 14
PARG00658	GRAS	GRAS family protein
PARG00659	GRAS	SCARECROW-like 14
PARG00660	GRAS	SCARECROW-like 14
PARG00661	GRAS	SCARECROW-like 14
PARG00662	GRAS	SCARECROW-like 14
PARG00861	Dof	Dof family protein
PARG00880	LBD	LOB domain-containing protein 11
PARG00918	NAC	Arabidopsis NAC domain containing protein 87
PARG00919	NAC	NAC domain containing protein 100
PARG00921	NAC	NAC domain containing protein 25
PARG00929	NF-YC	nuclear factor Y, subunit C10
PARG00958	GATA	GATA transcription factor 10
PARG00966	NAC	NAC domain containing protein 5
PARG00967	NAC	NAC domain containing protein 5
PARG01055	NAC	NAC domain containing protein 83
PARG01060	NAC	NAC domain containing protein 50
PARG01067	bHLH	FER-like regulator of iron uptake

PARG01068	bHLH	FER-like regulator of iron uptake
PARG01076	NAC	NAC domain containing protein 83
PARG01077	NAC	NAC domain containing protein 50
PARG01190	NAC	NAC domain containing protein 90
PARG01194	NAC	NAC family protein
PARG01238	NF-YB	nuclear factor Y, subunit B11
PARG01251	bZIP	bZIP family protein
PARG01253	bZIP	bZIP family protein
PARG01385	NAC	NTM1-like 8
PARG01469	FAR1	FAR1-related sequence 5
PARG01507	YABBY	YABBY family protein
PARG01559	GRAS	SCARECROW-like 21
PARG01572	FAR1	FAR1 family protein
PARG01601	bHLH	bHLH family protein
PARG01652	bZIP	ABA-responsive element binding protein 3
PARG01683	WRKY	WRKY DNA-binding protein 13
PARG01780	EIL	EIL family protein
PARG01781	EIL	EIL family protein
PARG01901	bHLH	bHLH family protein
PARG01916	ZF-HD	homeobox protein 22
PARG01991	Dof	Dof family protein
PARG02042	bZIP	basic leucine-zipper 6
PARG02071	GRAS	GRAS family protein
PARG02092	LBD	LOB domain-containing protein 38
PARG02093	GRAS	GRAS family protein
PARG02169	WRKY	WRKY DNA-binding protein 7
PARG02200	TCP	TCP family protein
PARG02241	NAC	NAC domain containing protein 25
PARG02253	S1Fa-like	S1FA-like DNA-binding protein
PARG02275	NF-YB	nuclear factor Y, subunit B8
PARG02307	WRKY	WRKY family protein
PARG02410	E2F/DP	Transcription factor DP
PARG02411	Dof	DNA binding with one finger 2.4
PARG02441	SBP	squamosa promoter binding protein-like 9

PARG02448 GRAS	GRAS family protein
PARG02452 WRKY	WRKY DNA-binding protein 49
PARG02523 WRKY	WRKY DNA-binding protein 51
PARG02580 NF-YB	nuclear factor Y, subunit B3
PARG02594 NF-YB	nuclear factor Y, subunit B3
PARG02620 bZIP	bZIP family protein
PARG02695 GRAS	GRAS family protein
PARG02718 bHLH	phytochrome interacting factor 3
PARG02836 GATA	GATA transcription factor 17
PARG02901 NF-YB	nuclear factor Y, subunit B4
PARG02937 NF-YC	nuclear factor Y, subunit C9
PARG02947 bHLH	bHLH family protein
PARG03040 GeBP	DNA-binding storekeeper protein-related transcriptional regulator
PARG03041 GeBP	DNA-binding storekeeper protein-related transcriptional regulator
PARG03073 Trihelix	Trihelix family protein
PARG03089 bZIP	bZIP family protein
PARG03092 bHLH	bHLH family protein
PARG03107 WRKY	WRKY DNA-binding protein 74
PARG03189 E2F/DP	DP-E2F-like 1
PARG03258 LBD	LBD family protein
PARG03273 FAR1	FAR1 family protein
PARG03298 WRKY	WRKY DNA-binding protein 2
PARG03365 bHLH	bHLH family protein
PARG03469 CPP	Tesmin/TSO1-like CXC domain-containing protein
PARG03486 HSF	heat shock transcription factor A6B
PARG03658 bHLH	bHLH family protein
PARG03881 TCP	TCP family protein
PARG03931 NAC	NAC domain containing protein 50
PARG03967 bHLH	bHLH family protein
PARG03993 WRKY	WRKY DNA-binding protein 11
PARG04002 TCP	TCP family protein
PARG04144 NAC	NAC-like, activated by AP3/PI
PARG04257 Trihelix	Trihelix family protein
PARG04349 CAMTA	signal responsive 1

PARG04352	NAC	NAC domain containing protein 64
PARG04374	NAC	NAC domain containing protein 11
PARG04377	NAC	NAC domain containing protein 64
PARG04493	WRKY	WRKY DNA-binding protein 48
PARG04526	GRAS	RGA-like 2
PARG04527	GRAS	RGA-like 2
PARG04567	CAMTA	calmodulin binding;transcription regulators
PARG04578	GATA	GATA transcription factor 15
PARG04662	FAR1	FAR1 family protein
PARG04676	FAR1	FAR1 family protein
PARG04680	FAR1	FAR1 family protein
PARG04692	FAR1	FAR1 family protein
PARG04693	FAR1	FAR1 family protein
PARG04696	FAR1	FAR1 family protein
PARG04757	Nin-like	Nin-like family protein
PARG04811	GRAS	GRAS family protein
PARG04844	bZIP	bZIP family protein
PARG04887	bZIP	HY5-homolog
PARG04972	Trihelix	Trihelix family protein
PARG05002	bHLH	bHLH family protein
PARG05071	E2F/DP	E2F transcription factor 1
PARG05190	bHLH	bHLH family protein
PARG05319	bHLH	bHLH family protein
PARG05433	HSF	HSF family protein
PARG05492	NF-YB	nuclear factor Y, subunit B5
PARG05544	SBP	squamosa promoter binding protein-like 7
PARG05630	NF-YC	nuclear factor Y, subunit C9
PARG05702	STAT	SH2 domain protein B
PARG05704	NAC	NAC domain protein 66
PARG05706	NAC	NAC domain protein 66
PARG05756	NAC	NAC domain containing protein 83
PARG05771	NF-YC	nuclear factor Y, subunit C13
PARG05785	SBP	squamosa promoter binding protein-like 2
PARG05795	CAMTA	Calmodulin-binding transcription activator protein with CG-1 and Ankyrin domains

PARG05801 SBP	SBP family protein
PARG05805 WRKY	WRKY DNA-binding protein 75
PARG05816 TCP	TCP family protein
PARG00915 ARF	auxin response factor 4
PARG00957 ARF	auxin response factor 10
PARG02222 ARF	auxin response factor 16
PARG03914 ARF	auxin response factor 19
PARG00335 B3	B3 family protein
PARG00351 B3	HSI2-like 1
PARG02158 B3	related to vernalization1 1
PARG05735 B3	B3 family protein
PARG00183 AP2	AP2 family protein
PARG00843 AP2	related to AP2.7
PARG01970 AP2	AINTEGUMENTA-like 6
PARG02036 AP2	AP2 family protein
PARG02179 AP2	AP2 family protein
PARG02280 AP2	related to AP2.7
PARG03274 AP2	AP2 family protein
PARG04104 AP2	AINTEGUMENTA-like 5
PARG04421 AP2	ARIA-interacting double AP2 domain protein
PARG00066 ERF	related to AP2 11
PARG00343 ERF	cytokinin response factor 4
PARG00570 ERF	ethylene responsive element binding factor 1
PARG01733 ERF	ERF family protein
PARG01786 ERF	related to AP2 4
PARG02177 ERF	DREB and EAR motif protein 2
PARG03138 ERF	ethylene response factor 1
PARG03190 ERF	ERF family protein
PARG03325 ERF	ERF family protein
PARG03384 ERF	ERF family protein
PARG03661 ERF	ERF family protein
PARG03662 ERF	ERF family protein
PARG03663 ERF	ethylene response factor 1
PARG04103 ERF	related to AP2 11

PARG04928	ERF	ERF family protein
PARG04962	ERF	related to AP2 6l
PARG05504	ERF	ERF family protein
PARG05751	DBB	B-box type zinc finger protein with CCT domain
PARG04581	ARR-B	response regulator 2
PARG00643	G2-like	G2-like family protein
PARG02426	G2-like	response regulator 2
PARG02428	G2-like	response regulator 21
PARG03159	G2-like	G2-like family protein
PARG03368	G2-like	G2-like family protein
PARG01939	MIKC_MADS	MIKC_MADS family protein
PARG05800	MIKC_MADS	MIKC_MADS family protein
PARG01127	M-type_MADS	AGAMOUS-like 103
PARG01148	M-type_MADS	AGAMOUS-like 61
PARG01149	M-type_MADS	AGAMOUS-like 62
PARG01321	M-type_MADS	AGAMOUS-like 62
PARG01348	M-type_MADS	AGAMOUS-like 103
PARG02013	M-type_MADS	AGAMOUS-like 61
PARG02721	M-type_MADS	AGAMOUS-like 80
PARG03771	M-type_MADS	MIKC_MADS family protein
PARG03802	M-type_MADS	MIKC_MADS family protein
PARG04132	M-type_MADS	AGAMOUS-like 64
PARG05375	M-type_MADS	AGAMOUS-like 36
PARG05377	M-type_MADS	AGAMOUS-like 36
PARG02040	GRF	growth-regulating factor 1
PARG00826	WOX	WOX family protein
PARG00726	WOX	WUSCHEL related homeobox 2
PARG01473	TALE	BEL1-like homeodomain 1
PARG00979	TALE	BEL1-like homeodomain 8
PARG01888	HD-ZIP	homeobox protein 6
PARG02005	HD-ZIP	HD-ZIP family protein
PARG03857	HD-ZIP	HD-ZIP family protein
PARG03889	HD-ZIP	HD-ZIP family protein
PARG00953	HD-ZIP	HD-ZIP family protein

PARG03960 HB-PHD	pathogenesis related homeodomain protein A
PARG00089 C2H2	C2H2-like zinc finger protein
PARG00090 C2H2	C2H2-like zinc finger protein
PARG00094 C3H	C3H family protein
PARG00191 C2H2	C2H2 family protein
PARG00301 C2H2	C2H2 family protein
PARG00458 C2H2	C2H2 family protein
PARG00496 C3H	C3H family protein
PARG00568 C2H2	indeterminate(ID)-domain 14
PARG00631 C2H2	C2H2 family protein
PARG00754 C2H2	C2H2 family protein
PARG00872 C2H2	zinc-finger protein 2
PARG00874 C2H2	C2H2 family protein
PARG00922 C2H2	C2H2-like zinc finger protein
PARG01081 C2H2	C2H2 family protein
PARG01263 C2H2	WIP domain protein 3
PARG01602 C3H	C3H family protein
PARG01849 C2H2	C2H2 family protein
PARG02240 C2H2	C2H2 family protein
PARG02287 C3H	C3H family protein
PARG02376 C2H2	C2H2 family protein
PARG02479 C2H2	C2H2 family protein
PARG02480 C2H2	zinc-finger protein 10
PARG02745 C3H	C3H family protein
PARG02898 C2H2	indeterminate(ID)-domain 2
PARG03304 C3H	zinc finger protein 1
PARG03311 C3H	C3H family protein
PARG03628 C2H2	C2H2 family protein
PARG03629 C2H2	C2H2 family protein
PARG03695 C2H2	zinc finger protein 2
PARG04135 C2H2	zinc finger protein 2
PARG04575 C2H2	C2H2 family protein
PARG05503 C3H	C3H family protein
PARG00063 MYB	myb domain protein 5

PARG00461	MYB	myb domain protein 119
PARG00761	MYB	myb domain protein 48
PARG01029	MYB	myb domain protein 36
PARG01847	MYB	myb domain protein 93
PARG02019	MYB	myb domain protein 84
PARG02152	MYB	myb domain protein 73
PARG02431	MYB	myb domain protein 3r-5
PARG02437	MYB	MYB family protein
PARG02603	MYB	myb domain protein 113
PARG02605	MYB	myb domain protein 113
PARG02716	MYB	myb domain protein 61
PARG03145	MYB	cell division cycle 5
PARG03204	MYB	myb domain protein 113
PARG03679	MYB	myb domain protein 14
PARG03797	MYB	myb domain protein 305
PARG03798	MYB	myb domain protein 305
PARG04152	MYB	myb domain protein 36
PARG04468	MYB	myb domain protein 108
PARG04573	MYB	myb domain protein 111
PARG00100	MYB_related	DNA-binding bromodomain-containing protein
PARG00282	MYB_related	myb domain protein 103
PARG00358	MYB_related	TRF-like 7
PARG00449	MYB_related	MYB family protein
PARG00541	MYB_related	MYB family protein
PARG00663	MYB_related	TRF-like 2
PARG00987	MYB_related	TRF-like 6
PARG02063	MYB_related	MYB_related family protein
PARG02189	MYB_related	TSL-kinase interacting protein 1
PARG02212	MYB_related	TRF-like 6
PARG02285	MYB_related	MYB_related family protein
PARG02447	MYB_related	TRF-like 2
PARG02607	MYB_related	myb domain protein 113
PARG02904	MYB_related	DNA binding
PARG03143	MYB_related	myb domain protein 15

PARG05501	MYB_related	DNA binding
PARG06628	RAV	RAV family protein
PARG05904	Trihelix	Trihelix family protein
PARG05962	GRAS	RGA-like 1
PARG06098	YABBY	YABBY family protein
PARG06119	bHLH	bHLH family protein
PARG06229	bHLH	BIG PETAL P
PARG06284	Whirly	ssDNA-binding transcriptional regulator
PARG06285	WRKY	WRKY DNA-binding protein 72
PARG06312	bHLH	bHLH family protein
PARG06319	TCP	TEOSINTE BRANCHED, cycloidea and PCF (TCP) 14
PARG06339	NAC	NAC family protein
PARG06343	ZF-HD	zinc finger homeodomain 1
PARG06357	Dof	cycling DOF factor 2
PARG06423	YABBY	YABBY family protein
PARG06424	SBP	SBP family protein
PARG06464	bHLH	BES1-interacting Myc-like protein 2
PARG06510	WRKY	WRKY DNA-binding protein 3
PARG06540	WRKY	WRKY DNA-binding protein 57
PARG06548	NAC	NAC-like, activated by AP3/PI
PARG06549	NAC	NAC-like, activated by AP3/PI
PARG06611	TCP	TCP domain protein 12
PARG06613	bHLH	bHLH family protein
PARG06621	NAC	NAC family protein
PARG06644	bZIP	bZIP family protein
PARG06680	LBD	LOB domain-containing protein 41
PARG06698	bHLH	bHLH family protein
PARG06724	bHLH	phytochrome interacting factor 3-like 5
PARG06835	GRAS	GRAS family protein
PARG06890	HSF	heat shock transcription factor A8
PARG06918	BBR-BPC	basic pentacysteine1
PARG06944	bHLH	bHLH family protein
PARG07123	bZIP	bZIP family protein
PARG07168	E2F/DP	E2F/DP family protein

PARG07187	bZIP	bZIP family protein
PARG07210	SRS	SHI-related sequence 5
PARG07251	bZIP	basic leucine-zipper 44
PARG07257	bHLH	bHLH family protein
PARG07327	NAC	NAC domain containing protein 28
PARG07376	Dof	OBF binding protein 1
PARG07414	WRKY	WRKY DNA-binding protein 18
PARG07513	Trihelix	Trihelix family protein
PARG07529	Nin-like	Nin-like family protein
PARG07544	WRKY	WRKY DNA-binding protein 50
PARG07570	HSF	heat shock transcription factor A2
PARG07578	LBD	LOB domain-containing protein 22
PARG07588	GATA	cytokinin-responsive gata factor 1
PARG07643	bHLH	bHLH family protein
PARG07652	bZIP	basic leucine-zipper 4
PARG07689	bHLH	ROOT HAIR DEFECTIVE 6-LIKE 2
PARG07692	LBD	LOB domain-containing protein 39
PARG07759	WRKY	WRKY DNA-binding protein 7
PARG07771	NAC	NAC family protein
PARG07773	bHLH	bHLH family protein
PARG07774	bHLH	bHLH family protein
PARG07781	HSF	heat shock transcription factor B4
PARG07785	bHLH	bHLH family protein
PARG07789	bZIP	abscisic acid responsive elements-binding factor 2
PARG07929	CPP	Tesmin/TSO1-like CXC domain-containing protein
PARG07963	NAC	NAC domain containing protein 38
PARG07967	bZIP	basic leucine zipper 9
PARG07975	Trihelix	sequence-specific DNA binding transcription factors
PARG07994	WRKY	WRKY DNA-binding protein 11
PARG08132	CPP	TESMIN/TSO1-like CXC 2
PARG08161	WRKY	WRKY DNA-binding protein 9
PARG08220	bZIP	bZIP family protein
PARG08347	NAC	NAC family protein
PARG08415	GRAS	GRAS family protein

PARG08475 bZIP	bZIP family protein
PARG08477 bZIP	ABA-responsive element binding protein 3
PARG08623 bHLH	bHLH family protein
PARG08649 ZF-HD	homeobox protein 22
PARG08685 bHLH	basic helix-loop-helix 32
PARG08716 GATA	GATA-type zinc finger protein with TIFY domain
PARG08867 bHLH	bHLH family protein
PARG08924 NAC	xylem NAC domain 1
PARG08948 bZIP	bZIP family protein
PARG09010 bHLH	beta HLH protein 93
PARG09151 bZIP	bZIP family protein
PARG09157 TCP	TCP family protein
PARG09205 NAC	NAC domain containing protein 86
PARG09207 NAC	NAC domain containing protein 86
PARG09216 FAR1	FAR1-related sequence 11
PARG09248 ZF-HD	homeobox protein 31
PARG09263 NAC	NAC domain containing protein 35
PARG09309 NAC	NAC domain containing protein 32
PARG09312 FAR1	FAR1-related sequence 5
PARG09364 NF-YA	nuclear factor Y, subunit A1
PARG09408 NAC	NAC-domain protein 101
PARG09435 Trihelix	sequence-specific DNA binding transcription factors
PARG09452 NF-YB	nuclear factor Y, subunit B3
PARG09557 SRS	Lateral root primordium (LRP) protein-related
PARG09622 NAC	NAC family protein
PARG09715 NF-YB	nuclear factor Y, subunit B7
PARG10075 GRAS	scarecrow-like 3
PARG10247 GRAS	GRAS family protein
PARG10248 WRKY	WRKY family protein
PARG10262 NAC	NAC-like, activated by AP3/PI
PARG10264 NAC	NAC family protein
PARG10282 WRKY	WRKY family protein
PARG10336 WRKY	WRKY family protein
PARG10338 WRKY	WRKY family protein

PARG10439 FAR1	FAR1-related sequence 5
PARG10518 GRAS	GRAS family protein
PARG10578 bHLH	bHLH family protein
PARG10614 NZZ/SPL	sporocyteless (SPL)
PARG10668 NAC	NAC domain containing protein 2
PARG10669 NAC	NAC family protein
PARG10753 SBP	squamosa promoter binding protein-like 4
PARG10766 NAC	NAC family protein
PARG10864 bZIP	bZIP family protein
PARG10874 NZZ/SPL	sporocyteless (SPL)
PARG10875 TCP	TCP family protein
PARG11014 BES1	BES1/BZR1 homolog 4
PARG11123 NAC	NAC domain protein 66
PARG11125 NAC	NAC domain protein 66
PARG11141 NAC	NAC family protein
PARG11143 NAC	NAC domain protein 66
PARG11155 HSF	heat shock factor 3
PARG11160 NAC	NAC domain containing protein 1
PARG11238 NAC	NAC family protein
PARG11240 LBD	LOB domain-containing protein 4
PARG11275 bHLH	bHLH family protein
PARG11298 NF-YC	nuclear factor Y, subunit C2
PARG11300 GRAS	RGA-like 1
PARG11372 NAC	NAC family protein
PARG11400 NAC	NAC domain containing protein 20
PARG07177 ARF	ARF family protein
PARG08462 ARF	auxin response factor 17
PARG09241 ARF	auxin response factor 1
PARG07371 B3	B3 family protein
PARG07372 B3	B3 family protein
PARG07373 B3	B3 family protein
PARG07818 B3	B3 family protein
PARG07819 B3	B3 family protein
PARG07820 B3	B3 family protein

PARG08562	B3	B3 family protein
PARG09001	B3	B3 family protein
PARG09130	B3	auxin response factor 17
PARG09132	B3	related to ABI3/VP1 2
PARG09183	B3	B3 family protein
PARG10454	B3	B3 family protein
PARG10743	B3	B3 family protein
PARG10744	B3	B3 family protein
PARG10745	B3	B3 family protein
PARG10746	B3	B3 family protein
PARG10747	B3	B3 family protein
PARG06281	AP2	AP2 family protein
PARG07644	AP2	AP2 family protein
PARG06049	ERF	DORNROSCHEN-like
PARG06672	ERF	ERF family protein
PARG07216	ERF	ERF family protein
PARG07396	ERF	ERF family protein
PARG07767	ERF	DREB and EAR motif protein 3
PARG07913	ERF	ERF family protein
PARG08234	ERF	ERF family protein
PARG08409	ERF	ERF family protein
PARG08527	ERF	related to AP2 4
PARG08766	ERF	ERF family protein
PARG08769	ERF	ERF family protein
PARG10301	ERF	ethylene responsive element binding factor 3
PARG10762	ERF	ethylene responsive element binding factor 4
PARG07207	DBB	salt tolerance homolog2
PARG10976	DBB	light-regulated zinc finger protein 1
PARG06678	CO-like	B-box type zinc finger protein with CCT domain
PARG07458	CO-like	CONSTANS-like 4
PARG05976	ARR-B	response regulator 11
PARG06738	ARR-B	pseudo-response regulator 6
PARG06879	ARR-B	response regulator 2
PARG07402	ARR-B	response regulator 12

PARG06354	G2-like	G2-like family protein
PARG06632	G2-like	G2-like family protein
PARG06936	G2-like	G2-like family protein
PARG07369	G2-like	G2-like family protein
PARG10069	G2-like	G2-like family protein
PARG11410	G2-like	G2-like family protein
PARG06428	MIKC_MADS	MIKC_MADS family protein
PARG06429	MIKC_MADS	MIKC_MADS family protein
PARG07208	MIKC_MADS	MIKC_MADS family protein
PARG08500	MIKC_MADS	AGAMOUS-like 104
PARG08688	MIKC_MADS	MIKC_MADS family protein
PARG08689	MIKC_MADS	AGAMOUS-like 24
PARG08690	MIKC_MADS	AGAMOUS-like 24
PARG08793	MIKC_MADS	AGAMOUS-like 12
PARG08990	MIKC_MADS	AGAMOUS-like 16
PARG06361	M-type_MADS	AGAMOUS-like 30
PARG07724	M-type_MADS	AGAMOUS-like 104
PARG08709	M-type_MADS	AGAMOUS-like 80
PARG08863	M-type_MADS	AGAMOUS-like 87
PARG09945	M-type_MADS	AGAMOUS-like 61
PARG07647	GRF	growth-regulating factor 1
PARG08219	HB-other	overexpressor of cationic peroxidase 3
PARG09562	HB-other	HB-other family protein
PARG10856	HB-other	sequence-specific DNA binding transcription factors;sequence-specific DNA binding
PARG07243	TALE	BEL1-like homeodomain 3
PARG07244	TALE	BEL1-like homeodomain 11
PARG07604	TALE	homeobox gene 1
PARG06309	HD-ZIP	HD-ZIP family protein
PARG08611	HD-ZIP	homeobox protein 16
PARG06265	HD-ZIP	homeobox 1
PARG07630	HD-ZIP	HD-ZIP family protein
PARG06803	HD-ZIP	homeobox-leucine zipper protein 17

PARG10842	HD-ZIP	homeobox protein 52
PARG07629	TALE	KNOTTED-like from Arabidopsis thaliana
PARG08287	TALE	KNOTTED1-like homeobox gene 3
PARG06089	TALE	KNOTTED1-like homeobox gene 6
PARG07899	HD-ZIP	homeobox gene 8
PARG06195	C2H2	C2H2 family protein
PARG06631	C2H2	C2H2-like zinc finger protein
PARG06744	C3H	C3H family protein
PARG06902	C2H2	zinc finger protein 4
PARG06920	C2H2	C2H2-like zinc finger protein
PARG06941	C2H2	indeterminate(ID)-domain 5
PARG06942	C2H2	indeterminate(ID)-domain 4
PARG06955	C3H	floral homeotic protein (HUA1)
PARG07165	C2H2	C2H2-like zinc finger protein
PARG07316	C2H2	indeterminate(ID)-domain 2
PARG07573	C2H2	indeterminate(ID)-domain 7
PARG07691	C2H2	salt tolerance zinc finger
PARG07766	C2H2	C2H2-like zinc finger protein
PARG08433	C2H2	C2H2-like zinc finger protein
PARG08580	C2H2	C2H2 family protein
PARG09154	C2H2	C2H2 family protein
PARG09239	C3H	C3H family protein
PARG09355	C2H2	C2H2 family protein
PARG09370	C3H	CCCH-type zinc finger protein with ARM repeat domain
PARG10120	C2H2	C2H2-like zinc finger protein
PARG11374	C3H	floral homeotic protein (HUA1)
PARG06125	MYB	myb domain protein 66
PARG06307	MYB	myb domain protein 88
PARG06360	MYB	myb domain protein 105
PARG06487	MYB	myb domain protein 16
PARG06699	MYB	myb domain protein 62
PARG06792	MYB	myb domain protein 4
PARG07017	MYB	MYB family protein
PARG07510	MYB	myb domain protein 23

PARG07514	MYB	myb domain protein 3
PARG07516	MYB	myb domain protein 3
PARG07517	MYB	myb domain protein 5
PARG07518	MYB	myb domain protein 7
PARG07627	MYB	MYB family protein
PARG07634	MYB	myb domain protein 84
PARG07749	MYB	myb domain protein r1
PARG07854	MYB	myb domain protein 73
PARG07937	MYB	Homeodomain-like protein
PARG08563	MYB	myb domain protein 93
PARG08838	MYB	myb domain protein 3
PARG09394	MYB	myb domain protein 83
PARG10363	MYB	myb domain protein 101
PARG10364	MYB	myb domain protein 97
PARG10468	MYB	myb domain protein 3
PARG10471	MYB	MYB family protein
PARG10618	MYB	MYB-like 102
PARG11257	MYB	myb domain protein 63
PARG11337	MYB	myb domain protein 67
PARG11401	MYB	myb domain protein 67
PARG06212	MYB_related	MYB_related family protein
PARG07284	MYB_related	RAD-like 6
PARG07601	MYB_related	MYB_related family protein
PARG08702	MYB_related	MYB_related family protein
PARG08703	MYB_related	MYB_related family protein
PARG09409	MYB_related	myb domain protein 3r-4
PARG09358	bHLH	
PARG06854	C2H2	
PARG17394	Dof	cycling DOF factor 3
PARG17422	Trihelix	Trihelix family protein
PARG17500	Trihelix	Trihelix family protein
PARG17511	NAC	NAC domain containing protein 52
PARG17512	NAC	NAC domain containing protein 2
PARG17563	bHLH	bHLH family protein

PARG17568 bHLH	bHLH family protein
PARG17807 Trihelix	Trihelix family protein
PARG17946 WRKY	WRKY family protein
PARG17956 SBP	squamosa promoter binding protein-like 8
PARG17960 GATA	GATA transcription factor 4
PARG17967 bHLH	bHLH family protein
PARG17984 GRAS	GRAS family protein
PARG18001 YABBY	YABBY family protein
PARG18099 LBD	LOB domain-containing protein 31
PARG18100 LBD	LOB domain-containing protein 18
PARG18152 TCP	TCP family protein
PARG18374 WRKY	WRKY family protein
PARG18379 WRKY	WRKY DNA-binding protein 56
PARG18436 bZIP	G-box binding factor 3
PARG18453 WRKY	WRKY family protein
PARG18491 bHLH	bHLH family protein
PARG18500 LBD	LOB domain-containing protein 1
PARG18509 Dof	Dof family protein
PARG18533 bHLH	bHLH family protein
PARG18546 NAC	NAC family protein
PARG18567 bHLH	bHLH family protein
PARG18585 NAC	NAC transcription factor-like 9
PARG18586 NAC	NAC domain containing protein 52
PARG18588 NAC	NAC transcription factor-like 9
PARG18590 NAC	NAC domain containing protein 50
PARG18591 NAC	NAC transcription factor-like 9
PARG18638 NAC	NAC transcription factor-like 9
PARG18641 NAC	NAC transcription factor-like 9
PARG18642 NAC	NAC 014
PARG18665 NAC	NAC 014
PARG18666 NAC	NAC transcription factor-like 9
PARG18667 NAC	NAC 014
PARG18668 NAC	NAC transcription factor-like 9
PARG18669 NAC	NAC domain containing protein 71

PARG18670	NAC	NAC 014
PARG18672	NAC	NAC 014
PARG18677	NAC	NAC domain containing protein 50
PARG18679	NAC	NTM1-like 8
PARG18683	NAC	NTM1-like 8
PARG18685	NAC	NAC domain containing protein 50
PARG18689	NAC	NTM1-like 8
PARG18691	NAC	NAC domain containing protein 50
PARG18730	GATA	GATA transcription factor 9
PARG18766	LBD	LOB domain-containing protein 1
PARG18787	NF-YB	nuclear factor Y, subunit B1
PARG18894	bHLH	bHLH family protein
PARG18905	WRKY	WRKY DNA-binding protein 43
PARG18925	Dof	OBF-binding protein 3
PARG18962	GRAS	GRAS family protein
PARG18980	LBD	LOB domain-containing protein 33
PARG19084	NF-X1	[illegible] transcription factors;zinc ion binding;sequence-specific DNA binding transcription factors
PARG19103	bHLH	bHLH family protein
PARG19109	Trihelix	sequence-specific DNA binding transcription factors
PARG19110	LBD	LOB domain-containing protein 21
PARG19131	LBD	LBD family protein
PARG19170	WRKY	WRKY DNA-binding protein 70
PARG19171	WRKY	WRKY DNA-binding protein 55
PARG19180	EIL	EIL family protein
PARG19220	LBD	LOB domain-containing protein 15
PARG19329	NAC	NAC 007
PARG19399	bHLH	bHLH family protein
PARG19400	bHLH	bHLH family protein
PARG19401	bHLH	bHLH family protein
PARG19402	Trihelix	Trihelix family protein
PARG19415	TCP	TCP family protein
PARG19445	HSF	heat shock transcription factor B2A

PARG19514	GRAS	SCARECROW-like 8
PARG19547	WRKY	WRKY DNA-binding protein 27
PARG19604	bHLH	bHLH family protein
PARG19605	WRKY	WRKY family protein
PARG19669	Dof	Dof family protein
PARG19720	NAC	TCV-interacting protein
PARG19765	E2F/DP	Transcription factor DP
PARG19859	WRKY	WRKY DNA-binding protein 69
PARG19860	GRAS	RGA-like 1
PARG19871	bZIP	basic region/leucine zipper motif 53
PARG19882	GATA	GATA transcription factor 16
PARG20084	bHLH	bHLH family protein
PARG20152	HSF	heat shock transcription factor A6B
PARG20172	bZIP	basic leucine zipper 25
PARG20174	bHLH	bHLH family protein
PARG20193	WRKY	WRKY DNA-binding protein 21
PARG20214	bHLH	bHLH family protein
PARG20222	LBD	lateral organ boundaries-domain 16
PARG20223	LBD	lateral organ boundaries-domain 29
PARG20224	LBD	lateral organ boundaries-domain 29
PARG20240	bHLH	conserved peptide upstream open reading frame 7
PARG20271	NAC	NAC domain containing protein 74
PARG20285	GRAS	GRAS family protein
PARG20409	bHLH	phytochrome interacting factor 3-like 5
PARG20480	Trihelix	Trihelix family protein
PARG20549	bHLH	bHLH family protein
PARG20599	FAR1	FAR1 family protein
PARG20600	FAR1	FAR1 family protein
PARG20601	FAR1	FAR1 family protein
PARG20602	FAR1	FAR1 family protein
PARG20636	bZIP	bZIP family protein
PARG20693	Nin-like	Nin-like family protein
PARG20780	NAC	NAC domain containing protein 36
PARG20854	bHLH	bHLH family protein

PARG20868 bZIP	bZIP family protein
PARG20910 bHLH	bHLH family protein
PARG20938 bHLH	bHLH family protein
PARG21068 bHLH	ROOT HAIR DEFECTIVE 6-LIKE 2
PARG21069 bHLH	ROOT HAIR DEFECTIVE 6-LIKE 2
PARG21080 LBD	LOB domain-containing protein 38
PARG21123 bHLH	bHLH family protein
PARG21213 bHLH	bHLH family protein
PARG21224 bZIP	abscisic acid responsive elements-binding factor 3
PARG21460 bZIP	G-box binding factor 1
PARG21505 NAC	NAC family protein
PARG21520 bHLH	cryptochrome-interacting basic-helix-loop-helix 1
PARG21525 NAC	NAC domain containing protein 47
PARG21556 bZIP	basic leucine-zipper 44
PARG21596 SRS	Lateral root primordium (LRP) protein-related
PARG21623 GATA	GATA transcription factor 5
PARG21659 BBR-BPC	basic pentacysteine 4
PARG21731 NAC	NAC family protein
PARG21935 NAC	vascular related NAC-domain protein 7
PARG21944 GRAS	SCARECROW-like 21
PARG21968 FAR1	FAR1-related sequence 7
PARG22029 NAC	NAC domain containing protein 17
PARG22058 FAR1	FAR1-related sequence 5
PARG22215 FAR1	FAR1-related sequence 7
PARG22279 FAR1	FAR1-related sequence 5
PARG22294 bZIP	VIRE2-interacting protein 1
PARG22326 Trihelix	sequence-specific DNA binding transcription factors
PARG22327 Trihelix	Trihelix family protein
PARG22369 bZIP	bZIP family protein
PARG22421 NAC	NAC domain containing protein 103
PARG22423 NAC	NAC domain containing protein 103
PARG22550 GRAS	SCARECROW-like 1
PARG22668 Whirly	WHIRLY 2
PARG22726 Nin-like	Nin-like family protein

PARG22797 NAC	NAC family protein
PARG22822 bZIP	basic region/leucine zipper motif 60
PARG22873 BES1	beta-amylase 2
PARG22899 Trihelix	Trihelix family protein
PARG23015 bZIP	basic region/leucine zipper transcription factor 16
PARG18494 ARF	auxin response factor 18
PARG18731 ARF	auxin response factor 16
PARG19990 ARF	auxin response factor 1
PARG17615 B3	B3 family protein
PARG17655 B3	B3 family protein
PARG18583 B3	B3 family protein
PARG20424 B3	B3 family protein
PARG21257 B3	B3 family protein
PARG21259 B3	B3 family protein
PARG21260 B3	B3 family protein
PARG21261 B3	B3 family protein
PARG21299 B3	B3 family protein
PARG21302 B3	B3 family protein
PARG21315 B3	B3 family protein
PARG21359 B3	related to ABI3/VP1 1
PARG21369 B3	B3 family protein
PARG21373 B3	B3 family protein
PARG18791 AP2	related to AP2.7
PARG17829 ERF	ERF family protein
PARG17903 ERF	ethylene-responsive element binding factor 13
PARG17904 ERF	ethylene-responsive element binding factor 13
PARG17905 ERF	ethylene-responsive element binding factor 13
PARG17906 ERF	ethylene-responsive element binding factor 13
PARG17907 ERF	ethylene-responsive element binding factor 13
PARG18439 ERF	cytokinin response factor 4
PARG18492 ERF	ERF family protein
PARG19106 ERF	ERF family protein
PARG19138 ERF	ERF family protein
PARG19277 ERF	ethylene responsive element binding factor 1

PARG19278	ERF	ERF family protein
PARG19279	ERF	ERF family protein
PARG19427	ERF	C-repeat-binding factor 4
PARG19428	ERF	ERF family protein
PARG19596	ERF	cytokinin response factor 4
PARG19851	ERF	ERF family protein
PARG20194	ERF	ERF domain protein 12
PARG20249	ERF	ERF family protein
PARG20250	ERF	ERF family protein
PARG20251	ERF	ethylene response factor 1
PARG20509	ERF	ERF family protein
PARG21235	ERF	redox responsive transcription factor 1
PARG22450	ERF	ERF family protein
PARG21599	DBB	DBB family protein
PARG21634	DBB	DBB family protein
PARG19975	CO-like	B-box type zinc finger protein with CCT domain
PARG17695	ARR-B	response regulator 2
PARG18169	ARR-B	response regulator 2
PARG19430	ARR-B	response regulator 1
PARG19878	ARR-B	response regulator 2
PARG18889	G2-like	G2-like family protein
PARG19114	G2-like	G2-like family protein
PARG20332	G2-like	phosphate starvation response 1
PARG21337	G2-like	G2-like family protein
PARG22967	G2-like	G2-like family protein
PARG18141	MIKC_MADS	AGAMOUS-like 6
PARG18142	MIKC_MADS	AGAMOUS-like 20
PARG19408	MIKC_MADS	AGAMOUS-like 6
PARG19411	MIKC_MADS	AGAMOUS-like 42
PARG21741	MIKC_MADS	AGAMOUS-like 16
PARG17700	M-type_MADS	AGAMOUS-like 80
PARG17714	M-type_MADS	M-type_MADS family protein
PARG17791	M-type_MADS	AGAMOUS-like 62
PARG20928	M-type_MADS	AGAMOUS-like 62

PARG20929	M-type_MADS	AGAMOUS-like 62
PARG20980	M-type_MADS	AGAMOUS-like 62
PARG20981	M-type_MADS	AGAMOUS-like 62
PARG22422	M-type_MADS	AGAMOUS-like 82
PARG19690	GRF	growth-regulating factor 7
PARG19797	GRF	growth-regulating factor 5
PARG22946	GRF	growth-regulating factor 5
PARG19959	HB-other	HB-other family protein
PARG19058	WOX	WUSCHEL related homeobox 5
PARG22755	WOX	WUSCHEL related homeobox 13
PARG21576	TALE	BEL1-like homeodomain 6
PARG19009	TALE	TALE family protein
PARG18527	HD-ZIP	homeobox 7
PARG18190	HD-ZIP	HD-ZIP family protein
PARG19441	HD-ZIP	HD-ZIP family protein
PARG21116	HB-PHD	Homeodomain-like protein with RING/FYVE/PHD-type zinc finger domain
PARG17970	C2H2	C2H2-like zinc finger protein
PARG18626	C2H2	C2H2 family protein
PARG18718	C2H2	C2H2 family protein
PARG18900	C2H2	C2H2 family protein
PARG19092	C3H	C3H family protein
PARG19309	C2H2	C2H2-like zinc finger protein
PARG19421	C3H	C3H family protein
PARG19492	C2H2	C2H2-like zinc finger protein
PARG19966	C3H	C3H family protein
PARG20153	C2H2	indeterminate(ID)-domain 12
PARG20218	C2H2	C2H2 family protein
PARG21077	C2H2	salt tolerance zinc finger
PARG21627	C3H	nucleic acid binding;zinc ion binding;DNA binding
PARG21661	C3H	C3H family protein
PARG21753	C3H	floral homeotic protein (HUA1)
PARG21823	C2H2	C2H2 family protein
PARG21866	C2H2	C2H2 family protein
PARG17841	MYB	MYB family protein

PARG18205	MYB	Homeodomain-like protein
PARG18243	MYB	myb domain protein 17
PARG18508	MYB	myb domain protein 7
PARG18754	MYB	MYB family protein
PARG19034	MYB	myb domain protein 109
PARG19048	MYB	MYB family protein
PARG19104	MYB	myb domain protein 73
PARG19291	MYB	MYB family protein
PARG19459	MYB	myb domain protein 19
PARG19783	MYB	myb domain protein 9
PARG19891	MYB	myb domain protein 12
PARG20254	MYB	myb domain protein 15
PARG20877	MYB	myb domain protein 4
PARG21139	MYB	myb domain protein 73
PARG22552	MYB	myb domain protein 52
PARG17742	MYB_related	MYB family protein
PARG18366	MYB_related	MYB_related family protein
PARG18576	MYB_related	MYB_related family protein
PARG18577	MYB_related	MYB_related family protein
PARG19273	MYB_related	MYB_related family protein
PARG19607	MYB_related	MYB_related family protein
PARG20592	MYB_related	DnaJ domain ;Myb-like DNA-binding domain
PARG21040	MYB_related	telomere repeat binding factor 1
PARG21514	MYB_related	RAD-like 1
PARG21519	MYB_related	RAD-like 6
PARG21554	MYB_related	MYB_related family protein
PARG18330	MYB_related	
PARG22805	MYB_related	
PARG28299	RAV	RAV family protein
PARG23073	HSF	heat shock transcription factor A1D
PARG23108	bHLH	bHLH family protein
PARG23125	bHLH	bHLH family protein
PARG23158	NAC	NAC domain containing protein 71
PARG23210	WRKY	WRKY DNA-binding protein 21

PARG23225	YABBY	YABBY family protein
PARG23229	NAC	NAC 007
PARG23370	GRAS	GRAS family protein
PARG23488	GATA	GATA transcription factor 26
PARG23559	SRS	
PARG23600	Trihelix	Trihelix family protein
PARG23653	FAR1	FAR1-related sequence 5
PARG23675	FAR1	FAR1 family protein
PARG23709	bHLH	bHLH family protein
PARG23715	bHLH	bHLH family protein
PARG23723	TCP	TCP family protein
PARG23754	GRAS	GRAS family protein
PARG23768	HSF	HSF family protein
PARG23796	NF-YC	nuclear factor Y, subunit C3
PARG23841	bHLH	bHLH family protein
PARG23892	WRKY	WRKY family protein
PARG23926	FAR1	FAR1-related sequence 10
PARG23962	bZIP	G-box binding factor 3
PARG23999	WRKY	WRKY family protein
PARG24080	Nin-like	NIN like protein 7
PARG24106	Dof	Dof family protein
PARG24120	WRKY	WRKY DNA-binding protein 33
PARG24147	bHLH	bHLH family protein
PARG24148	bHLH	bHLH family protein
PARG24149	bHLH	bHLH family protein
PARG24150	bHLH	bHLH family protein
PARG24151	bHLH	bHLH family protein
PARG24173	NAC	NAC domain containing protein 83
PARG24189	bHLH	bHLH family protein
PARG24202	bHLH	bHLH family protein
PARG24210	Trihelix	Trihelix family protein
PARG24221	LFY	floral meristem identity control protein LEAFY (LFY)
PARG24247	Trihelix	Trihelix family protein
PARG24268	NAC	NAC 014

PARG24293	NAC	NAC family protein
PARG24382	WRKY	WRKY DNA-binding protein 75
PARG24385	SBP	SBP family protein
PARG24400	NF-YC	nuclear factor Y, subunit C1
PARG24420	Trihelix	Trihelix family protein
PARG24448	GRAS	RGA-like 1
PARG24587	LBD	LBD family protein
PARG24649	Dof	Dof family protein
PARG24664	bHLH	bHLH family protein
PARG24702	E2F/DP	DP-E2F-like 1
PARG24729	bHLH	bHLH family protein
PARG24742	LBD	LOB domain-containing protein 27
PARG24770	WRKY	WRKY DNA-binding protein 72
PARG24781	bHLH	BANQUO 3
PARG24783	bHLH	BANQUO 3
PARG24788	bHLH	bHLH family protein
PARG24815	TCP	TEOSINTE BRANCHED, cycloidea and PCF (TCP) 14
PARG24838	ZF-HD	mini zinc finger 1
PARG24842	Nin-like	RWP-RK domain-containing protein
PARG24857	NAC	NAC domain containing protein 100
PARG24920	bHLH	bHLH family protein
PARG24945	NAC	NAC domain containing protein 32
PARG24970	TCP	TEOSINTE BRANCHED 1, cycloidea and PCF transcription factor 5
PARG24983	SBP	SBP family protein
PARG24998	Dof	OBF binding protein 4
PARG25002	bHLH	bHLH family protein
PARG25009	bZIP	basic leucine-zipper 70
PARG25026	FAR1	FAR1 family protein
PARG25045	NF-YB	nuclear factor Y, subunit B13
PARG25065	TCP	TCP family protein
PARG25105	NAC	NAC domain containing protein 58
PARG25111	bHLH	BR enhanced expression 3
PARG25226	NF-X1	NF-X-like 1
PARG25314	NAC	NAC domain containing protein 57

PARG25347	NAC	NAC domain containing protein 47
PARG25348	NAC	NAC domain containing protein 47
PARG25374	NAC	NAC-like, activated by AP3/PI
PARG25411	HSF	heat shock transcription factor B3
PARG25529	FAR1	FAR1-related sequence 12
PARG25679	LBD	LOB domain-containing protein 20
PARG25720	NAC	NAC domain containing protein 50
PARG25757	Dof	cycling DOF factor 2
PARG26144	bHLH	bHLH family protein
PARG26250	BES1	BES1 family protein
PARG26267	bHLH	bHLH family protein
PARG26268	bHLH	bHLH family protein
PARG26273	bHLH	bHLH family protein
PARG26279	bHLH	bHLH family protein
PARG26312	FAR1	FAR1-related sequence 5
PARG26332	FAR1	FAR1-related sequence 5
PARG26346	bHLH	bHLH family protein
PARG26356	bHLH	bHLH family protein
PARG26437	bZIP	bZIP family protein
PARG26450	SBP	squamosa promoter binding protein-like 9
PARG26477	FAR1	FAR1-related sequence 5
PARG26603	NAC	NAC domain containing protein 103
PARG26611	NF-YA	nuclear factor Y, subunit A10
PARG26652	E2F/DP	E2F transcription factor 3
PARG26667	TCP	TCP family protein
PARG26699	bZIP	bZIP family protein
PARG26717	bZIP	bZIP family protein
PARG26733	HSF	heat shock transcription factor A3
PARG26739	Trihelix	Trihelix family protein
PARG26787	Trihelix	sequence-specific DNA binding transcription factors
PARG26796	Trihelix	Trihelix family protein
PARG26906	bHLH	bHLH family protein
PARG26915	bHLH	bHLH family protein
PARG26926	HSF	heat shock factor 4

PARG26991	GATA	ZIM-LIKE 2
PARG26993	GATA	ZIM-like 1
PARG27042	Dof	OBF binding protein 1
PARG27082	LBD	ASYMMETRIC LEAVES 2-like 1
PARG27109	Dof	TARGET OF MONOPTEROS 6
PARG27127	LBD	LOB domain-containing protein 22
PARG27159	BES1	BES1 family protein
PARG27179	bZIP	G-box binding factor 1
PARG27200	GRAS	GRAS family protein
PARG27214	GATA	GATA family protein
PARG27216	ZF-HD	homeobox protein 24
PARG27263	bZIP	basic leucine-zipper 44
PARG27296	SRS	Lateral root primordium (LRP) protein-related
PARG27304	GATA	GATA transcription factor 5
PARG27321	NAC	vascular related NAC-domain protein 1
PARG27341	bHLH	bHLH family protein
PARG27379	bZIP	bZIP family protein
PARG27458	Nin-like	RWP-RK domain-containing protein
PARG27565	bHLH	LJRHL1-like 3
PARG27622	LBD	LOB domain-containing protein 22
PARG27630	bHLH	bHLH family protein
PARG27636	HSF	heat shock transcription factor A2
PARG27665	NF-YA	nuclear factor Y, subunit A3
PARG27742	HRT-like	effector of transcription2
PARG27771	GATA	cytokinin-responsive gata factor 1
PARG27810	NAC	NAC domain containing protein 75
PARG27895	bHLH	bHLH family protein
PARG27926	Trihelix	Trihelix family protein
PARG27943	HSF	heat shock transcription factor C1
PARG27965	CPP	Tesmin/TSO1-like CXC domain-containing protein
PARG27984	GRAS	GRAS family protein
PARG28076	YABBY	YABBY family protein
PARG28284	WRKY	WRKY DNA-binding protein 32
PARG28300	bHLH	LJRHL1-like 1

PARG28413	bHLH	bHLH family protein
PARG28426	bZIP	basic leucine-zipper 42
PARG28506	GeBP	DNA-binding storekeeper protein-related transcriptional regulator
PARG28507	GeBP	DNA-binding storekeeper protein-related transcriptional regulator
PARG28525	GeBP	DNA-binding storekeeper protein-related transcriptional regulator
PARG28696	CAMTA	Calmodulin-binding transcription activator protein with CG-1 and Ankyrin domains
PARG28742	NF-YA	nuclear factor Y, subunit A1
PARG28745	LBD	LOB domain-containing protein 23
PARG24069	ARF	auxin response factor 9
PARG24203	ARF	auxin response factor 2
PARG27594	ARF	auxin response factor 19
PARG24271	B3	B3 family protein
PARG24461	B3	B3 family protein
PARG25350	B3	B3 family protein
PARG26261	B3	B3 family protein
PARG26331	B3	B3 family protein
PARG26670	B3	B3 family protein
PARG26936	B3	B3 family protein
PARG27018	B3	B3 family protein
PARG27048	B3	related to vernalization1 1
PARG27049	B3	B3 family protein
PARG27050	B3	B3 family protein
PARG27876	B3	B3 family protein
PARG27877	B3	B3 family protein
PARG27878	B3	B3 family protein
PARG25402	AP2	AP2 family protein
PARG26364	AP2	AP2 family protein
PARG23414	ERF	ERF family protein
PARG23452	ERF	ethylene responsive element binding factor 5
PARG23453	ERF	ethylene responsive element binding factor 1
PARG23457	ERF	RAV family protein
PARG23729	ERF	C-repeat-binding factor 4
PARG23730	ERF	C-repeat/DRE binding factor 2
PARG23731	ERF	ERF family protein

PARG23732	ERF	C-repeat-binding factor 4
PARG23733	ERF	ERF family protein
PARG23734	ERF	ERF family protein
PARG23735	ERF	ERF family protein
PARG23736	ERF	ERF family protein
PARG23969	ERF	cytokinin response factor 2
PARG24008	ERF	ERF family protein
PARG24216	ERF	ERF family protein
PARG24217	ERF	ERF family protein
PARG24262	ERF	related to AP2 11
PARG25041	ERF	related to AP2 11
PARG25099	ERF	ERF family protein
PARG25582	ERF	related to AP2 11
PARG26389	ERF	ERF family protein
PARG26684	ERF	ERF family protein
PARG26740	ERF	ERF family protein
PARG26931	ERF	redox responsive transcription factor 1
PARG27864	ERF	ERF family protein
PARG28062	ERF	ERF family protein
PARG27314	DBB	DBB family protein
PARG28604	DBB	DBB family protein
PARG24308	CO-like	CONSTANS-like 5
PARG25102	CO-like	B-box type zinc finger protein with CCT domain
PARG23515	ARR-B	ARR-B family protein
PARG28314	ARR-B	response regulator 12
PARG23043	G2-like	G2-like family protein
PARG25241	G2-like	G2-like family protein
PARG25809	G2-like	G2-like family protein
PARG26063	G2-like	G2-like family protein
PARG26456	G2-like	G2-like family protein
PARG27084	G2-like	G2-like family protein
PARG27982	G2-like	G2-like family protein
PARG23708	MIKC_MADS	AGAMOUS-like 19
PARG24389	MIKC_MADS	AGAMOUS-like 19

PARG24984	MIKC_MADS	MIKC_MADS family protein
PARG24985	MIKC_MADS	AGAMOUS-like 8
PARG25423	MIKC_MADS	AGAMOUS-like 18
PARG26964	MIKC_MADS	MIKC_MADS family protein
PARG27300	MIKC_MADS	MIKC_MADS family protein
PARG24851	M-type_MADS	AGAMOUS-like 65
PARG25289	M-type_MADS	AGAMOUS-like 29
PARG26101	M-type_MADS	M-type_MADS family protein
PARG26123	M-type_MADS	M-type_MADS family protein
PARG26153	M-type_MADS	M-type_MADS family protein
PARG26594	M-type_MADS	AGAMOUS-like 62
PARG26607	M-type_MADS	AGAMOUS-like 80
PARG26608	M-type_MADS	M-type_MADS family protein
PARG26610	M-type_MADS	AGAMOUS-like 80
PARG24159	GRF	growth-regulating factor 8
PARG26690	GRF	growth-regulating factor 4
PARG27688	GRF	growth-regulating factor 5
PARG25196	WOX	WUSCHEL related homeobox 1
PARG27334	WOX	WOX family protein
PARG25896	WOX	WUSCHEL related homeobox 11
PARG26416	TALE	BEL1-like homeodomain 1
PARG27139	TALE	BEL1-like homeodomain 2
PARG23870	TALE	TALE family protein
PARG23424	HD-ZIP	homeobox protein 2
PARG24755	HD-ZIP	homeobox 1
PARG26488	HD-ZIP	homeobox from Arabidopsis thaliana
PARG26799	HD-ZIP	homeobox 51
PARG27166	HD-ZIP	homeobox protein 40
PARG24626	TALE	KNOTTED1-like homeobox gene 6
PARG28175	TALE	KNOTTED1-like homeobox gene 3
PARG23717	TALE	KNOTTED-like homeobox of Arabidopsis thaliana 7
PARG25318	HD-ZIP	homeodomain GLABROUS 11
PARG23053	HD-ZIP	homeobox-7
PARG23105	C3H	C3H family protein

PARG23140	C2H2	C2H2 family protein
PARG23153	LSD	lsd one like 1
PARG23263	C2H2	C2H2 family protein
PARG24467	C3H	C3H family protein
PARG24468	C3H	C3H family protein
PARG24469	C3H	C3H family protein
PARG24470	C3H	C3H family protein
PARG24471	C3H	C3H family protein
PARG24473	C2H2	relative of early flowering 6
PARG24929	C2H2	C2H2-like zinc finger protein
PARG24955	C2H2	DNAJ heat shock N-terminal domain-containing protein
PARG25246	C2H2	indeterminate(ID)-domain 14
PARG25343	C2H2	indeterminate(ID)-domain 14
PARG25359	C2H2	C2H2 family protein
PARG25715	C3H	floral homeotic protein (HUA1)
PARG26378	C2H2	C2H2 family protein
PARG26397	C3H	CCCH-type zinc finger protein with ARM repeat domain
PARG26408	C3H	C3H family protein
PARG26464	C3H	C3H family protein
PARG26507	C2H2	C2H2 family protein
PARG26620	C3H	C3H family protein
PARG26779	C2H2	histone deacetylase 2C
PARG26855	C2H2	C2H2 family protein
PARG27126	C2H2	transcription factor IIIA
PARG27180	C2H2	indeterminate(ID)-domain 2
PARG27634	C3H	C3H family protein
PARG27779	C3H	C3H family protein
PARG27803	C3H	C3H family protein
PARG27936	C2H2	transcription factor IIIA
PARG28010	C3H	C3H family protein
PARG28014	C3H	zinc finger WD40 repeat protein 1
PARG28761	C2H2	C2H2 family protein
PARG23088	MYB	myb domain protein 9
PARG23244	MYB	myb domain protein 85

PARG23413	MYB	myb domain protein 5
PARG23770	MYB	myb domain protein 3r-5
PARG23881	MYB	myb domain protein 103
PARG23882	MYB	myb domain protein 103
PARG24105	MYB	myb domain protein 5
PARG24574	MYB	myb domain protein 86
PARG24640	MYB	myb domain protein 98
PARG24655	MYB	myb domain protein 66
PARG24732	MYB	MYB family protein
PARG24835	MYB	myb domain protein 94
PARG24858	MYB	myb domain protein 16
PARG25070	MYB	MYB family protein
PARG25112	MYB	myb domain protein 116
PARG25173	MYB	myb domain protein 4r1
PARG25176	MYB	myb domain protein 4
PARG25177	MYB	myb domain protein 4
PARG25328	MYB	myb domain protein 52
PARG25564	MYB	myb domain protein 60
PARG25895	MYB	myb domain protein 105
PARG26860	MYB	myb domain protein 27
PARG26875	MYB	myb domain protein 36
PARG27680	MYB	MYB family protein
PARG27681	MYB	MYB family protein
PARG27682	MYB	MYB family protein
PARG28102	MYB	myb domain protein 82
PARG28632	MYB	myb domain protein 2
PARG23418	MYB_related	MYB_related family protein
PARG24718	MYB_related	MYB_related family protein
PARG24930	MYB_related	WRKY family protein
PARG25317	MYB_related	MYB_related family protein
PARG26461	MYB_related	DNA-binding bromodomain-containing protein
PARG27210	MYB_related	RAD-like 1
PARG27221	MYB_related	RAD-like 6
PARG27506	MYB_related	MYB family protein

PARG27645	MYB_related	MYB_related family protein
PARG27745	MYB_related	MYB_related family protein
PARG27802	MYB_related	myb domain protein 84
PARG25000	bHLH	
PARG25050	C2H2	
PARG27909	C2H2	
PARG23858	MYB_related	
PARG29164	WRKY	WRKY DNA-binding protein 70
PARG29165	WRKY	WRKY DNA-binding protein 70
PARG29166	WRKY	WRKY DNA-binding protein 70
PARG29228	bHLH	bHLH family protein
PARG29259	WRKY	WRKY DNA-binding protein 33
PARG29288	GRAS	GRAS family protein
PARG29294	bZIP	bZIP family protein
PARG29299	bHLH	APRATAXIN-like
PARG29552	Trihelix	Trihelix family protein
PARG29593	HSF	heat shock transcription factor A3
PARG29682	YABBY	YABBY family protein
PARG29705	bZIP	bZIP family protein
PARG29759	bHLH	bHLH family protein
PARG29760	bHLH	bHLH family protein
PARG29949	BES1	beta-amylase 7
PARG29951	bHLH	bHLH family protein
PARG30216	NAC	NAC domain containing protein 47
PARG30225	GeBP	DNA-binding storekeeper protein-related transcriptional regulator
PARG30226	GeBP	DNA-binding storekeeper protein-related transcriptional regulator
PARG30424	WRKY	WRKY DNA-binding protein 2
PARG29184	B3	HSI2-like 1
PARG29274	ERF	ERF family protein
PARG29402	ERF	ethylene-responsive element binding protein
PARG30073	ERF	related to AP2 11
PARG30407	ERF	ERF family protein
PARG29293	G2-like	G2-like family protein
PARG29549	G2-like	G2-like family protein

PARG30223	G2-like	G2-like family protein
PARG29730	MIKC_MADS	AGAMOUS-like 21
PARG29233	C3H	C3H family protein
PARG29452	MYB	myb domain protein 1
PARG29492	MYB	MYB family protein
PARG30010	MYB	myb domain protein 20
PARG13517	RAV	related to ABI3/VP1 2
PARG12655	NF-YB	nuclear factor Y, subunit B6
PARG12657	Trihelix	Trihelix family protein
PARG12659	NF-YB	nuclear factor Y, subunit B3
PARG12701	bHLH	bHLH family protein
PARG12719	NAC	NAC domain containing protein 83
PARG12726	GRAS	RGA-like 1
PARG12735	TCP	TCP family protein
PARG12740	WRKY	WRKY DNA-binding protein 75
PARG12808	Trihelix	Trihelix family protein
PARG12825	FAR1	FAR1 family protein
PARG12872	NAC	NAC domain containing protein 44
PARG12897	LBD	LBD family protein
PARG13054	LBD	LOB domain-containing protein 27
PARG13075	WRKY	WRKY DNA-binding protein 72
PARG13086	bHLH	bHLH family protein
PARG13093	NAC	NAC family protein
PARG13097	ZF-HD	mini zinc finger 2
PARG13098	ZF-HD	homeobox protein 30
PARG13113	Dof	cycling DOF factor 2
PARG13128	NAC	NAC domain containing protein 100
PARG13152	WRKY	WRKY DNA-binding protein 3
PARG13254	TCP	plastid transcription factor 1
PARG13328	bHLH	BES1-interacting Myc-like protein 2
PARG13349	bZIP	basic leucine-zipper 42
PARG13519	TCP	TCP family protein
PARG13584	FAR1	FAR1-related sequence 5
PARG13656	LBD	LOB domain-containing protein 41

PARG13678	GRAS	GRAS family protein
PARG13681	bHLH	bHLH family protein
PARG13687	GeBP	DNA-binding storekeeper protein-related transcriptional regulator
PARG12706	B3	B3 family protein
PARG13069	AP2	AP2 family protein
PARG13072	AP2	AP2 family protein
PARG13146	ERF	related to AP2 11
PARG13530	ERF	ERF family protein
PARG13359	DBB	CONSTANS-like 2
PARG13322	G2-like	G2-like family protein
PARG12743	MIKC_MADS	MIKC_MADS family protein
PARG13313	MIKC_MADS	MIKC_MADS family protein
PARG13314	MIKC_MADS	AGAMOUS-like 8
PARG12758	M-type_MADS	M-type_MADS family protein
PARG12964	HD-ZIP	homeobox 1
PARG13082	HD-ZIP	homeobox protein 20
PARG12749	C2H2	zinc ion binding;nucleic acid binding;zinc ion binding
PARG13212	C2H2	C2H2-like zinc finger protein
PARG12821	MYB	myb domain protein 40
PARG12922	MYB	myb domain protein 98
PARG12931	MYB	myb domain protein 21
PARG13040	MYB	MYB family protein
PARG13096	MYB	myb domain protein 30
PARG13130	MYB	myb domain protein 16
PARG13587	MYB	myb domain protein 15
PARG13588	MYB	myb domain protein 15
PARG13594	MYB	MYB-like 102
PARG15006	FAR1	FAR1-related sequence 5
PARG15038	bZIP	bZIP family protein
PARG15043	TCP	TCP family protein
PARG15059	HSF	HSF family protein
PARG15119	WRKY	WRKY DNA-binding protein 69
PARG15232	WRKY	WRKY DNA-binding protein 40
PARG15301	FAR1	FAR1-related sequence 5

PARG15436 CPP	Tesmin/TSO1-like CXC domain-containing protein
PARG15474 LBD	LOB domain-containing protein 9
PARG15496 Trihelix	Trihelix family protein
PARG15545 NAC	NAC domain containing protein 86
PARG15549 bHLH	bHLH family protein
PARG15643 GATA	GATA transcription factor 9
PARG15715 bHLH	bHLH family protein
PARG15729 Dof	DOF zinc finger protein 1
PARG15756 LBD	LOB domain-containing protein 22
PARG15775 GeBP	DNA-binding storekeeper protein-related transcriptional regulator
PARG15799 NF-YA	nuclear factor Y, subunit A3
PARG15834 GRAS	GRAS family protein
PARG15877 Trihelix	sequence-specific DNA binding transcription factors
PARG15954 LBD	LOB domain-containing protein 24
PARG15961 GATA	ZIM-like 1
PARG16088 NAC	NAC domain containing protein 28
PARG15394 B3	B3 family protein
PARG15170 AP2	ARIA-interacting double AP2 domain protein
PARG15816 AP2	AP2 family protein
PARG15266 ERF	ERF family protein
PARG15282 ERF	ERF family protein
PARG15386 ERF	ERF family protein
PARG15623 ERF	ERF family protein
PARG15911 ERF	related to AP2 12
PARG15962 ERF	cytokinin response factor 4
PARG16046 G2-like	G2-like family protein
PARG15136 M-type_MADS	AGAMOUS-like 103
PARG15606 M-type_MADS	AGAMOUS-like 61
PARG15886 M-type_MADS	AGAMOUS-like 62
PARG15913 M-type_MADS	AGAMOUS-like 62
PARG15915 M-type_MADS	M-type_MADS family protein
PARG15818 GRF	growth-regulating factor 5
PARG15943 HB-other	Homeodomain-like transcriptional regulator
PARG15536 HD-ZIP	HD-ZIP family protein

PARG15645	HD-ZIP	HD-ZIP family protein
PARG16079	HD-ZIP	homeodomain GLABROUS 8
PARG15261	C2H2	zinc-finger protein 1
PARG15426	C3H	C3H family protein
PARG15601	C2H2	zinc-finger protein 1
PARG15604	C2H2	C2H2 family protein
PARG15759	C2H2	indeterminate(ID)-domain 7
PARG15575	MYB	myb domain protein 33
PARG15773	MYB	myb domain protein 26
PARG15814	MYB	MYB family protein
PARG15815	MYB	myb domain protein 6
PARG15369	MYB_related	myb domain protein 106
PARG15371	MYB_related	myb domain protein 106
PARG15657	MYB_related	MYB_related family protein
PARG15785	MYB_related	MYB_related family protein
PARG15808	MYB_related	TRF-like 6
PARG16082	MYB_related	MYB_related family protein
PARG15868	C3H	
PARG16143	WRKY	WRKY DNA-binding protein 23
PARG16211	WRKY	WRKY family protein
PARG16236	bHLH	bHLH family protein
PARG16339	bHLH	bHLH family protein
PARG16399	LBD	LOB domain-containing protein 24
PARG16423	bZIP	bZIP family protein
PARG16498	FAR1	FAR1-related sequence 5
PARG16526	bZIP	bZIP family protein
PARG16565	GRAS	scarecrow-like 3
PARG16653	SRS	Lateral root primordium (LRP) protein-related
PARG16683	Trihelix	sequence-specific DNA binding transcription factors
PARG16780	NF-YC	nuclear factor Y, subunit C11
PARG16878	GeBP	DNA-binding storekeeper protein-related transcriptional regulator
PARG16952	bZIP	ABA-responsive element binding protein 3
PARG17022	FAR1	FAR1-related sequence 5
PARG17170	EIL	EIL family protein

PARG17173	EIL	EIL family protein
PARG16111	ARF	auxin response factor 8
PARG17190	AP2	AP2 family protein
PARG16483	MIKC_MADS	AGAMOUS-like 15
PARG16293	HD-ZIP	homeobox from Arabidopsis thaliana
PARG16379	C3H	C3H family protein
PARG16172	MYB	myb domain protein 2
PARG16388	MYB	myb domain protein 83
PARG17078	MYB	MYB family protein
PARG16162	MYB_related	MYB_related family protein
PARG16289	MYB_related	DNA-binding bromodomain-containing protein
PARG17211	MYB_related	MYB-like 102
PARG16734	C2H2	
PARG16737	C2H2	
PARG12077	Trihelix	sequence-specific DNA binding transcription factors
PARG12081	Dof	Dof family protein
PARG12093	bHLH	bHLH family protein
PARG12132	TCP	TEOSINTE BRANCHED 1, cycloidea and PCF transcription factor 3
PARG12150	LBD	LOB domain-containing protein 2
PARG12157	VOZ	vascular plant one zinc finger protein
PARG12168	NAC	NAC domain containing protein 73
PARG12191	Dof	Dof family protein
PARG12192	Dof	OBF binding protein 4
PARG12207	bHLH	bHLH family protein
PARG12229	HSF	heat shock transcription factor B2A
PARG12269	Dof	TARGET OF MONOPTEROS 6
PARG12294	NAC	NAC domain containing protein 25
PARG12381	bHLH	bHLH family protein
PARG12384	bHLH	bHLH family protein
PARG12400	bHLH	bHLH family protein
PARG12520	WRKY	WRKY family protein
PARG12609	GRAS	SCARECROW-like 13
PARG12163	ARF	ARF family protein
PARG12199	B3	B3 family protein

PARG12339	B3	HSI2-like 1
PARG12147	ERF	ethylene responsive element binding factor 2
PARG12184	ERF	erf domain protein 9
PARG12070	G2-like	G2-like family protein
PARG12511	G2-like	G2-like family protein
PARG12631	G2-like	G2-like family protein
PARG12175	HB-other	homeobox-1
PARG12151	WOX	homeobox-3
PARG12540	TALE	KNOX/ELK homeobox transcription factor
PARG12187	C3H	C3H family protein
PARG12225	LSD	lsd one like 2
PARG12349	MYB	MYB-like 102
PARG12359	MYB	myb domain protein 15
PARG12148	MYB_related	cell division cycle 5
PARG12322	MYB_related	cell division cycle 5
PARG12325	MYB_related	cell division cycle 5
PARG12461	MYB_related	DNA binding
PARG12493	MYB_related	MYB_related family protein
PARG14308	NF-YC	nuclear factor Y, subunit C1
PARG14390	bHLH	RHD SIX-LIKE 1
PARG14420	GRAS	RGA-like 2
PARG14422	GeBP	DNA-binding storekeeper protein-related transcriptional regulator
PARG14446	NF-YC	nuclear factor Y, subunit C1
PARG14656	SBP	squamosa promoter binding protein-like 4
PARG14700	FAR1	FAR1-related sequence 5
PARG14802	Trihelix	sequence-specific DNA binding transcription factors
PARG14917	VOZ	vascular plant one zinc finger protein
PARG14528	ERF	ERF family protein
PARG14605	DBB	DBB family protein
PARG14441	G2-like	G2-like family protein
PARG14820	G2-like	G2-like family protein
PARG14852	G2-like	GBF's pro-rich region-interacting factor 1
PARG14298	M-type_MADS	M-type_MADS family protein
PARG14451	M-type_MADS	M-type_MADS family protein

PARG14306	C3H	C3H family protein
PARG14447	C3H	C3H family protein
PARG14883	C2H2	C2H2 family protein
PARG14380	MYB	myb domain protein 6
PARG14432	MYB	myb domain protein 113
PARG14434	MYB	myb domain protein 113
PARG14436	MYB	myb domain protein 113
PARG14437	MYB	myb domain protein 113
PARG14438	MYB	myb domain protein 113
PARG14499	C2H2	C2H2 family protein

Table S11. Annotation of the genes from expanded families

Group	No. of Gene	Function
OG000001[illegible]	69	Conserved protein
OG000001[illegible]	16	Receptor-like protein EIX2
OG000001[illegible]	10	Uncharacterized mitochondrial protein AtMg00310
OG000001[illegible]	9	Pentatricopeptide repeat-containing protein At1g62670
OG000002[illegible]	75	Conserved protein
OG000002[illegible]	16	Receptor-like protein 3
OG000002[illegible]	9	Conserved protein
OG000002[illegible]	9	Putative pinene synthase
OG000003[illegible]	4	Premnaspirodiene oxygenase
OG000003[illegible]	5	Receptor-like protein EIX1
OG000003[illegible]	24	UDP-glycosyltransferase 85A8
OG000003[illegible]	9	E3 ubiquitin-protein ligase AIRP2
OG000004[illegible]	5	Conserved protein
OG000005[illegible]	4	Wall-associated receptor kinase-like 4
OG000005[illegible]	12	Conserved protein
OG000005[illegible]	33	Conserved protein
OG000005[illegible]	13	Wall-associated receptor kinase 3
OG000005[illegible]	2	Receptor-like protein 6
OG000005[illegible]	2	TMV resistance protein N
OG000006[illegible]	2	Disease resistance protein RPM1
OG000006[illegible]	2	Probable LRR receptor-like serine/threonine-protein kinase At3g47570
OG000006[illegible]	10	Conserved protein
OG000006[illegible]	5	Conserved protein
OG000006[illegible]	18	L-type lectin-domain containing receptor kinase IX.1
OG000007[illegible]	4	Senescence-specific cysteine protease SAG39
OG000007[illegible]	29	Conserved protein
OG000007[illegible]	54	Conserved protein
OG000007[illegible]	2	Probable disease resistance protein At5g66900

OG000007[illegible]	14	Conserved protein
OG000008[illegible]	11	Exopolygalacturonase (Fragment)
OG000009[illegible]	15	Conserved protein
OG000009[illegible]	3	Conserved protein
OG000010[illegible]	2	UDP-glucose flavonoid 3-O-glucosyltransferase 7
OG000011[illegible]	18	Putative AC transposase
OG000012[illegible]	12	Conserved protein
OG000013[illegible]	2	Cysteine-rich receptor-like protein kinase 41
OG000015[illegible]	2	Acidic endochitinase
OG000015[illegible]	9	Patatin-like protein 2
OG000016[illegible]	5	Stemmadenine O-acetyltransferase
OG000016[illegible]	10	Ankyrin repeat-containing protein ITN1
OG000017[illegible]	91	Conserved protein
OG000018[illegible]	73	Conserved protein
OG000018[illegible]	4	L10-interacting MYB domain-containing protein
OG000020[illegible]	4	Uncharacterized protein At2g29880
OG000020[illegible]	3	S-protein homolog 3
OG000022[illegible]	22	Conserved protein
OG000023[illegible]	8	Conserved protein
OG000024[illegible]	25	Conserved protein
OG000024[illegible]	4	Conserved protein
OG000024[illegible]	2	Glu S.griseus protease inhibitor
OG000025[illegible]	2	3,9-dihydroxypterocarpan 6A-monooxygenase
OG000025[illegible]	85	Protein TAR1
OG000026[illegible]	84	Conserved protein
OG000026[illegible]	4	Putative ripening-related protein 2
OG000027[illegible]	83	Putative uncharacterized protein ART3
OG000027[illegible]	2	Receptor-like protein EIX2
OG000028[illegible]	2	Conserved protein
OG000029[illegible]	79	Putative uncharacterized protein ART2

OG0000294	2	Conserved protein
OG0000298	5	Protein DMR6-LIKE OXYGENASE 2
OG0000300	33	Conserved protein
OG0000308	1	Auxin-responsive protein SAUR64
OG0000310	3	Late embryogenesis abundant protein 14
OG0000322	1	Conserved protein
OG0000325	1	Cytochrome P450 89A9
OG0000331	5	Conserved protein
OG0000333	63	Conserved protein
OG0000338	1	Probable terpene synthase 9
OG0000342	2	Basic form of pathogenesis-related protein 1
OG0000353	2	Retrovirus-related Pol polyprotein from transposon TNT 1-94
OG0000356	9	Epidermis-specific secreted glycoprotein EP1
OG0000359	5	Conserved protein
OG0000360	5	SMR domain-containing protein At5g58720
OG0000362	47	Conserved protein
OG0000364	2	UDP-glycosyltransferase 92A1
OG0000370	7	Putative F-box protein At4g22180
OG0000378	11	Conserved protein
OG0000381	10	Protein ACCELERATED CELL DEATH 6
OG0000400	68	Conserved protein
OG0000432	3	Conserved protein
OG0000437	65	Conserved protein
OG0000456	1	Methylesterase 10
OG0000461	4	Egg cell-secreted protein 1.1
OG0000473	1	Cytochrome P450 704C1
OG0000476	4	Conserved protein
OG0000477	1	Disease resistance protein TAO1
OG0000478	6	Conserved protein
OG0000492	15	Conserved protein

OG0000528	2	NAC domain-containing protein JA2
OG0000530	14	Conserved protein
OG0000535	2	Early nodulin-55-2
OG0000538	1	Acyl carrier protein 1, chloroplastic
OG0000543	34	Conserved protein
OG0000555	1	17.6 kDa class II heat shock protein
OG0000563	3	Isoleucine N-monooxygenase 2
OG0000564	3	Conserved protein
OG0000565	1	Xanthotoxin 5-hydroxylase CYP82C4
OG0000568	1	Zinc finger CCCH domain-containing protein 55
OG0000585	3	Retrovirus-related Pol polyprotein from transposon TNT 1-94
OG0000604	1	Importin subunit alpha-2
OG0000624	2	Ent-kaurenoic acid oxidase 1
OG0000633	2	Ubiquinol oxidase, mitochondrial
OG0000643	14	Zinc finger BED domain-containing protein RICESLEEPER 1
OG0000648	1	36.4 kDa proline-rich protein
OG0000659	1	Transcription factor DIVARICATA
OG0000668	10	Conserved protein
OG0000685	3	Probable pre-mRNA-splicing factor ATP-dependent RNA helicase DEAH5
OG0000689	2	Uncharacterized protein At2g34160
OG0000690	1	Probable sulfate transporter 3.5
OG0000691	1	Non-specific lipid-transfer protein-like protein At2g13820
OG0000718	3	Conserved protein
OG0000722	4	Conserved protein
OG0000749	2	Subtilisin-like protease SBT1.7
OG0000754	1	Peroxidase 53
OG0000756	2	Conserved protein
OG0000760	27	Conserved protein
OG0000761	3	Conserved protein
OG0000763	4	Conserved protein

OG0000765	9	WAT1-related protein At3g53210
OG0000777	1	Conserved protein
OG0000791	3	Conserved protein
OG0000794	48	Conserved protein
OG0000795	39	Conserved protein
OG0000800	1	Protein STRICTOSIDINE SYNTHASE-LIKE 5
OG0000806	1	Laccase-4
OG0000807	2	Pentatricopeptide repeat-containing protein DOT4
OG0000811	2	Chitinase 1
OG0000821	1	Conserved protein
OG0000825	2	Conserved protein
OG0000827	2	ABC transporter C family member 3
OG0000830	2	Cyclic nucleotide-gated ion channel 11
OG0000834	47	Conserved protein
OG0000836	1	Conserved protein
OG0000876	1	Conserved protein
OG0000885	46	Conserved protein
OG0000893	3	ABC transporter A family member 7
OG0000902	1	G-type lectin S-receptor-like serine/threonine-protein kinase RKS1
OG0000906	3	Serine/threonine-protein kinase BLUS1
OG0000928	2	3-ketoacyl-CoA synthase 19
OG0000932	30	Conserved protein
OG0000933	45	Conserved protein
OG0000954	2	Flotillin-like protein 2
OG0001003	1	60S ribosomal protein L6
OG0001008	3	Senescence-specific cysteine protease SAG39
OG0001013	1	Ubiquitin carboxyl-terminal hydrolase 12
OG0001020	1	Probable aquaporin NIP5-1
OG0001021	1	Conserved protein
OG0001031	1	4-coumarate--CoA ligase-like 9

OG0001037	1	EH domain-containing protein 2
OG0001044	2	Transcription elongation factor 1 homolog
OG0001051	3	Thioredoxin H-type
OG0001057	6	Conserved protein
OG0001061	1	Cytochrome P450 CYP736A12
OG0001070	2	Putative fructokinase-8
OG0001079	1	Protein RALF-like 4
OG0001083	2	Conserved protein
OG0001096	1	Gamma-glutamyl peptidase 5
OG0001102	1	Pectinesterase 2
OG0001116	4	(R)-mandelonitrile lyase 3
OG0001120	4	Pentatricopeptide repeat-containing protein At3g53700, chloroplastic
OG0001121	2	Retrovirus-related Pol polyprotein from transposon TNT 1-94
OG0001151	3	Conserved protein
OG0001173	1	EG45-like domain containing protein
OG0001181	4	S-protein homolog 3
OG0001186	13	Conserved protein
OG0001187	1	Probable pectinesterase/pectinesterase inhibitor 51
OG0001207	1	Glyceraldehyde-3-phosphate dehydrogenase GAPC2, cytosolic
OG0001220	1	Peroxidase 44
OG0001232	8	Transposon Ty3-I Gag-Pol polyprotein
OG0001234	2	Conserved protein
OG0001236	2	Putative fasciclin-like arabinogalactan protein 20
OG0001237	1	G-type lectin S-receptor-like serine/threonine-protein kinase B120
OG0001241	16	Conserved protein
OG0001263	1	Retrovirus-related Pol polyprotein from transposon RE2
OG0001267	1	LIM domain-containing protein WLIM1
OG0001271	8	Conserved protein
OG0001294	1	Werner Syndrome-like exonuclease
OG0001306	11	Conserved protein

OG000132!	7	Probable inactive leucine-rich repeat receptor-like protein kinase At3g03770
OG000132!	4	Conserved protein
OG000134!	1	Conserved protein
OG000138!	4	Primary amine oxidase
OG000139(	2	Early nodulin-like protein 1
OG000139:	37	Conserved protein
OG000139:	29	Conserved protein
OG000139!	1	Conserved protein
OG000140:	1	G2/mitotic-specific cyclin-1
OG000142!	2	Early nodulin-like protein 1
OG000143:	4	Protein HIGH CHLOROPHYLL FLUORESCENCE PHENOTYPE 173, chloroplastic
OG000143:	1	Ribonuclease 3-like protein 2
OG000144!	1	Protein-tyrosine-phosphatase MKP1
OG000144(	1	Conserved protein
OG000147:	1	Histidine kinase CKI1
OG000147:	1	Protein MICRORCHIDIA 7
OG000149!	1	Conserved protein
OG000150:	35	Conserved protein
OG000150:	25	Conserved protein
OG000151:	1	Transcription repressor OFP6
OG000158:	2	Flavonol synthase/flavanone 3-hydroxylase
OG000160:	1	Anthocyanidin 3-O-glucosyltransferase 5
OG000160!	1	Putative F-box protein At1g65770
OG000161(	35	Conserved protein
OG000161(	1	Conserved protein
OG000168·	1	Conserved protein
OG000168!	1	Conserved protein
OG000173:	34	Conserved protein
OG000173!	27	Conserved protein
OG000175(	1	RNA-binding protein 39

OG0001764	2	PHD finger protein MALE MEIOCYTE DEATH 1
OG0001791	1	TMV resistance protein N
OG0001792	1	Axial regulator YABBY 1
OG0001806	1	Dirigent protein 18
OG0001828	1	Transmembrane protein 87A
OG0001830	1	Conserved protein
OG0001832	1	Transcription factor bHLH122
OG0001835	4	Probable RNA helicase SDE3
OG0001852	2	Conserved protein
OG0001873	1	Probable flavin-containing monooxygenase 1
OG0001917	16	Conserved protein
OG0001919	3	Conserved protein
OG0001920	31	Conserved protein
OG0001921	31	Movement protein
OG0001932	1	Subtilisin-like protease SBT1.7
OG0001934	1	ATP-dependent Clp protease ATP-binding subunit CLPT1
OG0001950	1	Conserved protein
OG0001980	6	Kinesin-like protein KIN-10A
OG0001994	1	Conserved protein
OG0001998	1	Amino acid transporter AVT3C
OG0002002	2	26S proteasome non-ATPase regulatory subunit 11 homolog
OG0002022	2	Protein DJ-1 homolog D
OG0002024	1	Conserved protein
OG0002049	7	Protein disulfide isomerase pTAC5, chloroplastic
OG0002053	2	Conserved protein
OG0002066	6	Transposon Ty3-G Gag-Pol polyprotein
OG0002108	2	Protein CROWDED NUCLEI 1
OG0002118	1	LOB domain-containing protein 36
OG0002121	3	NDR1/HIN1-like protein 3
OG0002123	1	G-type lectin S-receptor-like serine/threonine-protein kinase At4g03230

OG000213	32	Conserved protein
OG000213	31	Conserved protein
OG000214	1	Cytochrome P450 84A1
OG000214	2	Putative B3 domain-containing protein At4g03170
OG000214	1	Ubiquitin domain-containing protein DSK2b
OG000220	1	Probable LRR receptor-like serine/threonine-protein kinase At3g47570
OG000222	1	G-type lectin S-receptor-like serine/threonine-protein kinase At2g19130
OG000226	3	Conserved protein
OG000227	2	Transcription elongation factor SPT6 homolog
OG000232	1	Conserved protein
OG000233	1	Conserved protein
OG000233	1	B3 domain-containing protein Os01g0234100
OG000233	3	Vinorine synthase
OG000233	1	Receptor-like serine/threonine-protein kinase SD1-8
OG000233	31	Conserved protein
OG000234	1	Sn1-specific diacylglycerol lipase alpha
OG000235	1	Beta-glucosidase 25
OG000241	1	Conserved protein
OG000242	21	Conserved protein
OG000245	1	Conserved protein
OG000246	1	26S proteasome non-ATPase regulatory subunit 14 homolog
OG000247	1	Stress response protein nst1
OG000250	7	Putative exosome complex exonuclease RRP42
OG000252	1	GDSL esterase/lipase At5g55050
OG000253	2	Eukaryotic translation initiation factor 3 subunit C
OG000253	1	E3 ubiquitin-protein ligase PUB24
OG000254	4	Conserved protein
OG000254	2	citrate synthase, glyoxysomal
OG000271	1	ATP synthase gamma chain, chloroplastic
OG000273	1	S-protein homolog 1

OG000274[illegible]	3	Beta-glucosidase 11
OG000274[illegible]	1	Major pollen allergen Ole e 10
OG000275[illegible]	28	Conserved protein
OG000275[illegible]	1	NAC domain-containing protein 82
OG000276[illegible]	3	Conserved protein
OG000279[illegible]	4	ABC transporter C family member 14
OG000287[illegible]	1	Receptor-like kinase TMK4
OG000290[illegible]	1	Putative nuclear RNA export factor SDE5
OG000292[illegible]	1	Conserved protein
OG000295[illegible]	3	Cyclic nucleotide-gated ion channel 1
OG000295[illegible]	2	Cytochrome P450 81E8
OG000297[illegible]	28	Conserved protein
OG000297[illegible]	18	Conserved protein
OG000297[illegible]	1	Conserved protein
OG000299[illegible]	1	U-box domain-containing protein 75
OG000300[illegible]	1	Conserved protein
OG000302[illegible]	4	Pentatricopeptide repeat-containing protein At4g32430
OG000303[illegible]	2	Precursor of CEP8
OG000303[illegible]	1	Kelch repeat-containing protein At3g27220
OG000304[illegible]	1	Conserved protein
OG000309[illegible]	2	U4/U6.U5 tri-snRNP-associated protein 2
OG000309[illegible]	1	E2F transcription factor-like E2FE
OG000311[illegible]	1	Transcription factor bHLH91
OG000315[illegible]	1	Transcriptional activator DEMETER
OG000316[illegible]	4	Putative F-box protein At4g10190
OG000316[illegible]	8	Putative AC transposase
OG000317[illegible]	26	Conserved protein
OG000317[illegible]	27	Conserved protein
OG000317[illegible]	1	Stromal 70 kDa heat shock-related protein
OG000318[illegible]	2	60S ribosomal protein L18a

OG0003197	1	Conserved protein
OG0003215	1	Conserved protein
OG0003219	1	ABC transporter C family member 7
OG0003231	10	Pentatricopeptide repeat-containing protein At4g26680
OG0003250	7	Conserved protein
OG0003255	1	T-complex protein 1 subunit zeta 1
OG0003262	1	Conserved protein
OG0003268	1	L-lactate dehydrogenase A
OG0003280	1	Enhancer of mRNA-decapping protein 4
OG0003288	7	Conserved protein
OG0003319	1	Conserved protein
OG0003333	11	Conserved protein
OG0003336	23	Conserved protein
OG0003337	3	Conserved protein
OG0003349	4	Sulfate transporter 3.1
OG0003353	1	Conserved protein
OG0003363	2	ATP synthase subunit alpha, mitochondrial
OG0003395	2	Adenylosuccinate lyase
OG0003408	1	General transcription factor 3C polypeptide 3
OG0003448	1	Conserved protein
OG0003470	1	Agamous-like MADS-box protein AGL82
OG0003500	3	Conserved protein
OG0003505	11	Conserved protein
OG0003507	24	Conserved protein
OG0003508	24	DNA repair protein XRCC3 homolog
OG0003509	1	Conserved protein
OG0003513	1	Conserved protein
OG0003522	1	Serine/threonine receptor-like kinase NFP
OG0003541	4	Conserved protein
OG0003567	1	Conserved protein

OG0003591	1	Conserved protein
OG0003595	1	ABC transporter G family member 9
OG0003608	1	Sm-like protein LSM8
OG0003626	1	Protein PHYTOCHROME KINASE SUBSTRATE 1
OG0003632	1	Conserved protein
OG0003637	1	Biogenesis of lysosome-related organelles complex 1 subunit 1
OG0003638	1	DNA-directed RNA polymerases II and IV subunit 5A
OG0003639	3	Putative L,D-transpeptidase YkuD
OG0003646	2	Conserved protein
OG0003655	1	Protein OSB3, chloroplastic/mitochondrial
OG0003675	1	Putative F-box/FBD/LRR-repeat protein At4g03220
OG0003676	1	GATA transcription factor 20
OG0003678	5	Probable calcium-binding protein CML23
OG0003685	3	Retrovirus-related Pol polyprotein from transposon RE1
OG0003686	7	Conserved protein
OG0003687	3	Conserved protein
OG0003690	24	Conserved protein
OG0003691	21	Alpha-1,4 glucan phosphorylase L isozyme
OG0003740	1	Conserved protein
OG0003754	1	Ureide permease 2
OG0003764	1	Origin of replication complex subunit 1B
OG0003775	1	Conserved protein
OG0003786	1	Conserved protein
OG0003872	1	Conserved protein
OG0003874	1	Conserved protein
OG0003878	6	Probable protein phosphatase 2C 55
OG0003886	2	Conserved protein
OG0003893	3	Conserved protein
OG0003907	23	Conserved protein
OG0003908	22	Conserved protein

OG0003921	1	Conserved protein
OG0003947	1	Pentatricopeptide repeat-containing protein At5g39710
OG0003972	1	Pentatricopeptide repeat-containing protein At2g44880
OG0003979	1	LRR receptor-like serine/threonine-protein kinase HSL2
OG0003986	1	Protein ILITYHIA
OG0004024	1	Cysteine-rich receptor-like protein kinase 2
OG0004037	1	Conserved protein
OG0004129	2	Conserved protein
OG0004136	1	Conserved protein
OG0004138	1	Kiwellin
OG0004147	7	Conserved protein
OG0004152	22	Conserved protein
OG0004161	1	Putative disease resistance protein At1g50180
OG0004165	2	Auxin transport protein BIG
OG0004173	1	Exosome complex component RRP41 homolog
OG0004190	1	60S ribosomal protein L2, mitochondrial
OG0004196	1	Vacuolar protein sorting-associated protein 27
OG0004236	1	Fatty acid desaturase 4, chloroplastic
OG0004239	1	Allantoate deiminase 2
OG0004246	1	Conserved protein
OG0004252	1	Probable acetyltransferase NATA1-like
OG0004257	1	Non-classical arabinogalactan protein 30
OG0004269	1	Zinc finger CCCH domain-containing protein 1
OG0004282	1	Integrin-linked protein kinase 1
OG0004300	2	Sister chromatid cohesion protein SCC2
OG0004337	1	Protein terminal ear1 homolog
OG0004351	1	Pre-mRNA-splicing factor 38
OG0004352	1	Aladin
OG0004354	2	Conserved protein
OG0004358	1	Ethylene-responsive transcription factor ERF024

OG0004378	2	Conserved protein
OG0004426	1	Probable calcium-binding protein CML49
OG0004433	2	Conserved protein
OG0004438	2	Beta-amyrin 28-monooxygenase
OG0004440	17	Conserved protein
OG0004441	1	Cytochrome P450 705A22
OG0004448	18	Transcription initiation factor TFIID subunit 1
OG0004451	21	Conserved protein
OG0004452	21	Conserved protein
OG0004453	21	Conserved protein
OG0004462	1	Conserved protein
OG0004467	1	RNA polymerase sigma factor sigB
OG0004518	1	Pentatricopeptide repeat-containing protein At5g47360
OG0004534	1	BTB/POZ domain-containing protein At3g56230
OG0004583	1	Uncharacterized protein C6C3.02c
OG0004584	5	Conserved protein
OG0004587	1	Conserved protein
OG0004598	1	Alpha-1,3-mannosyl-glycoprotein 2-beta-N-acetylglucosaminyltransferase
OG0004620	3	Conserved protein
OG0004653	2	NADH dehydrogenase [ubiquinone] 1 alpha subcomplex subunit 12
OG0004706	1	COP9 signalosome complex subunit 8
OG0004728	1	CLAVATA3/ESR (CLE)-related protein 13
OG0004737	2	Conserved protein
OG0004830	1	Conserved protein
OG0004833	1	Stigma-specific STIG1-like protein 3
OG0004834	4	E3 ubiquitin-protein ligase RMA1
OG0004862	12	Conserved protein
OG0004863	18	Conserved protein
OG0004864	20	Conserved protein
OG0004897	1	Conserved protein

OG0004904	1	Golgi to ER traffic protein 4 homolog
OG0004914	3	Sulfite exporter TauE/SafE family protein 3
OG0004936	1	Conserved protein
OG0004973	1	Translocator protein homolog
OG0004979	1	Conserved protein
OG0004981	1	Conserved protein
OG0005011	1	Methyl-CpG-binding domain protein 4-like protein
OG0005033	1	Probable pectinesterase/pectinesterase inhibitor 46
OG0005065	1	O-fucosyltransferase 8
OG0005070	1	Conserved protein
OG0005129	1	Mitochondrial outer membrane import complex protein METAXIN
OG0005133	1	Conserved protein
OG0005158	2	Conserved protein
OG0005213	3	KDEL-tailed cysteine endopeptidase CEP1
OG0005228	1	Conserved protein
OG0005274	1	Chalcone--flavonone isomerase
OG0005282	1	Berberine bridge enzyme-like 7
OG0005319	1	GPI transamidase component PIG-T
OG0005341	1	Urea-proton symporter DUR3
OG0005372	1	Mannan endo-1,4-beta-mannosidase 2
OG0005380	1	Conserved protein
OG0005381	1	Conserved protein
OG0005418	2	Ribosomal protein S14, mitochondrial
OG0005458	1	Conserved protein
OG0005466	1	Conserved protein
OG0005470	1	Conserved protein
OG0005477	1	F-box protein SKIP23
OG0005484	3	Conserved protein
OG0005487	1	(+)-pulegone reductase
OG0005490	1	RING-H2 finger protein ATL52

OG0005491	1	F-box protein At1g49360
OG0005493	1	Conserved protein
OG0005494	10	Glucose-6-phosphate isomerase, cytosolic 1
OG0005496	2	Putative disease resistance protein RGA4
OG0005507	16	Alpha-glucan phosphorylase 2, cytosolic
OG0005628	3	Pentatricopeptide repeat-containing protein DOT4
OG0005690	1	Geranyl diphosphate phosphohydrolase
OG0005707	1	Pre-mRNA-processing factor 17
OG0005735	1	Argininosuccinate lyase, chloroplastic
OG0005743	1	Photosystem II core complex proteins psbY
OG0005890	1	Erlin-2-B
OG0005941	1	Ubiquinone biosynthesis O-methyltransferase
OG0005995	1	Pentatricopeptide repeat-containing protein At4g17616
OG0006010	1	Jacalin-related lectin 3
OG0006120	1	Pentatricopeptide repeat-containing protein At5g46100
OG0006135	1	Protein ENDOSPERM DEFECTIVE 1
OG0006151	1	Conserved protein
OG0006247	1	UPF0047 protein YjbQ
OG0006279	1	Protein TIC 55, chloroplastic
OG0006304	2	EPIDERMAL PATTERNING FACTOR-like protein 8
OG0006306	1	Conserved protein
OG0006325	2	ETHYLENE INSENSITIVE 3-like 5 protein
OG0006330	1	Probable ubiquitin-conjugating enzyme E2 25
OG0006336	4	Conserved protein
OG0006337	1	Conserved protein
OG0006349	2	Rhamnogalacturonan I rhamnosyltransferase 1
OG0006354	18	Conserved protein
OG0006355	16	Conserved protein
OG0006356	18	Conserved protein
OG0006357	17	Conserved protein

OG0006358	2	Conserved protein
OG0006461	1	Conserved protein
OG0006551	1	Conserved protein
OG0006632	1	Conserved protein
OG0006674	1	50S ribosomal protein 6, chloroplastic
OG0006707	1	Conserved protein
OG0006753	1	Diphthine methyltransferase homolog
OG0006771	1	Conserved protein
OG0006796	1	Probable transcription factor PosF21
OG0006835	1	Nitrate regulatory gene2 protein
OG0006863	1	Probable methyltransferase PMT5
OG0006881	1	Uncharacterized protein At5g08430
OG0006999	1	Conserved protein
OG0007187	1	Uncharacterized CRM domain-containing protein At3g25440
OG0007349	2	tRNA (guanine(37)-N1)-methyltransferase 2
OG0007418	1	Pentatricopeptide repeat-containing protein At1g09220
OG0007427	1	Conserved protein
OG0007429	1	Conserved protein
OG0007430	2	Inactive FRIGIDA-like protein 2
OG0007437	2	Conserved protein
OG0007474	1	F-box protein At5g07610
OG0007492	2	Conserved protein
OG0007494	16	Conserved protein
OG0007497	2	Cytosolic invertase 1
OG0007504	7	Putative AC9 transposase
OG0007641	1	Conserved protein
OG0007773	2	CLAVATA3/ESR (CLE)-related protein 1
OG0007787	1	Pentatricopeptide repeat-containing protein At1g66345
OG0007858	1	Conserved protein
OG0008153	1	Transcription factor TCP7

OG0008531	1	Conserved protein
OG0008537	4	RHOMBOID-like protein 10, chloroplastic
OG0008749	1	Zinc finger CCCH domain-containing protein 14
OG0008801	1	Polygalacturonase QRT3
OG0008841	1	E3 ubiquitin-protein ligase RNF13
OG0008862	1	Probable aspartic protease At2g35615
OG0008863	1	Cytochrome P450 71AV8
OG0008868	1	Conserved protein
OG0008871	1	1-aminocyclopropane-1-carboxylate oxidase homolog 1
OG0008879	6	Conserved protein
OG0008880	16	Conserved protein
OG0008881	8	Conserved protein
OG0008882	16	Conserved protein
OG0008884	1	Cytochrome P450 71D10
OG0008970	1	Pentatricopeptide repeat-containing protein At4g19191
OG0009025	1	Conserved protein
OG0009117	1	Conserved protein
OG0009121	1	Probable leucine-rich repeat receptor-like protein kinase At1g68400
OG0009193	1	Probable amidase At4g34880
OG0009333	1	Protein CROWDED NUCLEI 4
OG0009776	1	APO protein 3, mitochondrial
OG0009970	1	RNA pseudouridine synthase 7
OG0010099	1	Chaperone protein DnaJ
OG0010183	1	Conserved protein
OG0010184	1	Probable receptor-like protein kinase At5g24010
OG0010190	1	Putative defensin-like protein 20
OG0010197	1	Conserved protein
OG0010199	2	Defensin-like protein 183
OG0010230	1	F-box protein CPR1
OG0010234	2	Conserved protein

OG0010235	1	DUF724 domain-containing protein 3
OG0010238	1	Conserved protein
OG0010242	1	Conserved protein
OG0010247	1	ABC transporter G family member 15
OG0010250	2	Agamous-like MADS-box protein AGL62
OG0010259	1	Conserved protein
OG0010273	6	Retrovirus-related Pol polyprotein from transposon TNT 1-94
OG0010275	9	Conserved protein
OG0010286	15	Conserved protein
OG0010287	11	Conserved protein
OG0010288	13	Conserved protein
OG0010289	6	Conserved protein
OG0010290	14	Conserved protein
OG0010291	14	Conserved protein
OG0010292	1	tRNAse Z TRZ4
OG0010293	1	Conserved protein
OG0010294	2	Conserved protein
OG0010295	9	Pentatricopeptide repeat-containing protein At4g32430
OG0010360	1	RING-H2 finger protein ATL65
OG0010399	1	Conserved protein
OG0010588	1	Conserved protein
OG0011109	1	Conserved protein
OG0011132	1	70 kDa peptidyl-prolyl isomerase
OG0011148	1	Homeobox-leucine zipper protein HAT9
OG0011294	1	Pentatricopeptide repeat-containing protein At5g64320
OG0011346	1	NDR1/HIN1-like protein 10
OG0011372	2	Conserved protein
OG0011408	1	Ribosomal protein S10
OG0011430	1	Protein DOG1-like 4
OG0011483	1	Conserved protein

OG0011490	1	Ethylene-responsive transcription factor ERF114
OG0011508	2	Conserved protein
OG0011517	3	Putative F-box protein At1g33530
OG0011522	6	Conserved protein
OG0011526	14	Conserved protein
OG0011527	12	Conserved protein
OG0011530	8	Conserved protein
OG0011535	2	Conserved protein
OG0011870	3	Cannabidiolic acid synthase-like 2
OG0011883	2	Putative AC transposase
OG0011980	1	AT-hook motif nuclear-localized protein 16
OG0012249	1	Conserved protein
OG0012347	1	CASP-like protein 4D1
OG0012400	1	Conserved protein
OG0012486	1	Root meristem growth factor 6
OG0012507	1	Conserved protein
OG0012527	3	Conserved protein
OG0012532	1	Probable acyl-activating enzyme 1
OG0012541	1	Conserved protein
OG0012548	1	Non-functional pseudokinase ZED1
OG0012600	10	Sulfate transporter 3.1
OG0012601	1	Transcription factor MYB3
OG0012620	13	Conserved protein
OG0012621	13	Protein TOPLESS
OG0012622	13	Conserved protein
OG0012623	13	Conserved protein
OG0012625	8	Conserved protein
OG0012922	2	Conserved protein
OG0012976	1	Conserved protein
OG0013028	1	Taxane 13-alpha-hydroxylase

OG001305[illegible]	1	Conserved protein
OG001306[illegible]	1	Conserved protein
OG001310[illegible]	1	Conserved protein
OG001318[illegible]	1	Zinc finger protein AZF2
OG001321[illegible]	1	E3 ubiquitin-protein ligase SIRP1
OG001321[illegible]	2	Probable leucine-rich repeat receptor-like protein kinase At1g35710
OG001322[illegible]	1	FBD-associated F-box protein At1g66310
OG001322[illegible]	1	Pentatricopeptide repeat-containing protein At2g35030
OG001323[illegible]	1	Conserved protein
OG001324[illegible]	1	MDIS1-interacting receptor like kinase 2
OG001324[illegible]	1	Conserved protein
OG001327[illegible]	1	Pentatricopeptide repeat-containing protein At1g15510
OG001328[illegible]	1	Wall-associated receptor kinase-like 22
OG001331[illegible]	11	Conserved protein
OG001331[illegible]	1	Conserved protein
OG001331[illegible]	1	Conserved protein
OG001331[illegible]	3	Conserved protein
OG001331[illegible]	3	Conserved protein
OG001331[illegible]	9	Conserved protein
OG001332[illegible]	1	Protein FAR1-RELATED SEQUENCE 5
OG001332[illegible]	2	DNA topoisomerase 6 subunit A
OG001344[illegible]	1	Alcohol dehydrogenase-like 6
OG001357[illegible]	1	Precursor of CEP3
OG001364[illegible]	1	Pectinesterase/pectinesterase inhibitor
OG001365[illegible]	1	1-aminocyclopropane-1-carboxylate oxidase homolog 1
OG001365[illegible]	1	Conserved protein
OG001366[illegible]	2	Aspartic proteinase CDR1
OG001372[illegible]	1	Basic blue protein
OG001372[illegible]	1	Serpin-ZX
OG001372[illegible]	3	Conserved protein

OG0013738	2	Conserved protein
OG0013745	3	Uncharacterized mitochondrial protein AtMg00310
OG0013747	4	Retrovirus-related Pol polyprotein from transposon TNT 1-94
OG0013751	5	E3 ubiquitin-protein ligase RING1-like
OG0013769	9	Conserved protein
OG0013770	11	Conserved protein
OG0013771	10	Conserved protein
OG0013772	10	Conserved protein
OG0013773	1	Conserved protein
OG0013775	5	Probable cinnamyl alcohol dehydrogenase 1
OG0013776	11	Conserved protein
OG0013777	4	Conserved protein
OG0013779	3	Wall-associated receptor kinase 2
OG0013780	11	Pentatricopeptide repeat-containing protein At4g32430
OG0013781	1	Conserved protein
OG0013782	3	Conserved protein
OG0013783	1	Protein trichome birefringence-like 6
OG0013785	1	Conserved protein
OG0013819	3	Monooxygenase 2
OG0013828	1	NADH dehydrogenase [ubiquinone] iron-sulfur protein 2
OG0013847	3	Putative fasciclin-like arabinogalactan protein 20
OG0013900	1	Dimethylnonatriene synthase
OG0013925	2	Serpin-ZX
OG0013982	1	VQ motif-containing protein 31
OG0014011	1	Peroxisome biogenesis factor 10
OG0014036	1	GBF-interacting protein 1
OG0014038	1	Conserved protein
OG0014055	1	Conserved protein
OG0014071	1	Putative invertase inhibitor
OG0014094	2	Conserved protein

OG0014097	2	Conserved protein
OG0014125	9	Probable sodium/metabolite cotransporter BASS2
OG0014126	10	Conserved protein
OG0014127	8	Conserved protein
OG0014128	10	Conserved protein
OG0014129	10	Conserved protein
OG0014130	10	Conserved protein
OG0014131	8	Conserved protein
OG0014132	9	Protein TPR3
OG0014133	9	Conserved protein
OG0014135	10	Probable LRR receptor-like serine/threonine-protein kinase At1g56130
OG0014136	9	Sulfate transporter 3.1
OG0014171	1	Cytochrome c biogenesis CcmF C-terminal-like mitochondrial protein
OG0014287	3	Conserved protein
OG0014295	2	Putative calcium-transporting ATPase 13, plasma membrane-type
OG0014389	1	Alcohol-forming fatty acyl-CoA reductase
OG0014391	2	Conserved protein
OG0014411	2	Conserved protein
OG0014418	1	DNA-directed RNA polymerase I subunit 2
OG0014459	2	Conserved protein
OG0014460	2	Conserved protein
OG0014461	9	Conserved protein
OG0014462	8	Conserved protein
OG0014463	9	Conserved protein
OG0014465	9	Conserved protein
OG0014467	3	Elongation factor 2
OG0014470	9	Probable cinnamyl alcohol dehydrogenase 1
OG0014471	1	Probable aquaporin TIP-type RB7-5A
OG0014472	9	Conserved protein
OG0014474	9	Protein STIG1

OG0014476	1	Cyclic nucleotide-gated ion channel 1
OG0014478	8	Conserved protein
OG0014480	1	Conserved protein
OG0014501	3	Retrovirus-related Pol polyprotein from transposon 17.6
OG0014514	1	L-type lectin-domain containing receptor kinase IV.2
OG0014534	1	Conserved protein
OG0014542	1	ATP-dependent RNA helicase DEAH13
OG0014568	2	Geranylgeranyl pyrophosphate synthase, chloroplastic
OG0014571	1	Conserved protein
OG0014618	1	Aminopeptidase M1
OG0014654	1	Palmitoyl-acyl carrier protein thioesterase
OG0014702	1	Conserved protein
OG0014705	5	Conserved protein
OG0014706	1	MDIS1-interacting receptor like kinase 2
OG0014711	1	Flavanone 3-dioxygenase 3
OG0014716	1	Pathogenesis-related protein 1C
OG0014720	1	Protein DMP5
OG0014782	3	Sulfate transporter 3.1
OG0014794	5	Conserved protein
OG0014795	1	Conserved protein
OG0014796	1	Conserved protein
OG0014798	8	Conserved protein
OG0014799	7	Conserved protein
OG0014800	7	Conserved protein
OG0014801	8	Conserved protein
OG0014802	8	Conserved protein
OG0014803	8	Conserved protein
OG0014804	8	Chaperone protein dnaJ 10
OG0014805	7	Conserved protein
OG0014806	6	Putative transposon Ty5-1 protein YCL075W

OG0014807	8	Conserved protein
OG0014808	4	Monothiol glutaredoxin-S17
OG0014809	1	Disease resistance protein Piks-2
OG0014810	8	Conserved protein
OG0014811	3	Conserved protein
OG0014815	5	Conserved protein
OG0014818	2	Conserved protein
OG0014819	7	Conserved protein
OG0014820	2	Conserved protein
OG0014821	6	Protein tas
OG0014822	5	Conserved protein
OG0014823	5	Conserved protein
OG0014824	1	Conserved protein
OG0014827	1	Conserved protein
OG0014828	1	Uncharacterized protein L728
OG0014901	2	Conserved protein
OG0014919	1	Cytochrome P450 82A4
OG0014939	2	Conserved protein
OG0014948	1	Conserved protein
OG0014980	1	Exopolygalacturonase (Fragment)
OG0015011	1	Receptor-like protein EIX2
OG0015013	1	Conserved protein
OG0015026	3	BTB/POZ domain-containing protein DOT3
OG0015028	1	Conserved protein
OG0015029	1	Cysteine-rich receptor-like protein kinase 27
OG0015041	1	Lupeol synthase
OG0015062	1	Conserved protein
OG0015067	5	3-hydroxyisobutyryl-CoA hydrolase-like protein 3
OG0015082	2	G-type lectin S-receptor-like serine/threonine-protein kinase CES101
OG0015090	1	Alpha-amylase

OG0015093	1	G-type lectin S-receptor-like serine/threonine-protein kinase At4g03230
OG001510[illegible]	2	Retrovirus-related Pol polyprotein from transposon RE1
OG001514[illegible]	2	Leucine--tRNA ligase, cytoplasmic
OG001516[illegible]	1	Zeta-carotene desaturase, chloroplastic/chromoplastic
OG001516[illegible]	1	Conserved protein
OG001516[illegible]	6	Retrovirus-related Pol polyprotein from transposon TNT 1-94
OG001516[illegible]	7	Protein ANTAGONIST OF LIKE HETEROCHROMATIN PROTEIN 1
OG001517[illegible]	6	Conserved protein
OG001517[illegible]	6	Conserved protein
OG001517[illegible]	6	Conserved protein
OG001517[illegible]	6	Pentatricopeptide repeat-containing protein At4g32430
OG001517[illegible]	2	Conserved protein
OG001517[illegible]	7	Conserved protein
OG001517[illegible]	7	Conserved protein
OG001518[illegible]	6	Conserved protein
OG001518[illegible]	1	Conserved protein
OG001518[illegible]	4	Conserved protein
OG001518[illegible]	1	S-locus-specific glycoprotein S6
OG001518[illegible]	6	Conserved protein
OG001518[illegible]	2	Conserved protein
OG001518[illegible]	1	Conserved protein
OG001519[illegible]	4	Conserved protein
OG001519[illegible]	6	Conserved protein
OG001519[illegible]	7	ABC transporter C family member 4
OG001519[illegible]	2	Conserved protein
OG001519[illegible]	6	Conserved protein
OG001520[illegible]	5	Conserved protein
OG001520[illegible]	3	G-type lectin S-receptor-like serine/threonine-protein kinase At4g27290
OG001520[illegible]	6	Conserved protein
OG001527[illegible]	1	Pentatricopeptide repeat-containing protein At4g21300

OG0015398	1	Conserved protein
OG0015418	1	Conserved protein
OG0015442	5	Conserved protein
OG0015453	1	Heat shock cognate 70 kDa protein
OG0015491	5	Conserved protein
OG0015517	1	Disease resistance protein RPP2B
OG0015549	4	Berberine bridge enzyme-like 25
OG0015558	3	Conserved protein
OG0015565	1	Conserved protein
OG0015572	4	Cellulose synthase-like protein G1
OG0015573	1	Glutamate receptor 3.2
OG0015608	2	Conserved protein
OG0015640	6	Conserved protein
OG0015642	6	Sterol 3-beta-glucosyltransferase UGT80A2
OG0015643	4	Conserved protein
OG0015644	3	Conserved protein
OG0015645	6	Conserved protein
OG0015646	1	Conserved protein
OG0015648	6	Conserved protein
OG0015649	6	Receptor-like serine/threonine-protein kinase SD1-8
OG0015650	5	Conserved protein
OG0015651	5	G-type lectin S-receptor-like serine/threonine-protein kinase At4g27290
OG0015652	2	Conserved protein
OG0015653	3	Conserved protein
OG0015654	6	Conserved protein
OG0015655	6	Conserved protein
OG0015656	5	Conserved protein
OG0015658	1	Conserved protein
OG0015659	2	Conserved protein
OG0015660	6	Conserved protein

OG0015661	2	DExH-box ATP-dependent RNA helicase DExH14
OG0015662	6	Conserved protein
OG0015663	4	Homeobox-DDT domain protein RLT3
OG0015664	1	Conserved protein
OG0015665	4	Conserved protein
OG0015666	6	Conserved protein
OG0015667	1	Conserved protein
OG0015668	1	General transcription and DNA repair factor IIH subunit TFB1-1
OG0015672	4	Conserved protein
OG0015673	4	Conserved protein
OG0015674	1	Conserved protein
OG0015676	6	Conserved protein
OG0015679	1	Protein STABILIZED1
OG0015680	6	Conserved protein
OG0015685	5	B3 domain-containing transcription factor NGA4
OG0015689	6	Conserved protein
OG0015690	4	Probable leucine-rich repeat receptor-like protein kinase At2g33170
OG0015692	1	AP2-like ethylene-responsive transcription factor CRL5
OG0015696	1	Patatin-like protein 3
OG0015815	2	Pentatricopeptide repeat-containing protein At4g21300
OG0015891	1	Conserved protein
OG0015904	1	Conserved protein
OG0015907	2	Conserved protein
OG0015922	3	Conserved protein
OG0015948	1	Tyrosine-sulfated glycopeptide receptor 1
OG0015969	2	Conserved protein
OG0016002	1	Uncharacterized protein L728
OG0016026	1	Probable LRR receptor-like serine/threonine-protein kinase At1g06840
OG0016170	4	Conserved protein
OG0016171	4	Alcohol dehydrogenase-like 6

OG0016173	4	Conserved protein
OG0016174	2	Conserved protein
OG0016175	1	Conserved protein
OG0016176	1	Conserved protein
OG0016177	4	(-)-alpha-pinene synthase
OG0016178	1	Conserved protein
OG0016179	5	Conserved protein
OG0016180	3	Conserved protein
OG0016181	1	Bifunctional dethiobiotin synthetase/7,8-diamino-pelargonic acid aminotransferase
OG0016183	4	Conserved protein
OG0016185	2	Zinc finger BED domain-containing protein RICESLEEPER 2
OG0016188	5	Conserved protein
OG0016189	1	Conserved protein
OG0016190	5	Conserved protein
OG0016191	5	Sugar transporter ERD6-like 7
OG0016192	4	Mitochondrial substrate carrier family protein B
OG0016193	4	Conserved protein
OG0016194	5	ABC transporter B family member 26, chloroplastic
OG0016195	5	Conserved protein
OG0016196	3	Conserved protein
OG0016197	5	Conserved protein
OG0016199	1	Conserved protein
OG0016200	4	Glucose-6-phosphate isomerase, cytosolic
OG0016201	1	Conserved protein
OG0016202	5	Conserved protein
OG0016203	4	Conserved protein
OG0016204	5	Conserved protein
OG0016205	2	Conserved protein
OG0016207	1	Conserved protein
OG0016209	3	Protein ROOT HAIR DEFECTIVE 3 homolog 2

OG0016210	5	Nudix hydrolase 20, chloroplastic
OG0016216	5	Conserved protein
OG0016217	5	Ferric reduction oxidase 8
OG0016219	4	Conserved protein
OG0016220	2	Conserved protein
OG0016221	1	Conserved protein
OG0016222	4	Conserved protein
OG0016225	4	Conserved protein
OG0016226	1	Conserved protein
OG0016230	1	Conserved protein
OG0016231	4	Elongator complex protein 6
OG0016235	1	Conserved protein
OG0016238	1	7-deoxyloganetin glucosyltransferase
OG0016239	1	Putative F-box protein At3g21120
OG0016240	5	Conserved protein
OG0016241	4	Conserved protein
OG0016242	4	Conserved protein
OG0016244	1	Conserved protein
OG0016246	1	Conserved protein
OG0016249	1	Conserved protein
OG0016253	5	Conserved protein
OG0016377	2	Splicing factor SF3a60 homolog
OG0016383	1	Oligopeptide transporter 7
OG0016453	1	Tyrosine/DOPA decarboxylase 2
OG0016490	1	Conserved protein
OG0016637	1	Short-chain dehydrogenase TIC 32
OG0016651	2	Auxin response factor 22
OG0016686	1	Conserved protein
OG0016697	1	Putative receptor-like protein kinase At3g47110
OG0016736	2	Polyphenol oxidase latent form, chloroplastic

OG0016742	2	G-type lectin S-receptor-like serine/threonine-protein kinase LECRK4
OG0016752	1	Pyruvate decarboxylase 1
OG0016758	2	Conserved protein
OG0016762	1	Polygalacturonase
OG0016916	3	Conserved protein
OG0016917	3	Protein DJ-1 homolog D
OG0016918	1	Methyl-CpG-binding domain-containing protein 5
OG0016919	1	RING-H2 finger protein ATL72
OG0016920	3	Conserved protein
OG0016921	1	Agamous-like MADS-box protein AGL103
OG0016924	2	Conserved protein
OG0016925	4	Conserved protein
OG0016926	4	Conserved protein
OG0016927	3	Conserved protein
OG0016928	2	Conserved protein
OG0016930	3	Conserved protein
OG0016932	3	Conserved protein
OG0016933	4	Conserved protein
OG0016934	4	Conserved protein
OG0016935	2	Conserved protein
OG0016936	4	ABC transporter C family member 14
OG0016940	1	Homeobox-DDT domain protein RLT3
OG0016941	1	Conserved protein
OG0016942	3	Pentatricopeptide repeat-containing protein At2g13600
OG0016943	4	Retrovirus-related Pol polyprotein from transposon 17.6
OG0016944	4	Conserved protein
OG0016945	3	Conserved protein
OG0016946	2	Conserved protein
OG0016947	4	Conserved protein
OG0016949	1	Conserved protein

OG0016952	4	Conserved protein
OG0016953	4	Conserved protein
OG0016954	4	Conserved protein
OG0016955	4	Conserved protein
OG0016956	4	Conserved protein
OG0016961	3	Conserved protein
OG0016969	4	Activator of 90 kDa heat shock protein ATPase homolog
OG0016972	2	Anthocyanidin 3-O-glucoside 2"-O-glucosyltransferase
OG0016975	1	Protein DETOXIFICATION 19
OG0016979	3	Senescence-specific cysteine protease SAG12
OG0016985	1	DExH-box ATP-dependent RNA helicase DExH14
OG0016986	3	Mucin-19
OG0016992	4	Protein NETWORKED 1D
OG0016994	3	ABC transporter F family member 4
OG0016995	1	Conserved protein
OG0016996	1	AP-4 complex subunit epsilon
OG0017001	2	Auxin-induced protein 10A5
OG0017007	3	Conserved protein
OG0017008	2	NAC domain-containing protein 40
OG0017014	1	Flavonol 4'-sulfotransferase
OG0017017	2	Conserved protein
OG0017018	2	UDP-glycosyltransferase 74G1
OG0017020	2	Conserved protein
OG0017022	1	Conserved protein
OG0017028	2	Conserved protein
OG0017036	1	Conserved protein
OG0017040	1	Conserved protein
OG0017041	1	Conserved protein
OG0017045	4	STOREKEEPER protein
OG0017046	4	Conserved protein

OG0017047	4	Conserved protein
OG0017302	1	DNA topoisomerase 3-beta
OG0017349	1	Sucrose transport protein SUT4
OG0017621	2	Conserved protein
OG0017626	1	Conserved protein
OG0017673	1	Putative cytochrome P450 71A28
OG0017794	1	Leucine-rich repeat receptor protein kinase MSP1
OG0017842	1	Tyrosine/DOPA decarboxylase 1
OG0018117	2	Conserved protein
OG0018119	2	Conserved protein
OG0018120	3	Sterol 3-beta-glucosyltransferase UGT80A2
OG0018121	2	Conserved protein
OG0018122	1	NAC domain-containing protein 5
OG0018125	3	Conserved protein
OG0018126	3	Conserved protein
OG0018128	2	Conserved protein
OG0018130	1	Conserved protein
OG0018131	3	Conserved protein
OG0018132	3	Conserved protein
OG0018134	1	Conserved protein
OG0018140	3	Retrovirus-related Pol polyprotein from transposon TNT 1-94
OG0018142	3	WAT1-related protein At5g40230
OG0018145	1	Conserved protein
OG0018147	2	Receptor-like protein 35
OG0018149	1	Conserved protein
OG0018151	2	Conserved protein
OG0018152	1	Endochitinase CHI
OG0018153	3	Conserved protein
OG0018154	3	Conserved protein
OG0018155	2	Conserved protein

OG001815	1	G-type lectin S-receptor-like serine/threonine-protein kinase LECRK2
OG001816	3	Conserved protein
OG001816	2	Conserved protein
OG001816	3	Polygalacturonate 4-alpha-galacturonosyltransferase
OG001816	1	Anaphase-promoting complex subunit 2
OG001816	2	Conserved protein
OG001816	3	Conserved protein
OG001816	3	Ubiquitin carboxyl-terminal hydrolase 12
OG001816	1	Conserved protein
OG001816	2	Conserved protein
OG001817	2	Conserved protein
OG001817	1	Conserved protein
OG001817	1	Conserved protein
OG001817	2	Conserved protein
OG001817	3	Conserved protein
OG001817	3	Putative rRNA methylase YtqB
OG001817	3	Retrovirus-related Pol polyprotein from transposon RE1
OG001817	3	Conserved protein
OG001818	1	Probable receptor-like protein kinase At1g11050
OG001818	1	Retrovirus-related Pol polyprotein from transposon RE2
OG001819	3	Conserved protein
OG001819	2	Conserved protein
OG001819	2	Palmitoyl-monogalactosyldiacylglycerol delta-7 desaturase, chloroplastic
OG001820	3	L-type lectin-domain containing receptor kinase IX.1
OG001820	1	L-type lectin-domain containing receptor kinase IX.1
OG001820	3	Conserved protein
OG001820	3	3-hydroxyisobutyryl-CoA hydrolase-like protein 3, mitochondrial
OG001820	1	Pre-mRNA-splicing factor cef1
OG001821	2	Conserved protein
OG001821	1	Retrovirus-related Pol polyprotein from transposon TNT 1-94

OG0018217	1	Transcription factor TCP24
OG0018218	1	Conserved protein
OG0018222	3	Conserved protein
OG0018225	1	Probable BOI-related E3 ubiquitin-protein ligase 2
OG0018227	1	Conserved protein
OG0018229	2	Conserved protein
OG0018233	1	Endochitinase 1
OG0018234	1	Receptor-like protein EIX2
OG0018235	3	Conserved protein
OG0018237	3	Sister chromatid cohesion protein SCC2
OG0018246	1	Conserved protein
OG0018247	3	Ammonium transporter 1 member 2
OG0018248	3	Conserved protein
OG0018257	1	Conserved protein
OG0018258	1	Conserved protein
OG0018260	3	Conserved protein
OG0018261	1	THO complex subunit 7A
OG0018263	2	DExH-box ATP-dependent RNA helicase DExH14
OG0018270	1	Conserved protein
OG0018271	1	Conserved protein
OG0018274	3	Conserved protein
OG0018277	3	Conserved protein
OG0018278	2	DNA repair protein RAD5A
OG0018285	1	Conserved protein
OG0018286	1	Conserved protein
OG0018289	3	Protein tas
OG0018290	1	Conserved protein
OG0018297	3	Protein TAR1
OG0018298	3	Transcription initiation factor TFIID subunit 1
OG0018299	1	Conserved protein

OG0019266	1	Conserved protein
OG0019271	1	Conserved protein
OG0019485	1	Conserved protein
OG0019602	1	Conserved protein
OG0020025	1	Conserved protein
OG0020124	1	Conserved protein
OG0020215	1	60S ribosomal protein L18a-3
OG0020216	2	Conserved protein
OG0020218	1	Conserved protein
OG0020219	1	Conserved protein
OG0020220	2	Protein ACTIVITY OF BC1 COMPLEX KINASE 3
OG0020221	2	Sterol 3-beta-glucosyltransferase UGT80A2
OG0020222	2	Conserved protein
OG0020223	1	Histone acetyltransferase type B catalytic subunit
OG0020226	1	Conserved protein
OG0020227	1	Syntaxin-related protein KNOLLE
OG0020228	2	Kinesin-like protein KIN-10A
OG0020229	2	Conserved protein
OG0020231	2	Conserved protein
OG0020234	2	Conserved protein
OG0020236	2	Conserved protein
OG0020238	2	Conserved protein
OG0020239	2	Conserved protein
OG0020240	1	Conserved protein
OG0020241	1	Conserved protein
OG0020244	2	Conserved protein
OG0020245	2	Conserved protein
OG0020248	2	Conserved protein
OG0020249	2	Conserved protein
OG0020250	1	Zeta-carotene desaturase

OG0020252	2	Protein CHROMATIN REMODELING 20
OG0020259	2	Conserved protein
OG0020260	1	Pentatricopeptide repeat-containing protein At1g50270
OG0020261	2	Conserved protein
OG0020262	2	Myosin-13
OG0020265	2	Conserved protein
OG0020268	2	Conserved protein
OG0020270	2	Conserved protein
OG0020274	2	Conserved protein
OG0020276	2	Conserved protein
OG0020277	2	Conserved protein
OG0020279	2	Conserved protein
OG0020280	2	Polyphenol oxidase latent form, chloroplastic
OG0020281	2	Conserved protein
OG0020282	2	Conserved protein
OG0020283	2	Conserved protein
OG0020284	1	Conserved protein
OG0020287	2	Conserved protein
OG0020289	2	Conserved protein
OG0020290	1	Conserved protein
OG0020292	2	Heat shock cognate protein 80
OG0020295	1	Conserved protein
OG0020299	1	Probable inactive ATP-dependent zinc metalloprotease FTSHI 2, chloroplastic
OG0020300	2	Eukaryotic translation initiation factor 3 subunit A
OG0020301	1	Conserved protein
OG0020302	2	Conserved protein
OG0020303	2	Conserved protein
OG0020304	2	Putative pectinesterase 63
OG0020305	2	Conserved protein
OG0020309	2	Conserved protein

OG0020310	2	Conserved protein
OG0020311	1	Retrovirus-related Pol polyprotein from transposon RE1
OG0020312	2	Conserved protein
OG0020313	1	Conserved protein
OG0020316	2	Conserved protein
OG0020324	2	Conserved protein
OG0020330	1	Conserved protein
OG0020334	2	Conserved protein
OG0020335	2	Conserved protein
OG0020338	1	Conserved protein
OG0020339	2	Probable anion transporter 1, chloroplastic
OG0020344	2	7-deoxyloganetin glucosyltransferase
OG0020347	1	Conserved protein
OG0020348	1	Conserved protein
OG0020352	1	Conserved protein
OG0020353	2	Conserved protein
OG0020354	1	Conserved protein
OG0020355	2	Conserved protein
OG0020357	1	Conserved protein
OG0020362	2	Conserved protein
OG0020363	1	Conserved protein
OG0020364	1	Putative leucine-rich repeat receptor-like protein kinase At2g19210
OG0020368	2	UBP1-associated protein 2A
OG0020369	1	Conserved protein
OG0020371	2	Conserved protein
OG0020373	2	Conserved protein
OG0020374	2	Conserved protein
OG0020382	2	Sterol 3-beta-glucosyltransferase UGT80A2
OG0020384	1	Pentatricopeptide repeat-containing protein At4g32430, mitochondrial
OG0020388	2	Copia protein

OG002039[illegible]	2	Disease resistance protein RGA2
OG002039[illegible]	2	Conserved protein
OG002039[illegible]	2	Conserved protein
OG002039[illegible]	1	Glycine-rich RNA-binding protein 10
OG002039[illegible]	1	Conserved protein
OG002040[illegible]	2	Conserved protein
OG002040[illegible]	2	Conserved protein
OG002041[illegible]	1	Ubinuclein-1
OG002041[illegible]	1	Conserved protein
OG002041[illegible]	1	Conserved protein
OG002041[illegible]	1	Conserved protein
OG002041[illegible]	1	Aspartic proteinase-like protein 2
OG002042[illegible]	1	Conserved protein
OG002042[illegible]	2	Conserved protein
OG002042[illegible]	2	Conserved protein
OG002042[illegible]	2	Retrovirus-related Pol polyprotein from transposon RE1
OG002042[illegible]	1	Mitogen-activated protein kinase kinase kinase 1
OG002043[illegible]	2	Pentatricopeptide repeat-containing protein At4g32430, mitochondrial
OG002044[illegible]	2	Conserved protein
OG002044[illegible]	2	Conserved protein
OG002044[illegible]	1	Pyruvate decarboxylase 2
OG002044[illegible]	2	Pyruvate decarboxylase 2
OG002506[illegible]	1	Conserved protein
OG002507[illegible]	1	Conserved protein
OG002507[illegible]	1	Conserved protein
OG002507[illegible]	1	Conserved protein
OG002508[illegible]	1	Conserved protein
OG002508[illegible]	1	Conserved protein
OG002508[illegible]	1	Conserved protein
OG002508[illegible]	1	Conserved protein

OG0025088	1	Conserved protein
OG0025091	1	Proteasome subunit alpha type-7-A
OG0025095	1	Conserved protein
OG0025096	1	Conserved protein
OG0025097	1	Conserved protein
OG0025100	1	Conserved protein
OG0025101	1	Conserved protein
OG0025102	1	Conserved protein
OG0025103	1	Conserved protein
OG0025104	1	Conserved protein
OG0025110	1	Probable LRR receptor-like serine/threonine-protein kinase At3g47570
OG0025113	1	Conserved protein
OG0025114	1	Kinesin-like protein KIN-10A
OG0025120	1	ABC transporter C family MRP4
OG0025128	1	Conserved protein
OG0025130	1	Conserved protein
OG0025131	1	Primary amine oxidase 1
OG0025132	1	Peroxisomal (S)-2-hydroxy-acid oxidase GLO3
OG0025134	1	Conserved protein
OG0025138	1	Putative pentatricopeptide repeat-containing protein At2g01510
OG0025139	1	Conserved protein
OG0025140	1	Allantoinase
OG0025141	1	Conserved protein
OG0025144	1	Mitogen-activated protein kinase kinase 7
OG0025148	1	Protein TOPLESS
OG0025149	1	Conserved protein
OG0025150	1	Wall-associated receptor kinase-like 22
OG0025156	1	Conserved protein
OG0025157	1	Conserved protein
OG0025160	1	Conserved protein

OG0025161	1	Conserved protein
OG0025162	1	Conserved protein
OG0025163	1	Conserved protein
OG0025164	1	Conserved protein
OG0025165	1	Conserved protein
OG0025166	1	Ubiquitin carboxyl-terminal hydrolase 8
OG0025169	1	Conserved protein
OG0025170	1	Histone H3.3
OG0025176	1	Conserved protein
OG0025178	1	Conserved protein
OG0025185	1	3-hydroxyisobutyryl-CoA hydrolase-like protein 5
OG0025187	1	Pentatricopeptide repeat-containing protein At4g18520, chloroplastic
OG0025189	1	Beta-galactosidase 3
OG0025191	1	Conserved protein
OG0025194	1	Conserved protein
OG0025200	1	30S ribosomal protein S1, chloroplastic
OG0025204	1	Conserved protein
OG0025205	1	Glucose-6-phosphate isomerase, cytosolic
OG0025208	1	Conserved protein
OG0025211	1	Conserved protein
OG0025212	1	Conserved protein
OG0025215	1	Conserved protein
OG0025217	1	Nucleoside diphosphate kinase 1
OG0025222	1	Conserved protein
OG0025224	1	Conserved protein
OG0025227	1	Conserved protein
OG0025229	1	Conserved protein
OG0025231	1	Conserved protein
OG0025232	1	Conserved protein
OG0025234	1	Haloacid dehalogenase-like hydrolase domain-containing protein At2g33255

OG0025237	1	Conserved protein
OG0025238	1	Conserved protein
OG0025240	1	Pentatricopeptide repeat-containing protein At5g02860
OG0025244	1	Conserved protein
OG0025246	1	Kinesin-like protein KIN-10A
OG0025247	1	Conserved protein
OG0025252	1	Conserved protein
OG0025253	1	Conserved protein
OG0025254	1	Cytochrome P450 705A5
OG0025255	1	Conserved protein
OG0025256	1	Conserved protein
OG0025258	1	Conserved protein
OG0025261	1	Transposon Ty3-G Gag-Pol polyprotein
OG0025268	1	Conserved protein
OG0025272	1	Equilibrative nucleotide transporter 3
OG0025273	1	Conserved protein
OG0025275	1	Conserved protein
OG0025279	1	Conserved protein
OG0025280	1	Conserved protein
OG0025289	1	Ferric reduction oxidase 8, mitochondrial
OG0025290	1	NDR1/HIN1-like protein 10
OG0025292	1	Conserved protein
OG0025297	1	7-deoxyloganetin glucosyltransferase
OG0025299	1	Conserved protein
OG0025300	1	Conserved protein
OG0025303	1	Conserved protein
OG0025304	1	Peroxisomal (S)-2-hydroxy-acid oxidase GLO4
OG0025311	1	Conserved protein
OG0025312	1	Conserved protein
OG0025316	1	Shikimate O-hydroxycinnamoyltransferase

OG0025317	1	Conserved protein
OG0025321	1	Conserved protein
OG0025322	1	Conserved protein
OG0025327	1	Conserved protein
OG0025328	1	Conserved protein
OG0025329	1	Geranylgeranyl transferase type-2 subunit alpha 1
OG0025339	1	Conserved protein
OG0025340	1	Conserved protein
OG0025341	1	Conserved protein
OG0025343	1	Conserved protein
OG0025344	1	Retrovirus-related Pol polyprotein from transposon RE2
OG0025345	1	Conserved protein
OG0025346	1	Conserved protein
OG0025347	1	Conserved protein
OG0025348	1	Conserved protein
OG0025349	1	Conserved protein
OG0025355	1	G-type lectin S-receptor-like serine/threonine-protein kinase At4g27290
OG0025369	1	Arginase 1, mitochondrial
OG0025370	1	Conserved protein
OG0025376	1	GDSL esterase/lipase CPRD49
OG0025378	1	Probable LRR receptor-like serine/threonine-protein kinase RFK1
OG0025379	1	Conserved protein
OG0025383	1	Sister chromatid cohesion protein SCC2
OG0025384	1	Chloroplastic group IIA intron splicing facilitator CRS1
OG0025385	1	Uncharacterized AAA domain-containing protein C24B10.10c
OG0025396	1	Conserved protein
OG0025397	1	Conserved protein
OG0025401	1	Conserved protein
OG0025402	1	Conserved protein
OG0025404	1	Protein CYPRO4

OG0025405	1	O-fucosyltransferase 16
OG0025407	1	Monooxygenase 3
OG0025408	1	Conserved protein
OG0025409	1	Conserved protein
OG0025412	1	Conserved protein
OG0025419	1	Retrovirus-related Pol polyprotein from transposon RE1
OG0025422	1	Conserved protein
OG0025425	1	Conserved protein
OG0025427	1	Conserved protein
OG0025428	1	Calcium-transporting ATPase 8, plasma membrane-type
OG0025434	1	DEAD-box ATP-dependent RNA helicase 38
OG0025437	1	Conserved protein
OG0025438	1	Conserved protein
OG0025441	1	Conserved protein
OG0025446	1	Conserved protein
OG0025448	1	Conserved protein
OG0025454	1	DNA-(apurinic or apyrimidinic site) lyase
OG0025455	1	Conserved protein
OG0025462	1	Conserved protein
OG0025465	1	Conserved protein
OG0025467	1	Probable inactive leucine-rich repeat receptor-like protein kinase At3g03770
OG0025468	1	Conserved protein
OG0025469	1	Conserved protein
OG0025472	1	Cytochrome P450 93A3
OG0025476	1	Conserved protein
OG0025479	1	Probable acyl-activating enzyme 2
OG0025486	1	Conserved protein
OG0025488	1	Pentatricopeptide repeat-containing protein At4g32430
OG0025491	1	Conserved protein
OG0025494	1	2-alkenal reductase (NADP(+)-dependent)

OG002549[illegible]	1	Threonine--tRNA ligase, cytoplasmic
OG002549[illegible]	1	Conserved protein
OG002550[illegible]	1	Conserved protein
OG002550[illegible]	1	Pyruvate decarboxylase 1
OG002550[illegible]	1	Conserved protein
OG002551[illegible]	1	Uncharacterized aarF domain-containing protein kinase At1g71810

Table S12. KEGG enrichment analysis of the genes from expanded families

ID	Description	GeneRati	BgRatio	pvalue	KOID
ko04626	Plant-pathogen interaction	8/167	29/2390	0.00058	K04079/K05391/K13420/K13448/K13449/K13457/K13459/K18875
ko00940	Phenylpropanoid biosynthesis	6/167	20/2390	0.00181	K09754/K09755/K12356/K13065/K22395/K23260
ko00941	Flavonoid biosynthesis	5/167	14/2390	0.00187	K01859/K05278/K09754/K13065/K23179
ko00040	Pentose and glucuronate interconversions	5/167	17/2390	0.00487	K00699/K01051/K01184/K01213/K01728
ko03020	RNA polymerase	6/167	30/2390	0.01545	K03000/K03013/K03018/K03042/K03046/K03049
ko00350	Tyrosine metabolism	4/167	18/2390	0.03248	K00121/K00276/K00422/K18857
ko01110	Biosynthesis of secondary metabolites	39/167	424/2390	0.0342	K00016/K00121/K00276/K00422/K00591/K00699/K00930/K01568/K01581/K01647/K01653/K01755/K01756/K01785/K01803/K01845/K01859/K01961/K02259/K04123/K05278/K08248/K09754/K09755/K09828/K09840/K12153/K13065/K13679/K13789/K14173/K15397/K15919/K17911/K18693/K18857/K20659/K20986/K22395
ko00010	Glycolysis / Gluconeogenesis	6/167	36/2390	0.03585	K00016/K00121/K01568/K01785/K01803/K18857

Table S13.　GO enrichment analysis of the apricot-specific genes

GO ID	Class	p-value	GeneRatio	BgRatio	Description	Gene ID
GO:08272	P	6.04E-09	13/444	38/9718	sulfate transport	PARG19232|PARG19341|PARG27925|PARG25248|PARG19236|PARG18772|PARG19339|PARG30247|PARG11755|PARG30250|PARG13307|PARG27786|PARG28668
GO:15698	P	3.39E-06	13/444	62/9718	inorganic anion transport	PARG19232|PARG19341|PARG27925|PARG25248|PARG19236|PARG18772|PARG19339|PARG30247|PARG11755|PARG30250|PARG13307|PARG27786|PARG28668
GO:06820	P	6.10E-06	14/444	75/9718	anion transport	PARG19232|PARG19341|PARG27925|PARG25248|PARG19236|PARG18772|PARG19339|PARG30247|PARG11755|PARG30250|PARG13307|PARG13826|PARG27786|PARG28668
GO:55114	P	1.79E-05	90/444	1291/9718	oxidation reduction	PARG03408|PARG25751|PARG25079|PARG13810|PARG13935|PARG28186|PARG05145|PARG12924|PARG28188|PARG09387|PARG08176|PARG05545|PARG21438|PARG00770|PARG28227|PARG19481|PARG29714|PARG09502|PARG21632|PARG07568|PARG07569|PARG24436|PARG00325|PARG02503|PARG22133|PARG22372|PARG17782|PARG17787|PARG15168|PARG17788|PARG16854|PARG16417|PARG09897|PARG13748|PARG06025|PARG10165|PARG22619|PARG26253|PARG10163|PARG22859|PARG29962|PARG29965|PARG23506|PARG21528|PARG06026|PARG02502|PARG22898|PARG17350|PARG21530|PARG17075|PARG26824|PARG19252|PARG15096|PARG15098|PARG08917|PARG17154|PARG23472|PARG11723|PARG11167|PARG08797|PARG29133|PARG06453|PARG02131|PARG26341|PARG08870|PARG20724|PARG26421|PARG21616|PARG24258|PARG24452|PARG24572|PARG24571|PARG15102|PARG13523|PARG13524|PARG12955|

						PARG10188|PARG13407|PARG13969|PARG13408|PARG26395|PARG03371|PARG00381|PARG11075|PARG13808|PARG25907|PARG27003|PARG07736|PARG11070|PARG25229
GO:55085	P	5.12E-05	56/444	720/9718	transmembrane transport	PARG19232|PARG28905|PARG19236|PARG24740|PARG19510|PARG18467|PARG12882|PARG16524|PARG16887|PARG19917|PARG09347|PARG08412|PARG03001|PARG22209|PARG28668|PARG06559|PARG24504|PARG03885|PARG23743|PARG25248|PARG18311|PARG20073|PARG25167|PARG08321|PARG13307|PARG03990|PARG00040|PARG27786|PARG03992|PARG00762|PARG20158|PARG24756|PARG20682|PARG16382|PARG14121|PARG19339|PARG30247|PARG19811|PARG16346|PARG16349|PARG15139|PARG05680|PARG09484|PARG20925|PARG29499|PARG26862|PARG19341|PARG27925|PARG24774|PARG12591|PARG18772|PARG25263|PARG11755|PARG28182|PARG30250|PARG14936
GO:09308	P	4.11E-04	28/444	309/9718	amine metabolic process	PARG03408|PARG19252|PARG24422|PARG12142|PARG11847|PARG19516|PARG11200|PARG05723|PARG18033|PARG00105|PARG24572|PARG24571|PARG16212|PARG11857|PARG18039|PARG16119|PARG28293|PARG10202|PARG11851|PARG09751|PARG14812|PARG11199|PARG04086|PARG23805|PARG29962|PARG29965|PARG23506|PARG04168
GO:16021	C	1.71E-05	64/165	895/3680	integral to membrane	PARG19232|PARG28905|PARG25873|PARG18469|PARG15597|PARG12321|PARG12882|PARG16524|PARG16887|PARG10775|PARG11466|PARG12328|PARG19917|PARG01181|PARG08412|PARG22926|PARG22725|PARG03001|PARG28668|PARG06559|PARG03885|PARG06558|PARG23743|PARG18311|PARG20073|PARG25167|PARG13307|PARG05134|PARG03990|PARG26298|PARG27786|PARG03992|PARG2

						0158|PARG06989|PARG20166|PARG20682|PARG20167|PARG03508|PARG19339|PARG30247|PARG19811|PARG19215|PARG16346|PARG16349|PARG15139|PARG05680|PARG06337|PARG26783|PARG26862|PARG21536|PARG19341|PARG27925|PARG20013|PARG24774|PARG24254|PARG02606|PARG18772|PARG11755|PARG28182|PARG10984|PARG30250|PARG14936|PARG22635|PARG02248
GO:16020	C	1.75E-05	108/165	1822/3680	Membrane	PARG18469|PARG15597|PARG11947|PARG18467|PARG12882|PARG01181|PARG09347|PARG08412|PARG22725|PARG03001|PARG22209|PARG28668|PARG24504|PARG03885|PARG25323|PARG25167|PARG00807|PARG24072|PARG05134|PARG03990|PARG03992|PARG00762|PARG20158|PARG06989|PARG00246|PARG21524|PARG24756|PARG27989|PARG16382|PARG14121|PARG19811|PARG05680|PARG29690|PARG09484|PARG20925|PARG26862|PARG20013|PARG20376|PARG12591|PARG02606|PARG12871|PARG10984|PARG14936|PARG01073|PARG00785|PARG19232|PARG28905|PARG25873|PARG19236|PARG19510|PARG12321|PARG16524|PARG12445|PARG16887|PARG10775|PARG11466|PARG12328|PARG19917|PARG21438|PARG22926|PARG29832|PARG22767|PARG06559|PARG06558|PARG22258|PARG23743|PARG25765|PARG25248|PARG18311|PARG20073|PARG13144|PARG08321|PARG13307|PARG13826|PARG26298|PARG00040|PARG27786|PARG06664|PARG20166|PARG20682|PARG15491|PARG20167|PARG14162|PARG03508|PARG20681|PARG19339|PARG30247|PARG02819|PARG19215|PARG16346|PARG16349|PARG15139|PARG29499|PARG06337|PARG26783|PARG21536|PARG18131|PARG19341|PARG27925|PARG30419|PARG24774|PARG24254|PARG18772|PARG11755|PARG28182|PARG30250|PARG22635|PARG02248

GO:31224	C	2.32E-05	64/165	903/3680	intrinsic to membrane	PARG19232|PARG28905|PARG25873|PARG18469|PARG15597|PARG12321|PARG12882|PARG16524|PARG16887|PARG10775|PARG11466|PARG12328|PARG19917|PARG01181|PARG08412|PARG22926|PARG22725|PARG03001|PARG28668|PARG06559|PARG03885|PARG06558|PARG23743|PARG18311|PARG20073|PARG25167|PARG13307|PARG05134|PARG03990|PARG26298|PARG27786|PARG03992|PARG20158|PARG06989|PARG20166|PARG20682|PARG20167|PARG03508|PARG19339|PARG30247|PARG19811|PARG19215|PARG16346|PARG16349|PARG15139|PARG05680|PARG06337|PARG26783|PARG26862|PARG21536|PARG19341|PARG27925|PARG20013|PARG24774|PARG24254|PARG02606|PARG18772|PARG11755|PARG28182|PARG10984|PARG30250|PARG14936|PARG22635|PARG02248
GO:09507	C	6.48E-05	10/165	51/3680	Chloroplast	PARG19232|PARG30250|PARG19341|PARG13307|PARG25248|PARG19339|PARG30247|PARG27786|PARG28668|PARG11755
GO:09536	C	9.15E-05	10/165	53/3680	Plastid	PARG19232|PARG30250|PARG19341|PARG13307|PARG25248|PARG19339|PARG30247|PARG27786|PARG28668|PARG11755
GO:08271	F	3.90E-09	13/665	37/14688	secondary active sulfate transmembrane transporter activity	PARG19232|PARG19341|PARG27925|PARG25248|PARG19236|PARG18772|PARG19339|PARG30247|PARG11755|PARG30250|PARG13307|PARG27786|PARG28668
GO:15116	F	5.68E-09	13/665	38/14688	sulfate transmembrane transporter activity	PARG19232|PARG19341|PARG27925|PARG25248|PARG19236|PARG18772|PARG19339|PARG30247|PARG11755|PARG30250|PARG13307|PARG27786|PARG28668
GO:05215	F	3.00E-07	74/665	895/14688	transporter activity	PARG19232|PARG28905|PARG19236|PARG24740|PARG22165|PARG19510|PARG18469|PARG13451|PARG18467|PARG12321|PARG12882|PARG16524|PARG16887|PARG11466|PARG12328|PARG19917|PARG09347|PARG08412|PARG22725|PARG03001|PARG22209|PARG28

						668|PARG06559|PARG24504|PARG03885|PARG23743|PARG25248|PARG18311|PARG20073|PARG25167|PARG18310|PARG24196|PARG08321|PARG13307|PARG03990|PARG26298|PARG00040|PARG27786|PARG03992|PARG00762|PARG20158|PARG24756|PARG02786|PARG16382|PARG14121|PARG19339|PARG30247|PARG19811|PARG02819|PARG19215|PARG16346|PARG16349|PARG15139|PARG05680|PARG29690|PARG09484|PARG20925|PARG29499|PARG26783|PARG18131|PARG19341|PARG27925|PARG30419|PARG20376|PARG24774|PARG24254|PARG12591|PARG02606|PARG18772|PARG25263|PARG11755|PARG30250|PARG14936|PARG21822
GO:15103	F	4.71E-07	13/665	53/14688	inorganic anion transmembrane transporter activity	PARG19232|PARG19341|PARG27925|PARG25248|PARG19236|PARG18772|PARG19339|PARG30247|PARG11755|PARG30250|PARG13307|PARG27786|PARG28668
GO:22857	F	2.35E-06	68/665	844/14688	transmembrane transporter activity	PARG19232|PARG28905|PARG19236|PARG24740|PARG22165|PARG19510|PARG18467|PARG12321|PARG12882|PARG16524|PARG16887|PARG12328|PARG19917|PARG09347|PARG08412|PARG03001|PARG22209|PARG28668|PARG06559|PARG24504|PARG03885|PARG23743|PARG25248|PARG18311|PARG20073|PARG25167|PARG18310|PARG24196|PARG08321|PARG13307|PARG03990|PARG26298|PARG00040|PARG27786|PARG03992|PARG00762|PARG20158|PARG24756|PARG16382|PARG14121|PARG19339|PARG30247|PARG19811|PARG02819|PARG19215|PARG16346|PARG16349|PARG15139|PARG05680|PARG29690|PARG09484|PARG20925|PARG29499|PARG26783|PARG18131|PARG19341|PARG27925|PARG30419|PARG20376|PARG24774|PARG24254|PARG02606|PARG18772|PARG25263|PARG11755|PARG30250|PARG14936|PARG21822

GO:22804	F	8.21E-06	30/665	276/14688	active transmembrane transporter activity	PARG19232|PARG19236|PARG19510|PARG14121|PARG19339|PARG30247|PARG19811|PARG02819|PARG12882|PARG16346|PARG16349|PARG15139|PARG16524|PARG19917|PARG03001|PARG22209|PARG28668|PARG06559|PARG19341|PARG27925|PARG25248|PARG24774|PARG25167|PARG18772|PARG11755|PARG30250|PARG14936|PARG13307|PARG27786|PARG20158
GO:20037	F	4.19E-05	37/665	406/14688	heme binding	PARG26824|PARG25751|PARG08917|PARG25079|PARG11167|PARG05145|PARG12924|PARG29133|PARG03363|PARG06453|PARG02131|PARG26341|PARG20724|PARG26421|PARG00770|PARG21616|PARG19481|PARG09502|PARG07568|PARG07569|PARG00325|PARG22133|PARG24452|PARG22372|PARG17782|PARG17787|PARG15168|PARG17788|PARG10188|PARG10165|PARG26395|PARG22619|PARG10163|PARG00381|PARG25907|PARG07736|PARG22898
GO:46906	F	4.19E-05	37/665	406/14688	tetrapyrrole binding	PARG26824|PARG25751|PARG08917|PARG25079|PARG11167|PARG05145|PARG12924|PARG29133|PARG03363|PARG06453|PARG02131|PARG26341|PARG20724|PARG26421|PARG00770|PARG21616|PARG19481|PARG09502|PARG07568|PARG07569|PARG00325|PARG22133|PARG24452|PARG22372|PARG17782|PARG17787|PARG15168|PARG17788|PARG10188|PARG10165|PARG26395|PARG22619|PARG10163|PARG00381|PARG25907|PARG07736|PARG22898
GO:08509	F	4.35E-05	13/665	78/14688	anion transmembrane transporter activity	PARG19232|PARG19341|PARG27925|PARG25248|PARG19236|PARG18772|PARG19339|PARG30247|PARG11755|PARG30250|PARG13307|PARG27786|PARG28668
GO:16491	F	1.20E-04	94/665	1428/14688	oxidoreductase activity	PARG03408|PARG12280|PARG25751|PARG25079|PARG18941|PARG18988|PARG13810|PARG13935|PARG05145|PARG12924|PARG28188|PARG09387|PARG08176|PARG05545|PARG21438|PARG00770|PA

						RG19481|PARG09502|PARG21632|PARG07568|PARG07569|PARG24436|PARG00325|PARG02503|PARG22133|PARG22372|PARG17782|PARG17787|PARG15168|PARG17788|PARG16854|PARG16417|PARG09897|PARG06025|PARG10165|PARG22619|PARG10163|PARG22859|PARG29962|PARG29965|PARG23506|PARG21528|PARG06026|PARG02502|PARG22898|PARG17350|PARG21530|PARG17075|PARG26824|PARG19252|PARG15096|PARG15098|PARG08917|PARG17154|PARG23472|PARG11723|PARG07222|PARG11167|PARG08797|PARG29133|PARG06453|PARG02131|PARG26341|PARG08870|PARG20724|PARG26421|PARG21616|PARG24258|PARG25983|PARG24452|PARG24572|PARG24571|PARG15102|PARG15982|PARG13523|PARG13524|PARG12955|PARG10188|PARG13407|PARG13969|PARG13408|PARG26395|PARG03371|PARG00381|PARG11075|PARG13808|PARG03531|PARG25907|PARG27003|PARG07736|PARG11070|PARG03536|PARG03534|PARG25229
GO:03860	F	1.38E-04	6/665	19/14688	3-hydroxyisobutyryl-CoA hydrolase activity	PARG18609|PARG28683|PARG18311|PARG18613|PARG16524|PARG06558
GO:16887	F	1.51E-04	27/665	277/14688	ATPase activity	PARG02999|PARG16521|PARG19811|PARG12882|PARG16346|PARG16349|PARG15139|PARG16524|PARG11466|PARG19917|PARG16508|PARG03003|PARG03001|PARG28823|PARG00772|PARG20029|PARG06559|PARG20034|PARG20014|PARG24774|PARG25167|PARG16511|PARG14936|PARG01073|PARG29267|PARG20038|PARG20158
GO:16817	F	2.85E-04	44/665	562/14688	hydrolase activity, acting on acid anhydrides	PARG02999|PARG27718|PARG24044|PARG16521|PARG19811|PARG19931|PARG02819|PARG12882|PARG16346|PARG16349|PARG15139|PARG16524|PARG11466|PARG19917|PARG16508|PARG03003|PA

<table>
<tr><td></td><td></td><td></td><td></td><td></td><td></td><td>RG27059|PARG03001|PARG28823|PARG00772|PARG02015|PARG20029|PARG06559|PARG06855|PARG20034|PARG20014|PARG00789|PARG24774|PARG18630|PARG16692|PARG20252|PARG25167|PARG15022|PARG18635|PARG16511|PARG14936|PARG07179|PARG04083|PARG01073|PARG29267|PARG28777|PARG11193|PARG20038|PARG20158</td></tr>
<tr><td>GO:17111</td><td>F</td><td>3.15E-04</td><td>42/665</td><td>531/14688</td><td>nucleoside-triphosphatase activity</td><td>PARG02999|PARG27718|PARG24044|PARG16521|PARG19811|PARG12882|PARG16346|PARG16349|PARG15139|PARG16524|PARG11466|PARG19917|PARG16508|PARG03003|PARG27059|PARG03001|PARG28823|PARG00772|PARG02015|PARG20029|PARG06559|PARG06855|PARG20034|PARG20014|PARG00789|PARG24774|PARG18630|PARG16692|PARG20252|PARG25167|PARG15022|PARG18635|PARG16511|PARG14936|PARG07179|PARG04083|PARG01073|PARG29267|PARG28777|PARG11193|PARG20038|PARG20158</td></tr>
<tr><td>GO:48038</td><td>F</td><td>3.37E-04</td><td>6/665</td><td>22/14688</td><td>quinone binding</td><td>PARG03408|PARG17154|PARG24572|PARG24571|PARG29962|PARG29965</td></tr>
<tr><td>GO:16462</td><td>F</td><td>3.56E-04</td><td>43/665</td><td>551/14688</td><td>pyrophosphatase activity</td><td>PARG02999|PARG27718|PARG24044|PARG16521|PARG19811|PARG02819|PARG12882|PARG16346|PARG16349|PARG15139|PARG16524|PARG11466|PARG19917|PARG16508|PARG03003|PARG27059|PARG03001|PARG28823|PARG00772|PARG02015|PARG20029|PARG06559|PARG06855|PARG20034|PARG20014|PARG00789|PARG24774|PARG18630|PARG16692|PARG20252|PARG25167|PARG15022|PARG18635|PARG16511|PARG14936|PARG07179|PARG04083|PARG01073|PARG29267|PARG28777|PARG11193|PARG20038|PARG20158</td></tr>
<tr><td>GO:05506</td><td>F</td><td>4.37E-04</td><td>37/665</td><td>456/14688</td><td>iron ion binding</td><td>PARG26824|PARG25751|PARG08917|PARG25079|PARG11167|PARG05145|PARG12924|PARG29133|PARG03363|PARG06453|PARG021</td></tr>
</table>

						31|PARG26341|PARG20724|PARG26421|PARG00770|PARG21616|PARG19481|PARG09502|PARG07568|PARG07569|PARG00325|PARG22133|PARG24452|PARG22372|PARG17782|PARG17787|PARG15168|PARG17788|PARG10188|PARG10165|PARG26395|PARG22619|PARG10163|PARG00381|PARG25907|PARG07736|PARG22898
GO:16818	F	4.64E-04	43/665	558/14688	hydrolase activity, acting on acid anhydrides, in phosphorus-containing anhydrides	PARG02999|PARG27718|PARG24044|PARG16521|PARG19811|PARG02819|PARG12882|PARG16346|PARG16349|PARG15139|PARG16524|PARG11466|PARG19917|PARG16508|PARG03003|PARG27059|PARG03001|PARG28823|PARG00772|PARG02015|PARG20029|PARG06559|PARG06855|PARG20034|PARG20014|PARG00789|PARG24774|PARG18630|PARG16692|PARG20252|PARG25167|PARG15022|PARG18635|PARG16511|PARG14936|PARG07179|PARG04083|PARG01073|PARG29267|PARG28777|PARG11193|PARG20038|PARG20158
GO:16638	F	7.12E-04	6/665	25/14688	oxidoreductase activity, acting on the CH-NH2 group of donors	PARG03408|PARG24572|PARG24571|PARG29962|PARG29965|PARG23506
GO:22892	F	7.19E-04	33/665	402/14688	substrate-specific transporter activity	PARG19232|PARG19236|PARG22165|PARG18469|PARG19339|PARG13451|PARG30247|PARG18467|PARG12321|PARG19215|PARG16887|PARG11466|PARG29690|PARG12328|PARG09484|PARG22725|PARG28668|PARG24504|PARG18131|PARG19341|PARG27925|PARG30419|PARG25248|PARG20376|PARG18772|PARG11755|PARG30250|PARG13307|PARG03990|PARG27786|PARG03992|PARG20158|PARG02786
GO:08131	F	7.36E-04	5/665	17/14688	amine oxidase activity	PARG03408|PARG24572|PARG24571|PARG29962|PARG29965
GO:03824	F	7.96E-04	374/665	7372/1468	catalytic activity	PARG12882|PARG18988|PARG28780|PARG08493|PARG30068|PAR

G26122|PARG01063|PARG28786|PARG05545|PARG03003|PARG030
01|PARG00770|PARG06994|PARG03249|PARG28668|PARG00772|PA
RG04218|PARG24989|PARG20029|PARG20034|PARG25843|PARG18
630|PARG01979|PARG16692|PARG20274|PARG25167|PARG21483|P
ARG12097|PARG17782|PARG17787|PARG18635|PARG16212|PARG1
8877|PARG17788|PARG13741|PARG24072|PARG15009|PARG12658|
PARG08126|PARG06067|PARG26495|PARG29641|PARG25609|PAR
G25608|PARG07716|PARG25606|PARG20038|PARG01851|PARG201
58|PARG06989|PARG20831|PARG17075|PARG25139|PARG19252|PA
RG26904|PARG25255|PARG13394|PARG24044|PARG24165|PARG19
811|PARG25252|PARG19931|PARG30048|PARG11206|PARG18609|P
ARG11200|PARG11684|PARG26341|PARG05561|PARG26221|PARG2
7311|PARG27432|PARG07988|PARG20803|PARG27435|PARG23518|
PARG20488|PARG00797|PARG20014|PARG21586|PARG00789|PAR
G20252|PARG14255|PARG15102|PARG18613|PARG08271|PARG082
72|PARG12871|PARG12873|PARG12875|PARG14812|PARG14936|PA
RG07179|PARG13969|PARG01073|PARG08384|PARG03371|PARG02
162|PARG07736|PARG28777|PARG07979|PARG05311|PARG06649|P
ARG28657|PARG02962|PARG21469|PARG02999|PARG24422|PARG2
3576|PARG24300|PARG25751|PARG25079|PARG04938|PARG21271|
PARG10811|PARG12445|PARG19516|PARG11466|PARG09387|PAR
G08176|PARG07880|PARG29311|PARG26049|PARG01663|PARG065
59|PARG06558|PARG24436|PARG27704|PARG00325|PARG25765|PA
RG02503|PARG18311|PARG22133|PARG22372|PARG10804|PARG15
168|PARG22491|PARG18559|PARG05210|PARG06545|PARG26732|P
ARG02502|PARG26824|PARG18880|PARG20282|PARG16346|PARG1

6349|PARG15139|PARG10832|PARG02091|PARG11124|PARG28683|
PARG06453|PARG29892|PARG08870|PARG05127|PARG20724|PAR
G06337|PARG04159|PARG25615|PARG23557|PARG06217|PARG001
05|PARG05915|PARG25621|PARG24774|PARG05913|PARG25983|PA
RG18771|PARG15022|PARG16234|PARG00907|PARG12795|PARG10
821|PARG13523|PARG19747|PARG16119|PARG13524|PARG08983|P
ARG13407|PARG29541|PARG13408|PARG26395|PARG08980|PARG0
7779|PARG27487|PARG27003|PARG21028|PARG02766|PARG05949|
PARG03408|PARG25678|PARG00812|PARG10619|PARG25673|PAR
G00816|PARG17375|PARG16166|PARG05145|PARG12924|PARG034
00|PARG27059|PARG28823|PARG09502|PARG21632|PARG04975|PA
RG07568|PARG01468|PARG07569|PARG27869|PARG24358|PARG00
801|PARG20552|PARG25323|PARG24353|PARG22173|PARG16854|P
ARG10165|PARG10285|PARG22619|PARG10163|PARG22859|PARG2
4915|PARG04168|PARG21528|PARG17390|PARG08523|PARG27866|
PARG00489|PARG04843|PARG00246|PARG27989|PARG25536|PAR
G17350|PARG27718|PARG28809|PARG23472|PARG22263|PARG118
48|PARG25890|PARG11847|PARG16029|PARG11723|PARG07222|PA
RG12108|PARG11167|PARG08797|PARG27031|PARG08551|PARG21
616|PARG08798|PARG01124|PARG05723|PARG06807|PARG05715|P
ARG16035|PARG24452|PARG19300|PARG25661|PARG24572|PARG2
4571|PARG15982|PARG13563|PARG13567|PARG16953|PARG11039|
PARG13569|PARG09751|PARG29582|PARG10188|PARG05156|PAR
G00381|PARG23805|PARG05952|PARG03531|PARG28378|PARG050
39|PARG03536|PARG03534|PARG19232|PARG19351|PARG12280|PA
RG16521|PARG18941|PARG11988|PARG12047|PARG18705|PARG16

						524|PARG18702|PARG13810|PARG14903|PARG19917|PARG18706|PARG13935|PARG28188|PARG04654|PARG21438|PARG28625|PARG22767|PARG19481|PARG18033|PARG19240|PARG12054|PARG16776|PARG11857|PARG18710|PARG18039|PARG16417|PARG11851|PARG09897|PARG04083|PARG06025|PARG29045|PARG04086|PARG07231|PARG29962|PARG29965|PARG27786|PARG23506|PARG26214|PARG06026|PARG27425|PARG07237|PARG02469|PARG21205|PARG22898|PARG21530|PARG01964|PARG15096|PARG15098|PARG08917|PARG17154|PARG24363|PARG12142|PARG30247|PARG12022|PARG02819|PARG24480|PARG10557|PARG10554|PARG29251|PARG29252|PARG16508|PARG29133|PARG02131|PARG27870|PARG22628|PARG26421|PARG06731|PARG21896|PARG05768|PARG02015|PARG07701|PARG06855|PARG24258|PARG05639|PARG25586|PARG16511|PARG11875|PARG28293|PARG07496|PARG12955|PARG11199|PARG10780|PARG29267|PARG11075|PARG13808|PARG25907|PARG11193|PARG22879|PARG28853|PARG11070|PARG03216|PARG25229
GO:51879	F	8.63E-04	3/665	5/14688	Hsp90 protein binding	PARG23189|PARG09744|PARG16170
GO:16705	F	8.80E-04	31/665	374/14688	oxidoreductase activity, acting on paired donors, with incorporation or reduction of molecular oxygen	PARG26824|PARG25751|PARG08917|PARG25079|PARG11167|PARG05145|PARG06453|PARG02131|PARG26341|PARG20724|PARG26421|PARG00770|PARG21616|PARG19481|PARG09502|PARG07568|PARG07569|PARG00325|PARG24452|PARG22372|PARG17782|PARG17787|PARG15168|PARG17788|PARG10188|PARG10165|PARG26395|PARG22619|PARG10163|PARG07736|PARG22898
GO:15399	F	8.90E-04	14/665	118/14688	primary active transmembrane transporter activity	PARG24774|PARG25167|PARG19811|PARG02819|PARG12882|PARG16346|PARG16349|PARG15139|PARG16524|PARG14936|PARG19917|PARG03001|PARG20158|PARG06559

GO:15405	F	8.90E-04	14/665	118/14688	P-P-bond-hydrolysis-driven transmembrane transporter activity	PARG24774|PARG25167|PARG19811|PARG02819|PARG12882|PARG16346|PARG16349|PARG15139|PARG16524|PARG14936|PARG19917|PARG03001|PARG20158|PARG06559
GO:16289	F	8.91E-04	6/665	26/14688	CoA hydrolase activity	PARG18609|PARG28683|PARG18311|PARG18613|PARG16524|PARG06558
GO:43492	F	1.14E-03	14/665	121/14688	ATPase activity, coupled to movement of substances	PARG24774|PARG25167|PARG19811|PARG12882|PARG16346|PARG16349|PARG15139|PARG16524|PARG11466|PARG14936|PARG19917|PARG03001|PARG20158|PARG06559
GO:42626	F	1.51E-03	13/665	111/14688	ATPase activity, coupled to transmembrane movement of substances	PARG24774|PARG25167|PARG19811|PARG12882|PARG16346|PARG16349|PARG15139|PARG16524|PARG14936|PARG19917|PARG03001|PARG20158|PARG06559
GO:16820	F	1.51E-03	13/665	111/14688	hydrolase activity, acting on acid anhydrides, catalyzing transmembrane movement of substances	PARG24774|PARG25167|PARG19811|PARG12882|PARG16346|PARG16349|PARG15139|PARG16524|PARG14936|PARG19917|PARG03001|PARG20158|PARG06559
GO:33897	F	1.54E-03	4/665	12/14688	ribonuclease T2 activity	PARG27031|PARG12054|PARG12022|PARG11875
GO:16641	F	1.65E-03	5/665	20/14688	oxidoreductase activity, acting on the CH-NH2 group of donors, oxygen as acceptor	PARG03408|PARG24572|PARG24571|PARG29962|PARG29965
GO:15291	F	1.90E-03	16/665	156/14688	secondary active transmembrane transporter activity	PARG19232|PARG19341|PARG27925|PARG25248|PARG19236|PARG19510|PARG14121|PARG18772|PARG19339|PARG30247|PARG11755|PARG30250|PARG13307|PARG27786|PARG22209|PARG28668
GO:16892	F	2.15E-03	4/665	13/14688	endoribonuclease activity, producing	PARG27031|PARG12054|PARG12022|PARG11875

GO:16894	F	2.15E-03	4/665	13/14688	3'-phosphomonoesters endonuclease activity, active with either ribo- or deoxyribonucleic acids and producing 3'-phosphomonoesters	PARG27031|PARG12054|PARG12022|PARG11875
GO:01671	F	2.82E-03	3/665	7/14688	ATPase activator activity	PARG23189|PARG09744|PARG16170
GO:04568	F	2.90E-03	4/665	14/14688	chitinase activity	PARG18033|PARG16212|PARG04168|PARG18039
GO:04347	F	3.81E-03	4/665	15/14688	glucose-6-phosphate isomerase activity	PARG29582|PARG08551|PARG08271|PARG08272
GO:16853	F	4.51E-03	16/665	170/14688	isomerase activity	PARG05639|PARG16035|PARG08271|PARG08272|PARG16029|PARG12875|PARG29582|PARG08126|PARG08493|PARG05561|PARG11075|PARG08551|PARG04159|PARG05311|PARG11070|PARG01468
GO:16790	F	5.13E-03	6/665	36/14688	thiolester hydrolase activity	PARG18609|PARG28683|PARG18311|PARG18613|PARG16524|PARG06558
GO:22891	F	5.16E-03	28/665	368/14688	substrate-specific transmembrane transporter activity	PARG19232|PARG19236|PARG22165|PARG19339|PARG30247|PARG18467|PARG12321|PARG19215|PARG16887|PARG29690|PARG12328|PARG09484|PARG28668|PARG24504|PARG18131|PARG19341|PARG27925|PARG30419|PARG25248|PARG20376|PARG18772|PARG11755|PARG30250|PARG13307|PARG03990|PARG27786|PARG03992|PARG20158
GO:16787	F	5.34E-03	135/665	2431/14688	hydrolase activity	PARG00816|PARG17375|PARG12882|PARG26122|PARG03003|PARG27059|PARG03001|PARG06994|PARG28823|PARG03249|PARG00772|PARG20029|PARG20034|PARG25843|PARG00801|PARG18630|PARG16692|PARG24353|PARG25167|PARG12097|PARG18635|PARG16212|PARG18877|PARG15009|PARG12658|PARG06067|PARG26495|P

						ARG25609|PARG25608|PARG24915|PARG07716|PARG04168|PARG25606|PARG20038|PARG01851|PARG17390|PARG20158|PARG00489|PARG25536|PARG27718|PARG13394|PARG24044|PARG19811|PARG19931|PARG30048|PARG18609|PARG12108|PARG27031|PARG07988|PARG01124|PARG06807|PARG20014|PARG21586|PARG00789|PARG20252|PARG25661|PARG18613|PARG12873|PARG16953|PARG11039|PARG14936|PARG07179|PARG01073|PARG08384|PARG02162|PARG28378|PARG05039|PARG28777|PARG07979|PARG06649|PARG19351|PARG02999|PARG24300|PARG16521|PARG21271|PARG11988|PARG16524|PARG11466|PARG19917|PARG07880|PARG22767|PARG01663|PARG06559|PARG06558|PARG27704|PARG18033|PARG19240|PARG18311|PARG12054|PARG16776|PARG18039|PARG18559|PARG04083|PARG07231|PARG06545|PARG26214|PARG07237|PARG18880|PARG12022|PARG02819|PARG24480|PARG16346|PARG16349|PARG15139|PARG10554|PARG02091|PARG11124|PARG16508|PARG28683|PARG05127|PARG06337|PARG21896|PARG25615|PARG02015|PARG07701|PARG06855|PARG06217|PARG25621|PARG24774|PARG18771|PARG15022|PARG16234|PARG00907|PARG16511|PARG11875|PARG07496|PARG08983|PARG29267|PARG08980|PARG07779|PARG11193|PARG22879|PARG28853|PARG21028|PARG03216
GO:16860	F	5.90E-03	6/665	37/14688	intramolecular oxidoreductase activity	PARG29582|PARG11075|PARG08551|PARG08271|PARG08272|PARG11070
GO:10329	F	5.96E-03	2/665	3/14688	auxin efflux transmembrane transporter activity	PARG03990|PARG03992
GO:15562	F	5.96E-03	2/665	3/14688	efflux transmembrane	PARG03990|PARG03992

					transporter activity	
GO:04815	F	5.96E-03	2/665	3/14688	aspartate-tRNA ligase activity	PARG11200|PARG11199
GO:10011	F	5.96E-03	2/665	3/14688	auxin binding	PARG19918|PARG16352

Table S14. KEGG enrichment analysis of the genes from segmental duplication regions

ID	Description	GeneRatio	BgRatio	pvalue	KOID
ko04626	Plant-pathogen interaction	8/130	29/2052	0.00029	K13424/K13457/K02183/K13448/K05391/K13412/K13420/K13459
ko00940	Phenylpropanoid biosynthesis	6/130	20/2052	0.00107	K10775/K01188/K00430/K13066/K09755/K12356
ko04075	Plant hormone signal transduction	7/130	41/2052	0.01278	K14498/K14494/K14515/K14497/K13464/K14488/K14484
ko00010	Glycolysis / Gluconeogenesis	6/130	36/2052	0.02324	K00128/K01223/K01792/K01803/K01623/K01568

Table S15A. Differentially expressed genes between G1 and G2 of *P. armeniaca*

Gene ID	logFC	logCPM	PValue	FDR
PARG10659	-9.019642495	4.444687211	8.39E-91	1.32E-86
PARG23120	-5.564183296	3.95115338	7.86E-60	4.62E-56
PARG19622	-6.250793476	3.284006337	8.84E-60	4.62E-56
PARG21115	-5.013621426	9.327280172	1.86E-56	7.32E-53
PARG05759	5.784117663	5.028586391	1.27E-51	3.97E-48
PARG08087	-5.428506828	3.1084014	1.85E-50	4.85E-47
PARG23829	-5.407620563	4.316701143	6.62E-49	1.49E-45
PARG08405	4.498834488	6.650318574	8.19E-46	1.61E-42
PARG11447	-5.176747929	2.377590963	1.56E-44	2.73E-41
PARG12197	-4.243052955	5.915539186	1.98E-43	3.12E-40
PARG23119	-4.87787674	2.131156386	1.34E-42	1.91E-39
PARG06124	4.135362329	7.530410819	3.23E-41	4.22E-38
PARG18147	-8.030326863	0.460508316	1.02E-40	1.23E-37
PARG00130	-8.003096807	0.940695948	2.31E-40	2.60E-37
PARG23731	-5.253205639	1.360500005	3.89E-40	4.07E-37
PARG12245	-4.037732739	5.044094102	3.23E-39	3.17E-36
PARG03770	-4.655504269	2.478522529	1.58E-38	1.46E-35
PARG06106	-4.278790154	2.402934412	2.13E-38	1.86E-35
PARG08071	-5.831343711	1.478331532	2.61E-38	2.15E-35
PARG11948	-3.692154903	5.749786766	6.04E-36	4.74E-33
PARG03832	-3.843139963	4.874835429	7.97E-36	5.96E-33
PARG03753	-5.736369305	0.92407185	1.11E-35	7.91E-33
PARG24000	3.702876293	7.380498233	1.47E-35	1.00E-32
PARG15819	-4.338628061	1.820372431	5.20E-35	3.40E-32
PARG28258	-3.891840094	3.13079356	2.36E-34	1.48E-31
PARG20144	-4.550856783	3.852334062	2.48E-34	1.50E-31
PARG19143	-4.803890188	1.202273532	3.59E-34	2.09E-31
PARG04568	-3.890391241	3.146497246	3.89E-34	2.18E-31
PARG26415	4.997621871	4.512835605	4.78E-34	2.58E-31
PARG13459	3.556072988	8.888466238	4.92E-34	2.58E-31
PARG00648	-3.837259537	3.16497185	1.19E-33	6.00E-31
PARG12040	-3.879370264	2.730865445	2.57E-33	1.26E-30
PARG06585	-4.369514512	1.647316956	3.21E-33	1.53E-30
PARG24199	-3.724579274	3.646357861	3.50E-33	1.62E-30
PARG13320	-3.48486464	6.32575185	6.61E-33	2.96E-30
PARG06142	-3.471467524	6.616450683	1.20E-32	5.24E-30
PARG20331	-3.634223603	3.470185659	9.15E-32	3.88E-29
PARG05781	-3.746777567	2.703955267	1.37E-31	5.66E-29
PARG29491	-3.632585741	3.096619655	5.53E-31	2.23E-28
PARG11217	-3.423968734	4.586940012	6.17E-31	2.42E-28
PARG06249	-9.014255531	0.195476351	7.82E-31	2.99E-28
PARG29747	3.355707831	7.137747471	1.84E-30	6.89E-28
PARG00927	-4.046748438	1.651557613	6.53E-30	2.38E-27

PARG18426	3.275939571	9.974123612	9.33E-30	3.33E-27
PARG27269	-3.238694522	6.227942537	3.41E-29	1.19E-26
PARG30007	-6.136919581	0.457140706	5.22E-29	1.78E-26
PARG00730	-3.97387174	1.30825366	5.96E-29	1.99E-26
PARG27306	-3.719177835	2.925176102	6.71E-29	2.19E-26
PARG20114	3.665224589	5.711006545	7.04E-29	2.26E-26
PARG28453	-3.703034337	1.883439267	1.01E-28	3.18E-26
PARG19842	-4.518142877	0.921208713	3.26E-28	1.00E-25
PARG19714	-3.262853849	4.806984887	3.49E-28	1.05E-25
PARG13364	-3.925683458	1.675850814	4.77E-28	1.41E-25
PARG04129	-8.771351259	-0.300839747	6.78E-28	1.97E-25
PARG12804	3.220567918	6.167062252	1.06E-27	3.03E-25
PARG19097	-3.871465321	1.691484477	1.55E-27	4.33E-25
PARG16361	-3.094969334	7.503314518	3.25E-27	8.95E-25
PARG01859	3.292216435	7.391165706	3.57E-27	9.67E-25
PARG20811	-3.099118443	6.081149702	9.07E-27	2.41E-24
PARG08916	-3.115997696	4.945133138	2.42E-26	6.32E-24
PARG07726	3.094599894	4.237677151	5.96E-26	1.53E-23
PARG02454	-3.855595867	1.436849097	6.37E-26	1.61E-23
PARG28111	-3.009829318	5.492252802	7.07E-26	1.76E-23
PARG00964	-4.222521652	0.799517575	8.62E-26	2.12E-23
PARG19967	-3.141921927	3.59172978	1.34E-25	3.23E-23
PARG13912	-3.10638908	3.589931347	1.61E-25	3.83E-23
PARG07805	-3.462998911	1.942126416	2.17E-25	5.10E-23
PARG21735	-3.080476003	6.386504356	3.21E-25	7.42E-23
PARG26342	-4.049975748	2.447983658	3.97E-25	8.89E-23
PARG26420	-4.049975748	2.447983658	3.97E-25	8.89E-23
PARG08614	-4.121786088	0.715897557	5.25E-25	1.16E-22
PARG11119	-8.478426902	-0.217968889	1.40E-24	3.04E-22
PARG11651	-2.975433468	4.143957506	1.92E-24	4.13E-22
PARG03330	4.949354719	3.01220424	3.60E-24	7.64E-22
PARG06892	-6.067610615	0.258380239	3.72E-24	7.78E-22
PARG26618	-2.887606913	5.232021037	3.95E-24	8.14E-22
PARG02456	-3.83004646	1.086177443	3.99E-24	8.14E-22
PARG16786	4.67869641	2.617984444	4.32E-24	8.69E-22
PARG15259	-4.300891675	0.617257369	5.09E-24	1.01E-21
PARG22729	-3.033312538	3.019803489	5.43E-24	1.07E-21
PARG24981	-3.261028017	2.283021756	5.58E-24	1.08E-21
PARG27383	-4.733057149	0.033678317	7.85E-24	1.50E-21
PARG02630	-4.342914975	0.908262316	9.42E-24	1.78E-21
PARG02238	-3.649529253	1.226462066	2.84E-23	5.31E-21
PARG16227	-3.225964557	1.905852005	5.08E-23	9.38E-21
PARG28155	-2.86537072	4.264810704	5.17E-23	9.40E-21
PARG07840	5.14697521	2.745929077	5.21E-23	9.40E-21
PARG26911	-2.798567108	6.217260483	5.48E-23	9.77E-21
PARG19277	3.899110953	4.460930975	7.76E-23	1.37E-20

PARG10639	-2.774354226	6.158826819	1.20E-22	2.08E-20
PARG09356	-2.894679371	3.501397896	1.26E-22	2.17E-20
PARG23207	-2.888004477	4.734893732	1.40E-22	2.39E-20
PARG26546	-3.567837498	0.856481405	1.45E-22	2.45E-20
PARG20176	-2.818856086	7.73471866	1.64E-22	2.73E-20
PARG06847	-2.794063863	4.948629072	1.66E-22	2.73E-20
PARG11208	-2.898757105	4.356310232	1.67E-22	2.73E-20
PARG16202	-3.081321609	3.053312384	2.12E-22	3.44E-20
PARG02229	-2.807439071	4.300193414	2.38E-22	3.79E-20
PARG11947	-2.743994706	7.613069938	2.39E-22	3.79E-20
PARG27657	-3.44291166	1.479053333	2.75E-22	4.31E-20
PARG23937	3.670067319	1.720262074	3.39E-22	5.27E-20
PARG02172	-3.24557293	1.743185693	3.95E-22	6.07E-20
PARG23916	-2.946578766	2.18997684	3.99E-22	6.07E-20
PARG26827	-3.77740132	0.507402574	4.44E-22	6.70E-20
PARG28170	-3.612701343	1.733542568	5.59E-22	8.36E-20
PARG15548	-4.705879126	-0.0815608	6.09E-22	9.02E-20
PARG00800	-3.494632981	0.669079964	6.23E-22	9.14E-20
PARG27500	-3.042340764	3.190487279	6.65E-22	9.66E-20
PARG06678	-2.721986585	5.699298752	7.05E-22	1.02E-19
PARG04601	7.100473378	1.679781095	9.32E-22	1.33E-19
PARG12397	-2.699357506	7.09497188	1.01E-21	1.43E-19
PARG28062	-8.197326127	-0.654126572	1.38E-21	1.93E-19
PARG16151	-2.993201287	2.247999966	1.55E-21	2.15E-19
PARG10999	3.32082012	5.063388315	1.93E-21	2.66E-19
PARG19518	-2.977734052	2.674567484	2.23E-21	3.05E-19
PARG18352	3.816557829	5.749598588	2.30E-21	3.11E-19
PARG21074	-2.682434595	6.129831039	2.43E-21	3.26E-19
PARG02231	-2.960081493	4.136228227	3.24E-21	4.31E-19
PARG00429	3.541718151	3.796496676	3.60E-21	4.75E-19
PARG02888	-3.199369785	1.962408129	4.23E-21	5.54E-19
PARG00491	-3.485295298	1.205842962	5.51E-21	7.15E-19
PARG22096	-4.041362148	-0.045691485	5.67E-21	7.29E-19
PARG20799	-2.765667525	6.652479619	6.28E-21	8.01E-19
PARG29725	-5.759537313	-0.254662276	6.49E-21	8.22E-19
PARG06366	5.272204519	0.832753898	8.50E-21	1.07E-18
PARG23747	-3.042964574	2.397598837	8.73E-21	1.09E-18
PARG16679	-2.632903883	5.74744331	1.12E-20	1.39E-18
PARG08093	-2.645516662	6.065422994	1.59E-20	1.95E-18
PARG07944	-4.220774873	0.053577953	1.95E-20	2.37E-18
PARG05624	-2.665771737	4.588069614	3.11E-20	3.76E-18
PARG02278	3.139071885	7.232505963	3.48E-20	4.18E-18
PARG08689	2.643361342	5.608910581	3.69E-20	4.39E-18
PARG28532	-3.601309248	1.214971723	4.33E-20	5.11E-18
PARG20870	-2.62474739	3.162392886	2.28E-19	2.68E-17
PARG18722	-2.530551482	5.382941203	2.76E-19	3.21E-17

PARG29568	-2.798153506	2.058386198	3.01E-19	3.47E-17
PARG24857	3.618726395	4.809427209	3.39E-19	3.88E-17
PARG11661	2.869029064	3.86229958	3.43E-19	3.90E-17
PARG24852	-2.498526032	7.329719991	3.98E-19	4.49E-17
PARG03266	2.537032973	7.141177913	4.10E-19	4.58E-17
PARG12205	-2.507493397	5.91854037	4.11E-19	4.58E-17
PARG06887	2.895016128	2.346369466	5.37E-19	5.94E-17
PARG03178	-2.870227961	2.790522913	5.92E-19	6.50E-17
PARG26786	-2.499663171	5.185679998	6.17E-19	6.72E-17
PARG25739	-2.520284742	5.688008721	6.83E-19	7.40E-17
PARG00025	-3.042336926	0.796167418	6.96E-19	7.49E-17
PARG16312	-3.028316345	1.381715729	7.53E-19	8.04E-17
PARG10145	2.73214669	8.59979686	7.74E-19	8.21E-17
PARG27385	2.553526122	6.812555639	7.81E-19	8.23E-17
PARG18476	-3.461948898	0.535809997	8.10E-19	8.48E-17
PARG00266	-2.676368081	2.572072674	1.01E-18	1.05E-16
PARG10150	-2.902804988	5.220183431	1.04E-18	1.08E-16
PARG14598	-2.562702422	4.319342742	1.37E-18	1.41E-16
PARG12295	-2.598505227	3.551923873	1.48E-18	1.51E-16
PARG23224	-2.447471148	9.097944029	1.55E-18	1.56E-16
PARG05721	-2.674415539	3.501046141	1.62E-18	1.63E-16
PARG05964	-3.234784599	0.613966333	1.73E-18	1.73E-16
PARG08522	-2.511632093	4.336083148	1.85E-18	1.84E-16
PARG17968	-2.905794532	1.900462821	2.02E-18	2.00E-16
PARG25155	-4.616132093	0.772143672	2.42E-18	2.35E-16
PARG11695	-4.60589007	0.646779159	2.42E-18	2.35E-16
PARG15965	-2.880112274	1.427743919	2.43E-18	2.35E-16
PARG21211	-2.450961498	5.817017394	2.46E-18	2.37E-16
PARG11341	-2.553029727	6.390015649	2.66E-18	2.55E-16
PARG06006	-2.441735743	6.004376269	2.76E-18	2.62E-16
PARG21286	2.471372391	4.771536959	5.34E-18	5.05E-16
PARG00930	-2.45889522	4.989474632	5.68E-18	5.33E-16
PARG00990	-2.875314667	1.402355633	5.72E-18	5.34E-16
PARG08122	-2.986197038	1.840639881	5.91E-18	5.49E-16
PARG14430	3.158662222	3.023163977	6.19E-18	5.72E-16
PARG19761	-2.620811382	2.22660345	7.46E-18	6.85E-16
PARG09623	-3.467393711	0.641007178	7.69E-18	7.02E-16
PARG21270	2.648305201	3.265395339	1.01E-17	9.19E-16
PARG10303	-2.551244257	2.819163078	1.20E-17	1.08E-15
PARG13361	-2.494466704	3.533959587	1.22E-17	1.10E-15
PARG29277	-3.145261295	0.752614223	1.58E-17	1.41E-15
PARG08314	-2.371739678	7.003564976	1.61E-17	1.43E-15
PARG30259	-2.404184355	3.942188352	1.63E-17	1.44E-15
PARG15564	-2.472802163	3.51024955	1.68E-17	1.48E-15
PARG02095	2.386206977	5.315126319	1.75E-17	1.52E-15
PARG01099	-3.001272062	1.030803365	1.82E-17	1.58E-15

PARG23946	-3.604979892	0.07587826	1.86E-17	1.60E-15
PARG21065	-2.488648861	3.152606628	1.99E-17	1.71E-15
PARG27282	2.397850208	5.894254307	2.13E-17	1.82E-15
PARG10708	-2.396693827	4.356117311	2.22E-17	1.88E-15
PARG10987	-3.012587595	1.074831529	2.39E-17	2.02E-15
PARG03641	2.564173703	3.031882342	2.54E-17	2.13E-15
PARG08621	-2.44065314	3.3887544	2.91E-17	2.43E-15
PARG02255	-3.110693479	0.61282364	3.69E-17	3.07E-15
PARG18144	-2.465939126	3.663549985	3.76E-17	3.11E-15
PARG00201	2.356618091	7.869194265	3.81E-17	3.13E-15
PARG19016	-2.908552341	1.138550457	4.06E-17	3.32E-15
PARG25383	-2.333322667	8.138976861	4.13E-17	3.36E-15
PARG14187	-6.027853788	0.239558377	4.42E-17	3.58E-15
PARG00172	-2.334280058	6.376466441	5.09E-17	4.09E-15
PARG16072	-3.04550743	0.695291381	5.56E-17	4.44E-15
PARG19431	2.857467556	3.377581544	5.58E-17	4.44E-15
PARG02637	3.774013052	1.525137862	5.84E-17	4.63E-15
PARG10540	2.325850094	6.679984331	6.89E-17	5.43E-15
PARG20063	-2.486764391	3.263760283	7.58E-17	5.95E-15
PARG25102	-2.422808737	3.983112605	7.65E-17	5.98E-15
PARG03653	-2.596646328	1.740450772	9.12E-17	7.08E-15
PARG29945	2.402605097	3.422043691	9.46E-17	7.29E-15
PARG21339	-3.925059555	-0.34297414	9.47E-17	7.29E-15
PARG13822	-2.941947865	0.252079339	9.63E-17	7.37E-15
PARG07598	2.341547067	7.829101663	1.03E-16	7.84E-15
PARG27025	-2.66084137	0.932235585	1.15E-16	8.74E-15
PARG12132	-4.650812505	-0.422849462	1.18E-16	8.91E-15
PARG13940	-3.949938661	-0.211380422	1.20E-16	9.05E-15
PARG07079	-2.67300023	1.473381232	1.26E-16	9.38E-15
PARG27948	-3.224940564	0.778125817	1.60E-16	1.19E-14
PARG17555	-3.13320052	0.273308806	1.61E-16	1.19E-14
PARG19110	-3.140769387	0.657404458	1.82E-16	1.34E-14
PARG11900	2.340048387	4.564264168	2.49E-16	1.82E-14
PARG20667	-2.351046477	4.28584534	2.63E-16	1.92E-14
PARG14188	-3.54169135	0.359948317	3.08E-16	2.24E-14
PARG16567	-3.161785943	0.440165541	3.29E-16	2.38E-14
PARG13116	-2.256267181	7.648694314	3.59E-16	2.58E-14
PARG13683	-2.391967423	3.619196697	3.73E-16	2.67E-14
PARG03307	-2.472425624	2.749647101	4.38E-16	3.13E-14
PARG13044	-2.567085034	2.019653043	5.29E-16	3.76E-14
PARG25121	2.316170693	3.372626466	5.42E-16	3.83E-14
PARG29156	-2.334498145	3.780226703	5.78E-16	4.07E-14
PARG05825	-2.292080111	3.741991444	6.79E-16	4.76E-14
PARG09088	-2.2311607	6.759129309	7.29E-16	5.09E-14
PARG24285	2.235089716	8.468259564	7.89E-16	5.48E-14
PARG27582	2.368934838	10.12594402	1.09E-15	7.53E-14

PARG27896	2.410770205	4.091614028	1.14E-15	7.84E-14
PARG19010	-2.42538995	3.202424718	1.20E-15	8.20E-14
PARG00916	3.489575383	1.460193436	1.32E-15	9.00E-14
PARG22832	-2.227845456	4.920380521	1.32E-15	9.00E-14
PARG06353	-2.520533186	1.809902458	1.33E-15	9.01E-14
PARG08253	-3.67499825	-0.398524064	1.36E-15	9.15E-14
PARG28007	-2.961839932	0.923180118	1.44E-15	9.63E-14
PARG09652	-2.260270209	3.723885058	1.47E-15	9.79E-14
PARG00542	-3.286434198	0.697389236	1.48E-15	9.82E-14
PARG11056	2.223389965	7.186768366	1.50E-15	9.95E-14
PARG03197	-2.488598422	2.424180539	1.52E-15	1.00E-13
PARG08190	-2.32026297	3.132647824	1.64E-15	1.07E-13
PARG16256	-2.497698482	1.984482322	1.64E-15	1.07E-13
PARG24752	-2.482541825	1.835484491	1.86E-15	1.21E-13
PARG12451	-2.191427477	9.229520181	1.93E-15	1.25E-13
PARG10505	-2.686750924	1.293592	1.96E-15	1.27E-13
PARG11592	-4.783715164	-0.704673242	2.07E-15	1.33E-13
PARG13588	3.044720097	0.275658179	2.16E-15	1.39E-13
PARG06059	-2.357228324	2.232203171	2.32E-15	1.48E-13
PARG29228	2.31229359	3.249996202	2.39E-15	1.52E-13
PARG11118	-3.873852178	0.45759092	2.52E-15	1.60E-13
PARG16049	2.927110386	3.438220242	2.97E-15	1.87E-13
PARG27772	-2.180887477	9.091796253	3.37E-15	2.11E-13
PARG29956	-2.181849388	6.196001655	3.41E-15	2.13E-13
PARG27270	-2.319857911	2.633617999	3.50E-15	2.18E-13
PARG12998	3.343270974	0.858683393	3.84E-15	2.38E-13
PARG02441	-2.182741904	5.529634432	3.97E-15	2.45E-13
PARG00249	-2.206604964	4.609023205	4.05E-15	2.49E-13
PARG26646	-2.209940739	3.938474266	4.79E-15	2.94E-13
PARG02829	-3.65755375	0.230124545	5.13E-15	3.13E-13
PARG12724	-3.464810482	0.168452934	5.44E-15	3.31E-13
PARG24508	-2.171915712	5.521564704	5.53E-15	3.35E-13
PARG27360	-2.27747086	3.106201862	5.98E-15	3.61E-13
PARG12283	-2.173634982	4.306508795	6.28E-15	3.78E-13
PARG24621	-2.661825106	1.151572373	6.90E-15	4.14E-13
PARG24732	3.92168089	1.451690958	7.16E-15	4.27E-13
PARG10011	2.386037934	6.834896713	8.20E-15	4.88E-13
PARG05884	-3.507002093	-0.321910938	8.36E-15	4.95E-13
PARG07303	-2.400023177	4.528784792	9.44E-15	5.57E-13
PARG10608	-2.532016076	3.063434659	9.94E-15	5.85E-13
PARG06164	-2.759639403	0.801453682	1.00E-14	5.86E-13
PARG19897	-2.203502552	3.795065825	1.07E-14	6.25E-13
PARG23241	-2.57251195	2.686559051	1.08E-14	6.26E-13
PARG03702	2.364754485	3.807377164	1.10E-14	6.39E-13
PARG23988	-2.540030177	1.212852807	1.11E-14	6.43E-13
PARG00106	-2.198173279	4.763651594	1.45E-14	8.32E-13

PARG18167	-2.140539659	4.427306026	1.47E-14	8.44E-13
PARG13945	-2.624129679	1.282256297	1.53E-14	8.75E-13
PARG11196	-2.73741054	2.050371818	1.75E-14	9.96E-13
PARG20475	-2.219667063	3.049569601	1.79E-14	1.01E-12
PARG12725	-3.407350711	-0.478102935	1.79E-14	1.01E-12
PARG02834	2.165848813	4.552809329	1.83E-14	1.03E-12
PARG16638	-2.276515723	3.181628697	1.90E-14	1.06E-12
PARG02078	2.118652091	5.418663104	2.09E-14	1.17E-12
PARG26450	-2.545218591	1.477180706	2.21E-14	1.23E-12
PARG06509	-5.047939176	-0.447743063	2.52E-14	1.40E-12
PARG11107	-2.105968281	7.733607307	2.53E-14	1.40E-12
PARG00819	-2.427946578	2.164834303	2.71E-14	1.49E-12
PARG01970	-2.386154308	1.806749358	2.89E-14	1.58E-12
PARG24919	-2.434538726	1.607493154	2.95E-14	1.61E-12
PARG12115	-2.338569374	2.087615239	3.16E-14	1.72E-12
PARG18194	-2.170200428	3.209859983	3.31E-14	1.80E-12
PARG20563	-2.792822055	0.522960458	3.63E-14	1.97E-12
PARG01858	2.29293326	4.473824507	3.75E-14	2.02E-12
PARG26687	-2.152821533	3.540503561	3.76E-14	2.02E-12
PARG29530	-2.429394266	2.286131583	3.83E-14	2.05E-12
PARG19293	-3.9886479	-0.178024401	3.98E-14	2.12E-12
PARG07377	-2.119027352	4.490976442	4.17E-14	2.22E-12
PARG29823	-2.420252827	2.453547577	4.32E-14	2.29E-12
PARG09192	-2.11081704	5.016134317	4.66E-14	2.47E-12
PARG00045	-2.09323584	6.00730287	4.82E-14	2.54E-12
PARG26573	-2.446408363	1.546250236	5.01E-14	2.63E-12
PARG25363	-2.19776053	2.735686599	5.09E-14	2.66E-12
PARG06172	-2.602962946	1.693839944	5.38E-14	2.81E-12
PARG19294	2.70374829	1.344456003	5.73E-14	2.98E-12
PARG06885	2.355580559	2.242363068	5.78E-14	3.00E-12
PARG29528	-2.058865203	8.705177766	6.68E-14	3.44E-12
PARG19832	-2.76934828	0.340466067	6.69E-14	3.44E-12
PARG06005	-2.07064417	5.881364148	6.94E-14	3.56E-12
PARG12355	2.288518528	2.034800469	7.05E-14	3.61E-12
PARG21818	2.520649911	4.782586591	7.13E-14	3.63E-12
PARG25154	-2.136759233	2.94063225	7.67E-14	3.90E-12
PARG23754	-2.326603015	1.788486464	7.91E-14	4.01E-12
PARG20401	-2.085498296	5.047108376	7.97E-14	4.02E-12
PARG06549	2.198316544	3.671568919	8.16E-14	4.11E-12
PARG19181	2.084326905	5.679368849	8.46E-14	4.24E-12
PARG29258	2.127452544	3.569489576	8.47E-14	4.24E-12
PARG06891	-2.115841495	5.594203155	8.86E-14	4.41E-12
PARG03974	-2.068017635	5.248924146	9.17E-14	4.55E-12
PARG16825	2.25540537	4.404977238	9.19E-14	4.55E-12
PARG00167	-2.474706596	1.265250396	9.28E-14	4.58E-12
PARG23157	3.416009617	-0.09276775	9.40E-14	4.63E-12

PARG07858	-2.974031313	0.264850635	9.49E-14	4.66E-12
PARG18546	-2.616518946	1.015939317	9.77E-14	4.78E-12
PARG24615	-2.122835598	3.930342804	9.90E-14	4.83E-12
PARG03699	2.21574763	3.054872378	1.10E-13	5.35E-12
PARG16535	-2.273479298	3.377114092	1.15E-13	5.55E-12
PARG04133	-2.460950972	1.140306882	1.22E-13	5.91E-12
PARG02700	-2.074134581	4.863477351	1.25E-13	6.01E-12
PARG19170	2.160740738	2.436974899	1.25E-13	6.02E-12
PARG09816	-2.226346429	2.625871203	1.31E-13	6.27E-12
PARG20885	-2.146376701	2.940747719	1.41E-13	6.71E-12
PARG06944	-2.607384179	1.019598537	1.54E-13	7.34E-12
PARG12839	-2.060223134	4.443764948	1.71E-13	8.09E-12
PARG15407	2.152030264	5.838425831	1.73E-13	8.20E-12
PARG08510	-3.098129325	-0.111801236	1.88E-13	8.85E-12
PARG00361	-2.113978355	8.2241675	1.94E-13	9.11E-12
PARG05099	-2.166251746	2.388416522	1.97E-13	9.24E-12
PARG07518	2.030200344	5.235779098	2.15E-13	1.00E-11
PARG01088	-2.256292204	2.038842881	2.35E-13	1.09E-11
PARG19681	2.197584633	4.548205559	2.35E-13	1.09E-11
PARG10669	-2.020462669	6.434850034	2.55E-13	1.18E-11
PARG26429	-2.140513636	2.862103534	2.65E-13	1.22E-11
PARG21631	-2.086197748	3.478053336	2.66E-13	1.22E-11
PARG23732	-2.100695168	2.719157053	2.69E-13	1.23E-11
PARG04197	-2.001509455	8.127885135	2.84E-13	1.30E-11
PARG12055	-3.34437585	-0.238122796	2.86E-13	1.30E-11
PARG07278	-2.005960585	5.900035483	3.32E-13	1.51E-11
PARG24312	-2.934335978	0.027043272	3.45E-13	1.57E-11
PARG15708	-2.099338523	6.270926396	3.68E-13	1.66E-11
PARG28168	-2.434611322	1.349600709	3.76E-13	1.70E-11
PARG26913	-2.073815722	5.129740196	3.80E-13	1.71E-11
PARG03375	-2.104088337	3.131087379	4.00E-13	1.79E-11
PARG10681	-1.99432558	6.684421644	4.16E-13	1.86E-11
PARG10196	-2.134959813	1.798076347	4.23E-13	1.89E-11
PARG01345	-2.764237701	0.537433168	4.27E-13	1.90E-11
PARG13939	-2.030350297	4.242447449	4.38E-13	1.94E-11
PARG05897	-2.987294584	0.94178682	4.44E-13	1.96E-11
PARG00957	-1.992911301	6.916034472	4.46E-13	1.97E-11
PARG21106	-2.408716241	0.976690604	4.75E-13	2.09E-11
PARG00299	-1.994049113	5.716670837	4.86E-13	2.13E-11
PARG30381	-2.233255323	1.565286054	5.21E-13	2.28E-11
PARG19138	-2.062168203	3.043070324	5.31E-13	2.32E-11
PARG29428	-2.26215821	1.727854715	5.69E-13	2.47E-11
PARG12547	-2.113275938	2.911894606	5.75E-13	2.49E-11
PARG29350	1.971515289	11.01680965	5.85E-13	2.53E-11
PARG14083	-2.199304641	2.109998451	6.06E-13	2.62E-11
PARG05333	-2.481440546	2.658891624	6.21E-13	2.67E-11

PARG19331	-4.863964263	-0.813443373	6.46E-13	2.77E-11
PARG18358	-1.967637327	6.779770584	7.19E-13	3.07E-11
PARG11595	-2.041653684	3.557770031	7.25E-13	3.09E-11
PARG07066	-3.71193106	-0.367194005	7.67E-13	3.26E-11
PARG03655	3.167253364	2.842824179	8.21E-13	3.48E-11
PARG14514	-2.24139244	2.823205434	8.28E-13	3.50E-11
PARG11918	-3.168918614	-0.384525977	8.36E-13	3.53E-11
PARG06465	-2.594141637	0.225381463	8.59E-13	3.61E-11
PARG26859	-2.054133436	3.352405666	9.31E-13	3.91E-11
PARG29451	-2.01484689	4.208033329	1.08E-12	4.51E-11
PARG04493	2.209395248	3.931140719	1.09E-12	4.55E-11
PARG23798	-2.05246958	3.033846216	1.15E-12	4.78E-11
PARG05037	2.544933655	4.329543725	1.17E-12	4.86E-11
PARG01972	-2.118509776	2.967130758	1.23E-12	5.11E-11
PARG10953	-2.042690716	3.732329695	1.24E-12	5.13E-11
PARG21176	2.192947354	2.221887304	1.26E-12	5.19E-11
PARG06683	-1.958552942	5.145138417	1.31E-12	5.40E-11
PARG24668	-1.952673352	5.129292605	1.33E-12	5.43E-11
PARG24706	-2.904501375	0.171417145	1.37E-12	5.59E-11
PARG19553	-2.002941163	3.781767506	1.44E-12	5.87E-11
PARG10062	-3.808413658	1.47991312	1.45E-12	5.88E-11
PARG07356	-3.467408962	0.074715757	1.54E-12	6.25E-11
PARG15534	-1.9454029	5.450859691	1.55E-12	6.26E-11
PARG19707	-2.044697459	3.186503748	1.58E-12	6.39E-11
PARG16366	1.939512183	7.499358661	1.61E-12	6.49E-11
PARG19393	-1.979683632	4.149109822	1.62E-12	6.49E-11
PARG07714	2.250036812	4.464796566	1.63E-12	6.54E-11
PARG26040	-2.715309826	0.207566226	1.70E-12	6.80E-11
PARG19406	3.458806073	-0.322191197	1.71E-12	6.80E-11
PARG30223	2.064980943	3.85111114	1.71E-12	6.80E-11
PARG19023	2.113693587	8.981044124	1.73E-12	6.87E-11
PARG13797	7.086149399	-0.5697773	1.76E-12	6.96E-11
PARG06160	-2.553505279	0.764392671	1.80E-12	7.12E-11
PARG01043	2.114823452	9.265096106	1.97E-12	7.74E-11
PARG21800	-2.047411884	3.397095576	1.98E-12	7.75E-11
PARG18971	1.945815578	5.800736283	2.17E-12	8.51E-11
PARG05881	2.013045693	5.458240203	2.25E-12	8.80E-11
PARG24109	-2.01478322	3.676798512	2.26E-12	8.80E-11
PARG19880	-1.927962088	5.352558103	2.48E-12	9.64E-11
PARG29273	-2.124597368	2.007925713	2.53E-12	9.82E-11
PARG24609	-2.045047485	4.934882515	2.78E-12	1.07E-10
PARG11227	-2.029285373	4.304295388	2.84E-12	1.09E-10
PARG03746	-2.752766813	0.17143826	2.84E-12	1.09E-10
PARG30101	1.90107127	9.897831997	3.38E-12	1.30E-10
PARG12930	1.89928348	11.59509161	3.47E-12	1.33E-10
PARG27774	-2.122425523	2.196413383	3.50E-12	1.34E-10

PARG06316	-1.996892987	3.138170995	3.60E-12	1.37E-10
PARG18457	-2.040904236	3.185211603	4.09E-12	1.56E-10
PARG00131	-7.055316461	-1.294389643	4.22E-12	1.60E-10
PARG00367	-4.758169898	-0.985177271	4.22E-12	1.60E-10
PARG20941	1.936806321	6.156639028	4.29E-12	1.62E-10
PARG11224	-1.91390155	4.75480434	4.31E-12	1.62E-10
PARG06058	-2.467671545	0.351579197	4.31E-12	1.62E-10
PARG16388	-3.409420308	-0.658984154	4.66E-12	1.75E-10
PARG08146	-1.887587873	9.014529711	4.70E-12	1.76E-10
PARG23198	-2.32226767	0.454152234	5.01E-12	1.87E-10
PARG09014	-2.392485206	0.874487212	5.24E-12	1.95E-10
PARG20977	-2.435403166	0.512489231	5.53E-12	2.05E-10
PARG11561	-2.896226945	0.268735848	5.81E-12	2.15E-10
PARG20315	-2.085359288	1.99460557	5.84E-12	2.16E-10
PARG24362	-1.994716794	3.092637013	5.91E-12	2.18E-10
PARG22658	-1.879218464	8.056628324	6.02E-12	2.21E-10
PARG11870	-1.941923669	4.144520596	6.13E-12	2.25E-10
PARG14163	-1.878817641	7.360377101	6.22E-12	2.28E-10
PARG23027	-2.008755	2.687741582	6.30E-12	2.30E-10
PARG21287	1.872601971	10.33016875	6.68E-12	2.43E-10
PARG10864	-1.940645203	4.857672363	7.39E-12	2.69E-10
PARG20806	-2.13459822	4.610467416	7.49E-12	2.71E-10
PARG07776	-1.929404924	3.703688967	8.13E-12	2.94E-10
PARG16607	-3.575009698	2.583306097	8.32E-12	3.00E-10
PARG27828	1.882725104	6.28255635	8.41E-12	3.03E-10
PARG02815	-1.985055549	4.020311598	8.68E-12	3.12E-10
PARG21093	1.901624186	6.157327745	8.94E-12	3.20E-10
PARG12600	1.866016331	7.498016262	9.11E-12	3.25E-10
PARG20343	-1.878571924	6.260654999	9.11E-12	3.25E-10
PARG29281	-1.862137992	9.441587798	9.28E-12	3.30E-10
PARG00829	-5.353788282	-1.04074258	9.53E-12	3.38E-10
PARG04598	1.955627283	5.634842752	9.87E-12	3.50E-10
PARG18508	1.909472481	3.625695873	9.89E-12	3.50E-10
PARG00655	-1.892067379	4.549205146	1.01E-11	3.55E-10
PARG06046	2.121873951	4.649726503	1.08E-11	3.82E-10
PARG11540	-2.355918936	0.99502679	1.09E-11	3.84E-10
PARG02428	2.035232234	3.129295159	1.11E-11	3.90E-10
PARG07260	-1.925513096	3.103268069	1.29E-11	4.50E-10
PARG19466	-1.862912097	5.382934495	1.32E-11	4.61E-10
PARG16308	-2.18196942	1.793698468	1.36E-11	4.74E-10
PARG23955	-2.412266984	0.46316233	1.40E-11	4.87E-10
PARG17110	1.840506422	11.1684675	1.43E-11	4.96E-10
PARG16859	-1.937721962	3.30208086	1.44E-11	4.97E-10
PARG06673	2.939665096	0.668852055	1.44E-11	4.98E-10
PARG27587	-1.854540975	5.499271835	1.46E-11	5.04E-10
PARG13228	-1.84964767	5.092055756	1.63E-11	5.61E-10

PARG27567	-2.09632132	1.604277315	1.66E-11	5.69E-10
PARG11903	-2.136866102	1.499370259	1.78E-11	6.08E-10
PARG23543	2.265147657	2.629719502	1.79E-11	6.11E-10
PARG06701	-2.910211093	-0.016680256	1.80E-11	6.12E-10
PARG09622	-1.845715038	5.076288106	1.87E-11	6.37E-10
PARG27372	-1.882846968	3.41196716	2.19E-11	7.42E-10
PARG02071	-1.898721682	3.824031588	2.27E-11	7.70E-10
PARG22526	-3.413767706	-0.26762416	2.33E-11	7.88E-10
PARG30033	-1.942992845	2.952789568	2.40E-11	8.08E-10
PARG06349	-3.655195903	-0.532269919	2.45E-11	8.23E-10
PARG16245	2.275321391	2.9594722	2.48E-11	8.33E-10
PARG27261	-2.503866341	0.184486645	2.57E-11	8.59E-10
PARG29969	-1.892233228	3.091276449	2.71E-11	9.06E-10
PARG29555	1.824475475	7.698583447	2.74E-11	9.12E-10
PARG12210	-1.846957411	6.317377973	2.78E-11	9.23E-10
PARG29229	-3.511494795	-0.187611108	2.83E-11	9.38E-10
PARG06612	-1.859075761	3.949629031	3.06E-11	1.01E-09
PARG24251	-1.881391008	5.761858551	3.22E-11	1.07E-09
PARG02315	-2.428104517	0.58181021	3.28E-11	1.08E-09
PARG29421	-1.847653817	4.109783592	3.29E-11	1.08E-09
PARG17395	-1.842442056	4.696038336	3.41E-11	1.12E-09
PARG15814	1.96661766	3.043896426	3.42E-11	1.12E-09
PARG12938	1.884034886	4.055763846	3.46E-11	1.13E-09
PARG25577	1.999016277	3.593685468	3.47E-11	1.13E-09
PARG04921	-1.997967889	2.383309377	3.57E-11	1.16E-09
PARG20647	-1.816099749	5.514356508	3.57E-11	1.16E-09
PARG11813	-2.023552528	2.002974281	3.87E-11	1.25E-09
PARG08406	6.861859181	0.806603805	3.97E-11	1.29E-09
PARG10314	-2.398220763	0.722177283	4.23E-11	1.37E-09
PARG01881	1.80932726	8.150363915	4.26E-11	1.37E-09
PARG25067	-3.976683742	-0.64845761	4.40E-11	1.42E-09
PARG29766	-1.882160068	2.627835439	4.46E-11	1.43E-09
PARG13158	-1.874761141	3.633369103	4.47E-11	1.43E-09
PARG01959	-2.21342601	0.990733872	4.62E-11	1.48E-09
PARG13864	-2.132594452	1.222640915	4.69E-11	1.50E-09
PARG29379	-1.915595057	1.781447427	4.75E-11	1.51E-09
PARG27461	-2.905041385	-0.145616772	4.83E-11	1.54E-09
PARG21077	-1.992812598	2.734695346	4.96E-11	1.57E-09
PARG29406	-1.902711389	2.802023999	5.24E-11	1.66E-09
PARG01639	-1.849715745	3.497476288	5.31E-11	1.68E-09
PARG06814	-1.823268725	4.716202415	5.32E-11	1.68E-09
PARG12159	-1.915348232	2.866468169	5.37E-11	1.69E-09
PARG18245	1.983042198	2.535977788	5.39E-11	1.69E-09
PARG06846	-1.870286683	3.571271745	5.41E-11	1.69E-09
PARG14268	-1.788923281	6.452431416	5.52E-11	1.73E-09
PARG24219	-1.952777424	2.219418763	5.53E-11	1.73E-09

PARG15135	-1.797808263	5.751032709	5.64E-11	1.76E-09
PARG23340	-2.065010269	1.240921979	5.67E-11	1.76E-09
PARG10960	-1.788626676	7.552704679	5.68E-11	1.76E-09
PARG06093	-1.788586422	7.023772119	5.69E-11	1.76E-09
PARG03411	-2.331766547	2.19312129	5.71E-11	1.76E-09
PARG16784	3.343617213	4.050273438	6.01E-11	1.86E-09
PARG21566	-2.994351429	-0.209095234	6.29E-11	1.93E-09
PARG07504	-1.858487828	3.00429027	6.30E-11	1.93E-09
PARG16509	-2.148926348	4.35752533	6.31E-11	1.93E-09
PARG20506	-1.848718386	3.613739007	6.78E-11	2.07E-09
PARG08600	-1.890436143	2.467750273	7.68E-11	2.34E-09
PARG00267	1.781107198	6.2838292	8.06E-11	2.46E-09
PARG21050	-1.781422755	6.05302271	8.70E-11	2.65E-09
PARG19363	-3.911742326	2.335803076	8.98E-11	2.72E-09
PARG06455	1.885825857	3.944129086	8.99E-11	2.72E-09
PARG25221	-1.774401787	5.780459614	9.03E-11	2.73E-09
PARG21141	-1.819332804	3.670319917	9.06E-11	2.74E-09
PARG00564	-2.018846122	1.131410867	9.50E-11	2.86E-09
PARG05189	-1.823274829	2.885470894	9.98E-11	3.00E-09
PARG27291	-1.762176355	6.258921555	1.06E-10	3.17E-09
PARG09155	-3.724390637	0.091786626	1.09E-10	3.27E-09
PARG11804	-2.379513769	0.420444335	1.11E-10	3.32E-09
PARG29721	1.76266725	7.080864987	1.21E-10	3.60E-09
PARG09452	-1.788768515	4.727342759	1.21E-10	3.61E-09
PARG13005	-1.805957063	4.628560441	1.25E-10	3.72E-09
PARG24540	-1.866889583	6.228670193	1.31E-10	3.87E-09
PARG04359	-2.034545881	1.550430624	1.32E-10	3.91E-09
PARG07304	-1.795368669	4.664838114	1.37E-10	4.05E-09
PARG00475	-1.809584956	4.585214582	1.38E-10	4.08E-09
PARG03744	-2.444804297	0.523113807	1.39E-10	4.10E-09
PARG17985	-1.970131429	2.376568635	1.40E-10	4.13E-09
PARG04340	-1.885569618	2.495194694	1.47E-10	4.30E-09
PARG07523	-1.833804114	3.24490587	1.48E-10	4.33E-09
PARG26402	-2.063707811	1.106144755	1.50E-10	4.38E-09
PARG03834	-1.959486359	1.923861988	1.54E-10	4.50E-09
PARG18833	1.759077576	5.735074183	1.55E-10	4.51E-09
PARG10998	2.860399095	3.295450132	1.56E-10	4.54E-09
PARG06905	-1.853419083	2.825836826	1.65E-10	4.80E-09
PARG24278	-1.799158688	3.748836378	1.68E-10	4.86E-09
PARG19361	-1.932279227	2.342442477	1.80E-10	5.20E-09
PARG29742	2.908614991	-0.025847452	1.81E-10	5.21E-09
PARG26517	1.764884715	5.621426744	1.91E-10	5.50E-09
PARG12452	-2.987176337	0.985908126	1.91E-10	5.50E-09
PARG24765	-2.123246513	2.467046391	2.03E-10	5.82E-09
PARG01195	1.810386439	3.812353391	2.03E-10	5.82E-09
PARG19216	-1.734421227	5.373863946	2.16E-10	6.17E-09

PARG10667	1.723315995	8.972258474	2.25E-10	6.42E-09
PARG06204	-1.776113175	4.74858026	2.36E-10	6.71E-09
PARG01177	-1.843420397	2.42859424	2.40E-10	6.81E-09
PARG23158	-2.545946307	0.431528937	2.47E-10	7.01E-09
PARG09459	-1.888150558	2.384577196	2.54E-10	7.20E-09
PARG06433	-2.350673765	0.517009344	2.57E-10	7.27E-09
PARG19014	-1.807367504	3.881608208	2.60E-10	7.34E-09
PARG25052	-1.775574065	3.484035877	2.61E-10	7.34E-09
PARG24409	-2.43242228	-0.403437354	2.61E-10	7.34E-09
PARG24185	-1.791018969	7.651244289	2.64E-10	7.42E-09
PARG13392	-3.864895675	-0.899814154	2.65E-10	7.44E-09
PARG22178	1.763292433	4.111717302	2.68E-10	7.51E-09
PARG14943	-2.206203581	0.831215072	2.70E-10	7.53E-09
PARG06980	1.716330738	8.142704598	2.73E-10	7.62E-09
PARG14659	-1.878674544	2.120109942	2.85E-10	7.93E-09
PARG22865	-1.783924592	4.416496096	2.88E-10	8.01E-09
PARG14706	-1.892878167	2.000010069	2.96E-10	8.21E-09
PARG22515	-2.093425471	1.35190091	2.97E-10	8.21E-09
PARG15799	1.834345273	3.336255862	3.07E-10	8.47E-09
PARG12266	-1.875627756	3.396156645	3.14E-10	8.67E-09
PARG11553	-1.870720954	3.663098965	3.16E-10	8.71E-09
PARG03479	1.739083609	6.853821275	3.20E-10	8.80E-09
PARG20800	-2.786760148	2.714263175	3.25E-10	8.91E-09
PARG10069	1.727195107	4.880705781	3.33E-10	9.12E-09
PARG00107	-1.834539239	2.775589162	3.33E-10	9.12E-09
PARG07112	1.831197179	3.380681025	3.36E-10	9.16E-09
PARG01159	-1.706160899	7.692360326	3.40E-10	9.28E-09
PARG05475	-3.187851433	-0.26488477	3.49E-10	9.49E-09
PARG21320	-1.709766169	6.531463087	3.54E-10	9.63E-09
PARG08529	-2.324378007	0.534921212	3.57E-10	9.69E-09
PARG27675	-1.741595674	4.60733211	3.62E-10	9.81E-09
PARG13589	-2.574410133	0.095608972	3.66E-10	9.89E-09
PARG28512	-1.716017218	5.564219186	3.73E-10	1.01E-08
PARG12895	-2.802839672	0.323713678	3.76E-10	1.01E-08
PARG04811	-2.249730599	1.174416748	3.83E-10	1.03E-08
PARG17748	-2.943361007	-0.274431054	4.02E-10	1.08E-08
PARG19533	1.80257658	7.017536159	4.08E-10	1.09E-08
PARG02333	-1.810903104	4.230579456	4.26E-10	1.14E-08
PARG19449	1.712162007	5.660574018	4.35E-10	1.16E-08
PARG02036	-1.727175068	4.171261163	4.43E-10	1.18E-08
PARG02930	-1.837812793	2.502240902	4.59E-10	1.22E-08
PARG26561	-1.878379788	1.951687293	4.60E-10	1.22E-08
PARG00777	-1.706698674	4.664086585	4.61E-10	1.22E-08
PARG13225	-1.750469613	4.983713218	4.70E-10	1.24E-08
PARG12116	-1.922205555	2.148006807	4.85E-10	1.28E-08
PARG06789	-2.455459846	-0.096835957	5.12E-10	1.35E-08

PARG08244	-1.74626022	5.512956097	5.19E-10	1.37E-08
PARG06708	2.204111951	2.783588836	5.20E-10	1.37E-08
PARG20381	-1.735560368	3.905760245	5.30E-10	1.39E-08
PARG12192	-2.394678749	0.12941599	5.48E-10	1.44E-08
PARG21289	-1.698055952	4.965576133	5.51E-10	1.44E-08
PARG02235	2.619888131	0.937267227	5.57E-10	1.45E-08
PARG23229	-2.617869796	-0.017648678	5.57E-10	1.45E-08
PARG19940	1.79983413	5.462216445	5.76E-10	1.50E-08
PARG29460	-2.722123073	-0.148952805	5.81E-10	1.51E-08
PARG13932	-1.742913638	4.922834685	5.91E-10	1.53E-08
PARG22600	-1.765235	3.142198831	5.91E-10	1.53E-08
PARG18751	1.691949257	5.625407316	6.11E-10	1.58E-08
PARG27685	2.160401477	1.570757196	6.24E-10	1.61E-08
PARG22722	1.729333268	4.299540684	6.40E-10	1.65E-08
PARG13767	1.767168457	9.94507246	6.47E-10	1.67E-08
PARG15263	-1.776039317	3.334927841	6.72E-10	1.73E-08
PARG01451	-1.678402892	6.565048778	6.89E-10	1.77E-08
PARG11677	-2.097932188	0.8808458	7.13E-10	1.83E-08
PARG19857	-1.695059273	4.837508826	7.22E-10	1.85E-08
PARG19158	-1.70554285	4.278813783	7.30E-10	1.86E-08
PARG21456	1.671020481	8.864272828	7.38E-10	1.88E-08
PARG23908	-1.948620971	0.854472577	7.65E-10	1.94E-08
PARG30010	-1.807139378	2.629435776	7.65E-10	1.94E-08
PARG02477	2.428600582	4.175265458	7.95E-10	2.02E-08
PARG25669	-1.719575677	4.084640207	8.06E-10	2.04E-08
PARG25287	1.734158715	3.66417025	8.11E-10	2.05E-08
PARG00427	-1.912053256	1.746225504	8.37E-10	2.11E-08
PARG08741	-2.033899145	3.212514796	8.54E-10	2.15E-08
PARG19593	-1.677186636	5.866697807	8.74E-10	2.20E-08
PARG29436	2.10827031	2.2174641	8.86E-10	2.23E-08
PARG03727	1.847007611	3.670651625	8.89E-10	2.23E-08
PARG07500	-1.673046675	5.979898124	8.92E-10	2.23E-08
PARG15083	1.810213337	2.836451312	9.02E-10	2.25E-08
PARG16484	-1.664111633	6.967355005	9.06E-10	2.26E-08
PARG22952	-2.008335394	1.823479062	9.10E-10	2.27E-08
PARG03221	3.34916049	1.021476944	9.14E-10	2.27E-08
PARG27667	-1.725126024	3.659686546	9.17E-10	2.28E-08
PARG15733	2.303370678	2.719437512	9.18E-10	2.28E-08
PARG25396	-1.848921876	1.508940594	9.29E-10	2.30E-08
PARG25986	-2.410725601	1.247253769	9.30E-10	2.30E-08
PARG14709	-1.950306885	1.4484185	9.56E-10	2.36E-08
PARG04182	-1.720539653	2.617569302	9.81E-10	2.42E-08
PARG24151	3.931812793	1.048927538	9.84E-10	2.42E-08
PARG22993	1.755090284	4.545871839	1.01E-09	2.48E-08
PARG23452	-1.655861852	6.71898999	1.09E-09	2.67E-08
PARG08581	1.892040907	6.912156597	1.12E-09	2.74E-08

PARG15485	-1.675311729	3.91445385	1.16E-09	2.84E-08
PARG14810	-2.017917648	1.481967847	1.19E-09	2.90E-08
PARG06086	1.767648639	3.267826231	1.24E-09	3.01E-08
PARG15218	-1.688348639	4.473119159	1.27E-09	3.08E-08
PARG27682	-2.056429527	0.98947741	1.30E-09	3.16E-08
PARG15195	-2.824634565	0.470033415	1.31E-09	3.17E-08
PARG25815	-1.647092563	6.976259428	1.34E-09	3.25E-08
PARG28562	-1.697203903	3.652335769	1.38E-09	3.34E-08
PARG20183	-1.656674062	5.271388416	1.45E-09	3.50E-08
PARG12792	1.78276694	3.629908371	1.50E-09	3.61E-08
PARG23883	-2.303104755	-0.153585195	1.57E-09	3.78E-08
PARG29765	-1.713740771	2.64742808	1.58E-09	3.80E-08
PARG25195	-1.758186792	2.264801143	1.61E-09	3.86E-08
PARG04489	1.736737714	10.32133236	1.64E-09	3.94E-08
PARG10873	-1.650288214	4.854996673	1.68E-09	4.02E-08
PARG19415	-1.715046998	3.555328166	1.69E-09	4.04E-08
PARG18525	-1.649263954	4.900813778	1.71E-09	4.09E-08
PARG20219	1.652029061	6.116827228	1.72E-09	4.10E-08
PARG06613	-2.210646233	0.180831876	1.75E-09	4.16E-08
PARG07510	-3.259109704	-0.617339526	1.75E-09	4.17E-08
PARG19177	-1.672854093	3.781658605	1.77E-09	4.20E-08
PARG16214	3.44705565	0.083245188	1.79E-09	4.23E-08
PARG16194	-1.630192833	8.360597192	1.80E-09	4.25E-08
PARG09365	-2.22106176	1.08335979	1.82E-09	4.30E-08
PARG19099	1.715995376	3.456774845	1.88E-09	4.44E-08
PARG05770	-1.891339973	1.877796404	1.90E-09	4.48E-08
PARG11188	-1.734562413	2.047875037	1.92E-09	4.52E-08
PARG00705	1.629888735	8.071346576	1.94E-09	4.55E-08
PARG15430	-1.833349276	1.842786529	2.04E-09	4.78E-08
PARG06929	-1.646279594	4.862844844	2.05E-09	4.80E-08
PARG28068	1.683296067	5.574033798	2.06E-09	4.82E-08
PARG13095	1.624741463	8.138852535	2.11E-09	4.92E-08
PARG07400	-2.107552749	1.384426367	2.14E-09	4.97E-08
PARG27804	-1.688945523	6.06172814	2.14E-09	4.97E-08
PARG18357	-1.63804535	7.274809404	2.16E-09	5.02E-08
PARG27343	-1.641486496	6.305277776	2.17E-09	5.03E-08
PARG11389	2.480425428	0.568825111	2.18E-09	5.06E-08
PARG03079	1.811951173	3.429927357	2.20E-09	5.09E-08
PARG06205	-1.651828963	4.332511564	2.28E-09	5.26E-08
PARG12166	-1.68042853	3.007299628	2.34E-09	5.40E-08
PARG02859	-1.709383149	2.86145986	2.56E-09	5.90E-08
PARG13768	-1.7057342	4.940683828	2.57E-09	5.91E-08
PARG18444	-2.019066029	1.314239554	2.57E-09	5.91E-08
PARG05244	-1.64911348	4.456333064	2.59E-09	5.92E-08
PARG28010	1.727350096	3.039041804	2.59E-09	5.92E-08
PARG25356	-1.669993741	5.254177487	2.61E-09	5.95E-08

PARG11593	-1.835535549	1.984512252	2.61E-09	5.95E-08
PARG07712	-2.217702304	0.505678737	2.67E-09	6.07E-08
PARG01254	-1.661881112	4.514317486	2.79E-09	6.35E-08
PARG27449	-1.992863295	0.788789982	2.91E-09	6.61E-08
PARG07849	-2.374839395	0.508465669	2.92E-09	6.63E-08
PARG00827	-1.801500983	1.718007382	3.00E-09	6.79E-08
PARG16088	-1.953384213	1.170973982	3.03E-09	6.86E-08
PARG26295	-1.837617972	2.314869365	3.09E-09	6.98E-08
PARG07605	2.297827831	1.999272494	3.11E-09	7.02E-08
PARG03407	-3.943906282	-0.469945358	3.21E-09	7.24E-08
PARG12486	-1.607729009	6.735443472	3.24E-09	7.29E-08
PARG27939	1.856488857	3.264813274	3.30E-09	7.41E-08
PARG30015	-1.659466391	6.548153575	3.34E-09	7.49E-08
PARG03373	-3.180267487	0.642334471	3.38E-09	7.57E-08
PARG25398	-1.856250357	1.387157629	3.60E-09	8.05E-08
PARG23593	1.793971635	2.640126036	3.70E-09	8.26E-08
PARG15352	-1.988427438	0.86761682	3.72E-09	8.29E-08
PARG26627	-1.956211818	0.495434983	3.72E-09	8.29E-08
PARG06863	-2.166377345	-0.042272448	3.85E-09	8.57E-08
PARG27943	-1.832976059	2.554408081	3.88E-09	8.61E-08
PARG26624	-2.021384338	0.033144792	3.95E-09	8.77E-08
PARG15367	-1.737760673	2.154186026	4.00E-09	8.85E-08
PARG08347	-1.607415924	5.438234211	4.02E-09	8.89E-08
PARG24502	1.598575066	6.599835615	4.05E-09	8.95E-08
PARG24749	1.592509581	8.271552864	4.07E-09	8.98E-08
PARG08766	-2.006641637	1.979604014	4.11E-09	9.05E-08
PARG06050	-1.826396246	2.286627561	4.47E-09	9.82E-08
PARG18093	1.784993739	2.535360113	4.72E-09	1.04E-07
PARG16140	-1.98585284	2.557633171	4.80E-09	1.05E-07
PARG26921	-1.816350862	1.949575343	4.87E-09	1.07E-07
PARG29278	-1.587487641	6.973959547	5.03E-09	1.10E-07
PARG20983	-1.840176378	1.434959109	5.13E-09	1.12E-07
PARG10466	1.592605479	6.180345348	5.14E-09	1.12E-07
PARG14511	-1.995252747	1.060968781	5.14E-09	1.12E-07
PARG01361	1.8455042	5.94419924	5.18E-09	1.13E-07
PARG01962	-2.262043269	0.757638144	5.26E-09	1.14E-07
PARG02891	-1.878666907	1.347776617	5.35E-09	1.16E-07
PARG24964	-1.66993453	3.073968283	5.38E-09	1.16E-07
PARG05777	-1.602099184	4.780806355	5.58E-09	1.21E-07
PARG08024	-1.626273914	4.0373201	5.63E-09	1.22E-07
PARG07968	1.659340169	4.726055104	5.81E-09	1.25E-07
PARG19748	1.591788135	6.933401012	6.01E-09	1.29E-07
PARG15350	-1.828732645	1.418371256	6.10E-09	1.31E-07
PARG19915	1.688464038	3.7368189	6.14E-09	1.32E-07
PARG03416	-2.036757481	0.572630058	6.34E-09	1.36E-07
PARG27276	-1.647548814	3.077843332	6.39E-09	1.37E-07

PARG18069	-2.115550231	0.36954499	6.72E-09	1.44E-07
PARG12427	1.617014608	4.678304475	7.16E-09	1.53E-07
PARG27758	-1.909078652	1.976316936	7.19E-09	1.53E-07
PARG12459	1.631158455	3.106791114	7.36E-09	1.57E-07
PARG04962	3.339698039	0.614003379	7.53E-09	1.60E-07
PARG07306	-1.727062024	2.11535991	7.59E-09	1.61E-07
PARG15204	-6.485848927	-0.756194101	7.63E-09	1.62E-07
PARG20891	-1.869284979	1.653810249	7.95E-09	1.68E-07
PARG18310	1.85116305	2.524247231	7.96E-09	1.68E-07
PARG10875	-1.56361234	6.035576856	8.72E-09	1.84E-07
PARG12125	-1.875543952	1.252930894	8.81E-09	1.86E-07
PARG07787	-1.565680385	6.352319769	8.94E-09	1.88E-07
PARG08352	1.55828155	7.662705228	9.08E-09	1.91E-07
PARG07213	-1.564441801	5.443566521	9.45E-09	1.99E-07
PARG06442	2.081305269	2.704083164	9.59E-09	2.01E-07
PARG25314	-2.960709321	-0.475106951	9.71E-09	2.03E-07
PARG20148	-1.661863418	2.527398892	9.71E-09	2.03E-07
PARG06949	-1.575449719	4.536934735	9.73E-09	2.03E-07
PARG29282	-1.730326643	2.722131099	9.73E-09	2.03E-07
PARG27337	-1.782850562	1.474583682	9.75E-09	2.03E-07
PARG08459	1.557587553	6.416337495	9.90E-09	2.06E-07
PARG13439	-1.648981555	2.762159656	1.00E-08	2.09E-07
PARG02868	1.548080824	10.7121184	1.01E-08	2.09E-07
PARG01391	4.777026074	0.092248019	1.02E-08	2.11E-07
PARG06899	-1.595887366	3.501454111	1.04E-08	2.15E-07
PARG12781	1.583583377	4.780298794	1.06E-08	2.20E-07
PARG18434	-1.566165954	4.635326802	1.09E-08	2.25E-07
PARG27761	-2.975414062	0.715287982	1.12E-08	2.31E-07
PARG18443	-1.596688516	3.568764174	1.12E-08	2.31E-07
PARG08956	1.542976536	7.963268819	1.18E-08	2.43E-07
PARG27129	-1.541346335	8.688777252	1.18E-08	2.43E-07
PARG17097	-1.662360974	2.430612895	1.19E-08	2.45E-07
PARG12499	-2.160847542	0.268055051	1.21E-08	2.48E-07
PARG27138	-1.559786232	5.094672865	1.22E-08	2.49E-07
PARG20785	6.439070865	0.929062306	1.26E-08	2.58E-07
PARG29929	-1.549073264	6.363926469	1.26E-08	2.58E-07
PARG01357	2.078760139	1.188811612	1.27E-08	2.59E-07
PARG19410	-1.631205448	2.967590333	1.27E-08	2.59E-07
PARG26445	-1.631827071	3.695828404	1.28E-08	2.59E-07
PARG24376	-3.127926764	0.066971404	1.28E-08	2.59E-07
PARG14248	-1.553403137	5.768277729	1.29E-08	2.62E-07
PARG21084	-1.829105022	2.123874595	1.29E-08	2.62E-07
PARG11792	-1.571890017	3.845794816	1.31E-08	2.65E-07
PARG19573	-2.936043928	1.625004778	1.32E-08	2.67E-07
PARG06984	1.535934559	9.010691114	1.35E-08	2.72E-07
PARG00493	-1.551711909	5.638679805	1.36E-08	2.75E-07

PARG06154	-2.491302938	-0.433216748	1.39E-08	2.80E-07
PARG04944	-1.552422197	4.619562067	1.39E-08	2.80E-07
PARG11868	-1.553708589	5.022469338	1.42E-08	2.85E-07
PARG02102	-1.546620895	6.052944805	1.42E-08	2.85E-07
PARG01827	2.14716915	2.579108973	1.45E-08	2.90E-07
PARG10046	-1.822803233	1.137890036	1.45E-08	2.90E-07
PARG27319	-2.119363883	0.405898957	1.47E-08	2.93E-07
PARG06171	-1.55544588	4.881995302	1.47E-08	2.93E-07
PARG01175	-1.624054594	3.115053957	1.47E-08	2.93E-07
PARG25755	-2.301942803	0.099634831	1.50E-08	2.99E-07
PARG16841	-1.806289188	1.659496539	1.50E-08	2.99E-07
PARG02154	-1.573387216	3.976308616	1.50E-08	2.99E-07
PARG13955	-1.605204702	3.441965646	1.61E-08	3.19E-07
PARG05596	-2.219718921	-0.683957582	1.64E-08	3.25E-07
PARG18962	-1.618553187	3.32040785	1.64E-08	3.25E-07
PARG23933	1.537885971	5.523510656	1.68E-08	3.31E-07
PARG21807	1.530112848	6.49927069	1.68E-08	3.32E-07
PARG18737	-1.522949584	9.197080572	1.71E-08	3.37E-07
PARG26882	-2.518226402	-0.186763531	1.80E-08	3.53E-07
PARG00717	-1.660923521	2.55351133	1.85E-08	3.64E-07
PARG19819	1.528503625	8.147501767	1.86E-08	3.64E-07
PARG13646	-1.724181796	1.500446923	1.86E-08	3.64E-07
PARG16311	-1.800861107	2.357731834	2.02E-08	3.95E-07
PARG21232	-1.648772526	3.008732309	2.04E-08	4.00E-07
PARG22971	2.863066138	0.829186391	2.05E-08	4.01E-07
PARG22747	1.73605006	3.467669988	2.08E-08	4.06E-07
PARG30014	-1.780015227	1.405761931	2.11E-08	4.11E-07
PARG21624	1.552452655	5.50210444	2.12E-08	4.13E-07
PARG18610	2.413295821	6.241156788	2.15E-08	4.17E-07
PARG07227	1.564805153	5.93430044	2.16E-08	4.19E-07
PARG25311	-1.580692536	3.16870845	2.17E-08	4.21E-07
PARG08428	-1.595287006	3.929112145	2.29E-08	4.43E-07
PARG27339	1.522863549	6.034319959	2.30E-08	4.44E-07
PARG00510	-1.762684859	1.551415972	2.34E-08	4.51E-07
PARG11152	-1.646254332	2.256872735	2.34E-08	4.51E-07
PARG06519	-1.702676561	1.928495736	2.35E-08	4.52E-07
PARG21665	-1.894880623	1.529834236	2.37E-08	4.56E-07
PARG19916	1.698947544	2.417696427	2.40E-08	4.60E-07
PARG07002	1.874752253	2.320805862	2.47E-08	4.74E-07
PARG14152	-1.661972777	2.560202103	2.48E-08	4.75E-07
PARG29654	-1.543127662	5.14333846	2.58E-08	4.94E-07
PARG28527	-1.610990748	3.206882951	2.71E-08	5.19E-07
PARG24166	-1.613959533	2.501719112	2.81E-08	5.36E-07
PARG14456	-1.587706192	3.422429578	2.83E-08	5.40E-07
PARG09540	-1.570462344	3.777647813	2.84E-08	5.42E-07
PARG06427	-1.609711614	3.829001666	2.88E-08	5.48E-07

PARG21175	-1.527192806	4.312290126	2.91E-08	5.53E-07
PARG02413	1.499804736	8.144137546	2.93E-08	5.55E-07
PARG04069	2.105860988	3.286864706	2.95E-08	5.59E-07
PARG17109	-1.536838509	4.409081972	2.96E-08	5.60E-07
PARG02041	2.211747262	2.214495145	2.97E-08	5.61E-07
PARG11844	-1.504559829	5.568860605	3.00E-08	5.67E-07
PARG19374	-1.599583778	6.273275777	3.02E-08	5.69E-07
PARG26918	-1.514584009	3.8338281	3.02E-08	5.69E-07
PARG26398	-1.588398792	3.396406924	3.07E-08	5.78E-07
PARG04038	-1.57314385	3.267692484	3.32E-08	6.24E-07
PARG24063	1.591659546	4.45728561	3.32E-08	6.24E-07
PARG23244	-2.879074371	-0.715004839	3.37E-08	6.32E-07
PARG17984	-1.515360016	5.001701038	3.38E-08	6.34E-07
PARG23832	-1.523309709	5.512588284	3.39E-08	6.34E-07
PARG09273	-1.597621881	2.81898495	3.44E-08	6.42E-07
PARG06756	-1.575676179	2.701162583	3.52E-08	6.57E-07
PARG22667	-2.267991251	-0.074999376	3.52E-08	6.57E-07
PARG24835	-1.648173935	2.14952939	3.53E-08	6.58E-07
PARG13752	-1.773279793	0.98300445	3.55E-08	6.60E-07
PARG07517	1.555204411	2.952461446	3.59E-08	6.67E-07
PARG15650	-3.755385119	0.1383388	3.65E-08	6.76E-07
PARG07816	1.491074248	8.459370406	3.67E-08	6.80E-07
PARG11404	-1.738640742	1.320399817	3.67E-08	6.80E-07
PARG05785	-1.500572766	5.036873526	3.71E-08	6.86E-07
PARG05989	-1.491807965	6.430645549	3.73E-08	6.88E-07
PARG20145	-1.705035417	1.911134566	3.75E-08	6.91E-07
PARG03816	1.493044611	6.449099955	3.77E-08	6.95E-07
PARG10610	-1.493049299	5.975474699	3.78E-08	6.95E-07
PARG16516	-1.724614305	4.519420844	3.81E-08	7.01E-07
PARG14065	-1.518416538	3.706237232	3.95E-08	7.26E-07
PARG28165	1.55935164	5.632091367	4.10E-08	7.52E-07
PARG27134	-1.963123309	0.897321363	4.15E-08	7.61E-07
PARG07325	-1.698563046	1.676438847	4.16E-08	7.61E-07
PARG08905	-1.480896299	8.142971262	4.20E-08	7.68E-07
PARG08327	-1.497230746	4.686603912	4.22E-08	7.71E-07
PARG07282	-1.488753897	5.485674749	4.24E-08	7.73E-07
PARG27184	1.566567731	3.495821932	4.30E-08	7.82E-07
PARG16557	-2.301063595	0.413192231	4.31E-08	7.83E-07
PARG02372	1.843013181	3.333630102	4.37E-08	7.94E-07
PARG17771	1.499873809	4.977079533	4.38E-08	7.95E-07
PARG27759	-2.146307736	0.490792017	4.41E-08	8.00E-07
PARG21997	1.61042453	2.75871944	4.43E-08	8.02E-07
PARG29417	1.48298218	7.345827812	4.46E-08	8.07E-07
PARG25180	-1.568440619	2.866277116	4.49E-08	8.12E-07
PARG22886	1.968995225	0.742821048	4.58E-08	8.26E-07
PARG21957	1.515703502	5.505264097	4.64E-08	8.36E-07

PARG15447	-1.667230559	1.64003614	4.70E-08	8.46E-07
PARG20147	-1.565278876	2.463101386	4.71E-08	8.46E-07
PARG15193	-1.491297991	5.562150308	4.74E-08	8.52E-07
PARG21388	1.4843015	6.253029221	4.81E-08	8.62E-07
PARG22201	-1.511439401	4.230822529	4.82E-08	8.65E-07
PARG08535	-1.714935034	1.92680901	4.85E-08	8.68E-07
PARG18978	-1.509015776	4.378299673	5.03E-08	8.99E-07
PARG24627	-1.525868596	3.02432379	5.03E-08	8.99E-07
PARG18826	-1.649814303	1.879168261	5.06E-08	9.03E-07
PARG04115	-2.633393537	-0.532535016	5.08E-08	9.04E-07
PARG10647	-1.949291551	0.914068685	5.08E-08	9.04E-07
PARG06132	-1.738107308	2.686797916	5.09E-08	9.04E-07
PARG02084	-1.527758281	3.334837659	5.12E-08	9.09E-07
PARG04501	1.919192758	2.485993827	5.23E-08	9.28E-07
PARG29449	1.4770679	6.812604202	5.24E-08	9.28E-07
PARG08437	1.47317921	7.459780481	5.30E-08	9.38E-07
PARG23354	-1.721059243	1.276373246	5.48E-08	9.67E-07
PARG27336	-2.191055181	-0.180688416	5.48E-08	9.67E-07
PARG03372	-2.189970745	0.232381647	5.48E-08	9.67E-07
PARG12859	-1.471554452	6.346687878	5.53E-08	9.74E-07
PARG23385	-1.79640444	0.891293818	5.55E-08	9.76E-07
PARG23910	-1.619065074	1.43395363	5.56E-08	9.77E-07
PARG05634	-1.524211995	4.539550383	5.61E-08	9.85E-07
PARG01764	-1.984887545	-0.102412059	5.68E-08	9.96E-07
PARG15680	-1.495471189	5.417796303	5.86E-08	1.03E-06
PARG08987	-1.961586768	0.496925076	5.92E-08	1.04E-06
PARG21167	-1.527967126	3.528964651	6.09E-08	1.06E-06
PARG19386	1.459129244	9.765601424	6.17E-08	1.08E-06
PARG06970	1.478465327	5.232431932	6.23E-08	1.09E-06
PARG11904	-1.540274494	3.405231306	6.25E-08	1.09E-06
PARG10344	-1.716197685	0.861358955	6.29E-08	1.09E-06
PARG00711	-2.532961301	-0.169093827	6.35E-08	1.10E-06
PARG13050	1.650383122	2.015205065	6.36E-08	1.10E-06
PARG29248	-1.611691362	2.395422464	6.37E-08	1.11E-06
PARG20793	1.579910605	9.31716554	6.46E-08	1.12E-06
PARG29344	-1.724033443	1.180715372	6.47E-08	1.12E-06
PARG01985	-1.60752683	2.410912888	6.49E-08	1.12E-06
PARG28101	-4.680175193	2.188905858	6.60E-08	1.14E-06
PARG07796	-1.635238402	2.457281602	6.68E-08	1.15E-06
PARG10652	-1.89630773	0.745725027	6.76E-08	1.17E-06
PARG10595	-2.260581861	-0.448991996	6.83E-08	1.18E-06
PARG06578	-1.513848193	3.39521291	6.86E-08	1.18E-06
PARG14178	-1.541804514	2.848205436	7.41E-08	1.27E-06
PARG18635	1.514552522	5.80114477	7.45E-08	1.28E-06
PARG06343	-2.885788695	0.12030062	7.86E-08	1.35E-06
PARG04360	-1.81795899	0.846863109	8.00E-08	1.37E-06

PARG21150	-1.45995251	5.604739222	8.00E-08	1.37E-06
PARG07592	-2.122901566	1.615770578	8.02E-08	1.37E-06
PARG20887	-2.133690899	0.148425605	8.15E-08	1.39E-06
PARG27641	-1.560095166	2.294274289	8.19E-08	1.40E-06
PARG07898	-1.628541794	1.933686945	8.20E-08	1.40E-06
PARG27970	-3.12079524	-0.988331661	8.38E-08	1.42E-06
PARG13731	-3.107381058	-0.803620855	8.38E-08	1.42E-06
PARG24209	-1.457341174	5.690289932	8.45E-08	1.43E-06
PARG21648	-2.372992505	0.024644534	8.48E-08	1.44E-06
PARG19664	-1.651193151	1.794899252	8.49E-08	1.44E-06
PARG04407	1.45798131	7.082876272	8.53E-08	1.44E-06
PARG16380	-1.491587542	4.075807044	8.56E-08	1.45E-06
PARG01703	-2.351065016	-0.095595577	8.59E-08	1.45E-06
PARG26537	-1.454401581	6.503964693	8.75E-08	1.48E-06
PARG26482	-1.591981048	2.370250183	8.94E-08	1.51E-06
PARG19779	1.755940749	3.33980402	9.35E-08	1.57E-06
PARG11455	-1.487210604	3.455246427	9.49E-08	1.60E-06
PARG14416	-1.4769717	4.069459873	9.57E-08	1.61E-06
PARG11898	-1.997662638	0.27004038	9.68E-08	1.62E-06
PARG07422	-1.506092949	3.005897598	9.73E-08	1.63E-06
PARG19350	-2.984854848	0.426482939	9.75E-08	1.63E-06
PARG06642	-1.622056761	1.868870386	9.76E-08	1.63E-06
PARG09181	-1.496008304	3.495993002	9.76E-08	1.63E-06
PARG12713	-3.232934141	-0.838601287	9.98E-08	1.67E-06
PARG20944	1.940048054	1.411035103	1.00E-07	1.67E-06
PARG00582	-1.452091904	5.422641511	1.00E-07	1.67E-06
PARG19665	-1.478829414	4.085427414	1.02E-07	1.69E-06
PARG13592	1.638449412	4.573005227	1.04E-07	1.73E-06
PARG11333	1.433956872	8.525313853	1.05E-07	1.74E-06
PARG02134	-1.467090781	4.329989878	1.07E-07	1.77E-06
PARG26804	-2.217156073	-0.297029575	1.09E-07	1.80E-06
PARG19222	-1.437178133	6.132473518	1.09E-07	1.81E-06
PARG07366	2.76541312	1.436682116	1.09E-07	1.81E-06
PARG08688	2.44807679	0.713095352	1.10E-07	1.82E-06
PARG08629	2.343788246	2.311690505	1.11E-07	1.82E-06
PARG11658	-1.591796852	1.705830736	1.11E-07	1.83E-06
PARG27305	-1.867851253	0.182307666	1.12E-07	1.85E-06
PARG27228	-1.791582286	1.026677403	1.13E-07	1.85E-06
PARG21480	-1.519534267	3.728258856	1.16E-07	1.91E-06
PARG19921	1.435801114	7.213806315	1.17E-07	1.93E-06
PARG20008	-1.822376673	0.578418139	1.19E-07	1.95E-06
PARG15182	-2.113184649	-0.173091431	1.23E-07	2.01E-06
PARG13641	-1.712116769	1.037538838	1.23E-07	2.01E-06
PARG26919	-1.706050943	0.336938187	1.24E-07	2.03E-06
PARG13867	-1.502057936	3.10885432	1.25E-07	2.04E-06
PARG01683	-2.127221721	-0.093620752	1.26E-07	2.05E-06

PARG20318	-1.564331493	2.345373051	1.29E-07	2.09E-06
PARG16074	-1.671520295	3.683968051	1.30E-07	2.12E-06
PARG03225	-1.704864898	1.629645418	1.30E-07	2.12E-06
PARG18650	-1.475609236	3.71278552	1.31E-07	2.12E-06
PARG16777	-1.424782472	6.62937097	1.31E-07	2.13E-06
PARG11828	1.473401384	6.501917664	1.31E-07	2.13E-06
PARG05579	-2.152553679	0.436208164	1.31E-07	2.13E-06
PARG05638	-1.518212973	2.047848204	1.32E-07	2.14E-06
PARG02771	-1.483189625	3.876021523	1.35E-07	2.18E-06
PARG24385	-1.449050667	4.559205463	1.35E-07	2.18E-06
PARG16210	-1.713871583	0.888277923	1.35E-07	2.18E-06
PARG03109	-1.474631545	4.385657973	1.36E-07	2.19E-06
PARG25424	-2.021751707	-0.058390617	1.38E-07	2.22E-06
PARG14084	-1.417872368	9.466798252	1.38E-07	2.22E-06
PARG14473	1.536517507	3.958383737	1.39E-07	2.23E-06
PARG25753	-1.703047163	0.731276933	1.39E-07	2.24E-06
PARG28244	-1.546022083	2.40781015	1.40E-07	2.24E-06
PARG10286	-2.276372348	0.02181599	1.42E-07	2.27E-06
PARG17593	1.793436598	4.184181299	1.44E-07	2.30E-06
PARG13595	1.422725118	7.859011092	1.44E-07	2.30E-06
PARG11477	1.658738878	2.203743865	1.45E-07	2.31E-06
PARG07947	-3.235383621	-0.637626175	1.45E-07	2.32E-06
PARG23911	-1.923389803	-0.276949453	1.46E-07	2.32E-06
PARG19855	-1.621538526	2.986615857	1.47E-07	2.34E-06
PARG14150	-1.495886859	3.128967359	1.48E-07	2.35E-06
PARG16490	-1.428715337	5.645235328	1.48E-07	2.36E-06
PARG29289	-1.737902118	1.815413937	1.50E-07	2.38E-06
PARG27901	-1.833244159	0.716109264	1.50E-07	2.38E-06
PARG11409	-1.543083131	2.251623813	1.54E-07	2.44E-06
PARG14211	-1.803609606	0.504614203	1.55E-07	2.45E-06
PARG27725	1.425743515	6.285481546	1.58E-07	2.49E-06
PARG27497	-1.444419811	6.84570749	1.59E-07	2.50E-06
PARG25263	2.062406538	0.86989856	1.62E-07	2.55E-06
PARG23917	-1.487943689	2.46785716	1.65E-07	2.60E-06
PARG24039	-1.611250054	2.037601617	1.66E-07	2.61E-06
PARG26898	-2.048898449	-0.130494639	1.67E-07	2.63E-06
PARG02252	-1.442170879	4.563724118	1.68E-07	2.63E-06
PARG23123	-1.478429485	3.58362599	1.70E-07	2.67E-06
PARG27711	1.799864408	2.098076698	1.70E-07	2.67E-06
PARG12504	1.652792755	1.822178543	1.71E-07	2.67E-06
PARG10024	4.527596998	-0.442530873	1.72E-07	2.68E-06
PARG02749	-3.071022804	1.280207443	1.72E-07	2.68E-06
PARG16811	1.550769686	6.321661088	1.73E-07	2.70E-06
PARG20097	-2.228769266	-0.042210908	1.79E-07	2.79E-06
PARG18544	-1.8690679	0.352094919	1.80E-07	2.81E-06
PARG27665	1.557366754	2.672981546	1.80E-07	2.81E-06

PARG10508	-1.464271344	3.51729505	1.81E-07	2.81E-06
PARG12660	-2.286932775	0.107844169	1.84E-07	2.86E-06
PARG24292	-1.505515022	2.368769421	1.85E-07	2.87E-06
PARG19209	-1.659869996	1.193453237	1.91E-07	2.95E-06
PARG27437	-1.617682715	2.719164219	1.92E-07	2.97E-06
PARG05546	-1.424823335	4.648925776	1.92E-07	2.97E-06
PARG27318	-1.429272798	4.422574904	1.94E-07	3.00E-06
PARG05667	-1.546243654	2.447811677	1.97E-07	3.04E-06
PARG12943	1.452630622	4.581021611	2.00E-07	3.09E-06
PARG06828	-1.606897025	2.645402868	2.02E-07	3.11E-06
PARG06696	1.402173466	7.166142741	2.09E-07	3.22E-06
PARG07172	-1.39854768	7.544425616	2.11E-07	3.24E-06
PARG06522	-1.421528374	5.165018934	2.14E-07	3.28E-06
PARG22134	1.842885931	2.163063113	2.14E-07	3.28E-06
PARG23985	-1.453682276	3.142116895	2.16E-07	3.32E-06
PARG08138	-1.643215036	1.449004385	2.22E-07	3.40E-06
PARG26868	-1.788071663	0.002273992	2.27E-07	3.47E-06
PARG27403	-2.227589253	0.06458022	2.28E-07	3.49E-06
PARG09308	-1.600094139	1.989054021	2.28E-07	3.49E-06
PARG14381	-1.608623451	1.725948258	2.36E-07	3.60E-06
PARG00338	-1.402380155	5.093040606	2.39E-07	3.65E-06
PARG02954	-1.428427868	4.040026067	2.40E-07	3.66E-06
PARG26938	-1.491585351	2.836519142	2.43E-07	3.70E-06
PARG20890	-3.037914796	-0.600263809	2.47E-07	3.75E-06
PARG29447	-1.506268056	2.220708933	2.51E-07	3.80E-06
PARG16796	2.146833795	5.138063728	2.54E-07	3.85E-06
PARG06400	-1.439097681	3.545335827	2.55E-07	3.86E-06
PARG05179	-1.434080983	3.195865925	2.55E-07	3.86E-06
PARG18642	-1.47601406	2.705874127	2.57E-07	3.89E-06
PARG04424	1.466022866	3.473599978	2.64E-07	3.99E-06
PARG08735	-1.386308978	7.440224195	2.71E-07	4.09E-06
PARG27074	-1.473178526	3.345961817	2.72E-07	4.10E-06
PARG10000	1.401057566	4.508410345	2.79E-07	4.21E-06
PARG13744	-1.513286313	4.565646275	2.81E-07	4.23E-06
PARG14129	-1.383978838	7.497029012	2.84E-07	4.26E-06
PARG19908	1.588253338	3.826929939	2.91E-07	4.38E-06
PARG11861	-1.385388785	7.138212719	2.95E-07	4.42E-06
PARG29383	1.549418173	3.428851388	2.96E-07	4.44E-06
PARG05595	1.385303711	6.884903532	3.02E-07	4.53E-06
PARG20913	-1.801531014	0.739283522	3.11E-07	4.65E-06
PARG13081	-1.633432245	1.244677999	3.13E-07	4.67E-06
PARG25395	-1.962058163	-0.402499413	3.14E-07	4.69E-06
PARG20643	-1.467098788	2.920474148	3.19E-07	4.75E-06
PARG02101	1.391394243	5.883801347	3.21E-07	4.78E-06
PARG18272	-1.410790301	4.375946804	3.22E-07	4.79E-06
PARG20561	-1.480152566	2.340132712	3.23E-07	4.80E-06

PARG16615	1.412066925	4.19283559	3.24E-07	4.81E-06
PARG30213	-1.466242376	2.641602976	3.26E-07	4.84E-06
PARG24048	-1.480867181	2.92657663	3.28E-07	4.87E-06
PARG21434	-1.470805267	3.825951546	3.29E-07	4.88E-06
PARG19185	1.522223053	2.879978387	3.37E-07	4.99E-06
PARG10756	1.400426639	4.380988341	3.41E-07	5.05E-06
PARG24340	1.39978949	4.652395436	3.54E-07	5.23E-06
PARG21214	-1.393236043	4.356236915	3.56E-07	5.26E-06
PARG03714	1.763804075	3.298491373	3.57E-07	5.27E-06
PARG11921	-1.488955173	2.465199106	3.58E-07	5.27E-06
PARG03636	-1.604935816	1.686777877	3.60E-07	5.31E-06
PARG26460	-1.989083022	0.305286591	3.63E-07	5.34E-06
PARG18819	1.916913448	1.052931412	3.67E-07	5.40E-06
PARG29458	-1.765713706	0.976478385	3.76E-07	5.52E-06
PARG15872	-2.00321655	-0.052410641	3.82E-07	5.59E-06
PARG13018	1.462936781	4.760762407	3.82E-07	5.59E-06
PARG27455	1.433429593	3.526591479	3.83E-07	5.60E-06
PARG04362	-1.450525515	4.019899494	3.87E-07	5.66E-06
PARG14484	1.630912342	1.675968434	3.91E-07	5.71E-06
PARG19660	-1.421093976	3.617574538	3.92E-07	5.72E-06
PARG06222	-1.447566885	3.084890411	3.93E-07	5.74E-06
PARG06100	-1.458984534	3.261754834	3.96E-07	5.77E-06
PARG16319	-1.597601543	1.283053861	4.00E-07	5.82E-06
PARG07417	1.378279699	6.254737116	4.01E-07	5.84E-06
PARG04521	-2.262109451	-0.362285144	4.10E-07	5.95E-06
PARG08608	-1.580818895	1.093749946	4.13E-07	6.00E-06
PARG28064	-2.112397279	0.833163766	4.14E-07	6.00E-06
PARG09023	1.713326685	2.44928533	4.15E-07	6.02E-06
PARG15794	1.424021765	3.418506693	4.16E-07	6.02E-06
PARG17831	-1.531358178	1.321504846	4.23E-07	6.12E-06
PARG17415	-1.438623312	2.690198452	4.25E-07	6.14E-06
PARG02066	-1.739711463	0.73519827	4.26E-07	6.15E-06
PARG17960	-2.130996814	-0.665493671	4.33E-07	6.25E-06
PARG21173	-1.379050901	4.656804801	4.35E-07	6.27E-06
PARG02466	-1.490835023	2.450268178	4.35E-07	6.27E-06
PARG18974	2.587689744	-0.174664373	4.38E-07	6.30E-06
PARG06590	-1.380565795	5.664974344	4.45E-07	6.40E-06
PARG06007	-1.703338858	0.831638046	4.48E-07	6.43E-06
PARG24597	1.574844956	1.635293725	4.49E-07	6.44E-06
PARG12700	1.364856491	6.436882359	4.50E-07	6.46E-06
PARG07873	1.35958983	7.596973568	4.52E-07	6.47E-06
PARG13543	-2.05472815	0.938107115	4.68E-07	6.69E-06
PARG15836	-1.729814211	0.136273503	4.77E-07	6.82E-06
PARG04134	-1.369629608	5.223573305	4.80E-07	6.86E-06
PARG08429	-1.502361974	2.451014098	4.82E-07	6.87E-06
PARG24421	-1.464731207	2.705150502	4.90E-07	6.98E-06

PARG25200	-1.367868938	5.157504887	4.92E-07	7.01E-06
PARG17428	2.103531627	0.399317016	4.93E-07	7.01E-06
PARG06922	-1.363211235	5.473319704	5.14E-07	7.30E-06
PARG02145	-1.408744939	3.517584567	5.16E-07	7.32E-06
PARG06711	-1.437570653	3.132696454	5.16E-07	7.32E-06
PARG27757	-1.535355068	2.912354412	5.19E-07	7.37E-06
PARG03512	-2.246465733	0.737704538	5.34E-07	7.56E-06
PARG05769	-1.514743254	1.915410022	5.38E-07	7.61E-06
PARG07850	-1.366830881	4.649678035	5.46E-07	7.72E-06
PARG11831	-1.445045425	3.276424304	5.54E-07	7.83E-06
PARG05694	-2.331901861	-0.509709057	5.68E-07	8.02E-06
PARG06109	-1.472024397	2.365954359	5.69E-07	8.02E-06
PARG11852	-3.276947649	-0.109342141	5.70E-07	8.03E-06
PARG23386	-1.360203757	5.05031386	5.78E-07	8.13E-06
PARG06488	-1.572454016	1.528678261	5.83E-07	8.20E-06
PARG12316	-1.365209563	4.949289513	5.84E-07	8.21E-06
PARG12013	-2.651318986	-0.92626469	5.90E-07	8.28E-06
PARG15463	-1.349030837	9.020114558	5.92E-07	8.31E-06
PARG03046	-1.404497567	4.569610761	5.97E-07	8.36E-06
PARG29144	1.644941375	0.801628499	6.00E-07	8.40E-06
PARG07336	-1.515199483	1.600065468	6.26E-07	8.76E-06
PARG11108	-1.344936099	5.933578756	6.32E-07	8.83E-06
PARG12455	-1.400311296	3.858458238	6.42E-07	8.97E-06
PARG03553	-1.388365391	3.849543096	6.44E-07	8.98E-06
PARG08215	1.857407192	0.03577299	6.52E-07	9.09E-06
PARG23842	1.346623595	6.140199983	6.53E-07	9.10E-06
PARG02357	-1.539705117	1.595790842	6.54E-07	9.11E-06
PARG14153	-1.451925782	4.324102709	6.56E-07	9.12E-06
PARG24428	-2.199325623	-0.578560073	6.65E-07	9.23E-06
PARG24277	2.983673085	-0.014861311	6.65E-07	9.23E-06
PARG18960	-1.400658349	3.192804626	6.92E-07	9.60E-06
PARG14600	-1.93148012	0.149811416	6.96E-07	9.65E-06
PARG19725	-1.343159952	6.213664142	7.19E-07	9.95E-06
PARG12240	1.437557789	2.066021923	7.20E-07	9.95E-06
PARG29151	-1.376653845	3.60680758	7.25E-07	1.00E-05
PARG04104	-1.579953991	1.161746835	7.31E-07	1.01E-05
PARG07781	-1.712282597	0.648648117	7.36E-07	1.02E-05
PARG01460	-1.501789742	1.590646509	7.49E-07	1.03E-05
PARG10640	-1.349735505	5.457634849	7.53E-07	1.04E-05
PARG19105	-1.599324719	2.743317911	7.56E-07	1.04E-05
PARG19204	-1.33966596	6.322602488	7.61E-07	1.05E-05
PARG29433	1.335150981	7.904554396	7.81E-07	1.07E-05
PARG09033	-1.652891202	0.800361933	7.88E-07	1.08E-05
PARG08767	-2.37339551	0.156554253	7.98E-07	1.09E-05
PARG21521	1.341780175	6.615949138	7.98E-07	1.09E-05
PARG06383	-1.388273433	3.354444838	8.00E-07	1.09E-05

PARG14488	1.354644506	5.061025974	8.11E-07	1.11E-05
PARG15383	-1.480517008	2.297830425	8.11E-07	1.11E-05
PARG15342	-1.597648241	1.406541618	8.12E-07	1.11E-05
PARG19448	-2.620520175	-0.845427407	8.15E-07	1.11E-05
PARG18475	1.337354933	8.471933642	8.19E-07	1.12E-05
PARG10003	6.040491008	1.884377916	8.23E-07	1.12E-05
PARG20103	5.990190012	0.391875076	8.23E-07	1.12E-05
PARG13219	-1.539421523	1.319534159	8.26E-07	1.12E-05
PARG09182	-1.362650342	4.407941332	8.26E-07	1.12E-05
PARG24538	-1.931743594	-0.402603844	8.45E-07	1.15E-05
PARG00284	-1.518990333	1.322064341	8.56E-07	1.16E-05
PARG01206	1.389011698	3.221642549	8.59E-07	1.16E-05
PARG21748	-1.33501296	4.756333893	8.63E-07	1.17E-05
PARG01456	-1.341332138	5.216448333	8.77E-07	1.19E-05
PARG10674	-1.37899892	4.019512905	8.92E-07	1.20E-05
PARG04908	-1.323165401	6.898835134	8.95E-07	1.21E-05
PARG00836	1.331650016	5.657746864	9.12E-07	1.23E-05
PARG26706	-1.350278512	4.46780456	9.15E-07	1.23E-05
PARG07478	-1.42509958	2.094046399	9.19E-07	1.24E-05
PARG07136	1.350751729	4.864089481	9.26E-07	1.25E-05
PARG24419	-1.407554887	1.992789114	9.50E-07	1.28E-05
PARG08340	1.874974409	6.385860039	9.89E-07	1.33E-05
PARG07527	-1.42853841	2.442558393	1.01E-06	1.35E-05
PARG15803	-1.439332459	2.718165454	1.01E-06	1.36E-05
PARG25188	-1.349905297	4.421460547	1.02E-06	1.36E-05
PARG05490	-1.353408067	4.019708957	1.02E-06	1.37E-05
PARG20908	-2.095778091	-0.825352713	1.03E-06	1.37E-05
PARG16038	1.995997129	2.042097083	1.04E-06	1.39E-05
PARG01475	2.447105896	0.683861319	1.04E-06	1.39E-05
PARG20596	-1.433835114	2.252306257	1.06E-06	1.41E-05
PARG23038	-1.428764339	4.609119998	1.06E-06	1.41E-05
PARG26855	-1.731810152	1.200467299	1.07E-06	1.42E-05
PARG09762	-1.326901002	4.740036724	1.07E-06	1.42E-05
PARG00668	-1.443589292	3.162110226	1.07E-06	1.42E-05
PARG26344	1.379525734	2.981523704	1.08E-06	1.43E-05
PARG19713	1.592202515	2.078538104	1.10E-06	1.45E-05
PARG06350	1.329141429	4.750428132	1.11E-06	1.47E-05
PARG26512	-1.998773721	-0.292106963	1.11E-06	1.48E-05
PARG09015	-1.320863465	6.207442286	1.12E-06	1.48E-05
PARG02977	-1.343717041	3.761342839	1.12E-06	1.49E-05
PARG08670	-2.594776118	0.42611782	1.13E-06	1.49E-05
PARG15337	1.379055603	2.92435738	1.14E-06	1.50E-05
PARG07104	-1.310568408	6.442376653	1.15E-06	1.52E-05
PARG18911	-1.437275582	2.056320218	1.15E-06	1.52E-05
PARG26666	-1.576605611	2.287151371	1.16E-06	1.52E-05
PARG04293	1.553249795	2.855977171	1.16E-06	1.53E-05

PARG07355	-1.382518849	3.29120111	1.17E-06	1.53E-05
PARG23576	1.313896826	6.229883768	1.17E-06	1.53E-05
PARG10216	1.312360992	8.035148755	1.17E-06	1.53E-05
PARG11403	-1.318182881	5.464868409	1.17E-06	1.53E-05
PARG00217	2.203079892	0.294106771	1.18E-06	1.55E-05
PARG28288	1.30363647	9.747171006	1.20E-06	1.57E-05
PARG26896	-1.567610835	2.383318079	1.21E-06	1.59E-05
PARG23025	1.335720797	5.849478903	1.24E-06	1.63E-05
PARG24594	-1.642315061	3.416659566	1.25E-06	1.63E-05
PARG28029	-1.3566853	3.327969852	1.25E-06	1.63E-05
PARG14062	-1.455346906	1.799831587	1.26E-06	1.64E-05
PARG29953	-1.923317882	-0.026567559	1.26E-06	1.64E-05
PARG06291	-1.305656445	7.051417733	1.26E-06	1.65E-05
PARG02430	-1.547568554	1.289645331	1.27E-06	1.65E-05
PARG12068	-1.695510906	1.737095421	1.30E-06	1.69E-05
PARG00563	-1.351345983	4.435595602	1.32E-06	1.71E-05
PARG29208	1.493427233	4.369759296	1.33E-06	1.73E-05
PARG11967	1.344005332	4.132460813	1.33E-06	1.73E-05
PARG07913	-1.461985483	2.426016415	1.34E-06	1.74E-05
PARG14292	-1.304172345	6.725834115	1.36E-06	1.76E-05
PARG12574	1.299416467	7.545200297	1.37E-06	1.77E-05
PARG06079	1.301355713	7.051565317	1.39E-06	1.80E-05
PARG07759	1.30650499	5.906533545	1.40E-06	1.80E-05
PARG26431	-1.572359295	2.872187525	1.41E-06	1.82E-05
PARG00087	-1.295521533	8.341367484	1.41E-06	1.82E-05
PARG17969	5.972145056	3.018838557	1.42E-06	1.83E-05
PARG15618	-1.70674225	-0.042068914	1.46E-06	1.87E-05
PARG12441	-1.705880767	2.006995125	1.46E-06	1.87E-05
PARG14991	-2.330337708	-0.283805134	1.46E-06	1.87E-05
PARG11915	1.817639368	7.223375872	1.48E-06	1.90E-05
PARG04167	1.337769456	3.943740968	1.49E-06	1.91E-05
PARG27545	-1.385701085	2.38885577	1.51E-06	1.94E-05
PARG06245	1.307810513	6.426368121	1.52E-06	1.95E-05
PARG03262	-1.358459807	2.832655116	1.54E-06	1.97E-05
PARG06521	-1.335610717	4.403189518	1.57E-06	2.00E-05
PARG24006	-1.3003812	5.419777111	1.58E-06	2.01E-05
PARG13718	-1.691334847	0.329471832	1.58E-06	2.02E-05
PARG12354	-1.376695687	3.402600747	1.59E-06	2.03E-05
PARG09463	-2.07827963	2.694560162	1.61E-06	2.05E-05
PARG21107	-1.377312202	3.036899121	1.62E-06	2.06E-05
PARG10888	3.33731109	4.509890828	1.62E-06	2.07E-05
PARG27158	1.307655582	5.720111277	1.63E-06	2.07E-05
PARG10337	1.34532673	4.801237517	1.65E-06	2.10E-05
PARG09184	-1.914164248	0.253537998	1.67E-06	2.11E-05
PARG05426	-1.30489096	4.887605923	1.68E-06	2.13E-05
PARG19307	-1.292427985	5.903198804	1.68E-06	2.13E-05

PARG10577	-1.306666568	5.041194689	1.70E-06	2.16E-05
PARG25390	-1.291264645	5.946425108	1.71E-06	2.16E-05
PARG13690	-1.295105654	5.962498157	1.71E-06	2.16E-05
PARG05563	-1.805637433	1.925765889	1.72E-06	2.17E-05
PARG24522	1.316944938	6.284952424	1.75E-06	2.20E-05
PARG15732	1.334816816	4.536232312	1.77E-06	2.23E-05
PARG18026	-1.282532245	8.70631205	1.77E-06	2.23E-05
PARG23407	1.282325467	8.859156923	1.79E-06	2.25E-05
PARG00833	-1.363542585	3.449873743	1.80E-06	2.26E-05
PARG10210	1.311758128	6.04070632	1.80E-06	2.27E-05
PARG25633	-1.646478647	0.359975882	1.81E-06	2.28E-05
PARG00969	-1.330260639	5.048874149	1.82E-06	2.28E-05
PARG18451	-1.287915235	6.317650533	1.82E-06	2.28E-05
PARG23764	1.32027058	4.186650819	1.87E-06	2.34E-05
PARG23951	1.303047514	4.430901827	1.88E-06	2.36E-05
PARG02908	-1.480607931	1.653056602	1.89E-06	2.36E-05
PARG16104	-1.2834155	8.641151885	1.91E-06	2.39E-05
PARG21597	-1.365920552	3.263966609	1.93E-06	2.41E-05
PARG13833	-1.308071388	4.636037125	1.94E-06	2.42E-05
PARG00597	-1.654273512	2.946272287	1.94E-06	2.42E-05
PARG28341	1.673825374	7.91436219	1.95E-06	2.42E-05
PARG12258	2.458072931	1.197720115	1.98E-06	2.46E-05
PARG04524	-1.421624212	2.172929086	1.98E-06	2.46E-05
PARG19839	4.294974619	3.465362209	1.99E-06	2.48E-05
PARG15857	-1.279085062	8.409528786	2.00E-06	2.48E-05
PARG14286	1.279159277	8.680396848	2.03E-06	2.51E-05
PARG27763	-1.741835421	0.811728237	2.07E-06	2.56E-05
PARG26369	-1.315629004	3.054133969	2.09E-06	2.59E-05
PARG09013	-1.32025889	7.099883102	2.10E-06	2.60E-05
PARG26535	-1.463019657	1.558447756	2.11E-06	2.61E-05
PARG25047	-1.410445804	1.928509116	2.13E-06	2.63E-05
PARG18849	1.427059729	5.345647396	2.13E-06	2.64E-05
PARG19768	1.285376733	6.292245646	2.14E-06	2.64E-05
PARG00368	-2.565223119	-0.729401152	2.16E-06	2.66E-05
PARG07974	1.295967647	4.844946659	2.17E-06	2.68E-05
PARG06760	-1.569949072	1.750035947	2.17E-06	2.68E-05
PARG20716	-1.521237884	1.460967471	2.19E-06	2.69E-05
PARG10064	1.27199804	8.411019503	2.21E-06	2.72E-05
PARG10955	2.5386982	2.474755173	2.22E-06	2.73E-05
PARG27263	-2.739870321	0.13897947	2.24E-06	2.76E-05
PARG27503	-1.668504337	0.331260054	2.25E-06	2.76E-05
PARG08131	1.48979479	1.871205768	2.26E-06	2.77E-05
PARG27904	-1.387923829	2.325520036	2.28E-06	2.79E-05
PARG27420	-1.304630393	4.012790367	2.28E-06	2.79E-05
PARG23424	-1.274280931	6.600164939	2.29E-06	2.80E-05
PARG08607	-1.291452931	3.853323602	2.33E-06	2.84E-05

PARG00497	-2.206888567	-0.811248588	2.34E-06	2.85E-05
PARG18382	-1.295135995	5.932420614	2.41E-06	2.94E-05
PARG23191	-2.024941943	-0.191159016	2.41E-06	2.94E-05
PARG11283	-1.803290759	0.003662284	2.41E-06	2.94E-05
PARG15815	-1.306000654	3.630568447	2.44E-06	2.97E-05
PARG15643	-1.715627692	0.233511397	2.44E-06	2.97E-05
PARG07251	1.276520537	8.441186226	2.46E-06	2.99E-05
PARG05066	-1.304030349	4.172085709	2.50E-06	3.03E-05
PARG10354	3.30811759	-0.71179949	2.51E-06	3.04E-05
PARG17910	2.391311747	0.261232107	2.51E-06	3.04E-05
PARG26897	-1.309631949	3.13853811	2.53E-06	3.06E-05
PARG25647	-1.263030853	8.278113323	2.54E-06	3.08E-05
PARG02727	1.317201548	8.717336886	2.56E-06	3.09E-05
PARG12128	-1.27599216	5.236736497	2.56E-06	3.10E-05
PARG13433	-1.872519722	0.085287895	2.56E-06	3.10E-05
PARG05484	-1.337975403	3.668173428	2.58E-06	3.12E-05
PARG01286	1.27544794	6.214682993	2.60E-06	3.14E-05
PARG22368	-1.715765964	2.256853966	2.64E-06	3.17E-05
PARG17053	-1.705055129	0.493413952	2.65E-06	3.19E-05
PARG04195	-1.269916629	4.599632293	2.66E-06	3.19E-05
PARG04445	-1.39563192	1.580972701	2.67E-06	3.21E-05
PARG29279	-1.341246144	3.607637874	2.70E-06	3.24E-05
PARG15444	1.262292265	8.654300941	2.71E-06	3.25E-05
PARG20169	-1.264679462	6.243013919	2.74E-06	3.29E-05
PARG19200	-1.458674435	3.197228076	2.75E-06	3.30E-05
PARG12667	-1.291057812	4.204331668	2.76E-06	3.31E-05
PARG08277	-1.309432016	4.636065086	2.78E-06	3.32E-05
PARG19646	-1.271082992	6.168121517	2.82E-06	3.38E-05
PARG03526	-1.283997793	4.118111659	2.84E-06	3.40E-05
PARG19091	1.311685195	3.86374875	2.92E-06	3.49E-05
PARG20212	-1.352458643	1.930684344	2.93E-06	3.49E-05
PARG00958	-1.289530484	4.196690895	2.94E-06	3.50E-05
PARG15235	-1.27222236	5.998852192	2.94E-06	3.50E-05
PARG12747	-1.260111713	6.315960113	2.96E-06	3.52E-05
PARG04576	-1.637846735	0.688223201	2.97E-06	3.54E-05
PARG25878	2.428137616	1.708767293	2.99E-06	3.55E-05
PARG28426	1.476845983	2.007920794	3.00E-06	3.55E-05
PARG23918	-1.352718304	1.761328869	3.00E-06	3.55E-05
PARG04075	-1.607807959	0.614003509	3.03E-06	3.60E-05
PARG15677	-1.681537534	0.300163246	3.08E-06	3.64E-05
PARG23112	1.31836794	5.016172035	3.11E-06	3.68E-05
PARG20108	-2.178910467	1.077603114	3.11E-06	3.68E-05
PARG09818	1.311732259	4.289313694	3.11E-06	3.68E-05
PARG13327	-1.38080289	3.026714527	3.12E-06	3.68E-05
PARG15121	-1.319400035	4.11541542	3.14E-06	3.71E-05
PARG06401	1.407589143	3.172843945	3.15E-06	3.71E-05

PARG05891	-1.77182445	2.143620605	3.15E-06	3.72E-05
PARG27966	1.39770239	2.984805841	3.18E-06	3.75E-05
PARG15191	1.27716969	6.639673867	3.20E-06	3.76E-05
PARG07982	-1.278129765	3.929741282	3.21E-06	3.78E-05
PARG25631	-4.283482386	1.096433088	3.29E-06	3.86E-05
PARG14234	-1.313446288	3.066980098	3.29E-06	3.87E-05
PARG24685	-1.376592403	2.410364129	3.32E-06	3.90E-05
PARG21864	1.593143714	0.827918653	3.33E-06	3.90E-05
PARG11347	1.253901556	9.475010229	3.38E-06	3.95E-05
PARG04040	-1.34440857	2.563948569	3.44E-06	4.03E-05
PARG10932	-1.30403363	3.377711026	3.46E-06	4.04E-05
PARG07854	-1.266860338	3.881042266	3.49E-06	4.07E-05
PARG12520	1.254359265	6.92392521	3.52E-06	4.11E-05
PARG07990	1.287198373	4.193266493	3.59E-06	4.19E-05
PARG18324	1.979817478	-0.378679244	3.65E-06	4.26E-05
PARG08861	-1.243391872	7.022219791	3.77E-06	4.39E-05
PARG03544	-1.862782939	1.138848689	3.78E-06	4.40E-05
PARG03504	-1.248963984	5.397330472	3.78E-06	4.40E-05
PARG20517	-1.360658781	2.090726249	3.78E-06	4.40E-05
PARG11501	-1.248938965	6.132982156	3.81E-06	4.42E-05
PARG21406	1.244401943	6.34294906	3.82E-06	4.43E-05
PARG11970	-1.39396661	1.73577138	3.84E-06	4.45E-05
PARG05911	-1.565415866	1.118271195	3.85E-06	4.46E-05
PARG25201	1.243618328	7.334440361	3.86E-06	4.47E-05
PARG25299	-1.382760869	1.827851034	3.88E-06	4.49E-05
PARG14978	-1.33560687	2.778926175	3.93E-06	4.54E-05
PARG26386	-1.241944486	6.218715263	3.95E-06	4.56E-05
PARG06239	1.431712962	1.76964929	3.99E-06	4.60E-05
PARG20555	1.260769792	4.861482259	4.02E-06	4.64E-05
PARG22949	-1.468411853	1.863491224	4.05E-06	4.67E-05
PARG00562	-1.262542748	5.413547576	4.15E-06	4.79E-05
PARG15377	1.251074653	4.982703791	4.16E-06	4.79E-05
PARG07015	-1.785873028	2.724250185	4.17E-06	4.79E-05
PARG08838	-1.878949873	3.116340262	4.17E-06	4.80E-05
PARG04529	1.268006379	3.435494494	4.20E-06	4.83E-05
PARG20904	1.270896503	4.237760677	4.25E-06	4.88E-05
PARG26143	-1.356467548	3.701314729	4.25E-06	4.88E-05
PARG12299	1.235810679	7.377286201	4.25E-06	4.88E-05
PARG00436	1.438585747	2.817989646	4.26E-06	4.88E-05
PARG13088	-1.264896075	3.759731034	4.30E-06	4.92E-05
PARG27801	-1.271068805	4.389644001	4.32E-06	4.94E-05
PARG21796	1.240524919	7.924075106	4.36E-06	4.99E-05
PARG05778	-1.233112475	7.122068845	4.41E-06	5.04E-05
PARG13750	-1.28737808	4.132446429	4.42E-06	5.05E-05
PARG26820	-1.960315007	1.468452408	4.47E-06	5.10E-05
PARG11340	-1.953796967	-0.666431697	4.47E-06	5.10E-05

PARG14392	1.297344658	3.472990206	4.58E-06	5.22E-05
PARG29923	-1.419555281	1.481940937	4.68E-06	5.33E-05
PARG11442	-2.062521965	-0.413783658	4.78E-06	5.44E-05
PARG19453	-1.344524533	4.196866241	4.80E-06	5.46E-05
PARG17941	1.234807283	6.687768845	4.87E-06	5.53E-05
PARG13952	-1.440377378	1.271231694	4.88E-06	5.54E-05
PARG09035	-2.173247197	-0.582243797	4.98E-06	5.65E-05
PARG29241	1.270080373	3.30026678	5.03E-06	5.70E-05
PARG00651	-1.366167287	1.873175736	5.03E-06	5.70E-05
PARG04299	1.256475082	5.070191494	5.08E-06	5.75E-05
PARG00022	-1.690307349	0.584976901	5.24E-06	5.92E-05
PARG12948	-1.285348512	3.387758816	5.38E-06	6.07E-05
PARG20943	-1.522653491	0.749299551	5.38E-06	6.07E-05
PARG28530	-1.381981882	1.171708193	5.40E-06	6.10E-05
PARG23339	-1.251893701	4.696763574	5.41E-06	6.10E-05
PARG26752	-1.228615352	5.650223982	5.46E-06	6.16E-05
PARG24262	-1.403905172	1.507434633	5.49E-06	6.18E-05
PARG10523	2.163880266	0.006736221	5.50E-06	6.18E-05
PARG00740	1.221786889	7.299185781	5.53E-06	6.21E-05
PARG20804	-1.549090587	3.327438369	5.54E-06	6.22E-05
PARG00334	1.237132016	5.518871172	5.54E-06	6.22E-05
PARG06840	-1.623997576	0.666816644	5.55E-06	6.22E-05
PARG01380	1.358116278	1.651113602	5.56E-06	6.24E-05
PARG04886	-1.508151938	1.267582964	5.68E-06	6.36E-05
PARG25161	-1.540826837	0.391148379	5.71E-06	6.39E-05
PARG22863	-1.896717026	0.445338492	5.72E-06	6.40E-05
PARG21396	1.306299718	3.032349467	5.74E-06	6.42E-05
PARG25129	-1.313379653	2.175050366	5.75E-06	6.42E-05
PARG05589	-1.40222819	1.534606489	5.75E-06	6.42E-05
PARG00454	-1.3045582	3.508626435	5.80E-06	6.47E-05
PARG27486	-1.222570643	5.699728603	5.86E-06	6.53E-05
PARG19356	1.214691162	8.364715322	5.96E-06	6.64E-05
PARG07761	-1.26243527	2.928925279	5.98E-06	6.66E-05
PARG06706	-1.230676989	5.100785747	6.00E-06	6.68E-05
PARG04574	-1.293215451	3.135261038	6.01E-06	6.68E-05
PARG17543	1.221393509	6.579233404	6.02E-06	6.68E-05
PARG02920	-1.521897208	0.653529039	6.04E-06	6.70E-05
PARG26811	-2.170186559	0.362003385	6.06E-06	6.73E-05
PARG08431	-1.230963881	4.85660415	6.08E-06	6.74E-05
PARG05199	-1.284717194	2.880623123	6.11E-06	6.77E-05
PARG24918	-1.230418327	5.574868313	6.14E-06	6.80E-05
PARG02310	1.314364133	4.156167945	6.19E-06	6.85E-05
PARG12214	-1.318575413	1.352304956	6.24E-06	6.89E-05
PARG00952	-1.643740121	2.100172598	6.26E-06	6.92E-05
PARG12783	-1.587036299	0.836344353	6.28E-06	6.93E-05
PARG20568	-1.275867597	2.933104569	6.31E-06	6.96E-05

PARG23846	-1.214399729	6.565365764	6.33E-06	6.98E-05
PARG07497	-1.402025132	2.252695541	6.34E-06	6.98E-05
PARG20872	-1.846653306	-0.242754658	6.35E-06	6.98E-05
PARG17710	1.413871975	5.836481046	6.35E-06	6.98E-05
PARG20697	-1.597244806	-0.135226624	6.38E-06	7.01E-05
PARG02245	1.685223531	0.328634746	6.46E-06	7.09E-05
PARG12440	-1.347751178	1.769744087	6.47E-06	7.10E-05
PARG10612	1.378956903	1.257742726	6.47E-06	7.10E-05
PARG21676	1.447407991	3.802504997	6.50E-06	7.13E-05
PARG25715	1.23184706	4.333193894	6.51E-06	7.13E-05
PARG05882	-1.752118599	1.766114897	6.55E-06	7.17E-05
PARG14745	-1.546079852	0.61151031	6.59E-06	7.21E-05
PARG27856	-1.365039268	1.817321178	6.70E-06	7.33E-05
PARG07396	-1.254603363	3.836027445	6.76E-06	7.38E-05
PARG03643	2.65700794	0.253292701	6.76E-06	7.38E-05
PARG00949	-1.206084503	8.309613706	6.82E-06	7.45E-05
PARG24030	-1.372134531	2.610187676	6.88E-06	7.50E-05
PARG23963	-1.426770604	1.733554029	6.91E-06	7.52E-05
PARG03635	-1.444849341	1.764944121	6.92E-06	7.53E-05
PARG15530	-1.212242972	5.799323947	6.92E-06	7.53E-05
PARG06362	-1.205797456	8.917564273	6.98E-06	7.59E-05
PARG12394	-1.569845771	0.633074461	7.07E-06	7.68E-05
PARG15549	-1.250262067	4.137777879	7.08E-06	7.68E-05
PARG19205	-1.225062389	5.188436182	7.14E-06	7.74E-05
PARG00658	1.25041343	3.74309245	7.26E-06	7.87E-05
PARG06246	1.215400513	6.396338576	7.36E-06	7.97E-05
PARG29284	-1.261778621	2.989021598	7.37E-06	7.98E-05
PARG19987	-1.432866357	1.791389487	7.38E-06	7.98E-05
PARG13331	1.235648121	4.884617336	7.51E-06	8.12E-05
PARG27095	1.202490458	6.526235312	7.55E-06	8.15E-05
PARG26641	1.200023121	8.230320877	7.58E-06	8.19E-05
PARG18460	-1.244195937	3.537708416	7.61E-06	8.21E-05
PARG19072	-1.213204627	4.641988651	7.69E-06	8.29E-05
PARG22713	1.435483798	1.593780954	7.79E-06	8.39E-05
PARG01141	1.199143308	8.170363592	7.79E-06	8.39E-05
PARG04423	-1.370098218	1.746788444	7.81E-06	8.40E-05
PARG08090	-2.05587388	1.314742134	7.95E-06	8.55E-05
PARG28581	-1.425518071	0.749171628	8.01E-06	8.61E-05
PARG16178	1.277665114	3.007863747	8.11E-06	8.71E-05
PARG03645	-1.229685708	4.566613657	8.16E-06	8.75E-05
PARG06951	-1.314173	2.779838979	8.26E-06	8.85E-05
PARG24202	-1.309737406	2.298165969	8.28E-06	8.87E-05
PARG23581	1.205515326	5.848259632	8.44E-06	9.04E-05
PARG06162	1.324968205	3.381113962	8.45E-06	9.05E-05
PARG11775	-1.204407333	4.63450306	8.52E-06	9.10E-05
PARG19900	-1.272695939	2.977362538	8.52E-06	9.10E-05

PARG15745	1.253167591	3.542835236	8.54E-06	9.12E-05
PARG00143	1.205929335	4.656032267	8.59E-06	9.16E-05
PARG20430	-1.205426371	4.936655127	8.65E-06	9.23E-05
PARG19417	-1.219642071	4.542144958	8.76E-06	9.33E-05
PARG21324	-1.270995691	3.705023318	8.86E-06	9.43E-05
PARG17627	-1.52514485	1.260551747	8.92E-06	9.49E-05
PARG15410	-1.206131538	5.024560672	8.98E-06	9.55E-05
PARG00279	1.211804101	5.487968116	9.02E-06	9.58E-05
PARG24762	-1.442635116	1.004416968	9.04E-06	9.60E-05
PARG18400	4.134628752	0.805505324	9.05E-06	9.61E-05
PARG19464	-1.196508267	6.770088687	9.18E-06	9.73E-05
PARG24635	-1.370366669	2.065760996	9.20E-06	9.75E-05
PARG03409	1.426106108	4.205965904	9.30E-06	9.85E-05
PARG28742	1.266729602	3.84322781	9.52E-06	0.000100796
PARG25346	1.199429912	6.259926721	9.59E-06	0.000101497
PARG11912	1.191235942	7.160683285	9.65E-06	0.000101993
PARG16191	-1.21474259	4.891400107	9.76E-06	0.000103136
PARG15231	-1.203824092	5.155728042	9.79E-06	0.000103393
PARG16100	-1.433863734	1.489783433	9.81E-06	0.000103532
PARG04361	-1.413042132	1.133451446	9.90E-06	0.000104372
PARG30394	-1.273171754	5.042736226	9.99E-06	0.000105302
PARG14063	-1.501581542	0.936470845	1.00E-05	0.000105696
PARG29414	2.387513367	1.740831047	1.01E-05	0.000105907
PARG19262	-1.506715966	0.962090016	1.01E-05	0.000106109
PARG07142	-1.189365308	6.120126411	1.01E-05	0.000106521
PARG19405	1.553201976	1.777553663	1.02E-05	0.000106701
PARG05658	-1.426237003	1.386200923	1.02E-05	0.000107006
PARG21808	1.182696024	7.922755978	1.02E-05	0.000107475
PARG07254	-1.971381699	0.094225271	1.03E-05	0.000107529
PARG02269	-1.379555723	2.295897953	1.03E-05	0.000108342
PARG22403	1.357858824	4.838533175	1.04E-05	0.000109014
PARG21622	-1.195566809	5.346655668	1.04E-05	0.000109116
PARG00008	-1.212498217	4.717016027	1.05E-05	0.000109282
PARG12819	-1.615975299	0.32649604	1.05E-05	0.000109698
PARG11823	1.237783052	3.547726012	1.05E-05	0.000109735
PARG04597	1.5596283	1.854092794	1.06E-05	0.000111014
PARG10988	-1.248472264	3.864003578	1.07E-05	0.000111179
PARG17887	-1.217349955	3.807672649	1.07E-05	0.000111928
PARG14157	-1.35098364	3.645522869	1.08E-05	0.000112151
PARG03289	-1.230980306	3.071782156	1.08E-05	0.000112861
PARG22255	1.185688218	7.077514118	1.09E-05	0.000113291
PARG19706	-1.218471436	5.114285805	1.09E-05	0.00011334
PARG06090	-1.190757773	4.936080184	1.09E-05	0.000113616
PARG17120	-1.739486095	-0.021907818	1.10E-05	0.000114237
PARG00948	-1.228526694	3.46815319	1.11E-05	0.000114926
PARG25310	-1.188197923	6.252574612	1.12E-05	0.000115772

PARG02257	1.212130721	4.405064991	1.12E-05	0.000116046
PARG10753	-1.843873085	-0.515223733	1.12E-05	0.000116046
PARG21081	-1.226716005	3.451104064	1.12E-05	0.000116301
PARG02504	1.26965926	2.752254776	1.13E-05	0.000116304
PARG03840	1.204984658	4.937792366	1.13E-05	0.000116993
PARG07409	-1.177608	7.079841328	1.13E-05	0.000116993
PARG19423	-1.302187584	1.958491115	1.14E-05	0.000117354
PARG26704	-1.197055022	4.36582098	1.14E-05	0.000117661
PARG12480	1.225841236	4.866533758	1.14E-05	0.000117661
PARG14180	1.184898213	5.660416705	1.16E-05	0.000119363
PARG26931	-1.91765996	0.015976944	1.16E-05	0.000119607
PARG03185	-1.188839735	5.141827349	1.18E-05	0.000121753
PARG01158	-1.257195583	2.429886988	1.20E-05	0.000123367
PARG06928	1.207244022	5.258659331	1.21E-05	0.000124007
PARG12217	1.188517824	5.85172449	1.21E-05	0.000124283
PARG08515	-1.633392915	2.000972854	1.24E-05	0.000126665
PARG02417	-1.736668733	0.122536035	1.24E-05	0.000127095
PARG18432	-1.187826013	5.135564295	1.25E-05	0.000127672
PARG07942	-1.193084663	4.936582227	1.25E-05	0.000127783
PARG23873	-1.855865636	-0.396971495	1.27E-05	0.000130239
PARG07751	1.178080815	7.153235663	1.27E-05	0.000130269
PARG08115	-1.491277088	0.6889273	1.29E-05	0.000131901
PARG09222	1.948786937	0.418361879	1.30E-05	0.000133184
PARG29179	-1.188562649	4.356565219	1.31E-05	0.000133885
PARG24073	-5.733551801	-0.830769858	1.32E-05	0.000133975
PARG26343	-4.467097773	-0.585811103	1.32E-05	0.000133975
PARG26419	-4.467097773	-0.585811103	1.32E-05	0.000133975
PARG20468	-1.215775333	3.820131848	1.32E-05	0.000134121
PARG19187	-1.201018524	5.264298682	1.32E-05	0.000134121
PARG11371	-1.188350724	8.899758252	1.32E-05	0.000134295
PARG15174	-1.178709178	5.73875904	1.32E-05	0.000134295
PARG27698	-1.206816434	4.157925105	1.32E-05	0.000134347
PARG06532	-1.323910163	1.74183158	1.33E-05	0.000134717
PARG17149	-2.569685844	-1.053932874	1.33E-05	0.000135033
PARG07901	-1.727404882	0.476741143	1.34E-05	0.00013533
PARG02807	-1.554469657	0.600909436	1.34E-05	0.000135707
PARG08362	1.181450166	6.650621278	1.34E-05	0.000135782
PARG18943	1.31232755	2.228222911	1.35E-05	0.000136136
PARG23868	-1.182543259	5.150289251	1.35E-05	0.000136665
PARG21538	-1.276182488	3.308486229	1.37E-05	0.0001381
PARG02953	-1.39512535	1.456995009	1.38E-05	0.000139538
PARG05848	-1.21333636	3.441758835	1.38E-05	0.000139575
PARG03362	-1.266601454	3.071528364	1.39E-05	0.000140116
PARG29493	1.246345947	6.54138001	1.40E-05	0.000140853
PARG12117	-1.559316374	0.439998692	1.42E-05	0.000142459
PARG03012	1.201204859	5.25746001	1.42E-05	0.000143123

PARG06910	-1.284182596	2.668140592	1.42E-05	0.000143123
PARG23461	1.192911676	3.583565211	1.43E-05	0.000143493
PARG12432	-1.501839468	0.353352364	1.43E-05	0.000143816
PARG12634	-1.408282359	0.932480733	1.44E-05	0.00014407
PARG25630	1.167517438	7.197440067	1.45E-05	0.000145804
PARG12311	1.172961853	5.602941072	1.46E-05	0.000145897
PARG20636	-1.378600932	1.64265532	1.49E-05	0.000148864
PARG01032	-1.168587834	7.342753394	1.49E-05	0.00014888
PARG20630	-1.162390835	6.631439541	1.49E-05	0.000149181
PARG07631	-1.199653847	5.059984754	1.50E-05	0.000149648
PARG04387	1.184928981	7.077798793	1.50E-05	0.000149648
PARG21579	-1.185568105	3.65352095	1.53E-05	0.000153156
PARG08023	1.247394158	3.520471416	1.56E-05	0.000155435
PARG06080	-1.22011068	3.084383374	1.57E-05	0.00015666
PARG02841	-1.368562237	1.562713687	1.59E-05	0.000157881
PARG00612	1.167307027	6.018305806	1.60E-05	0.000159517
PARG08334	-1.286057656	1.622125013	1.63E-05	0.000161929
PARG30350	1.458743336	1.218149598	1.63E-05	0.000162123
PARG07611	1.155911717	7.189799205	1.65E-05	0.000164165
PARG07165	-1.234642577	2.73671264	1.65E-05	0.000164289
PARG17734	1.154261119	9.207364586	1.67E-05	0.00016538
PARG02665	-1.193568463	3.940827003	1.67E-05	0.00016538
PARG26846	-1.205776358	3.962738788	1.67E-05	0.00016538
PARG23208	-1.172750234	4.934349661	1.67E-05	0.00016538
PARG04495	-1.174987654	5.007281191	1.67E-05	0.000165675
PARG06740	1.185346909	4.55346048	1.68E-05	0.000166649
PARG10986	-1.193806815	3.156895156	1.69E-05	0.000167013
PARG22334	-2.444059543	-0.272223973	1.73E-05	0.000170402
PARG24090	2.364124367	-0.752767941	1.73E-05	0.000170402
PARG25211	-2.138731148	0.312579932	1.73E-05	0.000170534
PARG29762	-1.152915412	6.758164943	1.73E-05	0.000170836
PARG01306	-2.071850117	0.016190122	1.73E-05	0.000170947
PARG02925	1.163549661	6.233802883	1.74E-05	0.000171277
PARG01809	1.481563777	1.106178451	1.76E-05	0.000173638
PARG01046	-1.168162494	6.62931584	1.77E-05	0.000173638
PARG00972	-1.151819238	6.733578535	1.78E-05	0.000174627
PARG19055	-1.306706704	3.656820119	1.78E-05	0.000175139
PARG29153	1.150078504	7.879842616	1.79E-05	0.000175594
PARG26621	-1.266539701	1.179051789	1.79E-05	0.000175777
PARG10755	-1.599071401	0.21543377	1.80E-05	0.000176011
PARG16942	1.148399257	7.557910268	1.80E-05	0.000176011
PARG26857	-1.160119105	5.931784546	1.81E-05	0.000176847
PARG26975	-1.170206077	5.00255238	1.81E-05	0.000176847
PARG15289	1.173893497	5.144342896	1.82E-05	0.000178063
PARG14780	1.157945895	6.609810468	1.82E-05	0.000178279
PARG19753	-1.281290803	1.588004655	1.83E-05	0.000179026

PARG01041	-1.238724012	1.780759932	1.84E-05	0.000179999
PARG19000	-1.15658725	7.647234824	1.90E-05	0.000185004
PARG21863	-1.504987519	0.442274279	1.90E-05	0.000185741
PARG12422	1.158702075	5.144629054	1.92E-05	0.000186536
PARG08430	-1.248925313	2.438304472	1.92E-05	0.000186536
PARG07310	-1.436143819	0.783747861	1.93E-05	0.000188215
PARG01433	1.203905889	4.805893126	1.98E-05	0.000192337
PARG18624	1.151895172	5.169394741	1.98E-05	0.00019234
PARG06546	-2.180074384	-0.790616873	1.98E-05	0.00019234
PARG24297	-2.143292717	-0.689830962	1.98E-05	0.00019234
PARG08502	-1.14637343	6.539190074	1.99E-05	0.000193193
PARG02138	3.453659619	1.036546234	2.01E-05	0.000195208
PARG27940	1.233748658	3.107400401	2.04E-05	0.000197359
PARG13502	-1.265060843	1.797690425	2.04E-05	0.000197815
PARG00265	-1.237604097	2.914314911	2.05E-05	0.000198194
PARG18523	-1.150202982	5.037128352	2.05E-05	0.000198221
PARG12377	1.473251154	2.816746316	2.05E-05	0.000198257
PARG19328	1.239429975	5.0918006	2.05E-05	0.000198365
PARG10043	1.173951432	5.408374276	2.07E-05	0.000199583
PARG06033	1.154855775	5.397509709	2.07E-05	0.000199707
PARG06586	-2.649316703	1.451474988	2.08E-05	0.00020063
PARG22059	-1.676583449	2.243574187	2.10E-05	0.000202052
PARG12302	-1.426332376	0.782613394	2.10E-05	0.000202702
PARG27594	-1.147448333	5.658640999	2.11E-05	0.000202702
PARG13755	1.149111438	6.286394444	2.11E-05	0.000202702
PARG23595	-1.142223574	8.063690392	2.11E-05	0.000203275
PARG19285	-1.172780903	3.797989534	2.15E-05	0.000206924
PARG07789	-1.141483341	6.658719492	2.16E-05	0.000207671
PARG07299	1.146880509	6.32430409	2.17E-05	0.000208104
PARG26457	-1.335372393	1.091400591	2.17E-05	0.000208124
PARG27843	1.148616496	6.864789163	2.18E-05	0.000209394
PARG11385	1.243706211	3.46619774	2.20E-05	0.000210993
PARG09486	-1.907223734	-0.181145563	2.21E-05	0.000211095
PARG21271	1.304512926	1.592635471	2.22E-05	0.000212492
PARG21203	-1.136680472	7.214757209	2.24E-05	0.000214549
PARG24343	2.049932745	4.405325532	2.27E-05	0.000217144
PARG20164	-1.143612796	5.482875331	2.28E-05	0.000217244
PARG27578	-1.337068493	1.097272267	2.28E-05	0.000217244
PARG27518	-1.142775494	6.084297207	2.29E-05	0.000218679
PARG05908	-1.815226295	-0.506840689	2.33E-05	0.000221786
PARG16787	5.648054043	0.98938218	2.33E-05	0.000221786
PARG01526	1.224268266	5.554066516	2.34E-05	0.000223111
PARG26794	-1.135666585	6.390242836	2.35E-05	0.00022318
PARG25192	1.136632268	6.446915883	2.36E-05	0.000224363
PARG12824	-1.219223168	2.270103198	2.36E-05	0.000224363
PARG06267	1.183497088	5.177750495	2.37E-05	0.000224938

PARG07972	-1.16762656	6.257582483	2.40E-05	0.000227763
PARG14812	1.759186114	0.825833893	2.40E-05	0.000227914
PARG22450	2.344987895	1.874235576	2.44E-05	0.00023084
PARG11817	-1.143931263	5.180134471	2.45E-05	0.000232413
PARG01263	-1.204555879	3.038205868	2.50E-05	0.000237153
PARG14447	-4.07815011	-1.137186924	2.53E-05	0.000239199
PARG19395	-1.134017021	5.81040785	2.54E-05	0.00023994
PARG21041	-1.804699545	0.299208664	2.54E-05	0.000240178
PARG07927	-1.452129307	1.400647699	2.56E-05	0.000241406
PARG29440	1.381056354	2.79292058	2.56E-05	0.000241977
PARG27737	-1.141899937	4.733918841	2.57E-05	0.000241996
PARG27769	-1.195950906	2.231039948	2.57E-05	0.000241996
PARG26488	-1.197736719	5.419400446	2.62E-05	0.000247223
PARG19732	-1.411904139	0.622383363	2.64E-05	0.000248212
PARG27419	-1.179459584	3.090799168	2.64E-05	0.000248212
PARG22730	-1.314083507	2.454239797	2.69E-05	0.00025338
PARG21168	1.12262736	7.843187539	2.76E-05	0.000259626
PARG21934	-1.274986744	1.412608678	2.76E-05	0.000259626
PARG18383	-1.51559269	0.325691108	2.78E-05	0.000261248
PARG11423	1.124895152	6.917106338	2.82E-05	0.000264143
PARG00632	-1.126138598	6.584205904	2.82E-05	0.000264234
PARG24084	-1.130716762	5.900845316	2.85E-05	0.00026739
PARG25455	-1.484965736	0.76728183	2.88E-05	0.000269569
PARG24898	-1.597546731	0.303155162	2.88E-05	0.000269569
PARG08593	-1.529890208	0.114142644	2.90E-05	0.000271089
PARG13359	-1.128538308	5.53371246	2.90E-05	0.000271089
PARG07542	1.205711898	5.450570546	2.90E-05	0.000271111
PARG19631	-1.162516848	3.84253242	2.91E-05	0.000271976
PARG13525	-1.286103045	1.070495018	2.93E-05	0.00027363
PARG23479	-1.126089204	5.303946111	2.93E-05	0.000273732
PARG22869	1.152457826	4.759604982	2.96E-05	0.000275966
PARG09361	1.238929094	2.468644624	2.96E-05	0.000275966
PARG13910	-1.438500557	0.740530741	2.98E-05	0.000277532
PARG19404	-1.267647182	1.741907913	2.98E-05	0.000277722
PARG06867	-1.169241398	3.300251087	3.00E-05	0.000278636
PARG07524	-1.20260467	2.007667878	3.01E-05	0.000279661
PARG24179	-1.130860595	5.174030845	3.02E-05	0.000280147
PARG15190	1.13027366	6.36300896	3.02E-05	0.000280776
PARG07967	-1.181474536	4.954806014	3.07E-05	0.00028503
PARG24497	-1.122234765	6.713881697	3.08E-05	0.000285451
PARG15503	-1.412710066	1.146272481	3.08E-05	0.000285451
PARG03710	-1.205003048	2.245118727	3.08E-05	0.000285451
PARG16577	1.373998142	1.554286917	3.08E-05	0.000285528
PARG07957	-1.228229709	2.137397362	3.11E-05	0.000287416
PARG29145	-1.533695623	0.419855199	3.13E-05	0.000289499
PARG12285	-1.530774991	1.968194985	3.13E-05	0.000289499

PARG10582	-1.902832152	0.289682909	3.14E-05	0.00028977
PARG20658	-1.852562959	-0.520292895	3.14E-05	0.00028977
PARG19635	1.194391657	3.78599935	3.14E-05	0.00028977
PARG26377	-1.122169692	5.394514958	3.15E-05	0.000290516
PARG23740	1.133343866	5.435110791	3.18E-05	0.000292624
PARG13754	1.121015514	6.583555094	3.18E-05	0.000292624
PARG23560	-1.154283525	4.443222566	3.19E-05	0.000293975
PARG13133	1.118352363	7.626978705	3.24E-05	0.000297714
PARG08264	-1.184917123	3.010625397	3.27E-05	0.000300135
PARG01897	-1.180363299	3.099662315	3.27E-05	0.000300256
PARG00663	1.115699228	7.010813789	3.30E-05	0.000302613
PARG07853	-1.158005086	5.891381587	3.30E-05	0.000302613
PARG07650	1.184357932	4.517949943	3.30E-05	0.000303041
PARG27241	-1.11518186	6.581682035	3.33E-05	0.000305065
PARG18477	-1.673060813	-0.369576237	3.33E-05	0.000305065
PARG25736	-1.221057689	2.949827106	3.38E-05	0.000309043
PARG07117	1.111084117	7.34488193	3.39E-05	0.000309865
PARG04426	1.119090359	7.25392369	3.40E-05	0.000310868
PARG07047	-1.125938389	4.632348215	3.40E-05	0.000311038
PARG00075	-1.192427531	3.223924908	3.44E-05	0.000314044
PARG15209	-1.11824424	5.999691551	3.48E-05	0.000317687
PARG10751	-1.162403046	3.45223366	3.48E-05	0.000317687
PARG27499	1.112859073	7.754259502	3.52E-05	0.000320787
PARG25448	1.108440978	7.513407618	3.58E-05	0.000326593
PARG20655	-2.238050565	-0.735987537	3.59E-05	0.000327311
PARG24892	-1.13362832	5.272975002	3.61E-05	0.000328075
PARG20455	-1.323755241	3.080337418	3.62E-05	0.000329011
PARG30050	-1.403511612	0.651902204	3.63E-05	0.000330325
PARG30009	1.279773431	1.559730374	3.68E-05	0.000334738
PARG07813	-1.795388507	0.1270536	3.73E-05	0.000338906
PARG03487	-1.116735451	5.308207782	3.75E-05	0.000339841
PARG02830	-1.113956721	5.248245169	3.79E-05	0.00034356
PARG04518	1.169812156	3.687795868	3.82E-05	0.000346182
PARG15611	-1.454408398	-0.171726773	3.83E-05	0.000347112
PARG15637	-1.454408398	-0.171726773	3.83E-05	0.000347112
PARG24702	-1.333556564	1.16933045	3.84E-05	0.000347741
PARG11663	-1.108925123	5.873884657	3.86E-05	0.000349393
PARG23885	-1.187449207	1.789071315	3.87E-05	0.000349994
PARG26665	1.119219096	5.347170229	4.00E-05	0.000361085
PARG25444	1.403900558	2.975067219	4.01E-05	0.000361817
PARG27820	-1.238212579	2.688810909	4.05E-05	0.000364749
PARG01446	-1.104310599	6.015171734	4.05E-05	0.000364749
PARG08346	-1.448867427	0.650362248	4.05E-05	0.000364749
PARG26223	-1.566464434	0.16465868	4.05E-05	0.000364749
PARG18240	-1.104760732	6.763348849	4.10E-05	0.00036869
PARG07360	1.105288418	8.007678699	4.12E-05	0.000370268

PARG09413	5.523942387	0.749785001	4.14E-05	0.000372244
PARG17302	5.517762431	0.945494022	4.14E-05	0.000372244
PARG13003	-1.14350621	3.855124671	4.15E-05	0.000372677
PARG23512	-1.111606809	4.764096527	4.17E-05	0.000374643
PARG01333	-1.122248085	4.046780835	4.18E-05	0.000375361
PARG04584	1.097468293	7.374181856	4.19E-05	0.00037576
PARG17758	-1.131481859	3.263500387	4.22E-05	0.000378294
PARG16276	-1.093605977	10.40824656	4.25E-05	0.000380207
PARG10268	-1.156141743	2.657478141	4.26E-05	0.000381662
PARG02186	1.158347344	3.793965809	4.27E-05	0.000381808
PARG02209	-1.183494569	3.115954416	4.30E-05	0.000384397
PARG25422	-1.855354898	-0.91548619	4.32E-05	0.000386299
PARG03949	1.155244797	4.4166524	4.33E-05	0.00038644
PARG17675	1.132362384	4.631198923	4.34E-05	0.000386969
PARG07210	1.206684053	1.958243342	4.39E-05	0.000391946
PARG22833	1.104982578	5.841842327	4.41E-05	0.00039299
PARG15821	-1.187896814	2.206902081	4.45E-05	0.000396592
PARG02714	-1.094388034	6.897890452	4.48E-05	0.000398949
PARG00264	1.156997223	2.430837211	4.49E-05	0.00039993
PARG12382	-1.118428748	4.721824987	4.52E-05	0.000402319
PARG27474	-1.141361031	3.67928688	4.58E-05	0.000406738
PARG15525	-1.216382401	1.968268533	4.58E-05	0.000406738
PARG27663	1.10034908	5.747165319	4.62E-05	0.000409794
PARG15345	-1.094034237	7.419642489	4.63E-05	0.000410557
PARG20502	-1.448548058	0.613190988	4.65E-05	0.000411996
PARG27697	1.090549588	7.661733302	4.65E-05	0.000411996
PARG03698	1.360398097	1.866361959	4.71E-05	0.00041733
PARG07757	-1.12291416	5.409029516	4.71E-05	0.00041733
PARG28185	1.159403873	4.047911641	4.74E-05	0.000419267
PARG09190	-1.156616593	3.400504723	4.74E-05	0.000419267
PARG20293	1.115759151	4.694994778	4.76E-05	0.000420942
PARG13380	1.10758849	8.144457297	4.77E-05	0.000421221
PARG24566	-1.24408012	2.678339263	4.79E-05	0.000423305
PARG24712	-1.098068021	6.276652278	4.80E-05	0.00042334
PARG09147	-1.332939935	0.12928776	4.81E-05	0.000424057
PARG24846	-1.086499685	8.809242266	4.82E-05	0.000424801
PARG19348	-1.085769812	10.36663074	4.87E-05	0.000428658
PARG18956	1.463173409	0.785375667	4.88E-05	0.00042993
PARG10454	1.330851545	1.95090491	4.95E-05	0.000435395
PARG24100	1.0917941	6.168672478	5.00E-05	0.000439191
PARG00917	1.113277919	3.585172331	5.00E-05	0.000439191
PARG23707	1.093850334	9.282896958	5.01E-05	0.000439774
PARG24244	-1.305715575	1.517503201	5.02E-05	0.000440481
PARG00076	-1.110039579	4.09891424	5.04E-05	0.00044262
PARG00620	-1.097633693	7.05212646	5.08E-05	0.00044568
PARG14455	-1.965702995	-0.696336729	5.11E-05	0.000448017

PARG11474	-1.930171451	-0.832133399	5.11E-05	0.000448017
PARG11660	1.866481933	1.469125788	5.16E-05	0.000452046
PARG19347	1.627635711	7.607759947	5.18E-05	0.000453641
PARG15294	1.154767994	3.635914355	5.20E-05	0.000454978
PARG19505	-1.330380684	1.739127964	5.29E-05	0.000462468
PARG03639	1.158033301	2.922251531	5.29E-05	0.000462468
PARG30165	1.093494826	5.83548426	5.33E-05	0.000465296
PARG11773	-1.087442788	4.629658493	5.35E-05	0.000466575
PARG05139	1.085699931	7.091210171	5.37E-05	0.000468411
PARG12413	1.087677494	5.818090805	5.38E-05	0.000468896
PARG20270	-1.088006488	5.512859993	5.38E-05	0.000469037
PARG21235	-1.082836858	5.71475641	5.39E-05	0.00046966
PARG02081	-1.230463843	1.918948393	5.47E-05	0.00047618
PARG05196	1.514045858	0.876006325	5.49E-05	0.00047735
PARG22587	-1.151917316	2.665811335	5.49E-05	0.00047735
PARG07554	1.106685767	5.596434019	5.57E-05	0.000484004
PARG26555	-1.080954519	5.831188478	5.60E-05	0.000486499
PARG27489	1.086364284	6.444565646	5.66E-05	0.000491022
PARG11650	-1.131206141	2.80804502	5.70E-05	0.000494671
PARG09360	1.211567672	1.862848489	5.74E-05	0.000497435
PARG28239	-1.080679612	5.52702782	5.75E-05	0.000497895
PARG25425	1.15398881	5.214908377	5.80E-05	0.000502445
PARG24697	-1.403373794	-0.060007433	5.83E-05	0.000504417
PARG08257	-1.527800792	-0.212780654	5.89E-05	0.000509047
PARG17676	1.076922553	7.029789033	5.91E-05	0.00051127
PARG08880	1.08500785	6.238093397	5.94E-05	0.000513194
PARG18166	1.200356543	2.808736602	5.97E-05	0.000515227
PARG24683	1.073524259	7.82064234	6.07E-05	0.000523937
PARG07233	1.977784984	0.112020829	6.08E-05	0.000524557
PARG28095	1.078745193	7.309496913	6.08E-05	0.000524557
PARG14299	-1.357334877	1.037066611	6.19E-05	0.00053277
PARG14450	-1.357334877	1.037066611	6.19E-05	0.00053277
PARG02090	1.075921488	6.549164243	6.20E-05	0.000533592
PARG22444	1.077807547	6.01009161	6.21E-05	0.000533749
PARG07832	1.23503838	2.677517999	6.21E-05	0.000533763
PARG03750	1.073548429	7.60492008	6.24E-05	0.000536143
PARG18459	-1.184296443	2.090888767	6.26E-05	0.000537552
PARG24815	-1.103428184	3.806081487	6.27E-05	0.000537818
PARG22086	-1.084843893	5.34586719	6.28E-05	0.000538936
PARG22697	-1.10803676	3.860062968	6.29E-05	0.000538936
PARG13602	-1.145632024	1.889384682	6.30E-05	0.000540221
PARG22213	-1.14482334	3.358832437	6.35E-05	0.000543516
PARG05821	1.102947551	3.822363145	6.35E-05	0.000543516
PARG00731	1.070924696	7.585559858	6.39E-05	0.000546523
PARG15325	-1.282903886	0.954106802	6.48E-05	0.000553874
PARG27735	1.177370713	2.898713139	6.48E-05	0.000553874

PARG21984	-1.078873456	6.036389188	6.50E-05	0.000554816
PARG06193	1.608695251	0.197756097	6.55E-05	0.00055939
PARG03511	-1.07998638	4.31070308	6.56E-05	0.000559633
PARG22471	-1.070895913	6.322375407	6.58E-05	0.000561311
PARG24753	-1.860358286	-0.594276728	6.62E-05	0.000563936
PARG27224	1.072232469	6.336220446	6.62E-05	0.000563936
PARG24570	-1.156467972	2.015136156	6.67E-05	0.000567577
PARG12666	-1.509493771	0.193739841	6.75E-05	0.000574441
PARG07266	1.136405138	4.101873993	6.81E-05	0.000579399
PARG14656	-1.130436116	3.066268205	6.84E-05	0.000580995
PARG12503	2.223009866	0.507528065	6.86E-05	0.000582419
PARG07677	-1.332214195	1.09729709	6.86E-05	0.000582419
PARG25611	-1.413078511	0.376514736	6.89E-05	0.000584356
PARG05515	1.07613417	7.368646396	6.92E-05	0.000586527
PARG01880	1.075514896	6.060074342	6.92E-05	0.000586527
PARG25737	-1.075438206	5.426580771	6.92E-05	0.00058663
PARG08578	1.2745922	2.111582484	6.94E-05	0.000587596
PARG29805	-1.074987359	10.30760297	6.94E-05	0.000587596
PARG14926	-1.131025134	2.690898276	6.99E-05	0.000591215
PARG07562	1.074409996	5.597292033	7.01E-05	0.000592828
PARG26644	-1.110641946	4.280864786	7.04E-05	0.000595109
PARG01594	-1.128223775	4.228334286	7.05E-05	0.000595217
PARG15464	-1.073003999	7.444636179	7.15E-05	0.000603041
PARG00579	1.276599034	1.930068408	7.22E-05	0.000609163
PARG07452	-1.067454139	5.630375441	7.24E-05	0.000610301
PARG07416	-1.099730386	3.784613887	7.25E-05	0.000610568
PARG23583	1.063071493	7.277970133	7.26E-05	0.000611729
PARG01573	-1.997922582	0.236131697	7.28E-05	0.000612562
PARG04102	-1.063181018	7.25197882	7.28E-05	0.00061263
PARG18427	-1.208698713	2.968606934	7.29E-05	0.000613056
PARG03677	1.059297127	8.638795917	7.38E-05	0.000620492
PARG14420	-1.063231549	6.72189438	7.46E-05	0.000626743
PARG19074	-1.077552909	6.766837901	7.51E-05	0.000630712
PARG20490	-1.14097319	3.677478245	7.57E-05	0.000634901
PARG00588	-1.12145528	3.724243223	7.59E-05	0.000636645
PARG24149	-1.377064218	-0.1080843	7.70E-05	0.000645438
PARG08293	-1.087292183	4.594092101	7.75E-05	0.000649508
PARG08259	1.153811791	6.536433078	7.78E-05	0.000651229
PARG07164	-1.301478659	1.041880436	7.82E-05	0.000654675
PARG07169	1.097487795	4.324050125	7.96E-05	0.000665375
PARG26601	-1.395528921	0.076677822	8.07E-05	0.00067429
PARG13734	-2.722603734	-0.306775465	8.10E-05	0.000676564
PARG07852	1.056169098	7.191382749	8.15E-05	0.000680739
PARG22734	1.07090104	4.311874932	8.16E-05	0.000680739
PARG19865	-1.081962963	4.208029504	8.17E-05	0.00068112
PARG03546	-1.552258434	1.69777314	8.20E-05	0.000683793

PARG12782	-1.053714636	7.050052573	8.21E-05	0.000684467
PARG28295	-1.061220466	5.48197224	8.23E-05	0.000685622
PARG19887	-1.084382845	2.926556765	8.24E-05	0.000685622
PARG23834	-1.077247947	3.955206372	8.30E-05	0.000690208
PARG02123	-1.356419794	0.729159097	8.31E-05	0.000691205
PARG18910	1.050341431	9.360637031	8.39E-05	0.000697335
PARG15573	-1.063735281	4.922628323	8.40E-05	0.000697385
PARG01385	-1.343341003	1.075498092	8.43E-05	0.000699706
PARG00379	1.064213892	3.947440625	8.44E-05	0.000700381
PARG18195	-1.19309251	2.136054108	8.49E-05	0.000703797
PARG23927	-1.083165187	4.797316227	8.51E-05	0.000704966
PARG21275	-1.166377783	2.215098162	8.58E-05	0.000710634
PARG27344	-1.382739666	2.236157978	8.63E-05	0.000714401
PARG00042	-1.06396437	5.954033453	8.81E-05	0.000729015
PARG03477	-1.24721651	0.625251174	8.94E-05	0.000739063
PARG11578	-1.072618347	5.247249845	8.97E-05	0.000741212
PARG10556	1.107849908	5.517692902	9.11E-05	0.000752312
PARG08357	-1.307064373	2.609197989	9.18E-05	0.000758019
PARG07103	1.220106502	3.028781012	9.21E-05	0.000759734
PARG12986	-1.053814328	5.032523941	9.34E-05	0.0007704
PARG16090	-1.061389779	4.827843021	9.34E-05	0.0007704
PARG12297	-1.498305752	0.607575213	9.49E-05	0.000781846
PARG16351	1.111707037	4.657020056	9.54E-05	0.000786093
PARG06210	-1.048554922	5.888068183	9.56E-05	0.000786677
PARG19551	1.070546839	4.77108812	9.57E-05	0.000786982
PARG00666	1.137319547	4.309516226	9.58E-05	0.00078802
PARG07580	-1.061073508	4.103492263	9.60E-05	0.00078894
PARG15720	1.052865116	6.460612809	9.60E-05	0.00078894
PARG16653	-1.304995743	0.984941922	9.66E-05	0.000792964
PARG26716	-1.092883542	2.673223253	9.67E-05	0.000793705
PARG20104	1.061713762	4.888051356	9.73E-05	0.000797925
PARG21351	-1.057667027	4.430347964	9.73E-05	0.000797925
PARG26914	1.070065445	4.639807128	9.82E-05	0.000804287
PARG08132	-1.078438361	3.434751264	9.84E-05	0.000805495
PARG01329	1.378363416	0.88935818	9.97E-05	0.000815793
PARG27980	-1.042594834	6.349339303	0.000100088	0.000818748
PARG06920	-1.103158245	3.22134715	0.000100226	0.000819453
PARG01916	-1.08483784	3.257112021	0.000100996	0.000825314
PARG22806	1.081466826	4.373507784	0.000101709	0.000830716
PARG24019	1.835605809	6.251846757	0.000102071	0.000832802
PARG01691	1.789608751	-0.151186458	0.000102071	0.000832802
PARG22270	-1.036585498	10.62900828	0.000102284	0.000834104
PARG11287	-1.276497991	1.334862898	0.000102582	0.000836103
PARG06748	1.275755676	3.684664679	0.000103378	0.000842157
PARG13605	-1.047990973	5.48683421	0.000104082	0.000847447
PARG24393	-1.045005266	5.630702885	0.000104219	0.000848123

PARG03067	-1.070465988	3.950573195	0.000104322	0.000848523
PARG00616	1.057612555	6.106174931	0.000104866	0.000852502
PARG18549	-1.135122365	1.870600946	0.000105358	0.000856059
PARG11160	-1.483193231	1.478504187	0.000105921	0.000859745
PARG20494	-1.450485515	0.772571637	0.000105921	0.000859745
PARG08724	1.079875765	4.059254544	0.000106382	0.000863041
PARG18279	-1.503820737	3.019672664	0.000107276	0.000869841
PARG25040	-1.040147229	6.408006482	0.00010751	0.00087129
PARG06948	1.428070998	0.850815153	0.000107929	0.000874232
PARG03957	2.790078066	1.292233318	0.000109008	0.000882523
PARG15043	-1.097599698	2.773933655	0.000109623	0.000887042
PARG08440	-1.05829464	6.318213685	0.000110085	0.000890326
PARG07872	-1.145813948	1.852378968	0.000111024	0.000897451
PARG03418	-1.037849267	6.133090998	0.000111261	0.000898904
PARG11470	1.186825894	0.614858443	0.000111411	0.000899655
PARG24002	-1.355270255	-0.176917498	0.00011203	0.000904186
PARG22064	-1.030901494	11.12005746	0.000112229	0.000905326
PARG16160	-1.062707057	3.720662291	0.000114401	0.00092208
PARG24066	1.030479359	8.998386352	0.000114423	0.00092208
PARG18509	-1.301856245	1.596091017	0.000114579	0.000922867
PARG07098	1.708045008	-0.740144889	0.000115283	0.000927805
PARG27251	-1.088077545	4.290516812	0.000115311	0.000927805
PARG17942	1.041112815	6.337815655	0.000116797	0.000939281
PARG26514	-1.216195974	0.877405351	0.000118341	0.000951215
PARG07935	-1.039798318	5.418259845	0.000118693	0.000953551
PARG00200	-1.055052267	4.087466896	0.000119176	0.000956941
PARG27302	-1.036350358	5.440926645	0.0001193	0.000957449
PARG22317	1.210038643	2.725091835	0.000119391	0.000957693
PARG18568	-1.066270163	3.863478539	0.000119558	0.000958537
PARG06119	-1.361712101	0.345229795	0.000120326	0.000964204
PARG09733	3.74385176	-0.441137081	0.000121093	0.000969854
PARG21224	-1.037578465	5.800463012	0.000121551	0.000973031
PARG07845	1.033853682	6.873923342	0.000122804	0.000982554
PARG00714	1.143674662	2.305716767	0.000123142	0.00098476
PARG12786	1.077407661	3.523280237	0.000123372	0.000985091
PARG14331	-1.108078273	4.031804599	0.000123478	0.000985091
PARG22034	2.669240634	2.146817559	0.000123497	0.000985091
PARG02478	2.662513253	4.69308581	0.000123497	0.000985091
PARG03620	2.659379196	1.026378809	0.000123497	0.000985091
PARG19461	-1.589036512	4.503551283	0.000124567	0.000993117
PARG25403	-1.336321773	0.642262465	0.000124849	0.000994866
PARG20586	-1.494019032	0.975315673	0.000125044	0.000995432
PARG07767	-1.196700566	4.011273546	0.000125047	0.000995432
PARG07628	-1.04379734	5.099697841	0.000125407	0.000997793
PARG12974	1.131302928	2.464812364	0.000125577	0.000998637
PARG23261	1.026531243	8.467582551	0.000125961	0.001001184

PARG20017	-1.039742403	4.309961999	0.000126875	0.001007938
PARG28078	-1.060496482	3.755538385	0.00012768	0.001013817
PARG15930	1.100208258	3.321004119	0.000128505	0.001019851
PARG01742	-1.335990503	1.037831117	0.000128964	0.001022978
PARG11571	1.052394089	5.20366867	0.000129378	0.00102523
PARG12881	-1.867368445	-0.321728796	0.000129378	0.00102523
PARG27599	1.02135418	9.706554371	0.0001296	0.001026468
PARG17422	-1.270623372	0.485434411	0.000129831	0.00102778
PARG13942	1.094380921	2.3062654	0.000130487	0.001032456
PARG05509	-1.042004077	4.271435152	0.000130779	0.001034241
PARG09243	-1.031080044	5.60566197	0.000131457	0.001039078
PARG03532	1.811083528	-0.119383186	0.000131735	0.001040233
PARG11538	1.801085979	0.529199595	0.000131735	0.001040233
PARG27836	1.170343932	2.733812069	0.000131856	0.001040663
PARG20585	-1.062524783	3.580685784	0.000132188	0.001042757
PARG15752	-1.028993755	5.841566273	0.000132613	0.001045581
PARG11128	-1.404275305	2.80647947	0.000133384	0.001051134
PARG06365	1.034576906	6.006759107	0.000133598	0.00105229
PARG18110	-1.01932923	8.23317952	0.000134099	0.001055714
PARG19245	1.041550804	5.320315919	0.000134187	0.001055873
PARG06716	1.201161057	0.738359683	0.000134425	0.001057219
PARG06388	1.15244747	3.351906149	0.000135546	0.001065497
PARG20587	-1.53830478	1.417627362	0.000136289	0.001070807
PARG26736	-1.385627351	1.22193708	0.000136724	0.001073683
PARG00048	1.026057316	6.722353337	0.000136955	0.001074343
PARG00515	-1.249557561	1.01134133	0.000137013	0.001074343
PARG17703	2.168986332	1.373939799	0.000137013	0.001074343
PARG24822	-3.264873886	-0.349431678	0.000137243	0.001075074
PARG14980	3.169877621	0.172591241	0.000137243	0.001075074
PARG03301	-1.02210978	6.720702812	0.000137734	0.001078376
PARG05890	-1.038296634	6.500190524	0.000138116	0.001080833
PARG13515	-1.025464809	5.477660361	0.000138348	0.001082109
PARG12298	-1.201636928	1.656012218	0.000138431	0.001082215
PARG18374	-1.07575573	2.5265921	0.000139181	0.001087538
PARG06877	-1.166733479	1.998987483	0.000139383	0.001088577
PARG12332	-1.789148753	0.008096521	0.000139469	0.001088703
PARG08783	-1.106259518	3.622446226	0.000139617	0.001088898
PARG00646	1.026274344	6.155204005	0.000139636	0.001088898
PARG12776	1.030585607	5.103376646	0.000139702	0.001088898
PARG12613	-1.069361129	3.462670007	0.000139855	0.001089554
PARG26750	-1.113673825	2.435013039	0.000140834	0.001096634
PARG08908	-1.073106996	3.005007174	0.000141135	0.001098434
PARG17793	-1.071159024	2.98232457	0.000141633	0.00110176
PARG28099	-1.189846741	2.934485815	0.000143417	0.001114537
PARG17830	-1.043747537	4.405824828	0.000143417	0.001114537
PARG12281	-1.013593938	9.93089886	0.00014429	0.001120764

PARG08730	-1.276534067	1.105009143	0.000144687	0.001123291
PARG28358	1.023084747	5.499880447	0.000145256	0.001127156
PARG04121	1.019347147	6.407191956	0.000146784	0.001138443
PARG20894	-1.016491555	7.911782076	0.000147459	0.001143113
PARG13084	1.206010753	3.444800223	0.000150051	0.001162639
PARG10239	1.409024078	2.269658181	0.000150711	0.001167174
PARG19436	1.779984929	0.883382462	0.000150805	0.001167322
PARG06436	1.042412702	5.871254531	0.000151548	0.001172503
PARG13671	1.304124021	2.579204668	0.000152391	0.001178442
PARG12840	-1.573988641	-0.306201917	0.000152932	0.00118204
PARG10947	1.051259853	4.28941631	0.000153821	0.001188329
PARG06037	1.027291831	5.689115059	0.000154311	0.00119153
PARG11081	-1.052468194	3.991577735	0.000155081	0.001196687
PARG03142	1.022152818	5.89509723	0.000155132	0.001196687
PARG18895	1.012472831	7.444896083	0.000156569	0.001207182
PARG02921	-1.079116122	2.655488517	0.000156945	0.001209484
PARG21404	1.038374877	4.739574946	0.000157228	0.001211069
PARG18100	-2.15096932	-0.698021412	0.000157671	0.001213846
PARG01214	1.03875635	4.339651695	0.000157743	0.001213846
PARG18742	-1.215202123	2.153319309	0.00015784	0.001214002
PARG28315	-1.987092128	-0.700808398	0.000158702	0.001220028
PARG01347	1.274578071	2.297009156	0.000161958	0.001244456
PARG18501	-1.026072399	4.635910636	0.000162104	0.001244966
PARG08846	-1.266282054	0.846449587	0.000163345	0.001253273
PARG00103	-1.243889034	1.555565279	0.000163345	0.001253273
PARG25239	1.041816799	3.201581325	0.000163788	0.001256052
PARG20253	1.01077722	5.927147956	0.000164486	0.001260793
PARG13405	1.237004573	2.128536952	0.000165858	0.001270691
PARG08304	-1.615388757	-0.508231532	0.000166489	0.001274901
PARG22808	1.004705659	8.996366887	0.00016678	0.001276504
PARG12519	-1.039920062	4.431019247	0.000166918	0.001276941
PARG28605	1.023877771	4.897882709	0.000167	0.001276941
PARG01086	1.03807413	5.354741428	0.000170104	0.001300047
PARG27632	1.00407465	8.332722616	0.000170609	0.001303268
PARG08769	1.228335507	4.675093367	0.000170884	0.001304325
PARG25224	-1.008214264	5.701956799	0.000170913	0.001304325
PARG15926	-1.005354057	7.28542008	0.000171208	0.001305602
PARG25246	-1.384334583	0.285887738	0.00017133	0.001305602
PARG25343	-1.384334583	0.285887738	0.00017133	0.001305602
PARG03743	1.003390869	8.465073418	0.000172809	0.001316233
PARG17893	-1.134570904	1.778027231	0.000173364	0.001319819
PARG19468	1.12751111	3.858306363	0.000173466	0.001319954
PARG05640	-1.019539529	4.902884784	0.00017382	0.00132201
PARG08684	-1.119426036	2.416975975	0.000174702	0.001328071
PARG01091	1.198238532	2.475694915	0.000175162	0.001330929
PARG10393	-1.485346526	1.570525185	0.000175704	0.001334396

PARG20196	-1.011317245	5.763292863	0.00017682	0.001341741
PARG02451	-1.010044417	7.180812674	0.000176842	0.001341741
PARG21267	1.011107788	5.633643608	0.000177341	0.001344879
PARG24257	1.005086676	7.10654602	0.000177883	0.00134834
PARG00520	-1.027060492	4.599118146	0.000178156	0.001349758
PARG26441	-1.03391113	3.618182913	0.000178494	0.001351663
PARG27109	-1.03806096	4.16831419	0.000178584	0.001351694
PARG06177	-1.025801364	3.572019716	0.00018046	0.001365232
PARG18188	-1.02989358	4.269398458	0.000180991	0.001368593
PARG01554	-1.03188875	4.170687227	0.000181441	0.001371333
PARG00506	-1.459858392	-0.000740003	0.000181719	0.001372772
PARG21282	1.012458223	4.671584375	0.000183246	0.001383646
PARG12550	1.092281264	2.341662331	0.000184813	0.001394803
PARG11666	1.014033098	5.465092009	0.000185162	0.00139677
PARG09755	-1.820665671	-0.812793273	0.000185422	0.001398057
PARG07094	1.007905369	6.694218023	0.000185989	0.001401657
PARG06516	-1.037411569	3.565424823	0.00018985	0.001428697
PARG30008	-1.304042976	-0.107155377	0.000190623	0.001433826
PARG28314	-1.06823592	2.80101875	0.000191033	0.001435695
PARG29970	1.004005464	5.279373839	0.000191054	0.001435695
PARG07161	1.017033294	6.12664434	0.000193511	0.001453461
PARG27174	-1.018170409	3.958396495	0.00019382	0.001454527
PARG27160	1.108894921	5.559593859	0.000193838	0.001454527
PARG26463	1.050868377	6.289830587	0.000194034	0.001455305
PARG15362	-1.637956568	-0.444859937	0.000194549	0.001458471
PARG20077	1.029791401	4.002279128	0.000194938	0.001460688
PARG08111	1.055735137	4.35675145	0.000195133	0.001461452
PARG06331	-2.383216394	-0.708650369	0.000201281	0.001505338
PARG09355	2.308790947	-0.170466782	0.000201281	0.001505338
PARG23373	-1.631612874	-0.597488584	0.000201673	0.001507555
PARG19524	-1.004171519	5.557878674	0.000202019	0.001509422
PARG12153	1.023917449	5.023199016	0.000202468	0.001512054
PARG08376	1.221927426	1.321084942	0.000204024	0.001522227
PARG07467	1.121871561	4.189520926	0.000205353	0.001531416
PARG24443	3.743381618	1.979701813	0.000205502	0.001531797
PARG20699	-1.127104177	2.52880921	0.000206753	0.001540393
PARG16936	1.482129125	0.099150971	0.000207522	0.001543927
PARG12785	-1.476067005	0.195771244	0.000207522	0.001543927
PARG04929	1.03791172	4.004951822	0.000207924	0.001546186
PARG23934	1.01012475	3.915542021	0.00020852	0.001549881
PARG16593	-1.710129704	1.837547075	0.000208821	0.001551382
PARG12381	-1.000828539	4.876049273	0.000210248	0.001561242
PARG23478	-1.00082005	5.31446015	0.000211695	0.001570507
PARG06722	-1.033532167	2.82987244	0.000212993	0.001579387
PARG16450	-1.052337178	2.486562502	0.000214344	0.001588654
PARG19107	-1.30359069	0.537578353	0.000214798	0.00159127

PARG05925	1.016344543	5.005305465	0.000217657	0.001610926
PARG22976	-1.059144308	4.335006193	0.000219059	0.00162054
PARG28130	-1.016914585	3.214085997	0.000220952	0.001633003
PARG09363	-1.250864806	0.186592623	0.000222396	0.001642908
PARG23268	-1.041784476	3.256933206	0.00022342	0.001648916
PARG19933	-1.221007021	3.230395931	0.000224045	0.001651202
PARG12356	1.073650592	2.294462763	0.000224193	0.001651516
PARG04098	-1.096654871	2.31134838	0.000226342	0.001665784
PARG18392	-1.00065699	4.967433101	0.000228187	0.001678573
PARG07797	-1.09935745	2.162492871	0.000228522	0.001680252
PARG03194	1.415683744	1.878712376	0.000229713	0.001688221
PARG09070	-1.008062684	4.137551228	0.000229929	0.001689014
PARG25006	-1.059976972	2.701380384	0.000230499	0.001692408
PARG19932	1.052383232	4.072710846	0.000232891	0.00170892
PARG27418	1.38433724	1.23093659	0.000232965	0.00170892
PARG02923	-1.367765877	1.031185789	0.000234416	0.001717955
PARG00233	-1.124519458	3.198968281	0.000235203	0.001722923
PARG00449	-1.035754043	2.617482207	0.000235488	0.001724206
PARG24649	-1.092987683	2.004167547	0.000236759	0.001732703
PARG24785	1.000669275	4.916452246	0.000237006	0.001733701
PARG08512	-1.603981641	0.526301005	0.000237895	0.001738581
PARG22956	-1.04105874	3.541036974	0.000239604	0.001748633
PARG14528	-1.043967628	2.244110215	0.000244049	0.001778592
PARG08249	-1.00474478	5.791533714	0.000248104	0.00180563
PARG05752	-1.028780186	3.272527147	0.0002489	0.001809745
PARG29310	1.093216085	3.166939892	0.000250536	0.001820797
PARG25227	1.46026493	7.458201631	0.000252702	0.001834835
PARG24574	-1.051882191	2.3586664	0.000254191	0.001844793
PARG15512	1.039244563	3.243763101	0.000255272	0.001850926
PARG11388	-1.070506041	4.526182455	0.000262248	0.001898
PARG27654	1.009687776	5.232101096	0.000263899	0.001909072
PARG02288	-1.064099567	2.519314854	0.000267005	0.001926213
PARG24106	-1.007166461	4.430112634	0.000268075	0.001932157
PARG15273	-1.529945782	-0.534749321	0.000272597	0.001962952
PARG03397	1.056331105	3.177782025	0.000273895	0.001971389
PARG05643	-1.211484818	1.037587275	0.000274271	0.001973191
PARG13692	-1.320261662	0.21107592	0.000276411	0.001986765
PARG21785	1.263253986	0.150776838	0.000277911	0.001996117
PARG00431	1.018225564	3.505396755	0.000278391	0.001998251
PARG13792	-1.056538088	2.104500706	0.000279461	0.002004553
PARG28061	-1.003587709	3.84256517	0.000279524	0.002004553
PARG09456	-1.669764155	-0.413032365	0.0002828	0.002025272
PARG26710	-1.654631153	2.541034271	0.0002828	0.002025272
PARG13132	-1.145162145	2.520930887	0.000286046	0.002046985
PARG02409	-1.002830047	3.536062327	0.000286598	0.002049662
PARG21313	1.503971837	2.202717931	0.000286912	0.002050978

PARG21250	-1.118447064	1.030446463	0.000291232	0.002080914
PARG20092	-1.158609168	0.861536863	0.00029268	0.002090304
PARG00828	-1.022782639	3.297135707	0.000295358	0.002108471
PARG11411	1.100713824	2.394723977	0.000298192	0.002124836
PARG13775	-1.092792401	1.782880953	0.000298192	0.002124836
PARG08385	1.177144816	1.278436799	0.000302504	0.002152635
PARG20580	-1.016565382	3.46212701	0.000305547	0.002167407
PARG19718	-1.877143129	2.048681737	0.000306258	0.002171468
PARG25632	-1.218491154	0.536616074	0.000309421	0.002191399
PARG03496	1.034346579	4.905304578	0.000309792	0.002192565
PARG26208	-1.385010749	-0.217667426	0.000312034	0.002207442
PARG12844	-1.052361173	4.212443075	0.000312287	0.002208236
PARG26763	-1.014576078	2.834251006	0.000314038	0.002218893
PARG18886	-1.06982238	2.145380054	0.000314077	0.002218893
PARG19574	-1.276391063	2.312713506	0.000317732	0.002243703
PARG21122	1.731702385	0.115112899	0.000323295	0.002279676
PARG19446	-1.081021707	1.180169778	0.000323407	0.002279676
PARG22817	-1.0003249	2.956874699	0.00032759	0.002305025
PARG12303	-1.068048428	2.071336643	0.000327775	0.00230529
PARG23921	-1.166442733	0.571666482	0.000328655	0.002309411
PARG18724	-1.130740891	1.12827592	0.000331557	0.002327718
PARG11241	-2.807629835	-0.454599051	0.000332489	0.002333219
PARG25801	-1.019088369	2.56590235	0.000332687	0.002333563
PARG01912	1.061814706	4.283449931	0.000334203	0.002343156
PARG13571	-1.085451312	1.661081222	0.000337027	0.002359793
PARG11100	-1.136670091	1.745080702	0.000338513	0.002369136
PARG07727	-1.207334568	1.104191013	0.000340786	0.002383986
PARG03461	-1.026544363	2.470437219	0.000347506	0.002426924
PARG10805	1.239931326	4.711315943	0.000353281	0.00246152
PARG29162	-1.236188741	0.462765149	0.000357626	0.002489581
PARG23153	-1.066219507	1.558984169	0.000359333	0.002499254
PARG06052	-1.250031081	1.174285754	0.000359689	0.002500282
PARG18854	1.108284726	4.268876888	0.0003598	0.002500282
PARG07760	-1.280025382	0.344002406	0.000362578	0.002516737
PARG19692	1.853531117	5.527498903	0.000362649	0.002516737
PARG21231	1.052380014	3.639801532	0.000364932	0.002530343
PARG24332	-1.007099353	3.565047573	0.000367482	0.002544122
PARG11284	-1.377622958	-0.126061959	0.000367684	0.002544122
PARG20130	-1.314996631	1.512696246	0.000367729	0.002544122
PARG12081	-1.379158916	1.527196803	0.000368281	0.002544575
PARG27823	-1.377735713	-0.109799925	0.000368281	0.002544575
PARG16675	-1.376004824	0.354823782	0.000368281	0.002544575
PARG06761	-1.32899206	0.193295606	0.000368944	0.002548033
PARG27216	-1.098888602	1.579162179	0.000374579	0.002582408
PARG14232	-1.051371804	2.546765919	0.000376955	0.002597252
PARG12624	-1.03904718	2.495874531	0.000377063	0.002597252

PARG23144	1.086326101	2.689705284	0.0003794	0.002609914
PARG27929	-1.076521654	2.114592277	0.000382334	0.002628943
PARG18900	-1.908391321	-0.244619333	0.000386604	0.002654813
PARG17108	-1.890808876	-1.024483998	0.000386604	0.002654813
PARG14213	-1.199856767	1.547825084	0.000390633	0.002678966
PARG14453	-1.109665921	1.576866535	0.000399236	0.002730812
PARG29311	1.102539708	1.707494183	0.000399236	0.002730812
PARG26030	-1.05309782	1.941239491	0.000399518	0.002731549
PARG04885	-1.558294125	-0.331727813	0.000400771	0.002738919
PARG16933	1.070243437	4.802405518	0.000402209	0.002747555
PARG11836	-1.004060025	3.37071284	0.0004024	0.002747664
PARG13652	-1.834394439	-0.513376668	0.000407876	0.002779011
PARG20620	-1.011577653	5.116801211	0.000415597	0.002827932
PARG22009	1.514957103	-0.082083984	0.000417457	0.002837026
PARG03480	1.083159424	2.513475646	0.000423158	0.002873154
PARG07748	-1.031739083	1.421964187	0.000426484	0.002893235
PARG10729	-1.310213237	0.049941162	0.000430908	0.002916943
PARG14132	-1.00853263	3.022847953	0.000433426	0.002930197
PARG00466	-1.072003005	2.6677412	0.000434294	0.00293358
PARG06157	-1.41600057	0.097607898	0.0004343	0.00293358
PARG19015	-1.398293015	1.255682583	0.000435	0.002937044
PARG27020	-1.002580501	3.180748992	0.000435862	0.002941598
PARG13682	-1.093009624	2.340700171	0.000437715	0.00295241
PARG02299	-1.152233637	1.301484261	0.000438949	0.002958621
PARG18551	-1.056564307	1.916683821	0.000439904	0.002963785
PARG04831	5.119757161	0.353911886	0.000440227	0.002964685
PARG27245	-1.060967783	2.712029049	0.000441805	0.002973973
PARG12331	1.09276315	6.080597087	0.000442174	0.002973973
PARG02682	-1.084531882	1.32014359	0.000442174	0.002973973
PARG06916	-1.066943421	1.437618608	0.000446985	0.003003753
PARG00028	-1.029400603	1.879164959	0.000447215	0.003004012
PARG27246	-1.002431931	2.718875089	0.000448218	0.003009443
PARG20862	1.037134297	2.667985335	0.000452417	0.003032468
PARG02105	1.027977817	4.805190562	0.000461199	0.00308343
PARG20456	1.022141204	2.978617099	0.000470182	0.00314215
PARG12961	1.000089902	3.797034445	0.000472508	0.003155011
PARG12969	-1.178754207	0.086360237	0.00047651	0.003177679
PARG22653	1.167936522	0.508989939	0.00047651	0.003177679
PARG27131	-1.035730425	2.46914527	0.000486369	0.00323381
PARG18935	-1.055195181	1.435765864	0.000487255	0.003238324
PARG00194	-1.145445468	1.056454732	0.000489427	0.00325012
PARG10392	-1.577701052	-0.563209507	0.000489443	0.00325012
PARG15774	-1.275387062	0.199361568	0.000492317	0.003266437
PARG02249	1.945292209	-0.329239744	0.000492851	0.003268601
PARG24546	1.305489906	1.291442463	0.000497792	0.003295799
PARG13408	1.077455126	2.397754233	0.000498242	0.003297384

PARG02286	-1.042961895	2.613378046	0.000502379	0.003321965
PARG20743	-1.49175312	2.05138814	0.000512948	0.003387572
PARG07615	-1.040645016	2.596574352	0.000513914	0.003392527
PARG23759	1.35022594	0.656058162	0.000514663	0.003394945
PARG22911	-1.154205686	0.711946559	0.000514808	0.003394945
PARG07283	-1.209039404	0.382123513	0.00051528	0.003395828
PARG20498	-1.01321748	2.798011256	0.000515777	0.003397681
PARG26570	-1.068125165	1.943451168	0.00051689	0.003402153
PARG13747	-1.173007899	2.780092692	0.000522711	0.003436147
PARG00191	-1.069175851	1.532513681	0.000523113	0.003437346
PARG00291	1.030148637	3.130457507	0.000538142	0.003527247
PARG20560	-2.416463949	0.013994941	0.000539457	0.003532912
PARG20329	1.807581057	1.22210512	0.000542941	0.00355277
PARG06011	1.718465775	-0.382224652	0.000542941	0.00355277
PARG16700	-1.233914712	1.783265122	0.000543481	0.003554817
PARG20296	1.012711004	4.269768763	0.000549521	0.003589836
PARG05171	1.058083013	2.682504166	0.000555673	0.003625499
PARG05190	-1.079784419	2.070895513	0.000565387	0.003682758
PARG12847	3.580571521	4.063156241	0.000597412	0.003870479
PARG00885	3.504628248	0.187497736	0.000597412	0.003870479
PARG21327	-1.163264072	0.383001459	0.000602387	0.003901104
PARG12027	-1.058792168	1.758594918	0.000605055	0.00391354
PARG08410	-1.33913824	-0.139308287	0.000612652	0.003956152
PARG21468	1.554545285	0.467148444	0.000614409	0.003965868
PARG00168	1.344355245	3.958563399	0.000616547	0.003978036
PARG07124	-1.218181772	-0.174803532	0.000621203	0.004001499
PARG25152	-1.370155429	-0.07390806	0.000627055	0.00403588
PARG15837	1.585585501	-0.070063638	0.000627766	0.004038679
PARG23432	-1.388862074	0.048864794	0.00063003	0.004050046
PARG03967	1.020591818	1.601431393	0.000669243	0.004280509
PARG17496	1.787870162	0.948853526	0.000669426	0.004280509
PARG26821	-1.754612317	-0.374345346	0.000669426	0.004280509
PARG29653	-1.150546577	0.447979911	0.000686926	0.004385262
PARG10962	-1.036848552	2.19863769	0.00070393	0.0044738
PARG12714	-1.109054349	0.737493029	0.000705576	0.004480638
PARG23222	-1.150129875	1.596797356	0.000709801	0.004502004
PARG19874	1.094225992	1.243145482	0.000713123	0.004521247
PARG18921	1.040110575	3.023095198	0.000732602	0.004633429
PARG06481	1.322009626	2.866347278	0.000733179	0.004633429
PARG16209	-1.067961892	1.231124116	0.000735514	0.004646315
PARG02632	1.649141349	0.940138305	0.000741766	0.004676406
PARG27214	-1.638186048	-0.879324874	0.000741766	0.004676406
PARG17247	-1.12834372	1.179292267	0.000744041	0.004688863
PARG02803	-1.259758671	0.124033504	0.000752169	0.00473059
PARG00945	-1.698722336	-0.307562885	0.000757333	0.004755444
PARG24922	-1.616976238	-0.278365868	0.000757333	0.004755444

PARG27969	-1.616869521	0.426326415	0.000757333	0.004755444
PARG25451	-1.231212684	1.168756925	0.000770381	0.00482772
PARG03358	-1.42910657	-0.645962468	0.000788606	0.004924237
PARG20834	-1.427697826	-0.224643738	0.000788606	0.004924237
PARG11835	-1.518507194	-0.597018842	0.000789803	0.004927794
PARG27558	1.031867177	2.970397367	0.000796376	0.004964856
PARG25281	-1.020805524	1.532379703	0.000811957	0.005053216
PARG12487	-1.004564691	2.586986685	0.000815454	0.005071615
PARG16987	-1.001137168	1.996860611	0.000817789	0.005082205
PARG10634	-2.677064246	0.124935918	0.000822852	0.00511165
PARG07472	-1.210195978	-0.245765119	0.000843987	0.005223973
PARG18249	-1.280365511	-0.321427016	0.000860816	0.005313838
PARG16495	1.092509403	1.37705424	0.000870159	0.005365183
PARG13349	1.813104096	1.499908998	0.000871975	0.005374268
PARG25122	-1.070906103	1.075556179	0.000875479	0.005391635
PARG18489	1.020940144	2.431611747	0.000891395	0.005481051
PARG27299	-1.242321593	0.985776595	0.000892196	0.005483826
PARG27079	-1.842431291	-0.418110807	0.000908636	0.005571784
PARG22823	-1.077672045	0.900870026	0.000936585	0.00572974
PARG04388	1.238056184	3.074890513	0.000959752	0.005855494
PARG21575	-1.129154779	1.020544905	0.000975867	0.00593766
PARG08031	-1.03633965	1.217671179	0.000979939	0.005957817
PARG22195	1.589007032	0.006342512	0.000989129	0.00601136
PARG05593	-1.138274827	2.728274933	0.000990397	0.006016735
PARG16046	-1.08867597	2.934766804	0.00100168	0.006078225
PARG19124	-1.492258683	-0.141908751	0.001002356	0.006079978
PARG13125	1.188884878	0.733973652	0.001023825	0.00618868
PARG06327	-1.062845275	1.535531667	0.001035733	0.006255843
PARG00538	1.151367772	1.011874668	0.001036838	0.006260107
PARG13869	1.311752596	1.852096757	0.001051608	0.006337101
PARG13134	1.034424788	4.369127624	0.001074833	0.006474571
PARG07039	-1.132979516	1.643757525	0.001075535	0.006476314
PARG08003	1.923874029	0.136848753	0.0010886	0.006534928
PARG25635	1.013451223	1.480063459	0.001097033	0.006579952
PARG07009	-1.33053314	1.127929417	0.001101228	0.006598119
PARG06056	-1.150588026	0.379549393	0.001144581	0.006816721
PARG03773	1.102984827	1.691123733	0.00114466	0.006816721
PARG16467	-1.063794102	0.585939696	0.001153102	0.006859185
PARG20616	1.391153165	-0.582231229	0.001185553	0.007033562
PARG05738	-1.375444281	-0.62597787	0.001185553	0.007033562
PARG19599	1.009623623	2.353809892	0.001202801	0.00712243
PARG20610	-1.156125211	0.048895973	0.00120732	0.007143802
PARG18515	1.407531802	0.067503525	0.001246956	0.007347863
PARG23651	-1.007238204	1.017815855	0.001251197	0.007367326
PARG05813	-1.646578939	1.616514333	0.001258008	0.007404656
PARG19699	-1.43500052	-0.329614671	0.001259872	0.007412843

PARG08433	-1.449010948	-0.171956628	0.001271196	0.007465484
PARG06684	1.109421914	2.277409892	0.001275018	0.007479748
PARG11166	-1.659404756	-0.334178585	0.001275441	0.007479748
PARG11946	-1.155536447	0.958917598	0.001275531	0.007479748
PARG25983	-1.529837987	0.879735789	0.001287306	0.007543163
PARG06557	1.174136054	0.490896618	0.001302154	0.007624476
PARG12561	1.053430581	2.975076264	0.001322921	0.00773453
PARG16442	-1.314195802	-0.040088794	0.001350423	0.007870842
PARG21407	1.092970714	1.649761812	0.001394442	0.008098394
PARG11394	1.198224789	0.950529323	0.001442372	0.008342797
PARG06671	1.381757303	-0.0588068	0.001455477	0.008412398
PARG07375	-1.097993319	0.995770603	0.001477182	0.008528428
PARG17939	-1.36266702	-0.065913794	0.001482317	0.008551786
PARG28319	4.88119712	1.412377032	0.001489124	0.008585967
PARG22216	1.028969416	1.929823185	0.001493181	0.008604976
PARG13430	-1.130494372	0.326530631	0.001506208	0.008670502
PARG21693	-1.382052819	4.617686888	0.001509109	0.008684013
PARG30344	1.031985071	2.387333547	0.001533463	0.008801571
PARG13582	1.332669929	0.349943806	0.001558693	0.008943117
PARG14144	2.874111116	3.347050823	0.001576871	0.009034203
PARG00470	1.806474281	0.784818377	0.001596885	0.009135534
PARG15516	-1.514350006	-0.622012503	0.001610856	0.009195353
PARG22005	1.525966103	0.194257565	0.00163313	0.009312342
PARG29952	-1.797936013	0.63510468	0.001640825	0.009352822
PARG09054	-1.475306055	-0.647902168	0.0016531	0.009409121
PARG27327	-1.749843483	2.404255064	0.001669622	0.009487925
PARG06917	1.733473585	-0.495286617	0.001686068	0.00956556
PARG15346	-1.090448643	0.141010007	0.00169704	0.009610436
PARG14196	-1.138732208	0.165114621	0.001719989	0.009726364
PARG23196	4.015499195	3.293872628	0.001756313	0.009913915
PARG12064	3.200760267	1.441771991	0.001756313	0.009913915
PARG10493	1.026250343	2.352207882	0.001770742	0.009978963
PARG00874	1.247907235	0.105016873	0.001775339	0.009999738
PARG06799	2.102191408	-0.015491509	0.00178809	0.010049924
PARG26667	-1.069568384	0.83923226	0.001796574	0.010083164
PARG16133	-1.055376039	-0.053607243	0.001801739	0.010101318
PARG06664	1.265808414	-0.174529732	0.001820284	0.010194367
PARG13239	-1.224813317	-0.250963569	0.001820284	0.010194367
PARG02089	1.156013385	1.014111089	0.001849053	0.010333367
PARG12296	-1.077887762	1.040310359	0.001867865	0.010423657
PARG03384	-1.119829749	2.183335009	0.001879977	0.010483792
PARG00802	-1.039874046	-0.208297177	0.001914949	0.010637252
PARG22247	-1.178429185	-0.1822106	0.00194516	0.010763172
PARG00259	1.165162881	1.271247328	0.00194516	0.010763172
PARG03518	1.054392621	1.589913803	0.001958447	0.01082906
PARG17594	-1.038096137	1.357569917	0.001988637	0.010972804

PARG10573	1.131815535	1.467920841	0.001993223	0.010990383
PARG23305	1.381900485	-0.593378585	0.001997065	0.011007701
PARG17638	1.068059552	1.949546457	0.002093295	0.011474682
PARG27312	1.079196218	0.357643495	0.0020995	0.011495621
PARG00255	-1.293551608	-0.539114635	0.002130286	0.01164794
PARG26332	1.209134514	0.785260653	0.002171902	0.011846598
PARG01901	1.578082388	0.007849442	0.00219211	0.011940228
PARG23942	-1.053466227	1.615392443	0.002229452	0.012122596
PARG16055	-1.252639838	-0.032645991	0.00223504	0.012148774
PARG10515	1.105514869	3.966200624	0.002267697	0.012313495
PARG00745	-1.212137073	0.293228182	0.002293598	0.01243263
PARG18342	1.018189391	1.308405025	0.002312352	0.01252132
PARG07610	-1.139322985	0.644130272	0.002331667	0.012604169
PARG03436	-1.034802399	0.504719696	0.002394191	0.012880058
PARG14378	-1.12096124	-0.214179963	0.002467841	0.013212879
PARG23256	1.128961489	1.264358881	0.002541212	0.013541053
PARG23498	1.123408818	0.930960813	0.002541212	0.013541053
PARG24558	1.080244792	1.828779156	0.002549134	0.013569449
PARG04642	1.105046861	0.396844339	0.002652586	0.014053423
PARG23093	1.080938454	1.490053551	0.002652586	0.014053423
PARG01622	2.094696535	5.315432386	0.002661869	0.014093092
PARG21733	2.046724312	1.047846825	0.002661869	0.014093092
PARG22771	-1.224119627	0.790392871	0.002673771	0.014146566
PARG03383	1.355147442	-0.539014888	0.002681412	0.014163127
PARG06184	1.324507714	1.621877443	0.002681412	0.014163127
PARG06002	1.005627809	1.067146214	0.002723234	0.014359866
PARG26039	-4.830192094	-0.806092937	0.002765516	0.01452428
PARG11986	-1.305899459	0.160293233	0.002768278	0.01453392
PARG00162	-1.011687426	0.826916297	0.002847482	0.014855356
PARG26944	-1.164919482	0.436231726	0.002875973	0.014994031
PARG01825	1.092497184	0.046157789	0.002896648	0.015083835
PARG29937	-1.858780985	-0.365149678	0.002967707	0.015400684
PARG16659	-1.001547239	1.755113835	0.003000628	0.015561233
PARG09251	1.784321231	0.11650781	0.003038521	0.015705861
PARG19441	1.780523755	0.081841453	0.003038521	0.015705861
PARG05736	1.385615799	1.658027168	0.003051839	0.01575395
PARG27906	-1.72648898	-0.159716099	0.003073318	0.015837331
PARG06376	1.061469363	0.892805943	0.003148822	0.016153662
PARG06614	-1.001540331	1.588885169	0.00315445	0.016173736
PARG17671	1.009662011	1.766914086	0.003158082	0.016185301
PARG16134	1.220720781	0.081482569	0.003313226	0.016837494
PARG11945	-1.003720185	1.729775337	0.003330099	0.016917766
PARG10342	-1.146275922	-0.259262023	0.003357266	0.017022725
PARG30402	-1.14381464	0.147382014	0.003357266	0.017022725
PARG07663	1.44600576	-0.349784692	0.003368038	0.017060812
PARG00751	1.423563272	0.593160056	0.003368038	0.017060812

PARG06288	-1.36751193	0.260027113	0.003368038	0.017060812
PARG09758	-1.458806053	0.783780563	0.003479215	0.017550359
PARG18142	1.085200905	0.139584072	0.003540215	0.0178237
PARG24543	-1.463182767	1.707685705	0.003592109	0.018040117
PARG21418	2.140938214	-0.255240489	0.003657417	0.018331461
PARG06699	-1.040603769	0.291850356	0.003664393	0.01836056
PARG21257	-1.189402213	0.946926832	0.003674627	0.018400093
PARG07240	1.518619797	1.412932634	0.003705543	0.018518268
PARG23905	-1.213264545	-0.438606809	0.003707664	0.018518268
PARG09811	-1.236949599	-0.084009399	0.003855555	0.019153916
PARG09705	1.532584753	1.393887826	0.003926832	0.019488903
PARG07737	1.304118124	1.064976161	0.00395706	0.019607267
PARG06827	1.505100002	5.690774773	0.004029327	0.019896941
PARG13533	-1.490606664	0.278102489	0.004029327	0.019896941
PARG12591	-1.490574347	2.322825645	0.004029327	0.019896941
PARG18440	-1.169365118	0.025963925	0.004198696	0.020565097
PARG00605	-2.769555752	0.058848587	0.004215024	0.020612909
PARG15170	2.705535039	4.688675566	0.004215024	0.020612909
PARG22136	2.620856899	0.350717365	0.004215024	0.020612909
PARG21539	-1.833786354	-0.557839069	0.004265862	0.020809665
PARG22716	-1.084798634	0.607740845	0.004370467	0.021234168
PARG04749	1.341491055	0.178005853	0.004447943	0.021530622
PARG28091	-1.059812152	1.860489308	0.004844373	0.02312864
PARG06838	-1.052826211	0.490567712	0.004844373	0.02312864
PARG00992	1.228110725	1.841160892	0.004901458	0.023351469
PARG07728	-1.217602737	-0.039243476	0.004901458	0.023351469
PARG03747	1.414962918	1.020894265	0.004994645	0.023737967
PARG10459	-2.431404321	0.433868935	0.005043113	0.023917458
PARG11887	-1.080367873	0.422389711	0.005105685	0.024192288
PARG07074	-1.424914869	0.352451563	0.005183326	0.024523161
PARG24962	1.183028415	-0.234158559	0.005194266	0.024567516
PARG26234	3.415106846	-0.427119988	0.005209351	0.024616613
PARG22828	3.160887474	0.78422229	0.005209351	0.024616613
PARG22388	1.221749654	1.289624856	0.005437803	0.025519473
PARG16192	1.065307748	3.146378133	0.005454001	0.025564919
PARG27244	-1.614758827	-0.357705838	0.005551881	0.025907677
PARG07799	1.028324103	1.318598829	0.005662739	0.026354486
PARG26642	-1.312237398	-0.617933152	0.005678384	0.026409882
PARG20955	1.041075487	6.079076006	0.005796975	0.02687564
PARG26212	1.02196314	0.50266472	0.005890995	0.027239128
PARG28410	1.114033246	1.851483434	0.005894155	0.027244882
PARG07181	-1.406779766	-0.251549599	0.005946334	0.027430372
PARG05120	1.188027961	1.064912052	0.005963274	0.027476219
PARG08194	1.167174563	0.380116301	0.005963274	0.027476219
PARG01735	1.164815704	0.703045451	0.005963274	0.027476219
PARG00567	1.687747094	-0.154221309	0.005977556	0.027525866

PARG13749	-1.185336921	-0.740979342	0.005980746	0.027532477
PARG23675	1.090819349	0.693918271	0.006195182	0.028328568
PARG21089	1.011954288	0.637573752	0.006209685	0.028365379
PARG27727	1.207699767	-0.334212328	0.006283709	0.028658242
PARG08673	1.18099791	0.149367081	0.006575453	0.029738249
PARG20048	-1.207688326	-0.399534068	0.00661035	0.029870258
PARG19691	-1.037128713	0.167545855	0.006670224	0.030069968
PARG21165	1.024587461	0.749178166	0.006670224	0.030069968
PARG08030	-1.024731402	0.995853793	0.006737216	0.030312645
PARG15826	-1.011473467	-0.62063238	0.006789502	0.030481421
PARG15478	1.13026307	-0.144754279	0.00680101	0.030494788
PARG07089	1.509347532	0.181952394	0.006829857	0.030586398
PARG27111	2.52543115	-0.057470765	0.006889411	0.030809609
PARG18838	-1.101590029	-0.053692791	0.006974632	0.031139868
PARG07097	1.137884243	0.839861686	0.007151898	0.031840754
PARG08748	1.261937477	0.078785908	0.007343964	0.032566537
PARG26560	-1.214990871	-0.68527238	0.007343964	0.032566537
PARG26193	-1.570632907	-0.193975742	0.007464685	0.033008626
PARG21752	-1.542818157	-0.02401801	0.007464685	0.033008626
PARG20660	-1.044284621	-0.550027853	0.007554319	0.033311154
PARG11462	-1.176812244	-0.103833569	0.007691098	0.033743671
PARG27796	1.118793155	0.222358352	0.007708837	0.033802604
PARG00604	-1.667202195	0.069483694	0.008084257	0.035163941
PARG17082	1.215657112	1.641681323	0.008127021	0.035282167
PARG08733	-1.036733922	-0.066185004	0.008328547	0.035967409
PARG10525	1.992230084	2.217590827	0.008469227	0.036404688
PARG11051	1.706376862	-0.149350549	0.008576896	0.036776869
PARG28249	-1.133226413	-0.26713641	0.008873304	0.037861683
PARG10120	1.46634646	0.954726203	0.008981842	0.038283183
PARG15458	2.977262235	0.794127406	0.008991581	0.038303891
PARG10589	4.446162473	1.512178056	0.009732353	0.040989895
PARG26400	4.445875539	1.716299788	0.009732353	0.040989895
PARG02396	1.060189124	-0.447798367	0.009750894	0.041048412
PARG15938	-1.267789605	2.434147913	0.009779196	0.041134465
PARG07337	1.544391013	2.51798488	0.010014438	0.041977743
PARG17970	-1.410295397	-0.576027408	0.010014438	0.041977743
PARG08883	-1.000263398	0.934722333	0.010248334	0.042764046
PARG11902	-1.568534037	-0.41926348	0.010560056	0.043854964
PARG13746	-1.436263506	1.678951676	0.010560056	0.043854964
PARG07679	-1.219933366	1.254038233	0.010963837	0.045197034
PARG12518	-1.570295205	0.475646441	0.011112639	0.04564265
PARG05137	2.411157415	3.591859361	0.011249028	0.046081088
PARG26733	1.190784077	0.20048732	0.011299144	0.046232474
PARG27346	1.087355389	1.547010401	0.011546123	0.047041535
PARG08117	-1.21675444	0.364337579	0.011699046	0.047455202
PARG15290	1.210191478	-0.251024956	0.011699046	0.047455202

PARG22918	-1.228925193	-0.25504411	0.012057704	0.048658569
PARG22405	1.173475149	1.810076127	0.012057704	0.048658569
PARG30098	-1.170186378	0.170581282	0.012057704	0.048658569
PARG23815	-1.110091869	0.649635682	0.012232431	0.049249732
PARG08762	-2.263654089	1.363656188	0.01240086	0.049838378

Table S15B. Differentially expressed genes between G2 and CT of *P. armeniaca*

Gene ID	logFC	logCPM	PValue	FDR
PARG22277	8.730285627	8.193930339	4.85E-119	7.61E-115
PARG25438	9.372259298	6.832771838	4.06E-116	3.19E-112
PARG17245	7.940251024	10.59841139	4.41E-108	2.31E-104
PARG10526	8.895112979	6.510675407	3.75E-106	1.47E-102
PARG13140	7.855343565	9.395248588	1.15E-105	3.61E-102
PARG22907	7.987188207	7.352352949	1.56E-103	4.08E-100
PARG10537	8.282716325	6.602181242	4.26E-102	9.56E-99
PARG11781	-8.167842668	6.113088335	1.27E-94	2.49E-91
PARG17298	8.954961246	5.035250279	1.13E-91	1.97E-88
PARG05219	-7.171498383	7.248659342	1.72E-88	2.70E-85
PARG21115	6.772460208	9.327280172	5.46E-87	7.79E-84
PARG25227	6.94733016	7.458201631	1.52E-84	1.93E-81
PARG04489	6.59199536	10.32133236	1.62E-84	1.93E-81
PARG04476	9.938624241	4.194383578	1.72E-84	1.93E-81
PARG19347	6.907654053	7.607759947	6.71E-84	7.02E-81
PARG13529	-6.510674463	9.275087172	2.81E-82	2.76E-79
PARG27196	-7.810046248	5.341966537	4.31E-82	3.98E-79
PARG12451	-6.512288841	9.229520181	1.61E-81	1.41E-78
PARG14084	-6.395431342	9.466798252	2.41E-80	1.99E-77
PARG28341	6.437842042	7.91436219	2.32E-78	1.82E-75
PARG15170	7.595657458	4.688675566	4.36E-78	3.26E-75
PARG00379	-8.416645755	3.947440625	8.61E-78	6.15E-75
PARG08422	-6.86864756	4.64642581	1.24E-75	8.43E-73
PARG01622	7.063176047	5.315432386	8.14E-75	5.33E-72
PARG02124	6.466112465	6.245969829	9.09E-74	5.71E-71
PARG20793	5.977072536	9.31716554	2.16E-73	1.30E-70
PARG18508	-7.950122761	3.625695873	1.15E-72	6.69E-70
PARG18207	7.041652742	5.538681479	2.12E-72	1.19E-69
PARG20144	6.903839736	3.852334062	4.61E-72	2.50E-69
PARG03568	-5.986000987	8.089594688	5.46E-72	2.86E-69
PARG13767	5.833352583	9.94507246	6.66E-72	3.37E-69
PARG06492	-7.09256853	4.664072937	1.79E-71	8.76E-69
PARG04195	-7.385187528	4.599632293	5.82E-69	2.77E-66
PARG20971	6.97894699	5.096349376	6.71E-69	3.10E-66
PARG23463	-5.907564842	6.719844608	1.86E-68	8.32E-66
PARG07745	6.220240296	5.710948452	1.92E-68	8.35E-66
PARG13116	-5.992675122	7.648694314	1.07E-67	4.53E-65
PARG22542	6.896769704	4.584814588	1.33E-67	5.49E-65
PARG07565	-6.377982585	5.044670576	2.59E-67	1.04E-64
PARG12281	-5.514072425	9.93089886	2.76E-66	1.08E-63
PARG18492	-6.585953401	5.440767058	2.54E-65	9.72E-63
PARG29805	5.433008979	10.30760297	5.73E-65	2.14E-62
PARG00480	-7.142396137	4.263435823	8.07E-65	2.95E-62

PARG24019	5.914515719	6.251846757	5.95E-64	2.12E-61
PARG19183	5.362224539	10.14716196	1.02E-63	3.58E-61
PARG27316	-5.788571844	5.216829071	1.68E-63	5.74E-61
PARG27924	7.469710588	3.274409324	3.49E-63	1.17E-60
PARG22901	-5.41116355	8.813600126	5.99E-63	1.96E-60
PARG08146	-5.363437085	9.014529711	3.06E-62	9.81E-60
PARG13638	-6.256788548	4.679933228	3.28E-62	1.03E-59
PARG19346	5.335394369	7.993819783	1.11E-61	3.40E-59
PARG01159	-5.410947182	7.692360326	5.41E-61	1.63E-58
PARG05631	6.389814105	4.934425924	3.40E-60	1.01E-57
PARG05984	6.295275065	4.671222124	9.60E-60	2.79E-57
PARG24499	5.122447163	11.18431737	1.17E-59	3.34E-57
PARG12048	7.378948089	2.537448315	2.51E-59	7.05E-57
PARG00044	-7.198665452	3.975131716	2.85E-59	7.86E-57
PARG02097	-5.604979553	5.588016367	5.86E-59	1.59E-56
PARG27582	5.081177722	10.12594402	7.02E-59	1.87E-56
PARG00361	5.19448301	8.2241675	1.13E-58	2.96E-56
PARG27587	-6.229897425	5.499271835	1.14E-57	2.94E-55
PARG22553	-6.440370716	4.776872896	1.17E-57	2.97E-55
PARG26578	-5.344687054	4.853115191	1.46E-57	3.65E-55
PARG23240	-6.698620653	3.371469134	1.67E-57	4.09E-55
PARG13228	-6.10024615	5.092055756	1.23E-56	2.96E-54
PARG19032	5.00593137	8.383062611	1.30E-56	3.10E-54
PARG21172	-7.902957969	3.671825215	1.50E-56	3.51E-54
PARG24952	-5.135907117	6.544324624	1.95E-56	4.49E-54
PARG20799	5.013373915	6.652479619	3.79E-55	8.63E-53
PARG10888	5.658601729	4.509890828	5.66E-55	1.27E-52
PARG23958	-5.18369827	6.167025266	1.60E-54	3.53E-52
PARG00468	5.057222877	6.449093071	1.83E-54	3.97E-52
PARG29164	-6.245391115	3.511569003	1.85E-54	3.97E-52
PARG00172	-5.545497854	6.376466441	2.71E-54	5.74E-52
PARG09988	-7.756201473	2.583237603	4.07E-54	8.52E-52
PARG11779	-5.129169134	5.967322074	4.38E-54	9.04E-52
PARG23846	-5.106756311	6.565365764	7.29E-54	1.49E-51
PARG07517	-6.429628347	2.952461446	1.48E-53	2.98E-51
PARG00777	-5.813954918	4.664086585	2.63E-53	5.22E-51
PARG00046	-7.36924171	3.009833691	4.47E-53	8.76E-51
PARG11568	6.717403043	3.096668554	7.63E-53	1.48E-50
PARG24185	4.812123811	7.651244289	1.19E-52	2.27E-50
PARG30259	-7.661156793	3.942188352	1.36E-52	2.56E-50
PARG16361	-5.116816391	7.503314518	1.44E-52	2.70E-50
PARG03795	4.871141587	6.316880089	1.85E-52	3.41E-50
PARG26917	-4.742844511	8.051723783	2.77E-52	5.05E-50
PARG18110	-4.744979738	8.23317952	3.07E-52	5.54E-50
PARG25239	-6.029925781	3.201581325	4.25E-52	7.58E-50
PARG19023	4.719167337	8.981044124	7.79E-52	1.37E-49

PARG25648	-5.455114554	5.370977861	8.97E-52	1.56E-49
PARG07116	5.308235886	4.772836214	9.88E-52	1.70E-49
PARG17956	-6.344199752	2.976423855	1.12E-51	1.91E-49
PARG11792	-7.584689663	3.845794816	1.83E-51	3.09E-49
PARG25305	-4.753652945	6.180139941	3.58E-51	5.97E-49
PARG07518	-4.842403801	5.235779098	4.22E-51	6.97E-49
PARG08502	-4.927135164	6.539190074	5.64E-51	9.22E-49
PARG15485	-6.507847743	3.91445385	7.02E-51	1.14E-48
PARG12283	-6.503218375	4.306508795	1.90E-50	3.04E-48
PARG16099	4.57638015	9.024628357	2.52E-50	4.00E-48
PARG24540	4.732962405	6.228670193	2.68E-50	4.21E-48
PARG11797	-4.60009564	7.040665438	4.73E-50	7.34E-48
PARG19225	4.931010866	5.133964049	8.20E-50	1.26E-47
PARG23901	-4.929185485	5.026243499	9.58E-50	1.46E-47
PARG21235	-4.814072558	5.71475641	1.84E-49	2.78E-47
PARG24197	-6.887322456	3.346868891	2.39E-49	3.57E-47
PARG21693	5.562032669	4.617686888	4.47E-49	6.62E-47
PARG19880	-5.250678365	5.352558103	4.74E-49	6.95E-47
PARG07606	-4.600807485	6.757822486	6.50E-49	9.45E-47
PARG11341	4.63513674	6.390015649	6.74E-49	9.71E-47
PARG09652	-10.41524543	3.723885058	7.96E-49	1.14E-46
PARG25174	5.092822093	4.085208464	1.14E-48	1.62E-46
PARG02116	-5.106213862	4.614519823	1.27E-48	1.78E-46
PARG00129	-4.491441284	7.800788745	2.47E-48	3.43E-46
PARG11915	4.715905319	7.223375872	2.79E-48	3.85E-46
PARG29804	4.568905822	7.247023953	4.57E-48	6.24E-46
PARG06615	-4.715939507	4.888758631	1.08E-47	1.46E-45
PARG27751	4.613080185	5.920677439	1.14E-47	1.53E-45
PARG03782	-4.978122271	3.808969837	1.26E-47	1.67E-45
PARG28239	-4.725571859	5.52702782	1.37E-47	1.81E-45
PARG23736	5.725538122	1.798890447	1.41E-47	1.84E-45
PARG19170	-5.688111215	2.436974899	1.47E-47	1.91E-45
PARG17710	4.5241774	5.836481046	2.00E-47	2.57E-45
PARG25758	-7.318769388	2.213966878	2.27E-47	2.89E-45
PARG02727	4.398619962	8.717336886	2.38E-47	3.01E-45
PARG01264	5.202528456	4.311839986	1.01E-46	1.27E-44
PARG27712	-8.375184029	3.83700515	1.11E-46	1.38E-44
PARG26566	5.924672444	2.134629261	1.27E-46	1.57E-44
PARG15708	4.474247438	6.270926396	1.88E-46	2.30E-44
PARG11892	4.322471452	9.069960687	4.38E-46	5.33E-44
PARG01190	-5.168203262	3.543673658	4.43E-46	5.35E-44
PARG18167	-5.618362392	4.427306026	4.73E-46	5.67E-44
PARG10062	6.542915212	1.47991312	5.30E-46	6.31E-44
PARG13796	-4.369197993	7.638701005	5.93E-46	7.00E-44
PARG19216	-4.708552723	5.373863946	1.10E-45	1.29E-43
PARG02095	-4.474847819	5.315126319	1.28E-45	1.48E-43

PARG18453	-4.581259029	5.107540447	1.34E-45	1.55E-43
PARG20176	4.313033192	7.73471866	6.84E-45	7.84E-43
PARG10150	4.749224453	5.220183431	8.16E-45	9.28E-43
PARG06177	-5.849138222	3.572019716	8.26E-45	9.33E-43
PARG16679	-5.145831919	5.74744331	1.01E-44	1.13E-42
PARG20865	-5.846043263	2.956353055	2.51E-44	2.79E-42
PARG24692	4.523456367	5.4475888	2.55E-44	2.81E-42
PARG16426	4.327775657	5.995352954	2.69E-44	2.96E-42
PARG29258	-4.748135045	3.569489576	3.47E-44	3.78E-42
PARG11132	5.603095462	4.464986062	3.95E-44	4.27E-42
PARG27173	-4.551450583	4.126915247	4.16E-44	4.48E-42
PARG12205	-4.845168847	5.91854037	5.21E-44	5.56E-42
PARG11775	-4.688302068	4.63450306	6.63E-44	7.04E-42
PARG20550	-4.512940848	4.875176285	8.92E-44	9.40E-42
PARG25647	-4.235632694	8.278113323	1.20E-43	1.25E-41
PARG03713	-4.98167815	3.651321382	1.55E-43	1.61E-41
PARG03481	-4.357522638	5.571462541	2.47E-43	2.55E-41
PARG15180	-4.294743583	6.582881807	2.67E-43	2.74E-41
PARG05089	4.236652044	5.959231917	3.79E-43	3.86E-41
PARG07630	4.616795951	5.555366392	5.42E-43	5.49E-41
PARG21173	-4.988989989	4.656804801	5.65E-43	5.68E-41
PARG25225	4.391542826	5.84433043	6.07E-43	6.07E-41
PARG04197	-4.177551893	8.127885135	7.65E-43	7.61E-41
PARG14777	-4.87456073	3.718937336	8.93E-43	8.82E-41
PARG18488	-4.178233498	7.189478947	1.09E-42	1.07E-40
PARG27730	7.483828827	2.161381367	1.18E-42	1.15E-40
PARG01859	4.10043977	7.391165706	1.48E-42	1.43E-40
PARG19222	-4.317617519	6.132473518	3.02E-42	2.91E-40
PARG27865	4.554792053	4.73437751	3.60E-42	3.45E-40
PARG08360	-4.830261512	4.218518664	4.38E-42	4.17E-40
PARG08259	4.139280283	6.536433078	5.18E-42	4.90E-40
PARG15201	-5.58921662	2.881780891	5.31E-42	5.00E-40
PARG12355	-5.967377759	2.034800469	6.16E-42	5.75E-40
PARG03693	4.223439757	4.860707651	6.64E-42	6.17E-40
PARG06006	-4.60425282	6.004376269	6.69E-42	6.18E-40
PARG23746	6.928389894	0.980446044	7.12E-42	6.53E-40
PARG07744	4.35437579	4.934802396	8.95E-42	8.16E-40
PARG01969	5.440661483	2.893871309	1.02E-41	9.26E-40
PARG16777	-4.192699838	6.62937097	1.40E-41	1.26E-39
PARG13320	-4.496944872	6.32575185	1.46E-41	1.31E-39
PARG18487	-4.08647701	7.505257791	1.50E-41	1.34E-39
PARG23515	-4.285203338	5.443572565	1.58E-41	1.40E-39
PARG00264	-5.338747316	2.430837211	2.09E-41	1.84E-39
PARG12168	4.116545343	6.652027382	2.32E-41	2.03E-39
PARG07278	-4.406171083	5.900035483	2.38E-41	2.08E-39
PARG05189	-8.088868158	2.885470894	2.72E-41	2.36E-39

PARG12240	-5.812259971	2.066021923	2.83E-41	2.44E-39
PARG14743	-4.476166224	4.493037737	3.66E-41	3.14E-39
PARG12431	8.559564301	1.884659778	4.23E-41	3.60E-39
PARG27852	3.994237137	12.51556735	5.40E-41	4.59E-39
PARG20806	4.34087445	4.610467416	5.57E-41	4.70E-39
PARG14105	-4.858147888	3.553484233	7.43E-41	6.24E-39
PARG27002	-4.362193204	5.272522663	8.19E-41	6.84E-39
PARG20938	-6.11549632	2.58770477	1.82E-40	1.51E-38
PARG29533	-8.031017284	2.228166351	1.90E-40	1.57E-38
PARG15735	-4.073523809	6.029933354	2.58E-40	2.12E-38
PARG24520	5.79104944	1.989019402	3.14E-40	2.56E-38
PARG01914	4.309678994	4.794801611	3.83E-40	3.12E-38
PARG20089	4.91278007	3.806816755	3.88E-40	3.14E-38
PARG12550	-6.520866554	2.341662331	4.88E-40	3.93E-38
PARG17811	-4.671679617	3.275094782	4.95E-40	3.96E-38
PARG19047	-4.266804856	4.587247797	5.03E-40	4.01E-38
PARG27288	-4.522120188	4.197876572	5.63E-40	4.46E-38
PARG28173	4.660031398	2.945030981	7.08E-40	5.59E-38
PARG14428	-4.027562904	6.030457351	1.03E-39	8.08E-38
PARG25907	-9.788557129	1.439985558	1.43E-39	1.12E-37
PARG19000	3.895938142	7.647234824	2.81E-39	2.18E-37
PARG16791	5.62244334	3.053031075	5.52E-39	4.27E-37
PARG13689	4.991513394	3.025313936	5.92E-39	4.55E-37
PARG24343	4.386469542	4.405325532	6.24E-39	4.78E-37
PARG17758	-5.375707576	3.263500387	8.88E-39	6.77E-37
PARG03434	-5.146567021	3.315655296	1.05E-38	7.98E-37
PARG25121	-4.151413169	3.372626466	1.55E-38	1.17E-36
PARG03289	-6.721671064	3.071782156	1.63E-38	1.22E-36
PARG27160	3.938611588	5.559593859	1.64E-38	1.23E-36
PARG18525	-4.416565708	4.900813778	1.73E-38	1.28E-36
PARG02780	3.937962	7.01106058	1.92E-38	1.42E-36
PARG07644	-4.215531911	4.249838185	2.00E-38	1.48E-36
PARG26786	-4.524710661	5.185679998	2.45E-38	1.80E-36
PARG12486	-3.992847502	6.735443472	3.79E-38	2.77E-36
PARG14419	3.821959397	7.635084153	4.76E-38	3.46E-36
PARG08997	6.370913694	0.586975555	7.69E-38	5.56E-36
PARG11371	3.82871997	8.899758252	1.06E-37	7.61E-36
PARG08769	3.959119299	4.675093367	1.49E-37	1.07E-35
PARG19719	3.77399994	9.797303367	1.98E-37	1.41E-35
PARG08340	4.055244982	6.385860039	2.29E-37	1.63E-35
PARG05137	5.255899056	3.591859361	2.33E-37	1.65E-35
PARG26687	-6.085182951	3.540503561	2.76E-37	1.95E-35
PARG13587	-4.093993118	3.860734379	3.42E-37	2.40E-35
PARG20800	5.098972651	2.714263175	3.99E-37	2.78E-35
PARG20647	-4.131988405	5.514356508	4.60E-37	3.20E-35
PARG14573	-3.862844816	5.781011327	5.63E-37	3.89E-35

PARG02007	-3.746143686	8.186296047	6.27E-37	4.32E-35
PARG10254	-3.981739603	4.945906366	6.42E-37	4.40E-35
PARG02441	-4.22970576	5.529634432	8.08E-37	5.51E-35
PARG07150	4.382039569	5.189614086	9.94E-37	6.75E-35
PARG26716	-6.039843139	2.673223253	1.66E-36	1.12E-34
PARG10865	-4.639298685	3.614944452	2.00E-36	1.35E-34
PARG00949	-3.722985538	8.309613706	2.07E-36	1.39E-34
PARG07525	-4.510896535	3.736580275	2.87E-36	1.92E-34
PARG22964	-9.543969669	2.087895129	3.24E-36	2.15E-34
PARG11480	-4.310240894	3.077912066	4.07E-36	2.70E-34
PARG02478	4.586513992	4.69308581	4.77E-36	3.15E-34
PARG26918	-4.24018749	3.8338281	4.83E-36	3.17E-34
PARG06814	-4.571992704	4.716202415	5.14E-36	3.36E-34
PARG27420	-4.859116819	4.012790367	5.37E-36	3.50E-34
PARG19897	-5.805721567	3.795065825	6.72E-36	4.36E-34
PARG06678	-4.222143904	5.699298752	7.61E-36	4.91E-34
PARG12385	-4.231184476	6.029414944	8.46E-36	5.44E-34
PARG28155	-5.504896596	4.264810704	8.54E-36	5.47E-34
PARG10540	-3.736873478	6.679984331	8.60E-36	5.47E-34
PARG11782	-3.7699444	6.404104332	8.61E-36	5.47E-34
PARG28111	-4.129705338	5.492252802	1.00E-35	6.32E-34
PARG23829	4.578113917	4.316701143	1.00E-35	6.32E-34
PARG01533	-3.707045946	6.254483681	1.38E-35	8.69E-34
PARG23166	-4.039264092	4.230315859	1.41E-35	8.83E-34
PARG20341	3.646602095	8.978916733	1.85E-35	1.15E-33
PARG18352	3.770049916	5.749598588	4.11E-35	2.55E-33
PARG00014	3.665305598	6.55969333	4.18E-35	2.58E-33
PARG07512	-3.832505508	5.599638533	4.38E-35	2.70E-33
PARG02036	-4.394787595	4.171261163	5.55E-35	3.40E-33
PARG11057	5.787955206	1.467023684	5.88E-35	3.59E-33
PARG21733	4.64958165	1.047846825	7.40E-35	4.50E-33
PARG02979	-3.616018854	7.896442031	8.83E-35	5.35E-33
PARG02376	-4.073900866	4.027927572	1.07E-34	6.45E-33
PARG26698	-3.704233405	5.787191315	1.18E-34	7.11E-33
PARG26555	-3.7240943	5.831188478	1.46E-34	8.72E-33
PARG05813	5.055360627	1.616514333	1.52E-34	9.05E-33
PARG16776	-3.658350661	6.433025883	1.77E-34	1.05E-32
PARG28541	-4.998725181	2.850432838	1.78E-34	1.06E-32
PARG06722	-5.207929407	2.82987244	1.83E-34	1.08E-32
PARG10762	-3.654968839	6.450185232	1.92E-34	1.13E-32
PARG11582	-3.827552751	4.73917078	2.82E-34	1.65E-32
PARG07213	-3.86244603	5.443566521	3.31E-34	1.93E-32
PARG16784	3.944470752	4.050273438	3.33E-34	1.93E-32
PARG18979	-3.5720221	7.771244793	3.57E-34	2.07E-32
PARG14831	-3.649388026	6.995467098	3.76E-34	2.17E-32
PARG07523	-6.783044524	3.24490587	4.07E-34	2.34E-32

PARG12386	-3.679205036	6.466359035	4.67E-34	2.68E-32
PARG02134	-4.42811262	4.329989878	5.07E-34	2.89E-32
PARG06087	3.612469783	6.988714936	5.24E-34	2.98E-32
PARG08886	-3.706075032	5.545223571	5.35E-34	3.03E-32
PARG00600	-5.188071063	1.951359713	8.09E-34	4.57E-32
PARG08462	3.676001764	5.429103949	8.12E-34	4.57E-32
PARG10302	6.082136831	0.609364475	8.29E-34	4.65E-32
PARG24423	-3.714537089	4.71284024	1.14E-33	6.39E-32
PARG08216	3.58665225	5.877418313	1.28E-33	7.10E-32
PARG29969	-5.829893907	3.091276449	1.29E-33	7.14E-32
PARG24618	-4.550358405	2.976709201	1.29E-33	7.14E-32
PARG05244	-4.269945717	4.456333064	1.38E-33	7.59E-32
PARG22798	-6.368710451	1.176634194	1.55E-33	8.50E-32
PARG07982	-4.188212562	3.929741282	1.82E-33	9.98E-32
PARG19686	3.575333233	7.039791176	2.03E-33	1.11E-31
PARG26400	5.413403789	1.716299788	2.54E-33	1.38E-31
PARG01043	3.55667928	9.265096106	2.82E-33	1.53E-31
PARG08581	3.596155008	6.912156597	3.05E-33	1.64E-31
PARG13237	-7.505643528	1.95757396	3.82E-33	2.05E-31
PARG10986	-4.642557158	3.156895156	4.86E-33	2.60E-31
PARG03262	-6.023305429	2.832655116	5.20E-33	2.77E-31
PARG20630	-3.574019824	6.631439541	7.43E-33	3.95E-31
PARG12381	-3.818518114	4.876049273	7.84E-33	4.16E-31
PARG07422	-5.757075169	3.005897598	1.17E-32	6.19E-31
PARG07047	-3.913630585	4.632348215	1.24E-32	6.52E-31
PARG06005	-3.777990291	5.881364148	1.28E-32	6.72E-31
PARG23111	-3.622239995	5.028134087	1.48E-32	7.74E-31
PARG02477	3.68774853	4.175265458	2.16E-32	1.13E-30
PARG25801	-5.684135044	2.56590235	2.23E-32	1.16E-30
PARG08849	-4.026676359	3.176938232	2.25E-32	1.16E-30
PARG14619	5.766721883	2.080165774	2.36E-32	1.22E-30
PARG06847	-4.084347314	4.948629072	2.46E-32	1.27E-30
PARG16075	3.450472078	7.89754604	2.73E-32	1.40E-30
PARG18722	-3.989837649	5.382941203	3.22E-32	1.65E-30
PARG26463	3.477702976	6.289830587	3.60E-32	1.84E-30
PARG12757	3.846878294	3.118209281	4.08E-32	2.07E-30
PARG02278	3.504289093	7.232505963	4.74E-32	2.40E-30
PARG21023	3.728093771	4.354439916	5.51E-32	2.78E-30
PARG12847	4.687436547	4.063156241	5.65E-32	2.85E-30
PARG03467	3.582268575	7.186372012	6.02E-32	3.02E-30
PARG27152	3.460467155	6.567940235	7.34E-32	3.67E-30
PARG15445	-6.610039723	1.653731697	7.75E-32	3.86E-30
PARG15815	-4.376482897	3.630568447	9.11E-32	4.53E-30
PARG04182	-4.811561415	2.617569302	9.61E-32	4.76E-30
PARG17531	-5.682575391	1.657955278	9.99E-32	4.93E-30
PARG26794	-3.506829696	6.390242836	1.03E-31	5.08E-30

PARG14144	4.586216843	3.347050823	1.28E-31	6.26E-30
PARG00597	3.969241787	2.946272287	1.51E-31	7.39E-30
PARG04927	-3.918664379	3.150710181	1.95E-31	9.51E-30
PARG27133	-3.564933593	5.117970021	2.15E-31	1.04E-29
PARG12740	-3.428510783	7.144148348	3.12E-31	1.51E-29
PARG13128	4.55052103	3.752673808	3.67E-31	1.77E-29
PARG29207	-4.479506308	5.486358705	3.71E-31	1.79E-29
PARG03967	-5.091454299	1.601431393	4.03E-31	1.94E-29
PARG06612	-4.286475975	3.949629031	4.77E-31	2.28E-29
PARG00670	-3.970456074	2.391064797	5.19E-31	2.48E-29
PARG28102	-5.634383601	2.686162656	5.87E-31	2.79E-29
PARG07504	-5.15841755	3.00429027	6.27E-31	2.98E-29
PARG13621	4.401316228	2.299666185	6.78E-31	3.20E-29
PARG07190	4.717453068	1.83632637	6.98E-31	3.29E-29
PARG29379	-9.131768694	1.781447427	7.44E-31	3.50E-29
PARG29281	3.340607661	9.441587798	7.49E-31	3.51E-29
PARG28181	-3.75320757	3.297524396	7.80E-31	3.65E-29
PARG11773	-3.503452951	4.629658493	8.16E-31	3.80E-29
PARG20354	3.47658651	5.408473597	9.11E-31	4.23E-29
PARG28418	-3.494934718	4.795608201	1.01E-30	4.68E-29
PARG29762	-3.399167532	6.758164943	1.19E-30	5.49E-29
PARG00249	-3.981935521	4.609023205	1.53E-30	7.04E-29
PARG21170	-6.435700147	2.013861131	1.54E-30	7.06E-29
PARG08908	-5.054822585	3.005007174	1.64E-30	7.52E-29
PARG07520	-7.216440133	1.185537875	1.77E-30	8.07E-29
PARG12331	3.453019395	6.080597087	1.86E-30	8.48E-29
PARG19410	-5.767017131	2.967590333	2.00E-30	9.07E-29
PARG11217	-4.494413841	4.586940012	2.18E-30	9.84E-29
PARG23974	4.95816767	3.079507663	2.29E-30	1.04E-28
PARG00251	-4.877280773	2.098041037	2.91E-30	1.31E-28
PARG12928	5.181955176	3.897305928	3.55E-30	1.59E-28
PARG21141	-4.285465682	3.670319917	3.99E-30	1.78E-28
PARG23537	-3.568037691	4.535288041	4.00E-30	1.78E-28
PARG07022	-4.303247623	2.928478154	4.00E-30	1.78E-28
PARG27151	3.345536711	6.245545028	4.29E-30	1.90E-28
PARG06237	-4.977474482	1.283786526	4.52E-30	2.00E-28
PARG06239	-5.368174318	1.76964929	4.85E-30	2.14E-28
PARG21516	-3.643662626	4.36186531	5.01E-30	2.20E-28
PARG23512	-3.639525041	4.764096527	5.33E-30	2.34E-28
PARG15043	-5.092840985	2.773933655	5.68E-30	2.48E-28
PARG09088	-3.355654515	6.759129309	6.42E-30	2.80E-28
PARG12404	-3.37296762	4.977456159	7.29E-30	3.17E-28
PARG11911	-3.541220777	4.639281226	8.99E-30	3.90E-28
PARG24245	-3.417396527	5.501414092	9.12E-30	3.94E-28
PARG09013	3.332343483	7.099883102	1.21E-29	5.21E-28
PARG08522	-4.355338341	4.336083148	1.30E-29	5.59E-28

PARG19996	-3.952595452	3.067800416	1.34E-29	5.73E-28
PARG19566	3.257802049	8.841542024	1.38E-29	5.89E-28
PARG11347	3.252476232	9.475010229	1.51E-29	6.43E-28
PARG03974	-3.624059028	5.248924146	1.65E-29	7.03E-28
PARG01361	3.350978963	5.94419924	1.90E-29	8.07E-28
PARG26344	-3.747921893	2.981523704	1.93E-29	8.17E-28
PARG06578	-4.602291465	3.39521291	2.16E-29	9.10E-28
PARG23707	3.242071377	9.282896958	2.27E-29	9.56E-28
PARG24602	8.926856017	1.579099982	2.62E-29	1.10E-27
PARG15463	3.23861551	9.020114558	2.73E-29	1.14E-27
PARG18947	-3.358172662	4.746226467	3.32E-29	1.38E-27
PARG13329	-3.690989283	3.696975711	3.62E-29	1.51E-27
PARG12356	-4.245256643	2.294462763	4.51E-29	1.87E-27
PARG29951	-3.870211853	3.323083703	4.82E-29	2.00E-27
PARG15135	-3.489812474	5.751032709	5.15E-29	2.13E-27
PARG03673	-5.473942335	2.101069582	5.32E-29	2.19E-27
PARG02094	-4.011512966	2.914544464	5.40E-29	2.22E-27
PARG05429	3.27297761	6.493181049	5.54E-29	2.27E-27
PARG05890	3.251254388	6.500190524	5.55E-29	2.27E-27
PARG00917	-3.491628121	3.585172331	5.64E-29	2.30E-27
PARG14504	-3.275393622	7.19905561	6.01E-29	2.44E-27
PARG10624	3.429053767	5.123110817	6.84E-29	2.78E-27
PARG02443	-3.784305538	3.380773188	6.94E-29	2.81E-27
PARG00280	3.208058879	9.191145857	7.42E-29	3.00E-27
PARG14135	-4.0778868	2.824851659	9.26E-29	3.73E-27
PARG04424	-3.729785014	3.473599978	9.34E-29	3.75E-27
PARG27650	-3.302546161	4.997432593	1.05E-28	4.21E-27
PARG21289	-3.462886004	4.965576133	1.07E-28	4.27E-27
PARG12563	3.191418868	12.15531093	1.07E-28	4.27E-27
PARG10875	-3.349906012	6.035576856	1.10E-28	4.39E-27
PARG11188	-5.993049946	2.047875037	1.21E-28	4.80E-27
PARG20475	-5.454781204	3.049569601	1.41E-28	5.58E-27
PARG16279	-5.918682951	2.820725174	1.47E-28	5.80E-27
PARG01206	-3.659560685	3.221642549	1.50E-28	5.89E-27
PARG23798	-5.449677931	3.033846216	1.66E-28	6.53E-27
PARG00168	3.543168645	3.958563399	1.79E-28	7.02E-27
PARG27579	3.164856633	10.72822455	2.76E-28	1.08E-26
PARG20804	3.654054772	3.327438369	3.67E-28	1.43E-26
PARG05669	-3.911635027	2.573849523	3.76E-28	1.46E-26
PARG00338	-3.385295779	5.093040606	3.95E-28	1.53E-26
PARG17150	-3.630274694	3.773216656	4.04E-28	1.56E-26
PARG06849	3.224037332	5.796583742	4.24E-28	1.64E-26
PARG04592	-3.159121232	7.368307661	5.86E-28	2.25E-26
PARG15623	-4.037819922	2.302201094	5.93E-28	2.28E-26
PARG20955	3.494617451	6.079076006	5.95E-28	2.28E-26
PARG24065	-4.856515157	2.902996627	6.43E-28	2.45E-26

PARG01874	-3.14513812	9.127555907	6.47E-28	2.46E-26
PARG20999	-4.923490546	2.14034574	7.00E-28	2.66E-26
PARG04512	-3.781550088	2.873464882	7.27E-28	2.75E-26
PARG06683	-3.446730059	5.145138417	7.75E-28	2.93E-26
PARG02935	-4.137538026	2.06425879	8.25E-28	3.11E-26
PARG17876	-4.448751492	2.643889828	8.82E-28	3.32E-26
PARG24142	3.137047926	8.891977445	9.37E-28	3.52E-26
PARG12163	-3.210041846	5.940583714	9.82E-28	3.68E-26
PARG10011	3.185857064	6.834896713	1.02E-27	3.82E-26
PARG06965	-3.429801508	4.005577975	1.19E-27	4.43E-26
PARG19632	-3.129836863	7.415529367	1.43E-27	5.34E-26
PARG21675	3.898174049	2.886536029	1.47E-27	5.44E-26
PARG12001	-3.117027401	7.060363756	1.70E-27	6.30E-26
PARG03265	3.109151792	10.19900094	1.74E-27	6.43E-26
PARG19014	-4.68649933	3.881608208	1.76E-27	6.47E-26
PARG03050	3.103208061	10.0343895	2.13E-27	7.85E-26
PARG01114	-6.248651981	2.031245387	2.20E-27	8.08E-26
PARG20307	3.209736096	5.032178826	2.30E-27	8.42E-26
PARG27711	-8.849087242	2.098076698	2.38E-27	8.67E-26
PARG07850	-3.421639899	4.649678035	2.43E-27	8.84E-26
PARG00620	3.111350149	7.05212646	2.60E-27	9.45E-26
PARG06146	-3.583420593	5.712872159	2.68E-27	9.71E-26
PARG18995	-3.428583635	4.264759107	2.74E-27	9.92E-26
PARG22468	-3.115155092	6.739563082	3.03E-27	1.09E-25
PARG03504	-3.249545356	5.397330472	3.40E-27	1.23E-25
PARG10941	-4.434008942	2.055539513	3.72E-27	1.33E-25
PARG12178	3.08701448	9.240210609	3.87E-27	1.39E-25
PARG22988	-3.421163638	3.191685269	4.35E-27	1.56E-25
PARG18434	-3.421290203	4.635326802	4.50E-27	1.60E-25
PARG03264	3.092266538	7.19631678	4.94E-27	1.76E-25
PARG15938	4.317817704	2.434147913	5.03E-27	1.79E-25
PARG05509	-3.504190271	4.271435152	5.45E-27	1.93E-25
PARG10612	-4.588561244	1.257742726	7.66E-27	2.71E-25
PARG19461	3.986879742	4.503551283	9.31E-27	3.28E-25
PARG19395	-3.187048616	5.81040785	9.75E-27	3.43E-25
PARG21543	3.053621659	10.84875741	1.03E-26	3.63E-25
PARG01980	3.230430568	4.219123935	1.04E-26	3.63E-25
PARG19839	3.930491869	3.465362209	1.04E-26	3.65E-25
PARG06533	-3.168650932	4.983662815	1.42E-26	4.94E-25
PARG28692	3.304318388	3.585735177	1.47E-26	5.13E-25
PARG01516	3.570351938	4.251837636	1.48E-26	5.13E-25
PARG15525	-8.779559888	1.968268533	1.51E-26	5.22E-25
PARG20345	3.103663516	5.733389395	1.59E-26	5.50E-25
PARG29414	3.611187766	1.740831047	1.67E-26	5.75E-25
PARG21631	-4.179773156	3.478053336	1.70E-26	5.85E-25
PARG24443	4.163739234	1.979701813	1.85E-26	6.34E-25

PARG25311	-4.155868233	3.16870845	1.90E-26	6.51E-25
PARG06059	-8.771285997	2.232203171	1.90E-26	6.51E-25
PARG27150	3.282001731	4.628440327	2.07E-26	7.06E-25
PARG19369	-3.706714866	2.703214492	2.07E-26	7.06E-25
PARG25127	-5.443316294	1.551394365	2.13E-26	7.24E-25
PARG13677	-3.421150307	4.012564414	2.32E-26	7.87E-25
PARG23234	3.035371282	8.331382825	2.52E-26	8.53E-25
PARG23027	-5.730313172	2.687741582	2.73E-26	9.22E-25
PARG15616	-4.303698877	1.505363781	2.81E-26	9.47E-25
PARG05371	-3.154396495	5.054807554	2.82E-26	9.49E-25
PARG29493	3.080791179	6.54138001	2.87E-26	9.61E-25
PARG29284	-4.256969723	2.989021598	3.14E-26	1.05E-24
PARG00421	3.062372842	6.134645437	3.40E-26	1.13E-24
PARG10268	-4.394261536	2.657478141	3.72E-26	1.24E-24
PARG19217	-3.212023803	5.153819549	3.74E-26	1.24E-24
PARG11379	3.032560761	7.047191018	3.77E-26	1.25E-24
PARG12715	3.019671551	8.100914022	3.99E-26	1.32E-24
PARG24760	-6.850814253	0.917087669	4.41E-26	1.46E-24
PARG10003	3.971920779	1.884377916	4.41E-26	1.46E-24
PARG18558	3.008439096	8.0054816	5.67E-26	1.86E-24
PARG13088	-3.554111732	3.759731034	6.61E-26	2.17E-24
PARG05638	-4.882625719	2.047848204	7.42E-26	2.43E-24
PARG16074	3.348254804	3.683968051	7.54E-26	2.47E-24
PARG13339	3.413551452	2.733872648	8.17E-26	2.67E-24
PARG18449	-3.349556289	3.284756138	9.07E-26	2.96E-24
PARG26486	-4.753679305	2.523669865	9.38E-26	3.05E-24
PARG16266	-5.036520946	1.995227471	9.54E-26	3.09E-24
PARG24224	-3.693969333	3.112557984	1.04E-25	3.36E-24
PARG12621	3.489038614	3.519390811	1.04E-25	3.37E-24
PARG11891	2.996651536	7.182108299	1.05E-25	3.38E-24
PARG23038	3.156843461	4.609119998	1.25E-25	4.01E-24
PARG10999	3.068088981	5.063388315	1.37E-25	4.41E-24
PARG06911	2.994860905	7.274821809	1.47E-25	4.71E-24
PARG16958	-3.052316944	5.55455745	1.49E-25	4.75E-24
PARG11844	-3.124650149	5.568860605	1.88E-25	6.00E-24
PARG24543	4.194464348	1.707685705	2.15E-25	6.85E-24
PARG28562	-3.685236028	3.652335769	2.22E-25	7.05E-24
PARG07853	3.035101061	5.891381587	2.59E-25	8.21E-24
PARG08916	-3.925854814	4.945133138	2.61E-25	8.26E-24
PARG28089	-2.97093713	7.170295455	3.11E-25	9.82E-24
PARG27107	-3.041995641	5.574000026	3.19E-25	1.01E-23
PARG06717	-3.017547449	5.401647073	3.20E-25	1.01E-23
PARG16796	3.273996266	5.138063728	3.37E-25	1.06E-23
PARG23153	-6.782958007	1.558984169	3.46E-25	1.08E-23
PARG07542	3.006065095	5.450570546	3.66E-25	1.15E-23
PARG07260	-4.169724386	3.103268069	3.89E-25	1.21E-23

PARG30213	-4.586395409	2.641602976	4.18E-25	1.30E-23
PARG16086	-5.396904872	1.512051572	4.83E-25	1.50E-23
PARG19933	3.343377622	3.230395931	5.03E-25	1.56E-23
PARG04069	3.188248739	3.286864706	5.11E-25	1.58E-23
PARG00513	-3.165860567	4.049504603	5.24E-25	1.62E-23
PARG10078	3.034770459	5.014926055	5.30E-25	1.64E-23
PARG09195	2.948773261	6.492783954	5.63E-25	1.73E-23
PARG05857	6.130873571	-0.100043934	5.64E-25	1.73E-23
PARG20696	-3.245887978	3.52855533	5.65E-25	1.73E-23
PARG22832	-3.225061549	4.920380521	6.38E-25	1.95E-23
PARG04282	-3.145773982	4.645099777	6.44E-25	1.97E-23
PARG26911	-3.124497765	6.217260483	7.56E-25	2.30E-23
PARG24857	3.082645698	4.809427209	8.05E-25	2.45E-23
PARG24292	-4.810244649	2.368769421	8.32E-25	2.53E-23
PARG03330	3.096055032	3.01220424	8.42E-25	2.55E-23
PARG27586	-4.007890581	2.224187394	9.08E-25	2.75E-23
PARG10515	3.212106235	3.966200624	9.39E-25	2.84E-23
PARG15845	-2.918302984	8.71924524	9.70E-25	2.92E-23
PARG18494	-3.019129475	5.405468619	9.84E-25	2.96E-23
PARG00509	-2.939298659	6.846842978	1.01E-24	3.04E-23
PARG26832	3.648081173	3.313123751	1.04E-24	3.11E-23
PARG11254	-3.148502821	4.23423155	1.07E-24	3.20E-23
PARG17623	-4.033539851	2.453462159	1.14E-24	3.41E-23
PARG07524	-4.832577934	2.007667878	1.16E-24	3.46E-23
PARG23878	-3.013426692	5.177948751	1.33E-24	3.95E-23
PARG20455	3.26361476	3.080337418	1.40E-24	4.16E-23
PARG27362	-8.600979739	1.239101484	1.54E-24	4.56E-23
PARG01095	4.544461542	1.75974924	1.70E-24	5.04E-23
PARG02859	-4.406404805	2.86145986	1.73E-24	5.10E-23
PARG14246	-3.26926247	3.309163511	2.10E-24	6.17E-23
PARG10474	-3.149008934	4.061031946	2.11E-24	6.21E-23
PARG03048	4.030983242	1.847805053	2.22E-24	6.50E-23
PARG29686	5.696229304	0.252428962	2.29E-24	6.72E-23
PARG00028	-5.332750169	1.879164959	2.34E-24	6.83E-23
PARG25195	-5.331877807	2.264801143	2.34E-24	6.83E-23
PARG11470	-4.448254619	0.614858443	2.35E-24	6.84E-23
PARG23935	-3.519792956	2.076027497	2.52E-24	7.31E-23
PARG03147	-5.992369079	0.714687623	2.70E-24	7.82E-23
PARG08672	-2.893659878	7.872191368	2.73E-24	7.90E-23
PARG07151	2.928339021	6.9946545	2.95E-24	8.54E-23
PARG01177	-5.097173653	2.42859424	2.97E-24	8.57E-23
PARG29494	2.879291096	9.819322205	3.04E-24	8.75E-23
PARG08741	3.347594279	3.212514796	3.29E-24	9.46E-23
PARG04098	-5.9751965	2.31134838	3.39E-24	9.73E-23
PARG10686	-3.794442434	2.037453098	3.61E-24	1.03E-22
PARG28358	-2.952978485	5.499880447	3.62E-24	1.03E-22

PARG10514	-6.684435615	1.361665264	3.72E-24	1.06E-22
PARG00948	-3.605635839	3.46815319	4.02E-24	1.14E-22
PARG14150	-4.087575908	3.128967359	4.05E-24	1.15E-22
PARG23272	-4.503497094	1.660098168	5.09E-24	1.44E-22
PARG29411	2.883225593	6.703298597	5.25E-24	1.49E-22
PARG11343	-2.971686011	5.318151451	5.80E-24	1.64E-22
PARG06582	-3.061124684	3.734404282	5.99E-24	1.69E-22
PARG03768	-3.175257567	4.226201287	6.53E-24	1.84E-22
PARG06318	-3.008026774	4.498106315	7.32E-24	2.06E-22
PARG16437	-3.265079262	3.551894968	7.97E-24	2.24E-22
PARG08244	2.924789235	5.512956097	8.52E-24	2.39E-22
PARG20114	2.885751997	5.711006545	9.70E-24	2.71E-22
PARG02715	2.845679495	8.35656797	1.13E-23	3.15E-22
PARG06109	-5.58238573	2.365954359	1.15E-23	3.20E-22
PARG19536	-8.515936063	0.838163661	1.21E-23	3.36E-22
PARG28555	-2.852227702	6.617118557	1.23E-23	3.42E-22
PARG18451	-2.944076593	6.317650533	1.34E-23	3.72E-22
PARG07718	-3.519721142	2.29229849	1.66E-23	4.58E-22
PARG02086	2.874660979	5.887007586	1.71E-23	4.74E-22
PARG27246	-3.938495985	2.718875089	1.88E-23	5.20E-22
PARG10998	3.123001858	3.295450132	2.05E-23	5.63E-22
PARG29757	-3.346864789	2.940052798	2.14E-23	5.87E-22
PARG24663	-5.557754217	1.611134555	2.18E-23	5.99E-22
PARG22817	-3.608275366	2.956874699	2.24E-23	6.14E-22
PARG03287	3.827424743	3.00021203	2.25E-23	6.15E-22
PARG03506	-5.895416051	0.997626344	2.77E-23	7.57E-22
PARG11595	-3.723087876	3.557770031	2.84E-23	7.74E-22
PARG10518	-2.82323954	6.905216119	2.84E-23	7.74E-22
PARG18172	2.837971748	6.582347495	3.55E-23	9.63E-22
PARG24815	-3.277913003	3.806081487	3.95E-23	1.07E-21
PARG06319	-3.00245925	4.798857189	4.18E-23	1.13E-21
PARG24653	-3.082023406	3.660451811	4.69E-23	1.27E-21
PARG20940	-3.504745043	2.733800351	4.77E-23	1.29E-21
PARG23125	-2.79314159	9.342733771	4.81E-23	1.30E-21
PARG05077	-2.804870409	7.812692107	4.90E-23	1.32E-21
PARG23934	-2.952295935	3.915542021	5.06E-23	1.36E-21
PARG06053	-3.846651299	1.542045627	5.68E-23	1.52E-21
PARG00027	-2.92651166	4.868051822	5.70E-23	1.52E-21
PARG24147	-4.745423075	1.022384483	5.77E-23	1.54E-21
PARG03336	-3.398410233	3.977255087	6.38E-23	1.70E-21
PARG19466	-3.029681997	5.382934495	6.45E-23	1.72E-21
PARG15193	-3.021687527	5.562150308	6.60E-23	1.75E-21
PARG22776	-2.827416139	6.071711867	6.90E-23	1.83E-21
PARG02155	-3.392253335	2.523762885	6.99E-23	1.85E-21
PARG07748	-4.784125231	1.421964187	7.66E-23	2.02E-21
PARG17829	-3.055135826	4.13874477	8.62E-23	2.27E-21

PARG11715	-3.43863671	2.876214074	8.68E-23	2.29E-21
PARG08334	-5.845791486	1.622125013	9.20E-23	2.41E-21
PARG03624	5.136902443	0.094844011	9.20E-23	2.41E-21
PARG25285	2.816764078	6.258511094	9.20E-23	2.41E-21
PARG07354	-2.776371851	8.588210428	9.28E-23	2.43E-21
PARG11409	-5.197821999	2.251623813	9.53E-23	2.49E-21
PARG24251	2.860101621	5.761858551	1.07E-22	2.80E-21
PARG19028	-3.268873312	2.501727181	1.10E-22	2.87E-21
PARG24609	2.922745695	4.934882515	1.16E-22	3.02E-21
PARG01376	-2.77691816	6.706338755	1.18E-22	3.07E-21
PARG27095	-2.775315384	6.526235312	1.23E-22	3.18E-21
PARG04593	3.964806467	0.547296843	1.24E-22	3.22E-21
PARG15722	-3.548805941	2.852329171	1.28E-22	3.31E-21
PARG16787	3.894453495	0.98938218	1.29E-22	3.33E-21
PARG16046	3.13530366	2.934766804	1.32E-22	3.40E-21
PARG19708	2.797108357	6.49205316	1.33E-22	3.41E-21
PARG26429	-4.61659939	2.862103534	1.42E-22	3.65E-21
PARG24594	3.239816504	3.416659566	1.45E-22	3.71E-21
PARG15910	-5.178342701	1.436774943	1.46E-22	3.73E-21
PARG15551	-2.979311701	4.616932911	1.49E-22	3.81E-21
PARG20619	-3.151428183	3.887019812	1.70E-22	4.33E-21
PARG14267	2.853407584	5.340458156	1.77E-22	4.49E-21
PARG27715	3.348171529	2.284685028	1.82E-22	4.61E-21
PARG11445	-5.2733176	0.512743065	1.97E-22	4.99E-21
PARG22064	2.741346901	11.12005746	2.23E-22	5.65E-21
PARG14539	5.899357676	1.522346512	2.32E-22	5.85E-21
PARG13092	-3.247716485	3.511057733	2.32E-22	5.87E-21
PARG28177	3.357875315	3.75000117	2.45E-22	6.17E-21
PARG04598	2.75620745	5.634842752	2.62E-22	6.59E-21
PARG06772	-2.75266557	7.107807654	2.86E-22	7.19E-21
PARG19857	-3.028407302	4.837508826	3.05E-22	7.65E-21
PARG27588	-3.059760308	3.368659359	3.35E-22	8.38E-21
PARG03375	-4.174509631	3.131087379	3.36E-22	8.39E-21
PARG01912	2.827763254	4.283449931	3.45E-22	8.61E-21
PARG20544	-3.109619515	3.787213929	3.70E-22	9.21E-21
PARG20315	-8.376666772	1.99460557	4.03E-22	1.00E-20
PARG23371	2.786149118	6.537077976	4.07E-22	1.01E-20
PARG07267	-3.045561308	3.345111705	4.38E-22	1.09E-20
PARG12839	-3.099063709	4.443764948	4.66E-22	1.15E-20
PARG14173	-2.947883281	4.157993063	4.77E-22	1.18E-20
PARG20139	3.213249648	1.463632534	4.77E-22	1.18E-20
PARG15363	2.785758975	5.114552978	4.84E-22	1.19E-20
PARG12243	-5.784114811	0.664562622	5.14E-22	1.26E-20
PARG15733	2.935907688	2.719437512	5.30E-22	1.30E-20
PARG18144	-4.191949123	3.663549985	5.55E-22	1.36E-20
PARG20870	-3.885581005	3.162392886	5.96E-22	1.46E-20

PARG12374	-3.043862813	3.261618279	6.04E-22	1.48E-20
PARG06209	2.742097407	7.094888377	7.05E-22	1.72E-20
PARG24516	-2.712689756	7.743540742	7.34E-22	1.79E-20
PARG27153	2.852419804	4.366511398	7.84E-22	1.91E-20
PARG03023	-2.999734629	4.31246366	7.92E-22	1.92E-20
PARG12316	-2.987690082	4.949289513	8.16E-22	1.98E-20
PARG22886	-5.764654868	0.742821048	8.48E-22	2.05E-20
PARG12184	-2.704028492	7.98905573	8.88E-22	2.15E-20
PARG18964	-3.413340507	2.842353307	9.32E-22	2.25E-20
PARG01207	-2.84022405	3.553698982	1.06E-21	2.55E-20
PARG20885	-3.851002491	2.940747719	1.18E-21	2.85E-20
PARG06905	-4.12421859	2.825836826	1.22E-21	2.94E-20
PARG13525	-8.32849967	1.070495018	1.24E-21	2.97E-20
PARG18479	4.304496934	2.428421327	1.27E-21	3.05E-20
PARG03038	2.699289402	7.202552581	1.36E-21	3.24E-20
PARG05590	-2.799848697	4.929753692	1.37E-21	3.27E-20
PARG15534	-2.835231256	5.450859691	1.41E-21	3.37E-20
PARG22850	-2.794327577	5.05586097	1.46E-21	3.49E-20
PARG07924	-3.059397916	3.726125844	1.48E-21	3.53E-20
PARG07922	2.681542074	7.519372024	1.73E-21	4.10E-20
PARG25344	3.178235003	2.586147262	1.75E-21	4.16E-20
PARG06628	-2.693076843	7.333019464	1.83E-21	4.33E-20
PARG07015	3.447297358	2.724250185	1.98E-21	4.68E-20
PARG15384	-2.874776646	4.001832764	2.12E-21	5.00E-20
PARG21234	-2.751330503	5.582164861	2.14E-21	5.04E-20
PARG05506	-2.894782427	4.536789397	2.31E-21	5.44E-20
PARG12577	-3.129389152	3.041548198	2.40E-21	5.65E-20
PARG02276	-2.763797261	5.112145597	2.47E-21	5.79E-20
PARG07478	-4.105895885	2.094046399	2.47E-21	5.80E-20
PARG10303	-4.78283876	2.819163078	2.51E-21	5.87E-20
PARG26646	-3.181113931	3.938474266	2.54E-21	5.94E-20
PARG19094	-2.707041523	6.001242263	2.64E-21	6.15E-20
PARG20468	-3.259006361	3.820131848	2.65E-21	6.16E-20
PARG21250	-8.299095863	1.030446463	2.91E-21	6.76E-20
PARG29234	2.663432249	8.507949155	3.03E-21	7.04E-20
PARG22471	-2.733772193	6.322375407	3.08E-21	7.14E-20
PARG06472	-2.873464341	4.286399195	3.29E-21	7.62E-20
PARG25154	-3.484194693	2.94063225	3.43E-21	7.93E-20
PARG17690	-2.945182558	3.92468545	3.50E-21	8.08E-20
PARG20549	3.621638476	2.251548245	3.61E-21	8.32E-20
PARG20164	-2.778417973	5.482875331	3.84E-21	8.84E-20
PARG23215	2.686852572	6.323647264	4.11E-21	9.44E-20
PARG12844	2.808526738	4.212443075	4.29E-21	9.84E-20
PARG21066	4.666652297	1.827345954	4.59E-21	1.05E-19
PARG20924	4.663367811	2.508895874	5.87E-21	1.34E-19
PARG07513	-3.058882668	2.742569177	5.92E-21	1.35E-19

PARG10955	3.184655187	2.474755173	6.00E-21	1.37E-19
PARG29269	-2.729935291	4.865055789	6.54E-21	1.49E-19
PARG07453	-3.162339365	3.358348611	6.58E-21	1.50E-19
PARG19534	-3.490458428	2.78237558	6.59E-21	1.50E-19
PARG04944	-2.871654419	4.619562067	6.72E-21	1.52E-19
PARG24357	2.678770076	5.635816497	8.21E-21	1.86E-19
PARG24124	2.639438121	7.170284191	8.45E-21	1.91E-19
PARG19428	2.83014177	2.924603592	9.96E-21	2.25E-19
PARG12440	-4.9454587	1.769744087	1.00E-20	2.25E-19
PARG25299	-4.9454587	1.827851034	1.00E-20	2.25E-19
PARG24199	-5.233474795	3.646357861	1.24E-20	2.79E-19
PARG18533	-2.658663335	6.652379432	1.27E-20	2.85E-19
PARG15494	2.749318895	4.7112467	1.28E-20	2.88E-19
PARG24668	-2.770033601	5.129292605	1.41E-20	3.16E-19
PARG10738	-3.373732045	2.532217771	1.45E-20	3.24E-19
PARG23149	2.602286162	10.92110824	1.56E-20	3.48E-19
PARG13248	-2.799368821	4.794160457	1.61E-20	3.58E-19
PARG17893	-4.9313435	1.778027231	1.62E-20	3.60E-19
PARG22178	-2.716993369	4.111717302	1.67E-20	3.71E-19
PARG13588	-4.295264471	0.275658179	1.76E-20	3.90E-19
PARG14416	-2.97276772	4.069459873	1.77E-20	3.93E-19
PARG14522	-3.158847143	2.886693917	1.84E-20	4.07E-19
PARG11417	3.062385021	1.122746165	2.00E-20	4.43E-19
PARG21139	-2.657812906	5.857641455	2.48E-20	5.47E-19
PARG14062	-5.275833125	1.799831587	2.62E-20	5.78E-19
PARG22469	-3.0916398	2.342581824	2.69E-20	5.93E-19
PARG20148	-3.806671111	2.527398892	2.73E-20	6.01E-19
PARG20568	-3.379245532	2.933104569	2.91E-20	6.39E-19
PARG06769	3.342505363	1.443760616	3.17E-20	6.95E-19
PARG24756	-5.193060643	1.783531797	3.40E-20	7.45E-19
PARG23179	2.679550913	4.579299918	3.44E-20	7.52E-19
PARG21634	-3.920749024	1.291848809	3.58E-20	7.82E-19
PARG27497	2.6195544	6.84570749	3.62E-20	7.89E-19
PARG02193	3.318563586	2.275244804	3.77E-20	8.20E-19
PARG06562	2.628895288	5.457043194	3.95E-20	8.59E-19
PARG29249	-8.173808758	1.132448861	4.08E-20	8.85E-19
PARG21171	-5.684518121	0.907463903	4.16E-20	9.01E-19
PARG13917	-3.743472728	2.053453503	4.68E-20	1.01E-18
PARG03620	3.428529808	1.026378809	4.71E-20	1.02E-18
PARG02466	-4.221412378	2.450268178	4.78E-20	1.03E-18
PARG08291	-2.76558843	3.900853421	4.86E-20	1.05E-18
PARG14257	-2.992968827	2.636783595	5.11E-20	1.10E-18
PARG11210	-2.9422496	4.378085393	5.11E-20	1.10E-18
PARG18358	-2.605386692	6.779770584	5.22E-20	1.12E-18
PARG07580	-2.815456254	4.103492263	5.59E-20	1.20E-18
PARG11658	-5.16922254	1.705830736	5.67E-20	1.21E-18

PARG08625	-2.951895891	4.420164471	5.81E-20	1.24E-18
PARG05029	6.279240617	-0.407340722	5.99E-20	1.28E-18
PARG06519	-6.286973803	1.928495736	6.06E-20	1.29E-18
PARG01221	-2.574617912	6.161981339	6.76E-20	1.44E-18
PARG15564	-3.527207095	3.51024955	7.15E-20	1.52E-18
PARG18637	-3.383275653	2.319518505	7.37E-20	1.56E-18
PARG19374	2.682370836	6.273275777	8.86E-20	1.88E-18
PARG10100	2.550385582	7.389299441	9.49E-20	2.01E-18
PARG19010	-5.14439659	3.202424718	9.50E-20	2.01E-18
PARG08190	-3.73060326	3.132647824	1.02E-19	2.16E-18
PARG17789	-3.029722281	2.807118601	1.03E-19	2.17E-18
PARG06039	3.572969624	2.793905715	1.08E-19	2.27E-18
PARG05246	2.632011562	5.219069704	1.08E-19	2.28E-18
PARG00885	3.745916755	0.187497736	1.14E-19	2.40E-18
PARG19161	2.594726438	5.514527125	1.15E-19	2.41E-18
PARG20715	-2.684254644	4.138403773	1.36E-19	2.85E-18
PARG14649	2.550520349	7.173803752	1.36E-19	2.85E-18
PARG11999	-8.125673875	-0.066323585	1.37E-19	2.86E-18
PARG23651	-8.121218957	1.017815855	1.37E-19	2.86E-18
PARG08385	-4.345066445	1.278436799	1.38E-19	2.88E-18
PARG11103	-2.569739445	6.835694752	1.39E-19	2.89E-18
PARG13439	-3.547459997	2.762159656	1.52E-19	3.17E-18
PARG20782	2.533208557	7.343904367	1.56E-19	3.24E-18
PARG02078	-2.552870095	5.418663104	1.58E-19	3.28E-18
PARG20321	-3.28026427	1.209094375	1.65E-19	3.42E-18
PARG02930	-4.214321229	2.502240902	1.65E-19	3.42E-18
PARG25129	-3.63596823	2.175050366	1.74E-19	3.59E-18
PARG07800	-2.903970638	3.098489565	1.77E-19	3.66E-18
PARG06115	3.104281992	3.578155021	1.82E-19	3.76E-18
PARG10688	-6.234779167	0.866635502	1.91E-19	3.92E-18
PARG14194	-2.929743989	3.15635327	1.91E-19	3.92E-18
PARG12459	-2.736053817	3.106791114	1.94E-19	3.98E-18
PARG01526	2.578806904	5.554066516	1.95E-19	4.00E-18
PARG19637	-3.397597348	1.720768729	2.01E-19	4.12E-18
PARG04316	2.835253584	3.30301582	2.08E-19	4.25E-18
PARG13593	-2.67051035	3.979217176	2.09E-19	4.27E-18
PARG25180	-3.495892512	2.866277116	2.12E-19	4.32E-18
PARG11108	-2.592651328	5.933578756	2.18E-19	4.44E-18
PARG25224	-2.589713137	5.701956799	2.19E-19	4.45E-18
PARG11224	-2.733992451	4.75480434	2.20E-19	4.47E-18
PARG05531	2.532892099	6.742728017	2.42E-19	4.91E-18
PARG21286	-2.594982672	4.771536959	2.42E-19	4.91E-18
PARG11351	-2.598886725	5.396855634	2.53E-19	5.11E-18
PARG13566	-2.56607978	5.922553827	2.63E-19	5.32E-18
PARG08016	2.524161662	7.070418216	2.65E-19	5.35E-18
PARG04563	2.511107787	7.927325989	2.75E-19	5.54E-18

PARG10805	2.726192233	4.711315943	2.83E-19	5.69E-18
PARG10508	-3.078648692	3.51729505	2.86E-19	5.75E-18
PARG06632	-3.252843407	3.038391352	2.88E-19	5.77E-18
PARG19555	-3.251944236	1.962692105	2.88E-19	5.77E-18
PARG19277	2.627262954	4.460930975	2.96E-19	5.93E-18
PARG19182	2.518162526	7.165088326	2.99E-19	5.98E-18
PARG13222	2.54009836	5.992449903	3.12E-19	6.24E-18
PARG11651	-3.087898969	4.143957506	3.25E-19	6.49E-18
PARG01173	2.568624054	3.956716194	3.62E-19	7.20E-18
PARG15464	2.510941127	7.444636179	3.63E-19	7.22E-18
PARG03417	2.503571838	6.927963462	3.70E-19	7.35E-18
PARG07957	-4.183445217	2.137397362	3.81E-19	7.57E-18
PARG07377	-2.797669176	4.490976442	3.88E-19	7.69E-18
PARG21489	2.49797395	7.475755239	4.02E-19	7.95E-18
PARG00725	2.577693714	4.949151279	4.06E-19	8.03E-18
PARG02048	2.543754557	5.255398337	4.51E-19	8.89E-18
PARG23637	-2.607326029	4.936137288	4.51E-19	8.89E-18
PARG05589	-6.195782904	1.534606489	4.58E-19	9.02E-18
PARG12014	-2.615027543	4.272847532	4.92E-19	9.68E-18
PARG13660	-4.238644403	1.15206994	5.05E-19	9.93E-18
PARG12147	-2.556334942	5.242157643	5.24E-19	1.03E-17
PARG19158	-2.767050281	4.278813783	5.38E-19	1.05E-17
PARG19327	-2.820782321	2.841798732	5.68E-19	1.11E-17
PARG29459	-2.592446702	4.819510286	5.74E-19	1.12E-17
PARG07282	-2.599872386	5.485674749	5.93E-19	1.16E-17
PARG18026	-2.488462266	8.70631205	5.98E-19	1.17E-17
PARG07761	-3.019537585	2.928925279	6.53E-19	1.27E-17
PARG09446	2.490505758	7.010524364	6.55E-19	1.27E-17
PARG27960	-3.660722343	1.482571561	6.64E-19	1.29E-17
PARG25302	-3.267073558	2.512121758	6.81E-19	1.32E-17
PARG04537	2.778534708	4.44566917	6.89E-19	1.34E-17
PARG24032	2.495090042	6.466931223	6.92E-19	1.34E-17
PARG05758	2.59431851	4.802730163	7.73E-19	1.49E-17
PARG02713	-2.549181836	5.416226458	7.73E-19	1.49E-17
PARG26570	-5.056179589	1.943451168	7.80E-19	1.50E-17
PARG16811	2.535451317	6.321661088	8.05E-19	1.55E-17
PARG07497	-6.164572432	2.252695541	8.27E-19	1.59E-17
PARG30010	-3.944103467	2.629435776	8.74E-19	1.68E-17
PARG30438	2.556624635	5.52737169	9.50E-19	1.82E-17
PARG11002	-2.827628022	2.821848117	1.04E-18	1.99E-17
PARG21175	-2.719796253	4.312290126	1.13E-18	2.15E-17
PARG23916	-4.739649432	2.18997684	1.14E-18	2.17E-17
PARG29450	-2.649795016	4.199744721	1.19E-18	2.27E-17
PARG19967	-3.896918915	3.59172978	1.20E-18	2.28E-17
PARG24854	-8.025872969	0.560696061	1.21E-18	2.31E-17
PARG26514	-8.017666162	0.877405351	1.21E-18	2.31E-17

PARG01425	2.469663292	6.841501166	1.22E-18	2.32E-17
PARG02771	-3.069727803	3.876021523	1.31E-18	2.48E-17
PARG20212	-3.638720438	1.930684344	1.31E-18	2.49E-17
PARG00827	-5.035839052	1.718007382	1.34E-18	2.53E-17
PARG06316	-3.279969574	3.138170995	1.35E-18	2.56E-17
PARG07767	2.724598318	4.011273546	1.36E-18	2.56E-17
PARG07337	3.217384501	2.51798488	1.39E-18	2.61E-17
PARG28162	-2.53443745	5.692386831	1.40E-18	2.63E-17
PARG10756	-2.541512068	4.380988341	1.45E-18	2.73E-17
PARG02984	-3.135822857	2.284246982	1.57E-18	2.95E-17
PARG03526	-2.707609899	4.118111659	1.64E-18	3.07E-17
PARG24138	2.558590427	4.665113619	1.67E-18	3.14E-17
PARG02946	-2.458588079	6.823088526	1.70E-18	3.18E-17
PARG28152	2.449193893	6.801679319	1.89E-18	3.55E-17
PARG11636	2.452839137	7.334619653	1.91E-18	3.56E-17
PARG16960	-2.513871389	4.564233675	1.92E-18	3.58E-17
PARG15430	-5.434840288	1.842786529	2.00E-18	3.72E-17
PARG11455	-2.847897004	3.455246427	2.16E-18	4.03E-17
PARG14107	3.430971212	1.913673704	2.20E-18	4.09E-17
PARG11859	-2.512809312	5.225751512	2.26E-18	4.20E-17
PARG19573	4.085629493	1.625004778	2.28E-18	4.23E-17
PARG00943	2.509236506	5.61063807	2.30E-18	4.25E-17
PARG23985	-3.019435389	3.142116895	2.31E-18	4.28E-17
PARG16104	2.436467821	8.641151885	2.46E-18	4.55E-17
PARG13273	-6.119988152	0.117474738	2.74E-18	5.07E-17
PARG27291	-2.490275865	6.258921555	2.76E-18	5.09E-17
PARG19882	-2.610672042	4.149798903	2.88E-18	5.31E-17
PARG22136	3.657753342	0.350717365	2.94E-18	5.40E-17
PARG26466	2.425776258	10.07692286	3.02E-18	5.55E-17
PARG19639	2.462563938	6.027483062	3.23E-18	5.93E-17
PARG06275	2.421560585	8.892177234	3.46E-18	6.35E-17
PARG29945	-2.555459896	3.422043691	3.59E-18	6.58E-17
PARG19268	-3.747758171	1.394276478	3.77E-18	6.90E-17
PARG19207	2.427031618	7.122291509	4.00E-18	7.32E-17
PARG09181	-2.915936059	3.495993002	4.20E-18	7.66E-17
PARG24649	-3.762369916	2.004167547	4.42E-18	8.06E-17
PARG27584	-2.531733844	4.266193104	4.57E-18	8.32E-17
PARG20043	2.416744449	7.141148458	4.70E-18	8.54E-17
PARG18666	-2.500415622	4.329006805	4.79E-18	8.71E-17
PARG17593	2.568917154	4.184181299	4.96E-18	9.00E-17
PARG29727	-4.068390801	1.506706082	5.01E-18	9.08E-17
PARG22115	-2.447354054	5.096674311	5.17E-18	9.36E-17
PARG14201	4.371918154	1.174512118	5.24E-18	9.47E-17
PARG09116	2.409416134	8.322715316	5.45E-18	9.85E-17
PARG28091	2.987779084	1.860489308	5.93E-18	1.07E-16
PARG12311	-2.457727772	5.602941072	6.00E-18	1.08E-16

PARG13057	3.265924812	2.864772658	6.04E-18	1.09E-16
PARG13361	-3.276673341	3.533959587	6.06E-18	1.09E-16
PARG21033	2.533391479	4.48715739	6.12E-18	1.10E-16
PARG18858	2.413645298	7.18482342	6.24E-18	1.12E-16
PARG12548	2.396543829	10.15548683	6.52E-18	1.17E-16
PARG18475	2.400945579	8.471933642	6.62E-18	1.19E-16
PARG10196	-3.74306363	1.798076347	6.69E-18	1.20E-16
PARG26431	2.774360909	2.872187525	7.34E-18	1.31E-16
PARG19718	3.756245721	2.048681737	8.06E-18	1.44E-16
PARG20561	-3.473630484	2.340132712	8.15E-18	1.45E-16
PARG08611	-2.477037802	5.323173632	8.35E-18	1.49E-16
PARG03477	-7.934226298	0.625251174	8.40E-18	1.49E-16
PARG00507	-2.69378701	3.338869436	8.49E-18	1.51E-16
PARG14706	-4.123957697	2.000010069	8.55E-18	1.52E-16
PARG29273	-4.652371845	2.007925713	9.04E-18	1.60E-16
PARG16450	-3.103272629	2.486562502	9.10E-18	1.61E-16
PARG10586	2.741364994	3.16726092	9.22E-18	1.63E-16
PARG22270	-2.383123834	10.62900828	9.75E-18	1.72E-16
PARG14769	-2.617240272	3.626465073	1.01E-17	1.78E-16
PARG24986	2.915363383	2.282410561	1.01E-17	1.79E-16
PARG21065	-3.38019848	3.152606628	1.02E-17	1.80E-16
PARG22734	-2.476324736	4.311874932	1.05E-17	1.84E-16
PARG22250	-2.991755428	2.364328288	1.08E-17	1.90E-16
PARG28441	-2.44162819	5.362585729	1.12E-17	1.97E-16
PARG19445	-2.469830484	4.865683818	1.14E-17	2.00E-16
PARG07619	2.473604096	5.141339745	1.17E-17	2.04E-16
PARG03123	-7.911319307	0.039914499	1.17E-17	2.04E-16
PARG05825	-2.795844592	3.741991444	1.30E-17	2.27E-16
PARG11208	-3.445131931	4.356310232	1.35E-17	2.36E-16
PARG28417	-4.145330636	0.689128582	1.46E-17	2.54E-16
PARG01639	-2.865652586	3.497476288	1.47E-17	2.57E-16
PARG27428	3.516678746	2.086253081	1.48E-17	2.57E-16
PARG19887	-2.757360369	2.926556765	1.49E-17	2.59E-16
PARG15367	-3.752420846	2.154186026	1.51E-17	2.63E-16
PARG03479	2.374251481	6.853821275	1.53E-17	2.65E-16
PARG21193	-2.860333616	2.729302357	1.60E-17	2.76E-16
PARG15407	2.387269702	5.838425831	1.72E-17	2.98E-16
PARG19753	-4.290486446	1.588004655	1.75E-17	3.01E-16
PARG13267	-2.37572379	6.95980121	1.77E-17	3.05E-16
PARG20102	2.44467017	4.455700469	1.79E-17	3.08E-16
PARG24265	-3.525509185	2.468739894	1.80E-17	3.11E-16
PARG13879	-3.039009845	2.03947338	1.82E-17	3.13E-16
PARG20603	-2.53448705	3.971524703	1.86E-17	3.19E-16
PARG09308	-5.33545092	1.989054021	2.05E-17	3.51E-16
PARG22450	2.962951778	1.874235576	2.05E-17	3.51E-16
PARG05848	-2.78976192	3.441758835	2.12E-17	3.63E-16

PARG18006	-2.428626139	5.035863553	2.19E-17	3.74E-16
PARG06090	-2.48760482	4.936080184	2.20E-17	3.75E-16
PARG06990	-4.083858348	0.600814859	2.22E-17	3.79E-16
PARG16276	-2.35484657	10.40824656	2.23E-17	3.80E-16
PARG25047	-4.013203228	1.928509116	2.36E-17	4.03E-16
PARG02084	-2.865598572	3.334837659	2.46E-17	4.18E-16
PARG08477	-2.405279598	5.611907702	2.53E-17	4.30E-16
PARG12942	-2.373534962	6.284994242	2.69E-17	4.57E-16
PARG14241	-3.391465121	1.261228928	3.17E-17	5.37E-16
PARG03011	2.479539046	3.949306032	3.25E-17	5.51E-16
PARG06860	2.341721423	8.71542651	3.39E-17	5.73E-16
PARG02716	-2.606533814	3.037060448	3.40E-17	5.74E-16
PARG07341	-2.710159136	4.355510217	3.75E-17	6.33E-16
PARG18008	-3.412498507	2.168779706	3.76E-17	6.34E-16
PARG00411	-2.766744988	2.720148587	3.93E-17	6.62E-16
PARG24508	-2.490732758	5.521564704	3.96E-17	6.67E-16
PARG02287	2.354895213	6.324974497	4.21E-17	7.07E-16
PARG01046	2.353223728	6.62931584	4.23E-17	7.10E-16
PARG18005	-2.983731064	1.57167214	4.32E-17	7.24E-16
PARG23754	-5.989398366	1.788486464	4.45E-17	7.45E-16
PARG19518	-5.982248729	2.674567484	4.45E-17	7.45E-16
PARG19489	-4.248493092	1.543576885	4.58E-17	7.65E-16
PARG01811	-2.616497639	3.290295484	4.83E-17	8.06E-16
PARG23247	-2.652618908	3.55913934	5.10E-17	8.50E-16
PARG26030	-3.694472301	1.941239491	5.18E-17	8.63E-16
PARG07672	-4.644910203	1.034867265	5.20E-17	8.65E-16
PARG30015	2.396161213	6.548153575	5.30E-17	8.82E-16
PARG24983	-2.439084279	4.550307557	5.64E-17	9.37E-16
PARG05769	-4.230606486	1.915410022	5.84E-17	9.69E-16
PARG09349	5.344520798	0.892552326	6.20E-17	1.03E-15
PARG04587	-2.591381165	2.922063199	6.40E-17	1.06E-15
PARG08629	2.647560054	2.311690505	6.83E-17	1.13E-15
PARG05037	2.463481885	4.329543725	6.89E-17	1.14E-15
PARG29481	2.312943066	8.617152263	7.46E-17	1.23E-15
PARG02800	2.321002765	7.318899668	7.57E-17	1.25E-15
PARG21633	-2.314356076	8.024846759	7.72E-17	1.27E-15
PARG23918	-3.38868633	1.761328869	7.73E-17	1.27E-15
PARG23638	-2.833263822	2.901268551	7.84E-17	1.29E-15
PARG27446	2.307428481	8.773176998	8.72E-17	1.43E-15
PARG08292	-2.332873968	6.357083947	9.25E-17	1.52E-15
PARG12944	-2.352870214	5.145672121	9.27E-17	1.52E-15
PARG17812	-3.17473903	2.776095272	9.38E-17	1.54E-15
PARG19092	-2.301289743	9.636169501	9.82E-17	1.61E-15
PARG06481	2.653093666	2.866347278	9.86E-17	1.61E-15
PARG20673	2.299011357	10.97019691	1.01E-16	1.64E-15
PARG10668	2.299619272	9.846666402	1.04E-16	1.69E-15

PARG19894	2.31642206	6.869746991	1.05E-16	1.71E-15
PARG12214	-3.370073758	1.352304956	1.06E-16	1.73E-15
PARG29167	-2.967828929	2.415807358	1.07E-16	1.74E-15
PARG12170	-2.322218083	6.318702768	1.09E-16	1.77E-15
PARG18635	2.315839198	5.80114477	1.12E-16	1.81E-15
PARG15514	-2.460937691	3.543575407	1.14E-16	1.85E-15
PARG22582	4.936664125	0.174255498	1.16E-16	1.88E-15
PARG11889	-2.524181121	3.375308237	1.17E-16	1.89E-15
PARG27360	-3.237587235	3.106201862	1.18E-16	1.90E-15
PARG17857	-5.244577196	0.291450736	1.24E-16	2.00E-15
PARG14498	4.676112713	0.234008334	1.24E-16	2.00E-15
PARG21748	-2.422436778	4.756333893	1.24E-16	2.00E-15
PARG06093	-2.345135549	7.023772119	1.28E-16	2.05E-15
PARG29766	-2.956402552	2.627835439	1.28E-16	2.06E-15
PARG07320	-2.637069811	3.167021061	1.30E-16	2.09E-15
PARG18374	-2.912628724	2.5265921	1.33E-16	2.13E-15
PARG29296	-2.856139738	2.163915716	1.36E-16	2.18E-15
PARG16083	-2.466108467	3.86885929	1.49E-16	2.38E-15
PARG13744	-3.516920093	4.565646275	1.50E-16	2.39E-15
PARG02977	-2.58178104	3.761342839	1.55E-16	2.48E-15
PARG14119	-2.430325499	3.876201311	1.58E-16	2.52E-15
PARG21032	-3.372685052	1.382371537	1.60E-16	2.54E-15
PARG22034	3.094077958	2.146817559	1.61E-16	2.56E-15
PARG24348	2.3658693	4.82155446	1.65E-16	2.62E-15
PARG12701	-2.530282535	3.771183664	1.71E-16	2.72E-15
PARG19446	-3.815936214	1.180169778	1.72E-16	2.74E-15
PARG04484	-2.322501156	6.313933307	1.77E-16	2.80E-15
PARG08509	-2.597579927	3.254115997	1.79E-16	2.84E-15
PARG02894	-2.283870587	7.759561472	1.84E-16	2.91E-15
PARG04483	2.585758068	2.396441327	1.85E-16	2.92E-15
PARG07092	-2.338869793	5.684380449	1.88E-16	2.96E-15
PARG02889	2.884969673	4.256543409	2.00E-16	3.16E-15
PARG11660	2.718556112	1.469125788	2.01E-16	3.17E-15
PARG19790	2.390669572	4.395458722	2.18E-16	3.43E-15
PARG02232	-2.328430664	5.264905668	2.27E-16	3.57E-15
PARG25669	-2.63101674	4.084640207	2.28E-16	3.58E-15
PARG03832	-3.43045019	4.874835429	2.30E-16	3.61E-15
PARG10672	-2.630933481	3.129918672	2.42E-16	3.79E-15
PARG00143	-2.323131423	4.656032267	2.44E-16	3.82E-15
PARG15861	-2.280252418	7.08030409	2.48E-16	3.88E-15
PARG27774	-4.577020979	2.196413383	2.56E-16	4.01E-15
PARG26833	-2.302973129	6.712738012	2.57E-16	4.02E-15
PARG08665	-2.315121626	5.797062159	2.63E-16	4.10E-15
PARG12945	2.27684695	6.951198915	2.80E-16	4.37E-15
PARG14147	-2.454642915	3.563776278	2.86E-16	4.45E-15
PARG18586	-2.406560292	3.824812343	3.03E-16	4.71E-15

PARG17097	-3.287532474	2.430612895	3.18E-16	4.95E-15
PARG18540	-2.562748928	3.082752375	3.39E-16	5.27E-15
PARG23340	-7.749729097	1.240921979	3.46E-16	5.35E-15
PARG07343	-7.748449418	0.676408459	3.46E-16	5.35E-15
PARG11809	-2.478869353	3.595620615	3.91E-16	6.05E-15
PARG23930	-2.324637589	4.492843366	3.94E-16	6.10E-15
PARG16082	-2.502227007	3.489894668	3.98E-16	6.15E-15
PARG23186	-3.399843612	1.962246586	4.02E-16	6.20E-15
PARG05810	2.304937796	5.376628741	4.06E-16	6.26E-15
PARG29354	-2.497790912	3.575143288	4.07E-16	6.27E-15
PARG11593	-4.479073216	1.984512252	4.23E-16	6.50E-15
PARG15675	4.052047399	0.385207212	4.23E-16	6.50E-15
PARG12536	-2.272920879	6.294889645	4.34E-16	6.66E-15
PARG03461	-2.941372257	2.470437219	4.38E-16	6.72E-15
PARG30410	-2.837612971	2.045997084	4.45E-16	6.82E-15
PARG18426	2.245699328	9.974123612	4.51E-16	6.90E-15
PARG27198	-2.474022656	3.207108436	5.00E-16	7.64E-15
PARG01549	4.305588216	-0.074542392	5.13E-16	7.84E-15
PARG12330	2.255902559	6.602556106	5.44E-16	8.31E-15
PARG26138	2.257340983	6.605546478	5.59E-16	8.53E-15
PARG26457	-5.158982071	1.091400591	5.83E-16	8.89E-15
PARG29738	2.243458045	6.932543214	6.33E-16	9.63E-15
PARG07872	-3.404661691	1.852378968	6.47E-16	9.85E-15
PARG23967	-2.531446779	3.579381473	6.52E-16	9.91E-15
PARG05980	2.420255262	4.335110303	6.59E-16	1.00E-14
PARG26295	-4.739881246	2.314869365	6.91E-16	1.05E-14
PARG28530	-3.925183001	1.171708193	7.01E-16	1.06E-14
PARG19507	-2.314379493	4.996299929	7.14E-16	1.08E-14
PARG15337	-2.448781724	2.92435738	7.40E-16	1.12E-14
PARG23085	-3.227461172	1.870963623	7.62E-16	1.15E-14
PARG06697	2.358057163	3.892692859	7.68E-16	1.16E-14
PARG23910	-3.667606329	1.43395363	8.10E-16	1.22E-14
PARG08440	2.265229057	6.318213685	8.23E-16	1.24E-14
PARG26928	-2.236146432	6.809988659	8.54E-16	1.29E-14
PARG06621	-2.371078568	4.303406109	8.92E-16	1.34E-14
PARG07336	-4.104571648	1.600065468	9.04E-16	1.36E-14
PARG22839	-2.264390195	4.973853176	9.24E-16	1.39E-14
PARG23999	-2.230599048	6.621336878	9.79E-16	1.47E-14
PARG29923	-4.43912314	1.481940937	9.95E-16	1.49E-14
PARG18142	-5.129263853	0.139584072	1.09E-15	1.64E-14
PARG19284	2.289550973	4.949957785	1.12E-15	1.67E-14
PARG11332	2.321074159	5.337327257	1.14E-15	1.71E-14
PARG08138	-5.825327577	1.449004385	1.15E-15	1.72E-14
PARG08787	3.73812097	0.445186798	1.17E-15	1.74E-14
PARG16047	-2.823875902	2.698666202	1.18E-15	1.76E-14
PARG24041	2.253125819	5.506919954	1.19E-15	1.77E-14

PARG05912	-2.285022926	5.253932024	1.20E-15	1.79E-14
PARG21093	-2.277126592	6.157327745	1.22E-15	1.81E-14
PARG29461	2.276663352	4.783487708	1.25E-15	1.85E-14
PARG08088	-2.218067804	7.808786714	1.29E-15	1.91E-14
PARG14052	-2.71778603	1.892054992	1.34E-15	1.99E-14
PARG12343	-2.257049171	5.649681598	1.37E-15	2.03E-14
PARG29765	-2.739549085	2.64742808	1.38E-15	2.04E-14
PARG15175	2.360484486	4.035618275	1.50E-15	2.22E-14
PARG16757	-2.214315865	6.903565273	1.58E-15	2.33E-14
PARG08377	-2.265147035	5.50217593	1.59E-15	2.35E-14
PARG07251	2.202197229	8.441186226	1.67E-15	2.46E-14
PARG17457	-2.633942439	2.976463862	1.68E-15	2.46E-14
PARG27567	-4.706185875	1.604277315	1.70E-15	2.50E-14
PARG27337	-4.701628307	1.474583682	1.70E-15	2.50E-14
PARG27639	-2.297745736	4.40871562	1.71E-15	2.51E-14
PARG23518	-2.375734718	3.974851529	1.72E-15	2.52E-14
PARG15504	-3.086518048	1.855730723	1.76E-15	2.58E-14
PARG07303	2.475293262	4.528784792	1.83E-15	2.67E-14
PARG16192	2.529266541	3.146378133	1.95E-15	2.85E-14
PARG19542	2.251340346	5.1779259	2.02E-15	2.95E-14
PARG03710	-3.05360975	2.245118727	2.03E-15	2.96E-14
PARG02091	-2.701516646	2.187237237	2.03E-15	2.96E-14
PARG07840	2.373766804	2.745929077	2.10E-15	3.06E-14
PARG13380	2.199313406	8.144457297	2.12E-15	3.09E-14
PARG11970	-3.702962147	1.73577138	2.13E-15	3.09E-14
PARG27710	-2.283374584	4.607525072	2.18E-15	3.16E-14
PARG29491	-4.474189575	3.096619655	2.30E-15	3.33E-14
PARG00284	-4.471628205	1.322064341	2.30E-15	3.33E-14
PARG29568	-4.681030943	2.058386198	2.31E-15	3.34E-14
PARG13683	-3.077455028	3.619196697	2.42E-15	3.51E-14
PARG29971	-2.192071161	7.498547938	2.46E-15	3.55E-14
PARG24696	-3.794162407	1.397899431	2.48E-15	3.58E-14
PARG05821	-2.313380648	3.822363145	2.64E-15	3.81E-14
PARG20430	-2.290212839	4.936655127	2.74E-15	3.95E-14
PARG01047	2.299622942	4.812522158	2.80E-15	4.03E-14
PARG04591	4.515342661	-0.6682106	2.84E-15	4.09E-14
PARG18820	-2.210856103	5.868253779	3.00E-15	4.32E-14
PARG26693	-2.219957672	5.65890394	3.03E-15	4.35E-14
PARG09413	3.2561059	0.749785001	3.10E-15	4.45E-14
PARG19997	3.046688055	2.348215947	3.16E-15	4.53E-14
PARG24125	2.38737291	3.696060989	3.20E-15	4.59E-14
PARG28246	2.443983225	2.260454238	3.23E-15	4.62E-14
PARG19579	2.178948546	7.397033361	3.27E-15	4.67E-14
PARG18437	-2.303027068	3.98091908	3.29E-15	4.70E-14
PARG07571	2.576632658	2.35290335	3.60E-15	5.14E-14
PARG14659	-3.513079336	2.120109942	3.74E-15	5.33E-14

PARG27950	2.191532448	5.975410135	3.82E-15	5.45E-14
PARG11365	-3.252441488	1.592358584	3.90E-15	5.54E-14
PARG02856	3.097153716	0.650683062	3.90E-15	5.54E-14
PARG01347	2.346459206	2.297009156	3.90E-15	5.54E-14
PARG17643	-2.229900945	4.268922532	4.02E-15	5.71E-14
PARG12532	2.170898414	7.331987724	4.04E-15	5.74E-14
PARG18266	-7.610236774	-0.661950078	4.17E-15	5.91E-14
PARG22697	-2.479586503	3.860062968	4.42E-15	6.26E-14
PARG26704	-2.320750162	4.36582098	4.81E-15	6.80E-14
PARG23353	-2.292170956	4.702296872	4.83E-15	6.82E-14
PARG21934	-3.767065191	1.412608678	5.13E-15	7.24E-14
PARG19307	-2.205568855	5.903198804	5.29E-15	7.46E-14
PARG09573	-2.21851082	4.525113381	5.36E-15	7.55E-14
PARG01970	-5.052702595	1.806749358	5.42E-15	7.64E-14
PARG19074	2.176887132	6.766837901	5.57E-15	7.84E-14
PARG01955	2.192778383	5.80465017	5.60E-15	7.88E-14
PARG20570	-2.205604032	5.924986593	5.68E-15	7.97E-14
PARG12665	-2.174906498	6.588776331	6.00E-15	8.42E-14
PARG16942	-2.152976628	7.557910268	6.06E-15	8.49E-14
PARG13445	-2.163030859	7.805778855	6.11E-15	8.56E-14
PARG16038	2.459956019	2.042097083	6.34E-15	8.88E-14
PARG07691	-2.151839183	7.986211711	6.46E-15	9.04E-14
PARG14528	-2.79101413	2.244110215	7.03E-15	9.82E-14
PARG06353	-5.044987602	1.809902458	7.51E-15	1.05E-13
PARG09192	-2.345013168	5.016134317	7.64E-15	1.07E-13
PARG03096	-2.143261174	8.320604044	7.73E-15	1.08E-13
PARG19263	2.184309595	5.363192558	7.74E-15	1.08E-13
PARG28231	2.2215621	5.283249939	7.89E-15	1.10E-13
PARG24752	-4.624854244	1.835484491	7.90E-15	1.10E-13
PARG25372	-2.750115945	2.072999017	8.04E-15	1.12E-13
PARG25878	2.640086553	1.708767293	8.04E-15	1.12E-13
PARG29259	-2.144410379	7.549405957	8.10E-15	1.12E-13
PARG11998	-2.206542085	5.683271077	8.30E-15	1.15E-13
PARG21200	2.178406536	5.619531083	8.33E-15	1.15E-13
PARG23037	2.818614814	2.035041331	8.50E-15	1.17E-13
PARG02314	2.95796361	3.406811144	8.61E-15	1.19E-13
PARG19782	-7.581671602	0.507326196	8.66E-15	1.19E-13
PARG15000	-3.542582708	3.619398694	8.98E-15	1.24E-13
PARG06080	-2.632326149	3.084383374	9.23E-15	1.27E-13
PARG16665	2.479377423	3.926347675	9.36E-15	1.29E-13
PARG29421	-2.36847831	4.109783592	9.43E-15	1.30E-13
PARG19735	2.292977191	4.132236625	9.80E-15	1.35E-13
PARG07308	-2.224686561	4.469487283	1.00E-14	1.38E-13
PARG28130	-2.410762089	3.214085997	1.03E-14	1.41E-13
PARG18526	-5.019672608	0.727585393	1.04E-14	1.43E-13
PARG08600	-2.978720218	2.467750273	1.04E-14	1.43E-13

PARG23184	-2.369197361	2.309450878	1.05E-14	1.44E-13
PARG16151	-4.611041982	2.247999966	1.08E-14	1.47E-13
PARG18194	-2.671577561	3.209859983	1.08E-14	1.47E-13
PARG20044	3.702592844	0.693081701	1.09E-14	1.49E-13
PARG08469	2.144197865	6.696659603	1.10E-14	1.50E-13
PARG19177	-2.390513607	3.781658605	1.11E-14	1.51E-13
PARG07854	-2.309757574	3.881042266	1.14E-14	1.55E-13
PARG05741	-2.146996515	7.074924449	1.17E-14	1.59E-13
PARG11964	-2.19497746	4.959923742	1.17E-14	1.59E-13
PARG23388	2.819942825	1.963405066	1.22E-14	1.66E-13
PARG00819	-5.239831548	2.164834303	1.25E-14	1.70E-13
PARG10459	4.704583722	0.433868935	1.25E-14	1.70E-13
PARG25363	-2.843758213	2.735686599	1.26E-14	1.71E-13
PARG24970	-2.415898218	2.996398389	1.28E-14	1.73E-13
PARG27665	-2.494408497	2.672981546	1.37E-14	1.86E-13
PARG19361	-3.482775658	2.342442477	1.41E-14	1.90E-13
PARG29366	-3.568069123	0.651485728	1.43E-14	1.92E-13
PARG06716	-2.85025132	0.738359683	1.54E-14	2.07E-13
PARG18534	-2.358967187	3.560782472	1.58E-14	2.13E-13
PARG05785	-2.228819763	5.036873526	1.60E-14	2.15E-13
PARG27385	2.121105172	6.812555639	1.62E-14	2.18E-13
PARG06949	-2.283965851	4.536934735	1.67E-14	2.25E-13
PARG25037	2.124570721	6.455890039	1.68E-14	2.25E-13
PARG22828	3.418859653	0.78422229	1.75E-14	2.35E-13
PARG26407	-2.715543714	2.569045904	1.76E-14	2.36E-13
PARG00802	-6.220160723	-0.208297177	1.81E-14	2.43E-13
PARG24120	3.673870847	1.807822557	1.89E-14	2.53E-13
PARG25241	-2.284426676	3.398792479	1.98E-14	2.64E-13
PARG08429	-3.240110376	2.451014098	2.05E-14	2.74E-13
PARG02782	-2.108498446	7.553102607	2.06E-14	2.75E-13
PARG08575	-2.125581794	5.670241332	2.10E-14	2.80E-13
PARG15447	-3.642393928	1.64003614	2.18E-14	2.90E-13
PARG07651	-3.359345027	1.425049007	2.19E-14	2.92E-13
PARG14104	-3.092190462	1.215092762	2.34E-14	3.11E-13
PARG07752	-2.534459857	2.572881225	2.36E-14	3.14E-13
PARG10145	-2.310803971	8.59979686	2.39E-14	3.18E-13
PARG24201	2.097413855	10.03640817	2.40E-14	3.19E-13
PARG18854	2.212940125	4.268876888	2.49E-14	3.29E-13
PARG06751	2.496883012	2.909392396	2.51E-14	3.32E-13
PARG06342	2.163319772	5.966243148	2.51E-14	3.32E-13
PARG01366	-3.30893299	0.463540216	2.53E-14	3.34E-13
PARG18549	-2.945282581	1.870600946	2.63E-14	3.47E-13
PARG13515	-2.163893058	5.477660361	2.74E-14	3.61E-13
PARG16512	-2.497391048	2.255514227	2.87E-14	3.79E-13
PARG27780	-3.125494101	0.913921356	3.01E-14	3.96E-13
PARG19162	-2.138329548	5.487798268	3.01E-14	3.96E-13

PARG07815	-2.18236353	4.85725264	3.05E-14	4.01E-13
PARG22686	2.096390007	7.068440012	3.05E-14	4.01E-13
PARG17765	-2.794197542	0.540703701	3.10E-14	4.07E-13
PARG28436	-2.091045623	8.58558227	3.15E-14	4.13E-13
PARG03749	-3.101664598	1.24169723	3.16E-14	4.15E-13
PARG11808	-2.929098096	1.723557888	3.27E-14	4.28E-13
PARG02718	-2.134317772	5.804849644	3.41E-14	4.46E-13
PARG00564	-4.267235493	1.131410867	3.44E-14	4.49E-13
PARG01990	-2.713252781	2.377874968	3.60E-14	4.71E-13
PARG03808	-2.362280405	2.264905076	3.65E-14	4.76E-13
PARG08735	-2.099503687	7.440224195	3.67E-14	4.78E-13
PARG19444	3.388035784	0.275958774	3.70E-14	4.82E-13
PARG04146	-2.140439142	4.889710115	3.74E-14	4.86E-13
PARG23917	-2.651139613	2.46785716	3.83E-14	4.98E-13
PARG05450	2.711931538	1.673604552	3.84E-14	4.99E-13
PARG23987	-2.753989656	1.649568659	3.90E-14	5.06E-13
PARG26876	2.228888195	4.25521698	4.05E-14	5.26E-13
PARG27613	2.079604772	8.713429923	4.08E-14	5.29E-13
PARG21796	2.079123447	7.924075106	4.20E-14	5.44E-13
PARG03555	-2.099379494	6.168655343	4.28E-14	5.54E-13
PARG29406	-2.733933163	2.802023999	4.43E-14	5.73E-13
PARG08215	-3.651553173	0.03577299	4.78E-14	6.17E-13
PARG06929	-2.214865001	4.862844844	4.89E-14	6.32E-13
PARG19002	2.213537535	3.540065959	4.92E-14	6.34E-13
PARG21025	4.627367951	1.026803772	5.10E-14	6.58E-13
PARG15121	2.161230287	4.11541542	5.12E-14	6.59E-13
PARG21351	-2.200201118	4.430347964	5.12E-14	6.59E-13
PARG30394	2.198463767	5.042736226	5.12E-14	6.59E-13
PARG30381	-4.01688295	1.565286054	5.33E-14	6.84E-13
PARG07079	-7.481409425	1.473381232	5.60E-14	7.18E-13
PARG13602	-2.727592146	1.889384682	5.60E-14	7.18E-13
PARG21064	-2.252853254	3.356681154	5.78E-14	7.40E-13
PARG06916	-3.460951434	1.437618608	5.84E-14	7.47E-13
PARG06711	-2.647066922	3.132696454	6.10E-14	7.81E-13
PARG20636	-4.22440031	1.64265532	6.33E-14	8.08E-13
PARG15350	-4.039724601	1.418371256	6.33E-14	8.08E-13
PARG11227	2.188796837	4.304295388	6.43E-14	8.21E-13
PARG24664	-3.141698151	0.978757421	6.46E-14	8.24E-13
PARG18625	2.073828334	5.961417986	6.57E-14	8.38E-13
PARG12166	-2.497874651	3.007299628	6.89E-14	8.77E-13
PARG16406	2.089987614	5.160648783	6.90E-14	8.77E-13
PARG14232	2.231543232	2.546765919	7.07E-14	8.99E-13
PARG02907	2.060971043	8.052426415	7.15E-14	9.08E-13
PARG04361	-4.515053116	1.133451446	7.21E-14	9.15E-13
PARG24919	-5.60071973	1.607493154	7.29E-14	9.24E-13
PARG01433	2.092367435	4.805893126	7.32E-14	9.27E-13

PARG21396	-2.291531109	3.032349467	7.42E-14	9.39E-13
PARG14270	2.073091868	6.243750997	7.47E-14	9.45E-13
PARG16624	-2.123938333	4.375568882	7.58E-14	9.58E-13
PARG24765	2.485927989	2.467046391	7.90E-14	9.98E-13
PARG19664	-3.700430954	1.794899252	7.95E-14	1.00E-12
PARG21807	-2.063371788	6.49927069	7.96E-14	1.00E-12
PARG18642	-2.648607044	2.705874127	8.15E-14	1.03E-12
PARG14429	-2.895497023	1.244502571	8.30E-14	1.05E-12
PARG00008	-2.254875261	4.717016027	8.55E-14	1.08E-12
PARG08690	3.788876163	0.327117192	8.60E-14	1.08E-12
PARG20400	-2.257288297	3.335575082	8.68E-14	1.09E-12
PARG23452	-2.071410749	6.71898999	8.89E-14	1.12E-12
PARG23931	-2.439674119	2.151011823	9.12E-14	1.14E-12
PARG22836	-2.051126097	7.36934794	9.30E-14	1.17E-12
PARG00462	-2.060657915	6.405961924	9.44E-14	1.18E-12
PARG27180	2.09322821	5.74800369	9.93E-14	1.24E-12
PARG16686	4.146051692	1.823506638	9.95E-14	1.24E-12
PARG27669	4.139377851	-0.16803836	9.95E-14	1.24E-12
PARG01511	2.049704525	7.393703617	1.00E-13	1.25E-12
PARG27545	-2.756302853	2.38885577	1.01E-13	1.26E-12
PARG11714	-3.736989263	0.817183677	1.04E-13	1.30E-12
PARG09016	2.038081004	12.73331873	1.07E-13	1.33E-12
PARG09011	-4.890171741	0.634832469	1.08E-13	1.34E-12
PARG14709	-4.885330641	1.4484185	1.08E-13	1.34E-12
PARG06792	-2.120359626	4.422409776	1.10E-13	1.37E-12
PARG28029	-2.387949399	3.327969852	1.11E-13	1.38E-12
PARG08607	-2.241287998	3.853323602	1.15E-13	1.42E-12
PARG06966	-2.878534678	1.486026818	1.16E-13	1.43E-12
PARG27695	2.100575293	5.308962584	1.20E-13	1.48E-12
PARG12969	-7.436353679	0.086360237	1.20E-13	1.48E-12
PARG03514	-7.424903054	0.567117012	1.20E-13	1.48E-12
PARG22384	2.097322675	5.204963922	1.25E-13	1.55E-12
PARG19900	-2.615582983	2.977362538	1.31E-13	1.61E-12
PARG08801	2.089811616	4.573045496	1.33E-13	1.64E-12
PARG18221	-3.719470303	0.297195722	1.36E-13	1.67E-12
PARG18152	-2.116295012	4.174463877	1.45E-13	1.79E-12
PARG30223	-2.184820033	3.85111114	1.48E-13	1.82E-12
PARG00580	2.040395982	6.851422898	1.49E-13	1.83E-12
PARG26919	-5.558330613	0.336938187	1.50E-13	1.84E-12
PARG06435	-2.290767705	3.821402443	1.50E-13	1.84E-12
PARG12569	4.304558683	0.554373374	1.52E-13	1.86E-12
PARG05770	-4.188315047	1.877796404	1.60E-13	1.96E-12
PARG16220	-2.031945828	7.279270576	1.69E-13	2.07E-12
PARG19135	2.070305484	5.144709366	1.70E-13	2.08E-12
PARG14083	-3.445551094	2.109998451	1.70E-13	2.08E-12
PARG14041	2.184820226	3.40574133	1.75E-13	2.14E-12

PARG04886	-7.416341735	1.267582964	1.76E-13	2.15E-12
PARG13271	-2.12213278	4.237314974	1.79E-13	2.18E-12
PARG22004	-2.069463563	4.927305082	1.79E-13	2.19E-12
PARG07817	2.652353796	1.39189084	1.84E-13	2.24E-12
PARG25128	2.077622484	4.872071025	1.86E-13	2.26E-12
PARG04104	-4.454695327	1.161746835	1.91E-13	2.32E-12
PARG16467	-4.45161509	0.585939696	1.91E-13	2.32E-12
PARG14073	-2.067115635	4.784244602	1.91E-13	2.32E-12
PARG05624	-2.458778786	4.588069614	1.92E-13	2.32E-12
PARG05736	2.489909965	1.658027168	2.18E-13	2.65E-12
PARG18491	-2.02271099	6.785642811	2.19E-13	2.65E-12
PARG12448	-2.070773143	5.48024165	2.22E-13	2.68E-12
PARG25425	2.059644019	5.214908377	2.23E-13	2.70E-12
PARG11152	-2.879831674	2.256872735	2.28E-13	2.75E-12
PARG06382	-2.109463389	4.912901256	2.29E-13	2.76E-12
PARG15524	-2.103461657	4.29996539	2.32E-13	2.80E-12
PARG27578	-3.94328475	1.097272267	2.34E-13	2.82E-12
PARG01102	-2.031593001	6.410157099	2.34E-13	2.82E-12
PARG26666	2.357058654	2.287151371	2.41E-13	2.90E-12
PARG13766	2.125848316	3.905519095	2.51E-13	3.02E-12
PARG27419	-2.390813674	3.090799168	2.55E-13	3.07E-12
PARG06771	-7.384366144	-0.608679613	2.60E-13	3.12E-12
PARG06130	4.900552246	-0.011398462	2.60E-13	3.12E-12
PARG28013	-2.423283623	2.382978074	2.62E-13	3.14E-12
PARG26910	2.02825251	5.89127024	2.63E-13	3.15E-12
PARG10733	-2.011107274	7.481020469	2.75E-13	3.29E-12
PARG06740	-2.100064775	4.55346048	2.84E-13	3.40E-12
PARG12287	-2.002211546	9.106313428	2.88E-13	3.45E-12
PARG18231	2.003671091	8.335327406	2.92E-13	3.49E-12
PARG00515	-4.83576474	1.01134133	3.02E-13	3.60E-12
PARG06758	2.017357721	5.954279996	3.05E-13	3.63E-12
PARG07757	2.049050277	5.409029516	3.09E-13	3.68E-12
PARG22911	-5.527224899	0.711946559	3.09E-13	3.69E-12
PARG08591	-2.02247148	6.261324448	3.10E-13	3.69E-12
PARG07395	2.002261268	7.648399542	3.10E-13	3.69E-12
PARG07700	1.99622631	10.70378157	3.17E-13	3.77E-12
PARG07507	2.155676424	4.03007106	3.19E-13	3.79E-12
PARG21215	-2.003381397	7.923384669	3.22E-13	3.83E-12
PARG26029	2.06737782	4.902130194	3.23E-13	3.83E-12
PARG12308	-2.476957507	2.427787654	3.23E-13	3.83E-12
PARG06380	2.019742917	5.670980413	3.35E-13	3.97E-12
PARG08065	-2.225557435	3.149014841	3.50E-13	4.14E-12
PARG00305	1.99720976	7.890956301	3.70E-13	4.37E-12
PARG26356	-2.222451674	3.6671821	3.71E-13	4.38E-12
PARG06128	-2.088716168	4.411043279	3.73E-13	4.41E-12
PARG00429	2.087286452	3.796496676	3.75E-13	4.42E-12

PARG13661	-7.367000811	-0.308189578	3.84E-13	4.52E-12
PARG02283	4.873541232	0.018285105	3.84E-13	4.52E-12
PARG19348	1.988530001	10.36663074	3.90E-13	4.59E-12
PARG15291	-2.521980936	2.079541295	3.90E-13	4.59E-12
PARG12538	-2.158585167	3.444258651	3.91E-13	4.59E-12
PARG25398	-4.125567627	1.387157629	4.08E-13	4.79E-12
PARG14154	-3.212368215	1.701197501	4.10E-13	4.81E-12
PARG27803	-2.374823896	3.142572715	4.46E-13	5.22E-12
PARG11446	-5.504005104	-0.527158223	4.47E-13	5.23E-12
PARG16210	-5.499743736	0.888277923	4.47E-13	5.23E-12
PARG18612	-2.127688509	4.097431728	4.64E-13	5.42E-12
PARG10602	-2.000702172	6.469849138	4.64E-13	5.42E-12
PARG27938	1.996227946	6.701252292	4.68E-13	5.46E-12
PARG29427	-1.982753766	9.228802411	4.74E-13	5.53E-12
PARG01265	-2.531339827	2.229493379	4.83E-13	5.63E-12
PARG02384	2.303109973	2.617569411	4.93E-13	5.74E-12
PARG04305	2.118503719	3.945538485	4.95E-13	5.76E-12
PARG29726	-2.083645814	4.889819436	4.96E-13	5.77E-12
PARG09463	2.981679664	2.694560162	5.04E-13	5.86E-12
PARG26366	-2.149852748	3.824815191	5.10E-13	5.93E-12
PARG16841	-4.409790131	1.659496539	5.11E-13	5.93E-12
PARG03046	2.054609589	4.569610761	5.38E-13	6.24E-12
PARG00076	-2.156370267	4.09891424	5.54E-13	6.42E-12
PARG23354	-4.118517321	1.276373246	5.60E-13	6.49E-12
PARG09150	-3.513510261	0.398518929	5.67E-13	6.56E-12
PARG22368	2.556164354	2.256853966	5.79E-13	6.69E-12
PARG00201	1.974858298	7.869194265	6.00E-13	6.94E-12
PARG29547	-1.987609782	6.779668264	6.23E-13	7.20E-12
PARG19055	-3.076718646	3.656820119	6.28E-13	7.24E-12
PARG13864	-5.487950266	1.222640915	6.46E-13	7.45E-12
PARG01856	-2.080070072	4.244721835	6.47E-13	7.45E-12
PARG11389	-3.643361476	0.568825111	6.79E-13	7.82E-12
PARG15330	-2.031720918	5.090569181	6.80E-13	7.82E-12
PARG18122	-2.546041389	2.109976457	6.92E-13	7.96E-12
PARG18400	2.797137397	0.805505324	7.09E-13	8.15E-12
PARG13134	2.108149946	4.369127624	7.17E-13	8.23E-12
PARG20025	1.987957115	5.651499448	7.29E-13	8.37E-12
PARG02016	-2.010099664	4.796420823	7.31E-13	8.38E-12
PARG21541	1.970065901	7.181311756	7.33E-13	8.40E-12
PARG18566	-1.974419738	6.782734863	7.35E-13	8.42E-12
PARG03045	1.979998968	6.135065936	7.66E-13	8.76E-12
PARG00174	-1.966552507	7.844652394	7.67E-13	8.76E-12
PARG24279	-3.088679929	1.759629558	7.77E-13	8.87E-12
PARG29053	-1.987049877	6.055978885	8.03E-13	9.16E-12
PARG01333	-2.139593628	4.046780835	8.05E-13	9.18E-12
PARG22508	2.593838677	1.399026837	8.25E-13	9.41E-12

PARG25104	-2.140841941	3.987333988	8.88E-13	1.01E-11
PARG21676	2.08548501	3.802504997	9.43E-13	1.07E-11
PARG11502	2.090969928	3.923511475	9.73E-13	1.11E-11
PARG02231	2.229926297	4.136228227	9.75E-13	1.11E-11
PARG11361	-4.368004906	-0.117121014	9.95E-13	1.13E-11
PARG06816	1.958703252	6.992841449	1.03E-12	1.16E-11
PARG27039	-2.19879259	3.64195293	1.13E-12	1.28E-11
PARG29144	-2.646958194	0.801628499	1.15E-12	1.30E-11
PARG22251	-2.500202573	1.930667406	1.17E-12	1.32E-11
PARG19139	-2.482248788	1.548332824	1.17E-12	1.33E-11
PARG18623	-2.021834503	5.340843054	1.22E-12	1.38E-11
PARG06903	-1.955118835	6.895256995	1.23E-12	1.39E-11
PARG19403	2.079671105	3.679056581	1.34E-12	1.51E-11
PARG22181	2.055774698	4.706089585	1.34E-12	1.51E-11
PARG02884	2.049872873	3.909332542	1.34E-12	1.52E-11
PARG02981	-2.39849844	1.748775411	1.37E-12	1.54E-11
PARG26897	-2.213559077	3.13853811	1.41E-12	1.59E-11
PARG21287	-1.936245965	10.33016875	1.42E-12	1.60E-11
PARG24912	2.157698235	3.367168957	1.43E-12	1.61E-11
PARG27820	-2.914221532	2.688810909	1.43E-12	1.61E-11
PARG22201	-2.15072517	4.230822529	1.45E-12	1.63E-11
PARG23925	-2.502075494	1.451398671	1.49E-12	1.67E-11
PARG00474	-2.940160755	1.010196129	1.49E-12	1.68E-11
PARG09143	-2.223559989	2.827081238	1.52E-12	1.70E-11
PARG18724	-3.603836233	1.12827592	1.54E-12	1.72E-11
PARG12452	3.311492161	0.985908126	1.54E-12	1.72E-11
PARG27801	-2.165741133	4.389644001	1.58E-12	1.76E-11
PARG17944	1.968559488	5.504760743	1.60E-12	1.79E-11
PARG16593	2.984013627	1.837547075	1.65E-12	1.85E-11
PARG11083	-2.039813289	4.235032047	1.66E-12	1.85E-11
PARG11306	1.93674879	7.382851464	1.66E-12	1.85E-11
PARG17653	1.97126573	5.0982217	1.67E-12	1.86E-11
PARG20408	-1.945604663	6.504004287	1.68E-12	1.87E-11
PARG11110	-2.020997778	4.456940963	1.70E-12	1.89E-11
PARG07972	1.982084143	6.257582483	1.70E-12	1.89E-11
PARG03686	-1.979301064	5.129303865	1.73E-12	1.92E-11
PARG29143	-1.99122888	4.814395098	1.73E-12	1.92E-11
PARG24697	-7.081061976	-0.060007433	1.76E-12	1.95E-11
PARG24002	-7.079166323	-0.176917498	1.76E-12	1.95E-11
PARG02280	2.104188362	3.335970297	1.79E-12	1.98E-11
PARG22751	-1.956087238	5.177206152	1.81E-12	2.00E-11
PARG22653	-2.926791216	0.508989939	1.83E-12	2.02E-11
PARG12962	2.19644567	2.977554313	1.84E-12	2.03E-11
PARG11774	-3.837235211	0.972594486	1.97E-12	2.18E-11
PARG29151	-2.200854858	3.60680758	1.97E-12	2.18E-11
PARG21818	2.019544468	4.782586591	1.98E-12	2.18E-11

PARG25632	-5.413560162	0.536616074	1.98E-12	2.18E-11
PARG13081	-4.048689849	1.244677999	2.01E-12	2.22E-11
PARG03357	2.628381919	1.08431298	2.10E-12	2.31E-11
PARG01165	2.088570562	2.152073866	2.14E-12	2.35E-11
PARG23500	2.066928689	4.112573846	2.21E-12	2.43E-11
PARG05880	-1.93182435	6.755736672	2.26E-12	2.48E-11
PARG30016	-2.12695282	3.638798857	2.35E-12	2.58E-11
PARG17831	-2.956822015	1.321504846	2.37E-12	2.60E-11
PARG18390	-1.933910882	6.233600139	2.38E-12	2.60E-11
PARG13060	1.915974163	9.455603902	2.38E-12	2.60E-11
PARG01084	1.949805139	6.424421706	2.45E-12	2.68E-11
PARG19063	2.011114075	4.49320579	2.59E-12	2.84E-11
PARG23594	1.933580654	6.797253323	2.64E-12	2.89E-11
PARG18618	3.000370339	3.706026946	2.65E-12	2.89E-11
PARG06490	7.107585202	0.489309173	2.72E-12	2.96E-11
PARG13990	-7.053933669	-0.71640183	2.72E-12	2.96E-11
PARG03156	-7.047162625	-0.062027102	2.72E-12	2.96E-11
PARG29201	-7.244940492	-0.15272976	2.80E-12	3.05E-11
PARG06007	-7.234303326	0.831638046	2.80E-12	3.05E-11
PARG11022	-2.025063042	4.530326519	2.85E-12	3.10E-11
PARG02042	2.303038758	3.010881959	2.85E-12	3.10E-11
PARG23548	-5.394427115	-0.405839873	2.89E-12	3.14E-11
PARG29156	-2.351115325	3.780226703	2.90E-12	3.15E-11
PARG14153	-2.582550842	4.324102709	2.90E-12	3.15E-11
PARG11868	-2.030987999	5.022469338	2.93E-12	3.17E-11
PARG24382	1.942188382	5.927407866	2.93E-12	3.18E-11
PARG24377	-2.023767208	4.238001701	2.97E-12	3.21E-11
PARG02482	-1.982331917	4.704423233	2.97E-12	3.22E-11
PARG19334	-2.086775784	3.199965077	2.99E-12	3.23E-11
PARG24271	-3.454869476	1.110031902	3.06E-12	3.30E-11
PARG14202	1.91053681	7.881166369	3.08E-12	3.32E-11
PARG02837	2.002312788	5.716504285	3.11E-12	3.35E-11
PARG11477	-2.334770691	2.203743865	3.11E-12	3.35E-11
PARG28010	-2.081002893	3.039041804	3.12E-12	3.36E-11
PARG25349	1.921152804	6.332469912	3.14E-12	3.38E-11
PARG14264	1.9067848	7.86534269	3.38E-12	3.63E-11
PARG20582	-2.577113529	2.529675524	3.40E-12	3.66E-11
PARG23461	-1.98703619	3.583565211	3.43E-12	3.68E-11
PARG05099	-2.645940804	2.388416522	3.46E-12	3.71E-11
PARG07604	-2.442610254	1.986059109	3.59E-12	3.85E-11
PARG27271	-2.007846612	4.07986731	3.62E-12	3.89E-11
PARG27496	1.899903781	9.036202405	3.64E-12	3.90E-11
PARG07447	1.905043587	7.267486425	3.71E-12	3.97E-11
PARG11194	3.623364989	0.827435176	3.85E-12	4.12E-11
PARG13443	-2.496496912	2.760356007	3.94E-12	4.21E-11
PARG14123	-1.919196988	5.469574105	4.06E-12	4.34E-11

PARG29550	-2.165298835	3.409375129	4.10E-12	4.38E-11
PARG19916	-2.205179658	2.417696427	4.16E-12	4.44E-11
PARG02504	-2.126461076	2.752254776	4.21E-12	4.49E-11
PARG14211	-7.037200949	0.504614203	4.22E-12	4.49E-11
PARG27312	-3.09694464	0.357643495	4.26E-12	4.53E-11
PARG01088	-3.335884976	2.038842881	4.42E-12	4.71E-11
PARG08964	-2.085252582	2.953940181	4.52E-12	4.81E-11
PARG19223	1.938339458	5.120797963	4.61E-12	4.90E-11
PARG15318	1.950897073	5.210779451	4.63E-12	4.92E-11
PARG09009	1.895168487	7.402792666	4.76E-12	5.05E-11
PARG24077	-3.24635363	0.332188845	4.80E-12	5.09E-11
PARG09305	1.920102261	5.655085715	4.85E-12	5.15E-11
PARG18597	1.922860924	5.43676743	4.97E-12	5.26E-11
PARG07152	1.945160076	4.521180217	5.13E-12	5.43E-11
PARG11817	-1.980860485	5.180134471	5.27E-12	5.57E-11
PARG18382	1.919224051	5.932420614	5.48E-12	5.79E-11
PARG26737	2.002167572	4.956207236	5.88E-12	6.21E-11
PARG21406	-1.890395992	6.34294906	5.89E-12	6.22E-11
PARG13726	3.385029328	1.071544431	6.15E-12	6.48E-11
PARG29289	-5.342523786	1.815413937	6.19E-12	6.52E-11
PARG02920	-5.333941344	0.653529039	6.19E-12	6.52E-11
PARG19659	-2.009608308	3.517590717	6.29E-12	6.62E-11
PARG11707	-1.971301589	4.395487561	6.33E-12	6.66E-11
PARG20498	-2.501018708	2.798011256	6.53E-12	6.87E-11
PARG13718	-6.9861402	0.329471832	6.56E-12	6.87E-11
PARG13797	-6.982279734	-0.5697773	6.56E-12	6.87E-11
PARG28101	5.300028089	2.188905858	6.56E-12	6.87E-11
PARG25631	5.279030517	1.096433088	6.56E-12	6.87E-11
PARG24701	-1.886614475	7.020174916	6.56E-12	6.87E-11
PARG14835	-2.101591545	2.946491957	6.61E-12	6.92E-11
PARG08447	-1.929877191	5.196474169	6.76E-12	7.08E-11
PARG04905	-1.92883102	4.939559883	6.79E-12	7.10E-11
PARG17415	-2.342569069	2.690198452	6.90E-12	7.21E-11
PARG24340	-1.929130397	4.652395436	7.08E-12	7.39E-11
PARG09049	1.89205543	6.077391587	7.08E-12	7.39E-11
PARG10046	-4.258134978	1.137890036	7.65E-12	7.98E-11
PARG27025	-4.238585525	0.932235585	7.65E-12	7.98E-11
PARG23762	-1.914598111	5.249913755	7.75E-12	8.07E-11
PARG08630	-2.03879002	3.395533446	7.80E-12	8.12E-11
PARG06659	1.875119374	6.80118846	7.88E-12	8.20E-11
PARG15448	1.923328991	5.276805295	7.99E-12	8.31E-11
PARG27327	3.227029533	2.404255064	8.17E-12	8.48E-11
PARG28319	3.218457282	1.412377032	8.17E-12	8.48E-11
PARG12609	-1.87002265	7.563468433	8.23E-12	8.54E-11
PARG18532	-1.907493432	5.403156251	8.33E-12	8.63E-11
PARG06565	-2.549133148	1.773207742	8.42E-12	8.72E-11

PARG26441	-2.075230491	3.618182913	8.79E-12	9.10E-11
PARG08986	-2.203739875	2.690197135	8.97E-12	9.29E-11
PARG06502	2.06459889	3.642631074	8.99E-12	9.30E-11
PARG05524	4.333292642	0.779498962	9.10E-12	9.40E-11
PARG02356	2.488775433	3.291666737	9.12E-12	9.42E-11
PARG29344	-3.747398045	1.180715372	9.41E-12	9.71E-11
PARG26450	-7.1639902	1.477180706	9.53E-12	9.83E-11
PARG17082	2.321867983	1.641681323	9.57E-12	9.87E-11
PARG05528	1.859615972	9.722505709	9.76E-12	1.00E-10
PARG03711	1.877673311	6.166752152	9.95E-12	1.02E-10
PARG26601	-6.978961295	0.076677822	1.02E-11	1.05E-10
PARG11521	1.969381007	3.832467824	1.04E-11	1.07E-10
PARG20597	-2.420322866	1.091445244	1.05E-11	1.08E-10
PARG01432	1.985509072	2.84065974	1.07E-11	1.09E-10
PARG20585	-2.108786512	3.580685784	1.07E-11	1.10E-10
PARG20919	2.613808533	0.210372914	1.08E-11	1.10E-10
PARG07371	-3.503323002	-0.003371455	1.08E-11	1.11E-10
PARG03639	-2.060772583	2.922251531	1.10E-11	1.13E-10
PARG17351	-1.975267387	3.45933257	1.15E-11	1.18E-10
PARG12753	-2.001738684	3.580855558	1.18E-11	1.20E-10
PARG08463	1.893227747	5.203591714	1.20E-11	1.23E-10
PARG07210	-2.126087718	1.958243342	1.21E-11	1.23E-10
PARG07006	1.871702982	5.658085256	1.21E-11	1.24E-10
PARG00200	-2.00549309	4.087466896	1.24E-11	1.27E-10
PARG04167	-1.939166466	3.943740968	1.25E-11	1.27E-10
PARG05720	-1.961644096	3.761630636	1.26E-11	1.29E-10
PARG20145	-3.279430144	1.911134566	1.26E-11	1.29E-10
PARG21350	-1.854567293	7.350500216	1.27E-11	1.30E-10
PARG02451	1.858220837	7.180812674	1.28E-11	1.30E-10
PARG21220	-1.888271523	5.430033099	1.28E-11	1.30E-10
PARG07385	-1.903175502	5.293987959	1.29E-11	1.31E-10
PARG23988	-5.291978917	1.212852807	1.34E-11	1.36E-10
PARG00987	2.022169793	2.62031017	1.39E-11	1.41E-10
PARG12620	-1.864344608	6.310237923	1.39E-11	1.41E-10
PARG10517	-2.524081486	1.980183146	1.39E-11	1.41E-10
PARG11065	-2.371202347	2.368165134	1.40E-11	1.41E-10
PARG25715	-1.900199971	4.333193894	1.43E-11	1.44E-10
PARG27693	-7.151359768	-0.243096708	1.44E-11	1.45E-10
PARG24195	-7.141484092	-0.914481866	1.44E-11	1.45E-10
PARG28453	-7.130937844	1.883439267	1.44E-11	1.45E-10
PARG14745	-7.125715832	0.61151031	1.44E-11	1.45E-10
PARG00191	-2.934757752	1.532513681	1.44E-11	1.46E-10
PARG14121	-2.81706452	0.096250082	1.44E-11	1.46E-10
PARG29803	1.843492498	8.240088184	1.46E-11	1.47E-10
PARG23552	2.009739106	3.739473524	1.48E-11	1.49E-10
PARG01759	1.888241801	5.45645993	1.52E-11	1.52E-10

PARG16227	-4.605631129	1.905852005	1.52E-11	1.53E-10
PARG16634	-1.878691796	5.121896887	1.57E-11	1.58E-10
PARG19328	1.890813167	5.0918006	1.57E-11	1.58E-10
PARG10601	-1.849280878	6.781225004	1.58E-11	1.59E-10
PARG24615	-2.160357774	3.930342804	1.65E-11	1.66E-10
PARG01840	-2.729574662	0.594651319	1.65E-11	1.66E-10
PARG20609	2.02034004	2.934564804	1.66E-11	1.66E-10
PARG12046	1.890508724	5.248362296	1.67E-11	1.67E-10
PARG16821	1.844671068	6.749829647	1.71E-11	1.71E-10
PARG20092	-3.413215005	0.861536863	1.74E-11	1.74E-10
PARG23921	-4.007215405	0.571666482	1.78E-11	1.77E-10
PARG15526	-2.004857795	3.435004744	1.80E-11	1.79E-10
PARG13085	1.908406846	4.808228827	1.80E-11	1.80E-10
PARG10394	2.831728494	1.089335182	1.82E-11	1.81E-10
PARG02173	-1.84993395	6.27359899	1.89E-11	1.88E-10
PARG12209	-3.471801222	0.729255797	1.91E-11	1.90E-10
PARG19103	-2.203747732	2.271844669	1.95E-11	1.94E-10
PARG21864	-2.65738823	0.827918653	1.98E-11	1.97E-10
PARG17925	-1.911348589	4.750718702	1.99E-11	1.98E-10
PARG20506	-2.158518238	3.613739007	2.04E-11	2.03E-10
PARG03914	1.835956906	7.369039416	2.06E-11	2.04E-10
PARG17840	1.871650657	5.536727176	2.08E-11	2.06E-10
PARG07647	-2.040936906	2.245588243	2.10E-11	2.08E-10
PARG19286	-2.805281841	1.161426452	2.13E-11	2.11E-10
PARG24150	-7.116271589	-0.64173343	2.19E-11	2.16E-10
PARG24149	-7.114352614	-0.1080843	2.19E-11	2.16E-10
PARG13980	-7.104993225	-0.768281448	2.19E-11	2.16E-10
PARG02269	-3.151596716	2.295897953	2.22E-11	2.20E-10
PARG17761	-2.171008221	1.335752326	2.27E-11	2.24E-10
PARG17250	-3.598493338	-0.300799374	2.33E-11	2.30E-10
PARG21015	-2.052103055	2.422699276	2.36E-11	2.33E-10
PARG16127	1.82435171	7.548598445	2.39E-11	2.35E-10
PARG02688	1.874235741	3.424955099	2.40E-11	2.36E-10
PARG19533	-1.955333379	7.017536159	2.41E-11	2.38E-10
PARG18354	-2.107813483	3.054778299	2.42E-11	2.38E-10
PARG08096	-2.400684315	1.88179898	2.44E-11	2.40E-10
PARG13920	2.14890007	2.23935357	2.47E-11	2.43E-10
PARG19279	1.910727133	4.31960871	2.48E-11	2.43E-10
PARG23022	-2.104709177	3.076191633	2.48E-11	2.43E-10
PARG07472	-6.9172414	-0.245765119	2.52E-11	2.47E-10
PARG11216	-2.544866808	1.87453798	2.57E-11	2.51E-10
PARG20333	1.864844235	5.270665871	2.57E-11	2.52E-10
PARG12237	1.824276646	7.134613514	2.57E-11	2.52E-10
PARG24308	-1.844913388	5.892911414	2.64E-11	2.59E-10
PARG06391	-2.625330042	1.427687547	2.67E-11	2.61E-10
PARG07432	2.556329625	1.88795123	2.67E-11	2.61E-10

PARG04568	-3.331878034	3.146497246	2.70E-11	2.63E-10
PARG25743	1.852657965	5.203457358	2.74E-11	2.67E-10
PARG04347	-2.735412148	1.098347049	2.77E-11	2.70E-10
PARG27547	1.816529974	8.338016007	2.79E-11	2.72E-10
PARG07721	-1.99102749	2.901275095	2.83E-11	2.76E-10
PARG23223	1.821838905	7.066952838	2.89E-11	2.81E-10
PARG22059	2.525620351	2.243574187	2.94E-11	2.86E-10
PARG00952	2.360195441	2.100172598	2.96E-11	2.88E-10
PARG04026	-1.814954143	7.941702999	3.04E-11	2.95E-10
PARG26047	2.03759128	2.339537152	3.06E-11	2.97E-10
PARG19386	-1.808771104	9.765601424	3.09E-11	3.00E-10
PARG09816	-2.736709532	2.625871203	3.12E-11	3.02E-10
PARG19646	1.824734603	6.168121517	3.17E-11	3.07E-10
PARG19938	4.001111281	-0.072866412	3.18E-11	3.08E-10
PARG16306	-2.832077019	1.516379184	3.23E-11	3.13E-10
PARG01835	1.80656034	9.044397741	3.34E-11	3.23E-10
PARG24908	-2.033706879	3.18381484	3.34E-11	3.23E-10
PARG07898	-2.739493617	1.933686945	3.34E-11	3.23E-10
PARG19350	3.538699898	0.426482939	3.38E-11	3.26E-10
PARG07631	1.859607754	5.059984754	3.39E-11	3.27E-10
PARG02240	-1.813243708	6.777401083	3.56E-11	3.43E-10
PARG02245	-2.958528998	0.328634746	3.63E-11	3.49E-10
PARG07527	-2.430710555	2.442558393	3.63E-11	3.49E-10
PARG02706	1.808650367	7.854439226	3.65E-11	3.51E-10
PARG23838	-1.845795751	5.850051258	3.67E-11	3.53E-10
PARG10708	-1.974741873	4.356117311	3.76E-11	3.61E-10
PARG27457	-1.850840873	4.714737042	4.11E-11	3.94E-10
PARG04601	2.026242748	1.679781095	4.13E-11	3.96E-10
PARG12345	-1.920998182	3.816989286	4.15E-11	3.98E-10
PARG22519	2.0726018	2.506760754	4.21E-11	4.04E-10
PARG08264	-2.240824301	3.010625397	4.22E-11	4.05E-10
PARG05317	1.828039516	5.557381466	4.24E-11	4.06E-10
PARG03659	1.800851694	7.189192434	4.29E-11	4.11E-10
PARG09040	4.208204569	-0.623300038	4.34E-11	4.15E-10
PARG06642	-2.816924148	1.868870386	4.44E-11	4.24E-10
PARG02932	3.122713283	0.08431386	4.51E-11	4.31E-10
PARG00087	-1.797991008	8.341367484	4.55E-11	4.34E-10
PARG04134	-1.87944457	5.223573305	4.56E-11	4.35E-10
PARG23157	-2.952685381	-0.09276775	4.57E-11	4.36E-10
PARG17519	3.522970669	-0.246091111	4.66E-11	4.44E-10
PARG09070	-1.934473841	4.137551228	4.76E-11	4.54E-10
PARG29422	-1.967445393	4.515943578	4.78E-11	4.55E-10
PARG06746	-2.106937646	2.029847293	4.78E-11	4.55E-10
PARG04831	3.021923774	0.353911886	4.80E-11	4.57E-10
PARG04387	1.801816791	7.077798793	4.82E-11	4.58E-10
PARG12115	-3.027907008	2.087615239	4.83E-11	4.59E-10

PARG19676	1.80185275	6.55601873	4.88E-11	4.63E-10
PARG29447	-2.378278254	2.220708933	4.93E-11	4.68E-10
PARG02353	-2.151672838	2.071947784	5.21E-11	4.93E-10
PARG03087	-1.787153173	9.80048066	5.23E-11	4.95E-10
PARG11903	-3.723498069	1.499370259	5.26E-11	4.98E-10
PARG12006	-1.799528594	6.643808769	5.38E-11	5.09E-10
PARG16746	-1.856416306	4.39433674	5.45E-11	5.16E-10
PARG29770	-1.827452648	6.244297829	5.47E-11	5.17E-10
PARG06586	3.634414476	1.451474988	5.52E-11	5.21E-10
PARG26357	1.797672893	6.482584361	5.60E-11	5.29E-10
PARG02343	-1.943734787	3.646572664	5.71E-11	5.39E-10
PARG08740	-1.838705641	4.716519613	5.73E-11	5.40E-10
PARG07500	-1.84795911	5.979898124	5.83E-11	5.49E-10
PARG06198	-1.824886798	4.932416978	5.87E-11	5.53E-10
PARG01175	-2.221561185	3.115053957	5.93E-11	5.58E-10
PARG15887	-2.969095561	1.551758534	6.11E-11	5.75E-10
PARG14077	-1.963466985	3.71935222	6.15E-11	5.79E-10
PARG08411	1.826586981	4.881508848	6.19E-11	5.81E-10
PARG27167	-2.296032969	1.323951049	6.38E-11	5.99E-10
PARG04530	-2.082684586	1.710880415	6.41E-11	6.01E-10
PARG12399	-3.635709656	0.876632294	6.45E-11	6.04E-10
PARG06118	-3.632451922	0.640832566	6.45E-11	6.04E-10
PARG13910	-5.197424216	0.740530741	6.46E-11	6.04E-10
PARG17968	-5.195819238	1.900462821	6.46E-11	6.04E-10
PARG21735	-2.240366895	6.386504356	6.51E-11	6.09E-10
PARG08361	1.784632226	6.930717291	6.56E-11	6.13E-10
PARG29409	-1.850300572	4.642103569	6.70E-11	6.26E-10
PARG09033	-4.377042066	0.800361933	6.72E-11	6.28E-10
PARG24559	2.00491326	4.228174027	6.76E-11	6.31E-10
PARG18357	1.793127491	7.274809404	6.87E-11	6.41E-10
PARG00431	-1.917907192	3.505396755	6.94E-11	6.47E-10
PARG20177	1.777915377	9.068280744	7.00E-11	6.52E-10
PARG29160	1.784878326	7.109120699	7.07E-11	6.58E-10
PARG22823	-3.456193389	0.900870026	7.18E-11	6.68E-10
PARG11948	-1.908275082	5.749786766	7.19E-11	6.69E-10
PARG08646	1.775347731	7.916708262	7.35E-11	6.83E-10
PARG15530	-1.815603984	5.799323947	7.37E-11	6.84E-10
PARG05781	-3.535239011	2.703955267	7.70E-11	7.15E-10
PARG24151	2.223828932	1.048927538	7.81E-11	7.25E-10
PARG21434	1.884615339	3.825951546	7.85E-11	7.28E-10
PARG26862	1.779560639	7.687556732	7.85E-11	7.28E-10
PARG00161	-2.202009159	2.415630817	7.88E-11	7.29E-10
PARG06367	-2.503760564	1.649879631	7.91E-11	7.32E-10
PARG08621	-2.16138156	3.3887544	8.03E-11	7.42E-10
PARG15349	1.780025236	6.496993696	8.08E-11	7.47E-10
PARG20147	-2.201217701	2.463101386	8.35E-11	7.72E-10

PARG07570	-1.851976473	4.187210185	8.55E-11	7.90E-10
PARG23338	-1.990990227	2.946671181	8.62E-11	7.95E-10
PARG02105	1.829754283	4.805190562	8.66E-11	7.98E-10
PARG24973	-1.778726955	6.527568838	8.66E-11	7.98E-10
PARG18441	1.833385717	4.939367045	8.71E-11	8.02E-10
PARG11314	1.831779688	3.304737113	8.77E-11	8.08E-10
PARG26402	-3.505737721	1.106144755	8.94E-11	8.23E-10
PARG27612	-2.409126387	2.252747543	8.96E-11	8.24E-10
PARG12603	3.767198337	0.995776621	8.98E-11	8.26E-10
PARG26614	-1.772607534	6.88661328	9.00E-11	8.27E-10
PARG04438	-1.866461761	4.184820441	9.03E-11	8.29E-10
PARG27269	-1.851706834	6.227942537	9.08E-11	8.33E-10
PARG13432	1.772704053	6.730470769	9.39E-11	8.61E-10
PARG24675	1.765869412	7.916894663	9.56E-11	8.76E-10
PARG23241	-5.174366257	2.686559051	9.62E-11	8.81E-10
PARG23800	3.940379217	-0.288202186	9.80E-11	8.97E-10
PARG22134	1.932149616	2.163063113	9.85E-11	9.01E-10
PARG19969	-6.809295113	-0.743563653	9.98E-11	9.12E-10
PARG00025	-6.807257154	0.796167418	9.98E-11	9.12E-10
PARG08956	-1.759881784	7.963268819	1.05E-10	9.56E-10
PARG06970	-1.789224327	5.232431932	1.06E-10	9.65E-10
PARG03124	-3.0697968	0.209986012	1.06E-10	9.71E-10
PARG29970	-1.77771457	5.279373839	1.08E-10	9.88E-10
PARG18999	-1.805959705	4.563974417	1.14E-10	1.04E-09
PARG25052	-2.022597191	3.484035877	1.14E-10	1.04E-09
PARG14047	-2.011130661	2.545946922	1.14E-10	1.04E-09
PARG27772	1.762243857	9.091796253	1.16E-10	1.05E-09
PARG03136	-1.829907707	4.277497821	1.17E-10	1.06E-09
PARG06211	1.857167419	3.536280252	1.17E-10	1.06E-09
PARG08746	1.768527225	5.853467179	1.22E-10	1.11E-09
PARG01758	1.763839635	6.584421698	1.24E-10	1.13E-09
PARG17792	-2.238882515	1.938744668	1.25E-10	1.13E-09
PARG10115	-1.847879716	3.602965705	1.29E-10	1.17E-09
PARG21842	1.761850714	6.165866752	1.31E-10	1.19E-09
PARG19819	1.749394108	8.147501767	1.32E-10	1.19E-09
PARG20716	-3.632197258	1.460967471	1.35E-10	1.22E-09
PARG23732	-2.167840762	2.719157053	1.35E-10	1.22E-09
PARG14079	-1.838587341	4.691447897	1.39E-10	1.26E-09
PARG24611	-1.825091813	4.395618656	1.44E-10	1.30E-09
PARG27449	-5.138936401	0.788789982	1.44E-10	1.30E-09
PARG10374	4.135078868	0.410119636	1.44E-10	1.30E-09
PARG27321	-3.354724313	0.570766425	1.44E-10	1.30E-09
PARG02429	-1.760006012	6.925695839	1.46E-10	1.32E-09
PARG24901	1.749722456	6.969037357	1.47E-10	1.33E-09
PARG04888	-1.76377585	6.179713615	1.50E-10	1.35E-09
PARG13747	-3.77192201	2.780092692	1.54E-10	1.38E-09

PARG00778	-1.748287572	6.954508031	1.57E-10	1.41E-09
PARG19990	-2.398142955	1.356011833	1.57E-10	1.41E-09
PARG06436	1.75604569	5.871254531	1.59E-10	1.42E-09
PARG09234	-1.852855154	4.022356025	1.59E-10	1.42E-09
PARG27696	-1.744974205	7.031894905	1.59E-10	1.43E-09
PARG19691	-6.77511447	0.167545855	1.59E-10	1.43E-09
PARG08406	-6.758125536	0.806603805	1.59E-10	1.43E-09
PARG18195	-2.706424127	2.136054108	1.63E-10	1.46E-09
PARG26820	2.643624774	1.468452408	1.63E-10	1.46E-09
PARG06883	-1.745074022	7.458898677	1.64E-10	1.47E-09
PARG00850	-1.738968036	8.245063804	1.64E-10	1.47E-09
PARG15117	-1.758722498	6.00764475	1.66E-10	1.48E-09
PARG23764	-1.815143096	4.186650819	1.66E-10	1.48E-09
PARG26965	2.3814082	0.474093995	1.66E-10	1.49E-09
PARG05059	-2.824458167	1.759261562	1.75E-10	1.56E-09
PARG24946	1.965605019	2.656338966	1.78E-10	1.59E-09
PARG04363	1.732607601	10.14863508	1.79E-10	1.60E-09
PARG06508	-1.79207193	4.926204974	1.87E-10	1.66E-09
PARG11997	-2.002452181	2.540496982	1.90E-10	1.69E-09
PARG16256	-3.214276139	1.984482322	1.91E-10	1.70E-09
PARG22819	-3.061243622	0.801737099	1.91E-10	1.70E-09
PARG02991	1.788673292	5.472905189	1.97E-10	1.75E-09
PARG15263	-2.142547052	3.334927841	1.99E-10	1.76E-09
PARG02685	-1.831930039	3.927731719	2.00E-10	1.78E-09
PARG01916	-1.974270114	3.257112021	2.02E-10	1.79E-09
PARG18891	2.173731661	3.130795196	2.03E-10	1.80E-09
PARG27405	2.037960261	2.367292892	2.05E-10	1.81E-09
PARG20118	1.729990052	7.933826069	2.05E-10	1.81E-09
PARG21712	-1.742568767	6.382233628	2.06E-10	1.83E-09
PARG00098	1.737598958	6.261694872	2.13E-10	1.89E-09
PARG04075	-5.121715109	0.614003509	2.15E-10	1.90E-09
PARG14351	-1.734809774	7.376114487	2.17E-10	1.91E-09
PARG03339	1.725406362	9.241325031	2.17E-10	1.92E-09
PARG16384	-2.681463109	0.926458493	2.17E-10	1.92E-09
PARG20785	2.39822508	0.929062306	2.19E-10	1.93E-09
PARG26423	-1.902451264	2.874065055	2.25E-10	1.98E-09
PARG02154	-1.894541989	3.976308616	2.25E-10	1.98E-09
PARG03593	1.722302344	8.650429048	2.38E-10	2.09E-09
PARG06900	1.724733394	7.704528556	2.39E-10	2.10E-09
PARG11252	-1.943676936	2.818865797	2.41E-10	2.12E-09
PARG05541	1.720552162	9.403138156	2.43E-10	2.13E-09
PARG06809	1.730120263	6.961035249	2.47E-10	2.17E-09
PARG18226	-6.743495424	-0.935540274	2.55E-10	2.24E-09
PARG00343	-1.773217601	4.527917674	2.56E-10	2.25E-09
PARG21074	1.736597743	6.129831039	2.62E-10	2.30E-09
PARG04898	1.747625233	5.555158079	2.62E-10	2.30E-09

PARG11661	1.76561896	3.86229958	2.64E-10	2.31E-09
PARG10578	-2.081027095	3.197908943	2.70E-10	2.36E-09
PARG19105	2.022761186	2.743317911	2.70E-10	2.36E-09
PARG07707	1.720570085	7.396408848	2.76E-10	2.41E-09
PARG11813	-2.800308474	2.002974281	2.77E-10	2.42E-09
PARG27901	-4.957101062	0.716109264	2.83E-10	2.47E-09
PARG20587	2.422996216	1.417627362	2.86E-10	2.50E-09
PARG28185	1.75659172	4.047911641	2.89E-10	2.52E-09
PARG12553	1.748882495	5.255541833	2.91E-10	2.54E-09
PARG09190	-2.053357094	3.400504723	2.97E-10	2.59E-09
PARG28779	1.886105185	8.459262788	2.98E-10	2.59E-09
PARG09978	-1.942161001	2.721015384	2.98E-10	2.60E-09
PARG12258	2.13361172	1.197720115	3.01E-10	2.62E-09
PARG12782	-1.722053151	7.050052573	3.03E-10	2.64E-09
PARG29747	-1.719364749	7.137747471	3.05E-10	2.65E-09
PARG01031	-3.015086975	0.855844975	3.08E-10	2.67E-09
PARG13429	1.888278295	3.408586302	3.10E-10	2.70E-09
PARG04445	-2.451333996	1.580972701	3.12E-10	2.71E-09
PARG27015	-1.863536411	3.343420879	3.13E-10	2.71E-09
PARG06887	-1.938553212	2.346369466	3.21E-10	2.78E-09
PARG20580	-1.95660825	3.46212701	3.22E-10	2.79E-09
PARG05773	-5.098658398	0.761678722	3.24E-10	2.80E-09
PARG00666	1.771088765	4.309516226	3.29E-10	2.85E-09
PARG15613	-3.52520354	-0.101664881	3.34E-10	2.89E-09
PARG01832	-1.777230906	4.370939104	3.37E-10	2.91E-09
PARG18751	-1.720966899	5.625407316	3.37E-10	2.91E-09
PARG27874	1.718009684	6.323283444	3.38E-10	2.91E-09
PARG25409	-1.793712088	4.022306552	3.38E-10	2.92E-09
PARG01809	-2.419137805	1.106178451	3.38E-10	2.92E-09
PARG00710	-2.105875289	2.604695605	3.43E-10	2.95E-09
PARG20296	1.77286873	4.269768763	3.51E-10	3.03E-09
PARG25231	1.739666541	5.480993559	3.52E-10	3.04E-09
PARG28165	1.726525038	5.632091367	3.56E-10	3.07E-09
PARG02090	-1.714619103	6.549164243	3.59E-10	3.09E-09
PARG23951	-1.748591609	4.430901827	3.60E-10	3.10E-09
PARG07480	-2.691546188	1.157707584	3.66E-10	3.15E-09
PARG04471	1.702974975	8.515095371	3.73E-10	3.20E-09
PARG01032	1.711278057	7.342753394	3.76E-10	3.23E-09
PARG08783	1.822430955	3.622446226	3.80E-10	3.26E-09
PARG16178	-1.862048224	3.007863747	3.86E-10	3.31E-09
PARG06885	-1.929485116	2.242363068	3.88E-10	3.33E-09
PARG13219	-2.983659575	1.319534159	4.02E-10	3.45E-09
PARG23224	-1.703103086	9.097944029	4.08E-10	3.49E-09
PARG22247	-6.710561996	-0.1822106	4.09E-10	3.50E-09
PARG15147	4.998249836	-0.040876529	4.09E-10	3.50E-09
PARG21216	-1.739730089	6.500826314	4.09E-10	3.50E-09

PARG05788	1.711633148	6.402452981	4.11E-10	3.51E-09
PARG07292	-1.705658814	6.504011611	4.13E-10	3.53E-09
PARG20994	2.881644101	0.230543768	4.28E-10	3.65E-09
PARG23938	-3.728193722	-0.041544648	4.35E-10	3.71E-09
PARG25444	1.867523938	2.975067219	4.47E-10	3.81E-09
PARG21872	1.70734975	5.921655864	4.62E-10	3.93E-09
PARG10222	-1.876255461	3.224836399	4.67E-10	3.97E-09
PARG15880	1.798241168	3.993467022	4.70E-10	4.00E-09
PARG10748	3.211987396	2.071207502	4.80E-10	4.08E-09
PARG27201	3.116497676	1.201723664	4.80E-10	4.08E-09
PARG06036	1.695650608	7.483123368	4.82E-10	4.10E-09
PARG29684	-1.696401784	7.66520982	4.84E-10	4.11E-09
PARG25633	-5.066739275	0.359975882	4.87E-10	4.13E-09
PARG26618	-1.789467401	5.232021037	4.90E-10	4.16E-09
PARG19026	-2.059808628	1.588290404	4.94E-10	4.18E-09
PARG13600	-1.701194695	6.336914501	4.94E-10	4.18E-09
PARG16638	-2.301322064	3.181628697	4.98E-10	4.22E-09
PARG02702	1.854047187	3.421297927	5.00E-10	4.23E-09
PARG10840	1.705309079	6.355279972	5.15E-10	4.35E-09
PARG10466	-1.69949451	6.180345348	5.37E-10	4.54E-09
PARG16505	1.685165202	8.010268291	5.62E-10	4.75E-09
PARG11404	-3.040989695	1.320399817	5.65E-10	4.77E-09
PARG13543	2.504889082	0.938107115	5.74E-10	4.84E-09
PARG09233	-2.040232674	2.478081276	5.79E-10	4.88E-09
PARG19018	1.694878548	6.610505277	5.81E-10	4.90E-09
PARG19940	1.714101263	5.462216445	5.93E-10	4.99E-09
PARG26376	-1.784729009	3.723070408	5.95E-10	5.01E-09
PARG20656	-1.685975579	7.364919651	5.97E-10	5.02E-09
PARG09146	-3.223412571	-0.22373688	6.00E-10	5.05E-09
PARG07773	-2.994867201	0.272743401	6.00E-10	5.05E-09
PARG19064	-1.689918657	7.271683524	6.04E-10	5.07E-09
PARG20809	1.686600955	6.975190321	6.13E-10	5.15E-09
PARG07106	-1.931747322	2.478897295	6.15E-10	5.16E-09
PARG07857	-3.702544761	-0.174839735	6.18E-10	5.18E-09
PARG19732	-3.697089071	0.622383363	6.18E-10	5.18E-09
PARG26621	-2.274632367	1.179051789	6.31E-10	5.28E-09
PARG12040	-3.271498198	2.730865445	6.31E-10	5.28E-09
PARG15458	2.968562866	0.794127406	6.31E-10	5.28E-09
PARG07758	1.680123007	8.753831743	6.35E-10	5.32E-09
PARG00914	1.698278066	6.177030907	6.36E-10	5.32E-09
PARG19947	-1.747225097	4.514277361	6.46E-10	5.40E-09
PARG19425	-1.750583674	4.512954099	6.53E-10	5.45E-09
PARG11902	3.031845153	-0.41926348	6.54E-10	5.46E-09
PARG17053	-6.674961232	0.493413952	6.59E-10	5.50E-09
PARG30101	-1.674307708	9.897831997	6.66E-10	5.55E-09
PARG11388	1.811884769	4.526182455	6.67E-10	5.56E-09

PARG27720	1.698188489	5.460901805	6.70E-10	5.58E-09
PARG23516	-1.692287255	6.333275883	6.74E-10	5.61E-09
PARG15284	1.754681484	4.636312192	6.78E-10	5.64E-09
PARG14381	-2.764004482	1.725948258	6.79E-10	5.64E-09
PARG18383	-4.878620323	0.325691108	6.79E-10	5.64E-09
PARG15618	-4.869629427	-0.042068914	6.79E-10	5.64E-09
PARG11828	1.691360835	6.501917664	6.79E-10	5.64E-09
PARG24633	-1.726409316	4.783856128	6.84E-10	5.68E-09
PARG12197	-1.966179343	5.915539186	6.91E-10	5.73E-09
PARG12019	-1.699381204	5.576333855	7.06E-10	5.86E-09
PARG27216	-2.580293146	1.579162179	7.09E-10	5.88E-09
PARG27893	-1.676007696	8.217620283	7.21E-10	5.97E-09
PARG18369	1.677046387	7.588285312	7.23E-10	5.99E-09
PARG00546	-2.059728523	2.392466215	7.24E-10	5.99E-09
PARG02252	-1.806917437	4.563724118	7.31E-10	6.05E-09
PARG24380	-1.682887	7.549189453	7.33E-10	6.06E-09
PARG04989	-1.834095681	3.233956679	7.37E-10	6.09E-09
PARG27641	-2.182908032	2.294274289	7.57E-10	6.25E-09
PARG24784	1.726055571	4.947019802	7.63E-10	6.30E-09
PARG23148	1.681409224	6.489090835	7.96E-10	6.56E-09
PARG27568	-3.946486275	-0.557633697	7.98E-10	6.58E-09
PARG04359	-3.136087469	1.550430624	8.01E-10	6.60E-09
PARG27085	1.678524646	6.918990302	8.08E-10	6.65E-09
PARG19419	2.074215024	2.808777481	8.60E-10	7.08E-09
PARG09186	-1.783414587	3.54778098	8.62E-10	7.09E-09
PARG07360	1.667031221	8.007678699	8.73E-10	7.18E-09
PARG26627	-3.669235	0.495434983	8.79E-10	7.22E-09
PARG03496	1.711777576	4.905304578	8.80E-10	7.23E-09
PARG00467	-1.748039997	4.223121572	8.82E-10	7.24E-09
PARG11414	-2.661116733	1.271562367	8.89E-10	7.30E-09
PARG00266	-2.46533339	2.572072674	8.93E-10	7.32E-09
PARG01289	-1.66607148	7.744459932	9.00E-10	7.38E-09
PARG20108	2.817800159	1.077603114	9.04E-10	7.41E-09
PARG14309	-1.703260891	4.818138498	9.06E-10	7.42E-09
PARG27263	3.34024975	0.13897947	9.14E-10	7.48E-09
PARG02244	-1.662249331	8.630853396	9.19E-10	7.52E-09
PARG06132	1.958617131	2.686797916	9.36E-10	7.65E-09
PARG06041	1.665977015	7.20855235	9.43E-10	7.71E-09
PARG27864	-1.711767806	5.298117805	9.44E-10	7.71E-09
PARG21794	-1.664908302	7.683691052	9.48E-10	7.74E-09
PARG27935	1.751969372	4.319590517	9.51E-10	7.76E-09
PARG07832	1.759004337	2.677517999	9.55E-10	7.79E-09
PARG04899	1.672113257	6.189651652	9.80E-10	7.98E-09
PARG22331	-4.342117744	0.079639204	9.84E-10	8.02E-09
PARG00011	-3.013742604	0.307572048	9.87E-10	8.04E-09
PARG23551	2.583742647	1.050294433	9.95E-10	8.10E-09

PARG21529	-2.038018721	2.302281603	1.00E-09	8.14E-09
PARG29551	-1.691173484	5.591540247	1.01E-09	8.25E-09
PARG26791	-1.909694339	3.049687558	1.01E-09	8.25E-09
PARG13114	-1.726384961	4.054528987	1.04E-09	8.47E-09
PARG05333	-4.844042653	2.658891624	1.06E-09	8.59E-09
PARG18272	-1.802935226	4.375946804	1.06E-09	8.62E-09
PARG26906	1.669797394	6.016770389	1.07E-09	8.64E-09
PARG11249	-6.628431094	-0.500316476	1.07E-09	8.64E-09
PARG24763	-6.611026875	0.441533649	1.07E-09	8.64E-09
PARG10342	-6.605560027	-0.259262023	1.07E-09	8.64E-09
PARG21576	1.659016959	7.357995876	1.07E-09	8.68E-09
PARG15760	1.705257457	4.552279856	1.07E-09	8.69E-09
PARG13502	-2.289672828	1.797690425	1.08E-09	8.69E-09
PARG18697	-1.66168354	7.184054886	1.08E-09	8.72E-09
PARG00374	-1.845392453	2.955019558	1.09E-09	8.81E-09
PARG08315	1.730224205	4.251720992	1.10E-09	8.85E-09
PARG00039	1.747035414	3.965222172	1.11E-09	8.99E-09
PARG27296	-2.630912993	0.977764182	1.13E-09	9.07E-09
PARG27771	-1.976016718	1.783829373	1.13E-09	9.09E-09
PARG19393	-1.817324249	4.149109822	1.15E-09	9.24E-09
PARG12714	-3.055729103	0.737493029	1.15E-09	9.25E-09
PARG01566	2.919267099	0.797450977	1.15E-09	9.25E-09
PARG25161	-3.923193302	0.391148379	1.16E-09	9.30E-09
PARG13266	-1.653610201	7.865993136	1.16E-09	9.36E-09
PARG01127	2.197369254	0.797364167	1.18E-09	9.47E-09
PARG15955	-1.658886338	7.119464285	1.18E-09	9.50E-09
PARG00050	2.786818321	1.125830659	1.19E-09	9.52E-09
PARG22684	-1.783583862	3.325596187	1.20E-09	9.63E-09
PARG22317	-2.0689143	2.725091835	1.21E-09	9.66E-09
PARG18408	-2.30808355	0.905189138	1.21E-09	9.66E-09
PARG25753	-3.352426781	0.731276933	1.22E-09	9.75E-09
PARG23120	-3.241323382	3.95115338	1.22E-09	9.75E-09
PARG06742	-2.193316693	1.538154659	1.24E-09	9.90E-09
PARG21214	-1.754550918	4.356236915	1.25E-09	9.97E-09
PARG07039	-3.650476503	1.643757525	1.25E-09	1.00E-08
PARG13903	-3.647915621	-0.319731678	1.25E-09	1.00E-08
PARG21800	-2.116764525	3.397095576	1.26E-09	1.00E-08
PARG26973	-2.682244526	0.689939608	1.27E-09	1.01E-08
PARG18624	-1.666985832	5.169394741	1.28E-09	1.02E-08
PARG12956	1.696601147	4.903055353	1.28E-09	1.02E-08
PARG27276	-1.950185201	3.077843332	1.28E-09	1.02E-08
PARG15912	-1.646442458	8.774559407	1.28E-09	1.02E-08
PARG23908	-3.076879222	0.854472577	1.31E-09	1.04E-08
PARG15817	-1.722763486	4.142324915	1.33E-09	1.06E-08
PARG07994	-1.678710211	5.266830176	1.36E-09	1.08E-08
PARG15799	-1.77170387	3.336255862	1.37E-09	1.09E-08

PARG24166	-2.104784502	2.501719112	1.39E-09	1.11E-08
PARG06752	1.643207865	8.251752994	1.40E-09	1.11E-08
PARG08050	1.749549039	3.908318432	1.42E-09	1.13E-08
PARG12441	2.129944482	2.006995125	1.43E-09	1.14E-08
PARG12470	-2.406386664	1.325732095	1.49E-09	1.18E-08
PARG10318	1.695775399	4.561499269	1.54E-09	1.22E-08
PARG17648	-3.214093042	-0.267090018	1.55E-09	1.23E-08
PARG20891	-3.196805252	1.653810249	1.55E-09	1.23E-08
PARG01358	1.675828733	5.771518924	1.56E-09	1.23E-08
PARG21382	-2.311150298	1.285655336	1.57E-09	1.24E-08
PARG19761	-2.403322575	2.22660345	1.58E-09	1.25E-08
PARG14605	-1.669330242	5.697916538	1.58E-09	1.25E-08
PARG20596	-2.183762901	2.252306257	1.60E-09	1.26E-08
PARG24627	-1.847557361	3.02432379	1.61E-09	1.27E-08
PARG07679	2.558591845	1.254038233	1.62E-09	1.27E-08
PARG20894	1.637918295	7.911782076	1.64E-09	1.29E-08
PARG21703	4.302927579	-0.686814319	1.65E-09	1.30E-08
PARG28104	-3.897296911	0.451972003	1.68E-09	1.32E-08
PARG21106	-3.890691476	0.976690604	1.68E-09	1.32E-08
PARG24181	-2.420581976	1.470871307	1.69E-09	1.33E-08
PARG18356	-1.946597596	3.280743161	1.70E-09	1.33E-08
PARG03920	1.643964281	6.423964689	1.70E-09	1.34E-08
PARG20247	-1.690483346	4.383740893	1.71E-09	1.35E-08
PARG23062	1.639208244	7.073034344	1.71E-09	1.35E-08
PARG25518	1.650717896	6.282417036	1.72E-09	1.35E-08
PARG04493	1.688430054	3.931140719	1.72E-09	1.35E-08
PARG13822	-6.577285883	0.252079339	1.73E-09	1.36E-08
PARG23955	-6.568217197	0.46316233	1.73E-09	1.36E-08
PARG14187	4.878061919	0.239558377	1.73E-09	1.36E-08
PARG08846	-3.509590751	0.846449587	1.75E-09	1.37E-08
PARG20505	-2.050937406	1.926551611	1.75E-09	1.37E-08
PARG01820	-1.830597785	2.195009237	1.76E-09	1.38E-08
PARG21575	-3.636801888	1.020544905	1.79E-09	1.40E-08
PARG25508	1.681897586	4.533209065	1.79E-09	1.40E-08
PARG12859	-1.65719605	6.346687878	1.79E-09	1.40E-08
PARG24281	-1.630737516	8.40287514	1.83E-09	1.43E-08
PARG03520	1.634020562	7.333473608	1.83E-09	1.43E-08
PARG09368	1.63207492	8.170765759	1.84E-09	1.44E-08
PARG08905	1.629821154	8.142971262	1.87E-09	1.46E-08
PARG10440	-1.910744945	2.665909625	1.90E-09	1.48E-08
PARG13775	-2.420631077	1.782880953	1.92E-09	1.50E-08
PARG21997	-1.792203014	2.75871944	1.96E-09	1.52E-08
PARG00233	1.825575007	3.198968281	1.96E-09	1.53E-08
PARG21137	1.62873487	8.416204015	1.97E-09	1.54E-08
PARG27814	1.682397234	4.957457569	2.00E-09	1.55E-08
PARG18329	1.636971974	7.095085274	2.02E-09	1.57E-08

PARG17983	1.672175816	4.27293637	2.03E-09	1.58E-08
PARG26415	1.692730618	4.512835605	2.04E-09	1.58E-08
PARG00106	1.699409332	4.763651594	2.07E-09	1.60E-08
PARG12781	-1.669216894	4.780298794	2.11E-09	1.64E-08
PARG12739	1.731299926	4.45715904	2.14E-09	1.66E-08
PARG22730	1.86273975	2.454239797	2.14E-09	1.66E-08
PARG21265	1.628670742	7.021275341	2.15E-09	1.66E-08
PARG04672	-2.170492526	0.892262218	2.15E-09	1.67E-08
PARG18523	-1.677042135	5.037128352	2.21E-09	1.71E-08
PARG18804	1.729027784	3.536134379	2.23E-09	1.72E-08
PARG25223	-2.003093957	1.752258224	2.24E-09	1.73E-08
PARG29728	2.951231767	0.933212926	2.28E-09	1.76E-08
PARG29799	1.694731507	3.881619888	2.28E-09	1.76E-08
PARG13270	-1.664235599	4.772484993	2.33E-09	1.80E-08
PARG02138	2.342830407	1.036546234	2.38E-09	1.83E-08
PARG26864	-1.772122636	3.536710154	2.39E-09	1.84E-08
PARG26751	1.662292478	4.917541979	2.41E-09	1.86E-08
PARG27513	1.676437342	4.296591522	2.52E-09	1.94E-08
PARG16247	1.621976837	7.104777085	2.54E-09	1.96E-08
PARG16514	1.619822659	7.91626374	2.54E-09	1.96E-08
PARG20021	1.786489016	3.224265855	2.63E-09	2.02E-08
PARG20128	-1.78270173	3.948799177	2.72E-09	2.09E-08
PARG19420	1.608453198	11.38168576	2.78E-09	2.14E-08
PARG28734	1.649213926	5.257776861	2.79E-09	2.14E-08
PARG01041	-2.108688185	1.780759932	2.82E-09	2.16E-08
PARG26208	-6.53437863	-0.217667426	2.83E-09	2.17E-08
PARG24787	-1.665333617	4.837892446	2.83E-09	2.17E-08
PARG06570	1.794324271	2.549479027	2.84E-09	2.17E-08
PARG27581	1.611470061	8.25738808	2.84E-09	2.17E-08
PARG17958	2.87536042	1.734581781	2.85E-09	2.18E-08
PARG08584	1.617021622	6.42181399	2.87E-09	2.19E-08
PARG04464	1.616494941	6.747797082	2.88E-09	2.20E-08
PARG07796	1.806468848	2.457281602	2.92E-09	2.23E-08
PARG19449	-1.626907995	5.660574018	2.93E-09	2.24E-08
PARG16766	1.672701843	4.373134793	2.93E-09	2.24E-08
PARG08089	1.60765813	8.283644028	3.00E-09	2.29E-08
PARG14268	-1.629829906	6.452431416	3.01E-09	2.30E-08
PARG23842	-1.619417011	6.140199983	3.04E-09	2.32E-08
PARG14145	1.674387951	4.633102251	3.12E-09	2.38E-08
PARG18615	1.60713804	8.238809664	3.19E-09	2.43E-08
PARG06901	-1.618859292	6.160269416	3.21E-09	2.44E-08
PARG15611	-4.243566983	-0.171726773	3.21E-09	2.44E-08
PARG15637	-4.243566983	-0.171726773	3.21E-09	2.44E-08
PARG10399	-1.715138541	4.085180501	3.24E-09	2.46E-08
PARG20253	-1.614170303	5.927147956	3.26E-09	2.48E-08
PARG08598	-1.604901235	7.876513363	3.39E-09	2.57E-08

PARG01881	1.602455578	8.150363915	3.42E-09	2.60E-08
PARG29567	1.619388434	5.488719777	3.52E-09	2.67E-08
PARG00990	-3.847650079	1.402355633	3.56E-09	2.70E-08
PARG27682	-3.845265822	0.98947741	3.56E-09	2.70E-08
PARG22991	3.503732047	-0.211794187	3.56E-09	2.70E-08
PARG24170	1.641460568	5.208993633	3.56E-09	2.70E-08
PARG28099	1.82972937	2.934485815	3.58E-09	2.71E-08
PARG07726	-1.625681632	4.237677151	3.59E-09	2.71E-08
PARG00942	1.598707713	8.73509804	3.62E-09	2.73E-08
PARG12398	-1.699602515	4.035370937	3.62E-09	2.74E-08
PARG08887	-3.685601939	-0.708044898	3.65E-09	2.76E-08
PARG24971	1.601075333	7.638913578	3.69E-09	2.79E-08
PARG13613	-1.792917624	3.319320119	3.71E-09	2.80E-08
PARG06368	1.688511674	4.458192449	3.73E-09	2.82E-08
PARG26789	-1.755386943	3.630189708	3.78E-09	2.85E-08
PARG06987	1.717122973	4.547307335	3.79E-09	2.86E-08
PARG27197	-1.748824684	2.969121562	3.80E-09	2.86E-08
PARG21073	-1.936100954	4.904593851	3.82E-09	2.88E-08
PARG24528	2.919536975	-0.018209345	3.86E-09	2.91E-08
PARG19737	2.53940627	1.606960425	3.90E-09	2.93E-08
PARG21024	1.638673653	5.05018862	3.91E-09	2.94E-08
PARG00329	-4.896031058	-0.686289136	3.92E-09	2.94E-08
PARG13945	-4.105173954	1.282256297	3.92E-09	2.94E-08
PARG14527	-1.595979263	8.269519114	3.94E-09	2.96E-08
PARG09762	-1.665290234	4.740036724	3.97E-09	2.98E-08
PARG16615	-1.645919225	4.19283559	4.09E-09	3.07E-08
PARG01894	1.742720271	3.7151677	4.12E-09	3.09E-08
PARG03831	1.629833222	5.475141842	4.17E-09	3.13E-08
PARG16108	1.620627036	5.020487222	4.21E-09	3.15E-08
PARG03652	-1.68829934	4.079995291	4.27E-09	3.19E-08
PARG07801	-1.822546708	2.529617066	4.30E-09	3.22E-08
PARG19841	-2.212553975	1.193072891	4.31E-09	3.22E-08
PARG00508	1.587810915	10.49649729	4.38E-09	3.27E-08
PARG15745	-1.701784053	3.542835236	4.41E-09	3.29E-08
PARG20452	1.597898451	7.279238205	4.44E-09	3.31E-08
PARG27608	1.61841681	5.402707929	4.44E-09	3.32E-08
PARG05319	1.590305931	8.227314274	4.45E-09	3.32E-08
PARG23956	1.592252815	7.674111312	4.48E-09	3.34E-08
PARG01956	-1.591900809	8.033199824	4.49E-09	3.35E-08
PARG00092	-1.659833255	4.217831626	4.50E-09	3.35E-08
PARG02195	1.590556134	7.298788522	4.62E-09	3.44E-08
PARG01764	-6.492182188	-0.102412059	4.64E-09	3.45E-08
PARG16403	1.603595878	5.930774688	4.65E-09	3.46E-08
PARG10614	-2.301035	1.147058165	4.68E-09	3.48E-08
PARG05642	-1.71200818	3.62776755	4.69E-09	3.48E-08
PARG23719	-1.933130125	2.374725772	4.79E-09	3.56E-08

PARG07760	-4.218417669	0.344002406	4.79E-09	3.56E-08
PARG22735	-1.74967456	2.412694925	4.87E-09	3.61E-08
PARG06267	1.612895516	5.177750495	4.92E-09	3.65E-08
PARG04024	1.832437305	2.209956166	5.12E-09	3.80E-08
PARG07117	-1.585944578	7.34488193	5.12E-09	3.80E-08
PARG16565	2.195908908	1.396470989	5.12E-09	3.80E-08
PARG07575	1.612020975	5.35747164	5.19E-09	3.85E-08
PARG02135	-3.55758796	0.013747818	5.24E-09	3.88E-08
PARG10523	-3.555685897	0.006736221	5.24E-09	3.88E-08
PARG20337	-3.131136704	0.358615825	5.25E-09	3.88E-08
PARG21276	1.648428113	4.647058922	5.35E-09	3.95E-08
PARG04147	-1.978038552	1.769629223	5.79E-09	4.28E-08
PARG28887	-1.68547375	3.641029927	5.88E-09	4.34E-08
PARG24069	-3.072016971	0.283277542	5.90E-09	4.35E-08
PARG10979	2.895080541	0.876387755	5.90E-09	4.35E-08
PARG27737	-1.652725584	4.733918841	5.93E-09	4.37E-08
PARG24530	-2.112075708	0.85789273	6.11E-09	4.50E-08
PARG07380	-1.57900329	6.840286175	6.29E-09	4.63E-08
PARG00821	-5.371517586	0.138699444	6.43E-09	4.73E-08
PARG01515	-1.910463997	2.540373884	6.46E-09	4.76E-08
PARG00646	-1.589610669	6.155204005	6.53E-09	4.81E-08
PARG06922	-1.619184157	5.473319704	6.64E-09	4.88E-08
PARG15345	1.574548338	7.419642489	6.64E-09	4.88E-08
PARG11496	1.672878501	3.868505605	6.74E-09	4.95E-08
PARG09757	1.569715579	8.421573715	6.80E-09	4.99E-08
PARG22774	-2.225408792	1.622553487	6.93E-09	5.09E-08
PARG03742	-1.570858649	7.502914565	6.97E-09	5.11E-08
PARG00863	-1.604814807	5.320614687	6.99E-09	5.13E-08
PARG07098	-3.330633492	-0.740144889	7.13E-09	5.22E-08
PARG06406	2.831164432	0.221065484	7.13E-09	5.23E-08
PARG14511	-4.200865108	1.060968781	7.16E-09	5.24E-08
PARG30009	-1.906272123	1.559730374	7.21E-09	5.27E-08
PARG02774	-1.702494858	3.768719353	7.24E-09	5.30E-08
PARG06906	1.614144672	5.429511437	7.34E-09	5.37E-08
PARG11966	1.579755074	5.962372751	7.35E-09	5.37E-08
PARG10619	1.746311804	3.692075207	7.37E-09	5.39E-08
PARG20652	-2.039746085	2.44646061	7.44E-09	5.43E-08
PARG06564	-1.627281313	4.91448514	7.53E-09	5.50E-08
PARG13646	-2.493417512	1.500446923	7.57E-09	5.52E-08
PARG18245	-1.764180719	2.535977788	7.59E-09	5.53E-08
PARG23904	-2.211491617	1.164625014	7.66E-09	5.58E-08
PARG12938	-1.636205541	4.055763846	7.77E-09	5.66E-08
PARG06184	1.932653421	1.621877443	7.83E-09	5.70E-08
PARG18943	-1.908390259	2.228222911	7.91E-09	5.76E-08
PARG19264	1.564546271	7.649158504	7.97E-09	5.79E-08
PARG14456	-1.8936691	3.422429578	7.98E-09	5.80E-08

PARG21000	1.708522423	4.251676554	8.05E-09	5.85E-08
PARG17088	1.587344252	5.36498321	8.11E-09	5.89E-08
PARG07505	-2.095696072	1.656131721	8.25E-09	5.99E-08
PARG27740	1.607301855	4.607396376	8.37E-09	6.07E-08
PARG07057	-2.829763255	0.52962961	8.37E-09	6.07E-08
PARG24762	-2.826783655	1.004416968	8.37E-09	6.07E-08
PARG19535	-1.569218659	6.142905732	8.40E-09	6.09E-08
PARG07939	-1.572082698	7.117016374	8.49E-09	6.15E-08
PARG12624	-1.964168807	2.495874531	8.77E-09	6.35E-08
PARG27952	1.556657108	8.402585617	9.00E-09	6.51E-08
PARG06899	-1.760858401	3.501454111	9.03E-09	6.53E-08
PARG10225	-2.153424743	0.427623729	9.15E-09	6.62E-08
PARG17530	-4.854810553	-1.015294405	9.20E-09	6.65E-08
PARG24519	-4.83954829	-0.070419685	9.20E-09	6.65E-08
PARG08904	1.586753235	5.096375533	9.21E-09	6.65E-08
PARG00340	-1.653115711	4.233398017	9.21E-09	6.65E-08
PARG06532	-2.257120499	1.74183158	9.34E-09	6.74E-08
PARG19224	1.575638228	5.30002429	9.48E-09	6.84E-08
PARG09147	-2.875583727	0.12928776	9.58E-09	6.91E-08
PARG12871	-1.677084752	3.191084982	9.64E-09	6.94E-08
PARG07999	-1.706591972	3.43937714	9.69E-09	6.98E-08
PARG28581	-2.764234803	0.749171628	9.93E-09	7.14E-08
PARG20983	-2.659331643	1.434959109	9.93E-09	7.14E-08
PARG14110	-2.655782454	0.291801218	9.93E-09	7.14E-08
PARG27289	-2.233302145	1.118890241	9.95E-09	7.16E-08
PARG04529	-1.609627721	3.435494494	9.97E-09	7.16E-08
PARG15232	-1.562615386	6.294050424	1.01E-08	7.24E-08
PARG13149	-3.31498966	0.021743913	1.01E-08	7.24E-08
PARG20796	-1.564013132	6.251207897	1.01E-08	7.26E-08
PARG06944	-4.663789391	1.019598537	1.02E-08	7.30E-08
PARG02986	1.804798405	2.653294075	1.02E-08	7.31E-08
PARG13490	-1.671128062	3.454849293	1.02E-08	7.33E-08
PARG08415	1.55578756	6.8370661	1.03E-08	7.37E-08
PARG13517	-1.550291289	8.130579969	1.04E-08	7.43E-08
PARG22748	1.597532848	4.414224824	1.04E-08	7.46E-08
PARG21579	-1.669242653	3.65352095	1.05E-08	7.51E-08
PARG26868	-4.167728263	0.002273992	1.07E-08	7.65E-08
PARG06219	-4.155083106	-0.141803231	1.07E-08	7.65E-08
PARG11400	-4.152322891	-0.023024972	1.07E-08	7.65E-08
PARG07283	-3.499419744	0.382123513	1.08E-08	7.73E-08
PARG06199	-3.484989406	-0.507453174	1.08E-08	7.73E-08
PARG26289	-1.546157598	9.020360342	1.09E-08	7.76E-08
PARG14726	1.877315466	1.731863301	1.10E-08	7.86E-08
PARG03300	-2.08660143	1.58208764	1.11E-08	7.90E-08
PARG20130	2.051659494	1.512696246	1.11E-08	7.93E-08
PARG19107	-3.771050124	0.537578353	1.11E-08	7.93E-08

PARG06056	-3.770256986	0.379549393	1.11E-08	7.93E-08
PARG09034	1.899448691	2.073700324	1.11E-08	7.93E-08
PARG07386	-1.581204267	5.158018323	1.12E-08	7.99E-08
PARG13027	-1.558109374	6.405171667	1.17E-08	8.35E-08
PARG14157	-2.373937511	3.645522869	1.21E-08	8.62E-08
PARG29179	-1.630674672	4.356565219	1.23E-08	8.72E-08
PARG15059	-1.557851315	5.857618135	1.23E-08	8.72E-08
PARG08694	-1.739509056	2.991618082	1.25E-08	8.87E-08
PARG00506	-6.417610736	-0.000740003	1.26E-08	8.93E-08
PARG30210	1.578272813	4.829698819	1.27E-08	8.99E-08
PARG14331	1.643245404	4.031804599	1.32E-08	9.36E-08
PARG20343	1.553376504	6.260654999	1.34E-08	9.51E-08
PARG19285	-1.683866129	3.797989534	1.35E-08	9.58E-08
PARG09557	-1.937401727	1.60301948	1.38E-08	9.79E-08
PARG13952	-2.678977637	1.271231694	1.39E-08	9.83E-08
PARG06721	-1.613991977	4.496443301	1.39E-08	9.85E-08
PARG14816	2.339661042	1.739132385	1.41E-08	9.95E-08
PARG14334	-1.740603823	2.683902106	1.46E-08	1.03E-07
PARG18097	-1.772593687	2.528736971	1.47E-08	1.04E-07
PARG27455	-1.630549176	3.526591479	1.47E-08	1.04E-07
PARG15679	-1.5561027	6.534068468	1.48E-08	1.04E-07
PARG13536	1.945223074	2.180448219	1.48E-08	1.04E-07
PARG17945	1.538862518	6.982679703	1.48E-08	1.05E-07
PARG15883	1.578229852	4.737902653	1.50E-08	1.06E-07
PARG22002	-1.592611722	4.320471372	1.50E-08	1.06E-07
PARG27868	-1.591730484	4.657248953	1.55E-08	1.09E-07
PARG16558	1.564245553	4.985138101	1.56E-08	1.10E-07
PARG29162	-3.485479319	0.462765149	1.56E-08	1.10E-07
PARG09393	1.532637346	6.86195038	1.59E-08	1.11E-07
PARG16371	1.606430955	4.950144979	1.59E-08	1.12E-07
PARG29241	-1.607667588	3.30026678	1.62E-08	1.14E-07
PARG00563	1.584745182	4.435595602	1.62E-08	1.14E-07
PARG05558	-1.528389413	7.805963189	1.63E-08	1.14E-07
PARG23275	-1.711424469	3.512303021	1.63E-08	1.14E-07
PARG12783	-3.755770048	0.836344353	1.64E-08	1.15E-07
PARG13430	-3.748934984	0.326530631	1.64E-08	1.15E-07
PARG11677	-3.746327763	0.8808458	1.64E-08	1.15E-07
PARG27982	-1.678320056	3.510217479	1.64E-08	1.15E-07
PARG23915	-2.256880134	1.047988016	1.70E-08	1.19E-07
PARG02039	1.559350497	5.02092291	1.71E-08	1.20E-07
PARG26561	-2.141554541	1.951687293	1.73E-08	1.21E-07
PARG30380	-1.840981277	2.05440546	1.76E-08	1.23E-07
PARG22400	-2.133154706	4.002692549	1.76E-08	1.23E-07
PARG29161	1.543647333	5.447485702	1.82E-08	1.27E-07
PARG28752	1.557032922	5.242162143	1.85E-08	1.29E-07
PARG16049	1.641428608	3.438220242	1.85E-08	1.29E-07

PARG29429	-1.580413393	4.903066322	1.87E-08	1.30E-07
PARG11823	-1.61084096	3.547726012	1.87E-08	1.31E-07
PARG03694	1.518767518	9.237884965	1.88E-08	1.31E-07
PARG29928	2.645213086	-0.001516174	1.88E-08	1.31E-07
PARG15888	1.550130616	4.972733449	1.92E-08	1.34E-07
PARG27270	-2.018030042	2.633617999	1.95E-08	1.35E-07
PARG10608	1.873302039	3.063434659	1.98E-08	1.38E-07
PARG17064	-1.66085487	3.002322492	2.01E-08	1.39E-07
PARG30166	-1.71598405	2.69922125	2.02E-08	1.40E-07
PARG17654	1.559931058	4.753274597	2.03E-08	1.41E-07
PARG16271	2.349012313	0.023909848	2.07E-08	1.44E-07
PARG13513	-2.168018883	1.069309354	2.08E-08	1.44E-07
PARG19866	-1.755005895	2.54603713	2.08E-08	1.44E-07
PARG27981	1.557837206	4.505147555	2.09E-08	1.45E-07
PARG23403	-6.377141664	-0.246083469	2.09E-08	1.45E-07
PARG03379	-6.375279049	-0.925565746	2.09E-08	1.45E-07
PARG07369	-6.373405334	-0.64478201	2.09E-08	1.45E-07
PARG18440	-6.369338939	0.025963925	2.09E-08	1.45E-07
PARG13239	-6.363655771	-0.250963569	2.09E-08	1.45E-07
PARG19108	-1.915877878	1.717311331	2.09E-08	1.45E-07
PARG05121	1.518757513	7.35353612	2.10E-08	1.45E-07
PARG19438	-1.547123015	5.298199383	2.13E-08	1.47E-07
PARG26884	-1.516910361	7.393123455	2.21E-08	1.52E-07
PARG27306	-3.453447824	2.925176102	2.26E-08	1.56E-07
PARG18918	1.525322781	5.896000711	2.28E-08	1.57E-07
PARG07109	1.596541402	3.837956993	2.32E-08	1.60E-07
PARG08667	-1.511131249	8.459619942	2.33E-08	1.61E-07
PARG16397	1.525611102	5.921014125	2.34E-08	1.61E-07
PARG27473	1.54190587	5.969097508	2.38E-08	1.64E-07
PARG15800	1.688447088	2.498582818	2.39E-08	1.65E-07
PARG18712	1.530040069	5.557715052	2.40E-08	1.65E-07
PARG11916	1.564470209	4.710367807	2.41E-08	1.65E-07
PARG27171	-2.261796451	0.411373958	2.41E-08	1.66E-07
PARG12985	-1.76082628	2.644319503	2.41E-08	1.66E-07
PARG12117	-4.103274995	0.439998692	2.42E-08	1.66E-07
PARG07414	-1.526506525	5.357629041	2.42E-08	1.66E-07
PARG12824	-1.868731529	2.270103198	2.44E-08	1.67E-07
PARG03042	1.624802754	3.654088786	2.49E-08	1.71E-07
PARG20943	-3.099027633	0.749299551	2.50E-08	1.71E-07
PARG06058	-4.576951294	0.351579197	2.57E-08	1.76E-07
PARG14852	-1.541112649	5.330232829	2.58E-08	1.77E-07
PARG20886	1.715081046	2.880791006	2.59E-08	1.77E-07
PARG16525	1.822487064	3.290217953	2.60E-08	1.78E-07
PARG10864	1.570642287	4.857672363	2.63E-08	1.80E-07
PARG19280	1.5338809	5.247982285	2.65E-08	1.81E-07
PARG01988	1.699133353	2.964532743	2.67E-08	1.82E-07

PARG07108	1.508738171	6.556821929	2.68E-08	1.83E-07
PARG14090	1.519709081	5.635985737	2.71E-08	1.85E-07
PARG15517	1.878351711	1.252908741	2.75E-08	1.87E-07
PARG18888	1.727693812	2.315779657	2.77E-08	1.89E-07
PARG03655	1.711258454	2.842824179	2.78E-08	1.90E-07
PARG10386	-1.634583088	3.937171612	2.79E-08	1.90E-07
PARG11354	1.986238732	1.120361117	2.84E-08	1.93E-07
PARG12682	-2.585577717	0.491906799	2.85E-08	1.94E-07
PARG23724	1.500404282	7.969787713	2.88E-08	1.96E-07
PARG06857	-1.61730007	4.416879172	2.89E-08	1.97E-07
PARG27663	-1.518719527	5.747165319	2.89E-08	1.97E-07
PARG00972	-1.509411187	6.733578535	2.93E-08	1.99E-07
PARG14248	-1.555987858	5.768277729	2.97E-08	2.02E-07
PARG00851	-1.598951514	4.459604138	2.99E-08	2.03E-07
PARG16319	-2.525974548	1.283053861	3.00E-08	2.04E-07
PARG07990	-1.563465833	4.193266493	3.19E-08	2.16E-07
PARG08310	1.501290126	6.813215193	3.26E-08	2.21E-07
PARG07781	-3.411153822	0.648648117	3.26E-08	2.21E-07
PARG01959	-3.405891226	0.990733872	3.26E-08	2.21E-07
PARG11059	3.203131533	-0.153239025	3.26E-08	2.21E-07
PARG28060	1.52187229	5.847130876	3.27E-08	2.21E-07
PARG21185	2.587073076	1.402855421	3.31E-08	2.24E-07
PARG29559	-1.593079701	3.635555871	3.31E-08	2.24E-07
PARG00544	1.509674654	6.241543406	3.36E-08	2.27E-07
PARG24855	1.575658332	4.30468522	3.36E-08	2.27E-07
PARG12285	-4.719360978	1.968194985	3.37E-08	2.28E-07
PARG15141	-1.508804589	6.464604328	3.42E-08	2.31E-07
PARG07261	-1.505011018	5.625981594	3.46E-08	2.34E-07
PARG03495	-2.133647169	2.131119856	3.52E-08	2.38E-07
PARG08871	1.488088884	8.847561308	3.55E-08	2.39E-07
PARG02316	-2.296106112	1.29757881	3.56E-08	2.40E-07
PARG22796	1.553071422	5.150955164	3.57E-08	2.40E-07
PARG28302	-1.494805266	6.902099077	3.57E-08	2.41E-07
PARG19754	-1.644634593	3.439913614	3.57E-08	2.41E-07
PARG00581	1.613172164	3.342544975	3.58E-08	2.41E-07
PARG06166	-2.150816426	1.826869088	3.60E-08	2.42E-07
PARG25189	-1.5403499	4.861454588	3.63E-08	2.44E-07
PARG07177	1.499476274	6.333287387	3.64E-08	2.45E-07
PARG09014	-4.07772572	0.874487212	3.65E-08	2.45E-07
PARG06777	1.489088002	8.19352399	3.65E-08	2.45E-07
PARG25980	1.490026473	7.513551483	3.66E-08	2.46E-07
PARG02005	1.595051698	4.028266402	3.74E-08	2.51E-07
PARG26649	-1.9126143	1.614256342	3.77E-08	2.53E-07
PARG27638	1.544921069	4.476542102	3.78E-08	2.53E-07
PARG07598	1.489504124	7.829101663	3.81E-08	2.56E-07
PARG28435	-1.497666076	6.535106033	3.82E-08	2.56E-07

PARG13422	-1.487418109	7.540323842	3.85E-08	2.58E-07
PARG24840	1.914902227	1.896951761	3.89E-08	2.61E-07
PARG05752	-1.720814686	3.272527147	3.93E-08	2.63E-07
PARG11160	2.024738265	1.478504187	3.95E-08	2.64E-07
PARG06627	-1.502458747	5.721501778	3.97E-08	2.66E-07
PARG23599	1.511148528	5.204677498	4.07E-08	2.72E-07
PARG00992	1.870969798	1.841160892	4.09E-08	2.73E-07
PARG18069	-4.549062663	0.36954499	4.12E-08	2.74E-07
PARG20194	-4.547561494	-0.474229493	4.12E-08	2.74E-07
PARG28079	-4.543068974	-0.189159531	4.12E-08	2.74E-07
PARG01733	4.046285453	0.67833696	4.12E-08	2.74E-07
PARG00530	-1.710240205	2.661578076	4.12E-08	2.75E-07
PARG07799	-2.492740219	1.318598829	4.14E-08	2.76E-07
PARG26979	1.502838457	5.785013617	4.15E-08	2.76E-07
PARG19301	-1.489917554	6.590698186	4.15E-08	2.77E-07
PARG12128	-1.53782212	5.236736497	4.20E-08	2.79E-07
PARG00252	1.509547879	5.157028612	4.22E-08	2.81E-07
PARG06413	-1.660213534	3.101636011	4.23E-08	2.81E-07
PARG16651	1.547305	3.613403328	4.24E-08	2.82E-07
PARG17110	-1.476342858	11.1684675	4.33E-08	2.88E-07
PARG20077	-1.552053841	4.002279128	4.34E-08	2.88E-07
PARG03898	-2.268855197	0.593547609	4.43E-08	2.94E-07
PARG17720	-1.478152359	7.639123666	4.57E-08	3.03E-07
PARG12964	1.517552063	5.217062346	4.58E-08	3.03E-07
PARG26144	-1.776050731	2.394037476	4.59E-08	3.04E-07
PARG06541	1.525670005	4.371340128	5.02E-08	3.33E-07
PARG12743	-1.575687503	3.382557211	5.06E-08	3.35E-07
PARG12183	-1.478726859	6.500734247	5.06E-08	3.35E-07
PARG09725	1.47780857	7.035414829	5.09E-08	3.37E-07
PARG09356	-1.847230835	3.501397896	5.17E-08	3.42E-07
PARG02897	-1.74874167	2.133695838	5.19E-08	3.43E-07
PARG20523	-1.533559181	4.190782869	5.21E-08	3.44E-07
PARG19314	-1.651764586	2.752440184	5.22E-08	3.45E-07
PARG02807	-3.649251247	0.600909436	5.25E-08	3.47E-07
PARG06119	-3.642664443	0.345229795	5.25E-08	3.47E-07
PARG16202	1.745263717	3.053312384	5.28E-08	3.48E-07
PARG19769	-1.529382371	4.196753296	5.33E-08	3.52E-07
PARG00602	1.477403925	6.453362288	5.37E-08	3.54E-07
PARG04126	1.565871821	3.715298348	5.38E-08	3.54E-07
PARG05975	-2.204360288	1.230458858	5.42E-08	3.57E-07
PARG13939	-1.60456075	4.242447449	5.44E-08	3.58E-07
PARG17158	2.253031966	0.689019818	5.47E-08	3.60E-07
PARG15774	-4.027071852	0.199361568	5.51E-08	3.62E-07
PARG11041	-1.506175978	5.055155355	5.53E-08	3.63E-07
PARG13875	1.48069676	5.726707311	5.59E-08	3.67E-07
PARG15662	1.518496449	4.577764187	5.65E-08	3.71E-07

PARG24385	-1.551745373	4.559205463	5.79E-08	3.80E-07
PARG02107	1.469097137	7.388866468	5.79E-08	3.80E-07
PARG04360	-3.178544569	0.846863109	5.87E-08	3.85E-07
PARG23261	1.463058627	8.467582551	5.90E-08	3.87E-07
PARG04643	-1.547332377	4.17652086	5.95E-08	3.90E-07
PARG01438	-2.601361725	-0.559286312	6.12E-08	4.01E-07
PARG03512	2.408923087	0.737704538	6.12E-08	4.01E-07
PARG00450	1.478594685	5.712908881	6.12E-08	4.01E-07
PARG23834	-1.558235551	3.955206372	6.17E-08	4.04E-07
PARG18370	1.946934848	3.427901061	6.18E-08	4.04E-07
PARG23385	-2.784516431	0.891293818	6.32E-08	4.13E-07
PARG08991	2.461112565	-0.021200253	6.35E-08	4.15E-07
PARG16670	-1.681997455	3.149780491	6.39E-08	4.18E-07
PARG03411	1.974182947	2.19312129	6.44E-08	4.21E-07
PARG10490	1.644918129	3.199536552	6.52E-08	4.26E-07
PARG21176	-1.636795684	2.221887304	6.55E-08	4.27E-07
PARG24262	-2.185688234	1.507434633	6.65E-08	4.34E-07
PARG03424	-1.498650571	4.357680846	6.72E-08	4.38E-07
PARG18378	1.47710585	5.816930187	6.73E-08	4.39E-07
PARG12116	-2.242385734	2.148006807	6.86E-08	4.47E-07
PARG25720	-1.537725178	3.565216311	6.93E-08	4.51E-07
PARG20995	1.462619602	6.705834472	7.02E-08	4.57E-07
PARG23025	1.46408201	5.849478903	7.09E-08	4.62E-07
PARG03492	-2.091952512	2.08390671	7.16E-08	4.65E-07
PARG13037	1.515772565	4.330001669	7.16E-08	4.66E-07
PARG22864	-1.753302434	2.159765228	7.18E-08	4.67E-07
PARG06676	1.584239317	3.521229766	7.28E-08	4.73E-07
PARG25396	-2.148010445	1.508940594	7.30E-08	4.74E-07
PARG23289	1.693192194	1.733999912	7.41E-08	4.81E-07
PARG12772	-1.502402036	4.546128348	7.42E-08	4.82E-07
PARG23520	-1.472038182	5.417671218	7.46E-08	4.84E-07
PARG02333	1.564607972	4.230579456	7.48E-08	4.85E-07
PARG14210	-1.797746235	2.593370902	7.50E-08	4.86E-07
PARG02332	1.500760894	4.896478233	7.55E-08	4.89E-07
PARG04076	-1.562438285	3.653220733	7.59E-08	4.91E-07
PARG04388	1.710945477	3.074890513	7.61E-08	4.93E-07
PARG07383	-1.458306049	7.166831709	7.70E-08	4.98E-07
PARG21030	1.685832846	3.095491008	7.70E-08	4.98E-07
PARG07397	-1.468850192	5.582951069	7.77E-08	5.02E-07
PARG28105	-1.482292544	5.243271752	7.79E-08	5.04E-07
PARG13240	-2.877361126	-0.045206697	7.86E-08	5.08E-07
PARG00425	-1.652363205	3.547762591	7.87E-08	5.08E-07
PARG13921	1.766883151	1.555308936	7.87E-08	5.08E-07
PARG03307	-2.165437156	2.749647101	7.88E-08	5.09E-07
PARG09087	1.48810924	4.428207556	8.04E-08	5.18E-07
PARG18847	2.486944245	0.847943429	8.06E-08	5.20E-07

PARG11871	1.452969035	7.179953059	8.21E-08	5.29E-07
PARG16132	-1.451334046	6.582314144	8.26E-08	5.32E-07
PARG21503	-1.471286146	5.460458158	8.34E-08	5.37E-07
PARG17627	-3.150088483	1.260551747	8.38E-08	5.40E-07
PARG28109	-1.533456864	3.377534205	8.41E-08	5.41E-07
PARG25689	-2.0034095	0.986475554	8.47E-08	5.45E-07
PARG07425	1.588717395	3.314603561	8.51E-08	5.47E-07
PARG21327	-2.942022602	0.383001459	8.67E-08	5.57E-07
PARG04584	-1.446582392	7.374181856	8.73E-08	5.61E-07
PARG12547	-1.89740276	2.911894606	8.89E-08	5.71E-07
PARG25736	-1.903843204	2.949827106	8.92E-08	5.72E-07
PARG24665	1.44657659	7.572999536	8.92E-08	5.72E-07
PARG16449	1.468865603	4.704261184	9.13E-08	5.85E-07
PARG02153	1.447745654	6.384698483	9.58E-08	6.14E-07
PARG01280	-1.513935896	4.069748136	9.69E-08	6.21E-07
PARG07822	1.456355178	6.362910389	9.74E-08	6.24E-07
PARG08115	-2.976679543	0.6889273	9.75E-08	6.24E-07
PARG02282	6.307890848	-0.49881741	9.81E-08	6.27E-07
PARG10590	-6.220975529	-0.832123934	9.81E-08	6.27E-07
PARG02255	-6.199669919	0.61282364	9.81E-08	6.27E-07
PARG05044	-6.184890183	-1.186552219	9.81E-08	6.27E-07
PARG20381	-1.583520637	3.905760245	9.88E-08	6.31E-07
PARG05990	1.451250834	5.941565511	1.00E-07	6.40E-07
PARG06362	1.436890354	8.917564273	1.00E-07	6.41E-07
PARG02745	-1.733263526	2.028258743	1.01E-07	6.42E-07
PARG15814	-1.551922044	3.043896426	1.01E-07	6.44E-07
PARG06664	-2.641868462	-0.174529732	1.03E-07	6.58E-07
PARG08838	2.129381637	3.116340262	1.06E-07	6.73E-07
PARG23869	-1.484655962	4.114756374	1.06E-07	6.74E-07
PARG19423	-1.932836925	1.958491115	1.06E-07	6.76E-07
PARG23119	-5.124195753	2.131156386	1.06E-07	6.77E-07
PARG14424	1.756512536	1.170578665	1.07E-07	6.78E-07
PARG11736	-1.432466413	8.961552341	1.07E-07	6.80E-07
PARG00648	-2.274485066	3.16497185	1.07E-07	6.82E-07
PARG07983	-1.444430782	6.317456206	1.10E-07	7.02E-07
PARG03701	-1.441504859	6.326909987	1.11E-07	7.07E-07
PARG12986	-1.469360804	5.032523941	1.12E-07	7.09E-07
PARG07888	-1.553840465	3.175032902	1.12E-07	7.09E-07
PARG23944	-1.764341446	1.833101172	1.12E-07	7.10E-07
PARG21802	-1.451518091	5.471207784	1.12E-07	7.13E-07
PARG29228	-1.490709469	3.249996202	1.13E-07	7.19E-07
PARG08923	-1.448790745	6.009411113	1.14E-07	7.20E-07
PARG10667	-1.427513245	8.972258474	1.17E-07	7.41E-07
PARG12767	1.491198222	4.184789213	1.19E-07	7.54E-07
PARG01144	1.426788076	9.131930577	1.19E-07	7.55E-07
PARG23449	1.688098981	2.088938577	1.22E-07	7.70E-07

PARG05563	2.00775467	1.925765889	1.22E-07	7.73E-07
PARG21337	-1.463254113	5.121977021	1.22E-07	7.73E-07
PARG20266	1.445471836	5.529277482	1.24E-07	7.83E-07
PARG18544	-3.982707889	0.352094919	1.27E-07	8.00E-07
PARG16933	1.503992471	4.802405518	1.28E-07	8.08E-07
PARG11789	-1.964955267	1.069963668	1.28E-07	8.08E-07
PARG10749	2.100167293	0.128967815	1.29E-07	8.12E-07
PARG19621	1.425791786	7.327568814	1.29E-07	8.13E-07
PARG04702	1.437903644	5.900449816	1.30E-07	8.20E-07
PARG28337	1.466599435	4.55882016	1.30E-07	8.21E-07
PARG27245	-1.911502489	2.712029049	1.32E-07	8.30E-07
PARG03747	1.937131048	1.020894265	1.32E-07	8.33E-07
PARG22876	-1.508975969	3.789827042	1.32E-07	8.33E-07
PARG07682	-1.426796911	7.421805089	1.34E-07	8.41E-07
PARG16987	-1.900881192	1.996860611	1.34E-07	8.44E-07
PARG12826	1.766416875	1.915144965	1.35E-07	8.47E-07
PARG01789	-1.597962041	3.863284164	1.37E-07	8.59E-07
PARG28090	-1.830353849	1.579516159	1.38E-07	8.67E-07
PARG07398	-1.428946487	7.066599489	1.39E-07	8.70E-07
PARG01575	1.419706637	8.332576239	1.40E-07	8.76E-07
PARG19138	-1.652068714	3.043070324	1.40E-07	8.77E-07
PARG24507	-1.579816566	2.890283443	1.41E-07	8.83E-07
PARG26499	1.551882183	3.017075529	1.41E-07	8.83E-07
PARG13000	-1.544358396	3.19061474	1.41E-07	8.85E-07
PARG08711	-1.660576602	2.48531101	1.42E-07	8.90E-07
PARG27418	1.646065974	1.23093659	1.42E-07	8.90E-07
PARG18101	1.723005309	2.007231567	1.43E-07	8.98E-07
PARG18107	-3.316416961	0.36078137	1.45E-07	9.10E-07
PARG04021	-1.717836769	1.743665262	1.45E-07	9.10E-07
PARG21320	-1.439354411	6.531463087	1.47E-07	9.19E-07
PARG12218	1.41955266	7.793378293	1.47E-07	9.20E-07
PARG06046	1.478689583	4.649726503	1.48E-07	9.27E-07
PARG23932	-1.623876175	2.419863414	1.49E-07	9.33E-07
PARG30050	-2.816685299	0.651902204	1.52E-07	9.49E-07
PARG07714	1.481696578	4.464796566	1.52E-07	9.50E-07
PARG07104	-1.429728819	6.442376653	1.56E-07	9.72E-07
PARG08658	1.462550839	4.138553274	1.58E-07	9.84E-07
PARG11333	-1.413109946	8.525313853	1.58E-07	9.85E-07
PARG04909	-1.414335063	8.017428951	1.59E-07	9.91E-07
PARG27486	-1.441891174	5.699728603	1.60E-07	9.96E-07
PARG23491	1.424011516	6.083197417	1.60E-07	9.98E-07
PARG06569	-1.450345386	5.000859963	1.62E-07	1.01E-06
PARG28410	1.731389456	1.851483434	1.63E-07	1.01E-06
PARG13046	-1.418562512	6.72153458	1.64E-07	1.02E-06
PARG13106	-1.509366475	4.140601887	1.65E-07	1.02E-06
PARG27155	1.414626149	7.182136549	1.66E-07	1.03E-06

PARG05830	-6.152359242	0.135214405	1.66E-07	1.03E-06
PARG26898	-6.146865458	-0.130494639	1.66E-07	1.03E-06
PARG27132	-1.935269213	1.781868154	1.66E-07	1.03E-06
PARG02845	-1.496533031	3.811127653	1.66E-07	1.03E-06
PARG12863	-1.432277625	5.548129584	1.67E-07	1.04E-06
PARG27503	-3.56014875	0.331260054	1.71E-07	1.06E-06
PARG12768	3.918233698	0.341512897	1.72E-07	1.06E-06
PARG23198	-3.109788567	0.454152234	1.72E-07	1.06E-06
PARG11861	1.413143571	7.138212719	1.74E-07	1.08E-06
PARG02424	-2.302302317	0.05503407	1.76E-07	1.09E-06
PARG16428	1.525517694	3.187111841	1.77E-07	1.09E-06
PARG20270	-1.44324856	5.512859993	1.77E-07	1.09E-06
PARG18309	-1.98423183	1.40448012	1.79E-07	1.10E-06
PARG17428	-2.358391097	0.399317016	1.79E-07	1.11E-06
PARG20786	-1.413695474	5.823186706	1.80E-07	1.11E-06
PARG07161	1.418232776	6.12664434	1.81E-07	1.11E-06
PARG07163	1.413117235	6.656930993	1.81E-07	1.12E-06
PARG00243	-1.499800938	3.866405847	1.81E-07	1.12E-06
PARG19278	-1.404757026	8.711220686	1.85E-07	1.14E-06
PARG13641	-2.53883651	1.037538838	1.85E-07	1.14E-06
PARG04524	-1.88003928	2.172929086	1.87E-07	1.15E-06
PARG09162	1.457966336	4.574640639	1.89E-07	1.16E-06
PARG08459	-1.411349184	6.416337495	1.89E-07	1.16E-06
PARG01842	-1.577477353	2.791088133	1.90E-07	1.17E-06
PARG10455	1.414467155	6.190352678	1.91E-07	1.18E-06
PARG07100	-1.536844404	3.669629977	1.92E-07	1.18E-06
PARG18670	-1.864335712	1.627968649	1.93E-07	1.19E-06
PARG07805	-2.92377304	1.942126416	1.94E-07	1.19E-06
PARG24194	1.972275755	0.205547841	1.95E-07	1.20E-06
PARG25317	-1.70803081	2.636577558	1.96E-07	1.20E-06
PARG14656	-1.640395883	3.066268205	1.97E-07	1.21E-06
PARG17818	1.563434896	2.69229141	2.02E-07	1.24E-06
PARG18989	1.401528564	8.170099584	2.02E-07	1.24E-06
PARG21606	-1.647849276	2.339131768	2.03E-07	1.24E-06
PARG08327	-1.466897267	4.686603912	2.04E-07	1.25E-06
PARG20017	-1.465159826	4.309961999	2.04E-07	1.25E-06
PARG22978	-1.402233675	7.533114878	2.08E-07	1.28E-06
PARG01827	1.581148649	2.579108973	2.09E-07	1.28E-06
PARG09165	1.416816783	5.777701616	2.09E-07	1.28E-06
PARG05565	-1.564730278	3.121903717	2.13E-07	1.30E-06
PARG18668	-1.581213492	2.970291993	2.13E-07	1.30E-06
PARG08722	-1.489873885	3.629511581	2.15E-07	1.31E-06
PARG27475	1.400596704	7.05576164	2.17E-07	1.32E-06
PARG02260	-1.539152646	3.383443166	2.17E-07	1.33E-06
PARG07115	1.415982443	5.399593193	2.19E-07	1.34E-06
PARG14350	-1.394825325	10.50954852	2.21E-07	1.35E-06

PARG02667	1.56381333	3.321488538	2.22E-07	1.35E-06
PARG02307	-1.414237485	5.644395401	2.22E-07	1.35E-06
PARG16260	1.426956677	5.04068101	2.22E-07	1.35E-06
PARG24962	-2.686927904	-0.234158559	2.25E-07	1.37E-06
PARG24280	-1.446547739	4.531422428	2.31E-07	1.41E-06
PARG02234	-1.401276045	6.86514831	2.33E-07	1.42E-06
PARG26667	-2.754487819	0.83923226	2.35E-07	1.43E-06
PARG11928	-1.873829728	0.812447686	2.36E-07	1.44E-06
PARG10313	-1.892027109	1.649867606	2.39E-07	1.45E-06
PARG11692	1.401904527	6.291449783	2.42E-07	1.47E-06
PARG07102	-1.498558231	3.852301896	2.43E-07	1.48E-06
PARG00998	-1.418292187	5.610944966	2.47E-07	1.50E-06
PARG07764	-1.419545151	5.037791516	2.48E-07	1.51E-06
PARG27184	-1.478609677	3.495821932	2.55E-07	1.55E-06
PARG15922	1.454510131	4.32524339	2.55E-07	1.55E-06
PARG16312	-3.528242683	1.381715729	2.55E-07	1.55E-06
PARG27348	-1.404965699	6.233878812	2.56E-07	1.55E-06
PARG19453	1.522179754	4.196866241	2.57E-07	1.56E-06
PARG12562	1.496708739	4.004758258	2.58E-07	1.56E-06
PARG13326	-1.463889048	3.797762278	2.59E-07	1.57E-06
PARG03150	1.517908528	3.256467045	2.60E-07	1.57E-06
PARG12200	1.395651647	6.239612737	2.64E-07	1.60E-06
PARG29372	1.632089571	2.300497829	2.65E-07	1.60E-06
PARG25156	1.472929478	3.177513083	2.70E-07	1.63E-06
PARG12786	-1.472653328	3.523280237	2.74E-07	1.66E-06
PARG06433	-4.382295251	0.517009344	2.78E-07	1.68E-06
PARG02671	1.396336871	6.260241232	2.80E-07	1.69E-06
PARG26546	-6.143708153	0.856481405	2.81E-07	1.70E-06
PARG11395	-6.093738942	-0.498110254	2.81E-07	1.70E-06
PARG03435	1.51004062	3.226392049	2.83E-07	1.71E-06
PARG09015	1.395512064	6.207442286	2.83E-07	1.71E-06
PARG29153	-1.384581051	7.879842616	2.84E-07	1.71E-06
PARG08627	-1.393295	6.144256064	2.88E-07	1.73E-06
PARG07503	-1.758174194	1.916424667	2.88E-07	1.73E-06
PARG19624	-1.406749035	5.734305259	2.89E-07	1.74E-06
PARG10647	-3.90193963	0.914068685	2.94E-07	1.77E-06
PARG02323	-1.483246481	3.736401821	2.94E-07	1.77E-06
PARG05002	1.401074097	5.492482037	2.94E-07	1.77E-06
PARG03443	-1.586127545	2.392627338	2.94E-07	1.77E-06
PARG21324	1.470668052	3.705023318	2.97E-07	1.78E-06
PARG02190	-1.844519182	1.500294682	2.99E-07	1.80E-06
PARG26018	-1.381194354	7.528235168	3.02E-07	1.81E-06
PARG09441	-1.687202718	2.540753522	3.02E-07	1.81E-06
PARG14443	1.633422163	1.982462981	3.02E-07	1.81E-06
PARG06241	-1.422203349	4.351272779	3.07E-07	1.84E-06
PARG17330	3.026657179	-0.219095754	3.10E-07	1.86E-06

PARG16675	-4.541484817	0.354823782	3.11E-07	1.86E-06
PARG08257	-4.537080942	-0.212780654	3.11E-07	1.86E-06
PARG21805	1.387277829	6.310387727	3.12E-07	1.87E-06
PARG08249	1.411851721	5.791533714	3.12E-07	1.87E-06
PARG23453	-1.382346079	6.94086117	3.12E-07	1.87E-06
PARG04088	-1.44925846	3.795683989	3.14E-07	1.88E-06
PARG09758	2.336857812	0.783780563	3.14E-07	1.88E-06
PARG25986	2.109138984	1.247253769	3.17E-07	1.89E-06
PARG16161	1.403118814	5.341778783	3.19E-07	1.91E-06
PARG01953	2.272877179	-0.420029152	3.20E-07	1.91E-06
PARG01266	-1.393026798	6.00893667	3.21E-07	1.91E-06
PARG23196	2.371503503	3.293872628	3.26E-07	1.94E-06
PARG17707	1.513373602	3.434271702	3.26E-07	1.95E-06
PARG05801	-1.858956609	1.806006124	3.27E-07	1.95E-06
PARG15040	-1.542956787	3.067985221	3.29E-07	1.96E-06
PARG15796	-1.394859401	5.607475732	3.33E-07	1.98E-06
PARG27342	-1.429027225	4.249416598	3.33E-07	1.98E-06
PARG08855	-1.556134875	2.647988807	3.33E-07	1.98E-06
PARG08323	1.423334972	4.490046585	3.34E-07	1.99E-06
PARG01918	1.421345322	4.590972072	3.43E-07	2.04E-06
PARG24158	-2.162088675	1.327295515	3.44E-07	2.04E-06
PARG27846	1.493398498	3.201081174	3.46E-07	2.06E-06
PARG18517	-1.440214955	4.5871506	3.47E-07	2.06E-06
PARG19859	1.453536572	4.314908095	3.49E-07	2.07E-06
PARG18250	-1.471231136	3.167223468	3.51E-07	2.08E-06
PARG06407	-1.370475099	9.045745831	3.53E-07	2.09E-06
PARG11581	-1.378701296	6.854720696	3.54E-07	2.10E-06
PARG17725	1.391757348	5.363253092	3.57E-07	2.12E-06
PARG15951	-1.37369906	7.817272105	3.63E-07	2.15E-06
PARG13656	-1.376606998	6.671533431	3.63E-07	2.15E-06
PARG00861	-1.602218339	3.262839475	3.64E-07	2.15E-06
PARG23950	1.487839517	3.69962403	3.65E-07	2.16E-06
PARG24502	-1.374480313	6.599835615	3.66E-07	2.17E-06
PARG27535	1.372457848	7.894957621	3.68E-07	2.18E-06
PARG28265	-1.44175741	4.162928643	3.71E-07	2.19E-06
PARG12409	-1.391812903	4.447372508	3.74E-07	2.21E-06
PARG24560	-1.409823159	5.189730931	3.76E-07	2.22E-06
PARG22722	-1.409408954	4.299540684	3.84E-07	2.27E-06
PARG15352	-2.890445162	0.86761682	3.88E-07	2.29E-06
PARG06052	-2.868450018	1.174285754	3.88E-07	2.29E-06
PARG16089	-1.479990597	3.18797538	3.89E-07	2.30E-06
PARG13346	-1.412597291	4.243738854	3.92E-07	2.31E-06
PARG01618	1.417936809	3.710601316	3.92E-07	2.31E-06
PARG16174	-1.37859316	5.986080451	3.95E-07	2.33E-06
PARG11550	-1.415458406	4.178617172	3.96E-07	2.33E-06
PARG10890	1.786075409	0.978375221	3.96E-07	2.33E-06

PARG27081	-1.40717154	4.504382245	3.99E-07	2.35E-06
PARG02126	1.516063671	2.820784382	4.00E-07	2.35E-06
PARG05243	1.460022773	4.517097381	4.00E-07	2.35E-06
PARG24030	1.549519417	2.610187676	4.01E-07	2.36E-06
PARG12041	-1.5380938	2.859671872	4.04E-07	2.37E-06
PARG04485	1.369584372	7.303794107	4.11E-07	2.42E-06
PARG05886	1.874563448	2.28484399	4.13E-07	2.43E-06
PARG04153	1.620893276	1.291625276	4.14E-07	2.43E-06
PARG22110	-1.379125166	5.657079091	4.16E-07	2.44E-06
PARG21450	-1.915568878	1.434800925	4.16E-07	2.44E-06
PARG21102	-1.399122527	5.101625621	4.18E-07	2.45E-06
PARG00178	-2.412376969	-0.498816303	4.20E-07	2.46E-06
PARG26913	1.435855977	5.129740196	4.21E-07	2.47E-06
PARG24981	-2.479581482	2.283021756	4.29E-07	2.51E-06
PARG04124	-1.801110596	1.841312032	4.31E-07	2.52E-06
PARG12357	1.428882158	3.853193727	4.31E-07	2.52E-06
PARG05814	1.375767631	5.818038866	4.32E-07	2.53E-06
PARG10745	2.124603998	1.201562554	4.33E-07	2.53E-06
PARG27339	-1.373679329	6.034319959	4.34E-07	2.54E-06
PARG26573	-2.610334304	1.546250236	4.38E-07	2.56E-06
PARG03237	3.304061619	-0.673262941	4.49E-07	2.62E-06
PARG07220	1.365681593	6.540652562	4.51E-07	2.64E-06
PARG07663	-3.215599389	-0.349784692	4.54E-07	2.65E-06
PARG10652	-3.213608823	0.745725027	4.54E-07	2.65E-06
PARG27213	-1.407525206	4.276965699	4.61E-07	2.69E-06
PARG04428	-1.373642942	5.669785086	4.70E-07	2.74E-06
PARG29208	1.433873984	4.369759296	4.74E-07	2.77E-06
PARG01345	-6.048750179	0.537433168	4.80E-07	2.80E-06
PARG05596	-6.039100721	-0.683957582	4.80E-07	2.80E-06
PARG11298	1.363992667	6.313392602	4.86E-07	2.83E-06
PARG12413	-1.366470147	5.818090805	4.87E-07	2.84E-06
PARG06160	-3.700756619	0.764392671	4.89E-07	2.85E-06
PARG00556	-1.383782664	4.846798477	4.92E-07	2.86E-06
PARG25113	1.433802903	3.959402317	4.97E-07	2.89E-06
PARG17804	1.398943633	4.679283689	5.00E-07	2.91E-06
PARG16291	1.49925658	3.121066541	5.02E-07	2.92E-06
PARG05039	-1.598476095	1.550886814	5.02E-07	2.92E-06
PARG06204	1.402523645	4.74858026	5.05E-07	2.93E-06
PARG09763	1.380721166	5.147159758	5.10E-07	2.96E-06
PARG27346	1.723993849	1.547010401	5.10E-07	2.96E-06
PARG27177	-2.370100937	1.706871209	5.14E-07	2.98E-06
PARG22812	-1.385679238	4.882536726	5.15E-07	2.98E-06
PARG27365	1.352490048	7.706864286	5.24E-07	3.04E-06
PARG13335	1.376992373	5.062136128	5.27E-07	3.05E-06
PARG27257	-1.362537075	5.522553058	5.28E-07	3.06E-06
PARG25960	-1.802806221	1.872508039	5.28E-07	3.06E-06

PARG27765	-1.421791651	4.170197294	5.32E-07	3.08E-06
PARG13275	-1.359255476	6.557184697	5.34E-07	3.09E-06
PARG13624	1.957165252	0.787046009	5.36E-07	3.10E-06
PARG11008	-1.42421369	3.87764449	5.36E-07	3.10E-06
PARG20788	-2.859655377	-0.231581286	5.50E-07	3.18E-06
PARG00864	1.353202944	6.492094525	5.51E-07	3.18E-06
PARG19581	1.352933775	6.835111425	5.51E-07	3.18E-06
PARG24592	-1.389684019	4.648495604	5.65E-07	3.26E-06
PARG07544	-2.437836183	-0.193536517	5.68E-07	3.28E-06
PARG27074	-1.630801873	3.345961817	5.70E-07	3.29E-06
PARG07172	-1.351868424	7.544425616	5.71E-07	3.29E-06
PARG06891	1.404611726	5.594203155	5.77E-07	3.32E-06
PARG27804	-1.538132111	6.06172814	5.77E-07	3.33E-06
PARG06366	-1.924995416	0.832753898	5.80E-07	3.34E-06
PARG05251	1.394347125	4.448617392	5.81E-07	3.34E-06
PARG08730	-2.500183849	1.105009143	5.82E-07	3.35E-06
PARG12895	2.390637065	0.323713678	5.82E-07	3.35E-06
PARG08204	2.375845627	-0.435352769	5.82E-07	3.35E-06
PARG12480	1.364154793	4.866533758	5.85E-07	3.36E-06
PARG11161	1.464642556	3.535858979	5.94E-07	3.42E-06
PARG29950	1.355571533	5.790228738	5.95E-07	3.42E-06
PARG00404	-1.349701794	6.71678002	6.05E-07	3.47E-06
PARG28026	1.374625903	5.26824318	6.07E-07	3.49E-06
PARG19468	1.413390158	3.858306363	6.14E-07	3.52E-06
PARG15462	1.387738405	4.56220042	6.17E-07	3.54E-06
PARG24425	-1.349364436	6.616089038	6.24E-07	3.58E-06
PARG20953	-1.375495741	4.650615344	6.31E-07	3.62E-06
PARG11585	-1.464232222	3.354117261	6.31E-07	3.62E-06
PARG24202	-1.727220574	2.298165969	6.47E-07	3.71E-06
PARG22713	-1.665510434	1.593780954	6.47E-07	3.71E-06
PARG14282	-1.38813435	4.51815243	6.60E-07	3.78E-06
PARG24623	-3.221614594	-0.287237385	6.65E-07	3.81E-06
PARG29789	-1.361760372	5.054221315	6.66E-07	3.81E-06
PARG07885	-1.378960271	5.193284832	6.75E-07	3.86E-06
PARG20061	1.380955709	4.70256222	6.83E-07	3.90E-06
PARG16923	-1.454995837	2.923961305	6.83E-07	3.90E-06
PARG10755	-3.843607675	0.21543377	6.88E-07	3.93E-06
PARG23233	-3.835592379	-0.390250103	6.88E-07	3.93E-06
PARG02021	-1.921321785	1.036080873	6.96E-07	3.97E-06
PARG21785	-1.803576322	0.150776838	7.00E-07	4.00E-06
PARG06839	1.346765366	6.014189301	7.08E-07	4.04E-06
PARG16032	1.34690104	6.776457845	7.08E-07	4.04E-06
PARG10872	1.333336672	9.13356242	7.09E-07	4.04E-06
PARG16689	-1.336691796	7.408399036	7.13E-07	4.06E-06
PARG06843	-1.350794811	5.407404486	7.18E-07	4.09E-06
PARG22414	-1.385347969	4.414405088	7.23E-07	4.12E-06

PARG06346	1.666602466	1.782202864	7.25E-07	4.13E-06
PARG00764	-1.351672093	5.451178849	7.27E-07	4.13E-06
PARG09191	-1.353985765	5.395139104	7.28E-07	4.14E-06
PARG11101	-1.357759427	5.182256923	7.31E-07	4.16E-06
PARG22245	1.854080454	1.602053726	7.38E-07	4.19E-06
PARG03546	-4.300704852	1.69777314	7.40E-07	4.20E-06
PARG12499	-4.29799602	0.268055051	7.40E-07	4.20E-06
PARG26790	-4.290686499	-0.492269306	7.40E-07	4.20E-06
PARG07936	1.332574161	8.165253566	7.41E-07	4.20E-06
PARG13125	-1.943176585	0.733973652	7.42E-07	4.21E-06
PARG21257	1.920545704	0.946926832	7.42E-07	4.21E-06
PARG23227	1.367500646	4.762499613	7.45E-07	4.22E-06
PARG12363	-1.526702476	3.099034891	7.46E-07	4.23E-06
PARG22388	1.687208963	1.289624856	7.51E-07	4.25E-06
PARG16653	-2.431149851	0.984941922	7.53E-07	4.27E-06
PARG28413	-1.387141778	4.362629368	7.55E-07	4.27E-06
PARG08536	-1.461917889	2.578504183	7.57E-07	4.28E-06
PARG30216	1.365481148	5.026913052	7.61E-07	4.30E-06
PARG27842	-1.334797476	7.576916443	7.63E-07	4.31E-06
PARG01195	-1.387955444	3.812353391	7.67E-07	4.33E-06
PARG06383	-1.502351832	3.354444838	7.67E-07	4.33E-06
PARG02914	1.380477671	4.757831897	7.71E-07	4.36E-06
PARG26903	-1.361798515	5.240034147	7.74E-07	4.37E-06
PARG18443	-1.486332568	3.568764174	7.80E-07	4.40E-06
PARG20930	1.370624145	4.661118374	7.86E-07	4.44E-06
PARG03288	-1.551373023	2.754348177	8.01E-07	4.52E-06
PARG06455	-1.415972243	3.944129086	8.04E-07	4.53E-06
PARG15836	-2.885141678	0.136273503	8.06E-07	4.54E-06
PARG01808	-2.623576009	-0.417199382	8.11E-07	4.57E-06
PARG20051	1.33484956	6.173203268	8.13E-07	4.58E-06
PARG05667	-1.797381135	2.447811677	8.15E-07	4.59E-06
PARG12634	-2.279289624	0.932480733	8.15E-07	4.59E-06
PARG13749	-5.994016627	-0.740979342	8.23E-07	4.63E-06
PARG19520	1.380133442	3.980592388	8.40E-07	4.73E-06
PARG27372	-1.469936372	3.41196716	8.45E-07	4.75E-06
PARG07221	-1.324693222	9.010669647	8.45E-07	4.75E-06
PARG15677	-3.419037186	0.300163246	8.53E-07	4.79E-06
PARG20560	3.082746769	0.013994941	8.53E-07	4.79E-06
PARG20330	-1.34913433	5.104751548	8.55E-07	4.80E-06
PARG06088	-1.748240337	1.424567026	8.66E-07	4.86E-06
PARG19595	1.330189697	6.530654787	8.66E-07	4.86E-06
PARG12210	1.351008046	6.317377973	8.79E-07	4.93E-06
PARG19189	1.431136023	2.739874172	8.81E-07	4.94E-06
PARG20751	-1.877280474	0.099689513	8.84E-07	4.96E-06
PARG24616	-1.573500018	3.086488811	8.92E-07	5.00E-06
PARG18419	-1.656411475	1.615262206	8.96E-07	5.02E-06

PARG21008	-2.011030058	0.609658227	8.98E-07	5.03E-06
PARG15738	-1.92181681	1.369074865	8.98E-07	5.03E-06
PARG29546	-1.331981748	6.156810568	9.04E-07	5.06E-06
PARG27557	-1.319911802	8.628828149	9.22E-07	5.15E-06
PARG15113	-1.723513987	2.091463948	9.22E-07	5.15E-06
PARG10935	-1.591627635	2.344758102	9.23E-07	5.16E-06
PARG14203	1.327435756	6.538156517	9.28E-07	5.18E-06
PARG02382	-1.383559046	3.709757012	9.34E-07	5.21E-06
PARG20104	-1.353138824	4.888051356	9.41E-07	5.25E-06
PARG18810	-1.319951425	7.637751793	9.43E-07	5.26E-06
PARG13942	-1.43906943	2.3062654	9.58E-07	5.34E-06
PARG12657	-2.093149115	0.602914693	9.58E-07	5.34E-06
PARG12093	1.340896914	5.123452735	9.59E-07	5.35E-06
PARG25242	1.352698819	4.896936711	9.71E-07	5.41E-06
PARG12667	-1.420239632	4.204331668	9.73E-07	5.42E-06
PARG00170	-3.165299387	-0.276347653	9.77E-07	5.44E-06
PARG20913	-3.149206166	0.739283522	9.77E-07	5.44E-06
PARG02459	-1.367759575	4.695324743	9.77E-07	5.44E-06
PARG25200	-1.357540907	5.157504887	9.87E-07	5.49E-06
PARG28325	1.377785831	3.380321358	9.91E-07	5.51E-06
PARG28295	-1.348412486	5.48197224	9.95E-07	5.53E-06
PARG27770	1.346760987	4.535046772	9.96E-07	5.54E-06
PARG18117	1.359876471	5.511361803	9.98E-07	5.54E-06
PARG05535	-1.406318329	3.660412592	9.99E-07	5.55E-06
PARG16160	-1.423078366	3.720662291	1.00E-06	5.56E-06
PARG27437	1.532297506	2.719164219	1.02E-06	5.67E-06
PARG22883	1.365107332	4.347290856	1.03E-06	5.71E-06
PARG12443	-1.402970225	3.851072312	1.03E-06	5.73E-06
PARG24356	1.347233015	5.022060584	1.04E-06	5.77E-06
PARG15276	-2.448789271	0.431839293	1.04E-06	5.77E-06
PARG20553	1.361312254	4.581884899	1.05E-06	5.80E-06
PARG10538	-3.804305396	1.956951107	1.06E-06	5.85E-06
PARG27763	-3.768627347	0.811728237	1.06E-06	5.85E-06
PARG06311	1.32839484	6.073351398	1.06E-06	5.86E-06
PARG06513	-2.963966632	-0.491232608	1.06E-06	5.86E-06
PARG24621	-2.952632035	1.151572373	1.06E-06	5.86E-06
PARG13792	-1.638409903	2.104500706	1.07E-06	5.93E-06
PARG29653	-2.553162308	0.447979911	1.08E-06	5.96E-06
PARG08588	1.429337896	3.201108621	1.09E-06	5.99E-06
PARG29184	1.31892613	6.845282061	1.09E-06	6.00E-06
PARG14152	-1.728262129	2.560202103	1.09E-06	6.01E-06
PARG17192	1.34437248	4.758652127	1.10E-06	6.07E-06
PARG24075	1.344018891	4.710644141	1.11E-06	6.11E-06
PARG06348	2.134053674	1.141121741	1.11E-06	6.15E-06
PARG26931	2.129404757	0.015976944	1.11E-06	6.15E-06
PARG11961	-1.377069352	4.178824104	1.12E-06	6.15E-06

PARG16884	-1.373954436	4.163894723	1.12E-06	6.15E-06
PARG28223	1.329881942	6.095791522	1.12E-06	6.19E-06
PARG20633	-2.753237736	-0.221498102	1.13E-06	6.20E-06
PARG27049	-2.558637119	-0.479204938	1.13E-06	6.20E-06
PARG06914	-1.312383008	7.077480762	1.14E-06	6.29E-06
PARG12508	-1.320100121	6.199463476	1.15E-06	6.30E-06
PARG09657	1.317509971	6.375471852	1.15E-06	6.30E-06
PARG11513	1.462750011	3.569487066	1.16E-06	6.38E-06
PARG27109	-1.428553244	4.16831419	1.16E-06	6.38E-06
PARG12941	-1.317695068	6.667525358	1.16E-06	6.38E-06
PARG08585	2.090908125	0.535454447	1.18E-06	6.45E-06
PARG12191	-1.35075936	4.649704105	1.18E-06	6.46E-06
PARG26413	1.309062318	7.158748503	1.18E-06	6.46E-06
PARG19574	1.710925739	2.312713506	1.18E-06	6.47E-06
PARG20282	1.935555232	1.662147965	1.19E-06	6.53E-06
PARG27498	1.345365035	4.755645182	1.19E-06	6.53E-06
PARG28005	1.675537325	2.210333757	1.21E-06	6.62E-06
PARG25756	4.555827062	-0.430430688	1.21E-06	6.63E-06
PARG23883	-4.208813837	-0.153585195	1.21E-06	6.63E-06
PARG14380	-1.370800811	4.358436615	1.21E-06	6.63E-06
PARG21211	-1.365041059	5.817017394	1.21E-06	6.64E-06
PARG13033	-4.420786083	-0.213169565	1.22E-06	6.66E-06
PARG11097	-1.322317462	5.554014699	1.22E-06	6.67E-06
PARG11738	1.306206643	7.703579129	1.22E-06	6.67E-06
PARG00701	1.307957471	7.161368634	1.22E-06	6.69E-06
PARG27531	1.348932799	5.001166888	1.23E-06	6.74E-06
PARG03645	-1.407115833	4.566613657	1.24E-06	6.78E-06
PARG05515	1.308515021	7.368646396	1.24E-06	6.78E-06
PARG20206	-1.472980685	3.050267822	1.25E-06	6.84E-06
PARG14231	1.357421927	4.124942045	1.26E-06	6.87E-06
PARG11406	1.899425293	1.482498318	1.26E-06	6.87E-06
PARG21107	-1.571598797	3.036899121	1.27E-06	6.93E-06
PARG20106	1.414267454	2.978403391	1.28E-06	6.95E-06
PARG08987	-3.358919132	0.496925076	1.28E-06	6.97E-06
PARG08132	-1.435161388	3.434751264	1.29E-06	7.00E-06
PARG29742	-2.108512145	-0.025847452	1.29E-06	7.00E-06
PARG15573	-1.347759282	4.922628323	1.30E-06	7.09E-06
PARG08814	-1.535186553	1.872881356	1.31E-06	7.10E-06
PARG03203	1.59565127	2.43124357	1.34E-06	7.27E-06
PARG08724	-1.369084102	4.059254544	1.34E-06	7.28E-06
PARG27203	1.453235963	2.468882976	1.35E-06	7.35E-06
PARG05623	1.324705369	5.390123215	1.36E-06	7.36E-06
PARG07975	-1.971613464	1.390864777	1.36E-06	7.37E-06
PARG01329	-1.832356911	0.88935818	1.38E-06	7.47E-06
PARG11495	-1.358302046	4.287485826	1.39E-06	7.52E-06
PARG13592	1.364971645	4.573005227	1.39E-06	7.53E-06

PARG19009	-1.664183048	2.50167996	1.41E-06	7.62E-06
PARG07376	-1.534509064	2.197892928	1.41E-06	7.66E-06
PARG19110	-5.943020495	0.657404458	1.42E-06	7.67E-06
PARG20834	-5.940813065	-0.224643738	1.42E-06	7.67E-06
PARG11986	-5.934199	0.160293233	1.42E-06	7.67E-06
PARG06789	-5.932392261	-0.096835957	1.42E-06	7.67E-06
PARG28061	-1.419206876	3.84256517	1.42E-06	7.68E-06
PARG22393	1.354894331	4.243681972	1.42E-06	7.69E-06
PARG06713	-3.10810177	-0.253313692	1.44E-06	7.76E-06
PARG15557	-3.099111752	0.238386078	1.44E-06	7.76E-06
PARG12999	1.396297727	3.760536733	1.44E-06	7.79E-06
PARG16909	1.373135175	4.164644547	1.45E-06	7.82E-06
PARG10043	1.307772747	5.408374276	1.46E-06	7.88E-06
PARG28426	-1.552639842	2.007920794	1.47E-06	7.91E-06
PARG23001	1.30646237	6.153305934	1.47E-06	7.92E-06
PARG11921	-1.671265058	2.465199106	1.50E-06	8.08E-06
PARG21253	1.307876074	5.847692514	1.51E-06	8.14E-06
PARG27806	1.311368254	5.713231126	1.53E-06	8.24E-06
PARG18786	1.325028871	4.789449933	1.54E-06	8.28E-06
PARG27200	-1.300214245	6.536312249	1.54E-06	8.32E-06
PARG15114	1.291697162	8.352907915	1.55E-06	8.37E-06
PARG29952	2.577437212	0.63510468	1.56E-06	8.39E-06
PARG05147	1.29456078	7.391549878	1.57E-06	8.47E-06
PARG07124	-2.773360628	-0.174803532	1.58E-06	8.48E-06
PARG17632	2.721378935	0.278011081	1.58E-06	8.48E-06
PARG05882	1.877492207	1.766114897	1.60E-06	8.59E-06
PARG27726	-1.293015029	6.997191082	1.61E-06	8.65E-06
PARG06682	-1.309086061	5.377153059	1.62E-06	8.71E-06
PARG00282	-1.292967601	6.728061898	1.64E-06	8.79E-06
PARG08239	1.36284547	2.612002698	1.68E-06	8.99E-06
PARG15509	1.364857901	3.727893981	1.69E-06	9.05E-06
PARG02145	-1.443189413	3.517584567	1.71E-06	9.16E-06
PARG19509	1.969804101	0.320344363	1.72E-06	9.22E-06
PARG07328	-1.32855608	4.448691848	1.72E-06	9.22E-06
PARG26641	-1.285514324	8.230320877	1.72E-06	9.22E-06
PARG04362	1.373687052	4.019899494	1.73E-06	9.28E-06
PARG19464	1.292505601	6.770088687	1.75E-06	9.36E-06
PARG19504	-1.827969615	1.079991751	1.75E-06	9.36E-06
PARG12558	-1.305072268	5.585030485	1.75E-06	9.36E-06
PARG02409	-1.400277301	3.536062327	1.76E-06	9.40E-06
PARG27758	-2.437786193	1.976316936	1.76E-06	9.41E-06
PARG19020	-1.304448559	5.938165481	1.76E-06	9.41E-06
PARG02852	1.389647701	3.486654219	1.77E-06	9.47E-06
PARG23588	1.301185421	5.495612009	1.79E-06	9.54E-06
PARG22301	-1.297367968	5.391724431	1.80E-06	9.59E-06
PARG18551	-1.707111675	1.916683821	1.81E-06	9.67E-06

PARG15461	1.286628306	7.490916466	1.82E-06	9.70E-06
PARG21167	-1.455503913	3.528964651	1.86E-06	9.90E-06
PARG20616	-2.400952027	-0.582231229	1.87E-06	9.97E-06
PARG08246	1.284246293	6.984031266	1.87E-06	9.99E-06
PARG29654	1.319426412	5.14333846	1.90E-06	1.01E-05
PARG20052	1.316366955	4.723052669	1.91E-06	1.02E-05
PARG05621	-3.583135061	-0.286343718	1.93E-06	1.03E-05
PARG20683	1.291740178	6.17424666	1.93E-06	1.03E-05
PARG12315	1.637870972	1.140104985	1.93E-06	1.03E-05
PARG26656	2.354201781	0.365493808	1.98E-06	1.05E-05
PARG12591	2.335171963	2.322825645	1.98E-06	1.05E-05
PARG17120	-4.200259284	-0.021907818	1.99E-06	1.06E-05
PARG25424	-4.19650803	-0.058390617	1.99E-06	1.06E-05
PARG26460	-4.158656834	0.305286591	1.99E-06	1.06E-05
PARG13199	1.303492971	4.669330347	2.02E-06	1.07E-05
PARG07816	1.27623277	8.459370406	2.02E-06	1.07E-05
PARG06980	-1.276596025	8.142704598	2.03E-06	1.08E-05
PARG11353	1.402559623	2.685020612	2.03E-06	1.08E-05
PARG23407	-1.275283572	8.859156923	2.04E-06	1.08E-05
PARG00288	-1.805256615	1.694341369	2.05E-06	1.08E-05
PARG28290	-1.364867089	3.440724028	2.06E-06	1.09E-05
PARG15346	-2.423312457	0.141010007	2.07E-06	1.10E-05
PARG18545	-1.306767492	4.905183079	2.08E-06	1.10E-05
PARG17626	-1.432472862	2.880843563	2.08E-06	1.10E-05
PARG23593	-1.423132654	2.640126036	2.09E-06	1.11E-05
PARG22385	1.284747127	6.338325961	2.10E-06	1.11E-05
PARG06828	-1.97714965	2.645402868	2.11E-06	1.11E-05
PARG18583	-1.921716825	0.538403442	2.11E-06	1.11E-05
PARG21590	1.272267363	10.47696619	2.11E-06	1.12E-05
PARG22777	1.301467134	5.042745315	2.13E-06	1.13E-05
PARG23581	-1.287495884	5.848259632	2.14E-06	1.13E-05
PARG18336	-1.296159981	5.137162495	2.16E-06	1.14E-05
PARG03320	1.289389532	6.426663389	2.17E-06	1.15E-05
PARG05443	1.270363747	10.63866478	2.18E-06	1.15E-05
PARG19463	1.29638808	5.530033607	2.19E-06	1.15E-05
PARG24100	-1.281654894	6.168672478	2.21E-06	1.17E-05
PARG29426	2.801670914	-1.112211087	2.22E-06	1.17E-05
PARG24376	2.781887326	0.066971404	2.22E-06	1.17E-05
PARG26390	-1.360720572	3.542531066	2.22E-06	1.17E-05
PARG26873	1.273112175	7.674255979	2.22E-06	1.17E-05
PARG01825	-2.053013796	0.046157789	2.23E-06	1.17E-05
PARG18978	-1.372357238	4.378299673	2.24E-06	1.18E-05
PARG15826	-2.725478966	-0.62063238	2.24E-06	1.18E-05
PARG15511	1.376295039	2.916769195	2.26E-06	1.19E-05
PARG04723	-1.325636259	5.01094614	2.28E-06	1.20E-05
PARG18761	1.271364896	7.602952866	2.28E-06	1.20E-05

PARG29906	-1.396830582	3.10228328	2.29E-06	1.20E-05
PARG07670	-1.283903041	5.629482856	2.31E-06	1.22E-05
PARG23577	1.340538119	3.861025193	2.34E-06	1.23E-05
PARG20516	-1.270086482	7.731463654	2.34E-06	1.23E-05
PARG19354	1.459637824	2.218293478	2.34E-06	1.23E-05
PARG27392	1.266126896	9.377572034	2.39E-06	1.25E-05
PARG27766	-1.268996736	7.311528966	2.39E-06	1.25E-05
PARG07659	-1.323182436	4.492737737	2.40E-06	1.26E-05
PARG18171	1.403764745	2.858748237	2.41E-06	1.26E-05
PARG18727	1.347491722	3.510485264	2.41E-06	1.26E-05
PARG06971	-1.513514501	2.249963324	2.43E-06	1.27E-05
PARG20048	-5.88563607	-0.399534068	2.46E-06	1.28E-05
PARG03372	-5.882674902	0.232381647	2.46E-06	1.28E-05
PARG11294	1.273073838	6.398673256	2.50E-06	1.31E-05
PARG19988	1.285303966	6.258215379	2.50E-06	1.31E-05
PARG12493	-3.694444829	-0.945404972	2.51E-06	1.31E-05
PARG01215	-1.707856722	0.821923029	2.54E-06	1.33E-05
PARG01985	-1.681868723	2.410912888	2.56E-06	1.34E-05
PARG18864	1.281622666	5.611659429	2.57E-06	1.34E-05
PARG18507	-1.366718982	3.303972063	2.59E-06	1.35E-05
PARG08478	1.272463132	6.329424273	2.61E-06	1.36E-05
PARG03712	-1.408507625	2.893324981	2.61E-06	1.36E-05
PARG13247	-2.208387003	1.694137397	2.64E-06	1.38E-05
PARG15515	-1.836918933	0.739893135	2.65E-06	1.38E-05
PARG07105	1.689172839	1.685493989	2.65E-06	1.38E-05
PARG14440	-1.268665305	6.497550208	2.65E-06	1.38E-05
PARG20575	1.27179208	6.077444812	2.67E-06	1.39E-05
PARG29041	-1.260430296	8.582097063	2.69E-06	1.40E-05
PARG06442	1.406931289	2.704083164	2.70E-06	1.40E-05
PARG11038	1.362358423	3.356378759	2.70E-06	1.40E-05
PARG11299	1.312106762	3.826724602	2.72E-06	1.42E-05
PARG20458	-1.268722645	6.408404302	2.74E-06	1.43E-05
PARG01987	-1.290154949	5.219654668	2.77E-06	1.44E-05
PARG02919	-1.49180668	2.540106143	2.77E-06	1.44E-05
PARG02898	-1.291137392	5.022999534	2.79E-06	1.45E-05
PARG30014	-2.007319988	1.405761931	2.80E-06	1.45E-05
PARG03227	1.276238439	5.600281061	2.80E-06	1.46E-05
PARG07246	1.269960966	6.008975605	2.81E-06	1.46E-05
PARG20821	-1.436714271	2.539708579	2.82E-06	1.46E-05
PARG23989	2.344088393	-0.468047977	2.84E-06	1.47E-05
PARG05978	-1.273702356	6.062004319	2.86E-06	1.48E-05
PARG05324	1.282948136	5.045919685	2.88E-06	1.49E-05
PARG17395	-1.33754	4.696038336	2.89E-06	1.50E-05
PARG12953	1.260319065	6.816879394	2.89E-06	1.50E-05
PARG04913	1.321941816	3.696240753	2.89E-06	1.50E-05
PARG04811	-3.298433406	1.174416748	2.90E-06	1.50E-05

PARG27386	-1.362799713	3.321631744	2.91E-06	1.50E-05
PARG12392	-1.29224379	4.727185771	2.92E-06	1.51E-05
PARG26182	1.394814208	3.083344672	2.92E-06	1.51E-05
PARG13663	-1.262086039	6.444603576	2.93E-06	1.51E-05
PARG07417	1.258381672	6.254737116	2.96E-06	1.53E-05
PARG13789	-1.258665818	7.928135245	2.96E-06	1.53E-05
PARG12140	1.26353713	6.687927137	2.96E-06	1.53E-05
PARG11890	-1.350371563	3.413064476	2.97E-06	1.53E-05
PARG23824	-1.31454109	4.346950112	2.97E-06	1.53E-05
PARG28527	1.360094492	3.206882951	2.98E-06	1.54E-05
PARG12063	1.353494716	4.456679136	3.02E-06	1.56E-05
PARG15527	-1.381203776	4.227828533	3.06E-06	1.58E-05
PARG18206	1.44717953	4.064493402	3.06E-06	1.58E-05
PARG12008	-4.328489998	-0.379685346	3.07E-06	1.58E-05
PARG28034	-1.265278929	6.020184462	3.07E-06	1.58E-05
PARG13949	1.254955664	7.74634209	3.08E-06	1.58E-05
PARG00668	1.390609952	3.162110226	3.08E-06	1.59E-05
PARG19706	1.295727809	5.114285805	3.09E-06	1.59E-05
PARG29270	-1.285861739	5.089563469	3.11E-06	1.60E-05
PARG05747	1.407889474	2.992799961	3.12E-06	1.60E-05
PARG22091	-2.786471611	-0.57165214	3.12E-06	1.60E-05
PARG22015	1.264404447	6.324131493	3.12E-06	1.60E-05
PARG19404	-1.771058666	1.741907913	3.13E-06	1.61E-05
PARG11410	-1.29261855	5.017169278	3.15E-06	1.62E-05
PARG20426	1.322767368	3.819389159	3.15E-06	1.62E-05
PARG00582	1.267148512	5.422641511	3.16E-06	1.62E-05
PARG28578	1.38901176	2.942246033	3.17E-06	1.63E-05
PARG20659	1.252473623	7.718544813	3.18E-06	1.63E-05
PARG15377	-1.268023461	4.982703791	3.18E-06	1.63E-05
PARG00061	1.339004547	3.491315184	3.22E-06	1.65E-05
PARG28526	-1.618316403	1.489079424	3.26E-06	1.67E-05
PARG11378	1.370810115	2.904662446	3.27E-06	1.67E-05
PARG13433	-4.152212848	0.085287895	3.29E-06	1.68E-05
PARG24822	3.641033845	-0.349431678	3.29E-06	1.68E-05
PARG13571	-1.74775048	1.661081222	3.34E-06	1.71E-05
PARG29182	1.338516397	3.145311984	3.36E-06	1.72E-05
PARG16509	-2.289482717	4.35752533	3.36E-06	1.72E-05
PARG27350	2.228598929	0.649622713	3.36E-06	1.72E-05
PARG07446	1.277495746	5.318918191	3.37E-06	1.72E-05
PARG22725	1.25456639	6.806012325	3.39E-06	1.73E-05
PARG05632	1.277139567	5.027518054	3.40E-06	1.74E-05
PARG06064	1.252497141	6.863741693	3.41E-06	1.74E-05
PARG03541	-1.264715118	5.667524732	3.42E-06	1.74E-05
PARG26921	-1.887014638	1.949575343	3.43E-06	1.75E-05
PARG01391	1.766794301	0.092248019	3.43E-06	1.75E-05
PARG00797	-1.638183503	1.490970947	3.48E-06	1.78E-05

PARG17683	1.257558484	5.652662475	3.50E-06	1.79E-05
PARG01446	-1.264679195	6.015171734	3.56E-06	1.81E-05
PARG27980	-1.255386973	6.349339303	3.58E-06	1.82E-05
PARG14802	-1.319593322	3.418117477	3.61E-06	1.84E-05
PARG28281	1.243994583	8.405189299	3.61E-06	1.84E-05
PARG10292	1.277336815	4.816570086	3.61E-06	1.84E-05
PARG25211	2.291171651	0.312579932	3.63E-06	1.84E-05
PARG03177	1.252602805	6.179643614	3.63E-06	1.84E-05
PARG25029	-1.282095572	4.634970583	3.64E-06	1.85E-05
PARG07025	1.649502348	1.564639233	3.65E-06	1.85E-05
PARG14746	-1.979430928	0.336701804	3.65E-06	1.86E-05
PARG25315	-1.324779002	3.587927596	3.66E-06	1.86E-05
PARG24141	1.276275855	4.708144605	3.68E-06	1.87E-05
PARG26681	-1.32094637	3.685156192	3.70E-06	1.88E-05
PARG21433	1.243354116	8.167471081	3.71E-06	1.88E-05
PARG18606	-1.255345213	5.59481011	3.73E-06	1.89E-05
PARG19655	1.247099446	6.799717262	3.73E-06	1.89E-05
PARG07274	1.249579244	6.181224042	3.73E-06	1.89E-05
PARG12376	1.423425874	2.651894274	3.74E-06	1.90E-05
PARG27260	-1.259645084	5.22971963	3.78E-06	1.91E-05
PARG23020	1.247409444	6.894475731	3.78E-06	1.91E-05
PARG19136	-1.571164238	1.286306669	3.79E-06	1.92E-05
PARG09681	-1.278501987	4.643961578	3.81E-06	1.93E-05
PARG22544	1.251501359	6.013966955	3.82E-06	1.93E-05
PARG02315	-3.655593017	0.58181021	3.87E-06	1.96E-05
PARG06465	-3.654880586	0.225381463	3.87E-06	1.96E-05
PARG19458	2.32179001	-0.151468244	3.90E-06	1.97E-05
PARG24080	-1.277197104	4.921017057	3.91E-06	1.97E-05
PARG19093	1.463931834	2.044804126	3.91E-06	1.97E-05
PARG07374	-1.265064622	5.103967706	3.92E-06	1.98E-05
PARG23748	1.245977714	6.827089416	3.95E-06	1.99E-05
PARG20049	-1.251418812	5.861616334	3.96E-06	2.00E-05
PARG10860	1.249955212	6.452626584	4.04E-06	2.04E-05
PARG04447	1.425639345	2.544942432	4.06E-06	2.05E-05
PARG16993	1.250888373	5.515737803	4.09E-06	2.06E-05
PARG20688	-1.292104586	4.64891212	4.15E-06	2.09E-05
PARG10384	1.835007235	1.136842251	4.17E-06	2.10E-05
PARG22416	-1.268499219	4.497268083	4.22E-06	2.13E-05
PARG04516	-1.409693343	2.395056625	4.27E-06	2.15E-05
PARG10753	-5.822971555	-0.515223733	4.27E-06	2.15E-05
PARG03746	-5.822730701	0.17143826	4.27E-06	2.15E-05
PARG25755	-5.812171641	0.099634831	4.27E-06	2.15E-05
PARG07686	1.23812398	7.46448778	4.28E-06	2.15E-05
PARG07774	-1.281070249	4.601178516	4.30E-06	2.16E-05
PARG24010	1.232220769	9.907944477	4.30E-06	2.16E-05
PARG25376	1.239421284	6.868450664	4.37E-06	2.19E-05

PARG26406	-1.281759747	4.355807192	4.37E-06	2.19E-05
PARG20791	-2.556497129	0.883419628	4.38E-06	2.20E-05
PARG10505	-2.540403123	1.293592	4.38E-06	2.20E-05
PARG23228	1.253057799	5.406573959	4.44E-06	2.23E-05
PARG10862	-1.314877274	3.54128717	4.45E-06	2.23E-05
PARG29830	1.954356641	-0.317046304	4.47E-06	2.24E-05
PARG11073	1.547998602	2.190560114	4.47E-06	2.24E-05
PARG25306	1.271767199	4.410676778	4.48E-06	2.24E-05
PARG14374	1.315260134	3.847400436	4.50E-06	2.26E-05
PARG06485	-2.677006629	0.141656098	4.56E-06	2.29E-05
PARG10239	1.423080785	2.269658181	4.62E-06	2.31E-05
PARG26984	-1.921963925	1.138289445	4.64E-06	2.32E-05
PARG03416	-2.821976998	0.572630058	4.65E-06	2.33E-05
PARG07026	1.359244765	2.885107137	4.67E-06	2.33E-05
PARG16606	1.25425921	5.62742316	4.67E-06	2.33E-05
PARG12353	-1.439947722	2.178039673	4.69E-06	2.34E-05
PARG02521	1.240865215	5.721933285	4.89E-06	2.44E-05
PARG12785	-3.491133929	0.195771244	4.89E-06	2.44E-05
PARG19332	-1.290786891	3.615467277	4.90E-06	2.45E-05
PARG02123	-2.298317328	0.729159097	4.92E-06	2.46E-05
PARG20559	-1.230781073	6.916423691	4.94E-06	2.46E-05
PARG26535	-1.779528489	1.558447756	4.94E-06	2.46E-05
PARG13501	-1.604734481	1.318634776	4.95E-06	2.47E-05
PARG27611	-1.309193258	4.380568215	4.96E-06	2.47E-05
PARG10546	2.14961488	-0.718897455	4.98E-06	2.48E-05
PARG12996	-1.225461557	8.3130721	5.01E-06	2.49E-05
PARG07593	-1.283290652	4.398534012	5.05E-06	2.52E-05
PARG27302	-1.252324861	5.440926645	5.08E-06	2.53E-05
PARG08590	1.918084022	0.435782367	5.08E-06	2.53E-05
PARG12776	-1.244048716	5.103376646	5.12E-06	2.55E-05
PARG09145	-1.878924749	0.48715025	5.12E-06	2.55E-05
PARG25334	1.279197864	4.009858387	5.14E-06	2.55E-05
PARG30344	1.354222306	2.387333547	5.16E-06	2.56E-05
PARG07154	1.221860525	9.173340045	5.17E-06	2.57E-05
PARG19076	-1.269999389	3.89839084	5.20E-06	2.59E-05
PARG23543	1.311572558	2.629719502	5.24E-06	2.60E-05
PARG27044	-1.690486147	1.347684647	5.35E-06	2.66E-05
PARG02040	-1.341070459	3.047941756	5.38E-06	2.67E-05
PARG05768	-4.06074264	-0.562006937	5.45E-06	2.70E-05
PARG19858	1.226294735	6.639986856	5.50E-06	2.73E-05
PARG03282	1.252332519	4.539920951	5.51E-06	2.73E-05
PARG21584	-1.259727974	4.862512125	5.52E-06	2.74E-05
PARG21635	-1.245520911	5.73450494	5.57E-06	2.76E-05
PARG28226	-1.518741094	1.612181286	5.57E-06	2.76E-05
PARG29941	-1.755605956	2.691395337	5.63E-06	2.79E-05
PARG07114	1.234944603	5.958934416	5.68E-06	2.81E-05

PARG26359	1.851572486	0.390720973	5.69E-06	2.82E-05
PARG22599	1.803549644	1.838837393	5.69E-06	2.82E-05
PARG27603	-1.325200793	3.703935002	5.70E-06	2.82E-05
PARG00782	-1.219599391	7.295385322	5.75E-06	2.85E-05
PARG02066	-2.603186437	0.73519827	5.77E-06	2.85E-05
PARG21274	1.219118095	7.050805223	5.84E-06	2.89E-05
PARG03083	-1.258610187	4.483011096	5.98E-06	2.95E-05
PARG16207	1.22550285	6.040987605	6.03E-06	2.98E-05
PARG21372	-1.64307492	0.853381604	6.04E-06	2.98E-05
PARG27166	-1.788401686	2.214163724	6.04E-06	2.98E-05
PARG05568	-2.327054724	-0.302447387	6.06E-06	2.99E-05
PARG05391	-2.212104651	-0.58967314	6.06E-06	2.99E-05
PARG18763	-1.251574139	4.875905563	6.09E-06	3.00E-05
PARG25144	-1.794819532	1.237169338	6.16E-06	3.04E-05
PARG19282	-1.281172564	3.804657992	6.16E-06	3.04E-05
PARG29655	-2.131904923	1.155512722	6.17E-06	3.04E-05
PARG19514	1.213340266	7.990235175	6.19E-06	3.05E-05
PARG11887	1.693817487	0.422389711	6.21E-06	3.06E-05
PARG03633	1.260960195	4.453344333	6.26E-06	3.08E-05
PARG13619	1.573755944	1.743045126	6.38E-06	3.14E-05
PARG21218	-1.970624356	0.474586821	6.39E-06	3.14E-05
PARG24654	1.218649776	6.611357302	6.43E-06	3.16E-05
PARG08405	-1.221121833	6.650318574	6.46E-06	3.18E-05
PARG04943	1.209948443	8.448925672	6.50E-06	3.20E-05
PARG12002	-1.229581374	5.411318899	6.60E-06	3.24E-05
PARG19366	-1.236370627	5.116499026	6.65E-06	3.26E-05
PARG11654	1.301247452	3.217095844	6.66E-06	3.27E-05
PARG00127	1.227132699	5.266758854	6.67E-06	3.27E-05
PARG03266	1.210888012	7.141177913	6.67E-06	3.27E-05
PARG07636	1.225189342	5.603233046	6.71E-06	3.29E-05
PARG10889	1.436202813	2.060222266	6.72E-06	3.29E-05
PARG18960	-1.378656932	3.192804626	6.75E-06	3.31E-05
PARG06852	-1.209839637	8.241436504	6.76E-06	3.31E-05
PARG17986	2.791471596	0.657213728	6.82E-06	3.34E-05
PARG23266	-1.247305195	4.756334073	6.83E-06	3.34E-05
PARG06265	-1.310617976	3.241414214	6.83E-06	3.34E-05
PARG08638	-1.352557104	2.288925127	6.84E-06	3.34E-05
PARG27314	-1.241028377	4.64724066	7.03E-06	3.44E-05
PARG21071	1.209260506	7.105238699	7.06E-06	3.45E-05
PARG04034	-1.234917128	5.423561355	7.06E-06	3.45E-05
PARG08512	1.99403753	0.526301005	7.08E-06	3.46E-05
PARG11232	1.278071255	3.764142152	7.22E-06	3.53E-05
PARG15331	-1.267021352	3.857849129	7.24E-06	3.53E-05
PARG12225	-1.307299313	3.257683333	7.24E-06	3.54E-05
PARG03022	1.214345144	6.205182464	7.25E-06	3.54E-05
PARG15503	-2.133896536	1.146272481	7.31E-06	3.57E-05

PARG03500	-2.490678304	0.012503868	7.33E-06	3.58E-05
PARG29892	-1.597249967	1.642162265	7.34E-06	3.58E-05
PARG03687	1.23055585	4.82099502	7.45E-06	3.63E-05
PARG21041	-5.756437841	0.299208664	7.48E-06	3.65E-05
PARG02254	-1.204676249	7.44194783	7.58E-06	3.69E-05
PARG08376	-1.573296443	1.321084942	7.59E-06	3.70E-05
PARG27430	1.671492744	1.566432098	7.61E-06	3.71E-05
PARG19429	-1.503349022	1.784316729	7.64E-06	3.72E-05
PARG15978	1.213325539	5.810100932	7.64E-06	3.72E-05
PARG19122	-1.280994858	3.280860798	7.65E-06	3.72E-05
PARG24430	-1.234246726	4.442710299	7.67E-06	3.73E-05
PARG06644	-2.14067397	0.371622124	7.72E-06	3.76E-05
PARG06203	1.607272089	2.140488262	7.77E-06	3.78E-05
PARG26046	1.392162422	1.891652247	7.79E-06	3.78E-05
PARG10628	1.19793405	9.291833768	7.79E-06	3.79E-05
PARG07037	1.203625542	6.761330456	7.85E-06	3.81E-05
PARG21458	-1.262900979	3.802252576	7.91E-06	3.84E-05
PARG02948	-1.261883252	3.634524354	7.92E-06	3.84E-05
PARG09360	-1.37625797	1.862848489	7.93E-06	3.85E-05
PARG26949	1.855915973	-0.234670678	7.96E-06	3.86E-05
PARG25739	1.230866041	5.688008721	7.99E-06	3.87E-05
PARG20008	-2.401612865	0.578418139	8.01E-06	3.88E-05
PARG13314	-2.024122955	0.535921767	8.05E-06	3.90E-05
PARG01443	-2.008050411	-0.266973748	8.05E-06	3.90E-05
PARG12422	-1.213752655	5.144629054	8.06E-06	3.90E-05
PARG07608	1.204446063	6.710898979	8.06E-06	3.90E-05
PARG06672	-1.224681587	4.913052503	8.07E-06	3.90E-05
PARG15710	1.295408381	3.707434863	8.11E-06	3.92E-05
PARG06350	-1.218169407	4.750428132	8.12E-06	3.93E-05
PARG21217	-1.215146942	5.001337209	8.14E-06	3.94E-05
PARG16092	1.217705689	5.313127239	8.15E-06	3.94E-05
PARG24167	1.202825692	6.378858156	8.20E-06	3.96E-05
PARG07011	1.196511254	8.367268854	8.21E-06	3.97E-05
PARG26988	-1.536496643	3.190179706	8.22E-06	3.97E-05
PARG13132	1.417449501	2.520930887	8.23E-06	3.97E-05
PARG15518	-1.209514278	5.745345806	8.28E-06	3.99E-05
PARG05775	-1.196433166	7.995077273	8.30E-06	4.00E-05
PARG06019	-1.222909411	4.905538654	8.48E-06	4.09E-05
PARG10764	1.222397165	5.092036202	8.52E-06	4.11E-05
PARG05690	-1.281923823	3.906140786	8.52E-06	4.11E-05
PARG22410	-1.249700224	3.821210816	8.53E-06	4.11E-05
PARG12303	-1.550229245	2.071336643	8.54E-06	4.11E-05
PARG13738	1.203491083	6.244267879	8.60E-06	4.14E-05
PARG00575	-1.318884303	3.698318065	8.60E-06	4.14E-05
PARG27747	-1.386514824	1.770797399	8.61E-06	4.14E-05
PARG20697	-2.525175242	-0.135226624	8.69E-06	4.18E-05

PARG20259	-1.65575184	1.224511583	8.70E-06	4.19E-05
PARG27667	-1.319100762	3.659686546	8.74E-06	4.20E-05
PARG06468	-1.487114642	2.123663801	8.75E-06	4.21E-05
PARG08362	1.196269118	6.650621278	8.86E-06	4.26E-05
PARG07409	-1.19644796	7.079841328	8.95E-06	4.30E-05
PARG22587	-1.4151079	2.665811335	9.01E-06	4.32E-05
PARG26040	-4.007874686	0.207566226	9.05E-06	4.34E-05
PARG13064	-3.990977049	-0.514614908	9.05E-06	4.34E-05
PARG09068	1.909113867	-0.217105175	9.06E-06	4.35E-05
PARG26234	2.230904463	-0.427119988	9.06E-06	4.35E-05
PARG17247	-2.063355879	1.179292267	9.07E-06	4.35E-05
PARG12611	-2.052385877	0.6275874	9.07E-06	4.35E-05
PARG10556	-1.274887012	5.517692902	9.08E-06	4.35E-05
PARG02369	-1.252183328	3.955319524	9.12E-06	4.37E-05
PARG20511	-1.475976878	1.835073138	9.14E-06	4.38E-05
PARG00707	1.276433872	3.403853516	9.18E-06	4.39E-05
PARG14598	1.270505776	4.319342742	9.23E-06	4.42E-05
PARG27256	-1.639481758	0.693403257	9.25E-06	4.42E-05
PARG27319	-3.576891873	0.405898957	9.30E-06	4.45E-05
PARG13416	1.194530658	6.487998227	9.31E-06	4.45E-05
PARG21078	-1.307981237	3.07086856	9.31E-06	4.45E-05
PARG01364	-1.22381262	4.842295545	9.32E-06	4.45E-05
PARG16516	-1.825164979	4.519420844	9.37E-06	4.48E-05
PARG02498	1.753097394	1.048423426	9.37E-06	4.48E-05
PARG08803	1.204767193	4.598687261	9.43E-06	4.50E-05
PARG03078	-1.303880044	2.936203346	9.44E-06	4.51E-05
PARG09031	-1.186200612	9.738854015	9.44E-06	4.51E-05
PARG16536	1.203900815	5.099536989	9.45E-06	4.51E-05
PARG27626	1.218254594	4.903484958	9.48E-06	4.52E-05
PARG25480	-1.212959463	4.873303476	9.53E-06	4.54E-05
PARG11546	1.195076937	6.708553958	9.53E-06	4.54E-05
PARG05388	1.213900354	5.177529475	9.57E-06	4.56E-05
PARG05446	1.3697662	3.316436006	9.58E-06	4.56E-05
PARG18466	-1.191887386	6.912754759	9.68E-06	4.61E-05
PARG05199	-1.384867305	2.880623123	9.70E-06	4.62E-05
PARG27292	-1.323197522	3.104450628	9.75E-06	4.64E-05
PARG02257	-1.225783301	4.405064991	9.79E-06	4.66E-05
PARG08122	-2.738628108	1.840639881	9.82E-06	4.67E-05
PARG13500	1.188278695	7.186434879	9.86E-06	4.69E-05
PARG14214	1.187008806	7.360605861	9.86E-06	4.69E-05
PARG19186	-1.188165972	7.173874908	9.98E-06	4.74E-05
PARG10673	-3.187036014	-0.683128191	9.99E-06	4.75E-05
PARG26488	1.263016994	5.419400446	1.00E-05	4.75E-05
PARG12819	-2.942485049	0.32649604	1.01E-05	4.79E-05
PARG15094	-2.937285476	-0.181554205	1.01E-05	4.79E-05
PARG01099	-2.915243265	1.030803365	1.01E-05	4.79E-05

PARG15060	-1.207726028	5.294390183	1.02E-05	4.82E-05
PARG24427	1.1842163	7.929451525	1.03E-05	4.86E-05
PARG12071	1.186568148	7.492839498	1.03E-05	4.87E-05
PARG05629	1.181554171	8.948321804	1.04E-05	4.92E-05
PARG10661	1.189167206	6.012074806	1.04E-05	4.95E-05
PARG17734	1.180515279	9.207364586	1.05E-05	4.95E-05
PARG19412	1.219897017	4.603975683	1.05E-05	4.97E-05
PARG13972	1.702481883	0.918981543	1.06E-05	5.00E-05
PARG09161	1.188916862	6.378475316	1.06E-05	5.00E-05
PARG11449	-1.270964842	3.141450412	1.06E-05	5.02E-05
PARG23180	1.23137591	4.163730642	1.07E-05	5.06E-05
PARG23847	1.179813269	8.241946229	1.08E-05	5.12E-05
PARG20334	-1.209893482	4.756342589	1.09E-05	5.16E-05
PARG04072	1.202435927	5.025027609	1.10E-05	5.19E-05
PARG24555	1.304735576	2.757381717	1.10E-05	5.20E-05
PARG18001	-1.299646043	2.381868723	1.11E-05	5.22E-05
PARG29682	-1.299646043	2.381868723	1.11E-05	5.22E-05
PARG29703	-1.312343498	2.340640621	1.11E-05	5.23E-05
PARG27061	1.526637039	1.710632642	1.11E-05	5.24E-05
PARG27122	1.526637039	1.710632642	1.11E-05	5.24E-05
PARG12394	-2.376268816	0.633074461	1.11E-05	5.25E-05
PARG27174	-1.256437499	3.958396495	1.12E-05	5.26E-05
PARG16936	-1.940888709	0.099150971	1.12E-05	5.28E-05
PARG19209	-1.894424313	1.193453237	1.12E-05	5.28E-05
PARG14404	1.184845643	6.232855195	1.14E-05	5.36E-05
PARG23831	1.186361632	6.198429887	1.14E-05	5.36E-05
PARG00627	-1.519274124	1.054845156	1.14E-05	5.37E-05
PARG12169	1.206779194	4.404349103	1.14E-05	5.38E-05
PARG10235	-1.904574957	0.289634746	1.15E-05	5.39E-05
PARG10677	1.177463861	8.158861569	1.15E-05	5.43E-05
PARG04156	-1.181971106	6.259931054	1.16E-05	5.45E-05
PARG06684	1.338562006	2.277409892	1.16E-05	5.47E-05
PARG04475	1.192551034	5.199660486	1.18E-05	5.54E-05
PARG23159	1.187744199	5.610484423	1.19E-05	5.57E-05
PARG22987	-1.196582833	5.458406826	1.19E-05	5.58E-05
PARG00510	-1.872546524	1.551415972	1.19E-05	5.59E-05
PARG13914	1.173690348	8.513126139	1.19E-05	5.59E-05
PARG23981	1.175324295	7.759042095	1.20E-05	5.63E-05
PARG03070	-1.177318552	6.957978578	1.20E-05	5.64E-05
PARG12295	1.277396527	3.551923873	1.20E-05	5.64E-05
PARG13713	-1.706193509	1.432478477	1.21E-05	5.68E-05
PARG06506	1.181709808	6.401120152	1.22E-05	5.70E-05
PARG18342	-1.551758081	1.308405025	1.22E-05	5.71E-05
PARG06941	-1.371453659	1.696567375	1.22E-05	5.73E-05
PARG05663	1.180390155	6.49370336	1.23E-05	5.75E-05
PARG20612	1.262169119	3.371005652	1.23E-05	5.76E-05

PARG00223	-2.349343549	1.170856566	1.23E-05	5.76E-05
PARG27208	1.217404785	4.426526358	1.24E-05	5.78E-05
PARG21306	-1.323592011	3.055155624	1.24E-05	5.78E-05
PARG12245	-1.453758423	5.044094102	1.24E-05	5.79E-05
PARG27973	1.174465419	7.274100799	1.24E-05	5.79E-05
PARG29277	-4.201418715	0.752614223	1.25E-05	5.85E-05
PARG02454	-4.153588669	1.436849097	1.25E-05	5.85E-05
PARG05964	-3.399193439	0.613966333	1.25E-05	5.85E-05
PARG04550	-1.213654075	4.371124544	1.27E-05	5.92E-05
PARG11630	1.281116115	2.960652511	1.28E-05	5.95E-05
PARG11967	-1.212115024	4.132460813	1.28E-05	5.95E-05
PARG23986	1.227554942	4.708093487	1.28E-05	5.99E-05
PARG03890	-1.749829513	0.915940804	1.30E-05	6.04E-05
PARG00757	-1.648940373	1.060957798	1.30E-05	6.07E-05
PARG16454	1.172124498	6.874520781	1.31E-05	6.11E-05
PARG19477	1.167004611	9.371832444	1.31E-05	6.11E-05
PARG01390	-5.690659755	-0.940886118	1.32E-05	6.12E-05
PARG11340	-5.689048582	-0.666431697	1.32E-05	6.12E-05
PARG07858	-5.688065959	0.264850635	1.32E-05	6.12E-05
PARG14378	-2.559843121	-0.214179963	1.33E-05	6.20E-05
PARG27769	-1.343213468	2.231039948	1.34E-05	6.22E-05
PARG24155	-1.58916917	1.7752943	1.34E-05	6.23E-05
PARG06848	1.326142613	3.091637062	1.35E-05	6.26E-05
PARG19434	-1.837967011	0.920098209	1.35E-05	6.27E-05
PARG07592	1.805295187	1.615770578	1.35E-05	6.27E-05
PARG03466	1.426479623	1.852270238	1.35E-05	6.28E-05
PARG02053	-1.176045629	6.467517825	1.35E-05	6.29E-05
PARG07144	-1.717803709	1.308506902	1.36E-05	6.31E-05
PARG19490	1.17224313	6.648269996	1.36E-05	6.31E-05
PARG20348	1.171195524	7.013054781	1.36E-05	6.31E-05
PARG26386	-1.176887107	6.218715263	1.38E-05	6.41E-05
PARG28158	1.166065919	8.22814956	1.38E-05	6.41E-05
PARG12632	-1.230633339	3.864182587	1.39E-05	6.42E-05
PARG01319	1.248264335	4.290587354	1.39E-05	6.43E-05
PARG24491	1.218758122	3.919922712	1.41E-05	6.55E-05
PARG24015	1.163868336	8.550982961	1.42E-05	6.56E-05
PARG04043	1.183758574	6.182003137	1.42E-05	6.57E-05
PARG27384	-1.189862113	5.109087623	1.43E-05	6.60E-05
PARG02772	-1.177853201	6.079804648	1.43E-05	6.60E-05
PARG18981	-2.722866725	-0.340127505	1.43E-05	6.60E-05
PARG06425	2.603457828	-0.471039751	1.43E-05	6.60E-05
PARG12700	-1.169572805	6.436882359	1.43E-05	6.61E-05
PARG06557	-1.754992812	0.490896618	1.43E-05	6.62E-05
PARG28190	1.179148071	4.700716044	1.43E-05	6.63E-05
PARG08087	-3.531516358	3.1084014	1.45E-05	6.68E-05
PARG27224	-1.168919776	6.336220446	1.45E-05	6.69E-05

PARG11586	-1.212798671	4.421847562	1.46E-05	6.73E-05
PARG05427	1.26082773	3.793784487	1.47E-05	6.77E-05
PARG10430	-1.173476257	6.016847364	1.47E-05	6.77E-05
PARG03660	-1.465616386	1.64284285	1.47E-05	6.78E-05
PARG10683	-1.205544934	4.406101421	1.48E-05	6.83E-05
PARG00727	1.164526977	6.988917378	1.49E-05	6.84E-05
PARG18564	-1.164356148	7.299972032	1.49E-05	6.84E-05
PARG12125	-1.941227356	1.252930894	1.49E-05	6.86E-05
PARG14926	-1.351094122	2.690898276	1.49E-05	6.87E-05
PARG24008	1.164258209	7.96974132	1.49E-05	6.87E-05
PARG06514	1.200794049	4.350504734	1.50E-05	6.89E-05
PARG23792	1.187442851	5.005161944	1.50E-05	6.90E-05
PARG15273	-3.943359505	-0.534749321	1.51E-05	6.94E-05
PARG06106	-2.022841229	2.402934412	1.51E-05	6.95E-05
PARG06613	-3.154125224	0.180831876	1.51E-05	6.96E-05
PARG19165	1.172951692	5.697434505	1.52E-05	6.97E-05
PARG04121	-1.166543194	6.407191956	1.52E-05	6.98E-05
PARG18520	-1.184950086	5.140086901	1.53E-05	7.03E-05
PARG13139	1.162898642	7.222873552	1.53E-05	7.05E-05
PARG19883	-1.848064989	0.984474317	1.54E-05	7.06E-05
PARG01239	1.161237605	7.394639801	1.54E-05	7.06E-05
PARG24345	1.314763426	2.650535688	1.54E-05	7.06E-05
PARG15656	1.156454202	10.13176524	1.54E-05	7.07E-05
PARG23628	1.176224613	5.455322956	1.55E-05	7.09E-05
PARG25043	-1.198315936	4.749061092	1.56E-05	7.13E-05
PARG20862	-1.314059085	2.667985335	1.56E-05	7.13E-05
PARG09258	1.19410779	4.165037279	1.56E-05	7.14E-05
PARG22165	1.286458472	2.113299356	1.57E-05	7.18E-05
PARG24132	1.206105485	4.098607281	1.57E-05	7.18E-05
PARG04382	1.241143667	3.517658674	1.59E-05	7.28E-05
PARG14279	1.156739996	8.245689883	1.60E-05	7.30E-05
PARG12645	1.161799236	6.323545423	1.60E-05	7.30E-05
PARG12527	1.190078834	4.78428241	1.60E-05	7.30E-05
PARG05152	1.850403847	-0.15793357	1.60E-05	7.33E-05
PARG02402	1.162160571	6.826951788	1.61E-05	7.36E-05
PARG27734	-1.216425252	3.934442233	1.61E-05	7.36E-05
PARG15821	-1.388038109	2.206902081	1.62E-05	7.38E-05
PARG21551	1.470614274	1.589189431	1.63E-05	7.44E-05
PARG07089	1.640358419	0.181952394	1.63E-05	7.46E-05
PARG07790	1.172986464	5.288371912	1.64E-05	7.49E-05
PARG00737	-1.392871536	1.876924222	1.66E-05	7.56E-05
PARG03636	1.378635725	1.686777877	1.66E-05	7.56E-05
PARG20460	1.165597035	6.07076849	1.66E-05	7.56E-05
PARG17823	-1.2404039	3.888494883	1.68E-05	7.64E-05
PARG27659	1.152373984	8.494143946	1.71E-05	7.81E-05
PARG22083	-1.161770421	6.093142195	1.72E-05	7.82E-05

PARG19681	1.186479434	4.548205559	1.72E-05	7.82E-05
PARG13938	-1.622002077	1.688844396	1.73E-05	7.86E-05
PARG03205	1.189885458	4.90584966	1.73E-05	7.86E-05
PARG15234	1.198555188	4.27714508	1.73E-05	7.89E-05
PARG24773	-1.156559165	6.996945944	1.74E-05	7.90E-05
PARG00619	-1.193003546	4.518857901	1.76E-05	7.99E-05
PARG03248	1.166348134	5.474151147	1.76E-05	8.01E-05
PARG25048	-1.160641302	5.980360737	1.78E-05	8.08E-05
PARG16045	1.211833661	3.921881591	1.82E-05	8.26E-05
PARG28264	1.288982997	2.743255805	1.82E-05	8.27E-05
PARG03933	-1.181940321	4.44977883	1.83E-05	8.29E-05
PARG27862	-1.160341996	5.782278416	1.83E-05	8.32E-05
PARG24129	1.164877267	5.133807375	1.84E-05	8.34E-05
PARG08640	-1.155998416	6.068458833	1.86E-05	8.44E-05
PARG07156	-1.24721292	3.361164096	1.89E-05	8.58E-05
PARG06694	2.183507867	1.999395229	1.90E-05	8.62E-05
PARG06172	-2.538235583	1.693839944	1.91E-05	8.64E-05
PARG19690	-2.531974628	-0.258133358	1.91E-05	8.64E-05
PARG02057	1.169572296	4.558947626	1.95E-05	8.83E-05
PARG22206	1.145486376	7.722527562	1.97E-05	8.90E-05
PARG12805	-1.203236717	4.428902698	1.99E-05	9.01E-05
PARG08876	2.010223156	-0.11220362	2.00E-05	9.03E-05
PARG10960	1.146618007	7.552704679	2.00E-05	9.05E-05
PARG17830	-1.22271476	4.405824828	2.01E-05	9.10E-05
PARG03744	-3.352686813	0.523113807	2.01E-05	9.10E-05
PARG11268	-1.203625041	3.495126704	2.02E-05	9.11E-05
PARG12438	-1.141713836	8.510512377	2.02E-05	9.13E-05
PARG08389	1.150576852	6.157587379	2.04E-05	9.21E-05
PARG19675	1.139345123	10.38896153	2.04E-05	9.23E-05
PARG20812	1.184406384	4.342669741	2.05E-05	9.26E-05
PARG03354	1.14173179	8.080617101	2.06E-05	9.28E-05
PARG06272	1.148701032	6.631792453	2.07E-05	9.32E-05
PARG10639	-1.175653554	6.158826819	2.07E-05	9.33E-05
PARG24674	-1.158856819	5.401559036	2.07E-05	9.33E-05
PARG12081	1.616404037	1.527196803	2.08E-05	9.36E-05
PARG08650	-1.257567065	3.419963781	2.08E-05	9.36E-05
PARG11000	-1.148584099	6.294159901	2.08E-05	9.39E-05
PARG10575	1.152573879	5.742242367	2.09E-05	9.43E-05
PARG12875	-1.192485967	4.75660091	2.09E-05	9.43E-05
PARG23832	1.169450485	5.512588284	2.10E-05	9.47E-05
PARG22167	1.16717267	5.013317476	2.11E-05	9.50E-05
PARG10486	1.851701058	1.559915913	2.12E-05	9.53E-05
PARG04038	-1.316078802	3.267692484	2.13E-05	9.58E-05
PARG12557	-1.189151897	4.019318509	2.13E-05	9.58E-05
PARG28297	1.13661128	10.70763995	2.13E-05	9.59E-05
PARG01389	-1.145164528	6.604708776	2.13E-05	9.59E-05

PARG18963	-1.266802659	2.380615255	2.14E-05	9.61E-05
PARG12228	-1.1677706	5.495995314	2.14E-05	9.61E-05
PARG01702	1.40607991	1.97978338	2.15E-05	9.65E-05
PARG06673	-1.617650004	0.668852055	2.15E-05	9.66E-05
PARG12619	2.209444043	0.308549717	2.16E-05	9.67E-05
PARG12462	1.141073543	7.487373399	2.16E-05	9.69E-05
PARG08331	-1.162674523	5.072911393	2.16E-05	9.71E-05
PARG25350	1.142367535	7.011507322	2.17E-05	9.73E-05
PARG17778	-1.196438846	3.668222305	2.18E-05	9.75E-05
PARG13349	1.634967758	1.499908998	2.19E-05	9.83E-05
PARG18187	1.153544407	6.314702069	2.20E-05	9.84E-05
PARG02473	1.317601636	3.031582355	2.20E-05	9.84E-05
PARG23911	-2.979348907	-0.276949453	2.22E-05	9.95E-05
PARG15428	1.177752311	4.349105813	2.23E-05	9.97E-05
PARG19670	1.341039584	2.476393412	2.23E-05	0.000100003
PARG17694	1.357896029	2.136225942	2.24E-05	0.000100198
PARG06157	-3.486850046	0.097607898	2.25E-05	0.000100682
PARG20563	-3.480780037	0.522960458	2.25E-05	0.000100682
PARG25395	-3.334315345	-0.402499413	2.25E-05	0.000100682
PARG13884	-1.224985012	3.484500667	2.27E-05	0.000101482
PARG13150	1.169286276	4.894834575	2.27E-05	0.000101543
PARG26824	1.186094589	4.256699546	2.27E-05	0.000101543
PARG26811	2.128206366	0.362003385	2.27E-05	0.000101557
PARG14488	-1.161166212	5.061025974	2.28E-05	0.000101888
PARG21390	-1.206033848	3.675102004	2.29E-05	0.000102102
PARG18401	-1.580182444	0.747138046	2.30E-05	0.000102543
PARG02182	1.158893664	5.095211916	2.30E-05	0.00010256
PARG05645	-1.222569487	3.310972091	2.30E-05	0.00010256
PARG28568	2.015329801	-0.441029984	2.30E-05	0.000102665
PARG13589	-5.625496742	0.095608972	2.33E-05	0.000103718
PARG24312	-5.62215872	0.027043272	2.33E-05	0.000103718
PARG19855	1.319546972	2.986615857	2.34E-05	0.000104182
PARG19700	1.149210205	5.746426559	2.34E-05	0.000104205
PARG03068	1.181337833	4.091994073	2.35E-05	0.000104474
PARG21871	1.216334108	3.530081895	2.36E-05	0.00010492
PARG16530	-1.137464052	6.725653605	2.36E-05	0.000105082
PARG02268	-1.165218845	4.959781317	2.36E-05	0.000105152
PARG21480	1.213286658	3.728258856	2.38E-05	0.000105591
PARG08003	1.671394155	0.136848753	2.39E-05	0.000106163
PARG20833	1.159957692	4.976455302	2.39E-05	0.000106163
PARG12021	1.139032514	6.521938636	2.39E-05	0.000106163
PARG15687	1.466940546	1.436770679	2.41E-05	0.000106792
PARG18495	1.146989583	5.901802227	2.41E-05	0.000106792
PARG24264	1.175368354	4.452505845	2.43E-05	0.000107658
PARG12849	1.13658713	6.523338543	2.43E-05	0.000107681
PARG17514	1.584867775	0.922918262	2.43E-05	0.000107681

PARG17496	1.569450437	0.948853526	2.43E-05	0.000107681
PARG02803	-2.384760319	0.124033504	2.44E-05	0.000107956
PARG25451	1.53228214	1.168756925	2.47E-05	0.000109509
PARG06361	1.16598415	4.815633687	2.47E-05	0.000109555
PARG23447	-1.136283203	6.827181923	2.49E-05	0.000110149
PARG01237	-1.147532106	5.478984661	2.49E-05	0.000110199
PARG16975	-1.238771571	4.350150547	2.50E-05	0.000110895
PARG14286	1.128100063	8.680396848	2.52E-05	0.000111318
PARG25706	1.270501859	2.802844049	2.52E-05	0.000111318
PARG26668	-1.151903453	5.38525118	2.52E-05	0.000111318
PARG27336	-3.893661559	-0.180688416	2.53E-05	0.000111783
PARG13131	1.126841396	8.975513916	2.55E-05	0.000112811
PARG19089	1.135581813	6.030336425	2.55E-05	0.000112811
PARG16088	-1.97241702	1.170973982	2.56E-05	0.000112975
PARG25790	-1.208466251	3.626623187	2.57E-05	0.000113336
PARG10720	-1.159831005	4.61497308	2.59E-05	0.000114523
PARG19288	1.15684328	4.737729332	2.63E-05	0.000116294
PARG03383	-2.173237672	-0.539014888	2.64E-05	0.000116359
PARG08424	1.151929507	5.136154843	2.64E-05	0.000116416
PARG05014	1.203443295	3.463927068	2.67E-05	0.000117568
PARG20471	-1.153461024	4.88056598	2.68E-05	0.000118031
PARG14411	-1.191728395	3.759096775	2.71E-05	0.00011936
PARG22030	1.134284385	5.895187422	2.72E-05	0.000119877
PARG25246	-2.392306362	0.285887738	2.73E-05	0.00012037
PARG25343	-2.392306362	0.285887738	2.73E-05	0.00012037
PARG09067	-1.4255294	1.850885824	2.74E-05	0.000120743
PARG24631	1.135134757	5.941709188	2.75E-05	0.000120928
PARG03675	-1.132758482	6.318397622	2.76E-05	0.000121416
PARG00435	-1.12247826	8.451843602	2.76E-05	0.000121473
PARG04839	1.134660512	5.908486522	2.78E-05	0.000122066
PARG03714	1.250678497	3.298491373	2.78E-05	0.000122207
PARG22932	1.175422832	4.169569611	2.79E-05	0.000122709
PARG07638	-1.374436617	1.955658445	2.79E-05	0.000122709
PARG24926	-1.13502502	5.518160808	2.80E-05	0.000123157
PARG29824	1.131536685	5.746839774	2.81E-05	0.000123226
PARG01392	-1.147644862	5.106696167	2.82E-05	0.000123925
PARG23689	1.337140205	3.407087493	2.84E-05	0.000124812
PARG25635	-1.359821454	1.480063459	2.85E-05	0.000124992
PARG11452	1.129864527	6.662854994	2.86E-05	0.000125348
PARG21271	-1.313307841	1.592635471	2.86E-05	0.000125442
PARG01244	1.189476165	3.719318701	2.87E-05	0.000125785
PARG06529	-1.120159067	8.537180342	2.88E-05	0.000126135
PARG03439	1.121078669	7.71342884	2.92E-05	0.000127783
PARG10069	-1.13182494	4.880705781	2.94E-05	0.000128774
PARG00496	-1.124369717	6.480334612	2.97E-05	0.000130123
PARG18974	-2.101232922	-0.174664373	2.98E-05	0.000130398

PARG00524	1.148669258	4.805868316	2.99E-05	0.000130743
PARG02888	-2.271600539	1.962408129	3.00E-05	0.00013133
PARG21314	-1.182819434	4.080257695	3.03E-05	0.000132526
PARG22661	-1.169610707	4.060067441	3.05E-05	0.000133607
PARG28238	1.141655021	5.15036515	3.06E-05	0.000133826
PARG15444	1.115662008	8.654300941	3.06E-05	0.000133885
PARG10964	-1.366020362	2.341566041	3.08E-05	0.000134815
PARG08795	-1.154447386	4.680392093	3.09E-05	0.000134898
PARG08766	-2.248458976	1.979604014	3.09E-05	0.000135065
PARG25403	-2.246558638	0.642262465	3.09E-05	0.000135065
PARG07588	-1.5551881	0.988269739	3.11E-05	0.000135677
PARG29276	1.143352104	4.934167439	3.12E-05	0.000136238
PARG24458	1.345945244	1.848402136	3.13E-05	0.000136658
PARG00298	1.117185911	7.537151783	3.13E-05	0.00013672
PARG01450	1.160839936	4.029994202	3.14E-05	0.00013713
PARG11280	1.173274838	3.737329298	3.16E-05	0.000137574
PARG24094	1.132292812	5.27118163	3.19E-05	0.000139185
PARG07582	-1.118751376	7.000605879	3.21E-05	0.000139897
PARG19052	1.125686282	5.609914425	3.23E-05	0.000140734
PARG00427	-1.663193289	1.746225504	3.23E-05	0.000140734
PARG06186	-1.117911085	6.680894482	3.24E-05	0.000141071
PARG23990	-1.148140428	4.754226178	3.26E-05	0.000142075
PARG15415	1.203754018	3.493068439	3.30E-05	0.00014354
PARG23365	1.122065984	6.011792393	3.30E-05	0.00014354
PARG27185	-1.279868824	2.005439064	3.30E-05	0.00014354
PARG26539	1.145504679	4.811504903	3.30E-05	0.000143629
PARG07265	1.111938715	7.599763653	3.33E-05	0.000144591
PARG28288	-1.1091153	9.747171006	3.34E-05	0.000145173
PARG02034	-1.145124702	4.575805386	3.34E-05	0.00014519
PARG26866	-1.318190602	2.295519968	3.35E-05	0.000145438
PARG00658	-1.160811258	3.74309245	3.37E-05	0.000146396
PARG12983	-1.174894206	2.666194093	3.38E-05	0.000146566
PARG15639	1.12298601	5.704457601	3.39E-05	0.000147191
PARG07291	-1.157890647	4.158598612	3.40E-05	0.000147347
PARG21809	-1.1616963	3.981368424	3.40E-05	0.000147374
PARG26653	-1.542407439	1.84578468	3.40E-05	0.000147438
PARG02412	-1.118682725	6.008901257	3.42E-05	0.000148305
PARG23933	-1.118065332	5.523510656	3.43E-05	0.000148588
PARG24294	-1.860982538	0.409172835	3.44E-05	0.000148842
PARG28000	-1.14827261	4.551861632	3.45E-05	0.000149572
PARG27827	1.11000039	7.926085041	3.48E-05	0.000150674
PARG18162	-1.521378234	1.148941012	3.48E-05	0.000150683
PARG13364	-3.065237514	1.675850814	3.49E-05	0.000151104
PARG16024	-3.058108338	0.345197933	3.49E-05	0.000151104
PARG18204	1.128057639	5.183355414	3.50E-05	0.000151301
PARG12176	-1.128131547	5.352947483	3.52E-05	0.000152078

PARG14274	1.380990442	0.716291692	3.52E-05	0.000152198
PARG24361	1.115579578	6.022133734	3.55E-05	0.00015335
PARG08589	1.207413301	2.807780939	3.56E-05	0.000153677
PARG02995	1.614504635	1.084773001	3.56E-05	0.000153831
PARG21687	-2.336527689	0.668228187	3.59E-05	0.000155194
PARG15358	1.332744135	1.414865268	3.60E-05	0.000155222
PARG22676	-1.114524411	6.819621926	3.60E-05	0.000155243
PARG25603	-1.165148194	4.076860547	3.62E-05	0.000156059
PARG22495	-1.326400202	1.596066338	3.64E-05	0.000157111
PARG06846	1.173534109	3.571271745	3.65E-05	0.000157577
PARG01419	1.15004894	4.331895432	3.67E-05	0.000158313
PARG15657	-1.114983	6.496899995	3.67E-05	0.000158313
PARG03765	-1.15956104	3.770238376	3.67E-05	0.000158313
PARG00019	-1.225638076	3.131287191	3.68E-05	0.000158446
PARG06113	1.106592509	7.559853062	3.70E-05	0.000159094
PARG16911	-1.641280653	0.859378807	3.71E-05	0.000159582
PARG01594	1.177454504	4.228334286	3.71E-05	0.000159823
PARG19804	1.112005257	6.174984143	3.76E-05	0.000161569
PARG27207	-1.202624641	3.169601505	3.79E-05	0.000162968
PARG01911	-1.444189268	1.424856936	3.83E-05	0.000164857
PARG15718	1.127737704	5.224769152	3.90E-05	0.000167474
PARG21874	-1.114692218	5.418147502	3.91E-05	0.00016791
PARG29145	-2.476091788	0.419855199	3.92E-05	0.000168306
PARG28992	-1.133800178	4.131926051	3.93E-05	0.000168991
PARG18430	-1.147404433	4.364866633	3.94E-05	0.000169331
PARG06907	1.108691072	6.347079733	3.95E-05	0.000169365
PARG18324	-1.706905183	-0.378679244	3.96E-05	0.000169829
PARG27164	-1.210324912	3.02104683	3.97E-05	0.000170286
PARG16677	-1.128895644	5.012666582	4.01E-05	0.000172177
PARG15661	-1.854449249	0.183629476	4.03E-05	0.00017266
PARG00438	-1.288909475	2.366610046	4.04E-05	0.000173335
PARG13061	-1.134633476	4.945934318	4.06E-05	0.000173899
PARG06730	-1.134325256	4.391768487	4.06E-05	0.000174153
PARG00291	1.15793272	3.130457507	4.07E-05	0.000174176
PARG23420	-1.222444163	2.949611253	4.07E-05	0.000174296
PARG29576	1.139604125	4.897857139	4.07E-05	0.000174324
PARG15083	-1.188844548	2.836451312	4.08E-05	0.000174443
PARG18460	-1.1928658	3.537708416	4.08E-05	0.000174519
PARG14978	-1.317715704	2.778926175	4.09E-05	0.000174761
PARG12064	2.06175486	1.441771991	4.09E-05	0.000175042
PARG15264	1.108504298	6.012603085	4.10E-05	0.000175184
PARG29114	-1.174193075	3.882046807	4.11E-05	0.00017564
PARG22248	-5.545322759	0.221252112	4.14E-05	0.000176955
PARG09054	-5.473305308	-0.647902168	4.14E-05	0.000176955
PARG15283	1.199815726	2.537971721	4.24E-05	0.000181141
PARG16567	-3.827884197	0.440165541	4.25E-05	0.000181304

PARG23237	1.129788103	4.845712803	4.25E-05	0.000181445
PARG00525	1.116011153	5.357897326	4.26E-05	0.000181642
PARG13241	1.624514905	0.690839142	4.27E-05	0.000182222
PARG10583	1.114470599	5.163912198	4.30E-05	0.00018356
PARG05478	-1.120333043	4.946136862	4.32E-05	0.000183992
PARG09194	-1.154107061	3.945671654	4.32E-05	0.00018416
PARG01141	-1.094115285	8.170363592	4.36E-05	0.000185717
PARG12377	1.220639889	2.816746316	4.36E-05	0.000185717
PARG01357	-1.473388434	1.188811612	4.38E-05	0.000186459
PARG08781	1.23849865	2.360062794	4.40E-05	0.000187394
PARG19820	-1.11374143	5.166725774	4.42E-05	0.000188233
PARG20152	-1.42472175	2.15412341	4.43E-05	0.000188528
PARG26377	-1.118618591	5.394514958	4.44E-05	0.000188993
PARG27904	-1.399050189	2.325520036	4.45E-05	0.000189439
PARG23209	1.336729063	2.159771366	4.52E-05	0.000192301
PARG07055	-1.200930802	3.095756519	4.54E-05	0.000192908
PARG18740	1.11953168	4.402515741	4.56E-05	0.00019375
PARG06868	1.095444996	6.938807938	4.58E-05	0.000194522
PARG21873	-1.111046133	4.920723574	4.59E-05	0.000195066
PARG07441	1.135639787	4.436097134	4.60E-05	0.000195415
PARG07181	1.967522391	-0.251549599	4.60E-05	0.000195415
PARG25356	1.136739364	5.254177487	4.63E-05	0.00019635
PARG24570	-1.320006524	2.015136156	4.63E-05	0.00019654
PARG25281	-1.461412646	1.532379703	4.65E-05	0.000197125
PARG20905	-1.08894407	8.987799735	4.67E-05	0.000198178
PARG20752	-1.638186687	0.239528543	4.69E-05	0.000198844
PARG06042	1.599331097	0.080146264	4.69E-05	0.000198844
PARG23256	1.314818633	1.264358881	4.70E-05	0.000199062
PARG11894	1.107140896	6.098953468	4.75E-05	0.000201272
PARG17636	1.094088221	6.305652418	4.79E-05	0.000202856
PARG19349	1.171651282	3.26997486	4.81E-05	0.000203721
PARG02732	1.104632988	5.550769986	4.83E-05	0.000204628
PARG12475	1.137604604	4.570337979	4.87E-05	0.0002061
PARG02221	-1.749378597	0.988965224	4.91E-05	0.000207546
PARG25050	-1.417849625	1.123556428	4.91E-05	0.00020756
PARG06863	-2.585965748	-0.042272448	4.91E-05	0.000207769
PARG05751	-1.095230665	6.515795258	4.94E-05	0.00020884
PARG03957	1.621437869	1.292233318	4.94E-05	0.000208944
PARG24635	-1.539263479	2.065760996	4.98E-05	0.000210288
PARG04255	-1.251302895	2.137945896	5.04E-05	0.000212691
PARG07640	-1.089291803	6.665827065	5.04E-05	0.000212927
PARG11904	-1.244942365	3.405231306	5.11E-05	0.000215669
PARG01146	1.264662634	2.452991564	5.11E-05	0.000215669
PARG11676	1.110515686	5.023110061	5.11E-05	0.000215669
PARG24733	-1.088695968	6.62512575	5.16E-05	0.000217768
PARG18130	-1.267572893	2.322971518	5.19E-05	0.000218773

PARG20707	1.081569288	8.879939571	5.19E-05	0.000218773
PARG28119	3.513371271	-0.400296914	5.23E-05	0.000220462
PARG10634	3.01969016	0.124935918	5.23E-05	0.000220462
PARG27891	1.085102519	7.202907701	5.23E-05	0.000220492
PARG25550	-1.133977402	4.391245867	5.25E-05	0.000220949
PARG02279	-1.093142982	5.78286669	5.27E-05	0.000221649
PARG19187	1.115389944	5.264298682	5.27E-05	0.000221812
PARG12647	1.083894028	7.290714744	5.30E-05	0.000222841
PARG14830	-3.020064413	-0.560072511	5.32E-05	0.000223535
PARG03770	-2.945385067	2.478522529	5.32E-05	0.000223535
PARG01257	1.083279181	7.711093823	5.32E-05	0.000223742
PARG25321	1.123852838	4.756482874	5.33E-05	0.000223883
PARG29401	1.088842942	6.60307597	5.33E-05	0.00022405
PARG02250	-1.099546639	6.033646884	5.36E-05	0.000225097
PARG07624	-1.089626112	6.201183714	5.36E-05	0.000225244
PARG10822	1.134487086	4.331170637	5.39E-05	0.000226317
PARG25228	1.087519145	6.758522906	5.39E-05	0.000226317
PARG30431	-1.486865641	0.953250041	5.43E-05	0.000227739
PARG13213	1.16147508	3.636389244	5.45E-05	0.000228569
PARG26822	1.148601036	3.588680124	5.49E-05	0.000230322
PARG13723	2.79219443	-0.671752507	5.49E-05	0.000230325
PARG07062	-1.084040814	7.535817997	5.50E-05	0.000230627
PARG21009	-1.196234063	3.185916766	5.53E-05	0.000231945
PARG26066	-1.115391426	4.816554083	5.56E-05	0.000233024
PARG26925	-1.168999412	3.525359646	5.57E-05	0.00023317
PARG07175	-1.090123622	5.663879891	5.59E-05	0.000233941
PARG18332	-1.090307238	5.952739549	5.59E-05	0.000234026
PARG20494	-2.438361884	0.772571637	5.62E-05	0.0002351
PARG08593	-2.420756386	0.114142644	5.62E-05	0.0002351
PARG09622	-1.114669565	5.076288106	5.65E-05	0.000236511
PARG25230	-1.079114327	7.66890272	5.66E-05	0.000236511
PARG28008	1.258285429	2.522699445	5.69E-05	0.000237839
PARG12503	1.521874517	0.507528065	5.69E-05	0.000237994
PARG20454	-1.082417251	6.779764225	5.70E-05	0.000238273
PARG22703	1.403191043	4.068749086	5.71E-05	0.00023843
PARG27994	-1.490995313	0.980156931	5.71E-05	0.000238542
PARG07617	1.075177143	9.816809576	5.72E-05	0.000238854
PARG29240	-1.088612426	6.13684648	5.73E-05	0.000239265
PARG27653	-1.099467647	5.25149697	5.76E-05	0.000240259
PARG26717	-1.111554396	4.693090487	5.76E-05	0.000240434
PARG07610	-2.380039449	0.644130272	5.82E-05	0.000242814
PARG04297	2.099318906	-0.337507203	5.82E-05	0.000242814
PARG22444	-1.083611135	6.01009161	5.83E-05	0.000243211
PARG27922	1.072753492	9.169491198	5.95E-05	0.000247885
PARG07569	-1.824756067	0.423718133	5.99E-05	0.00024938
PARG09800	-1.083475059	6.065534548	6.02E-05	0.000250652

PARG24465	-1.151766091	3.483548528	6.04E-05	0.000251494
PARG23661	1.091613419	5.557680256	6.06E-05	0.000252159
PARG18407	-1.416972374	1.027214281	6.07E-05	0.000252444
PARG21332	-1.140123995	4.085236275	6.07E-05	0.000252468
PARG12434	-1.337483773	2.021585119	6.07E-05	0.000252488
PARG11377	1.084726459	5.844374488	6.10E-05	0.000253457
PARG12056	-1.121758855	4.391300952	6.21E-05	0.000257965
PARG18826	-1.463433727	1.879168261	6.29E-05	0.000261498
PARG05756	1.073690583	7.049164834	6.29E-05	0.000261498
PARG29733	1.070760981	8.300541743	6.30E-05	0.000261788
PARG05276	-1.127659516	3.264149108	6.32E-05	0.000262461
PARG24351	1.547491374	2.332969445	6.32E-05	0.000262591
PARG06865	1.098377745	4.784082897	6.34E-05	0.000263287
PARG05103	-1.163067152	3.281559253	6.35E-05	0.000263424
PARG17542	1.109075047	4.762873234	6.35E-05	0.000263424
PARG04935	-1.094575455	5.202537552	6.39E-05	0.000264985
PARG18429	1.195203947	3.252575647	6.42E-05	0.000266401
PARG13062	1.105572524	4.474927783	6.45E-05	0.000267273
PARG29469	-1.075945495	5.846144173	6.45E-05	0.000267397
PARG13095	-1.068829196	8.138852535	6.48E-05	0.000268471
PARG02012	1.109766967	4.422995654	6.53E-05	0.000270327
PARG27617	-1.068934928	7.986670096	6.56E-05	0.000271583
PARG24666	-1.736873193	0.250271959	6.62E-05	0.000274064
PARG19672	-1.100769968	5.388723589	6.68E-05	0.000276563
PARG03433	-1.077801049	5.538557863	6.73E-05	0.000278549
PARG01606	-1.510711276	0.894188714	6.75E-05	0.000279274
PARG13946	-1.146990429	4.19597995	6.77E-05	0.000280125
PARG26736	-2.257909026	1.22193708	6.81E-05	0.000281228
PARG13692	-2.110988995	0.21107592	6.81E-05	0.000281228
PARG21181	2.08039494	1.062444716	6.81E-05	0.000281228
PARG04908	-1.074006297	6.898835134	6.81E-05	0.000281445
PARG13269	-1.128559155	4.302894683	6.84E-05	0.00028232
PARG00100	-1.121322153	4.164685409	6.85E-05	0.000282972
PARG23459	1.098674377	4.56565396	6.86E-05	0.000283004
PARG21822	-1.172561115	3.564039163	6.86E-05	0.000283108
PARG05856	-1.439133809	1.486153923	6.89E-05	0.000284115
PARG17237	-1.082650021	5.382477558	6.89E-05	0.000284251
PARG06952	1.083527727	5.015722909	6.90E-05	0.000284368
PARG10350	1.071972843	6.405801013	6.91E-05	0.000284718
PARG16484	-1.072664972	6.967355005	6.92E-05	0.000285035
PARG12678	1.196066471	2.762424682	6.92E-05	0.000285062
PARG07899	-1.082016939	5.609204248	6.95E-05	0.000286356
PARG08430	-1.320132067	2.438304472	7.00E-05	0.000288223
PARG24421	-1.301182762	2.705150502	7.00E-05	0.000288223
PARG22534	-1.090246133	4.890356661	7.02E-05	0.00028878
PARG17834	-1.063296614	8.0289989	7.07E-05	0.000290781

PARG28150	1.094188715	4.920919966	7.07E-05	0.000290977
PARG26318	1.077791287	5.089242418	7.09E-05	0.000291612
PARG20239	-1.211832259	2.946656611	7.10E-05	0.000291798
PARG18322	-1.810743846	0.478423383	7.11E-05	0.00029207
PARG03062	-1.106269031	4.354737682	7.11E-05	0.000292104
PARG02536	1.065439626	7.401593534	7.13E-05	0.000293003
PARG09434	-3.768521806	-0.33672938	7.16E-05	0.000293885
PARG04885	-3.766235778	-0.331727813	7.16E-05	0.000293885
PARG22667	-3.762530195	-0.074999376	7.16E-05	0.000293885
PARG14886	-1.086711681	4.056568702	7.19E-05	0.000295033
PARG13687	-1.073286953	5.63018098	7.19E-05	0.000295209
PARG02171	1.068681957	6.58409482	7.21E-05	0.000295751
PARG12664	-1.065751738	6.970981436	7.24E-05	0.000296711
PARG20156	1.061715469	7.966483506	7.30E-05	0.000299245
PARG00927	-2.727944287	1.651557613	7.32E-05	0.000299764
PARG30407	2.417805094	-0.250130947	7.32E-05	0.000299764
PARG03993	-1.076060859	5.564468995	7.32E-05	0.000299764
PARG16420	-1.155385234	3.075842572	7.35E-05	0.00030109
PARG12267	-1.081034968	5.352642915	7.38E-05	0.00030215
PARG03640	-1.125931299	4.501155708	7.41E-05	0.000303206
PARG08614	-5.395296236	0.715897557	7.41E-05	0.000303209
PARG19143	-5.391390233	1.202273532	7.41E-05	0.000303209
PARG24428	-5.390175141	-0.578560073	7.41E-05	0.000303209
PARG19356	-1.059553145	8.364715322	7.43E-05	0.000303698
PARG04070	1.096275901	4.584766121	7.50E-05	0.000306655
PARG14146	1.064103776	6.78440888	7.50E-05	0.000306655
PARG06597	1.091389004	4.339427335	7.51E-05	0.000306678
PARG02921	-1.230299039	2.655488517	7.53E-05	0.000307607
PARG25229	1.146054243	3.654693003	7.54E-05	0.000307812
PARG02299	-1.678871913	1.301484261	7.54E-05	0.000307834
PARG20952	-1.06465421	6.65395946	7.59E-05	0.000309739
PARG07550	-1.138757951	3.385189245	7.59E-05	0.000309745
PARG24173	-1.276108378	2.527887221	7.60E-05	0.000310188
PARG24227	1.072485158	5.614786352	7.63E-05	0.000311246
PARG23243	-1.061919521	7.160583285	7.63E-05	0.000311246
PARG22738	-1.350301148	0.924572133	7.64E-05	0.000311323
PARG22946	1.202121612	2.465848366	7.69E-05	0.000313463
PARG13145	1.072083478	5.615842509	7.71E-05	0.000313977
PARG26480	-1.14679068	3.509930038	7.72E-05	0.000314331
PARG25637	1.054919928	11.40392479	7.74E-05	0.000315363
PARG01256	1.092786062	4.637097929	7.75E-05	0.000315622
PARG08346	-2.17848834	0.650362248	7.79E-05	0.000316858
PARG26624	-2.177903675	0.033144792	7.79E-05	0.000316858
PARG30342	1.352304242	0.832667327	7.83E-05	0.000318439
PARG13084	1.150883762	3.444800223	7.83E-05	0.000318584
PARG12177	1.065656107	6.126440586	7.85E-05	0.00031908

PARG02841	-1.5686137	1.562713687	7.92E-05	0.00032201
PARG26708	-1.211333828	2.317417933	7.93E-05	0.00032241
PARG16616	-1.112541121	3.700500309	7.95E-05	0.000322807
PARG28300	-1.069796406	5.094046269	7.97E-05	0.000323546
PARG19296	1.060859166	6.806430107	7.97E-05	0.000323642
PARG08487	-1.066220584	6.203245999	7.97E-05	0.000323642
PARG06661	-1.054747943	8.521730883	7.99E-05	0.000324287
PARG30012	1.060067229	6.558843654	8.00E-05	0.00032476
PARG00800	-2.738283683	0.669079964	8.10E-05	0.000328492
PARG28199	1.06729544	5.929373479	8.11E-05	0.000328893
PARG21368	-2.299699632	0.05766539	8.12E-05	0.00032895
PARG15120	-2.17111728	0.88556436	8.12E-05	0.00032895
PARG15795	1.059305433	6.518851623	8.14E-05	0.000329822
PARG02620	1.079356653	5.052540967	8.19E-05	0.000331846
PARG20377	-1.37674203	1.635458242	8.20E-05	0.000331914
PARG12080	-1.067096473	5.815510191	8.22E-05	0.00033279
PARG08479	1.217743768	2.468516103	8.25E-05	0.000334008
PARG09437	-1.054410652	7.940041445	8.28E-05	0.000335144
PARG27594	-1.071393238	5.658640999	8.33E-05	0.000336875
PARG07415	-1.075916482	5.284068497	8.43E-05	0.000341065
PARG08608	-1.643827777	1.093749946	8.47E-05	0.000342688
PARG20063	1.172160105	3.263760283	8.48E-05	0.000342688
PARG09363	-1.804435992	0.186592623	8.49E-05	0.000343057
PARG19384	-1.191022993	2.590659667	8.51E-05	0.000343699
PARG12334	-1.059117493	5.972885871	8.51E-05	0.000343738
PARG15558	1.049871085	9.153732941	8.58E-05	0.000346491
PARG24436	1.0675501	5.123395655	8.58E-05	0.000346491
PARG00491	-3.296767617	1.205842962	8.60E-05	0.000347111
PARG17060	1.069623928	5.093323933	8.60E-05	0.000347221
PARG15495	-1.169022068	2.833998005	8.61E-05	0.000347421
PARG15413	1.471968544	1.004808328	8.65E-05	0.000348959
PARG07665	1.181939547	2.69663995	8.66E-05	0.00034928
PARG02700	1.079254054	4.863477351	8.67E-05	0.000349403
PARG08129	1.94879223	-0.177775754	8.76E-05	0.000352899
PARG08312	-1.079560001	4.703946007	8.76E-05	0.000353118
PARG08090	1.892943414	1.314742134	8.78E-05	0.000353833
PARG17595	1.053587751	6.768482337	8.81E-05	0.000354786
PARG29522	1.059442343	6.251276478	8.82E-05	0.000355165
PARG06055	1.077446703	4.900250489	8.86E-05	0.000356781
PARG23675	1.290407581	0.693918271	8.87E-05	0.00035706
PARG20101	-1.059320222	6.484129748	8.89E-05	0.000357691
PARG19091	-1.096276626	3.86374875	8.98E-05	0.000361272
PARG10210	1.055115749	6.04070632	9.04E-05	0.000363397
PARG16189	-1.049690036	7.018704894	9.14E-05	0.000367451
PARG15664	-1.14743927	3.21452101	9.14E-05	0.000367514
PARG07572	-1.149170613	2.81855851	9.23E-05	0.000370658

PARG01888	1.045839006	8.136155271	9.23E-05	0.00037074
PARG02310	1.086312336	4.156167945	9.24E-05	0.000371002
PARG13781	-1.104086483	3.699024231	9.29E-05	0.00037285
PARG27089	1.084340874	4.504567451	9.34E-05	0.000374767
PARG21808	-1.044830566	7.922755978	9.34E-05	0.000374951
PARG18873	-2.230858239	0.010484646	9.37E-05	0.000375778
PARG15909	-1.268612716	2.242704678	9.38E-05	0.000376155
PARG15996	1.165706451	3.008157737	9.40E-05	0.000376832
PARG27657	-2.509213917	1.479053333	9.43E-05	0.00037781
PARG11196	-2.507663257	2.050371818	9.43E-05	0.00037781
PARG02652	-1.054792649	5.851166995	9.44E-05	0.000378104
PARG17673	1.061975463	5.003786039	9.48E-05	0.000379856
PARG25399	-1.06487918	5.10566859	9.49E-05	0.000380118
PARG23064	1.331707655	1.12119985	9.50E-05	0.000380456
PARG23801	1.837683178	0.043788561	9.51E-05	0.000380537
PARG19860	1.048090572	6.917768963	9.52E-05	0.000380825
PARG00058	1.045854601	7.096240452	9.52E-05	0.000380825
PARG27838	1.055008254	5.840053404	9.53E-05	0.000381289
PARG06033	-1.058391274	5.397509709	9.56E-05	0.000382222
PARG27250	-1.050870933	5.826970275	9.62E-05	0.000384741
PARG18893	1.055178558	5.885183702	9.63E-05	0.00038481
PARG04216	2.241138896	0.269114517	9.69E-05	0.000387298
PARG00033	1.053123393	6.023001272	9.76E-05	0.000389707
PARG21701	1.041976077	7.975690265	9.81E-05	0.000391792
PARG13207	1.251346101	2.198132853	9.84E-05	0.000392731
PARG06714	-1.048574471	6.454915837	9.84E-05	0.000392833
PARG29407	1.043917067	7.172657321	9.88E-05	0.000394388
PARG23454	1.059796176	5.152183424	9.93E-05	0.000396188
PARG17697	1.068317851	4.660323827	9.95E-05	0.000396973
PARG30030	-1.06950493	4.914439813	0.000100998	0.000402711
PARG14490	-1.818971106	-0.181862549	0.000101222	0.000403499
PARG13496	-1.078474209	4.40480932	0.000101313	0.000403762
PARG18314	-1.534310076	1.336750043	0.000101715	0.000405261
PARG22515	-1.728160401	1.35190091	0.000102071	0.000406575
PARG03178	1.315329321	2.790522913	0.00010256	0.000408418
PARG16231	1.041031466	7.403417362	0.00010282	0.00040935
PARG12804	1.04165777	6.167062252	0.000103163	0.000410613
PARG08103	1.340314911	2.126137081	0.000104765	0.000416851
PARG26513	-1.112665028	3.310269423	0.000104784	0.000416851
PARG27943	1.231784997	2.554408081	0.0001051	0.000418002
PARG13827	1.041104346	7.193813618	0.000105255	0.000418514
PARG13204	-1.127720142	2.926962054	0.000105602	0.000419787
PARG05167	1.043598021	6.250333848	0.00010571	0.00042011
PARG07459	1.069838931	4.398427138	0.000105799	0.000420356
PARG23731	1.867658474	1.360500005	0.000106234	0.000421979
PARG28244	-1.318361388	2.40781015	0.000106391	0.000422495

PARG09361	-1.165092663	2.468644624	0.000106757	0.000423842
PARG10446	1.038365567	7.497992334	0.000106971	0.000424583
PARG27939	1.093775622	3.264813274	0.000107262	0.00042563
PARG19247	-1.068025367	4.451176326	0.000108163	0.000429088
PARG07020	-1.116973063	3.438107784	0.000108188	0.000429088
PARG09632	2.482535866	0.51867483	0.000109008	0.000432234
PARG04366	1.059499267	4.772167398	0.000109537	0.000434194
PARG11226	-1.089314064	3.708922443	0.000109558	0.000434194
PARG13470	1.198995313	2.334046101	0.000109609	0.000434288
PARG12299	-1.035317973	7.377286201	0.000110011	0.00043577
PARG26654	1.143495011	3.08963389	0.000110445	0.000437379
PARG04946	1.037324629	6.855533604	0.000110882	0.000438997
PARG06747	-1.732419618	0.175478671	0.00011115	0.000439801
PARG29552	1.642668097	-0.06846549	0.00011115	0.000439801
PARG19368	-1.052342489	5.193469198	0.000111169	0.000439801
PARG24755	1.032365877	9.184854881	0.000111393	0.000440575
PARG11344	1.081037247	3.94802339	0.000111765	0.000441938
PARG07143	-1.074628034	4.599232217	0.000112604	0.000445142
PARG27134	-2.141446486	0.897321363	0.000113118	0.000447062
PARG03759	-1.077580528	4.322015778	0.000113231	0.000447396
PARG22244	-1.216283467	1.749655499	0.000113948	0.000450116
PARG21713	1.0440398	6.165111969	0.00011497	0.000454037
PARG08586	1.747097226	0.647320161	0.000115283	0.000455045
PARG26226	1.711228169	-0.01761429	0.000115283	0.000455045
PARG29673	2.249498735	0.653977994	0.000115743	0.000456746
PARG12084	-1.031472696	7.838465488	0.000116434	0.000459358
PARG10000	-1.044040878	4.508410345	0.000116992	0.000461441
PARG20136	1.050032988	5.398632934	0.000117134	0.000461888
PARG15598	1.102954805	2.119055431	0.000117239	0.000462183
PARG01687	-1.0353397	6.97476995	0.000117338	0.000462458
PARG19661	1.030334331	7.809352627	0.00011835	0.000466328
PARG12709	-1.031970689	7.583704217	0.000118669	0.000467469
PARG08538	1.239597265	2.265435983	0.000118954	0.00046837
PARG06689	1.03429845	6.699297365	0.000118957	0.00046837
PARG02006	-1.425867716	1.522480978	0.000119557	0.000470611
PARG06307	-1.048781944	5.007213396	0.000119684	0.000470996
PARG14545	-1.589479894	-0.046095487	0.000120288	0.000473255
PARG15362	-3.696470666	-0.444859937	0.000121093	0.0004763
PARG15216	-1.312738404	1.775394938	0.000121728	0.000478678
PARG14071	1.060306952	3.701046554	0.000121776	0.000478749
PARG00235	1.033830032	6.591544206	0.000122065	0.000479766
PARG23052	-1.130606777	2.65499186	0.000122241	0.000480334
PARG05819	1.056245181	4.805556634	0.000122781	0.000482335
PARG08335	-1.316909632	1.713437453	0.000122972	0.000482964
PARG03480	-1.205159556	2.513475646	0.000123276	0.00048404
PARG28126	1.067850933	4.89742774	0.000123578	0.000485102

PARG23238	-1.032392063	7.351233441	0.000123773	0.000485746
PARG26853	1.215900512	2.088648647	0.000123918	0.000486195
PARG02351	1.468005533	0.987466051	0.000125044	0.000490489
PARG27731	1.030005611	6.838419997	0.000125202	0.000490915
PARG21881	1.042446538	5.513258257	0.000125215	0.000490915
PARG08124	1.040541444	5.549500821	0.000125477	0.000491818
PARG16081	1.023724579	9.391709918	0.000125868	0.000493228
PARG02185	1.090205887	3.543551946	0.000126179	0.000494324
PARG28515	1.099494254	3.59249703	0.000126642	0.000496015
PARG18427	1.17352338	2.968606934	0.000127118	0.000497754
PARG14474	-1.15738301	2.582915201	0.000127549	0.000499193
PARG07812	-1.146793795	3.675649061	0.000127549	0.000499193
PARG08920	1.02383704	8.261612562	0.000127694	0.000499637
PARG15404	1.088902363	3.70737827	0.000127761	0.000499772
PARG05842	-1.023052433	8.517056758	0.000128709	0.000503358
PARG25061	1.995157837	0.671356119	0.000128921	0.000503937
PARG03389	1.99292105	1.146938955	0.000128921	0.000503937
PARG16236	-1.045348313	4.735238835	0.000129055	0.000504332
PARG17416	-1.091454898	3.494385271	0.000129184	0.000504713
PARG20221	-1.0310128	6.337549696	0.000129658	0.000506436
PARG03392	1.072239492	4.059131518	0.000130087	0.000507988
PARG20058	-1.204088444	1.896570514	0.000131499	0.000513373
PARG00953	-1.027185584	6.802612623	0.000131693	0.000514
PARG26306	-1.060470264	4.686195788	0.000132131	0.000515583
PARG16148	1.067100547	3.904104732	0.000132365	0.000516369
PARG18366	-1.144285742	3.014032993	0.00013246	0.000516611
PARG21664	1.060191246	4.106201284	0.000132703	0.00051743
PARG17206	5.433126435	0.028435136	0.000133436	0.000519773
PARG00964	-5.310573705	0.799517575	0.000133436	0.000519773
PARG08510	-5.310052999	-0.111801236	0.000133436	0.000519773
PARG04521	-5.308108115	-0.362285144	0.000133436	0.000519773
PARG16125	-1.188709141	2.144678813	0.000133623	0.000520372
PARG07586	1.03842684	5.550787664	0.000133923	0.000521409
PARG19979	2.747576393	0.806278649	0.000134807	0.00052472
PARG16848	1.033084926	5.793187319	0.000135026	0.000525445
PARG14258	-1.03011679	6.146447401	0.000135232	0.000526114
PARG06708	1.128034877	2.783588836	0.000135787	0.000528142
PARG24670	-1.328691934	0.651449076	0.000136123	0.000529057
PARG15155	-1.321241077	1.637466241	0.000136123	0.000529057
PARG04180	1.299489428	0.406578862	0.000136123	0.000529057
PARG17969	-3.87343391	3.018838557	0.000137243	0.000533279
PARG13144	1.062101038	4.665366064	0.00013734	0.000533522
PARG24709	-1.022522768	7.187895035	0.000137474	0.000533912
PARG24553	-1.094950023	3.25403366	0.000137693	0.00053463
PARG24102	1.028496033	6.380850286	0.000137732	0.000534649
PARG13111	1.049162505	4.540201608	0.00013864	0.000538039

PARG20208	-1.087294026	4.597816369	0.000139168	0.000539954
PARG15478	-1.83511814	-0.144754279	0.000139469	0.000540989
PARG10291	-1.081244155	3.494085427	0.000139803	0.00054215
PARG19226	1.042043853	4.924002156	0.000140199	0.000543554
PARG05682	1.8554825	-0.129705869	0.000140363	0.000544055
PARG14180	-1.026418923	5.660416705	0.000140447	0.000544156
PARG24710	-1.050929749	4.524920359	0.000140459	0.000544156
PARG26143	1.131662134	3.701314729	0.000140579	0.00054449
PARG13480	1.023185776	6.64670486	0.000140744	0.000544993
PARG25134	1.072928195	3.622729968	0.000142589	0.000552
PARG13552	-1.42486573	1.391046594	0.000142732	0.000552416
PARG06790	-1.035896218	5.299862982	0.000143446	0.000555045
PARG11056	1.018329933	7.186768366	0.000143654	0.000555711
PARG03012	1.029942027	5.25746001	0.000144152	0.000557388
PARG11090	-2.123031511	-0.169746403	0.000144158	0.000557388
PARG10116	-1.117025852	2.7374115	0.000145775	0.000563499
PARG19488	-1.029919848	5.530373611	0.000146031	0.000564352
PARG24959	1.018329659	6.977736257	0.000146558	0.000566234
PARG27940	-1.102416338	3.107400401	0.00014659	0.000566234
PARG12302	-1.706798672	0.782613394	0.000146806	0.000566929
PARG00238	-1.012722207	10.36384085	0.000147001	0.000567541
PARG11453	1.016601115	7.378467543	0.000148229	0.00057214
PARG12747	-1.026537973	6.315960113	0.000148435	0.000572795
PARG29471	-1.016518813	7.102516634	0.000148774	0.000573962
PARG24001	1.085989443	3.001883881	0.000150025	0.000578647
PARG27310	1.019338254	6.613680948	0.000150583	0.000580657
PARG27163	1.01907221	6.567016053	0.000150783	0.000581287
PARG19050	1.013259663	7.828676789	0.000152331	0.000587109
PARG09734	-1.31066438	2.072616739	0.000152391	0.000587196
PARG12208	1.013977192	7.555934393	0.000152591	0.000587824
PARG08344	1.01303536	7.420463232	0.000152891	0.000588833
PARG19715	-1.573354993	0.6063279	0.000152932	0.000588845
PARG17974	1.091455357	3.2765559	0.000153834	0.000592175
PARG09236	1.028438785	5.325040064	0.0001553	0.00059767
PARG03791	1.023401826	5.390461381	0.000156021	0.000600298
PARG20466	-1.173419014	2.292456314	0.000156203	0.000600852
PARG02954	-1.088513732	4.040026067	0.000156378	0.000601376
PARG02501	1.12364911	2.585669531	0.000156701	0.000602473
PARG13918	1.014926833	6.721308326	0.000156776	0.000602614
PARG26929	1.531500254	0.494222535	0.000157277	0.000604389
PARG02923	-2.108602558	1.031185789	0.000157671	0.000605606
PARG07240	-2.085110323	1.412932634	0.000157671	0.000605606
PARG10873	-1.052401041	4.854996673	0.000158251	0.000607687
PARG17743	1.031848461	5.335915489	0.000158337	0.000607812
PARG20996	-1.007347186	10.702974	0.000158361	0.000607812
PARG20016	-1.038565015	4.453364673	0.000160771	0.000616912

PARG16918	1.099717939	2.626310327	0.000162443	0.000623174
PARG03239	-1.019061412	5.824838891	0.000163509	0.000627111
PARG26412	1.007917431	8.052951359	0.000164063	0.000629083
PARG24487	1.024562729	4.969365347	0.000164999	0.000632518
PARG02335	-1.532271666	0.214193011	0.000165277	0.000633429
PARG28314	-1.167054691	2.80101875	0.00016595	0.000635852
PARG07164	-1.614981468	1.041880436	0.000166489	0.000637762
PARG19687	1.016206857	5.819586562	0.000166948	0.000639362
PARG05701	1.017328305	5.700904176	0.000167084	0.000639727
PARG03044	-1.00942572	6.780230818	0.000167354	0.000640607
PARG16195	-1.004170495	9.507664325	0.000169067	0.000647003
PARG02717	1.005300222	8.184437252	0.000169466	0.000648375
PARG03591	-1.024832927	5.372179302	0.000169583	0.000648663
PARG07787	1.013299585	6.352319769	0.000170975	0.000653829
PARG21456	-1.003079233	8.864272828	0.000171475	0.000655579
PARG24203	-1.003670762	8.600091814	0.000171822	0.000656647
PARG04404	-1.043752499	3.957880613	0.000171838	0.000656647
PARG01972	-1.321734093	2.967130758	0.000172895	0.000660361
PARG30021	-1.292823142	1.598204074	0.000172895	0.000660361
PARG24956	-1.136957656	2.726765239	0.000172936	0.000660361
PARG13047	-1.074618637	2.885349396	0.000173205	0.000661227
PARG15435	-1.029580779	4.741926426	0.000173607	0.0006626
PARG30323	-1.066850188	3.559834342	0.000173787	0.000663127
PARG29015	-1.010298485	6.395349589	0.000174247	0.000664723
PARG08686	-1.386816163	1.346859843	0.00017498	0.000667357
PARG21554	-1.04092829	4.286811131	0.000175477	0.000669089
PARG18919	1.004216938	7.858298418	0.00017556	0.000669241
PARG10120	1.480518717	0.954726203	0.000175704	0.000669628
PARG00758	1.008478389	5.914606166	0.000175802	0.00066984
PARG17142	1.024051827	4.981923645	0.000175885	0.000669994
PARG13863	-1.110670228	2.792407539	0.000176039	0.000670419
PARG08626	-1.399666264	0.698692171	0.000178282	0.000678795
PARG21604	1.041317783	4.033581652	0.000178757	0.000680437
PARG11111	-1.030256442	4.787406092	0.000180664	0.000687532
PARG15480	-1.010068712	5.873874034	0.000181037	0.000688782
PARG16495	-1.25451212	1.37705424	0.000181405	0.000690016
PARG23927	1.031522042	4.797316227	0.000181504	0.000690224
PARG14872	-1.473147244	0.834626648	0.000181719	0.000690874
PARG29256	1.033250575	4.618360495	0.000181828	0.000691123
PARG23458	1.045391572	4.124021774	0.000182968	0.000695289
PARG03699	-1.056368113	3.054872378	0.000183743	0.000697972
PARG27354	1.006363802	6.216278572	0.000183785	0.000697972
PARG14140	1.001900219	7.435620779	0.000183808	0.000697972
PARG26160	1.001883539	7.656394604	0.000184511	0.000700472
PARG05709	-1.107101367	3.005926835	0.00018475	0.000701042
PARG05718	-1.107101367	3.005926835	0.00018475	0.000701042

PARG16140	1.316995546	2.557633171	0.000185222	0.000702494
PARG00370	-1.045831789	4.227423028	0.000186339	0.000706559
PARG11163	1.045844977	4.072349852	0.000187348	0.000710213
PARG08017	1.016286957	4.828724625	0.000187945	0.000712305
PARG29257	1.031707596	4.454859744	0.000188042	0.000712499
PARG02238	-2.829010069	1.226462066	0.000188491	0.000713683
PARG06585	-2.626894879	1.647316956	0.000188491	0.000713683
PARG10632	2.502461138	-0.427511023	0.000188491	0.000713683
PARG02637	-1.313653866	1.525137862	0.000188624	0.000714014
PARG06388	1.0613934	3.351906149	0.000190274	0.000720087
PARG12929	1.02459047	4.629038695	0.000191774	0.000725588
PARG19281	-1.011152255	5.713763184	0.000192937	0.000729815
PARG21424	-1.09191961	3.165863614	0.000193276	0.000730921
PARG29956	-1.015759812	6.196001655	0.000193405	0.000731232
PARG10751	-1.108977206	3.45223366	0.000193966	0.000733175
PARG11267	-1.029615171	3.908623562	0.00019403	0.000733243
PARG00455	-1.299353207	1.415362499	0.000194822	0.000736058
PARG19932	1.030681281	4.072710846	0.000195638	0.000738962
PARG19443	-1.022696086	4.95665258	0.000196378	0.000741577
PARG11037	-1.01135162	5.546641833	0.000196531	0.000741979
PARG19457	-1.025507103	4.583827929	0.000197527	0.000745062
PARG21665	-1.740934449	1.529834236	0.000197538	0.000745062
PARG09893	-1.022093466	4.685104613	0.000198837	0.000749424
PARG17353	-1.041345906	4.008300369	0.000199905	0.000753266
PARG07290	1.009705004	5.477847879	0.000202124	0.000761444
PARG28188	-1.238720504	1.435393515	0.000202545	0.000762804
PARG20813	-1.088113394	2.76144721	0.000202582	0.000762804
PARG14197	1.22864544	0.659431697	0.000202875	0.000763723
PARG06193	-1.537588978	0.197756097	0.000204077	0.000767696
PARG10344	-1.522470176	0.861358955	0.000204077	0.000767696
PARG12366	1.486387928	-0.303092957	0.000204077	0.000767696
PARG15214	1.105613865	2.84969755	0.000204259	0.000768197
PARG10506	1.049380953	3.769418994	0.000204815	0.000770105
PARG08433	-3.630341701	-0.171956628	0.000205502	0.000772316
PARG11835	-3.628009019	-0.597018842	0.000205502	0.000772316
PARG12710	1.008837757	5.734228816	0.000208078	0.000781811
PARG15673	1.025493554	4.8166057	0.000208742	0.000784117
PARG27228	-1.768239082	1.026677403	0.000208821	0.000784228
PARG29907	1.017808657	4.797977853	0.000208976	0.000784452
PARG01905	-1.011017785	4.998019187	0.000208981	0.000784452
PARG07058	1.042996304	4.061113873	0.000209367	0.000785713
PARG27652	1.005882327	6.151875887	0.00020949	0.000785802
PARG05738	-2.684655549	-0.62597787	0.000211598	0.000793326
PARG04150	1.003293573	5.636259283	0.000211803	0.000793907
PARG08639	-1.029355463	3.91609785	0.000212137	0.000794969
PARG27038	-1.00104964	6.335175614	0.000212347	0.00079544

PARG10876	1.0189782	4.920981004	0.000212399	0.00079544
PARG19705	1.090834721	3.178115656	0.000212415	0.00079544
PARG12791	1.001754306	5.592072159	0.000214482	0.000802801
PARG15566	1.001088403	5.893829213	0.000215744	0.000806947
PARG19072	-1.0238894	4.641988651	0.000218604	0.000817253
PARG16073	-1.507343288	1.018891242	0.000219147	0.000818891
PARG18546	-2.086164473	1.015939317	0.000219755	0.000820875
PARG09818	1.004533053	4.289313694	0.000219782	0.000820875
PARG07355	-1.128230271	3.29120111	0.000220798	0.000824473
PARG17474	-1.261911712	1.968319803	0.000222396	0.000826882
PARG21081	-1.092422977	3.451104064	0.000222568	0.000826882
PARG04595	-3.853135164	1.421915038	0.000222945	0.000826882
PARG08304	-3.018825649	-0.508231532	0.000222945	0.000826882
PARG02957	-1.068678452	3.146505527	0.000223075	0.000826882
PARG28919	-1.067817503	3.319093374	0.000223708	0.000826882
PARG28923	-1.067817503	3.319093374	0.000223708	0.000826882
PARG28928	-1.067817503	3.319093374	0.000223708	0.000826882
PARG28951	-1.067817503	3.319093374	0.000223708	0.000826882
PARG28956	-1.067817503	3.319093374	0.000223708	0.000826882
PARG28965	-1.067817503	3.319093374	0.000223708	0.000826882
PARG28970	-1.067817503	3.319093374	0.000223708	0.000826882
PARG28976	-1.067817503	3.319093374	0.000223708	0.000826882
PARG28981	-1.067817503	3.319093374	0.000223708	0.000826882
PARG28985	-1.067817503	3.319093374	0.000223708	0.000826882
PARG28990	-1.067817503	3.319093374	0.000223708	0.000826882
PARG29000	-1.067817503	3.319093374	0.000223708	0.000826882
PARG29005	-1.067817503	3.319093374	0.000223708	0.000826882
PARG29010	-1.067817503	3.319093374	0.000223708	0.000826882
PARG29014	-1.067817503	3.319093374	0.000223708	0.000826882
PARG29032	-1.067817503	3.319093374	0.000223708	0.000826882
PARG29036	-1.067817503	3.319093374	0.000223708	0.000826882
PARG29062	-1.067817503	3.319093374	0.000223708	0.000826882
PARG29068	-1.067817503	3.319093374	0.000223708	0.000826882
PARG29073	-1.067817503	3.319093374	0.000223708	0.000826882
PARG29084	-1.067817503	3.319093374	0.000223708	0.000826882
PARG29090	-1.067817503	3.319093374	0.000223708	0.000826882
PARG29096	-1.067817503	3.319093374	0.000223708	0.000826882
PARG29116	-1.067817503	3.319093374	0.000223708	0.000826882
PARG29125	-1.067817503	3.319093374	0.000223708	0.000826882
PARG30173	-1.067817503	3.319093374	0.000223708	0.000826882
PARG30179	-1.067817503	3.319093374	0.000223708	0.000826882
PARG30184	-1.067817503	3.319093374	0.000223708	0.000826882
PARG30189	-1.067817503	3.319093374	0.000223708	0.000826882
PARG30228	-1.067817503	3.319093374	0.000223708	0.000826882
PARG30233	-1.067817503	3.319093374	0.000223708	0.000826882
PARG30238	-1.067817503	3.319093374	0.000223708	0.000826882

PARG30243	-1.067817503	3.319093374	0.000223708	0.000826882
PARG30265	-1.067817503	3.319093374	0.000223708	0.000826882
PARG30270	-1.067817503	3.319093374	0.000223708	0.000826882
PARG30276	-1.067817503	3.319093374	0.000223708	0.000826882
PARG30282	-1.067817503	3.319093374	0.000223708	0.000826882
PARG16311	1.212624159	2.357731834	0.000224045	0.000827933
PARG23983	1.022231165	4.606019981	0.000224419	0.000829121
PARG23885	-1.18294083	1.789071315	0.000224787	0.000830285
PARG25565	-1.197926907	1.985526917	0.000224969	0.000830566
PARG24048	-1.187508722	2.92657663	0.000224969	0.000830566
PARG13004	1.019492888	4.541959951	0.000225128	0.000830958
PARG09942	1.014763175	4.385484093	0.000229257	0.000845403
PARG15832	-1.107033458	2.271207578	0.000229561	0.000846325
PARG21150	-1.01005319	5.604739222	0.000229929	0.000847483
PARG13258	1.343689401	1.159702091	0.0002314	0.000852505
PARG18448	-1.025772655	4.158469018	0.000232264	0.000855287
PARG19196	1.121065599	2.569179019	0.00023332	0.00085857
PARG02600	-1.015377145	4.485114893	0.000235342	0.000865403
PARG22366	-1.391400612	0.829109381	0.000235759	0.000866529
PARG27724	-1.342196526	0.9297173	0.000235759	0.000866529
PARG06352	-1.009818578	4.710926132	0.000236972	0.000870485
PARG01903	-1.390080414	1.414375017	0.000237002	0.000870485
PARG20555	-1.00155922	4.861482259	0.00023722	0.000871084
PARG01760	1.033373258	3.82552226	0.00024099	0.000884511
PARG11474	-5.267011158	-0.832133399	0.000241628	0.000886648
PARG21488	-2.58937673	0.478922029	0.000242371	0.000889167
PARG18410	-1.023407133	4.243584266	0.000243098	0.000891626
PARG16347	1.003688158	5.191457824	0.000243734	0.000893747
PARG06699	-1.86206613	0.291850356	0.000244881	0.000897743
PARG01782	-1.026162108	4.302544231	0.000246569	0.000903723
PARG05448	1.081340168	3.255682044	0.000246699	0.000903985
PARG11983	-1.201936449	2.224719139	0.000246804	0.000904161
PARG26490	1.246990996	1.8685044	0.00024789	0.000907926
PARG08684	-1.248257178	2.416975975	0.000248304	0.000909021
PARG08546	1.217660731	1.923184846	0.000248367	0.000909037
PARG03072	-1.018681032	4.325585424	0.000249146	0.000911251
PARG04912	-1.007230116	4.640779582	0.000250169	0.000914779
PARG02756	1.013642086	4.658057656	0.000254291	0.000928989
PARG11098	1.003380489	4.734010473	0.000255262	0.000932319
PARG12160	-1.144121673	2.292527725	0.000257639	0.000940344
PARG09705	1.392695607	1.393887826	0.000257973	0.000941251
PARG21588	1.030850356	2.972935524	0.000258211	0.000941774
PARG20684	-1.001555897	4.717577764	0.000258838	0.000943843
PARG12222	1.161210795	1.942730851	0.000265988	0.000968564
PARG03297	1.107561854	2.537346976	0.000266094	0.000968724
PARG23400	1.167182609	2.231178739	0.000266418	0.000969679

PARG10913	-1.115383782	3.094588606	0.000266567	0.000969997
PARG26456	-1.89457475	0.729156791	0.000266726	0.000970351
PARG04642	-1.411919969	0.396844339	0.000267656	0.000973507
PARG02960	-1.182294816	1.865079972	0.00026984	0.00098077
PARG11385	-1.074329432	3.46619774	0.000270154	0.000981684
PARG05911	-1.743388568	1.118271195	0.0002713	0.000985393
PARG16630	-1.050211221	3.316452328	0.000272865	0.000990618
PARG23969	-1.20956566	2.316396417	0.000276018	0.001001051
PARG14392	-1.024674093	3.472990206	0.000276057	0.001001051
PARG16322	-1.006376742	4.270090446	0.000276305	0.001001718
PARG12580	1.263328558	1.854205904	0.000278699	0.00100993
PARG14402	1.022905217	3.638086268	0.00028102	0.00101787
PARG16869	-1.116266432	2.977673023	0.000282835	0.001024209
PARG05725	-1.072271094	2.830717896	0.000285725	0.00103372
PARG29953	-2.78740915	-0.026567559	0.000287883	0.001040807
PARG24409	-2.787148798	-0.403437354	0.000287883	0.001040807
PARG00730	-2.383180476	1.30825366	0.0002942	0.001061202
PARG14998	-2.342983099	2.161288105	0.0002942	0.001061202
PARG10388	-1.121406566	2.206324212	0.000294778	0.001062553
PARG04293	1.048002038	2.855977171	0.000295222	0.001063911
PARG04653	-1.063636137	2.810539031	0.000296795	0.001068107
PARG04178	1.008474921	3.071390353	0.000297482	0.001070089
PARG27071	-1.20017221	2.077924292	0.000298251	0.001072611
PARG15325	-1.435072642	0.954106802	0.000300384	0.001074525
PARG28918	-1.195716872	1.943648723	0.000301795	0.001074525
PARG28922	-1.195716872	1.943648723	0.000301795	0.001074525
PARG28927	-1.195716872	1.943648723	0.000301795	0.001074525
PARG28932	-1.195716872	1.943648723	0.000301795	0.001074525
PARG28952	-1.195716872	1.943648723	0.000301795	0.001074525
PARG28957	-1.195716872	1.943648723	0.000301795	0.001074525
PARG28964	-1.195716872	1.943648723	0.000301795	0.001074525
PARG28968	-1.195716872	1.943648723	0.000301795	0.001074525
PARG28969	-1.195716872	1.943648723	0.000301795	0.001074525
PARG28975	-1.195716872	1.943648723	0.000301795	0.001074525
PARG28980	-1.195716872	1.943648723	0.000301795	0.001074525
PARG28984	-1.195716872	1.943648723	0.000301795	0.001074525
PARG28989	-1.195716872	1.943648723	0.000301795	0.001074525
PARG28995	-1.195716872	1.943648723	0.000301795	0.001074525
PARG29001	-1.195716872	1.943648723	0.000301795	0.001074525
PARG29006	-1.195716872	1.943648723	0.000301795	0.001074525
PARG29011	-1.195716872	1.943648723	0.000301795	0.001074525
PARG29028	-1.195716872	1.943648723	0.000301795	0.001074525
PARG29033	-1.195716872	1.943648723	0.000301795	0.001074525
PARG29037	-1.195716872	1.943648723	0.000301795	0.001074525
PARG29056	-1.195716872	1.943648723	0.000301795	0.001074525
PARG29061	-1.195716872	1.943648723	0.000301795	0.001074525

PARG29067	-1.195716872	1.943648723	0.000301795	0.001074525
PARG29072	-1.195716872	1.943648723	0.000301795	0.001074525
PARG29083	-1.195716872	1.943648723	0.000301795	0.001074525
PARG29089	-1.195716872	1.943648723	0.000301795	0.001074525
PARG29095	-1.195716872	1.943648723	0.000301795	0.001074525
PARG29111	-1.195716872	1.943648723	0.000301795	0.001074525
PARG29115	-1.195716872	1.943648723	0.000301795	0.001074525
PARG29124	-1.195716872	1.943648723	0.000301795	0.001074525
PARG30174	-1.195716872	1.943648723	0.000301795	0.001074525
PARG30180	-1.195716872	1.943648723	0.000301795	0.001074525
PARG30185	-1.195716872	1.943648723	0.000301795	0.001074525
PARG30190	-1.195716872	1.943648723	0.000301795	0.001074525
PARG30229	-1.195716872	1.943648723	0.000301795	0.001074525
PARG30234	-1.195716872	1.943648723	0.000301795	0.001074525
PARG30239	-1.195716872	1.943648723	0.000301795	0.001074525
PARG30244	-1.195716872	1.943648723	0.000301795	0.001074525
PARG30264	-1.195716872	1.943648723	0.000301795	0.001074525
PARG30269	-1.195716872	1.943648723	0.000301795	0.001074525
PARG30275	-1.195716872	1.943648723	0.000301795	0.001074525
PARG30281	-1.195716872	1.943648723	0.000301795	0.001074525
PARG17391	1.429378285	-0.353269919	0.000302943	0.001078123
PARG27138	-1.000087184	5.094672865	0.000303278	0.001079072
PARG08110	1.041183731	3.517960101	0.0003041	0.001081751
PARG28475	1.956691559	0.023033957	0.000306231	0.001088589
PARG05878	-1.083101765	2.887194433	0.000308832	0.001097588
PARG30402	1.387614535	0.147382014	0.00030906	0.001097901
PARG08515	1.381223167	2.000972854	0.00030906	0.001097901
PARG06222	-1.112903469	3.084890411	0.000310003	0.001101001
PARG04392	1.105191903	1.528123828	0.000310865	0.001103563
PARG00823	1.618007209	0.305333634	0.000311218	0.001104568
PARG20877	-1.403528833	1.277546272	0.000314282	0.001114687
PARG08031	-1.361924116	1.217671179	0.000315916	0.001119468
PARG24754	-1.289878721	1.026868485	0.000316567	0.001121522
PARG23104	-1.293843206	1.220430561	0.000317512	0.001124616
PARG00477	-1.054027363	3.181636186	0.000318287	0.001127108
PARG06799	1.552068484	-0.015491509	0.000318889	0.001128729
PARG12854	-1.040016426	3.379046886	0.00031903	0.001128975
PARG20948	1.069401687	2.95815083	0.000319417	0.001129582
PARG19680	-1.020957476	3.781161876	0.000322546	0.00113962
PARG27649	-1.02155678	3.157926484	0.000327714	0.001156316
PARG11929	2.542346915	2.136964314	0.000332489	0.001171585
PARG03465	-1.068670791	2.107919958	0.00033335	0.001174355
PARG19987	1.182726648	1.791389487	0.000334069	0.001176624
PARG07543	-1.021701683	3.761209813	0.000335564	0.001180861
PARG09452	-1.01247887	4.727342759	0.000335564	0.001180861
PARG25204	-1.03000539	3.618573399	0.000335573	0.001180861

PARG19311	-1.095420275	2.841491321	0.000344769	0.001211051
PARG14234	1.013588045	3.066980098	0.000347743	0.001220846
PARG09623	-3.485795192	0.641007178	0.000349856	0.001226629
PARG09456	-3.482883628	-0.413032365	0.000349856	0.001226629
PARG28429	-1.234956705	1.673768065	0.000349907	0.001226629
PARG21317	-1.432137841	0.919585846	0.000353693	0.00123907
PARG07334	-1.475227473	0.18621717	0.000356589	0.001248658
PARG18742	1.172060094	2.153319309	0.000359794	0.001259601
PARG23373	-2.938628124	-0.597488584	0.000362814	0.001269325
PARG21307	-1.2890544	1.603832939	0.000365123	0.001276264
PARG05780	1.043791548	3.35407958	0.000368135	0.001286141
PARG28618	-1.327263148	1.696386644	0.000368195	0.001286141
PARG29662	1.309255077	1.614942747	0.000368195	0.001286141
PARG17647	-1.397289552	-0.043680152	0.000368281	0.001286157
PARG17601	1.301261542	1.327473234	0.000368569	0.001286876
PARG09183	-1.373237012	0.677476979	0.000368695	0.001287031
PARG14425	-1.031911745	3.047043659	0.000369948	0.001291118
PARG15803	-1.173857125	2.718165454	0.000372058	0.001297614
PARG07375	-1.63618198	0.995770603	0.000375412	0.001307569
PARG27147	-1.006411421	3.776117436	0.00038732	0.001347252
PARG29183	1.061285782	2.613121202	0.000388868	0.001352336
PARG25371	1.026915067	3.569042629	0.00039532	0.00137295
PARG12973	1.107810768	1.951851088	0.000408768	0.001418399
PARG19321	-1.030794864	3.736181415	0.000411389	0.001427178
PARG13912	-1.125891058	3.589931347	0.000412794	0.001431422
PARG18290	1.132910181	2.072460842	0.000412964	0.001431695
PARG27230	1.00855987	3.26842128	0.000419587	0.001452409
PARG06561	1.294184855	1.308291027	0.000425537	0.001471009
PARG20977	-2.179674959	0.512489231	0.000426602	0.001473241
PARG15643	-2.175063368	0.233511397	0.000426602	0.001473241
PARG12378	2.007669758	1.566649044	0.000426602	0.001473241
PARG20610	-1.807619872	0.048895973	0.000426637	0.001473241
PARG03834	-1.326489507	1.923861988	0.000426998	0.001474163
PARG11804	-2.334272576	0.420444335	0.000430007	0.001483899
PARG20618	-2.332886923	-0.282651979	0.000430007	0.001483899
PARG29976	-1.024351221	3.107084152	0.000430864	0.001486527
PARG03544	-2.732856213	1.138848689	0.000439894	0.001515833
PARG29248	-1.202230356	2.395422464	0.000439996	0.001515833
PARG17358	5.206281208	-0.848169161	0.000440227	0.001515833
PARG09035	-5.149899309	-0.582243797	0.000440227	0.001515833
PARG23946	-5.119800036	0.07587826	0.000440227	0.001515833
PARG27461	-5.118269657	-0.145616772	0.000440227	0.001515833
PARG00497	-5.11501212	-0.811248588	0.000440227	0.001515833
PARG08854	-1.101488783	2.440575316	0.000446779	0.001537044
PARG14940	1.213943431	2.647654739	0.000450451	0.001549
PARG14838	-1.025647205	2.967294755	0.000451569	0.001552503

PARG13484	-1.070284975	2.722791282	0.000455133	0.001563655
PARG19745	1.083162302	2.146647403	0.000458493	0.001572863
PARG08403	-1.027357809	2.554413208	0.000462781	0.001586533
PARG10425	1.233171154	0.897920471	0.000464112	0.001590401
PARG08434	-1.016102773	2.735144522	0.000466838	0.001599377
PARG19678	-1.004275229	2.771592717	0.000466935	0.001599377
PARG25509	1.576691345	0.229768236	0.000469763	0.001607429
PARG12069	-1.133209758	2.018365829	0.000470187	0.001608411
PARG22559	-1.010655828	2.968739196	0.000472359	0.001615488
PARG21231	-1.037295614	3.639801532	0.000482187	0.00164623
PARG14178	1.003667522	2.848205436	0.000485263	0.001655654
PARG12164	1.539523583	0.282543183	0.000489443	0.001669553
PARG24610	-1.008698579	3.097665373	0.000492668	0.001678989
PARG18312	-1.901452395	0.562743734	0.000492851	0.001678989
PARG10249	1.884683021	-0.305355954	0.000492851	0.001678989
PARG03436	-1.5779956	0.504719696	0.000497083	0.00169267
PARG10347	-1.601763089	-0.055313937	0.000503459	0.001712523
PARG20024	-1.3408247	1.156356807	0.000512592	0.001742459
PARG08939	1.213303235	1.081800207	0.00051528	0.001750457
PARG24243	-1.467690755	1.156763524	0.000516297	0.001753532
PARG02297	-1.008559668	3.743271659	0.000520534	0.001767159
PARG18962	-1.06291616	3.32040785	0.000526304	0.001784433
PARG10603	-1.082868678	2.774365658	0.000529917	0.001794742
PARG22994	1.72589914	0.124390609	0.000542941	0.001836083
PARG01155	1.21959557	1.254842624	0.000543481	0.001837511
PARG13823	-1.002323985	3.075471887	0.000544907	0.001841538
PARG19371	-1.107248629	2.226811019	0.000556605	0.001879051
PARG01029	1.70632231	0.131177041	0.000562724	0.001899299
PARG23994	-1.207723736	1.650554175	0.000567216	0.001911996
PARG08475	1.043607777	3.356677835	0.000576149	0.001940024
PARG13845	-1.106110039	2.169011994	0.000578542	0.001946411
PARG01394	-1.726133746	-0.015497538	0.000579448	0.001948625
PARG02753	1.689974142	2.538899807	0.000579448	0.001948625
PARG26804	-3.696969266	-0.297029575	0.00059137	0.00198574
PARG18581	1.110766144	2.781602146	0.000592844	0.001990264
PARG00395	-1.088937569	2.20525518	0.000594736	0.00199576
PARG29963	-4.153581097	-0.482720685	0.000597412	0.002002599
PARG00542	-4.124262716	0.697389236	0.000597412	0.002002599
PARG09486	-3.467183735	-0.181145563	0.000597412	0.002002599
PARG13722	-1.336371315	0.875639029	0.000608614	0.00203754
PARG21950	-1.146401314	2.686778794	0.000610035	0.002041426
PARG10843	-1.017404453	2.55962988	0.000612019	0.002046756
PARG23767	1.165724126	1.635213012	0.000613798	0.00205227
PARG30033	-1.083835258	2.952789568	0.000623327	0.002081911
PARG07970	-1.363476284	0.726441134	0.000623787	0.002083005
PARG22325	-1.003797341	2.918997103	0.000625696	0.002088493

PARG30037	-1.399751162	-0.09829461	0.000627055	0.002092138
PARG14980	1.57677587	0.172591241	0.000627766	0.002093176
PARG10650	-2.294588947	-1.238686603	0.000628378	0.002094325
PARG12335	-2.286234318	-0.030411172	0.000628378	0.002094325
PARG18672	-1.101358874	1.848304391	0.000635615	0.002116202
PARG22874	-1.444678089	0.783059784	0.000636638	0.002119156
PARG18669	-1.172178394	1.647623248	0.000652203	0.002166373
PARG15182	-2.683030015	-0.173091431	0.000672342	0.002226671
PARG12713	2.317944632	-0.838601287	0.000672342	0.002226671
PARG26349	-1.052854855	2.746197714	0.000678965	0.002248131
PARG07024	-1.074928424	2.027203575	0.000690606	0.002284749
PARG08210	1.091479355	2.131471276	0.000692658	0.002290572
PARG22968	-1.007083219	2.286577201	0.000693051	0.00229139
PARG08251	-1.135374338	1.387247557	0.000694396	0.002295352
PARG04897	-1.018471691	2.956656016	0.000696629	0.002302249
PARG00651	-1.168996404	1.873175736	0.000702055	0.002319692
PARG04378	-1.093468323	2.179309319	0.000706141	0.00233172
PARG15301	1.10638095	1.278054005	0.00071289	0.002353513
PARG22600	-1.037163403	3.142198831	0.000716121	0.002362035
PARG01380	-1.007463402	1.651113602	0.000745574	0.002452131
PARG14070	-1.050407121	2.665672814	0.000750402	0.00246646
PARG27856	-1.200099774	1.817321178	0.00075231	0.002471265
PARG00717	-1.117596361	2.55351133	0.000752336	0.002471265
PARG23996	-1.703925154	1.109511349	0.000757333	0.002485078
PARG06229	-1.170871152	2.123107986	0.000768626	0.002517399
PARG25984	-1.44455968	-0.32073533	0.00078514	0.002568433
PARG11909	1.522987175	0.727247897	0.000785354	0.002568433
PARG16805	1.506764756	3.59461025	0.000785354	0.002568433
PARG20318	-1.123485757	2.345373051	0.000787776	0.002575282
PARG10416	1.486101792	0.151529648	0.000792361	0.002588115
PARG20197	-1.034490426	2.269137612	0.000799892	0.002609998
PARG12192	-2.409673048	0.12941599	0.000804721	0.002625211
PARG11918	-5.018714508	-0.384525977	0.000807082	0.00263182
PARG14188	-5.00900748	0.359948317	0.000807082	0.00263182
PARG04662	1.032457858	1.974071978	0.000825505	0.002684646
PARG10666	1.00054397	2.551613629	0.000828918	0.002694626
PARG23413	-1.179703119	1.331347939	0.000839637	0.002727777
PARG06488	-1.305930117	1.528678261	0.000850194	0.002760361
PARG23995	-1.195262585	0.968090359	0.000862623	0.002797821
PARG30220	1.076620742	2.036845253	0.000870159	0.002820515
PARG02096	-1.290176456	1.131067954	0.000903895	0.002921421
PARG05658	-1.353626466	1.386200923	0.00091463	0.002953586
PARG25130	-2.254132141	-0.685473304	0.000917882	0.002960533
PARG00194	-1.403260272	1.056454732	0.000925251	0.002982464
PARG18267	-1.01202079	1.459111319	0.000925951	0.002984105
PARG22931	1.353021283	1.193867593	0.000935693	0.003012649

PARG01093	1.698167909	0.757397844	0.00093771	0.003018284
PARG16557	-2.821550212	0.413192231	0.000965181	0.00310035
PARG16035	-1.604557008	-0.176920241	0.00097708	0.003136645
PARG13338	1.037880183	2.491785847	0.000979939	0.003144539
PARG27796	-1.458309608	0.222358352	0.000981873	0.003149458
PARG25377	1.435112643	0.725627532	0.00098897	0.003170925
PARG20743	1.450547443	2.05138814	0.000994924	0.003188061
PARG21863	-1.610111674	0.442274279	0.00099709	0.003192685
PARG13703	-1.586474565	0.202356095	0.001002356	0.003208602
PARG30013	1.498408426	1.420734032	0.001003195	0.003209977
PARG05908	1.491875005	-0.506840689	0.001003195	0.003209977
PARG06032	-2.041124053	-0.08404856	0.001012039	0.003233004
PARG12832	-1.287173498	0.718949935	0.001019758	0.003257001
PARG06154	-3.318445706	-0.433216748	0.001023013	0.003265096
PARG02779	-2.622826924	-0.479931012	0.001027615	0.003277426
PARG25455	1.210056331	0.76728183	0.001032936	0.003293731
PARG14238	-1.306410805	0.126082247	0.001035542	0.00330039
PARG23093	1.051295086	1.490053551	0.001056168	0.003359621
PARG23465	1.043447496	2.017406411	0.001056168	0.003359621
PARG07121	-1.111429034	2.326242837	0.001057992	0.003364742
PARG19118	-1.956775699	-0.087654097	0.0010886	0.003453686
PARG30388	-1.036036367	1.721283562	0.001091929	0.003462149
PARG07771	-1.029458855	2.188818436	0.001091929	0.003462149
PARG00470	1.319401492	0.784818377	0.001101228	0.003488814
PARG09261	1.07144206	1.750845779	0.001114234	0.003527884
PARG16915	1.25600481	2.875078642	0.001124173	0.0035572
PARG29262	-1.179715589	1.627724762	0.001125079	0.003558633
PARG26404	1.01814413	2.109220781	0.001134142	0.003583688
PARG26623	1.01814413	2.109220781	0.001134142	0.003583688
PARG20409	-1.092828766	1.882328322	0.001138513	0.003595327
PARG08733	-1.916886523	-0.066185004	0.001149481	0.003627047
PARG17536	1.87863878	0.139792416	0.001149481	0.003627047
PARG04578	-1.12607155	1.810567237	0.001163298	0.003667694
PARG15792	-1.099516897	1.500506864	0.001164246	0.00366921
PARG19793	-1.442035651	4.798676613	0.001168889	0.003683104
PARG06760	-1.420212728	1.750035947	0.001185553	0.003729583
PARG01299	1.246951243	0.600442399	0.001185778	0.003729583
PARG19892	-1.102224712	0.927750261	0.001193212	0.003749957
PARG09184	-2.071981022	0.253537998	0.001199951	0.003770382
PARG15411	-1.110755561	1.866900308	0.001202487	0.003776837
PARG04112	-1.129867784	1.550243393	0.001223098	0.003838503
PARG02961	-1.876539408	-0.474159352	0.001232302	0.003865842
PARG20215	-1.484157727	0.079784301	0.001232798	0.003866626
PARG08037	1.010649653	2.289776786	0.001247308	0.00390745
PARG16770	1.44423955	-0.601988891	0.001259872	0.003942876
PARG28596	-1.036933621	2.305858572	0.001271667	0.003975036

PARG04345	-1.719764943	-0.184703965	0.001275441	0.003985246
PARG23873	-2.443213126	-0.396971495	0.00129528	0.0040408
PARG08231	1.299123112	0.777020927	0.001325197	0.004129207
PARG08332	1.063311281	1.664813907	0.001344944	0.004183265
PARG27825	1.319887453	1.135639114	0.001376134	0.004275194
PARG29740	-1.029573227	2.077102644	0.001383744	0.004295435
PARG25057	-1.048141066	1.922515386	0.001424148	0.004400841
PARG16133	-1.406952222	-0.053607243	0.001428751	0.004414197
PARG00007	-1.024379079	1.227062684	0.001436781	0.004435515
PARG21504	-1.173705824	0.575821005	0.001465282	0.004514622
PARG23133	1.177738518	0.802632436	0.001473787	0.004535483
PARG11134	4.947378902	-0.379518493	0.001489124	0.004570141
PARG08094	4.94250858	-0.705991842	0.001489124	0.004570141
PARG05510	-4.906788475	-0.594450046	0.001489124	0.004570141
PARG17748	-4.906547324	-0.274431054	0.001489124	0.004570141
PARG22096	-4.904460622	-0.045691485	0.001489124	0.004570141
PARG17108	-4.903947172	-1.024483998	0.001489124	0.004570141
PARG21566	-4.901862949	-0.209095234	0.001489124	0.004570141
PARG12725	-4.900565269	-0.478102935	0.001489124	0.004570141
PARG17970	-4.900324764	-0.576027408	0.001489124	0.004570141
PARG05885	-1.134597164	2.25274883	0.001506208	0.004618058
PARG07712	-1.936093323	0.505678737	0.001534559	0.004696727
PARG11283	-1.921850135	0.003662284	0.001534559	0.004696727
PARG11079	-1.491886296	2.138173042	0.001561675	0.004772274
PARG02456	-2.560869921	1.086177443	0.001570152	0.004797244
PARG24706	-2.746311881	0.171417145	0.001576871	0.004814476
PARG07254	-2.677657327	0.094225271	0.001576871	0.004814476
PARG03532	-1.440927177	-0.119383186	0.001586876	0.004838115
PARG02947	-1.823325747	-0.441397081	0.001596885	0.004865665
PARG22750	1.42817059	0.561177278	0.001610856	0.004905378
PARG18248	-1.352013366	1.543808072	0.001667386	0.00506573
PARG29458	-1.652062883	0.976478385	0.001670029	0.005072779
PARG02352	1.186456654	1.471243008	0.001675368	0.005087026
PARG08677	1.00877479	2.93112736	0.001681935	0.005104992
PARG10729	-1.756497014	0.049941162	0.001686068	0.005116548
PARG06838	-1.635589648	0.490567712	0.001690966	0.005129426
PARG27807	1.591522358	1.105753459	0.001692526	0.005133168
PARG12989	1.037951604	1.583142916	0.001731837	0.005240241
PARG04554	3.53068628	-0.159982054	0.001756313	0.00530612
PARG18173	-1.360988533	-0.014016054	0.001785625	0.005386385
PARG20887	-2.009623478	0.148425605	0.00178809	0.005391748
PARG17357	1.260862408	-0.181186678	0.001820284	0.005481452
PARG29106	-1.076424057	1.294904171	0.001874552	0.005628675
PARG15229	1.202811002	0.84749913	0.0018961	0.005689024
PARG04713	-1.050526869	2.187591572	0.001914949	0.005742287
PARG01300	1.45934407	0.488645409	0.001997065	0.005963465

PARG18476	-2.887902227	0.535809997	0.002038892	0.006080133
PARG07813	-2.885270122	0.1270536	0.002038892	0.006080133
PARG20908	-2.390571805	-0.825352713	0.002038892	0.006080133
PARG01742	1.117770494	1.037831117	0.002043336	0.006090632
PARG10718	1.078395217	0.85179064	0.002050687	0.006106161
PARG20229	-1.292815421	0.10727444	0.002130286	0.006321144
PARG24902	1.012514304	2.250104378	0.00217203	0.006429669
PARG11821	1.540582955	0.166433465	0.00219211	0.006481774
PARG20329	-1.59607363	1.22210512	0.002221118	0.006557665
PARG28127	1.661888992	0.287371412	0.002243542	0.006620136
PARG12666	-1.7870637	0.193739841	0.002247131	0.006629479
PARG24461	-1.802962408	0.185705585	0.002259548	0.006661105
PARG12211	-1.017531354	1.738069386	0.002288499	0.006738859
PARG25132	-1.209323464	0.277869727	0.002293598	0.006748809
PARG15107	-1.250060477	0.50896733	0.002301161	0.006767258
PARG02137	-1.034785293	2.048587727	0.002313075	0.006799749
PARG03653	-1.171676989	1.740450772	0.002322292	0.006825564
PARG06588	-1.316228965	0.570921605	0.002370407	0.006953962
PARG00912	1.052815005	1.157091208	0.002419631	0.007090418
PARG29530	1.029356082	2.286131583	0.002423706	0.007101033
PARG00636	1.16375957	0.618357318	0.002434226	0.007130525
PARG13900	-1.117212994	1.835061924	0.002442092	0.007149565
PARG19148	-1.102019262	1.548645221	0.002442092	0.007149565
PARG11235	1.022372527	1.759652206	0.002448617	0.007163321
PARG08773	1.142330648	1.001081443	0.002467841	0.007212839
PARG20163	-1.39884702	0.287697337	0.002510955	0.007331903
PARG07697	-2.673518	-0.673989843	0.002577856	0.007512008
PARG11442	-2.633428381	-0.413783658	0.002577856	0.007512008
PARG06376	-1.131534921	0.892805943	0.002579011	0.007513978
PARG12721	-1.446512225	-0.174072615	0.00264887	0.007690395
PARG02749	2.040417171	1.280207443	0.002661869	0.007722421
PARG29076	-1.34506177	-0.45446703	0.002681412	0.007771937
PARG25851	-1.026241576	1.412216157	0.002710951	0.007843071
PARG06288	1.453554911	0.260027113	0.002717973	0.007861938
PARG13234	-1.095992066	1.687394691	0.002729877	0.007887653
PARG16635	4.797908991	0.997588642	0.002765516	0.007975946
PARG27079	-4.782065278	-0.418110807	0.002765516	0.007975946
PARG12055	-4.779953025	-0.238122796	0.002765516	0.007975946
PARG13746	-4.757715633	1.678951676	0.002765516	0.007975946
PARG15872	-2.099391902	-0.052410641	0.002846741	0.008187451
PARG12877	-1.090769428	1.390277895	0.002847718	0.008187451
PARG19667	-1.011076003	1.618749362	0.002926854	0.008395699
PARG28007	-1.958277183	0.923180118	0.002967707	0.008499739
PARG21123	-1.950641941	-0.179571921	0.002967707	0.008499739
PARG07901	-1.949978706	0.476741143	0.002967707	0.008499739
PARG29460	1.525336871	-0.148952805	0.002970248	0.008503914

PARG15342	1.00208853	1.406541618	0.003000628	0.008575251
PARG07074	1.53718863	0.352451563	0.003018025	0.008617125
PARG11128	1.127060698	2.80647947	0.00303571	0.008664469
PARG19462	1.757369678	-0.44814678	0.003070643	0.008746679
PARG00848	1.060919707	1.169505897	0.003135273	0.008912986
PARG22573	-1.302642405	-0.570253479	0.003166511	0.008988768
PARG07131	2.265231722	-0.922671168	0.003208044	0.009101729
PARG09222	-1.216229074	0.418361879	0.003437056	0.009667109
PARG07399	-1.145671418	0.843133262	0.003477276	0.009770229
PARG00596	-1.117245995	1.552253651	0.003477276	0.009770229
PARG29531	-1.411036934	1.198945545	0.003479215	0.009773929
PARG13653	-1.45056881	-0.974255441	0.003592109	0.010056881
PARG18528	-1.149469284	1.250795837	0.003604544	0.010088095
PARG13911	1.106369138	1.09618525	0.003642912	0.010182769
PARG16474	-2.464119853	-0.74655814	0.003657417	0.010217857
PARG04843	-1.349791915	-0.383173196	0.003666006	0.01024003
PARG29186	-1.013746273	2.075732092	0.003836697	0.010669348
PARG17703	1.179524306	1.373939799	0.003883233	0.010779663
PARG27676	1.482655122	-0.296668949	0.003926832	0.010889136
PARG23414	-2.190729649	0.107188359	0.003957315	0.010963982
PARG19838	1.210616813	1.360526968	0.004012633	0.011098576
PARG22971	1.004849033	0.829186391	0.004034764	0.01115297
PARG22805	1.157306315	0.588320828	0.004035992	0.011154402
PARG27890	1.31071338	-0.272639888	0.004113519	0.01135068
PARG24898	-1.692248874	0.303155162	0.004121166	0.011367784
PARG00217	-1.237501605	0.294106771	0.004133733	0.01139444
PARG26700	1.179333049	-0.061944419	0.004133733	0.01139444
PARG28258	-1.094484741	3.13079356	0.004213223	0.011595228
PARG03437	2.384640542	0.791094447	0.004215024	0.011598149
PARG15290	1.091838638	-0.251024956	0.004225991	0.011624251
PARG08582	1.800962923	0.629258212	0.004235879	0.011644269
PARG29741	-1.37606661	0.350495726	0.004277232	0.011744619
PARG19379	-1.212327152	0.061919969	0.004307214	0.011822808
PARG02375	-1.382622713	0.243734485	0.004447943	0.012160189
PARG09624	-1.319310294	0.034771757	0.004545486	0.012383727
PARG10715	1.27620857	-0.457499757	0.004545486	0.012383727
PARG09175	1.103829685	-0.235794316	0.004576802	0.012458235
PARG03129	-1.453205703	-0.259855771	0.004625119	0.012565787
PARG27782	1.150059555	0.497261434	0.004755391	0.01288404
PARG03114	1.062965784	0.633819097	0.004844373	0.013113807
PARG14655	-1.061489424	1.222986572	0.004957258	0.013387204
PARG19436	-1.372242164	0.883382462	0.00495882	0.013388392
PARG27969	1.452216831	0.426326415	0.004994645	0.013467183
PARG20097	-2.242991056	-0.042210908	0.005043113	0.013581538
PARG21263	-1.087754231	0.731995485	0.005105685	0.013717106
PARG21998	-1.068204904	0.463639402	0.005105685	0.013717106

PARG08253	-4.644798111	-0.398524064	0.005170313	0.013869373
PARG24725	1.359061637	0.224322027	0.005424611	0.014460103
PARG26225	1.346724739	-0.201552545	0.005424611	0.014460103
PARG08970	-1.233118902	-0.315840781	0.005437803	0.01448543
PARG18249	-1.680766492	-0.321427016	0.005551881	0.014753677
PARG02734	-1.646317574	-0.544815325	0.005551881	0.014753677
PARG17960	-2.371561131	-0.665493671	0.005571048	0.014797683
PARG30069	-1.22585584	1.761027981	0.005629371	0.014929865
PARG05097	1.172476182	0.266896841	0.0056994	0.015095187
PARG08758	1.630302823	1.90614807	0.005720387	0.015143108
PARG08529	-1.579535613	0.534921212	0.005720387	0.015143108
PARG21711	1.002852146	1.039688313	0.005813551	0.015374178
PARG25152	-1.659093826	-0.07390806	0.005866704	0.015504296
PARG15548	1.929068248	-0.0815608	0.005872939	0.01551555
PARG16523	1.900652003	0.783977124	0.005872939	0.01551555
PARG05643	-1.109809175	1.037587275	0.005894155	0.015561125
PARG26212	-1.059893581	0.50266472	0.006042443	0.015893813
PARG16203	-1.435844074	0.211180567	0.006228227	0.016325048
PARG22619	1.198123362	0.103646231	0.006283709	0.016448502

Table S15C. Differentially expressed genes between CT and FR of *P. armeniaca*

Gene ID	logFC	logCPM	PValue	FDR
PARG19692	8.194625932	5.527498903	3.88E-101	6.09E-97
PARG15381	7.775809071	5.290849828	1.36E-94	1.07E-90
PARG19533	7.269719584	7.017536159	6.28E-94	3.28E-90
PARG21026	7.309493644	2.868134914	2.74E-61	1.07E-57
PARG06661	-5.224118737	8.521730883	1.54E-58	4.84E-55
PARG03330	-6.198812555	3.01220424	2.28E-56	5.97E-53
PARG27306	7.626262401	2.925176102	2.79E-56	6.26E-53
PARG16032	-5.255405146	6.776457845	6.76E-55	1.33E-51
PARG18610	5.028792686	6.241156788	3.93E-54	6.85E-51
PARG22703	4.970861505	4.068749086	4.81E-52	7.54E-49
PARG22270	-4.694817936	10.62900828	9.77E-51	1.39E-47
PARG08672	-4.982368735	7.872191368	4.64E-47	6.07E-44
PARG08769	-4.72322674	4.675093367	2.10E-46	2.54E-43
PARG05325	4.430558474	4.863539609	4.12E-45	4.62E-42
PARG19578	-5.491755574	3.928818032	4.90E-43	5.13E-40
PARG15733	-4.856043854	2.719437512	7.11E-42	6.98E-39
PARG03409	4.209194151	4.205965904	1.18E-41	1.09E-38
PARG28325	-4.991658437	3.380321358	2.40E-41	2.09E-38
PARG15232	-4.465956594	6.294050424	1.56E-40	1.29E-37
PARG28101	4.500985449	2.188905858	1.57E-38	1.23E-35
PARG05178	-3.854168337	9.08452174	1.79E-38	1.34E-35
PARG02889	3.962095074	4.256543409	4.20E-38	2.87E-35
PARG29961	3.94452043	4.283306634	4.21E-38	2.87E-35
PARG04601	-6.178212742	1.679781095	5.30E-38	3.47E-35
PARG16049	-5.093050739	3.438220242	7.26E-38	4.56E-35
PARG29414	-5.189555697	1.740831047	8.95E-37	5.40E-34
PARG11661	-4.069174368	3.86229958	2.29E-36	1.33E-33
PARG24964	-6.285505935	3.073968283	4.99E-36	2.80E-33
PARG27418	-7.633649857	1.23093659	5.27E-36	2.85E-33
PARG23196	4.139442218	3.293872628	4.05E-35	2.09E-32
PARG05813	-5.987211786	1.616514333	4.12E-35	2.09E-32
PARG16038	-5.027052295	2.042097083	1.74E-33	8.56E-31
PARG01289	-3.629108899	7.744459932	2.30E-33	1.09E-30
PARG15861	-3.738876182	7.08030409	7.68E-33	3.54E-30
PARG07409	-3.674602804	7.079841328	1.19E-32	5.35E-30
PARG07161	-3.582480174	6.12664434	1.49E-32	6.51E-30
PARG26386	-3.806045388	6.218715263	1.91E-32	8.10E-30
PARG10509	-4.630296431	3.532198821	2.53E-32	1.04E-29
PARG21594	-3.676303205	4.916077229	3.53E-32	1.42E-29
PARG20144	-3.61989593	3.852334062	3.61E-32	1.42E-29
PARG00129	-4.114055048	7.800788745	5.07E-32	1.94E-29
PARG25006	-5.973996293	2.701380384	9.35E-32	3.47E-29
PARG11012	-3.611467745	4.729359345	9.49E-32	3.47E-29

PARG24151	-9.058698779	1.048927538	1.79E-31	6.39E-29
PARG12287	-3.415676238	9.106313428	3.49E-31	1.21E-28
PARG03663	-4.246660031	3.492479814	3.54E-31	1.21E-28
PARG28160	-3.526744636	5.410272454	4.68E-31	1.56E-28
PARG20180	-3.800618316	4.791291055	6.78E-31	2.22E-28
PARG19099	-3.880771459	3.456774845	1.87E-30	5.98E-28
PARG21002	3.476404779	3.387538715	3.81E-30	1.20E-27
PARG04593	-6.465071033	0.547296843	8.26E-30	2.52E-27
PARG23869	-4.37074314	4.114756374	8.36E-30	2.52E-27
PARG12928	3.411464322	3.897305928	9.94E-30	2.94E-27
PARG19277	-3.567851626	4.460930975	1.23E-29	3.57E-27
PARG19461	3.348178465	4.503551283	2.02E-29	5.78E-27
PARG06846	-3.964923833	3.571271745	2.20E-29	6.16E-27
PARG11660	-4.776353864	1.469125788	2.77E-29	7.63E-27
PARG00885	-8.85292348	0.187497736	6.52E-29	1.77E-26
PARG13515	-4.237913542	5.477660361	1.09E-28	2.90E-26
PARG27864	-3.959173384	5.298117805	1.28E-28	3.35E-26
PARG24007	-3.87250314	3.532653923	3.00E-28	7.72E-26
PARG07452	-3.423536358	5.630375441	4.03E-28	1.02E-25
PARG22198	-4.079192754	3.559637494	6.08E-28	1.51E-25
PARG01380	-6.978119559	1.651113602	9.64E-28	2.36E-25
PARG16825	-3.571443827	4.404977238	1.07E-27	2.59E-25
PARG04598	-3.151189946	5.634842752	2.26E-27	5.38E-25
PARG27772	3.09836874	9.091796253	2.62E-27	6.15E-25
PARG05771	-4.422654832	2.34292414	2.75E-27	6.34E-25
PARG14504	-3.768154471	7.19905561	4.10E-27	9.33E-25
PARG27710	-4.401374803	4.607525072	4.58E-27	1.03E-24
PARG21037	-3.484610715	4.02928581	7.05E-27	1.56E-24
PARG19023	3.062194154	8.981044124	8.13E-27	1.77E-24
PARG18487	-3.699254391	7.505257791	1.19E-26	2.55E-24
PARG23920	-4.171955406	2.635304304	1.50E-26	3.15E-24
PARG18533	-3.599528768	6.652379432	1.51E-26	3.15E-24
PARG19094	-3.475379086	6.001242263	2.23E-26	4.60E-24
PARG04915	-3.297655957	4.786502048	2.35E-26	4.80E-24
PARG21491	-4.016971002	4.892650306	3.07E-26	6.18E-24
PARG11346	-3.268429807	4.470138519	3.69E-26	7.33E-24
PARG13275	-3.21616027	6.557184697	4.50E-26	8.83E-24
PARG05741	-3.309756412	7.074924449	5.63E-26	1.09E-23
PARG08239	-3.531559756	2.612002698	7.33E-26	1.40E-23
PARG04597	-6.224821936	1.854092794	7.73E-26	1.46E-23
PARG24356	-3.200961863	5.022060584	9.54E-26	1.78E-23
PARG20139	-3.869111544	1.463632534	1.09E-25	2.02E-23
PARG18624	-3.27909904	5.169394741	1.14E-25	2.08E-23
PARG13796	-3.684103542	7.638701005	1.30E-25	2.35E-23
PARG02007	-3.185863443	8.186296047	1.64E-25	2.93E-23
PARG06740	-4.138085362	4.55346048	1.87E-25	3.29E-23

PARG23374	-2.983041788	8.139020289	2.74E-25	4.78E-23
PARG11135	-4.521843043	2.258196181	2.92E-25	5.04E-23
PARG07670	-3.221671806	5.629482856	4.28E-25	7.29E-23
PARG05428	3.814525952	1.433539925	4.32E-25	7.29E-23
PARG25647	3.002920049	8.278113323	5.70E-25	9.52E-23
PARG12344	-3.090667389	5.806527848	6.56E-25	1.08E-22
PARG30013	3.479396372	1.420734032	1.02E-24	1.67E-22
PARG11915	2.917986592	7.223375872	1.06E-24	1.72E-22
PARG13772	-2.957425162	8.142022613	1.10E-24	1.77E-22
PARG05624	3.416749063	4.588069614	1.44E-24	2.29E-22
PARG28340	4.856366582	0.076127742	2.02E-24	3.17E-22
PARG11251	-4.181258678	2.17747789	2.29E-24	3.54E-22
PARG05179	-3.838092206	3.195865925	2.30E-24	3.54E-22
PARG21579	-4.915138849	3.65352095	2.34E-24	3.57E-22
PARG10540	-3.538781822	6.679984331	2.96E-24	4.47E-22
PARG29789	-3.287521476	5.054221315	3.44E-24	5.14E-22
PARG07354	-2.958192964	8.588210428	4.49E-24	6.64E-22
PARG15598	-3.525436195	2.119055431	5.22E-24	7.65E-22
PARG11889	-6.644012572	3.375308237	7.17E-24	1.04E-21
PARG02687	-3.243616869	3.495725319	8.62E-24	1.24E-21
PARG15814	-5.104546698	3.043896426	9.63E-24	1.37E-21
PARG05368	-2.971379017	5.415144234	1.07E-23	1.50E-21
PARG15912	-2.861471799	8.774559407	1.08E-23	1.50E-21
PARG07306	-6.046616648	2.11535991	1.08E-23	1.50E-21
PARG27931	3.421365101	1.695405545	1.35E-23	1.86E-21
PARG13867	-4.430447352	3.10885432	2.01E-23	2.75E-21
PARG03467	2.819046411	7.186372012	2.60E-23	3.52E-21
PARG04423	-5.993693672	1.746788444	3.52E-23	4.72E-21
PARG06891	-3.10337875	5.594203155	4.40E-23	5.86E-21
PARG02278	2.790812134	7.232505963	5.62E-23	7.41E-21
PARG02372	-3.721188289	3.333630102	5.73E-23	7.50E-21
PARG07885	-3.26525906	5.193284832	6.15E-23	7.98E-21
PARG25155	4.196356853	0.772143672	6.69E-23	8.60E-21
PARG12386	-3.787753364	6.466359035	8.17E-23	1.04E-20
PARG20955	2.813105865	6.079076006	1.06E-22	1.34E-20
PARG19091	-3.5015415	3.86374875	1.10E-22	1.38E-20
PARG29465	2.826329339	5.229748007	1.56E-22	1.94E-20
PARG20785	-5.840751496	0.929062306	1.57E-22	1.94E-20
PARG23736	-3.173542158	1.798890447	1.59E-22	1.94E-20
PARG21687	4.563726763	0.668228187	1.69E-22	2.05E-20
PARG27557	-2.769698885	8.628828149	1.72E-22	2.07E-20
PARG10062	-3.35650598	1.47991312	1.78E-22	2.13E-20
PARG02173	-2.99530129	6.27359899	2.16E-22	2.57E-20
PARG26908	-2.864280949	5.274268814	2.28E-22	2.69E-20
PARG12550	5.20653284	2.341662331	2.56E-22	3.00E-20
PARG08216	-2.746178914	5.877418313	7.83E-22	9.11E-20

PARG22911	6.533408449	0.711946559	9.32E-22	1.08E-19
PARG07776	-3.158063962	3.703688967	1.31E-21	1.51E-19
PARG24659	-2.846956501	5.320749816	2.56E-21	2.92E-19
PARG04592	-2.878107552	7.368307661	2.96E-21	3.35E-19
PARG02473	-3.748142553	3.031582355	4.01E-21	4.50E-19
PARG11887	-8.140039376	0.422389711	4.72E-21	5.24E-19
PARG20652	3.279127987	2.44646061	4.74E-21	5.24E-19
PARG26836	-2.721355015	6.033112743	5.04E-21	5.54E-19
PARG00019	-4.841474891	3.131287191	6.19E-21	6.75E-19
PARG05892	-3.570221588	2.442012756	7.27E-21	7.87E-19
PARG28358	-3.136751179	5.499880447	1.61E-20	1.73E-18
PARG10556	-3.576675514	5.517692902	1.79E-20	1.91E-18
PARG21489	-2.599220713	7.475755239	2.27E-20	2.41E-18
PARG23933	-2.718679974	5.523510656	3.44E-20	3.63E-18
PARG19184	-2.807419011	4.243953774	4.67E-20	4.89E-18
PARG21733	-3.111738605	1.047846825	5.28E-20	5.49E-18
PARG27703	2.748164423	3.862008095	5.75E-20	5.92E-18
PARG16548	-2.71933324	5.408060772	5.77E-20	5.92E-18
PARG23453	-2.63188946	6.94086117	6.03E-20	6.15E-18
PARG17986	3.234847304	0.657213728	6.18E-20	6.23E-18
PARG27271	2.691309198	4.07986731	6.19E-20	6.23E-18
PARG27980	-2.716172156	6.349339303	6.38E-20	6.38E-18
PARG23111	-4.100103167	5.028134087	7.09E-20	7.04E-18
PARG18971	-2.605210509	5.800736283	7.82E-20	7.72E-18
PARG21543	-2.545824317	10.84875741	8.29E-20	8.14E-18
PARG12147	-3.067991957	5.242157643	1.27E-19	1.24E-17
PARG06682	-2.787840987	5.377153059	1.31E-19	1.27E-17
PARG25648	3.257791946	5.370977861	1.32E-19	1.27E-17
PARG19605	-2.825712064	4.069731315	1.35E-19	1.29E-17
PARG13690	-2.633418067	5.962498157	1.41E-19	1.34E-17
PARG02753	3.105162187	2.538899807	1.81E-19	1.72E-17
PARG27647	-2.855665827	3.671098548	2.00E-19	1.88E-17
PARG16293	2.641146538	4.592627314	2.23E-19	2.08E-17
PARG28177	2.603374344	3.75000117	2.43E-19	2.26E-17
PARG26618	-3.371864068	5.232021037	3.94E-19	3.64E-17
PARG02979	-2.724329176	7.896442031	4.78E-19	4.38E-17
PARG12409	-2.74436628	4.447372508	4.89E-19	4.47E-17
PARG18488	-3.010915279	7.189478947	5.53E-19	5.02E-17
PARG13517	-2.5103631	8.130579969	5.82E-19	5.25E-17
PARG29227	-3.866944338	3.076707782	6.35E-19	5.70E-17
PARG27807	3.157100928	1.105753459	6.74E-19	6.01E-17
PARG15517	-3.837975499	1.252908741	7.16E-19	6.35E-17
PARG06748	2.636249998	3.684664679	7.54E-19	6.65E-17
PARG12710	2.47504936	5.734228816	1.13E-18	9.86E-17
PARG16024	5.156434486	0.345197933	1.13E-18	9.86E-17
PARG07560	4.473245566	-0.305502385	1.14E-18	9.86E-17

PARG14524	-3.241706086	2.379664693	1.15E-18	9.91E-17
PARG27197	-4.056828568	2.969121562	1.22E-18	1.04E-16
PARG23199	-2.476858505	6.512571637	1.48E-18	1.26E-16
PARG07008	3.129343804	1.226705753	1.52E-18	1.29E-16
PARG19632	-2.638281368	7.415529367	1.65E-18	1.39E-16
PARG24219	-6.125864248	2.219418763	1.67E-18	1.40E-16
PARG05745	2.493091937	5.444083195	1.82E-18	1.52E-16
PARG19393	-4.437408669	4.149109822	2.28E-18	1.89E-16
PARG09013	2.437528503	7.099883102	2.31E-18	1.91E-16
PARG05880	-2.564268565	6.755736672	2.44E-18	2.01E-16
PARG23932	-5.495899802	2.419863414	2.66E-18	2.17E-16
PARG14831	-2.94139735	6.995467098	3.01E-18	2.44E-16
PARG01856	-3.351349486	4.244721835	3.13E-18	2.54E-16
PARG06694	3.01388037	1.999395229	3.43E-18	2.76E-16
PARG17771	-2.496848182	4.977079533	5.20E-18	4.17E-16
PARG00327	-2.464267589	5.321404269	6.34E-18	5.05E-16
PARG10953	-4.559430164	3.732329695	6.57E-18	5.21E-16
PARG01157	-3.851283599	2.83898373	6.68E-18	5.27E-16
PARG15180	-3.360860691	6.582881807	6.72E-18	5.27E-16
PARG16624	-2.892335455	4.375568882	7.11E-18	5.55E-16
PARG10465	2.667108183	3.344289936	8.48E-18	6.59E-16
PARG11948	-3.571807965	5.749786766	8.88E-18	6.86E-16
PARG11773	-3.795288449	4.629658493	9.98E-18	7.68E-16
PARG01280	-2.9968144	4.069748136	1.21E-17	9.27E-16
PARG27201	2.830263531	1.201723664	1.35E-17	1.03E-15
PARG06312	5.413949683	0.069919737	1.73E-17	1.32E-15
PARG04591	-7.726209133	-0.6682106	1.96E-17	1.48E-15
PARG23356	2.49206417	4.177658409	2.00E-17	1.50E-15
PARG11389	4.216311438	0.568825111	2.06E-17	1.54E-15
PARG02771	2.981536929	3.876021523	2.31E-17	1.71E-15
PARG07500	2.42912604	5.979898124	2.64E-17	1.95E-15
PARG20780	-2.530265595	4.408299949	3.42E-17	2.52E-15
PARG12245	2.747150796	5.044094102	3.66E-17	2.69E-15
PARG14119	-3.497249545	3.876201311	3.70E-17	2.70E-15
PARG29550	-5.290814136	3.409375129	4.45E-17	3.24E-15
PARG07854	-3.837539385	3.881042266	4.56E-17	3.30E-15
PARG26737	2.367072219	4.956207236	4.95E-17	3.57E-15
PARG06897	-2.457956876	5.203926895	5.20E-17	3.73E-15
PARG23485	-4.60599296	1.355469634	6.62E-17	4.72E-15
PARG24734	-2.386595607	6.914947693	7.45E-17	5.29E-15
PARG01221	-2.500666745	6.161981339	7.83E-17	5.54E-15
PARG23885	-5.915600874	1.789071315	8.71E-17	6.13E-15
PARG15845	-2.330466244	8.71924524	9.50E-17	6.66E-15
PARG16777	-3.267631882	6.62937097	1.18E-16	8.20E-15
PARG11371	2.29466573	8.899758252	1.18E-16	8.23E-15
PARG28109	-3.132462618	3.377534205	1.20E-16	8.30E-15

PARG20524	-2.297149892	9.524743505	1.42E-16	9.80E-15
PARG15930	-2.712544763	3.321004119	1.47E-16	1.01E-14
PARG29762	-2.764413931	6.758164943	1.64E-16	1.12E-14
PARG24836	3.121930731	3.733222342	2.21E-16	1.50E-14
PARG19987	-3.728372104	1.791389487	2.24E-16	1.52E-14
PARG08327	-2.846904192	4.686603912	2.29E-16	1.55E-14
PARG06318	-3.394013529	4.498106315	2.44E-16	1.62E-14
PARG23054	-3.384609852	1.549235347	2.44E-16	1.62E-14
PARG23934	-3.292698747	3.915542021	2.44E-16	1.62E-14
PARG13663	-2.346125591	6.444603576	2.87E-16	1.90E-14
PARG19427	-2.414251746	4.36881342	3.03E-16	2.00E-14
PARG17643	-2.605194943	4.268922532	3.06E-16	2.01E-14
PARG18849	2.30209468	5.345647396	4.30E-16	2.81E-14
PARG08658	-2.404370263	4.138553274	4.39E-16	2.85E-14
PARG12720	2.257865261	7.158930751	4.40E-16	2.85E-14
PARG00513	-3.874730494	4.049504603	5.45E-16	3.52E-14
PARG12503	-5.265934721	0.507528065	5.83E-16	3.75E-14
PARG03568	2.361927467	8.089594688	6.25E-16	4.01E-14
PARG05802	-2.615491676	2.942886988	6.61E-16	4.22E-14
PARG13600	-2.32270717	6.336914501	7.65E-16	4.86E-14
PARG00107	-3.167862871	2.775589162	7.74E-16	4.90E-14
PARG08093	-2.444023442	6.065422994	9.19E-16	5.80E-14
PARG05593	2.635638195	2.728274933	1.01E-15	6.36E-14
PARG14848	-7.532498156	0.903458617	1.15E-15	7.19E-14
PARG20901	-2.213528844	8.944874176	1.20E-15	7.50E-14
PARG13050	-2.816165192	2.015205065	1.38E-15	8.54E-14
PARG06794	2.851816272	1.472652002	1.47E-15	9.06E-14
PARG08340	2.214884527	6.385860039	1.51E-15	9.32E-14
PARG02229	-2.814912883	4.300193414	1.62E-15	9.96E-14
PARG11736	-2.202975982	8.961552341	1.88E-15	1.15E-13
PARG14123	-2.395588664	5.469574105	2.09E-15	1.27E-13
PARG07579	-2.344372717	4.929218089	2.16E-15	1.31E-13
PARG26913	2.225514321	5.129740196	2.19E-15	1.32E-13
PARG01043	2.183150496	9.265096106	2.60E-15	1.56E-13
PARG27269	2.283090942	6.227942537	2.70E-15	1.62E-13
PARG28527	-2.543585621	3.206882951	2.84E-15	1.69E-13
PARG28246	-2.55058552	2.260454238	3.13E-15	1.86E-13
PARG20786	-2.23998914	5.823186706	3.37E-15	1.99E-13
PARG12719	-3.070883015	2.007567644	3.44E-15	2.03E-13
PARG29469	-2.237374671	5.846144173	3.50E-15	2.06E-13
PARG03712	-3.464780718	2.893324981	3.59E-15	2.10E-13
PARG20953	-2.438240572	4.650615344	4.26E-15	2.48E-13
PARG01972	2.657236561	2.967130758	4.33E-15	2.52E-13
PARG08838	2.475080685	3.116340262	4.40E-15	2.55E-13
PARG04100	-2.198460448	5.567646126	5.12E-15	2.96E-13
PARG08105	-3.00114641	2.878278881	5.24E-15	3.01E-13

PARG15679	2.193033468	6.534068468	5.31E-15	3.04E-13
PARG06756	-3.165065717	2.701162583	5.56E-15	3.18E-13
PARG22757	-2.766565411	3.038428293	6.20E-15	3.53E-13
PARG27282	-2.191317467	5.894254307	6.48E-15	3.67E-13
PARG23974	2.279116225	3.079507663	6.51E-15	3.68E-13
PARG20523	-2.616296422	4.190782869	6.86E-15	3.86E-13
PARG29279	-2.621042323	3.607637874	6.89E-15	3.86E-13
PARG28046	-2.187625644	6.808319528	7.21E-15	4.03E-13
PARG12427	-2.214872579	4.678304475	7.58E-15	4.22E-13
PARG21231	2.315805824	3.639801532	7.64E-15	4.24E-13
PARG11947	-2.180721276	7.613069938	9.18E-15	5.08E-13
PARG09762	-2.674889913	4.740036724	9.73E-15	5.36E-13
PARG03544	4.701293658	1.138848689	1.04E-14	5.71E-13
PARG18310	-3.346334749	2.524247231	1.07E-14	5.87E-13
PARG22907	-2.130596701	7.352352949	1.15E-14	6.25E-13
PARG04908	-2.207617208	6.898835134	1.21E-14	6.56E-13
PARG16635	3.63996383	0.997588642	1.58E-14	8.56E-13
PARG25287	-2.387678845	3.66417025	1.72E-14	9.27E-13
PARG05887	-2.796009365	2.201803798	1.76E-14	9.47E-13
PARG24952	-3.173194647	6.544324624	1.88E-14	1.01E-12
PARG26047	-2.477875061	2.339537152	1.88E-14	1.01E-12
PARG07610	3.906836433	0.644130272	1.89E-14	1.01E-12
PARG27327	2.422886044	2.404255064	2.31E-14	1.23E-12
PARG07121	-5.619021607	2.326242837	2.63E-14	1.39E-12
PARG18725	-2.354978216	3.877566683	2.76E-14	1.45E-12
PARG26494	-3.740528975	1.189569754	2.82E-14	1.48E-12
PARG08314	2.10269405	7.003564976	3.13E-14	1.64E-12
PARG07840	-2.289994467	2.745929077	3.18E-14	1.66E-12
PARG12366	-4.94388012	-0.303092957	3.58E-14	1.86E-12
PARG18911	-3.574449421	2.056320218	3.66E-14	1.90E-12
PARG05690	-2.701543308	3.906140786	3.69E-14	1.90E-12
PARG15524	-2.512532644	4.29996539	4.10E-14	2.11E-12
PARG11549	-4.351225833	1.141999117	4.66E-14	2.39E-12
PARG18960	-3.717547679	3.192804626	4.75E-14	2.43E-12
PARG11417	-2.447657923	1.122746165	4.77E-14	2.43E-12
PARG27263	-7.303675084	0.13897947	6.04E-14	3.06E-12
PARG12404	-2.478605306	4.977456159	6.04E-14	3.06E-12
PARG23479	-2.169634174	5.303946111	6.61E-14	3.34E-12
PARG00754	2.1112429	4.765212861	7.00E-14	3.52E-12
PARG11929	3.071071686	2.136964314	7.17E-14	3.60E-12
PARG13566	-2.260129844	5.922553827	7.31E-14	3.65E-12
PARG01391	-3.796895724	0.092248019	7.95E-14	3.96E-12
PARG20935	2.781705542	0.694412401	8.30E-14	4.12E-12
PARG10988	-2.75505193	3.864003578	9.17E-14	4.54E-12
PARG12847	2.132862927	4.063156241	9.24E-14	4.56E-12
PARG15926	-2.095017502	7.28542008	9.36E-14	4.61E-12

PARG24635	2.711400459	2.065760996	1.06E-13	5.20E-12
PARG00712	-2.098483319	6.184494709	1.12E-13	5.46E-12
PARG06704	2.227505349	3.914286418	1.32E-13	6.43E-12
PARG19581	-2.046563901	6.835111425	1.37E-13	6.64E-12
PARG10890	-3.242126226	0.978375221	1.38E-13	6.67E-12
PARG13281	-2.04544233	7.005273921	1.65E-13	7.96E-12
PARG29555	-2.035336637	7.698583447	1.71E-13	8.22E-12
PARG23762	-2.238544969	5.249913755	1.86E-13	8.92E-12
PARG12498	2.167690055	3.322481041	1.89E-13	9.05E-12
PARG05978	-2.128389737	6.062004319	2.19E-13	1.05E-11
PARG19940	-2.061016483	5.462216445	2.44E-13	1.16E-11
PARG22178	-2.619543186	4.111717302	2.47E-13	1.17E-11
PARG16260	-2.083538709	5.04068101	2.69E-13	1.27E-11
PARG11925	-2.165794095	4.370255009	2.82E-13	1.33E-11
PARG13096	-2.102440662	5.521528382	2.84E-13	1.34E-11
PARG00429	-2.13799056	3.796496676	2.98E-13	1.40E-11
PARG27823	-7.190213201	-0.109799925	3.18E-13	1.49E-11
PARG28239	-3.887701446	5.52702782	3.20E-13	1.49E-11
PARG07939	-2.075388034	7.117016374	3.41E-13	1.59E-11
PARG02154	-3.176534413	3.976308616	3.66E-13	1.69E-11
PARG18891	-2.696392414	3.130795196	3.68E-13	1.70E-11
PARG08302	-2.302976778	4.220393702	3.92E-13	1.80E-11
PARG01136	2.212194636	3.499413338	3.94E-13	1.81E-11
PARG09169	-1.993347544	8.727327234	4.18E-13	1.91E-11
PARG06906	2.001915956	5.429511437	4.41E-13	2.01E-11
PARG12459	-2.995685331	3.106791114	4.53E-13	2.06E-11
PARG16214	-7.175211164	0.083245188	4.86E-13	2.20E-11
PARG03178	-3.046634476	2.790522913	5.17E-13	2.34E-11
PARG11094	2.164488571	3.003827894	5.24E-13	2.36E-11
PARG09573	-2.306441653	4.525113381	5.31E-13	2.39E-11
PARG29269	-2.391361653	4.865055789	5.33E-13	2.39E-11
PARG20944	-2.900982941	1.411035103	5.35E-13	2.39E-11
PARG13587	-3.701665547	3.860734379	5.51E-13	2.46E-11
PARG02478	2.031386513	4.69308581	5.91E-13	2.63E-11
PARG06632	2.679157078	3.038391352	6.04E-13	2.68E-11
PARG15191	1.979360911	6.639673867	6.28E-13	2.78E-11
PARG22083	-2.051952415	6.093142195	6.97E-13	3.07E-11
PARG07337	2.106576486	2.51798488	7.29E-13	3.20E-11
PARG26656	-7.171103872	0.365493808	7.44E-13	3.26E-11
PARG12665	-2.113792834	6.588776331	7.60E-13	3.32E-11
PARG13572	-2.018975048	6.293536832	7.65E-13	3.34E-11
PARG02923	3.518982042	1.031185789	7.67E-13	3.34E-11
PARG12334	-2.020339973	5.972885871	8.39E-13	3.64E-11
PARG13225	-2.447909272	4.983713218	9.02E-13	3.90E-11
PARG01460	-3.342912365	1.590646509	9.38E-13	4.04E-11
PARG08132	-2.796649092	3.434751264	9.66E-13	4.15E-11

PARG07267	-3.981375053	3.345111705	1.06E-12	4.56E-11
PARG22997	2.056377232	4.260663037	1.10E-12	4.72E-11
PARG12164	-7.09600995	0.282543183	1.14E-12	4.87E-11
PARG14573	-2.466850236	5.781011327	1.16E-12	4.91E-11
PARG08017	-2.015401506	4.828724625	1.20E-12	5.11E-11
PARG17726	-2.785646577	1.959837098	1.22E-12	5.15E-11
PARG18074	1.977795761	4.740433457	1.32E-12	5.55E-11
PARG08512	-4.773924022	0.526301005	1.36E-12	5.72E-11
PARG01874	-1.980023921	9.127555907	1.39E-12	5.83E-11
PARG09088	-2.276535654	6.759129309	1.52E-12	6.37E-11
PARG07328	-2.19669607	4.448691848	1.64E-12	6.86E-11
PARG08090	2.374330054	1.314742134	1.67E-12	6.94E-11
PARG12163	-2.276230331	5.940583714	1.75E-12	7.26E-11
PARG13079	-2.942938253	2.334195848	1.83E-12	7.57E-11
PARG05989	-1.969351621	6.430645549	1.91E-12	7.89E-11
PARG06042	-3.702305222	0.080146264	1.97E-12	8.12E-11
PARG06406	-4.72489248	0.221065484	1.98E-12	8.13E-11
PARG12619	2.460044125	0.308549717	2.04E-12	8.36E-11
PARG23238	1.931935412	7.351233441	2.06E-12	8.41E-11
PARG19431	-2.210681671	3.377581544	2.07E-12	8.44E-11
PARG20089	-2.142810389	3.806816755	2.30E-12	9.36E-11
PARG11797	-2.104172469	7.040665438	2.39E-12	9.67E-11
PARG13833	-2.292072021	4.636037125	2.39E-12	9.67E-11
PARG24443	-2.376056544	1.979701813	2.59E-12	1.05E-10
PARG11978	-2.866232238	1.806834271	2.74E-12	1.10E-10
PARG18810	-1.930751753	7.637751793	2.76E-12	1.11E-10
PARG00299	-2.051483903	5.716670837	2.81E-12	1.13E-10
PARG27348	1.928448689	6.233878812	2.91E-12	1.16E-10
PARG22962	1.929756756	5.454931849	3.03E-12	1.21E-10
PARG25356	1.936608289	5.254177487	3.22E-12	1.28E-10
PARG07303	1.964064083	4.528784792	3.24E-12	1.29E-10
PARG24194	-3.295099936	0.205547841	3.41E-12	1.35E-10
PARG11729	-2.509365089	4.15548738	3.44E-12	1.36E-10
PARG07377	-3.9094487	4.490976442	3.67E-12	1.44E-10
PARG23746	-2.261867333	0.980446044	3.77E-12	1.48E-10
PARG10515	1.93402369	3.966200624	4.00E-12	1.57E-10
PARG02323	-2.432601059	3.736401821	4.16E-12	1.62E-10
PARG12422	-2.009227889	5.144629054	4.17E-12	1.62E-10
PARG12056	-2.233146358	4.391300952	4.31E-12	1.67E-10
PARG04341	-2.058041196	5.076300496	4.36E-12	1.69E-10
PARG06615	-3.313290263	4.888758631	4.42E-12	1.71E-10
PARG02108	-1.910764358	6.860275978	4.59E-12	1.77E-10
PARG14424	-2.606819301	1.170578665	4.61E-12	1.77E-10
PARG22993	1.936649258	4.545871839	5.51E-12	2.11E-10
PARG28131	1.881440937	8.995679419	5.61E-12	2.15E-10
PARG13047	-2.375135829	2.885349396	5.62E-12	2.15E-10

PARG27133	-2.693363044	5.117970021	5.94E-12	2.26E-10
PARG23829	-2.138189695	4.316701143	6.21E-12	2.36E-10
PARG29228	-2.265033883	3.249996202	6.83E-12	2.59E-10
PARG12904	1.886171498	6.163616631	7.43E-12	2.81E-10
PARG12763	-2.366137931	3.02508448	8.13E-12	3.07E-10
PARG02688	-1.937020967	3.424955099	8.22E-12	3.09E-10
PARG05524	2.300900406	0.779498962	9.21E-12	3.46E-10
PARG15502	-2.139999175	3.317178804	9.79E-12	3.67E-10
PARG19556	-2.462217087	2.599165448	1.01E-11	3.78E-10
PARG27966	-2.492471779	2.984805841	1.08E-11	4.02E-10
PARG18110	-2.070973035	8.23317952	1.10E-11	4.10E-10
PARG08770	-1.918144428	6.140735019	1.23E-11	4.56E-10
PARG16083	-2.679275369	3.86885929	1.30E-11	4.81E-10
PARG19954	-4.605911288	1.047331195	1.34E-11	4.95E-10
PARG07644	-3.770475129	4.249838185	1.44E-11	5.30E-10
PARG07292	-1.88791624	6.504011611	1.46E-11	5.38E-10
PARG19506	1.840185312	8.02584316	1.59E-11	5.81E-10
PARG20251	-6.949845646	-0.364828703	1.60E-11	5.87E-10
PARG21324	-2.031324637	3.705023318	1.63E-11	5.96E-10
PARG02942	-1.891121329	6.56063026	1.67E-11	6.08E-10
PARG06597	-1.935588078	4.339427335	1.76E-11	6.40E-10
PARG06052	3.672334495	1.174285754	1.78E-11	6.44E-10
PARG22208	-1.915009581	5.116470623	1.78E-11	6.45E-10
PARG14473	-1.968871853	3.958383737	1.79E-11	6.46E-10
PARG04143	-2.032820137	3.671211994	1.86E-11	6.69E-10
PARG01207	-2.489655584	3.553698982	1.89E-11	6.79E-10
PARG00616	-1.897986014	6.106174931	2.04E-11	7.31E-10
PARG14065	-2.26389009	3.706237232	2.18E-11	7.79E-10
PARG14619	-2.39842142	2.080165774	2.54E-11	9.07E-10
PARG07770	-2.11021754	2.868235982	2.66E-11	9.47E-10
PARG12159	-2.932800997	2.866468169	2.91E-11	1.03E-09
PARG22751	-1.943876488	5.177206152	3.09E-11	1.10E-09
PARG03543	4.242139054	-0.579801391	3.18E-11	1.13E-09
PARG18553	-1.97757155	4.370403014	3.23E-11	1.14E-09
PARG08360	2.417156226	4.218518664	3.28E-11	1.15E-09
PARG19553	-1.989780431	3.781767506	3.59E-11	1.26E-09
PARG28089	-1.908331935	7.170295455	3.65E-11	1.28E-09
PARG27690	-1.806567258	8.893022808	3.70E-11	1.30E-09
PARG24333	1.819554252	6.470551352	3.72E-11	1.30E-09
PARG07972	1.811887609	6.257582483	3.84E-11	1.34E-09
PARG18820	-1.953517439	5.868253779	4.10E-11	1.42E-09
PARG00462	-1.873963294	6.405961924	4.19E-11	1.45E-09
PARG12510	-1.947724691	4.640462503	4.45E-11	1.54E-09
PARG19909	-1.877455271	4.765212932	4.48E-11	1.55E-09
PARG19395	-2.320899756	5.81040785	4.90E-11	1.69E-09
PARG24542	-1.924164509	5.411694553	5.12E-11	1.76E-09

PARG01533	-1.963665468	6.254483681	5.53E-11	1.90E-09
PARG08431	-1.928050439	4.85660415	5.93E-11	2.03E-09
PARG23537	-2.796395199	4.535288041	6.11E-11	2.08E-09
PARG20502	-6.832884332	0.613190988	6.29E-11	2.14E-09
PARG14743	-4.137800051	4.493037737	6.72E-11	2.28E-09
PARG14167	-2.081759628	4.299767046	7.29E-11	2.47E-09
PARG01826	-1.830298424	5.660766128	7.34E-11	2.48E-09
PARG20017	-2.109581419	4.309961999	8.46E-11	2.86E-09
PARG04146	-1.978865709	4.889710115	8.54E-11	2.88E-09
PARG13647	1.802582064	5.26428737	8.63E-11	2.90E-09
PARG25199	-1.889168286	4.323698507	9.69E-11	3.25E-09
PARG19294	-2.774013572	1.344456003	9.83E-11	3.29E-09
PARG07105	1.997046831	1.685493989	1.01E-10	3.38E-09
PARG21000	1.817151228	4.251676554	1.01E-10	3.38E-09
PARG00128	-1.909900452	4.422246637	1.04E-10	3.46E-09
PARG23512	-3.433374427	4.764096527	1.04E-10	3.46E-09
PARG09175	-3.9225281	-0.235794316	1.09E-10	3.61E-09
PARG23846	2.017661216	6.565365764	1.12E-10	3.70E-09
PARG12486	-2.39002231	6.735443472	1.15E-10	3.78E-09
PARG22734	-2.044484225	4.311874932	1.23E-10	4.06E-09
PARG26917	-1.922146271	8.051723783	1.25E-10	4.11E-09
PARG06681	3.607120486	-0.735145425	1.29E-10	4.22E-09
PARG12441	-2.473080302	2.006995125	1.31E-10	4.30E-09
PARG03816	-1.764240974	6.449099955	1.38E-10	4.51E-09
PARG08278	-1.884599391	4.780087817	1.39E-10	4.51E-09
PARG18434	-3.413961956	4.635326802	1.41E-10	4.58E-09
PARG03752	3.087789108	1.054102395	1.42E-10	4.60E-09
PARG00604	2.954858667	0.069483694	1.42E-10	4.60E-09
PARG18152	-2.068079874	4.174463877	1.44E-10	4.64E-09
PARG06093	-1.939301215	7.023772119	1.53E-10	4.92E-09
PARG16089	-2.306460058	3.18797538	1.55E-10	5.00E-09
PARG26306	-1.956276434	4.686195788	1.58E-10	5.05E-09
PARG28152	-1.746965569	6.801679319	1.58E-10	5.05E-09
PARG23595	1.738814113	8.063690392	1.68E-10	5.36E-09
PARG19523	-1.841203625	4.765340578	1.74E-10	5.54E-09
PARG05066	-1.94405151	4.172085709	1.75E-10	5.56E-09
PARG03636	-2.250396062	1.686777877	1.79E-10	5.70E-09
PARG06793	-1.9311864	4.033604756	1.87E-10	5.93E-09
PARG19162	-1.91875547	5.487798268	1.88E-10	5.94E-09
PARG23901	-3.25713633	5.026243499	1.93E-10	6.10E-09
PARG22600	-2.673775885	3.142198831	2.00E-10	6.31E-09
PARG22542	-1.790380541	4.584814588	2.02E-10	6.36E-09
PARG28329	1.748460619	5.58845526	2.03E-10	6.38E-09
PARG13270	-1.939960145	4.772484993	2.10E-10	6.58E-09
PARG27185	-2.895600747	2.005439064	2.12E-10	6.62E-09
PARG18451	-2.113659663	6.317650533	2.28E-10	7.11E-09

PARG00605	2.879174343	0.058848587	2.36E-10	7.34E-09
PARG06923	-2.964977956	2.394908991	2.46E-10	7.65E-09
PARG26369	-1.951795025	3.054133969	2.64E-10	8.18E-09
PARG13593	-2.344742662	3.979217176	2.84E-10	8.79E-09
PARG24610	-2.203068707	3.097665373	3.01E-10	9.30E-09
PARG08293	-1.943619054	4.594092101	3.12E-10	9.63E-09
PARG29823	2.018492926	2.453547577	3.15E-10	9.71E-09
PARG19321	1.840861078	3.736181415	3.30E-10	1.01E-08
PARG06769	-2.126754929	1.443760616	3.43E-10	1.05E-08
PARG00045	1.741806422	6.00730287	3.45E-10	1.06E-08
PARG06357	1.761150868	4.590514401	3.52E-10	1.07E-08
PARG03210	1.763196918	4.671063297	3.56E-10	1.09E-08
PARG12805	-2.021061963	4.428902698	3.62E-10	1.10E-08
PARG19307	-1.915121757	5.903198804	3.73E-10	1.13E-08
PARG02314	1.853331654	3.406811144	4.02E-10	1.22E-08
PARG24001	-1.877714555	3.001883881	4.03E-10	1.22E-08
PARG20309	6.721122288	-0.785004186	4.09E-10	1.23E-08
PARG05199	-2.756161967	2.880623123	4.51E-10	1.36E-08
PARG07605	-2.503270652	1.999272494	4.62E-10	1.39E-08
PARG15040	-2.57256294	3.067985221	4.64E-10	1.39E-08
PARG02749	2.542110217	1.280207443	5.14E-10	1.54E-08
PARG10663	-2.966255199	1.324187587	5.27E-10	1.57E-08
PARG08997	-2.048217617	0.586975555	5.46E-10	1.63E-08
PARG12895	-3.725551354	0.323713678	5.52E-10	1.64E-08
PARG21234	1.756413996	5.582164861	5.65E-10	1.68E-08
PARG05506	-2.61132101	4.536789397	5.74E-10	1.70E-08
PARG26833	1.714842253	6.712738012	5.94E-10	1.76E-08
PARG28226	-3.632544621	1.612181286	6.18E-10	1.83E-08
PARG02714	-1.698177821	6.897890452	6.42E-10	1.89E-08
PARG13973	-2.090162085	2.720863613	6.69E-10	1.97E-08
PARG13106	1.793772018	4.140601887	6.95E-10	2.04E-08
PARG23767	-2.498579455	1.635213012	6.98E-10	2.05E-08
PARG27801	-2.68239222	4.389644001	7.03E-10	2.06E-08
PARG03089	1.741852257	5.260945095	7.04E-10	2.06E-08
PARG05866	-2.181949117	3.36224936	7.15E-10	2.09E-08
PARG16108	-1.713725324	5.020487222	7.58E-10	2.21E-08
PARG11545	-2.263526487	2.248083003	7.67E-10	2.23E-08
PARG12519	1.719022469	4.431019247	7.72E-10	2.24E-08
PARG06472	-2.569282915	4.286399195	7.81E-10	2.26E-08
PARG05825	-3.711792083	3.741991444	7.98E-10	2.31E-08
PARG07913	-2.256158892	2.426016415	8.13E-10	2.35E-08
PARG22059	1.850408276	2.243574187	8.43E-10	2.43E-08
PARG12064	2.116312778	1.441771991	8.81E-10	2.53E-08
PARG13102	-2.143248481	2.828008481	9.05E-10	2.60E-08
PARG28188	-3.476445627	1.435393515	9.14E-10	2.62E-08
PARG24986	-2.014733536	2.282410561	9.55E-10	2.73E-08

PARG29241	-1.975569452	3.30026678	9.56E-10	2.73E-08
PARG15793	-1.724536376	5.737585667	9.79E-10	2.79E-08
PARG29554	-1.854660053	3.913066285	1.02E-09	2.89E-08
PARG20630	-1.96305681	6.631439541	1.04E-09	2.96E-08
PARG22876	-1.988170229	3.789827042	1.05E-09	2.97E-08
PARG00585	1.723440562	4.705825551	1.07E-09	3.02E-08
PARG21693	1.680104663	4.617686888	1.13E-09	3.20E-08
PARG22450	-2.022588332	1.874235576	1.21E-09	3.42E-08
PARG21167	-2.691218632	3.528964651	1.26E-09	3.54E-08
PARG04134	1.736238198	5.223573305	1.27E-09	3.56E-08
PARG04537	-1.834261078	4.44566917	1.27E-09	3.57E-08
PARG11859	-1.877675255	5.225751512	1.32E-09	3.69E-08
PARG21217	-1.742842947	5.001337209	1.34E-09	3.75E-08
PARG07894	-1.689082024	5.569165315	1.35E-09	3.76E-08
PARG10064	-1.650320203	8.411019503	1.40E-09	3.91E-08
PARG24357	-1.665301687	5.635816497	1.43E-09	3.98E-08
PARG19347	1.63840853	7.607759947	1.53E-09	4.24E-08
PARG20253	-1.693353711	5.927147956	1.54E-09	4.25E-08
PARG25040	-1.673600048	6.408006482	1.56E-09	4.30E-08
PARG00556	-1.780089991	4.846798477	1.61E-09	4.45E-08
PARG23594	1.639403607	6.797253323	1.65E-09	4.54E-08
PARG25375	-4.929185698	0.760183897	1.65E-09	4.54E-08
PARG28429	-3.638639779	1.673768065	1.68E-09	4.61E-08
PARG28526	-4.281612681	1.489079424	1.69E-09	4.63E-08
PARG19183	1.630837342	10.14716196	1.71E-09	4.68E-08
PARG21785	-6.581974662	0.150776838	1.73E-09	4.73E-08
PARG07151	1.631332943	6.9946545	1.87E-09	5.09E-08
PARG13346	-1.831388284	4.243738854	1.95E-09	5.30E-08
PARG24998	-1.686169006	5.440505721	2.03E-09	5.52E-08
PARG02475	-2.8585724	2.31574951	2.05E-09	5.54E-08
PARG27932	2.840244711	-0.08201327	2.05E-09	5.54E-08
PARG24943	-1.678101721	4.892019064	2.13E-09	5.75E-08
PARG24393	-1.727170151	5.630702885	2.16E-09	5.83E-08
PARG25790	-2.035510293	3.626623187	2.21E-09	5.95E-08
PARG27820	2.548410726	2.688810909	2.36E-09	6.34E-08
PARG27646	1.729229629	3.796590972	2.42E-09	6.51E-08
PARG13671	-2.154848104	2.579204668	2.50E-09	6.69E-08
PARG23969	-3.518697739	2.316396417	2.53E-09	6.78E-08
PARG27939	-1.75500936	3.264813274	2.54E-09	6.79E-08
PARG15291	-4.890144634	2.079541295	2.59E-09	6.90E-08
PARG06592	-2.008407139	3.216802231	2.62E-09	6.97E-08
PARG12820	2.405009108	0.409929021	2.74E-09	7.29E-08
PARG12001	-1.649513621	7.060363756	2.80E-09	7.44E-08
PARG29460	-6.542603319	-0.148952805	2.83E-09	7.49E-08
PARG21064	-2.27455371	3.356681154	2.86E-09	7.55E-08
PARG24499	1.605106592	11.18431737	2.97E-09	7.83E-08

PARG19222	-2.394405459	6.132473518	3.27E-09	8.61E-08
PARG07190	-1.952590998	1.83632637	3.43E-09	9.01E-08
PARG20580	-2.734677337	3.46212701	3.55E-09	9.31E-08
PARG26344	-3.607219698	2.981523704	3.56E-09	9.32E-08
PARG23719	-3.255965375	2.374725772	3.57E-09	9.35E-08
PARG12399	3.51168367	0.876632294	3.65E-09	9.53E-08
PARG08204	-3.421165481	-0.435352769	3.65E-09	9.53E-08
PARG18093	-2.0008433	2.535360113	3.78E-09	9.83E-08
PARG12613	-1.907361507	3.462670007	3.83E-09	9.94E-08
PARG00162	-2.951202261	0.826916297	3.86E-09	1.00E-07
PARG30008	-4.276579275	-0.107155377	3.92E-09	1.01E-07
PARG12308	-4.229608483	2.427787654	3.92E-09	1.01E-07
PARG29211	1.658724182	5.954379538	4.03E-09	1.04E-07
PARG27002	-2.919012654	5.272522663	4.06E-09	1.05E-07
PARG28483	1.867201881	1.787770322	4.26E-09	1.10E-07
PARG00886	-1.709518956	4.430438742	4.34E-09	1.12E-07
PARG00917	-2.427627803	3.585172331	4.42E-09	1.13E-07
PARG07971	1.661559288	4.099312935	4.49E-09	1.15E-07
PARG03437	2.700522143	0.791094447	4.54E-09	1.16E-07
PARG10469	1.692299018	3.254099872	4.55E-09	1.16E-07
PARG16977	2.347261042	0.20809962	4.64E-09	1.18E-07
PARG15377	-1.68202356	4.982703791	4.72E-09	1.20E-07
PARG03831	1.596576092	5.475141842	4.75E-09	1.21E-07
PARG15844	-1.649546601	4.963700414	4.80E-09	1.22E-07
PARG26710	2.181462182	2.541034271	4.98E-09	1.26E-07
PARG19181	-1.615018285	5.679368849	5.11E-09	1.29E-07
PARG13272	-1.60569501	6.426095607	5.26E-09	1.33E-07
PARG08377	-1.729075123	5.50217593	5.36E-09	1.35E-07
PARG20320	-1.703788529	3.654347137	5.65E-09	1.42E-07
PARG24070	-1.575122421	9.724965687	5.77E-09	1.45E-07
PARG11955	-4.189435476	-0.041895697	6.00E-09	1.50E-07
PARG03069	-2.434413666	1.763252194	6.09E-09	1.52E-07
PARG08667	1.575887689	8.459619942	6.12E-09	1.53E-07
PARG07261	-1.653831439	5.625981594	6.14E-09	1.53E-07
PARG27343	1.585659515	6.305277776	6.24E-09	1.56E-07
PARG07726	-1.681608518	4.237677151	6.33E-09	1.57E-07
PARG12160	-2.518413742	2.292527725	6.38E-09	1.59E-07
PARG29470	1.574471343	7.401220255	6.46E-09	1.60E-07
PARG24497	1.582102244	6.713881697	6.75E-09	1.67E-07
PARG25225	1.577097917	5.84433043	6.91E-09	1.71E-07
PARG00597	-1.717592993	2.946272287	6.98E-09	1.72E-07
PARG27828	-1.588174356	6.28255635	6.99E-09	1.72E-07
PARG19026	-3.20615419	1.588290404	7.13E-09	1.75E-07
PARG18567	-3.8217369	0.14423169	7.16E-09	1.76E-07
PARG29421	-2.510871781	4.109783592	7.19E-09	1.76E-07
PARG10844	-1.586787144	6.149281726	7.26E-09	1.78E-07

PARG02916	-2.148013926	2.826252709	7.54E-09	1.84E-07
PARG12455	-1.835569777	3.858458238	7.78E-09	1.90E-07
PARG19189	-1.718007387	2.739874172	7.88E-09	1.92E-07
PARG11902	-2.972924173	-0.41926348	8.12E-09	1.98E-07
PARG00583	1.57813576	5.610414286	8.56E-09	2.08E-07
PARG20818	-1.569536233	6.83525925	8.56E-09	2.08E-07
PARG10386	1.699548225	3.937171612	8.61E-09	2.08E-07
PARG19616	-1.586909808	6.437090834	9.37E-09	2.27E-07
PARG19367	1.55453108	6.440452732	1.05E-08	2.55E-07
PARG11228	1.615072459	4.157769895	1.06E-08	2.57E-07
PARG14234	-1.707015033	3.066980098	1.10E-08	2.65E-07
PARG17395	-1.898443362	4.696038336	1.11E-08	2.66E-07
PARG02095	-2.05918358	5.315126319	1.13E-08	2.71E-07
PARG22901	-1.757362092	8.813600126	1.15E-08	2.75E-07
PARG14232	-1.685610775	2.546765919	1.19E-08	2.84E-07
PARG23266	-1.708854132	4.756334073	1.23E-08	2.93E-07
PARG24008	1.541288637	7.96974132	1.23E-08	2.93E-07
PARG12393	1.56326107	4.89945417	1.26E-08	3.01E-07
PARG26143	1.61366779	3.701314729	1.31E-08	3.12E-07
PARG07217	-1.758818027	3.137452902	1.41E-08	3.34E-07
PARG00392	1.846175138	1.665366331	1.44E-08	3.42E-07
PARG09978	-2.423568573	2.721015384	1.46E-08	3.45E-07
PARG08319	-1.6545176	4.1515669	1.47E-08	3.48E-07
PARG10681	-1.575963369	6.684421644	1.63E-08	3.84E-07
PARG02632	1.937627902	0.940138305	1.68E-08	3.97E-07
PARG28078	-1.825548648	3.755538385	1.69E-08	3.97E-07
PARG27131	-1.969325168	2.46914527	1.72E-08	4.03E-07
PARG00280	1.522327079	9.191145857	1.72E-08	4.03E-07
PARG07518	-2.122711712	5.235779098	1.76E-08	4.12E-07
PARG21139	-1.696626157	5.857641455	1.76E-08	4.12E-07
PARG15359	-1.698672868	2.622011978	1.82E-08	4.26E-07
PARG00443	1.528658772	6.692869067	1.86E-08	4.34E-07
PARG18520	-1.629104991	5.140086901	1.97E-08	4.59E-07
PARG23930	-1.72988724	4.492843366	1.98E-08	4.61E-07
PARG30015	1.52528347	6.548153575	2.00E-08	4.64E-07
PARG11727	1.550821527	5.015060439	2.00E-08	4.65E-07
PARG23732	-3.364107537	2.719157053	2.03E-08	4.70E-07
PARG19452	-4.097466837	0.576489829	2.18E-08	5.04E-07
PARG27455	-1.859013819	3.526591479	2.23E-08	5.16E-07
PARG15458	1.829667398	0.794127406	2.31E-08	5.32E-07
PARG20591	-1.98248405	2.266601554	2.32E-08	5.34E-07
PARG19328	1.523823112	5.0918006	2.33E-08	5.35E-07
PARG16276	-1.516348113	10.40824656	2.34E-08	5.36E-07
PARG12753	-2.036673374	3.580855558	2.37E-08	5.44E-07
PARG26698	-1.78969779	5.787191315	2.44E-08	5.58E-07
PARG26431	1.564206164	2.872187525	2.45E-08	5.60E-07

PARG22792	-2.534888716	1.371013872	2.54E-08	5.80E-07
PARG18426	1.502507411	9.974123612	2.55E-08	5.81E-07
PARG20872	-4.694747599	-0.242754658	2.57E-08	5.86E-07
PARG29462	-1.675655819	4.132931679	2.71E-08	6.16E-07
PARG17761	-2.864145321	1.335752326	2.77E-08	6.29E-07
PARG14535	-1.814249375	2.861584008	2.85E-08	6.46E-07
PARG19972	2.011111038	1.563947544	2.86E-08	6.47E-07
PARG24627	-2.308066902	3.02432379	2.87E-08	6.48E-07
PARG04989	-2.107801963	3.233956679	2.92E-08	6.58E-07
PARG24683	-1.502740216	7.82064234	2.92E-08	6.58E-07
PARG23791	-1.992379564	3.272229346	3.07E-08	6.90E-07
PARG17685	-1.635436964	3.957215019	3.11E-08	6.99E-07
PARG13887	-2.733447105	-0.095778216	3.31E-08	7.42E-07
PARG28005	1.6633152	2.210333757	3.33E-08	7.46E-07
PARG09186	-1.88119096	3.54778098	3.37E-08	7.54E-07
PARG04483	-1.670840849	2.396441327	3.43E-08	7.65E-07
PARG17970	6.402520134	-0.576027408	3.48E-08	7.77E-07
PARG07978	1.497696638	6.180508219	3.53E-08	7.87E-07
PARG13088	-3.41537603	3.759731034	3.55E-08	7.90E-07
PARG02155	-3.675827591	2.523762885	3.65E-08	8.09E-07
PARG03693	-1.502419882	4.860707651	4.01E-08	8.89E-07
PARG07310	-3.044482209	0.783747861	4.08E-08	9.04E-07
PARG19438	-1.594869949	5.298199383	4.09E-08	9.05E-07
PARG23747	-3.107516058	2.397598837	4.11E-08	9.08E-07
PARG11550	-1.648375812	4.178617172	4.13E-08	9.11E-07
PARG15558	-1.480907032	9.153732941	4.22E-08	9.29E-07
PARG07273	1.481218643	7.213984715	4.28E-08	9.40E-07
PARG22931	1.810867581	1.193867593	4.29E-08	9.42E-07
PARG07356	2.246874189	0.074715757	4.42E-08	9.70E-07
PARG07453	-3.203600534	3.358348611	4.73E-08	1.04E-06
PARG16593	1.695145914	1.837547075	4.78E-08	1.04E-06
PARG19908	1.546547451	3.826929939	4.80E-08	1.05E-06
PARG00851	-1.831077453	4.459604138	4.81E-08	1.05E-06
PARG06630	-1.480721319	8.21637871	4.81E-08	1.05E-06
PARG07408	1.518726554	5.015883404	4.88E-08	1.06E-06
PARG12569	-2.691176933	0.554373374	5.08E-08	1.10E-06
PARG26285	-1.850432332	3.086993739	5.10E-08	1.10E-06
PARG01084	1.476168207	6.424421706	5.19E-08	1.12E-06
PARG09208	1.479647369	6.063882241	5.47E-08	1.18E-06
PARG07800	-2.619955918	3.098489565	5.49E-08	1.18E-06
PARG10507	-2.34518818	2.318116661	5.49E-08	1.18E-06
PARG26813	-1.677505404	3.594876676	5.60E-08	1.21E-06
PARG01741	-2.669378141	1.993151988	5.83E-08	1.25E-06
PARG05643	-6.280521936	1.037587275	5.83E-08	1.25E-06
PARG00952	1.601089334	2.100172598	5.93E-08	1.27E-06
PARG16065	-1.484678574	6.349493735	5.95E-08	1.27E-06

PARG22115	-1.563125975	5.096674311	6.13E-08	1.31E-06
PARG24846	-1.46186785	8.809242266	6.14E-08	1.31E-06
PARG06759	-1.672253373	3.062877431	6.17E-08	1.32E-06
PARG13496	-1.637364623	4.40480932	6.27E-08	1.34E-06
PARG11582	-2.181312545	4.73917078	6.29E-08	1.34E-06
PARG21030	1.548223948	3.095491008	6.57E-08	1.40E-06
PARG01590	-1.483488323	5.948336339	6.70E-08	1.42E-06
PARG26488	1.497887387	5.419400446	6.98E-08	1.48E-06
PARG06586	1.802713915	1.451474988	7.08E-08	1.50E-06
PARG08398	-1.533474269	5.376494728	7.15E-08	1.51E-06
PARG09182	-1.702874147	4.407941332	7.23E-08	1.53E-06
PARG18382	1.46002713	5.932420614	7.62E-08	1.60E-06
PARG28555	-1.513626161	6.617118557	7.63E-08	1.60E-06
PARG12886	-3.369461937	0.123604726	7.78E-08	1.63E-06
PARG07302	-1.513106758	5.438863283	7.81E-08	1.64E-06
PARG11388	1.488833633	4.526182455	7.81E-08	1.64E-06
PARG02713	-1.673714498	5.416226458	7.89E-08	1.65E-06
PARG02249	-3.955459567	-0.329239744	8.13E-08	1.70E-06
PARG23701	-1.529659068	4.138031836	8.17E-08	1.70E-06
PARG02667	1.523916225	3.321488538	8.19E-08	1.71E-06
PARG12782	-1.488509194	7.050052573	8.47E-08	1.76E-06
PARG25305	-1.73717807	6.180139941	8.53E-08	1.77E-06
PARG20498	2.027418653	2.798011256	8.55E-08	1.78E-06
PARG06683	-2.326443403	5.145138417	8.68E-08	1.80E-06
PARG11224	-2.069990443	4.75480434	8.75E-08	1.81E-06
PARG27814	-1.499847129	4.957457569	8.96E-08	1.85E-06
PARG02700	-1.501960878	4.863477351	9.18E-08	1.90E-06
PARG15422	-1.51696037	4.345519699	9.53E-08	1.97E-06
PARG10573	-2.154307841	1.467920841	9.59E-08	1.98E-06
PARG04772	3.241439514	0.073308805	9.98E-08	2.05E-06
PARG22865	1.480245088	4.416496096	1.01E-07	2.08E-06
PARG17121	-1.777255182	2.850922321	1.04E-07	2.13E-06
PARG21503	1.466025122	5.460458158	1.05E-07	2.15E-06
PARG19105	-1.724565819	2.743317911	1.05E-07	2.15E-06
PARG10014	-1.457917245	6.153326522	1.07E-07	2.19E-06
PARG27039	-2.283144057	3.64195293	1.09E-07	2.22E-06
PARG13529	-1.629175432	9.275087172	1.09E-07	2.23E-06
PARG00027	-1.799815879	4.868051822	1.16E-07	2.36E-06
PARG26787	-1.460015223	5.217580323	1.17E-07	2.37E-06
PARG12003	-1.498088899	5.482295327	1.17E-07	2.38E-06
PARG25851	-2.705671585	1.412216157	1.19E-07	2.41E-06
PARG17987	-2.09330799	1.776971358	1.23E-07	2.49E-06
PARG15888	-1.462627407	4.972733449	1.24E-07	2.51E-06
PARG18666	-1.644064084	4.329006805	1.25E-07	2.52E-06
PARG16245	-1.609103557	2.9594722	1.29E-07	2.60E-06
PARG21025	1.742058593	1.026803772	1.30E-07	2.62E-06

PARG20430	-1.709211617	4.936655127	1.34E-07	2.69E-06
PARG12295	-1.603676669	3.551923873	1.34E-07	2.69E-06
PARG28471	-1.498774914	5.620249186	1.35E-07	2.70E-06
PARG21816	1.792548796	0.750349556	1.35E-07	2.72E-06
PARG19047	-2.266782029	4.587247797	1.37E-07	2.73E-06
PARG08668	2.252536412	0.623517671	1.37E-07	2.73E-06
PARG19136	-3.079659952	1.286306669	1.37E-07	2.74E-06
PARG02089	-2.428151204	1.014111089	1.40E-07	2.80E-06
PARG18354	-2.356756124	3.054778299	1.43E-07	2.85E-06
PARG27597	-1.438219387	5.958429962	1.47E-07	2.93E-06
PARG23942	2.134111213	1.615392443	1.51E-07	2.99E-06
PARG12577	-2.786894501	3.041548198	1.52E-07	3.02E-06
PARG29459	-1.667280041	4.819510286	1.52E-07	3.02E-06
PARG07172	-1.437113679	7.544425616	1.59E-07	3.16E-06
PARG24835	1.72922039	2.14952939	1.60E-07	3.16E-06
PARG07832	-1.507294334	2.677517999	1.62E-07	3.20E-06
PARG12382	1.455709168	4.721824987	1.62E-07	3.20E-06
PARG05044	6.154932666	-1.186552219	1.66E-07	3.26E-06
PARG25204	1.511466761	3.618573399	1.66E-07	3.27E-06
PARG19678	-1.721347523	2.771592717	1.70E-07	3.35E-06
PARG15573	-1.594477642	4.922628323	1.71E-07	3.35E-06
PARG14467	-1.485116443	4.869535102	1.73E-07	3.39E-06
PARG26578	-2.284744338	4.853115191	1.76E-07	3.44E-06
PARG10875	-1.78300616	6.035576856	1.77E-07	3.47E-06
PARG08405	-1.461906454	6.650318574	1.79E-07	3.50E-06
PARG16132	-1.426339407	6.582314144	1.82E-07	3.55E-06
PARG27284	-3.028867032	0.437571465	1.94E-07	3.78E-06
PARG20822	1.546944994	1.932415879	1.96E-07	3.81E-06
PARG18477	-3.879684046	-0.369576237	1.98E-07	3.85E-06
PARG27584	-1.727679426	4.266193104	2.07E-07	4.01E-06
PARG11332	1.417047015	5.337327257	2.08E-07	4.02E-06
PARG10998	-1.506616976	3.295450132	2.08E-07	4.03E-06
PARG12715	-1.400043596	8.100914022	2.08E-07	4.03E-06
PARG18245	-2.040401446	2.535977788	2.09E-07	4.03E-06
PARG00303	1.95019548	1.670652442	2.09E-07	4.03E-06
PARG20548	1.437712454	5.100912287	2.10E-07	4.05E-06
PARG27582	1.395025378	10.12594402	2.16E-07	4.16E-06
PARG03336	1.873538543	3.977255087	2.27E-07	4.35E-06
PARG08629	-1.541339628	2.311690505	2.31E-07	4.44E-06
PARG15759	-1.515863093	3.70922433	2.35E-07	4.51E-06
PARG19659	-1.731474004	3.517590717	2.36E-07	4.52E-06
PARG24557	1.391137991	9.087310999	2.36E-07	4.52E-06
PARG29451	-1.606212859	4.208033329	2.45E-07	4.68E-06
PARG05845	1.395116038	7.343093141	2.46E-07	4.69E-06
PARG05891	1.978709894	2.143620605	2.46E-07	4.69E-06
PARG06348	1.709966812	1.141121741	2.47E-07	4.69E-06

PARG11110	-1.6622645	4.456940963	2.51E-07	4.78E-06
PARG26914	-1.453830702	4.639807128	2.53E-07	4.80E-06
PARG13380	1.389133322	8.144457297	2.53E-07	4.80E-06
PARG10459	1.680907039	0.433868935	2.56E-07	4.85E-06
PARG05444	-1.443325407	5.305046975	2.60E-07	4.93E-06
PARG22194	-1.904305933	1.631861884	2.64E-07	4.98E-06
PARG18979	-1.439449278	7.771244793	2.65E-07	5.00E-06
PARG03546	4.539947867	1.69777314	2.78E-07	5.25E-06
PARG22774	-6.129549249	1.622553487	2.81E-07	5.30E-06
PARG07244	-1.699979963	3.086558447	2.84E-07	5.34E-06
PARG18453	-1.994967779	5.107540447	2.86E-07	5.37E-06
PARG11000	-1.41859783	6.294159901	2.88E-07	5.40E-06
PARG11103	-1.480238026	6.835694752	2.88E-07	5.40E-06
PARG29248	-2.883764042	2.395422464	2.95E-07	5.52E-06
PARG06229	-2.879705349	2.123107986	2.95E-07	5.52E-06
PARG19839	1.456470006	3.465362209	3.00E-07	5.61E-06
PARG09031	-1.379531783	9.738854015	3.06E-07	5.70E-06
PARG11900	-1.421744495	4.564264168	3.10E-07	5.77E-06
PARG00411	-2.776283865	2.720148587	3.10E-07	5.77E-06
PARG28111	-2.602699529	5.492252802	3.25E-07	6.03E-06
PARG06797	-1.506555219	3.813463558	3.26E-07	6.05E-06
PARG06380	-1.39302533	5.670980413	3.26E-07	6.05E-06
PARG06162	-1.58599841	3.381113962	3.28E-07	6.08E-06
PARG24120	1.623014773	1.807822557	3.31E-07	6.11E-06
PARG14178	-1.54790949	2.848205436	3.33E-07	6.16E-06
PARG18944	-1.373201549	9.760034396	3.36E-07	6.19E-06
PARG06910	1.546258165	2.668140592	3.41E-07	6.28E-06
PARG18358	-1.485710073	6.779770584	3.45E-07	6.35E-06
PARG19173	1.389369849	5.2088349	3.54E-07	6.51E-06
PARG22812	-1.491664006	4.882536726	3.67E-07	6.74E-06
PARG27667	-1.882613531	3.659686546	3.73E-07	6.84E-06
PARG26934	2.86708764	0.336125711	3.88E-07	7.11E-06
PARG20400	-1.813722201	3.335575082	3.92E-07	7.18E-06
PARG00200	-1.865875102	4.087466896	3.97E-07	7.25E-06
PARG14835	-2.008718818	2.946491957	4.01E-07	7.32E-06
PARG02778	1.57367354	2.170261653	4.05E-07	7.39E-06
PARG12826	-1.79853613	1.915144965	4.06E-07	7.40E-06
PARG11529	-2.345019218	-0.061386923	4.10E-07	7.45E-06
PARG24618	2.121057042	2.976709201	4.14E-07	7.52E-06
PARG07640	-1.379594656	6.665827065	4.26E-07	7.73E-06
PARG03974	-2.419089647	5.248924146	4.29E-07	7.78E-06
PARG08621	-2.522041452	3.3887544	4.36E-07	7.89E-06
PARG29929	-1.390992545	6.363926469	4.42E-07	7.99E-06
PARG02720	1.369295084	6.617061315	4.45E-07	8.05E-06
PARG06761	-3.471045427	0.193295606	4.49E-07	8.10E-06
PARG05778	-1.37620124	7.122068845	4.51E-07	8.12E-06

PARG06732	-2.255462158	1.215486889	4.53E-07	8.15E-06
PARG19406	-3.042584016	-0.322191197	4.54E-07	8.16E-06
PARG02443	1.77836144	3.380773188	4.57E-07	8.21E-06
PARG26739	-1.504226215	3.274880452	4.73E-07	8.48E-06
PARG08536	-1.792841232	2.578504183	4.76E-07	8.53E-06
PARG23461	-1.545500723	3.583565211	4.80E-07	8.59E-06
PARG00143	-1.48610666	4.656032267	4.81E-07	8.59E-06
PARG05528	1.35332213	9.722505709	4.81E-07	8.59E-06
PARG28475	-4.150338361	0.023033957	4.89E-07	8.72E-06
PARG07150	1.387853141	5.189614086	4.91E-07	8.75E-06
PARG12331	1.362370915	6.080597087	4.94E-07	8.80E-06
PARG09484	-1.397245024	5.339806233	4.97E-07	8.83E-06
PARG16073	1.980952432	1.018891242	5.06E-07	8.99E-06
PARG10624	1.367133556	5.123110817	5.09E-07	9.03E-06
PARG15235	1.388855821	5.998852192	5.27E-07	9.34E-06
PARG27603	-1.716296234	3.703935002	5.30E-07	9.37E-06
PARG27241	1.357097042	6.581682035	5.32E-07	9.41E-06
PARG23554	-1.382745775	5.854737785	5.58E-07	9.85E-06
PARG00507	-2.258126345	3.338869436	5.75E-07	1.01E-05
PARG23478	-1.403561872	5.31446015	5.84E-07	1.03E-05
PARG19798	-1.53010646	3.06722351	5.87E-07	1.03E-05
PARG18753	-1.507618658	3.806712665	5.92E-07	1.04E-05
PARG06751	1.426519907	2.909392396	6.12E-07	1.08E-05
PARG19979	2.018368064	0.806278649	6.15E-07	1.08E-05
PARG19314	-1.917672734	2.752440184	6.16E-07	1.08E-05
PARG27497	1.344595082	6.84570749	6.20E-07	1.08E-05
PARG12210	1.35077072	6.317377973	6.39E-07	1.12E-05
PARG02378	-2.122734779	1.261779493	6.51E-07	1.14E-05
PARG08362	-1.345578817	6.650621278	6.64E-07	1.16E-05
PARG28375	2.239269391	0.143663199	6.65E-07	1.16E-05
PARG08923	-1.397454713	6.009411113	6.72E-07	1.17E-05
PARG29951	-3.447684445	3.323083703	6.88E-07	1.19E-05
PARG16047	-3.443301519	2.698666202	6.88E-07	1.19E-05
PARG13942	-1.719498999	2.3062654	6.89E-07	1.20E-05
PARG16200	1.928489759	0.486815311	6.92E-07	1.20E-05
PARG20113	2.325483968	-0.437166642	6.96E-07	1.20E-05
PARG16092	-1.37158375	5.313127239	7.17E-07	1.24E-05
PARG00291	-1.427614021	3.130457507	7.19E-07	1.24E-05
PARG05694	-4.396678283	-0.509709057	7.40E-07	1.28E-05
PARG02459	-1.488819616	4.695324743	7.44E-07	1.28E-05
PARG13921	-1.689618448	1.555308936	7.49E-07	1.29E-05
PARG10847	-1.334232618	8.209539529	7.58E-07	1.30E-05
PARG06562	-1.347446594	5.457043194	7.61E-07	1.31E-05
PARG22832	-2.076365328	4.920380521	7.67E-07	1.32E-05
PARG01190	-3.745406848	3.543673658	7.71E-07	1.32E-05
PARG04026	-1.34506206	7.941702999	7.75E-07	1.33E-05

PARG17150	-2.831854979	3.773216656	8.06E-07	1.38E-05
PARG02343	-1.75484618	3.646572664	8.15E-07	1.39E-05
PARG13869	1.636186656	1.852096757	8.19E-07	1.40E-05
PARG13638	-6.01748919	4.679933228	8.23E-07	1.40E-05
PARG18583	-6.003409282	0.538403442	8.23E-07	1.40E-05
PARG12128	-1.464816736	5.236736497	8.35E-07	1.42E-05
PARG27824	-1.740805537	3.230288551	8.35E-07	1.42E-05
PARG16535	-1.756929782	3.377114092	8.53E-07	1.45E-05
PARG00969	1.351585786	5.048874149	8.58E-07	1.45E-05
PARG10490	1.397568702	3.199536552	8.71E-07	1.47E-05
PARG12480	-1.351475931	4.866533758	8.84E-07	1.49E-05
PARG23275	-1.838507839	3.512303021	8.84E-07	1.49E-05
PARG13395	1.323404437	6.993960092	8.98E-07	1.52E-05
PARG20067	1.319589333	8.087149472	9.24E-07	1.56E-05
PARG27259	1.322521006	6.64319216	9.36E-07	1.58E-05
PARG16757	1.332076176	6.903565273	9.39E-07	1.58E-05
PARG28181	1.621140064	3.297524396	9.41E-07	1.58E-05
PARG28029	-2.344424686	3.327969852	9.54E-07	1.60E-05
PARG08434	-1.623453182	2.735144522	1.00E-06	1.68E-05
PARG22780	-1.355121964	5.390959264	1.00E-06	1.68E-05
PARG06911	1.313150745	7.274821809	1.06E-06	1.78E-05
PARG08584	-1.318473588	6.42181399	1.09E-06	1.82E-05
PARG02048	-1.329721941	5.255398337	1.10E-06	1.83E-05
PARG21066	1.483530547	1.827345954	1.14E-06	1.91E-05
PARG02075	1.391750347	4.018149672	1.14E-06	1.91E-05
PARG09463	1.48769292	2.694560162	1.15E-06	1.91E-05
PARG21136	-1.526152308	3.197227349	1.16E-06	1.93E-05
PARG04069	-1.382603845	3.286864706	1.16E-06	1.93E-05
PARG15647	-2.12616603	2.077537232	1.18E-06	1.95E-05
PARG17765	-4.348726395	0.540703701	1.21E-06	2.01E-05
PARG10313	-3.765494815	1.649867606	1.22E-06	2.02E-05
PARG13154	-3.709311394	0.763909856	1.22E-06	2.02E-05
PARG20176	1.305005825	7.73471866	1.22E-06	2.02E-05
PARG08611	-1.476445391	5.323173632	1.22E-06	2.02E-05
PARG07417	-1.310041333	6.254737116	1.23E-06	2.02E-05
PARG24602	1.447872978	1.579099982	1.25E-06	2.06E-05
PARG20118	-1.303561107	7.933826069	1.26E-06	2.08E-05
PARG12357	-1.382740225	3.853193727	1.29E-06	2.12E-05
PARG03136	-1.471445417	4.277497821	1.32E-06	2.17E-05
PARG04912	-1.411783232	4.640779582	1.35E-06	2.21E-05
PARG02195	-1.301642839	7.298788522	1.38E-06	2.26E-05
PARG18618	-1.980648489	3.706026946	1.42E-06	2.33E-05
PARG09972	1.312965606	4.777664314	1.43E-06	2.33E-05
PARG29296	-2.953141083	2.163915716	1.44E-06	2.35E-05
PARG19635	-1.369852805	3.78599935	1.44E-06	2.35E-05
PARG02097	-2.426046428	5.588016367	1.52E-06	2.47E-05

PARG21662	-1.309110109	5.924235416	1.55E-06	2.52E-05
PARG23731	-2.840612651	1.360500005	1.58E-06	2.56E-05
PARG05252	1.302385419	6.031977068	1.58E-06	2.57E-05
PARG00627	-2.752441302	1.054845156	1.58E-06	2.57E-05
PARG01376	-1.331789547	6.706338755	1.60E-06	2.59E-05
PARG20367	-3.330562907	0.317389429	1.62E-06	2.63E-05
PARG24967	-1.510478197	3.183106327	1.63E-06	2.63E-05
PARG16067	-1.303035665	6.988043476	1.63E-06	2.64E-05
PARG00057	-1.487730309	2.605128315	1.78E-06	2.88E-05
PARG24574	-1.765324117	2.3586664	1.79E-06	2.88E-05
PARG27984	-1.310392117	5.874133079	1.84E-06	2.97E-05
PARG16846	-2.016836468	1.05121365	1.87E-06	3.01E-05
PARG17710	-1.288485654	5.836481046	1.93E-06	3.10E-05
PARG22978	-1.291635127	7.533114878	1.93E-06	3.10E-05
PARG13792	-2.453851778	2.104500706	1.98E-06	3.17E-05
PARG16822	-2.355956534	1.052136493	1.98E-06	3.17E-05
PARG18026	-1.295871409	8.70631205	1.98E-06	3.18E-05
PARG29487	-1.342882365	5.485315519	1.99E-06	3.18E-05
PARG03592	-4.301485608	-0.220362343	1.99E-06	3.18E-05
PARG26561	-4.284150573	1.951687293	1.99E-06	3.18E-05
PARG01475	-4.262443304	0.683861319	1.99E-06	3.18E-05
PARG29576	1.297673651	4.897857139	2.01E-06	3.20E-05
PARG00282	-1.295513829	6.728061898	2.03E-06	3.23E-05
PARG11817	1.357511021	5.180134471	2.08E-06	3.31E-05
PARG10751	-1.69434951	3.45223366	2.09E-06	3.32E-05
PARG29262	1.692366017	1.627724762	2.09E-06	3.32E-05
PARG06516	-1.553624137	3.565424823	2.12E-06	3.36E-05
PARG30033	-1.993686347	2.952789568	2.14E-06	3.39E-05
PARG08024	-1.4874659	4.0373201	2.17E-06	3.43E-05
PARG15534	-1.593826245	5.450859691	2.17E-06	3.44E-05
PARG13619	1.450271429	1.743045126	2.22E-06	3.50E-05
PARG15657	1.282859994	6.496899995	2.24E-06	3.54E-05
PARG07062	1.276443778	7.535817997	2.26E-06	3.56E-05
PARG12451	-1.54022974	9.229520181	2.40E-06	3.77E-05
PARG19887	-2.113326241	2.926556765	2.41E-06	3.80E-05
PARG19770	-1.301882559	5.366792931	2.42E-06	3.80E-05
PARG12986	-1.387621802	5.032523941	2.49E-06	3.91E-05
PARG27166	1.889442066	2.214163724	2.51E-06	3.93E-05
PARG15360	-1.368848121	4.521031548	2.54E-06	3.99E-05
PARG12804	-1.270864376	6.167062252	2.58E-06	4.04E-05
PARG28203	-1.399681502	3.630360047	2.61E-06	4.08E-05
PARG05886	1.454449444	2.28484399	2.65E-06	4.14E-05
PARG07922	-1.261373593	7.519372024	2.69E-06	4.20E-05
PARG02078	-1.319737444	5.418663104	2.71E-06	4.22E-05
PARG20536	1.259661932	8.378837143	2.71E-06	4.22E-05
PARG15218	-1.461530016	4.473119159	2.75E-06	4.28E-05

PARG18830	-1.26997546	6.847328549	2.76E-06	4.29E-05
PARG16004	-1.434384924	3.051979188	2.78E-06	4.31E-05
PARG00168	1.287399412	3.958563399	2.82E-06	4.38E-05
PARG15803	-2.275933778	2.718165454	2.86E-06	4.44E-05
PARG06424	-1.271089922	6.443267976	2.90E-06	4.49E-05
PARG02780	1.258027789	7.01106058	2.94E-06	4.55E-05
PARG04643	-1.495489156	4.17652086	3.01E-06	4.65E-05
PARG14428	-1.446720093	6.030457351	3.01E-06	4.65E-05
PARG08592	-1.286317611	5.449002369	3.02E-06	4.65E-05
PARG01264	-1.325548616	4.311839986	3.05E-06	4.70E-05
PARG27381	1.382751641	2.935916017	3.06E-06	4.70E-05
PARG08422	-3.667655355	4.64642581	3.07E-06	4.72E-05
PARG03461	-3.651847002	2.470437219	3.07E-06	4.72E-05
PARG14084	-1.406779611	9.466798252	3.12E-06	4.79E-05
PARG07757	-1.280881544	5.409029516	3.23E-06	4.95E-05
PARG07990	-1.420948069	4.193266493	3.25E-06	4.97E-05
PARG21156	-1.306372081	4.598317466	3.27E-06	5.00E-05
PARG15732	-1.312282145	4.536232312	3.27E-06	5.00E-05
PARG06193	-4.035207012	0.197756097	3.29E-06	5.02E-05
PARG24351	-1.998724122	2.332969445	3.31E-06	5.06E-05
PARG10935	-2.083823736	2.344758102	3.37E-06	5.13E-05
PARG19483	2.075283668	0.119217406	3.37E-06	5.13E-05
PARG16665	-1.438874989	3.926347675	3.38E-06	5.14E-05
PARG03109	1.295990649	4.385657973	3.43E-06	5.21E-05
PARG24609	1.260588983	4.934882515	3.44E-06	5.23E-05
PARG21350	-1.262002887	7.350500216	3.49E-06	5.30E-05
PARG20140	-1.289743751	5.201762731	3.60E-06	5.45E-05
PARG24142	1.242709733	8.891977445	3.62E-06	5.48E-05
PARG04424	-2.391734889	3.473599978	3.63E-06	5.49E-05
PARG04184	-1.25326119	7.907135534	3.72E-06	5.62E-05
PARG11890	-1.503885925	3.413064476	3.72E-06	5.62E-05
PARG25239	2.131589262	3.201581325	3.78E-06	5.69E-05
PARG23752	-1.920957617	1.18869179	3.78E-06	5.69E-05
PARG06171	-1.41203373	4.881995302	3.81E-06	5.73E-05
PARG25200	-1.384667315	5.157504887	3.83E-06	5.77E-05
PARG20620	-1.699124157	5.116801211	3.84E-06	5.77E-05
PARG06248	-1.284996075	5.27534785	3.88E-06	5.83E-05
PARG05848	-2.497189547	3.441758835	3.90E-06	5.84E-05
PARG08250	1.244283957	6.534676384	3.98E-06	5.96E-05
PARG16942	-1.247035436	7.557910268	3.99E-06	5.97E-05
PARG02231	1.295663634	4.136228227	4.00E-06	5.98E-05
PARG10479	1.353054105	2.745055191	4.00E-06	5.98E-05
PARG19737	1.481885841	1.606960425	4.02E-06	5.99E-05
PARG28319	-1.980888658	1.412377032	4.04E-06	6.02E-05
PARG01914	1.251635505	4.794801611	4.12E-06	6.14E-05
PARG24419	-1.804831963	1.992789114	4.13E-06	6.14E-05

PARG15530	-1.336135509	5.799323947	4.13E-06	6.14E-05
PARG26752	-1.293988269	5.650223982	4.16E-06	6.18E-05
PARG19986	1.575414544	1.85498241	4.26E-06	6.32E-05
PARG14943	-5.857168013	0.831215072	4.27E-06	6.32E-05
PARG06760	-5.855293033	1.750035947	4.27E-06	6.32E-05
PARG29076	-5.842803343	-0.45446703	4.27E-06	6.32E-05
PARG21794	-1.250941968	7.683691052	4.28E-06	6.33E-05
PARG04316	1.274884135	3.30301582	4.31E-06	6.37E-05
PARG14177	2.05820598	-0.093310382	4.32E-06	6.37E-05
PARG20183	-1.339087728	5.271388416	4.51E-06	6.65E-05
PARG16203	2.231719385	0.211180567	4.51E-06	6.65E-05
PARG15718	1.244655174	5.224769152	4.52E-06	6.66E-05
PARG11544	-1.238738395	6.378894946	4.84E-06	7.12E-05
PARG08251	-2.297659102	1.387247557	4.92E-06	7.23E-05
PARG27581	1.224988315	8.25738808	4.93E-06	7.23E-05
PARG13682	-1.722404677	2.340700171	5.00E-06	7.34E-05
PARG27474	-1.331537607	3.67928688	5.08E-06	7.44E-05
PARG14416	-2.146644814	4.069459873	5.31E-06	7.76E-05
PARG16689	-1.236016504	7.408399036	5.31E-06	7.76E-05
PARG07745	1.224735766	5.710948452	5.41E-06	7.90E-05
PARG29242	-4.169991837	-0.396713937	5.45E-06	7.95E-05
PARG27371	-1.470890185	2.300288488	5.62E-06	8.19E-05
PARG27701	1.530492736	2.178820469	5.71E-06	8.30E-05
PARG24003	1.224504921	6.112299998	5.71E-06	8.30E-05
PARG10999	-1.239451335	5.063388315	5.71E-06	8.30E-05
PARG12374	-1.894741745	3.261618279	5.72E-06	8.30E-05
PARG12381	-1.937684334	4.876049273	5.73E-06	8.30E-05
PARG14498	-1.932892676	0.234008334	5.73E-06	8.30E-05
PARG16565	1.366290672	1.396470989	5.77E-06	8.36E-05
PARG08277	1.244671616	4.636065086	5.89E-06	8.52E-05
PARG24286	-1.228370826	7.416882863	5.90E-06	8.53E-05
PARG20589	-1.326047192	3.520514049	5.98E-06	8.63E-05
PARG07092	-1.329660651	5.684380449	6.04E-06	8.71E-05
PARG05431	1.221481379	6.343984934	6.06E-06	8.74E-05
PARG24559	1.27863213	4.228174027	6.24E-06	8.99E-05
PARG01238	-1.251854296	4.488580616	6.30E-06	9.06E-05
PARG12443	-1.453971564	3.851072312	6.32E-06	9.08E-05
PARG06881	-1.264003196	4.375273619	6.40E-06	9.20E-05
PARG06128	-1.433846247	4.411043279	6.51E-06	9.34E-05
PARG16308	-2.548108045	1.793698468	6.52E-06	9.34E-05
PARG07606	-1.399512091	6.757822486	6.63E-06	9.50E-05
PARG20377	1.574597792	1.635458242	6.72E-06	9.62E-05
PARG23935	-2.923057583	2.076027497	6.76E-06	9.66E-05
PARG00174	-1.218340218	7.844652394	6.80E-06	9.71E-05
PARG19064	-1.240726597	7.271683524	6.87E-06	9.81E-05
PARG06942	-1.21801842	5.799049876	6.96E-06	9.92E-05

PARG00464	-1.267129533	4.729975887	6.99E-06	9.96E-05
PARG27486	-1.275311034	5.699728603	7.02E-06	9.98E-05
PARG28341	1.204631234	7.91436219	7.02E-06	9.98E-05
PARG29384	-1.224459479	6.343916127	7.03E-06	9.98E-05
PARG14201	1.419273403	1.174512118	7.07E-06	0.000100361
PARG06746	-2.022064274	2.029847293	7.16E-06	0.000101523
PARG06628	-1.255961965	7.333019464	7.19E-06	0.000101848
PARG09284	-5.729843725	-0.127226551	7.48E-06	0.000105874
PARG15463	1.198724843	9.020114558	7.64E-06	0.000108052
PARG15923	-1.638628996	2.212047305	7.92E-06	0.000111852
PARG27344	1.493609115	2.236157978	7.93E-06	0.000111896
PARG20619	-2.049902233	3.887019812	7.95E-06	0.000112088
PARG11079	1.98139039	2.138173042	7.95E-06	0.000112088
PARG11645	-1.225273618	6.263170884	8.00E-06	0.000112679
PARG12454	-1.204104623	7.663798045	8.03E-06	0.000112974
PARG13808	1.194849613	10.69184471	8.13E-06	0.000114232
PARG02946	-1.233805008	6.823088526	8.17E-06	0.000114719
PARG04388	1.297945594	3.074890513	8.18E-06	0.000114719
PARG10872	1.194363289	9.13356242	8.23E-06	0.000115372
PARG09437	-1.205829677	7.940041445	8.25E-06	0.000115501
PARG11844	-1.468597738	5.568860605	8.44E-06	0.000118046
PARG23397	1.24484291	4.840114892	8.48E-06	0.000118537
PARG17206	2.153569718	0.028435136	8.67E-06	0.000121063
PARG21074	-1.207933806	6.129831039	8.77E-06	0.000122326
PARG19072	-1.3029859	4.641988651	8.83E-06	0.000123049
PARG23130	-1.371186192	3.90389686	8.84E-06	0.000123126
PARG25302	-4.163161959	2.512121758	9.05E-06	0.000125867
PARG10614	-4.134997658	1.147058165	9.05E-06	0.000125867
PARG09361	-1.578432062	2.468644624	9.25E-06	0.000128463
PARG02036	-3.169345643	4.171261163	9.30E-06	0.000129115
PARG04124	-2.705160484	1.841312032	9.31E-06	0.000129169
PARG06005	-1.800957301	5.881364148	9.37E-06	0.000129867
PARG26008	1.198139843	6.088325999	9.50E-06	0.000131511
PARG07650	-1.372159732	4.517949943	9.63E-06	0.000133172
PARG20696	-1.809738988	3.52855533	9.81E-06	0.000135536
PARG17811	-2.597939531	3.275094782	9.82E-06	0.000135536
PARG29594	-1.34520668	3.750329221	9.83E-06	0.000135536
PARG24712	1.193714398	6.276652278	9.88E-06	0.000136136
PARG25230	-1.194372118	7.66890272	9.92E-06	0.000136573
PARG14351	-1.211438099	7.376114487	9.97E-06	0.000137101
PARG13602	-3.294602339	1.889384682	9.99E-06	0.000137223
PARG21752	3.035014951	-0.02401801	9.99E-06	0.000137223
PARG19022	1.23478189	3.814266729	1.00E-05	0.000137227
PARG12985	-1.885731725	2.644319503	1.01E-05	0.000138713
PARG16951	-1.559795383	2.127697586	1.06E-05	0.000145307
PARG13082	-1.62052493	1.790017518	1.07E-05	0.000145861

PARG21282	-1.216456659	4.671584375	1.08E-05	0.000147135
PARG22271	-1.20070543	6.432558327	1.10E-05	0.000149856
PARG15117	-1.224830716	6.00764475	1.10E-05	0.000150458
PARG13267	-1.213062538	6.95980121	1.13E-05	0.000154422
PARG09192	1.333862372	5.016134317	1.14E-05	0.000154807
PARG11892	1.175241979	9.069960687	1.14E-05	0.000155471
PARG09818	-1.208407964	4.289313694	1.15E-05	0.000156751
PARG24840	1.309000561	1.896951761	1.16E-05	0.000157573
PARG15821	-2.068079055	2.206902081	1.16E-05	0.00015789
PARG19334	-1.605400461	3.199965077	1.17E-05	0.000159151
PARG12431	1.246079852	1.884659778	1.19E-05	0.000160862
PARG21458	-1.378902019	3.802252576	1.19E-05	0.00016119
PARG06342	1.182627197	5.966243148	1.22E-05	0.000165538
PARG08538	1.292251767	2.265435983	1.22E-05	0.000165538
PARG11403	-1.215231156	5.464868409	1.23E-05	0.000165556
PARG28090	-2.291989769	1.579516159	1.23E-05	0.000166061
PARG25223	-2.206051468	1.752258224	1.23E-05	0.000166061
PARG03553	-1.328672563	3.849543096	1.23E-05	0.000166145
PARG23166	-1.84198915	4.230315859	1.23E-05	0.000166223
PARG03742	-1.184343284	7.502914565	1.24E-05	0.000166739
PARG24647	-1.20869312	5.410267885	1.25E-05	0.000168018
PARG08041	-3.848095339	-0.202807372	1.25E-05	0.000168018
PARG15325	-3.498496571	0.954106802	1.25E-05	0.000168018
PARG20568	-3.491114879	2.933104569	1.25E-05	0.000168018
PARG01963	1.360578458	2.462152744	1.26E-05	0.000169278
PARG16099	1.168750836	9.024628357	1.27E-05	0.00016948
PARG20096	-1.428447656	2.559588921	1.28E-05	0.00017173
PARG13688	-1.282272515	3.787815712	1.29E-05	0.000172134
PARG04147	-2.143771662	1.769629223	1.30E-05	0.000173165
PARG05370	-1.255662627	3.779328356	1.31E-05	0.000174896
PARG19422	1.242089553	4.09494451	1.32E-05	0.000176343
PARG11354	1.332353583	1.120361117	1.33E-05	0.00017688
PARG19279	-1.217190795	4.31960871	1.34E-05	0.000177738
PARG29472	1.538585362	1.061337412	1.34E-05	0.000178223
PARG12397	-1.214347855	7.09497188	1.34E-05	0.000178408
PARG03095	1.185443548	5.553565912	1.36E-05	0.000180571
PARG21534	-1.165650302	8.92079074	1.36E-05	0.000180734
PARG21313	-1.645832982	2.202717931	1.38E-05	0.000182398
PARG20883	-1.162598538	10.57501888	1.39E-05	0.000184151
PARG15794	-1.260594831	3.418506693	1.39E-05	0.000184242
PARG25008	-1.902531558	1.030262136	1.40E-05	0.000185529
PARG24853	1.183568601	5.585239083	1.42E-05	0.000187171
PARG15849	-1.175015825	6.148233936	1.47E-05	0.000193644
PARG13205	1.162281948	7.650060021	1.47E-05	0.00019432
PARG19471	-1.308739832	3.538477955	1.48E-05	0.00019526
PARG15965	-2.70101043	1.427743919	1.50E-05	0.00019704

PARG28009	1.318239755	2.663705749	1.51E-05	0.000198367
PARG11595	-4.073153393	3.557770031	1.51E-05	0.000198367
PARG14187	-2.873729098	0.239558377	1.51E-05	0.000198758
PARG01842	-1.62657382	2.791088133	1.53E-05	0.000200657
PARG29245	-1.693326618	0.94553771	1.54E-05	0.000201971
PARG21193	-2.305512225	2.729302357	1.55E-05	0.000202772
PARG02818	-1.338825915	3.431508602	1.55E-05	0.000203049
PARG03686	-1.25691293	5.129303865	1.56E-05	0.00020397
PARG26911	-1.461042305	6.217260483	1.56E-05	0.00020397
PARG15904	-1.455821949	1.189261952	1.56E-05	0.00020397
PARG10254	-1.587546393	4.945906366	1.56E-05	0.00020397
PARG27766	-1.166253462	7.311528966	1.57E-05	0.000204077
PARG18623	-1.302811275	5.340843054	1.57E-05	0.000204077
PARG03711	1.159796603	6.166752152	1.61E-05	0.000210033
PARG07304	-1.22468893	4.664838114	1.63E-05	0.000211318
PARG28245	-1.164458117	6.981935819	1.65E-05	0.000214107
PARG26832	-1.319990047	3.313123751	1.67E-05	0.000216602
PARG07104	-1.192748716	6.442376653	1.70E-05	0.000220646
PARG15190	1.158810042	6.36300896	1.71E-05	0.000220905
PARG25227	1.151678656	7.458201631	1.72E-05	0.000222648
PARG06980	-1.152157062	8.142704598	1.80E-05	0.000232939
PARG18722	1.504372015	5.382941203	1.81E-05	0.000233424
PARG02083	-1.386692995	2.986797254	1.82E-05	0.000234904
PARG07823	-1.372430919	2.861215552	1.86E-05	0.000240023
PARG24127	1.410383349	2.525202491	1.87E-05	0.000241505
PARG02460	1.162595179	5.45017114	1.90E-05	0.000244759
PARG12205	-2.466263868	5.91854037	1.91E-05	0.000245448
PARG10964	-1.938526144	2.341566041	1.91E-05	0.000245613
PARG08814	1.370257071	1.872881356	1.94E-05	0.000249658
PARG23125	-1.147156818	9.342733771	2.00E-05	0.000255961
PARG13032	-2.03835659	0.871214045	2.00E-05	0.000255961
PARG19714	1.203157878	4.806984887	2.01E-05	0.000257465
PARG10865	-3.410214378	3.614944452	2.01E-05	0.000257783
PARG02152	-1.146572148	7.716611352	2.02E-05	0.000257973
PARG05243	1.177413123	4.517097381	2.04E-05	0.000260245
PARG19487	-1.239294936	4.149175758	2.06E-05	0.000262665
PARG27715	1.200841104	2.284685028	2.10E-05	0.000267816
PARG00173	-1.220771828	4.315496906	2.11E-05	0.000268638
PARG20667	-1.375978369	4.28584534	2.17E-05	0.000276542
PARG27270	1.593695953	2.633617999	2.20E-05	0.000280378
PARG18948	-1.734989193	1.310422422	2.24E-05	0.000284815
PARG05563	-1.591175488	1.925765889	2.26E-05	0.000287079
PARG29156	-2.256888087	3.780226703	2.27E-05	0.000288802
PARG18466	-1.153897746	6.912754759	2.28E-05	0.000288802
PARG00764	-1.180964455	5.451178849	2.29E-05	0.00029019
PARG26478	-1.209359249	4.697047701	2.29E-05	0.000290398

PARG21565	-1.291849552	2.814982376	2.30E-05	0.000291573
PARG09413	-1.569965674	0.749785001	2.32E-05	0.00029359
PARG28426	-1.878165091	2.007920794	2.33E-05	0.000294207
PARG20177	1.132147567	9.068280744	2.34E-05	0.000295108
PARG03225	1.660852158	1.629645418	2.36E-05	0.000297317
PARG11809	-1.630548436	3.595620615	2.36E-05	0.000298148
PARG18207	1.136763357	5.538681479	2.48E-05	0.000312087
PARG08589	-1.245294124	2.807780939	2.50E-05	0.000315051
PARG03263	-1.402177732	3.212270696	2.51E-05	0.000315231
PARG03993	-1.175290606	5.564468995	2.53E-05	0.000317211
PARG14545	-4.000479041	-0.046095487	2.53E-05	0.000317211
PARG12356	-3.972415173	2.294462763	2.53E-05	0.000317211
PARG03554	-1.167174287	5.293238621	2.54E-05	0.00031838
PARG25878	-1.357316134	1.708767293	2.57E-05	0.000321842
PARG15193	-1.577675674	5.562150308	2.58E-05	0.000322638
PARG06209	1.128228584	7.094888377	2.59E-05	0.000323712
PARG03591	-1.193026955	5.372179302	2.60E-05	0.0003252
PARG04927	-2.177736583	3.150710181	2.67E-05	0.000332945
PARG01742	-1.606624347	1.037831117	2.68E-05	0.000334706
PARG13537	1.516295204	1.496182209	2.69E-05	0.000334887
PARG00714	-1.346332608	2.305716767	2.74E-05	0.000341737
PARG20054	1.134150223	5.926039332	2.75E-05	0.000341764
PARG15805	-1.239814091	4.291802866	2.82E-05	0.000351115
PARG17925	-1.301590397	4.750718702	2.84E-05	0.000352428
PARG02092	1.183973617	4.413566235	2.86E-05	0.00035544
PARG18737	1.120301276	9.197080572	2.89E-05	0.000358481
PARG08885	-1.129454362	7.401178725	2.90E-05	0.000360042
PARG13104	-1.145744519	5.158723662	2.95E-05	0.000365647
PARG29322	1.95046586	-0.322531494	2.98E-05	0.000368672
PARG28061	1.238980877	3.84256517	3.00E-05	0.000370638
PARG00842	-2.381638938	0.619291951	3.00E-05	0.000370638
PARG01041	-2.352285132	1.780759932	3.00E-05	0.000370638
PARG08694	-1.610222056	2.991618082	3.01E-05	0.000371551
PARG06847	-2.782414381	4.948629072	3.03E-05	0.000373653
PARG07982	-2.756496777	3.929741282	3.03E-05	0.000373653
PARG11724	1.122466449	6.149618844	3.04E-05	0.000374405
PARG19278	-1.119664443	8.711220686	3.06E-05	0.00037609
PARG21665	2.03567972	1.529834236	3.07E-05	0.000377435
PARG22277	-1.113780275	8.193930339	3.11E-05	0.000381666
PARG08503	-1.127253705	6.512702761	3.13E-05	0.000384556
PARG29564	1.78216217	0.657746052	3.20E-05	0.00039243
PARG26469	-1.292557826	3.186419346	3.26E-05	0.0003993
PARG19520	-1.169442736	3.980592388	3.27E-05	0.000400802
PARG05669	-2.628872037	2.573849523	3.30E-05	0.000404213
PARG12977	-1.243789026	3.911769961	3.33E-05	0.000406993
PARG23561	-1.129459505	5.704224926	3.34E-05	0.000407666

PARG08702	-1.149379168	5.157590725	3.43E-05	0.000418618
PARG16121	-1.328412015	3.840800101	3.52E-05	0.000429557
PARG15231	-1.148609363	5.155728042	3.53E-05	0.00042985
PARG24231	1.571712171	0.987220577	3.53E-05	0.000429873
PARG12315	-1.457491899	1.140104985	3.55E-05	0.000431541
PARG23371	1.110510599	6.537077976	3.60E-05	0.00043793
PARG07266	1.138478809	4.101873993	3.62E-05	0.000439885
PARG11254	-1.443888792	4.23423155	3.63E-05	0.000440663
PARG17829	-1.624794454	4.13874477	3.71E-05	0.000449764
PARG20455	-1.190904464	3.080337418	3.74E-05	0.000453689
PARG23463	-1.49900012	6.719844608	3.82E-05	0.000463188
PARG02727	1.100250188	8.717336886	3.87E-05	0.000468511
PARG09540	-1.23424929	3.777647813	3.89E-05	0.000470122
PARG20916	-1.202929363	4.064804018	3.89E-05	0.000470257
PARG06088	-2.589268629	1.424567026	3.92E-05	0.000472706
PARG20468	-2.385501972	3.820131848	3.92E-05	0.000472706
PARG22697	-1.778456379	3.860062968	3.96E-05	0.000477139
PARG28287	1.126056646	4.725040972	3.99E-05	0.000480839
PARG24709	1.103758133	7.187895035	4.00E-05	0.000481371
PARG12645	-1.106357037	6.323545423	4.03E-05	0.000485278
PARG16069	-1.145431228	4.79851907	4.05E-05	0.000486602
PARG24420	-1.159925518	4.177201416	4.11E-05	0.000493697
PARG20707	-1.096623	8.879939571	4.12E-05	0.000494282
PARG09174	-1.438436064	2.812123792	4.14E-05	0.000495977
PARG30014	-5.566486338	1.405761931	4.14E-05	0.000496421
PARG06422	-1.338585962	2.429596612	4.16E-05	0.000497606
PARG00524	-1.139223658	4.805868316	4.18E-05	0.000499899
PARG07853	-1.110688454	5.891381587	4.18E-05	0.000499991
PARG02935	-3.950107264	2.06425879	4.25E-05	0.000507347
PARG08636	-1.203751524	3.947569261	4.36E-05	0.000520266
PARG19988	1.101161682	6.258215379	4.38E-05	0.000522482
PARG27599	-1.093198347	9.706554371	4.39E-05	0.000523246
PARG18006	-1.22157191	5.035863553	4.39E-05	0.000523335
PARG12507	-1.097541517	6.864082464	4.40E-05	0.000523335
PARG18401	-2.705336492	0.747138046	4.42E-05	0.000526
PARG28235	-1.095495091	7.567626391	4.46E-05	0.000529936
PARG14816	-1.696081264	1.739132385	4.48E-05	0.000532322
PARG27611	1.179584166	4.380568215	4.55E-05	0.000540239
PARG11782	-1.277215338	6.404104332	4.66E-05	0.000552876
PARG09004	1.194518439	4.727562998	4.70E-05	0.000556536
PARG16634	-1.166866452	5.121896887	4.88E-05	0.000577246
PARG22468	-1.133213955	6.739563082	4.90E-05	0.000580049
PARG27132	-2.58512968	1.781868154	4.91E-05	0.000580909
PARG00670	-2.095329839	2.391064797	4.95E-05	0.000584176
PARG08939	-1.539834535	1.081800207	4.98E-05	0.000588365
PARG05788	-1.093319285	6.402452981	5.15E-05	0.000607532

PARG27212	-1.65498892	1.131255644	5.19E-05	0.000611946
PARG18888	-1.260047791	2.315779657	5.20E-05	0.000612807
PARG15623	-3.325569365	2.302201094	5.23E-05	0.000615774
PARG08905	-1.082593869	8.142971262	5.24E-05	0.000616362
PARG06490	1.465365419	0.489309173	5.28E-05	0.000620612
PARG19478	-1.18061369	3.964001283	5.32E-05	0.000624944
PARG00477	-1.32241229	3.181636186	5.45E-05	0.000639559
PARG08615	-1.454243651	2.032042101	5.57E-05	0.000653091
PARG06114	1.077652048	8.266331601	5.71E-05	0.000669002
PARG11477	-2.062916291	2.203743865	5.72E-05	0.000670004
PARG02014	2.247905179	0.033670769	5.82E-05	0.00068071
PARG13234	-2.185587951	1.687394691	5.82E-05	0.00068071
PARG21271	-1.607159816	1.592635471	5.88E-05	0.000686875
PARG11341	1.075595764	6.390015649	5.93E-05	0.000691777
PARG07416	-1.267014996	3.784613887	5.95E-05	0.000694361
PARG20506	-1.800643601	3.613739007	5.99E-05	0.000697597
PARG18097	-1.675573737	2.528736971	6.02E-05	0.000701439
PARG02464	1.07985316	6.654103016	6.03E-05	0.000701439
PARG03957	-1.755464994	1.292233318	6.08E-05	0.000706785
PARG23123	-1.205466773	3.58362599	6.08E-05	0.000706785
PARG18532	-1.165947817	5.403156251	6.09E-05	0.000707003
PARG24594	1.115778493	3.416659566	6.10E-05	0.000707245
PARG03279	-1.101088906	5.737660924	6.11E-05	0.00070764
PARG12776	-1.124421526	5.103376646	6.11E-05	0.00070764
PARG27800	-1.882430842	1.225197391	6.13E-05	0.000709114
PARG19332	-1.249430768	3.615467277	6.17E-05	0.00071344
PARG17358	2.059358196	-0.848169161	6.50E-05	0.000751224
PARG23413	-2.000871558	1.331347939	6.50E-05	0.000751224
PARG07602	-1.678070767	0.795379011	6.55E-05	0.000755855
PARG13737	-1.652068151	1.109725994	6.55E-05	0.000755855
PARG05631	1.08188271	4.934425924	6.58E-05	0.000758959
PARG25344	1.114983732	2.586147262	6.59E-05	0.000759381
PARG06569	1.101731752	5.000859963	6.62E-05	0.000761731
PARG27473	1.076981292	5.969097508	6.63E-05	0.000762114
PARG03511	-1.149270269	4.31070308	6.76E-05	0.000776674
PARG27359	1.078953304	5.958837796	6.77E-05	0.000776674
PARG04720	-1.423563548	1.871679128	6.77E-05	0.000776674
PARG27804	-1.482594225	6.06172814	6.79E-05	0.000778761
PARG11480	-2.222627593	3.077912066	6.81E-05	0.000779524
PARG02685	-1.262976071	3.927731719	6.81E-05	0.000779524
PARG18551	-2.264526695	1.916683821	6.86E-05	0.000784814
PARG23353	-1.290442438	4.702296872	6.87E-05	0.000785644
PARG00957	1.068280318	6.916034472	6.88E-05	0.000785644
PARG20010	1.242885784	2.070908253	6.92E-05	0.000790601
PARG19285	1.195202673	3.797989534	6.95E-05	0.000792979
PARG24732	-1.568811416	1.451690958	7.06E-05	0.000804839

PARG11553	1.162331105	3.663098965	7.13E-05	0.000812447
PARG18890	-1.263050373	2.884395101	7.15E-05	0.000813771
PARG07022	-3.863665956	2.928478154	7.16E-05	0.000813771
PARG03825	-3.826081602	0.489692033	7.16E-05	0.000813771
PARG20330	-1.120571978	5.104751548	7.17E-05	0.000814524
PARG23812	1.058838079	10.59766787	7.30E-05	0.000828099
PARG19703	1.895791715	-0.2328227	7.31E-05	0.000829271
PARG26621	-2.632571507	1.179051789	7.32E-05	0.000829271
PARG11662	1.145032817	2.977620449	7.38E-05	0.000835362
PARG23224	-1.073961844	9.097944029	7.41E-05	0.000838107
PARG04347	-5.465869073	1.098347049	7.41E-05	0.000838411
PARG06203	1.248901382	2.140488262	7.42E-05	0.000839138
PARG13266	-1.069465812	7.865993136	7.48E-05	0.0008453
PARG10470	1.059140387	8.940125584	7.49E-05	0.000845767
PARG08835	-1.134705007	3.760810419	7.52E-05	0.000848042
PARG08502	-1.567182039	6.539190074	7.54E-05	0.000850101
PARG06046	1.077758802	4.649726503	7.61E-05	0.000856986
PARG09126	-1.080577095	5.475287351	7.62E-05	0.00085736
PARG29406	1.587545717	2.802023999	7.63E-05	0.00085846
PARG02702	1.10209066	3.421297927	7.76E-05	0.00087187
PARG27929	-1.329227407	2.114592277	7.80E-05	0.000875894
PARG07796	-1.173289851	2.457281602	7.81E-05	0.000875894
PARG00008	1.209808058	4.717016027	7.84E-05	0.000878619
PARG08666	-1.153262149	3.451604155	7.86E-05	0.000880828
PARG04060	1.054131526	9.108664326	7.91E-05	0.000885505
PARG03411	-1.446759897	2.19312129	7.92E-05	0.00088601
PARG22601	-1.057006776	8.749705122	7.98E-05	0.000892525
PARG16651	-1.103025585	3.613403328	8.02E-05	0.000896321
PARG15418	-1.811901163	0.157104465	8.04E-05	0.000897798
PARG11267	-1.139421954	3.908623562	8.06E-05	0.000898754
PARG29144	-2.685854078	0.801628499	8.10E-05	0.000902886
PARG07967	1.093019527	4.954806014	8.29E-05	0.000923479
PARG05246	1.06209399	5.219069704	8.32E-05	0.000926572
PARG15797	1.06887742	5.457434111	8.34E-05	0.000928291
PARG24749	-1.052770543	8.271552864	8.39E-05	0.000932311
PARG25077	-1.051646761	8.217355861	8.44E-05	0.000937563
PARG11424	-1.073211922	6.173581035	8.46E-05	0.00093799
PARG21864	-3.239891585	0.827918653	8.47E-05	0.00093799
PARG17812	-3.238027581	2.776095272	8.47E-05	0.00093799
PARG01318	-1.082066386	5.434079081	8.47E-05	0.00093799
PARG04015	1.054493358	6.502785373	8.73E-05	0.000966334
PARG23370	-1.065582798	5.983430501	8.80E-05	0.000973031
PARG01643	-1.057253111	6.374521863	8.83E-05	0.000976331
PARG21274	-1.052238946	7.050805223	8.84E-05	0.000976723
PARG14926	-1.546968285	2.690898276	9.00E-05	0.000993141
PARG04435	-1.080030297	4.802833682	9.06E-05	0.00099916

PARG18449	-1.578012441	3.284756138	9.26E-05	0.00102108
PARG25317	1.317912958	2.636577558	9.38E-05	0.001032913
PARG09407	-1.157574464	3.606074421	9.47E-05	0.001042407
PARG09191	-1.090977477	5.395139104	9.48E-05	0.001042424
PARG26144	-1.773138491	2.394037476	9.60E-05	0.00105418
PARG05509	-1.766562912	4.271435152	9.60E-05	0.00105418
PARG24010	1.040468929	9.907944477	9.68E-05	0.001062742
PARG00836	-1.059149767	5.657746864	9.70E-05	0.001063629
PARG06006	-2.003459976	6.004376269	9.70E-05	0.001063755
PARG16944	-1.051722933	6.751644283	9.82E-05	0.00107557
PARG00361	1.041416422	8.2241675	9.84E-05	0.001076779
PARG12529	1.08262903	4.060603121	9.91E-05	0.001084222
PARG13768	-1.162353228	4.940683828	9.98E-05	0.001091228
PARG20004	-1.227757473	3.059255454	0.000100233	0.001094959
PARG02486	1.314860988	2.008411468	0.000100521	0.00109734
PARG14073	-1.121109671	4.784244602	0.00010067	0.001098204
PARG15792	-1.749432919	1.500506864	0.000101222	0.001103457
PARG19819	-1.039477898	8.147501767	0.000101708	0.001107989
PARG27291	-1.153332648	6.258921555	0.000101889	0.001109196
PARG03481	-1.365235662	5.571462541	0.000102282	0.001112694
PARG12941	-1.06558404	6.667525358	0.000102856	0.001118165
PARG29529	1.062557625	4.923784781	0.000103167	0.001120771
PARG27652	1.045689378	6.151875887	0.000104657	0.001136169
PARG25948	-1.167878155	3.384369221	0.0001051	0.001140198
PARG22776	-1.081950255	6.071711867	0.000105897	0.001148051
PARG19028	-1.855701165	2.501727181	0.000106234	0.001150904
PARG24019	1.041899611	6.251846757	0.00010639	0.001151801
PARG14350	-1.036920596	10.50954852	0.000107078	0.001157684
PARG08051	1.039191382	7.037605017	0.000107081	0.001157684
PARG04962	1.495662444	0.614003379	0.000107276	0.00115899
PARG19018	-1.044058423	6.610505277	0.000107449	0.001160062
PARG03287	1.11930647	3.00021203	0.000108419	0.001169732
PARG11081	-1.117937492	3.991577735	0.000108624	0.001171141
PARG07527	-2.493382805	2.442558393	0.000109008	0.001174477
PARG12557	-1.140929576	4.019318509	0.000110736	0.00119227
PARG10605	-1.182163071	2.89536691	0.000111411	0.001198718
PARG24611	-1.219528578	4.395618656	0.000111847	0.001202589
PARG19973	1.114772291	3.464542741	0.000112832	0.001212348
PARG26456	2.010738239	0.729156791	0.000113118	0.001214591
PARG01344	-1.031496438	8.409027511	0.000113716	0.001219536
PARG12667	1.135882839	4.204331668	0.000113734	0.001219536
PARG21675	1.08020912	2.886536029	0.000114835	0.001230496
PARG11775	-1.921151452	4.63450306	0.000115102	0.001232519
PARG17830	1.11777738	4.405824828	0.000115654	0.001237581
PARG11118	2.35721052	0.45759092	0.000115743	0.001237695
PARG08357	1.235253115	2.609197989	0.000116137	0.001240034

PARG10608	-1.270780848	3.063434659	0.000116175	0.001240034
PARG24066	1.028999284	8.998386352	0.000116199	0.001240034
PARG11353	1.081045742	2.685020612	0.000116562	0.001243061
PARG22658	-1.036068942	8.056628324	0.000117132	0.001248298
PARG16683	-1.103927649	4.201431085	0.000118082	0.001257563
PARG28436	1.029706275	8.58558227	0.000121888	0.001297218
PARG29684	-1.042661021	7.66520982	0.000122908	0.001307184
PARG15722	-2.662058686	2.852329171	0.000123497	0.001312565
PARG27685	-1.512570281	1.570757196	0.000125044	0.001328103
PARG27752	1.677873367	-0.340957793	0.000127397	0.001351266
PARG13388	-1.657259775	1.291427609	0.000127397	0.001351266
PARG06086	-1.10771139	3.267826231	0.00012832	0.001360144
PARG24155	-1.997075232	1.7752943	0.000128921	0.001365593
PARG12168	1.024563992	6.652027382	0.000129533	0.001371142
PARG23844	-1.051815026	4.728035926	0.000129801	0.001373062
PARG29502	1.058723558	5.190077784	0.000130409	0.001378554
PARG19901	1.144496645	2.554765279	0.000131121	0.001385149
PARG20401	-1.085165724	5.047108376	0.000133115	0.001405275
PARG12721	-5.397650555	-0.174072615	0.000133436	0.001406376
PARG02375	-5.325154602	0.243734485	0.000133436	0.001406376
PARG12063	1.059173468	4.456679136	0.000133488	0.001406376
PARG06568	-1.282549119	2.245559182	0.000134021	0.001411043
PARG16791	1.069992217	3.053031075	0.000136454	0.001435691
PARG12041	-1.380139114	2.859671872	0.000138304	0.001454188
PARG05693	-1.081630135	4.848622274	0.00013946	0.00146536
PARG25121	-1.476478092	3.372626466	0.000140515	0.00147545
PARG22098	-1.063108371	4.428359553	0.000142816	0.001498414
PARG29956	-1.072922648	6.196001655	0.000142892	0.001498414
PARG06669	-1.019149816	8.134986008	0.000145236	0.001521968
PARG00972	-1.046086103	6.733578535	0.000147012	0.00153956
PARG28418	-1.21432645	4.795608201	0.000147426	0.001542866
PARG23950	1.056131164	3.69962403	0.000148338	0.001551375
PARG26734	-1.107984369	4.110669651	0.000148647	0.001553565
PARG18357	-1.023105041	7.274809404	0.000149552	0.001561984
PARG07466	-1.805367401	1.049827533	0.000150805	0.001574022
PARG20880	-1.012897801	9.083554389	0.00015331	0.001599111
PARG23842	-1.036270235	6.140199983	0.000154726	0.001612806
PARG10946	-1.156041946	3.153319231	0.00015676	0.00163292
PARG01159	-1.304041303	7.692360326	0.00015719	0.001636314
PARG15754	-1.023299543	6.626011061	0.000157859	0.001642197
PARG25383	-1.019539881	8.138976861	0.000158841	0.001650387
PARG06009	1.012475159	7.001200578	0.000158857	0.001650387
PARG06721	-1.156675705	4.496443301	0.000159246	0.001653334
PARG24682	-1.017400429	6.761875207	0.000159386	0.001653698
PARG08477	-1.100445136	5.611907702	0.000159792	0.001656815
PARG23931	-1.710664406	2.151011823	0.000160308	0.001660608

PARG00023	1.435888273	1.182815961	0.000160433	0.001660608
PARG21275	-1.302554113	2.215098162	0.000160475	0.001660608
PARG19719	1.00650145	9.797303367	0.000160996	0.001664902
PARG00343	-1.102305769	4.527917674	0.000162443	0.001678754
PARG24507	-1.361679104	2.890283443	0.000163117	0.001684616
PARG04392	-1.197556132	1.528123828	0.000163631	0.00168881
PARG00922	-1.030738378	5.83780105	0.000164037	0.0016913
PARG18855	1.021868187	5.648664444	0.000164088	0.0016913
PARG03981	1.00517875	9.305441842	0.000164943	0.001699002
PARG02891	-2.435398096	1.347776617	0.000166179	0.001709486
PARG10995	-2.372871387	1.216481275	0.000166179	0.001709486
PARG05854	-1.346923989	1.009942589	0.000168996	0.001737331
PARG06687	-1.019354873	5.371899498	0.000169515	0.001741032
PARG20106	-1.102014871	2.978403391	0.000169578	0.001741032
PARG10343	-1.038196431	4.59570707	0.000169691	0.00174105
PARG10703	-1.049387936	5.226246249	0.000170875	0.001752053
PARG06198	-1.079209696	4.932416978	0.000173269	0.001775438
PARG27477	-1.057027746	4.631434877	0.000174345	0.0017853
PARG15407	-1.010742106	5.838425831	0.000176089	0.001801982
PARG10150	1.013753641	5.220183431	0.000176606	0.001806103
PARG02361	1.748491126	0.140935179	0.000178725	0.001826579
PARG10374	1.363249704	0.410119636	0.000179813	0.0018365
PARG13754	-1.007823512	6.583555094	0.000180743	0.001844796
PARG15147	1.414939936	-0.040876529	0.000181719	0.001853554
PARG26814	-1.13042897	3.935303844	0.000183402	0.001868296
PARG07103	1.107546605	3.028781012	0.000184396	0.001877204
PARG03641	-1.079946464	3.031882342	0.000184804	0.001880141
PARG20505	-1.899683011	1.926551611	0.000185422	0.001885204
PARG15744	-1.001722569	8.508613386	0.000185771	0.001887525
PARG02429	1.013476783	6.925695839	0.000186102	0.001889668
PARG03480	1.182957267	2.513475646	0.000190127	0.001928041
PARG21533	-1.130461637	2.771756917	0.000194066	0.001965454
PARG26590	-1.613375205	0.49202648	0.000194549	0.001969076
PARG03444	-1.295709203	2.964899156	0.000196227	0.001984773
PARG07642	-1.000428333	7.277642196	0.000196462	0.001985874
PARG16311	-1.280026913	2.357731834	0.000196877	0.00198879
PARG08607	-1.285998005	3.853323602	0.000197495	0.001993751
PARG12439	-1.080676696	3.807496343	0.000199894	0.002016667
PARG13132	1.087132174	2.520930887	0.000201034	0.00202687
PARG30380	-1.611183709	2.05440546	0.000201673	0.002032006
PARG23958	-1.537962558	6.167025266	0.000204077	0.002053587
PARG06867	-1.117186503	3.300251087	0.000204744	0.002058986
PARG13046	-1.007538091	6.72153458	0.000204887	0.002059098
PARG27305	-3.745837698	0.182307666	0.000205502	0.002061319
PARG17623	-3.678134259	2.453462159	0.000205502	0.002061319
PARG23085	-3.67691922	1.870963623	0.000205502	0.002061319

PARG22444	-1.008577619	6.01009161	0.000205767	0.002062662
PARG19311	-1.376892321	2.841491321	0.00020607	0.002064379
PARG19020	-1.046265959	5.938165481	0.000206221	0.002064576
PARG28091	-1.137921138	1.860489308	0.000206928	0.00206847
PARG27174	-1.156897132	3.958396495	0.000207005	0.00206847
PARG12664	-1.005170016	6.970981436	0.000207312	0.002070217
PARG19874	-1.492138641	1.243145482	0.000207522	0.002071001
PARG11210	-1.627445522	4.378085393	0.000208301	0.002077454
PARG10970	-1.368635796	1.509219788	0.000208674	0.002079855
PARG12944	-1.062172628	5.145672121	0.000209256	0.002084323
PARG21317	-2.858815869	0.919585846	0.000211598	0.002106314
PARG05987	-1.035543886	4.303381173	0.000214741	0.002136256
PARG14377	1.053541827	3.39426826	0.000215316	0.002139258
PARG07794	-1.695475573	0.572567065	0.000219289	0.002177354
PARG22183	-1.296430246	1.77260195	0.000219506	0.002178131
PARG10003	1.048634948	1.884377916	0.000221985	0.002201343
PARG19596	-1.068112152	3.293353912	0.000222156	0.002201644
PARG24262	-3.162605655	1.507434633	0.000222945	0.002203894
PARG12713	-3.126406235	-0.838601287	0.000222945	0.002203894
PARG00628	-3.116484168	-0.942524136	0.000222945	0.002203894
PARG26909	-1.007956091	6.079068329	0.00022339	0.002206906
PARG20164	-1.208428181	5.482875331	0.000224594	0.002217403
PARG12345	-1.195620542	3.816989286	0.000224842	0.002218456
PARG24997	-1.616834883	1.347401671	0.000225459	0.002223146
PARG27558	-1.108686195	2.970397367	0.000225772	0.002224844
PARG14248	-1.108818909	5.768277729	0.000226534	0.002230951
PARG17098	-1.048641125	3.675204966	0.000227013	0.002234266
PARG07665	1.051690539	2.69663995	0.000229457	0.002255695
PARG18372	-1.37488484	2.52305989	0.000229713	0.002255695
PARG08119	-1.066988981	4.025909105	0.000229765	0.002255695
PARG09360	-1.363022274	1.862848489	0.000234416	0.002298476
PARG08349	1.357188615	1.112321831	0.000235759	0.002310203
PARG01554	1.03813972	4.170687227	0.000238503	0.002335633
PARG24488	1.129591044	2.214731255	0.000238887	0.002337939
PARG19672	-1.068525879	5.388723589	0.000239829	0.002345691
PARG20885	-5.292430701	2.940747719	0.000241628	0.002360349
PARG13653	-5.234505066	-0.974255441	0.000241628	0.002360349
PARG30407	-2.505189764	-0.250130947	0.000242371	0.002366137
PARG20587	-1.360114705	1.417627362	0.000242669	0.002367565
PARG00296	2.013244336	0.005000831	0.000244154	0.002377628
PARG19516	-1.962147042	0.046136018	0.000244154	0.002377628
PARG23723	-1.10990194	3.668449852	0.000246448	0.002396989
PARG14763	-1.285766767	1.415512086	0.000247287	0.002400866
PARG14797	-1.285766767	1.415512086	0.000247287	0.002400866
PARG06857	-1.219711346	4.416879172	0.000247305	0.002400866
PARG12144	-1.040392025	4.003819829	0.00024848	0.002409285

PARG13057	1.069312941	2.864772658	0.000253276	0.002451247
PARG24911	-1.10753481	2.797001701	0.000254407	0.002460676
PARG00658	-1.086314753	3.74309245	0.000254616	0.002461186
PARG25321	-1.021314679	4.756482874	0.000256688	0.002479689
PARG28295	1.005225144	5.48197224	0.000257202	0.002483119
PARG04216	1.390387598	0.269114517	0.000257973	0.002489034
PARG08080	1.827102741	-0.579977294	0.000258679	0.002494311
PARG27156	-1.035660897	4.968458017	0.000260255	0.002506672
PARG13912	-1.419588837	3.589931347	0.00026028	0.002506672
PARG10873	-1.066384025	4.854996673	0.000261621	0.002518042
PARG12019	-1.021225911	5.576333855	0.000263163	0.002529973
PARG01782	-1.079142107	4.302544231	0.000263688	0.002533277
PARG19445	-1.086946387	4.865683818	0.000267618	0.002564759
PARG06182	-1.175779161	2.365119454	0.00026984	0.002582893
PARG07924	-1.588192689	3.726125844	0.000272597	0.0026077
PARG03685	-1.032875783	3.88206666	0.00027341	0.002613879
PARG21289	-1.329462914	4.965576133	0.000275176	0.002629162
PARG00608	-1.024131392	4.49908271	0.000280573	0.002677469
PARG09108	-1.744374427	1.244987381	0.0002828	0.002697082
PARG05658	-2.566378752	1.386200923	0.000287883	0.002742224
PARG10892	-1.020943522	2.899246487	0.000296922	0.002824901
PARG06533	-1.148349302	4.983662815	0.000298515	0.002838333
PARG27773	-1.215115491	2.555646976	0.000302504	0.002872785
PARG26445	-1.080482019	3.695828404	0.000303398	0.002879527
PARG02180	-1.027804548	4.64409117	0.000306318	0.002905485
PARG07213	-1.459090901	5.443566521	0.000310646	0.002941203
PARG11779	-1.397813509	5.967322074	0.000310646	0.002941203
PARG18996	-1.086250554	3.264990965	0.000311827	0.002950605
PARG15675	1.144780261	0.385207212	0.000316542	0.002988016
PARG20390	1.303072803	0.088799946	0.000317704	0.002997189
PARG14456	-1.617722338	3.422429578	0.000318889	0.003006555
PARG23037	1.065153183	2.035041331	0.000323893	0.003051902
PARG07812	1.083208927	3.675649061	0.000329931	0.003101354
PARG26135	1.035235698	2.379692095	0.000331622	0.003115385
PARG06828	-2.745559199	2.645402868	0.000332489	0.003119791
PARG26855	1.546364984	1.200467299	0.00034227	0.00320773
PARG03017	1.070882933	2.010561491	0.000343354	0.003215978
PARG30037	1.484995361	-0.09829461	0.000346576	0.003244078
PARG08639	-1.042408426	3.91609785	0.000346818	0.003244078
PARG07512	1.090400625	5.599638533	0.000347434	0.003246436
PARG04905	-1.036494704	4.939559883	0.000354296	0.00329878
PARG08941	-1.44819289	1.70940055	0.000356589	0.003316194
PARG10302	-1.124557212	0.609364475	0.000357102	0.003319002
PARG12430	1.014473248	2.727363893	0.000358067	0.003326
PARG06327	-1.269923138	1.535531667	0.000358669	0.003329617
PARG07366	1.239790762	1.436682116	0.000359689	0.003336171

PARG06455	-1.118193681	3.944129086	0.0003598	0.003336171
PARG18900	1.942443807	-0.244619333	0.000362649	0.003360154
PARG27360	-3.095265808	3.106201862	0.000362814	0.003360154
PARG00428	-1.657419612	1.33717631	0.000364567	0.003374392
PARG28170	1.304564419	1.733542568	0.000367729	0.003395654
PARG22828	-1.368468399	0.78422229	0.000368569	0.003401407
PARG24125	-1.018881051	3.696060989	0.000369526	0.003408237
PARG02430	-2.068754871	1.289645331	0.000386835	0.003553269
PARG07321	-1.029726285	4.265863074	0.000391624	0.003595156
PARG07432	1.091534225	1.88795123	0.000393265	0.003608105
PARG13245	1.100332644	1.898606107	0.000401084	0.003667883
PARG20119	-1.086615654	2.976370027	0.000401084	0.003667883
PARG21270	-1.035388154	3.265395339	0.000401182	0.003667883
PARG06517	1.115504203	1.854419408	0.000408768	0.00373289
PARG26931	-1.579139576	0.015976944	0.000412709	0.003760131
PARG02232	-1.014547766	5.264905668	0.000417816	0.003802245
PARG26786	-2.127146762	5.185679998	0.000426602	0.00387546
PARG19900	-1.946243076	2.977362538	0.000426602	0.00387546
PARG27403	1.903892584	0.06458022	0.000426602	0.00387546
PARG19833	1.449348182	0.681639845	0.000427713	0.003883309
PARG00420	-1.06603678	2.674015186	0.000432288	0.003920307
PARG03443	-1.369157506	2.392627338	0.000433852	0.003932219
PARG11831	-1.142150769	3.276424304	0.000435009	0.003940437
PARG26897	-1.427171121	3.13853811	0.000435397	0.003941674
PARG17958	1.20366132	1.734581781	0.000437456	0.003958034
PARG12840	-5.205097211	-0.306201917	0.000440227	0.003976226
PARG00506	5.18014083	-0.000740003	0.000440227	0.003976226
PARG29458	-5.133382432	0.976478385	0.000440227	0.003976226
PARG10620	-1.232445019	1.923252175	0.000464112	0.004182336
PARG16923	-1.197756267	2.923961305	0.000467097	0.004204406
PARG12517	1.032817102	2.866438435	0.000474134	0.00426042
PARG06243	-1.184423847	1.581738576	0.000504025	0.004503232
PARG12311	-1.000418351	5.602941072	0.000509139	0.004541173
PARG10351	-1.083432523	2.198335005	0.000515355	0.004591393
PARG24383	-1.545730324	1.558284485	0.000516297	0.004597178
PARG12839	-1.376992811	4.443764948	0.000521932	0.004634067
PARG00288	1.432027916	1.694341369	0.000522564	0.004634067
PARG19402	-1.414444992	0.373267945	0.000522564	0.004634067
PARG23022	-1.436993922	3.076191633	0.000522801	0.004634067
PARG01978	-2.197150025	0.013753721	0.000546459	0.004827411
PARG27527	-2.173374281	0.319548291	0.000546459	0.004827411
PARG27836	-1.034229503	2.733812069	0.000549222	0.004849093
PARG11588	-1.053397342	2.631702146	0.000558713	0.004930115
PARG25509	1.254874905	0.229768236	0.00056065	0.00494443
PARG00619	-1.01079705	4.518857901	0.000563026	0.004962597
PARG02733	1.075743467	2.513255129	0.000565387	0.004980609

PARG13271	-1.07567298	4.237314974	0.000573356	0.005047979
PARG18084	-1.653619656	-0.075695198	0.000579448	0.0050959
PARG12370	-1.007497665	2.518452572	0.000585499	0.005141452
PARG28225	1.15408294	1.879459966	0.000586191	0.005141452
PARG28270	1.150268993	1.325371524	0.000586191	0.005141452
PARG28996	-1.204874977	1.873033488	0.000592943	0.005194211
PARG27780	-3.585686985	0.913921356	0.000597412	0.00521589
PARG07131	-3.578248217	-0.922671168	0.000597412	0.00521589
PARG22609	-3.578097195	0.11272987	0.000597412	0.00521589
PARG09495	3.574605215	0.168210196	0.000597412	0.00521589
PARG16306	-3.533772485	1.516379184	0.000597412	0.00521589
PARG01443	-3.4223948	-0.266973748	0.000597412	0.00521589
PARG19158	-1.346413274	4.278813783	0.00060022	0.005234583
PARG15510	1.115083359	1.196396042	0.000602903	0.005252149
PARG28013	-1.674071204	2.382978074	0.000604999	0.005263551
PARG19754	-1.170434007	3.439913614	0.000608029	0.005280349
PARG21332	-1.034794211	4.085236275	0.000611795	0.005300199
PARG25224	-1.002918655	5.701956799	0.000612785	0.005305848
PARG09557	-1.60551085	1.60301948	0.000614409	0.005316974
PARG10965	-1.229839087	2.833029357	0.000615345	0.005322144
PARG12518	1.498101959	0.475646441	0.000627766	0.005420613
PARG19262	1.520963106	0.962090016	0.000632148	0.005454125
PARG14802	-1.033615952	3.418117477	0.000632342	0.005454125
PARG11715	-1.959179373	2.876214074	0.000634837	0.005469629
PARG00249	-1.863198728	4.609023205	0.000634837	0.005469629
PARG06775	1.442539144	0.753584608	0.000637236	0.005486495
PARG14299	1.434773455	1.037066611	0.000637843	0.005486495
PARG14450	1.434773455	1.037066611	0.000637843	0.005486495
PARG07241	-1.011578828	3.645364556	0.000645452	0.005545871
PARG12333	1.032744111	2.200012981	0.000646743	0.005553927
PARG15113	1.261681115	2.091463948	0.000664993	0.005704407
PARG10812	-2.420557056	0.045123471	0.000672342	0.005761147
PARG02408	-1.082790953	2.933892519	0.000690606	0.005903488
PARG10145	1.039598738	8.59979686	0.000696646	0.005946682
PARG29428	-1.765789198	1.727854715	0.000698378	0.005958229
PARG02774	-1.129827084	3.768719353	0.00071289	0.006068846
PARG14144	-1.019718459	3.347050823	0.000720312	0.00612538
PARG13268	-1.001603304	4.505944894	0.000729799	0.006185952
PARG00574	-1.106416644	2.713001691	0.000754737	0.006376676
PARG14978	-1.452910344	2.778926175	0.000759279	0.00640471
PARG30342	-1.20648651	0.832667327	0.000761464	0.006419692
PARG10454	-1.051914982	1.95090491	0.00077769	0.00654945
PARG12211	-1.495918971	1.738069386	0.000788606	0.006630708
PARG19827	-1.444973617	0.484576407	0.000791228	0.006649197
PARG10578	-1.498817961	3.197908943	0.000792852	0.006659278
PARG13097	-1.124574287	2.592757908	0.000795742	0.006679975

PARG24297	-5.079354044	-0.689830962	0.000807082	0.006764359
PARG14110	-5.011091708	0.291801218	0.000807082	0.006764359
PARG19574	-1.13678571	2.312713506	0.000807511	0.006764359
PARG01827	-1.012903501	2.579108973	0.000822778	0.006877514
PARG24922	1.801717642	-0.278365868	0.000826643	0.006906146
PARG02276	-1.025141472	5.112145597	0.000832165	0.006947223
PARG09034	-1.067246778	2.073700324	0.000837816	0.006988333
PARG16140	1.039272505	2.557633171	0.000846391	0.007052359
PARG13624	1.079700445	0.787046009	0.000846886	0.007052741
PARG21249	-1.126903517	2.06592652	0.000848692	0.007064035
PARG15832	1.005368193	2.271207578	0.00087625	0.007277972
PARG07074	1.349368718	0.352451563	0.0008823	0.007320481
PARG29440	-1.237741954	2.79292058	0.000892196	0.007386967
PARG27167	-1.861870192	1.323951049	0.000908636	0.007507246
PARG12549	-1.05009577	2.793596697	0.000910761	0.007520846
PARG14769	-1.184941997	3.626465073	0.000912789	0.007533627
PARG14105	-2.36781715	3.553484233	0.000917882	0.007567706
PARG16697	1.364092587	0.403197233	0.000925251	0.007616465
PARG20206	-1.19582331	3.050267822	0.000926279	0.00762093
PARG11331	-1.035311718	3.049069116	0.000940347	0.007721186
PARG20331	-1.276819296	3.470185659	0.000945573	0.007759336
PARG12116	-2.931099285	2.148006807	0.000965181	0.007886994
PARG08883	-2.901845877	0.934722333	0.000965181	0.007886994
PARG27420	-2.88483611	4.012790367	0.000965181	0.007886994
PARG26750	-1.101713396	2.435013039	0.000974169	0.007940812
PARG12081	-1.276270406	1.527196803	0.000974265	0.007940812
PARG26834	1.149836213	1.092576668	0.000976986	0.007955495
PARG28076	1.672495419	-0.102994452	0.00097708	0.007955495
PARG27825	-1.515316541	1.135639114	0.000981873	0.007986243
PARG08638	-1.125178282	2.288925127	0.000983611	0.00799623
PARG29811	-1.045729123	2.388923935	0.000985995	0.008011463
PARG27288	-1.667541968	4.197876572	0.000989129	0.008016182
PARG29486	-1.654427117	0.927176845	0.000989129	0.008016182
PARG00167	-1.63458836	1.265250396	0.00099709	0.008076533
PARG29258	-1.59294178	3.569489576	0.001001596	0.008108849
PARG07752	-1.543226334	2.572881225	0.001002356	0.008110816
PARG27854	-1.003774567	3.154104136	0.001011849	0.008168114
PARG18371	-2.023648297	0.124883981	0.001012039	0.008168114
PARG19354	-1.008054099	2.218293478	0.001017908	0.008208185
PARG25129	-3.492332187	2.175050366	0.001023013	0.008239744
PARG17428	-3.425533427	0.399317016	0.001023013	0.008239744
PARG23800	1.178553785	-0.288202186	0.001023825	0.008242052
PARG03307	-2.36414346	2.749647101	0.001027615	0.00826832
PARG07801	-1.231781309	2.529617066	0.0010501	0.008419036
PARG00490	-1.066922457	3.414108001	0.001056168	0.008449518
PARG28562	-2.080537675	3.652335769	0.0010886	0.008683352

PARG17208	1.977424894	-0.863053512	0.0010886	0.008683352
PARG01175	1.185533116	3.115053957	0.001093786	0.008715863
PARG03078	-1.12958852	2.936203346	0.001121405	0.008917837
PARG29728	1.112655125	0.933212926	0.001128556	0.008970163
PARG16082	1.03456565	3.489894668	0.001132518	0.008992547
PARG03652	-1.018838205	4.079995291	0.001135061	0.009008186
PARG20493	1.015458354	2.284700815	0.001186762	0.009366409
PARG08535	-1.433349223	1.92680901	0.00121769	0.009591222
PARG10606	-1.116319317	1.881296481	0.001223098	0.009625765
PARG27998	1.45689539	0.598157854	0.001259872	0.009878854
PARG02835	-1.081248011	2.063231108	0.001279437	0.010017256
PARG29952	-1.55071747	0.63510468	0.001290986	0.010097608
PARG26295	2.642125822	2.314869365	0.00129528	0.010126148
PARG04831	1.05899212	0.353911886	0.001300228	0.010155964
PARG01127	-1.065346769	0.797364167	0.001323105	0.010312863
PARG08608	-2.057473392	1.093749946	0.001339905	0.010417943
PARG29928	1.087485993	-0.001516174	0.001394435	0.010783173
PARG06560	-1.074065535	2.22617429	0.00141444	0.010931053
PARG22805	-1.33334299	0.588320828	0.001428751	0.011032236
PARG18180	2.050609473	-0.642360663	0.001450197	0.011164049
PARG08117	2.006049443	0.364337579	0.001450197	0.011164049
PARG02272	-1.165254696	1.963673429	0.001529672	0.011730724
PARG10425	-1.146890645	0.897920471	0.001538302	0.011785393
PARG23937	-1.10324809	1.720262074	0.001550847	0.011869918
PARG13939	-1.047475188	4.242447449	0.001559627	0.011919679
PARG15687	-1.085912483	1.436770679	0.001562156	0.011927392
PARG07813	2.88437347	0.1270536	0.001576871	0.011993079
PARG29742	-2.873556133	-0.025847452	0.001576871	0.011993079
PARG14104	-2.871160957	1.215092762	0.001576871	0.011993079
PARG00648	-2.870888004	3.16497185	0.001576871	0.011993079
PARG10986	-2.863680173	3.156895156	0.001576871	0.011993079
PARG25611	-2.795956469	0.376514736	0.001576871	0.011993079
PARG12197	1.064878907	5.915539186	0.001614605	0.012262252
PARG21175	-1.190233143	4.312290126	0.001642757	0.012451954
PARG08000	-1.014511374	1.908486342	0.001654523	0.012522997
PARG11007	-1.16785561	0.338081323	0.001680557	0.012677259
PARG11651	-1.662889951	4.143957506	0.001686068	0.01269968
PARG06642	-3.407278345	1.868870386	0.001756313	0.013179063
PARG26938	-1.055133355	2.836519142	0.001766142	0.013240159
PARG12378	1.286322301	1.566649044	0.001775339	0.013296407
PARG18788	-2.166741904	2.373888705	0.00178809	0.013360038
PARG04445	-2.156018682	1.580972701	0.00178809	0.013360038
PARG19539	-2.128315815	0.094288751	0.00178809	0.013360038
PARG13955	-1.102553376	3.441965646	0.001796574	0.013391553
PARG18669	1.119620881	1.647623248	0.001826835	0.013565588
PARG12353	-1.246729835	2.178039673	0.001848009	0.013685256

PARG18540	-1.181263463	3.082752375	0.001848009	0.013685256
PARG28314	-1.17180872	2.80101875	0.001849053	0.013685256
PARG24244	-1.235972975	1.517503201	0.001867059	0.013805509
PARG24905	1.335405535	0.198885184	0.001868871	0.013812401
PARG18461	1.106700146	0.964987863	0.001879977	0.013879785
PARG08585	-1.292189545	0.535454447	0.001915727	0.014093812
PARG12590	1.054883406	1.24034287	0.001915925	0.014093812
PARG28189	1.182184422	0.158710357	0.00194516	0.014275418
PARG03233	1.17315438	1.092950958	0.00194516	0.014275418
PARG14135	-2.036096539	2.824851659	0.001954202	0.014335079
PARG21671	2.526231471	-0.260343651	0.002038892	0.014893682
PARG09127	-1.916989669	0.521752746	0.0020759	0.015121801
PARG22005	-1.479646669	0.194257565	0.002080945	0.015137477
PARG27906	1.460456896	-0.159716099	0.002080945	0.015137477
PARG23188	-1.430716196	1.313192045	0.002080945	0.015137477
PARG12179	-1.0625573	2.138814572	0.002093295	0.015213214
PARG07445	-1.205167656	1.78035556	0.00210696	0.01529129
PARG08332	-1.078004418	1.664813907	0.002130509	0.015447911
PARG07761	-1.529249509	2.928925279	0.002158222	0.015612799
PARG23904	-1.939112643	1.164625014	0.002160809	0.015617116
PARG16450	-1.808541463	2.486562502	0.002160809	0.015617116
PARG15890	1.006018792	1.837462157	0.002173425	0.015693847
PARG22771	1.440333238	0.790392871	0.00219211	0.015797576
PARG17690	-1.177511806	3.92468545	0.002195545	0.015809941
PARG03121	1.090681628	1.676068297	0.002209599	0.015889274
PARG17647	-1.744245801	-0.043680152	0.002215887	0.015927193
PARG10589	1.565536283	1.512178056	0.002221118	0.015957486
PARG13247	1.626065855	1.694137397	0.002243542	0.016103851
PARG19880	-1.715661571	5.352558103	0.002247131	0.016114873
PARG04524	-1.710430683	2.172929086	0.002247131	0.016114873
PARG01666	-1.127640348	2.459046526	0.002257368	0.016180897
PARG24713	1.028568698	1.871746869	0.002263816	0.016219713
PARG16845	-1.037535301	3.128019394	0.002288499	0.016359225
PARG24230	-1.028227842	1.927980606	0.002288499	0.016359225
PARG24528	1.058770582	-0.018209345	0.002336934	0.016667512
PARG03684	-1.22305764	1.171105379	0.002362157	0.016786395
PARG23967	-1.210867385	3.579381473	0.002362157	0.016786395
PARG08986	1.039347936	2.690197135	0.002367245	0.016802352
PARG24685	-1.144553433	2.410364129	0.002390522	0.016919034
PARG13436	-1.386175658	0.566456672	0.002443316	0.017253787
PARG27372	-1.037935263	3.41196716	0.002506456	0.017662988
PARG01990	1.19306541	2.377874968	0.002510026	0.01767716
PARG14320	-1.103735937	1.791372013	0.002529612	0.017783183
PARG20561	-2.794314972	2.340132712	0.002577856	0.018076894
PARG00474	-2.789029667	1.010196129	0.002577856	0.018076894
PARG24294	-2.78670241	0.409172835	0.002577856	0.018076894

PARG24666	-2.777128692	0.250271959	0.002577856	0.018076894
PARG12921	-2.732131168	-0.480441057	0.002577856	0.018076894
PARG19761	1.315338315	2.22660345	0.00257964	0.018076894
PARG05565	-1.027977253	3.121903717	0.002581755	0.018076894
PARG02282	1.314210029	-0.49881741	0.0025983	0.018168422
PARG00567	-1.471840198	-0.154221309	0.00264887	0.018472663
PARG23952	-1.403450318	0.233728627	0.00264887	0.018472663
PARG30355	-1.085598287	3.174581885	0.002650088	0.018472948
PARG19534	-2.074289786	2.78237558	0.002661869	0.018520399
PARG23278	-1.013568832	1.951873529	0.002675247	0.018598774
PARG12166	-1.307566552	3.007299628	0.002681412	0.018632664
PARG07478	-4.874765215	2.094046399	0.002765516	0.01909946
PARG10729	-4.752424761	0.049941162	0.002765516	0.01909946
PARG24077	-4.746391614	0.332188845	0.002765516	0.01909946
PARG07363	4.723993247	2.585264492	0.002765516	0.01909946
PARG25435	-1.090558335	1.667699088	0.002767583	0.019105331
PARG11952	1.083599009	0.746544841	0.00281114	0.019371937
PARG22212	2.031234054	-0.553291783	0.002846741	0.019574303
PARG18637	-1.97575384	2.319518505	0.002846741	0.019574303
PARG18670	1.147023209	1.627968649	0.002875973	0.01974072
PARG20091	-1.011682043	1.683027727	0.002910118	0.019931515
PARG13066	-1.03136536	2.254602751	0.002926854	0.020015113
PARG02245	-3.23046205	0.328634746	0.003022008	0.020536573
PARG17441	-3.193834625	-0.241309673	0.003022008	0.020536573
PARG01985	1.101754567	2.410912888	0.00303571	0.020602931
PARG13571	-1.699746901	1.661081222	0.003053868	0.020708257
PARG24277	-1.790855462	-0.014861311	0.003073318	0.020814746
PARG00893	1.291809464	0.323475158	0.003166511	0.021370545
PARG27206	-2.440737117	-0.110043041	0.003208044	0.021613677
PARG08522	-2.434782804	4.336083148	0.003208044	0.021613677
PARG27198	-1.091164523	3.207108436	0.003211911	0.021630452
PARG21427	-1.011872051	2.039252247	0.003337192	0.022399333
PARG19527	-1.321743965	0.28736159	0.003531164	0.023558098
PARG07320	-1.161313845	3.167021061	0.003539542	0.02360396
PARG18400	-1.017009223	0.805505324	0.003598507	0.023915902
PARG27111	-2.227848783	-0.057470765	0.003657417	0.024228207
PARG02221	-2.213210632	0.988965224	0.003657417	0.024228207
PARG00052	1.307088534	0.55656577	0.003666006	0.02423526
PARG28523	-1.274126969	0.672862897	0.003666006	0.02423526
PARG25263	-1.239951535	0.86989856	0.003666006	0.02423526
PARG23638	1.098531861	2.901268551	0.003668173	0.02423526
PARG05856	-1.512348855	1.486153923	0.003705543	0.024458345
PARG12487	-1.167785184	2.586986685	0.00373965	0.02465562
PARG16670	-1.08754592	3.149780491	0.003791549	0.024951857
PARG25141	-1.150153944	1.296455423	0.003883233	0.025441985
PARG23222	1.056952438	1.596797356	0.003925165	0.025684551

PARG01903	-1.503771207	1.414375017	0.003926832	0.025684751
PARG15815	-2.030115979	3.630568447	0.003957315	0.025801441
PARG00643	-1.167020273	1.23128833	0.003971006	0.025844469
PARG03221	-1.045380891	1.021476944	0.004073765	0.026425604
PARG07164	1.335983625	1.041880436	0.004113519	0.026628462
PARG08600	-2.107015215	2.467750273	0.004140647	0.026726921
PARG18248	-1.878650349	1.543808072	0.004140647	0.026726921
PARG05884	-1.871377147	-0.321910938	0.004140647	0.026726921
PARG26611	1.133645906	0.117864998	0.004198696	0.02703492
PARG15616	-2.631154533	1.505363781	0.004215024	0.027117803
PARG24771	-1.80676593	-0.312473303	0.004235879	0.027236801
PARG29251	-1.894464779	0.485861473	0.004265862	0.027377556
PARG21173	-1.894190237	4.656804801	0.004265862	0.027377556
PARG16256	1.819840095	1.984482322	0.004265862	0.027377556
PARG00488	1.35236361	0.347271466	0.004277232	0.027439308
PARG00722	-1.157203384	-0.114895832	0.004395762	0.028096565
PARG19436	1.414122255	0.883382462	0.004447943	0.028372096
PARG17204	1.34285142	0.171081886	0.004745895	0.029932122
PARG24670	-1.328821507	0.651449076	0.004745895	0.029932122
PARG26646	-1.302777588	3.938474266	0.004745895	0.029932122
PARG16361	-1.173693353	7.503314518	0.0047701	0.030039418
PARG00136	-1.063267448	1.940688156	0.004777524	0.03005915
PARG11065	-1.483506184	2.368165134	0.00480784	0.030213558
PARG20380	1.247160516	-0.165333234	0.004901458	0.030705677
PARG13861	-1.203985162	0.876820839	0.004901458	0.030705677
PARG07588	-1.491791167	0.988269739	0.004994645	0.031212556
PARG27266	-2.374217425	-0.225877019	0.005043113	0.031378037
PARG18838	-2.358421001	-0.053692791	0.005043113	0.031378037
PARG10235	-2.352581241	0.289634746	0.005043113	0.031378037
PARG13646	-2.317087462	1.500446923	0.005043113	0.031378037
PARG02441	-1.222136689	5.529634432	0.00512764	0.031765467
PARG19361	-4.739959955	2.342442477	0.005170313	0.031878856
PARG01611	-4.658008454	-0.473754661	0.005170313	0.031878856
PARG08766	-4.61721475	1.979604014	0.005170313	0.031878856
PARG11404	-4.61721475	1.320399817	0.005170313	0.031878856
PARG02734	-4.612679357	-0.544815325	0.005170313	0.031878856
PARG11970	-4.606330567	1.73577138	0.005170313	0.031878856
PARG15346	-4.597752763	0.141010007	0.005170313	0.031878856
PARG07773	-4.595505664	0.272743401	0.005170313	0.031878856
PARG13320	-1.229929127	6.32575185	0.005194266	0.031983544
PARG10748	-1.118976716	2.071207502	0.005194266	0.031983544
PARG15195	-3.20149093	0.470033415	0.005209351	0.032018946
PARG00178	-3.101303293	-0.498816303	0.005209351	0.032018946
PARG11252	-1.009373336	2.818865797	0.005331618	0.032655382
PARG08335	-1.265005059	1.713437453	0.00536984	0.032838234
PARG18194	-1.343893734	3.209859983	0.005424611	0.033108689

PARG06673	1.111037806	0.668852055	0.005435099	0.033139822
PARG25061	1.069108749	0.671356119	0.005550654	0.033741164
PARG19039	-1.534271226	-0.118393149	0.005551881	0.033741164
PARG12547	1.05306195	2.911894606	0.0055669	0.033819347
PARG25180	-2.141899468	2.866277116	0.005571048	0.033831452
PARG05977	-1.004282461	2.139003445	0.005666101	0.034246701
PARG23815	1.20202257	0.649635682	0.0056994	0.034358362
PARG23387	-1.694438868	0.147053141	0.005720387	0.034424967
PARG17422	-1.683430723	0.485434411	0.005720387	0.034424967
PARG01031	1.672478164	0.855844975	0.005720387	0.034424967
PARG17939	-1.784349685	-0.065913794	0.005866704	0.035132667
PARG22817	-1.617954691	2.956874699	0.005866704	0.035132667
PARG14539	-1.017771848	1.522346512	0.005872603	0.035132667
PARG27071	-1.138678791	2.077924292	0.005894155	0.035221335
PARG17457	-1.218786019	2.976463862	0.005980746	0.035643792
PARG26921	-2.079392206	1.949575343	0.006011354	0.035773328
PARG07692	-1.818655884	0.419963519	0.006011354	0.035773328
PARG02335	-1.750528396	0.214193011	0.006034377	0.035854523
PARG11519	-1.129640155	1.497066312	0.006060269	0.03594035
PARG01639	-1.310143516	3.497476288	0.006206097	0.036639079
PARG26918	-1.27619134	3.8338281	0.006206097	0.036639079
PARG07604	-1.446045839	1.986059109	0.006228227	0.036742094
PARG24376	-1.421846	0.066971404	0.006228227	0.036742094
PARG19379	1.217709775	0.061919969	0.006283709	0.036986002
PARG02682	-1.163019441	1.32014359	0.006283709	0.036986002
PARG12372	-1.095415847	0.69434419	0.006343188	0.037210526
PARG02716	-1.02655666	3.037060448	0.006465046	0.037812332
PARG04021	-1.084936903	1.743665262	0.006475976	0.037862152
PARG02038	-1.403104638	0.409030581	0.006523271	0.038053628
PARG00717	-1.147324119	2.55351133	0.00680101	0.039453899
PARG26821	2.667187643	-0.374345346	0.006889411	0.039878306
PARG29673	1.104963506	0.653977994	0.007327604	0.042017598
PARG05765	1.09592177	0.767875678	0.007458437	0.04263749
PARG27534	-1.306573194	0.384544207	0.007632474	0.04352146
PARG00948	-1.501465589	3.46815319	0.007781299	0.04428964
PARG07898	-2.273313519	1.933686945	0.007916445	0.044847477
PARG21631	-2.269844633	3.478053336	0.007916445	0.044847477
PARG00264	-2.230448309	2.430837211	0.007916445	0.044847477
PARG21703	1.054401077	-0.686814319	0.007926949	0.044874595
PARG22675	-1.140878632	1.60570626	0.007942357	0.044929411
PARG21184	1.245671899	0.367056325	0.008202477	0.046134891
PARG06671	-1.594542036	-0.0588068	0.008357437	0.046860389
PARG07400	1.137781272	1.384426367	0.008388388	0.046961808
PARG01549	-1.016956269	-0.074542392	0.008416722	0.047036564
PARG15322	-2.003440877	0.27379451	0.008469227	0.047245885
PARG18195	-1.938560671	2.136054108	0.008469227	0.047245885

PARG13879	-1.312531155	2.03947338	0.00850406	0.047406512
PARG08096	-1.269646802	1.88179898	0.00850406	0.047406512
PARG08030	-1.696984772	0.995853793	0.008576896	0.047693985
PARG06316	-1.688799013	3.138170995	0.008576896	0.047693985
PARG10416	-1.218107115	0.151529648	0.008600753	0.047809711
PARG16133	-1.764720354	-0.053607243	0.008707282	0.048316334
PARG18100	1.657452722	-0.698021412	0.008707282	0.048316334
PARG16696	1.067690267	0.607769138	0.008762087	0.048603266
PARG13349	-1.040764979	1.499908998	0.008795741	0.048703893
PARG18374	1.040948995	2.5265921	0.00880252	0.048724244
PARG13996	3.115952444	0.729503173	0.008991581	0.04952626
PARG03375	-3.017852174	3.131087379	0.008991581	0.04952626

Table S16. The transcription factors involved in the regulation of carotenoid metabolism pathways

TF ID	Gene ID	Correlation coefficient	PValue
PARG00125	PARG01425	0.868957602	3.63E-08
PARG00125	PARG26029	0.887044357	7.74E-09
PARG00191	PARG21232	0.894170154	3.91E-09
PARG00570	PARG01425	-0.870356096	3.25E-08
PARG01916	PARG21232	0.855985944	9.63E-08
PARG01970	PARG21232	0.864162493	5.27E-08
PARG02040	PARG01425	-0.897500099	2.80E-09
PARG02040	PARG03050	-0.916788451	3.09E-10
PARG02040	PARG20809	-0.882810338	1.14E-08
PARG02040	PARG26029	-0.895775962	3.33E-09
PARG02071	PARG21232	0.859379957	7.53E-08
PARG02287	PARG01425	0.927327942	7.33E-11
PARG02287	PARG03050	0.926802287	7.92E-11
PARG02287	PARG16099	0.875379867	2.16E-08
PARG02287	PARG20809	0.933367516	2.90E-11
PARG02287	PARG26029	0.882570127	1.16E-08
PARG02307	PARG03050	-0.862524163	5.96E-08
PARG02718	PARG03050	-0.851641449	1.31E-07
PARG02898	PARG01425	-0.905985144	1.13E-09
PARG02898	PARG03050	-0.927481428	7.17E-11
PARG02898	PARG20809	-0.861106083	6.63E-08
PARG04578	PARG03050	-0.871574846	2.95E-08
PARG04972	PARG01425	-0.866427258	4.43E-08
PARG04972	PARG03050	-0.8566653	9.17E-08
PARG04972	PARG20809	-0.873100885	2.60E-08
PARG05751	PARG01425	-0.868680649	3.71E-08
PARG05751	PARG03050	-0.88927862	6.28E-09
PARG05751	PARG20809	-0.854463159	1.07E-07
PARG05751	PARG21232	0.876160905	2.02E-08
PARG06119	PARG21232	0.860405659	6.99E-08
PARG06319	PARG03050	-0.852729178	1.21E-07
PARG06319	PARG20809	-0.865098403	4.91E-08
PARG06621	PARG01425	-0.855313208	1.01E-07
PARG06621	PARG03050	-0.884308109	9.94E-09
PARG06628	PARG03050	-0.864377143	5.18E-08
PARG06644	PARG03050	-0.875309505	2.17E-08
PARG06644	PARG20809	-0.864523694	5.13E-08
PARG07177	PARG01425	0.898980597	2.40E-09
PARG07177	PARG03050	0.888548512	6.73E-09
PARG07177	PARG26029	0.893877974	4.03E-09
PARG07251	PARG01425	0.91232945	5.38E-10
PARG07251	PARG03050	0.895148859	3.55E-09
PARG07251	PARG11892	0.923870196	1.20E-10

PARG07251	PARG16099	0.911479926	5.96E-10
PARG07251	PARG26029	0.901146508	1.91E-09
PARG07570	PARG03050	-0.894935592	3.62E-09
PARG07570	PARG20809	-0.918788054	2.39E-10
PARG07604	PARG03050	-0.853581813	1.14E-07
PARG07644	PARG01425	-0.885502804	8.92E-09
PARG07644	PARG03050	-0.922995415	1.36E-10
PARG07644	PARG20809	-0.90129066	1.88E-09
PARG07767	PARG01425	0.86857115	3.75E-08
PARG07771	PARG20809	-0.878463971	1.66E-08
PARG07774	PARG01425	-0.851671745	1.30E-07
PARG07774	PARG03050	-0.878584494	1.64E-08
PARG07975	PARG21232	0.850017072	1.46E-07
PARG08415	PARG01425	0.913361793	4.74E-10
PARG08415	PARG03050	0.879100258	1.57E-08
PARG08415	PARG03227	0.885610774	8.83E-09
PARG08462	PARG01425	0.933905791	2.66E-11
PARG08462	PARG03050	0.946918912	2.54E-12
PARG08462	PARG16099	0.901639895	1.81E-09
PARG08462	PARG20809	0.956030755	3.34E-13
PARG08462	PARG26029	0.927452169	7.20E-11
PARG08477	PARG01425	-0.909734477	7.32E-10
PARG08477	PARG03050	-0.94982053	1.39E-12
PARG08477	PARG20809	-0.936438385	1.75E-11
PARG08477	PARG26029	-0.871065954	3.07E-08
PARG08702	PARG16099	-0.852068875	1.27E-07
PARG09157	PARG01425	-0.873735255	2.47E-08
PARG09157	PARG03050	-0.895032153	3.59E-09
PARG09157	PARG20809	-0.863309428	5.62E-08
PARG10614	PARG20809	-0.872600999	2.71E-08
PARG10668	PARG01425	0.947755256	2.14E-12
PARG10668	PARG03050	0.928700511	5.98E-11
PARG10668	PARG20809	0.905706314	1.16E-09
PARG10668	PARG26029	0.903383761	1.50E-09
PARG11298	PARG01425	0.919049486	2.31E-10
PARG11298	PARG03050	0.949938376	1.35E-12
PARG11298	PARG20809	0.869184571	3.57E-08
PARG11410	PARG20809	-0.869586968	3.46E-08
PARG12093	PARG03050	0.880745146	1.36E-08
PARG12093	PARG20809	0.879966408	1.46E-08
PARG12163	PARG03050	-0.879212345	1.56E-08
PARG12168	PARG16099	0.913967971	4.40E-10
PARG12168	PARG20809	0.866501757	4.40E-08
PARG12225	PARG20809	-0.869278165	3.54E-08
PARG12294	PARG01425	-0.878606201	1.64E-08
PARG12294	PARG03050	-0.853058111	1.18E-07

PARG12381	PARG01425	-0.870587759	3.19E-08
PARG12381	PARG03050	-0.902468828	1.66E-09
PARG12381	PARG20809	-0.869928765	3.36E-08
PARG12964	PARG01425	0.863969104	5.35E-08
PARG13086	PARG01425	0.891248527	5.20E-09
PARG13086	PARG03050	0.874725421	2.28E-08
PARG13086	PARG11892	0.937134546	1.56E-11
PARG13086	PARG16099	0.932690129	3.24E-11
PARG13086	PARG26029	0.894216559	3.89E-09
PARG14656	PARG21232	0.872142759	2.81E-08
PARG15170	PARG05251	0.85126248	1.34E-07
PARG15645	PARG01425	-0.88780082	7.21E-09
PARG15645	PARG03050	-0.893534295	4.16E-09
PARG15645	PARG20809	-0.877900667	1.74E-08
PARG15868	PARG21232	0.866476141	4.41E-08
PARG16111	PARG16099	-0.898742029	2.46E-09
PARG16289	PARG11892	0.863082143	5.72E-08
PARG17500	PARG03050	-0.863193441	5.67E-08
PARG17500	PARG20809	-0.899196013	2.35E-09
PARG17500	PARG21232	0.861430476	6.47E-08
PARG18366	PARG03050	-0.876191437	2.01E-08
PARG18494	PARG01425	-0.903091059	1.55E-09
PARG18494	PARG03050	-0.935361148	2.10E-11
PARG18494	PARG20809	-0.909157541	7.83E-10
PARG18718	PARG03050	-0.85611052	9.55E-08
PARG18925	PARG20809	-0.881799871	1.24E-08
PARG19103	PARG20809	-0.861079693	6.64E-08
PARG19109	PARG21232	0.85183301	1.29E-07
PARG19430	PARG01425	-0.921587879	1.65E-10
PARG19430	PARG03050	-0.923472346	1.27E-10
PARG19430	PARG05251	-0.855639069	9.87E-08
PARG19430	PARG20809	-0.890208288	5.75E-09
PARG19430	PARG26029	-0.90232208	1.68E-09
PARG19690	PARG01425	-0.850733951	1.39E-07
PARG19860	PARG01425	0.853167381	1.18E-07
PARG19860	PARG03050	0.872331834	2.77E-08
PARG19860	PARG26029	0.890108847	5.80E-09
PARG19959	PARG01425	-0.874872866	2.25E-08
PARG19959	PARG03050	-0.904576883	1.32E-09
PARG19959	PARG20809	-0.879703339	1.49E-08
PARG20240	PARG21232	0.879223555	1.56E-08
PARG20332	PARG03050	-0.857692756	8.52E-08
PARG20332	PARG20809	-0.907336445	9.66E-10
PARG21661	PARG01425	-0.878537681	1.65E-08
PARG21661	PARG03050	-0.909002808	7.97E-10
PARG21661	PARG20809	-0.879457109	1.53E-08

PARG21753	PARG01425	0.874003932	2.42E-08
PARG21753	PARG03050	0.868848479	3.66E-08
PARG21753	PARG20809	0.857534837	8.62E-08
PARG22369	PARG01425	-0.921129324	1.75E-10
PARG22369	PARG03050	-0.909802319	7.26E-10
PARG22369	PARG20809	-0.861366612	6.50E-08
PARG23424	PARG21232	0.86449276	5.14E-08
PARG23515	PARG01425	-0.856848189	9.05E-08
PARG23515	PARG03050	-0.878122855	1.71E-08
PARG24080	PARG20809	-0.892042444	4.82E-09
PARG24202	PARG21232	0.872494657	2.73E-08
PARG24203	PARG20809	-0.850615766	1.40E-07
PARG24308	PARG01425	-0.878547409	1.65E-08
PARG24308	PARG03050	-0.908528969	8.42E-10
PARG24308	PARG20809	-0.8670686	4.21E-08
PARG24385	PARG21232	0.858370875	8.11E-08
PARG24468	PARG03050	0.850018708	1.46E-07
PARG24469	PARG01425	-0.902140231	1.72E-09
PARG24469	PARG03050	-0.897153254	2.90E-09
PARG24469	PARG20809	-0.894098782	3.94E-09
PARG24470	PARG20808	0.886354961	8.25E-09
PARG24473	PARG20809	-0.873861242	2.45E-08
PARG24649	PARG03050	-0.860452862	6.96E-08
PARG24815	PARG03050	-0.860611462	6.88E-08
PARG24815	PARG21232	0.87177011	2.90E-08
PARG24857	PARG01425	0.945096275	3.65E-12
PARG24857	PARG03050	0.943104719	5.35E-12
PARG24857	PARG11892	0.85859494	7.98E-08
PARG24857	PARG16099	0.856777696	9.10E-08
PARG24857	PARG20809	0.888397453	6.82E-09
PARG24857	PARG26029	0.899091969	2.37E-09
PARG24983	PARG01425	-0.898468026	2.53E-09
PARG24983	PARG03050	-0.917087686	2.98E-10
PARG24983	PARG20809	-0.89114588	5.25E-09
PARG25241	PARG03050	-0.858694645	7.92E-08
PARG25402	PARG20809	-0.888542681	6.73E-09
PARG26144	PARG01425	-0.872528352	2.73E-08
PARG26144	PARG03050	-0.902286878	1.69E-09
PARG26144	PARG20809	-0.87866178	1.63E-08
PARG26667	PARG21232	0.917274117	2.91E-10
PARG26926	PARG21232	-0.861047761	6.66E-08
PARG27180	PARG01425	0.853740077	1.13E-07
PARG27180	PARG03050	0.851554367	1.32E-07
PARG27216	PARG03050	-0.850527686	1.41E-07
PARG27296	PARG03050	-0.897448353	2.81E-09
PARG27296	PARG20809	-0.858260861	8.17E-08

PARG27682	PARG21232	0.873263185	2.57E-08
PARG27982	PARG01425	-0.856852727	9.05E-08
PARG27982	PARG03050	-0.864397697	5.18E-08
PARG27982	PARG20809	-0.89128657	5.18E-09
PARG28413	PARG03050	-0.862499411	5.98E-08
PARG29184	PARG01425	0.854288949	1.09E-07
PARG29184	PARG03050	0.881217371	1.31E-08
PARG29184	PARG20808	0.869256007	3.55E-08
PARG29949	PARG01425	-0.865969657	4.59E-08
PARG29949	PARG03050	-0.877134773	1.86E-08

Table S17. Differentially expressed genes between the two cultivars 'Chuanzhihong' and 'Dabaixing'

Gene ID	logFC	logCPM	PValue	FDR
PARG1014	8.623536626	8.59979686	1.20E-118	1.88E-114
PARG2070	-14.00624122	6.200321447	1.62E-111	1.27E-107
PARG1741	8.185921984	6.380044618	1.59E-101	8.33E-98
PARG1979	8.889676582	4.798676613	1.89E-99	7.40E-96
PARG2227	-7.601886291	8.193930339	5.92E-98	1.86E-94
PARG0614	8.353483485	5.712872159	2.89E-97	7.55E-94
PARG0143	-10.0328431	4.805893126	1.86E-93	4.16E-90
PARG0682	7.880850449	5.690774773	1.92E-90	3.76E-87
PARG2711	8.581433825	4.064205682	5.51E-89	9.61E-86
PARG1969	-7.211308426	5.527498903	9.42E-89	1.48E-85
PARG0185	-6.990629473	7.391165706	4.97E-88	7.09E-85
PARG2877	6.68092587	8.459262788	1.16E-84	1.52E-81
PARG2819	-9.172142679	4.700716044	6.36E-84	7.68E-81
PARG1796	10.58893917	3.018838557	7.40E-83	8.30E-80
PARG2636	-8.521655111	4.673354853	1.72E-81	1.80E-78
PARG3043	-8.181096016	5.170001205	1.11E-80	1.09E-77
PARG2818	-12.19759378	4.047911641	9.28E-80	8.57E-77
PARG1905	8.331947195	3.656820119	1.04E-78	9.05E-76
PARG1603	-6.653322028	7.576130691	4.30E-76	3.55E-73
PARG3042	-7.649268541	4.903821989	3.65E-74	2.86E-71
PARG1051	-6.933227242	3.966200624	1.17E-73	8.55E-71
PARG2545	-7.838851302	4.755189931	1.20E-73	8.55E-71
PARG0406	-7.336857368	5.023205015	6.01E-71	4.10E-68
PARG1538	-5.982899426	5.290849828	2.25E-70	1.47E-67
PARG2837	-7.995789001	4.045152277	9.51E-69	5.97E-66
PARG2708	7.515987028	4.633833866	9.36E-67	5.65E-64
PARG2543	-5.593531133	6.832771838	7.55E-66	4.39E-63
PARG0117	-8.174840419	3.956716194	1.08E-65	6.07E-63
PARG2996	-5.919160078	4.283306634	4.19E-65	2.27E-62
PARG0689	5.77312961	5.594203155	6.35E-65	3.32E-62
PARG2705	-6.798908014	4.328216733	7.57E-62	3.83E-59
PARG1582	-6.666544862	4.246038108	1.12E-61	5.47E-59
PARG0750	8.200168591	4.036990507	1.68E-61	7.97E-59
PARG1827	7.359976666	3.019672664	1.07E-59	4.90E-57
PARG2158	-10.99992378	2.972935524	1.09E-59	4.90E-57
PARG0161	-6.661947035	3.710601316	1.41E-59	6.13E-57
PARG0143	-8.575239935	2.84065974	3.25E-59	1.38E-56
PARG0466	-7.606488786	3.476185422	3.98E-59	1.64E-56
PARG1374	7.069291448	4.565646275	5.37E-59	2.16E-56
PARG1861	6.097847169	3.706026946	1.20E-58	4.70E-56
PARG2780	5.740077544	6.06172814	1.56E-57	5.99E-55
PARG2643	-6.369278126	2.872187525	2.54E-57	9.49E-55
PARG1322	5.611506373	4.983713218	3.53E-57	1.29E-54

PARG1243	-10.8328372	1.884659778	5.08E-57	1.81E-54
PARG1650	8.278995005	4.35752533	1.26E-56	4.39E-54
PARG2250	-5.958155016	4.792801719	2.82E-56	9.62E-54
PARG0809	5.152687019	6.065422994	1.52E-55	5.09E-53
PARG1768	-8.94710416	3.749223685	6.24E-55	2.04E-52
PARG1098	5.679618297	3.864003578	1.66E-54	5.32E-52
PARG2495	7.516571096	3.130617294	1.72E-54	5.40E-52
PARG1902	-4.795748134	8.981044124	4.03E-54	1.24E-51
PARG0401	-7.851653276	3.250830196	5.70E-54	1.72E-51
PARG1582	-8.863770239	2.841904161	1.35E-53	3.99E-51
PARG1611	-10.61251673	3.226894822	1.64E-53	4.75E-51
PARG1000	-8.088846433	1.884377916	2.33E-52	6.66E-50
PARG1420	-6.67470258	3.655148511	4.15E-52	1.16E-49
PARG2771	-6.499731577	2.284685028	1.57E-51	4.32E-49
PARG0417	-10.47501945	3.071390353	2.24E-51	6.07E-49
PARG0142	-7.008135894	3.423770528	2.29E-51	6.08E-49
PARG0234	6.64360318	2.242027983	6.09E-51	1.59E-48
PARG2727	-5.210635912	4.07986731	6.13E-50	1.58E-47
PARG1651	6.536173817	4.519420844	3.76E-49	9.51E-47
PARG1131	-6.607884475	3.304737113	4.85E-49	1.21E-46
PARG2899	-5.706226534	4.131926051	1.06E-48	2.61E-46
PARG2619	7.772325383	3.163293189	8.26E-48	2.00E-45
PARG0886	-7.78323503	2.170482474	1.35E-47	3.21E-45
PARG2328	-6.411492679	2.912684579	1.39E-47	3.25E-45
PARG2212	-4.985574061	5.654338841	3.12E-47	7.20E-45
PARG0447	-4.686254743	4.194383578	4.23E-47	9.63E-45
PARG0736	6.183611043	2.585264492	5.94E-47	1.33E-44
PARG1488	-7.336642479	4.056568702	8.72E-47	1.93E-44
PARG0340	-4.583572258	4.205965904	9.44E-47	2.06E-44
PARG2100	-4.786369156	3.387538715	3.53E-46	7.59E-44
PARG1429	-8.266567012	2.992003525	6.03E-46	1.28E-43
PARG0466	-7.235997859	2.993405619	9.29E-46	1.94E-43
PARG2979	-5.213797102	3.345761609	2.68E-45	5.53E-43
PARG1500	6.223173946	3.619398694	2.97E-45	6.06E-43
PARG2994	5.543060812	2.691395337	6.15E-45	1.24E-42
PARG2073	7.10688302	2.138174516	2.54E-44	5.04E-42
PARG2319	-7.679187486	2.526953675	3.23E-44	6.35E-42
PARG0685	4.599889183	4.416879172	7.46E-44	1.45E-41
PARG0247	9.971201619	2.208749922	8.02E-44	1.54E-41
PARG2613	-6.382634889	2.379692095	1.20E-43	2.27E-41
PARG0388	-4.770405458	4.950435139	1.33E-43	2.49E-41
PARG0384	-4.434533526	4.937792366	2.18E-43	4.03E-41
PARG2342	-4.56152701	6.600164939	2.63E-43	4.81E-41
PARG0535	6.531417112	2.681245544	9.30E-43	1.68E-40
PARG1476	-5.571854793	3.339377917	6.08E-42	1.09E-39
PARG2839	-4.371968823	5.26034164	6.71E-42	1.18E-39

PARG2319	-4.780781498	6.512571637	9.72E-42	1.69E-39
PARG1630	6.889331039	2.500678935	1.06E-41	1.83E-39
PARG1878	6.400903435	2.373888705	1.35E-41	2.31E-39
PARG0301	-8.057871312	2.010561491	2.32E-41	3.92E-39
PARG2227	4.107630228	10.62900828	3.45E-41	5.76E-39
PARG2584	-9.790016739	2.245865834	3.66E-41	6.05E-39
PARG1048	6.574763879	2.468195096	9.30E-41	1.52E-38
PARG1798	-9.756932315	0.657213728	1.16E-40	1.88E-38
PARG2008	4.243051134	3.806816755	1.73E-40	2.77E-38
PARG1837	4.217454796	3.427901061	3.08E-40	4.88E-38
PARG0268	-6.214400672	3.424955099	4.74E-40	7.44E-38
PARG0734	4.254232333	4.355510217	5.42E-40	8.43E-38
PARG1135	-4.727622491	2.685020612	1.14E-39	1.75E-37
PARG2216	-5.857010262	2.113299356	1.25E-39	1.90E-37
PARG0452	-6.286654427	3.435494494	1.69E-39	2.55E-37
PARG0509	-9.671867561	2.180447271	1.71E-39	2.56E-37
PARG1738	-6.785403372	3.305882326	3.94E-39	5.84E-37
PARG3040	-7.447853319	1.855007414	5.21E-39	7.64E-37
PARG0901	-4.097886461	5.110836118	5.29E-39	7.69E-37
PARG0661	5.597766262	4.888758631	5.73E-39	8.26E-37
PARG0134	-6.457061119	2.297009156	1.04E-38	1.48E-36
PARG1718	-6.21886757	2.620539966	1.63E-38	2.30E-36
PARG2213	-5.734975544	2.163063113	2.19E-38	3.07E-36
PARG1074	4.705715493	2.071207502	2.29E-38	3.18E-36
PARG0412	6.041066099	1.841312032	2.75E-38	3.78E-36
PARG0116	-6.719073939	2.152073866	4.26E-38	5.82E-36
PARG2931	-7.804896211	1.707494183	8.78E-38	1.19E-35
PARG0527	-6.395742644	3.264149108	9.16E-38	1.23E-35
PARG1110	3.799730828	7.733607307	9.26E-38	1.23E-35
PARG2270	-3.931047686	4.068749086	1.03E-37	1.36E-35
PARG1089	-6.384287672	2.899246487	1.19E-37	1.56E-35
PARG1135	-6.68532083	1.120361117	1.27E-37	1.64E-35
PARG0137	-4.84580836	6.706338755	2.61E-37	3.36E-35
PARG0237	4.743403417	3.333630102	9.03E-37	1.15E-34
PARG2483	3.908804224	3.733222342	1.17E-36	1.48E-34
PARG1953	-3.725561244	7.017536159	1.23E-36	1.54E-34
PARG1512	-4.245072631	4.151665647	3.20E-36	3.98E-34
PARG2240	4.785116637	4.002692549	7.77E-36	9.61E-34
PARG0490	3.74848903	6.898835134	1.44E-35	1.77E-33
PARG2051	-6.526564447	2.709358594	1.90E-35	2.31E-33
PARG1096	3.93874986	3.518411031	2.77E-35	3.35E-33
PARG2710	-7.613383538	1.434436033	3.85E-35	4.62E-33
PARG2107	4.064436665	4.904593851	4.54E-35	5.40E-33
PARG0044	-6.435799119	2.617482207	5.45E-35	6.44E-33
PARG1407	-4.289891432	3.701046554	8.48E-35	9.93E-33
PARG0051	5.469285298	4.049504603	8.76E-35	1.02E-32

PARG0716	3.729792487	6.12664434	9.56E-35	1.10E-32
PARG1374	6.57296895	2.780092692	1.86E-34	2.13E-32
PARG2403	-4.492404039	2.610187676	2.17E-34	2.47E-32
PARG2078	-4.706425144	5.823186706	2.23E-34	2.52E-32
PARG0479	-5.128062968	2.780013016	3.36E-34	3.77E-32
PARG3001	-4.636110059	1.420734032	3.50E-34	3.90E-32
PARG0779	5.234175273	1.318598829	5.35E-34	5.91E-32
PARG2564	-3.660397716	8.278113323	5.67E-34	6.22E-32
PARG1824	5.672451135	1.543808072	9.47E-34	1.03E-31
PARG1666	5.285558015	2.16033694	2.43E-33	2.63E-31
PARG1901	5.86872256	3.202424718	2.54E-33	2.73E-31
PARG2890	-7.466159203	1.289066319	3.64E-33	3.87E-31
PARG0115	-7.461467029	2.016338875	3.64E-33	3.87E-31
PARG2092	5.840029072	1.691814816	6.00E-33	6.32E-31
PARG2796	4.279376511	2.984805841	6.42E-33	6.72E-31
PARG1811	-4.319522373	3.201891588	7.06E-33	7.34E-31
PARG1842	-3.471995519	9.974123612	7.51E-33	7.75E-31
PARG2832	3.67447636	4.865671819	1.03E-32	1.05E-30
PARG1584	-3.80261314	6.148233936	1.77E-32	1.80E-30
PARG2683	4.99873415	1.238870441	2.53E-32	2.57E-30
PARG1084	-3.801426835	6.149281726	3.07E-32	3.09E-30
PARG3039	-6.433582495	1.887596779	3.10E-32	3.10E-30
PARG0876	4.819955071	3.620156537	3.59E-32	3.57E-30
PARG2748	-5.990207783	2.914600893	3.65E-32	3.60E-30
PARG1821	-3.913689979	3.577824469	4.91E-32	4.79E-30
PARG0137	-7.058066253	1.518799783	4.91E-32	4.79E-30
PARG2121	3.419377609	6.500826314	8.29E-32	8.03E-30
PARG0569	-9.078751707	1.729877368	1.46E-31	1.40E-29
PARG0453	3.615033805	4.44566917	2.55E-31	2.44E-29
PARG2688	7.272762781	0.904626335	4.11E-31	3.91E-29
PARG2676	-4.451992277	2.834251006	4.16E-31	3.94E-29
PARG1414	3.571651631	3.347050823	4.72E-31	4.44E-29
PARG1942	-4.135072524	2.924603592	6.23E-31	5.82E-29
PARG2640	-4.203599156	1.716299788	6.30E-31	5.85E-29
PARG0201	-5.192956932	4.796420823	7.14E-31	6.60E-29
PARG0362	-6.174265248	1.026378809	8.98E-31	8.24E-29
PARG0223	-7.277912828	0.937267227	9.08E-31	8.29E-29
PARG1338	-9.005923595	1.469329371	1.20E-30	1.08E-28
PARG2459	-9.002423078	1.635293725	1.20E-30	1.08E-28
PARG1551	-8.997148434	1.196396042	1.49E-30	1.33E-28
PARG0027	-6.565297491	1.62329708	2.14E-30	1.91E-28
PARG0288	-3.40647439	4.256543409	2.58E-30	2.28E-28
PARG1685	-6.559674171	3.30208086	2.59E-30	2.29E-28
PARG1592	3.356616906	7.28542008	3.25E-30	2.85E-28
PARG0573	-4.320129458	1.658027168	3.56E-30	3.10E-28
PARG2631	3.546803148	4.92796454	4.50E-30	3.90E-28

PARG0457	3.277895647	8.289891445	6.47E-30	5.58E-28
PARG1271	-3.284642717	5.734228816	9.78E-30	8.39E-28
PARG0061	3.346773872	6.106174931	1.16E-29	9.88E-28
PARG1567	-6.487090291	0.385207212	1.81E-29	1.54E-27
PARG2640	-8.900442243	1.421549017	2.09E-29	1.77E-27
PARG0698	-3.317920888	5.592307982	2.95E-29	2.48E-27
PARG1604	4.489794232	3.438220242	4.71E-29	3.93E-27
PARG0184	-5.46268289	1.549595649	1.73E-28	1.44E-26
PARG1680	3.874647518	3.59461025	1.84E-28	1.52E-26
PARG2320	3.434339674	4.734893732	2.28E-28	1.87E-26
PARG2950	-3.468680869	5.190077784	2.28E-28	1.87E-26
PARG0356	-3.48635706	8.089594688	2.37E-28	1.93E-26
PARG1253	-3.178035219	7.331987724	2.57E-28	2.08E-26
PARG1200	-3.755999227	7.060363756	3.42E-28	2.75E-26
PARG1653	3.780614599	3.377114092	4.56E-28	3.65E-26
PARG1762	3.976572264	2.828091876	7.44E-28	5.93E-26
PARG1107	3.590328798	2.138173042	9.02E-28	7.15E-26
PARG0907	-3.857996706	2.414941352	1.29E-27	1.02E-25
PARG1957	4.418813693	3.928818032	1.99E-27	1.56E-25
PARG1265	4.126456838	2.941592808	2.28E-27	1.78E-25
PARG1000	-3.512875511	4.508410345	2.44E-27	1.90E-25
PARG0671	4.059741891	3.132696454	2.60E-27	2.01E-25
PARG3035	-6.185932989	1.218149598	3.90E-27	3.00E-25
PARG1003	-8.700811763	0.663724087	4.72E-27	3.60E-25
PARG2213	-8.700325883	0.350717365	4.72E-27	3.60E-25
PARG0685	3.232480151	4.000069195	5.25E-27	3.98E-25
PARG0880	-3.274743925	4.598687261	5.98E-27	4.51E-25
PARG1852	3.685737322	4.900813778	7.94E-27	5.96E-25
PARG0600	-6.949597373	1.067146214	9.44E-27	7.05E-25
PARG2159	3.293386893	4.916077229	1.19E-26	8.83E-25
PARG0341	-3.065753106	6.927963462	1.28E-26	9.47E-25
PARG0698	3.142330609	4.547307335	1.31E-26	9.66E-25
PARG2310	-3.644363388	3.436383888	2.39E-26	1.75E-24
PARG1660	4.053410785	2.583306097	2.92E-26	2.13E-24
PARG2297	3.163762468	4.335006193	3.83E-26	2.78E-24
PARG0740	3.221636077	7.079841328	4.06E-26	2.94E-24
PARG0577	-3.241601096	7.122068845	4.92E-26	3.54E-24
PARG0862	-3.678119213	4.281527013	5.05E-26	3.62E-24
PARG2382	3.305142645	4.316701143	5.89E-26	4.20E-24
PARG2921	3.015949098	5.954379538	6.61E-26	4.70E-24
PARG1937	3.005037679	6.273275777	8.19E-26	5.79E-24
PARG1096	3.550341872	2.833029357	8.27E-26	5.80E-24
PARG2560	-2.992721384	9.412192417	8.28E-26	5.80E-24
PARG0556	3.686662537	1.925765889	1.43E-25	9.96E-24
PARG1730	5.429653486	2.108102243	1.73E-25	1.20E-23
PARG0042	3.232692473	3.547762591	3.05E-25	2.11E-23

PARG1542	6.779045871	1.713535111	3.43E-25	2.36E-23
PARG0772	-4.296981379	4.237677151	3.81E-25	2.61E-23
PARG2730	-3.322213601	2.925176102	4.64E-25	3.17E-23
PARG2032	8.523982804	0.601764474	4.76E-25	3.24E-23
PARG1277	-3.336390279	3.992947033	5.02E-25	3.40E-23
PARG2154	-2.935395627	10.84875741	5.23E-25	3.53E-23
PARG2237	-6.796889148	0.685003499	5.64E-25	3.78E-23
PARG0246	-5.413001351	1.963252816	6.13E-25	4.09E-23
PARG1276	4.09506696	1.263111968	8.45E-25	5.62E-23
PARG0001	5.202748656	3.131287191	8.68E-25	5.75E-23
PARG1110	3.058366819	6.835694752	8.83E-25	5.82E-23
PARG0455	4.947835766	4.799962061	1.29E-24	8.46E-23
PARG1374	6.342243729	1.678951676	1.42E-24	9.30E-23
PARG0299	2.946342819	5.472905189	1.73E-24	1.13E-22
PARG2500	5.32937728	2.701380384	1.79E-24	1.16E-22
PARG1543	3.868150612	1.299895158	1.88E-24	1.21E-22
PARG0908	3.290512912	6.759129309	2.04E-24	1.31E-22
PARG1371	4.232197535	1.432478477	2.40E-24	1.54E-22
PARG0347	-3.13001631	4.327993129	2.41E-24	1.54E-22
PARG1668	-3.057602764	7.408399036	2.78E-24	1.77E-22
PARG2534	-3.186042669	2.586147262	3.39E-24	2.14E-22
PARG2959	-5.704425573	1.378517071	3.39E-24	2.14E-22
PARG0598	3.094038432	3.18177694	3.41E-24	2.14E-22
PARG1199	2.924623521	5.222067991	3.70E-24	2.32E-22
PARG1105	-3.641893772	1.467023684	4.13E-24	2.57E-22
PARG0188	-2.87761307	7.662102051	5.91E-24	3.67E-22
PARG0614	2.912337592	6.616450683	8.81E-24	5.44E-22
PARG2438	3.877168158	1.558284485	9.30E-24	5.72E-22
PARG2871	-8.408481335	1.023587597	9.62E-24	5.90E-22
PARG1666	3.098247682	3.926347675	1.77E-23	1.08E-21
PARG2333	-4.897761476	2.946671181	1.78E-23	1.09E-21
PARG0751	2.891908735	5.599638533	1.86E-23	1.13E-21
PARG2683	3.070586124	3.313123751	2.07E-23	1.25E-21
PARG1838	-2.836071093	5.932420614	2.45E-23	1.47E-21
PARG2920	4.429811413	5.486358705	2.85E-23	1.71E-21
PARG2671	3.061529656	2.541034271	3.19E-23	1.91E-21
PARG0895	4.238899374	0.474919967	3.44E-23	2.05E-21
PARG1717	-2.876615585	5.331155213	4.82E-23	2.86E-21
PARG2367	-5.941762367	0.693918271	5.66E-23	3.34E-21
PARG2565	-3.105134846	3.653663327	5.76E-23	3.38E-21
PARG1237	-4.310704649	2.518452572	5.77E-23	3.38E-21
PARG0878	-4.582143645	0.445186798	6.29E-23	3.67E-21
PARG2032	4.108926376	1.22210512	7.30E-23	4.24E-21
PARG1259	3.0939563	2.322825645	7.56E-23	4.38E-21
PARG2293	3.260512648	2.360514327	8.19E-23	4.73E-21
PARG2564	-3.616597558	5.370977861	8.92E-23	5.13E-21

PARG2241	-3.483953642	3.821210816	9.57E-23	5.48E-21
PARG0109	3.844163261	0.757397844	1.03E-22	5.90E-21
PARG1377	2.812691307	8.142022613	1.06E-22	6.02E-21
PARG2250	-3.72172562	1.399026837	1.17E-22	6.61E-21
PARG1204	-3.202463742	2.537448315	1.55E-22	8.75E-21
PARG2720	-3.360766337	1.201723664	1.64E-22	9.24E-21
PARG1678	-4.053778377	0.98938218	1.73E-22	9.70E-21
PARG1640	-2.819516373	5.160648783	2.23E-22	1.25E-20
PARG0063	-2.741833931	9.083752196	2.71E-22	1.51E-20
PARG2254	2.805733063	4.584814588	3.36E-22	1.86E-20
PARG1427	-6.536328067	0.716291692	4.03E-22	2.23E-20
PARG1835	2.726836711	7.274809404	5.28E-22	2.91E-20
PARG1863	4.957787888	0.088571309	5.34E-22	2.93E-20
PARG0309	2.849580081	4.307860963	5.40E-22	2.95E-20
PARG2945	8.231297666	0.976478385	5.57E-22	3.02E-20
PARG1831	4.041472745	2.524247231	5.57E-22	3.02E-20
PARG2818	-4.037785444	3.297524396	6.56E-22	3.55E-20
PARG0178	2.867158321	3.863284164	7.71E-22	4.16E-20
PARG0581	4.952179326	1.616514333	8.28E-22	4.45E-20
PARG1298	-5.41429751	2.666194093	8.48E-22	4.54E-20
PARG2508	6.466672977	0.944650296	9.32E-22	4.97E-20
PARG1710	2.834709	3.771329237	9.34E-22	4.97E-20
PARG1427	-3.162797393	3.151616285	9.75E-22	5.17E-20
PARG1736	-4.199193308	1.910340825	1.26E-21	6.65E-20
PARG1915	3.20716535	3.503578704	1.32E-21	6.93E-20
PARG1261	-3.988924858	0.308549717	2.16E-21	1.13E-19
PARG2352	8.159320604	0.478904987	2.54E-21	1.33E-19
PARG2270	-3.059664819	3.142803127	2.86E-21	1.49E-19
PARG0919	-2.672071585	6.492783954	2.88E-21	1.50E-19
PARG2817	-2.899401791	2.945030981	2.98E-21	1.54E-19
PARG2832	3.564600197	3.380321358	3.17E-21	1.64E-19
PARG2992	-5.131135074	-0.001516174	3.59E-21	1.85E-19
PARG3001	2.927750405	3.638798857	4.23E-21	2.17E-19
PARG1831	4.314132052	0.562743734	4.40E-21	2.25E-19
PARG1669	-3.58800138	2.462167421	6.92E-21	3.53E-19
PARG2494	-3.045666747	4.892019064	8.56E-21	4.35E-19
PARG2384	-3.306147888	6.565365764	9.50E-21	4.81E-19
PARG0609	2.852944589	7.023772119	9.96E-21	5.03E-19
PARG2747	-2.628178386	7.05576164	1.09E-20	5.51E-19
PARG0235	2.95455089	3.291666737	1.25E-20	6.29E-19
PARG1481	3.4708409	1.739132385	1.36E-20	6.81E-19
PARG2435	3.612984642	2.332969445	1.42E-20	7.10E-19
PARG2267	-2.652553319	6.067484441	1.81E-20	8.97E-19
PARG1536	-2.657442259	5.613955293	1.84E-20	9.09E-19
PARG1889	3.394026792	3.130795196	1.94E-20	9.57E-19
PARG0344	3.063338104	2.964899156	2.44E-20	1.20E-18

PARG2400	3.275931924	3.532653923	2.52E-20	1.23E-18
PARG1741	6.319610605	-0.082893113	3.02E-20	1.48E-18
PARG2218	-2.633083664	6.179823005	3.34E-20	1.63E-18
PARG2839	-6.331127576	0.67314326	4.08E-20	1.98E-18
PARG1461	3.218396184	2.080165774	4.87E-20	2.36E-18
PARG1054	3.195242642	6.679984331	5.25E-20	2.54E-18
PARG1251	-2.68411681	4.431019247	5.69E-20	2.74E-18
PARG2082	-2.971462464	1.932415879	6.85E-20	3.29E-18
PARG0552	-3.508642784	0.779498962	7.56E-20	3.62E-18
PARG0081	-5.637312276	0.491028868	8.05E-20	3.84E-18
PARG1853	3.074570892	6.652379432	8.92E-20	4.24E-18
PARG2494	-3.242231825	1.899462354	9.02E-20	4.28E-18
PARG1046	-2.871679758	3.344289936	9.31E-20	4.40E-18
PARG0083	2.833527381	3.449873743	1.13E-19	5.34E-18
PARG1743	7.98811697	-0.002393767	1.15E-19	5.41E-18
PARG2211	-5.613647225	1.001584079	1.43E-19	6.70E-18
PARG0588	2.582588498	5.458240203	1.56E-19	7.29E-18
PARG0827	-2.645844635	4.636065086	1.57E-19	7.30E-18
PARG0585	-7.977143613	-0.100043934	1.60E-19	7.43E-18
PARG0330	-2.571248315	6.720702812	2.03E-19	9.38E-18
PARG0976	3.084238838	4.740036724	2.40E-19	1.11E-17
PARG0247	-2.673083613	4.175265458	2.51E-19	1.16E-17
PARG1657	-6.250268403	0.252705833	2.53E-19	1.16E-17
PARG1901	4.333724567	3.881608208	2.71E-19	1.24E-17
PARG1112	-2.512845469	7.819021885	2.98E-19	1.36E-17
PARG2318	-4.50003785	2.309450878	3.34E-19	1.52E-17
PARG0383	-2.523430836	5.475141842	3.39E-19	1.54E-17
PARG0087	-6.239590034	0.105016873	3.45E-19	1.56E-17
PARG2959	-2.820398944	3.564456531	3.65E-19	1.65E-17
PARG2636	-5.158288278	3.054133969	3.69E-19	1.66E-17
PARG2078	-2.501798186	7.343904367	4.02E-19	1.80E-17
PARG0000	-3.260058793	4.717016027	5.15E-19	2.30E-17
PARG0862	3.1418134	4.420164471	5.64E-19	2.52E-17
PARG1243	-6.70015525	0.353352364	6.08E-19	2.70E-17
PARG2537	6.183871648	0.760183897	6.45E-19	2.86E-17
PARG0857	-2.629178767	5.670241332	7.10E-19	3.14E-17
PARG0881	-3.714505099	1.931738624	7.14E-19	3.15E-17
PARG1424	2.669590695	5.768277729	7.30E-19	3.21E-17
PARG1362	-3.58561223	0.787046009	7.44E-19	3.26E-17
PARG1244	3.316476247	2.006995125	8.46E-19	3.70E-17
PARG2954	-6.197128607	0.323209206	8.84E-19	3.85E-17
PARG2452	-3.336253087	1.989019402	9.44E-19	4.10E-17
PARG1212	2.650423023	5.236736497	9.71E-19	4.21E-17
PARG1412	-2.505645825	7.497029012	1.01E-18	4.35E-17
PARG2983	-7.896623321	-0.317046304	1.20E-18	5.15E-17
PARG0268	-7.883937335	3.495725319	1.20E-18	5.15E-17

PARG0227	-2.457970819	7.232505963	1.25E-18	5.36E-17
PARG2711	4.839243958	-0.057470765	1.34E-18	5.72E-17
PARG0589	2.728253401	2.143620605	1.36E-18	5.82E-17
PARG0283	2.462822756	5.716504285	1.47E-18	6.26E-17
PARG0603	2.628028545	2.793905715	1.56E-18	6.61E-17
PARG2091	-6.166930512	0.210372914	1.67E-18	7.06E-17
PARG1134	-2.449651616	6.390015649	1.99E-18	8.40E-17
PARG1872	-5.093733848	1.168376587	2.00E-18	8.40E-17
PARG2452	-4.26437696	-0.018209345	2.40E-18	1.00E-16
PARG2324	4.198549823	2.686559051	2.40E-18	1.00E-16
PARG2726	-2.548492494	6.227942537	2.55E-18	1.07E-16
PARG1134	-3.189224594	5.318151451	2.60E-18	1.08E-16
PARG1542	5.480780717	1.208654585	2.74E-18	1.14E-16
PARG1555	2.428978015	9.153732941	2.77E-18	1.15E-16
PARG2237	-2.477480999	5.87945793	2.89E-18	1.19E-16
PARG1166	-2.784082797	2.977620449	3.46E-18	1.43E-16
PARG0086	-2.572288773	5.320614687	3.80E-18	1.56E-16
PARG0946	-2.430591412	7.031820424	3.93E-18	1.61E-16
PARG1611	-7.831587379	0.229620849	4.77E-18	1.95E-16
PARG0700	-3.096020272	1.226705753	5.15E-18	2.10E-16
PARG0851	2.883060198	2.000972854	5.27E-18	2.14E-16
PARG0325	-3.337350076	1.610232915	5.61E-18	2.28E-16
PARG1896	4.13805906	3.192804626	5.85E-18	2.37E-16
PARG2947	-2.409985155	7.401220255	5.94E-18	2.40E-16
PARG2837	-6.109053402	0.688922132	6.06E-18	2.44E-16
PARG2438	2.409801075	7.549189453	6.52E-18	2.62E-16
PARG0577	3.610313975	2.34292414	6.57E-18	2.63E-16
PARG2633	7.799150205	0.560126268	6.77E-18	2.70E-16
PARG2237	7.797546084	0.570563339	6.77E-18	2.70E-16
PARG2444	2.921826131	1.979701813	6.87E-18	2.73E-16
PARG1532	4.710188986	0.27379451	6.91E-18	2.74E-16
PARG2810	-2.588382039	2.188905858	7.15E-18	2.83E-16
PARG0285	-3.799439535	0.650683062	7.77E-18	3.07E-16
PARG1399	3.8088731	0.729503173	8.06E-18	3.17E-16
PARG1998	3.840572539	1.791389487	8.21E-18	3.22E-16
PARG2782	3.395310382	1.135639114	8.26E-18	3.23E-16
PARG0708	-4.74938715	0.181952394	9.12E-18	3.56E-16
PARG2696	-4.526542268	0.474093995	1.18E-17	4.60E-16
PARG0666	2.511167482	8.521730883	1.46E-17	5.66E-16
PARG0693	6.025682906	-0.374509139	1.62E-17	6.29E-16
PARG0532	-2.40265272	4.863539609	1.74E-17	6.71E-16
PARG0788	-3.687844601	3.175032902	1.86E-17	7.14E-16
PARG2296	-2.398090678	5.454931849	1.86E-17	7.14E-16
PARG1045	-3.324776248	0.433868935	1.95E-17	7.50E-16
PARG0987	7.749168068	0.621799512	1.96E-17	7.51E-16
PARG1830	3.430974374	1.40448012	2.03E-17	7.74E-16

PARG0369	2.920565175	1.866361959	2.22E-17	8.47E-16
PARG2144	4.648926378	0.643599196	2.81E-17	1.07E-15
PARG1545	-3.137436386	0.794127406	2.85E-17	1.08E-15
PARG1250	-3.728188679	1.822178543	2.88E-17	1.09E-15
PARG1603	3.729148679	2.042097083	3.27E-17	1.24E-15
PARG1260	3.066954959	0.995776621	3.67E-17	1.38E-15
PARG0418	-7.717079127	0.406578862	4.03E-17	1.51E-15
PARG1881	-3.711243851	1.052931412	4.40E-17	1.65E-15
PARG0316	-4.45761059	0.426586445	4.57E-17	1.71E-15
PARG1192	2.514827515	2.136964314	4.67E-17	1.74E-15
PARG0507	2.365540543	7.812692107	5.26E-17	1.96E-15
PARG0842	-7.695045776	0.426345926	5.79E-17	2.14E-15
PARG2276	-7.693659833	-0.074190529	5.79E-17	2.14E-15
PARG0400	-7.692455008	0.45168009	5.79E-17	2.14E-15
PARG2147	6.14801863	0.451679808	6.20E-17	2.28E-15
PARG2764	-2.516080732	3.796590972	6.20E-17	2.28E-15
PARG0491	2.561621193	4.786502048	6.44E-17	2.36E-15
PARG1716	4.236633641	1.426700771	6.76E-17	2.47E-15
PARG0234	3.866890796	0.28659391	7.08E-17	2.58E-15
PARG1622	-2.375641336	7.279270576	8.09E-17	2.95E-15
PARG1557	2.532695872	4.922628323	9.24E-17	3.36E-15
PARG1893	-4.128616599	1.435765864	9.33E-17	3.38E-15
PARG0733	-2.462961186	2.51798488	1.21E-16	4.35E-15
PARG1680	7.649701954	0.207029684	1.21E-16	4.35E-15
PARG1918	2.52589762	4.243953774	1.24E-16	4.46E-15
PARG1654	-2.332300574	6.312655662	1.43E-16	5.13E-15
PARG1666	2.390242902	4.352097448	1.47E-16	5.26E-15
PARG1053	-2.300176814	6.602181242	1.52E-16	5.43E-15
PARG1807	-2.336593163	4.740433457	1.52E-16	5.43E-15
PARG0785	2.301964231	5.891381587	1.56E-16	5.54E-15
PARG1046	-2.487386764	3.254099872	1.71E-16	6.06E-15
PARG0344	-7.629126068	-0.140711758	1.75E-16	6.18E-15
PARG1663	3.657497729	3.181628697	1.82E-16	6.42E-15
PARG2308	-4.272604134	0.958331114	1.96E-16	6.91E-15
PARG0095	-2.291004622	6.916034472	1.97E-16	6.93E-15
PARG1820	2.361822417	4.064493402	2.17E-16	7.63E-15
PARG2749	-2.490441564	3.911735753	2.21E-16	7.75E-15
PARG2399	5.240223003	1.109511349	2.22E-16	7.75E-15
PARG2993	5.885459066	-0.365149678	2.44E-16	8.51E-15
PARG2011	-7.62989752	-0.437166642	2.53E-16	8.80E-15
PARG2016	-7.615790865	0.511315318	2.53E-16	8.80E-15
PARG1999	-2.628656241	3.437063449	2.55E-16	8.82E-15
PARG1889	2.481078652	4.122425445	2.57E-16	8.88E-15
PARG2938	-2.417315475	3.428851388	3.14E-16	1.08E-14
PARG0126	2.353969549	4.311839986	3.16E-16	1.09E-14
PARG0878	-2.40028655	3.622446226	3.18E-16	1.09E-14

PARG0415	-3.33917654	1.291625276	3.33E-16	1.14E-14
PARG3040	-5.90890837	0.147382014	3.46E-16	1.18E-14
PARG0413	-2.419579044	5.223573305	3.58E-16	1.22E-14
PARG0881	-3.079801206	1.872881356	3.67E-16	1.25E-14
PARG0876	7.602552795	1.363656188	3.68E-16	1.25E-14
PARG2980	2.254577549	7.247023953	3.89E-16	1.32E-14
PARG1275	-2.45029325	3.118209281	4.40E-16	1.49E-14
PARG1570	-2.255797362	6.270926396	4.68E-16	1.58E-14
PARG1376	2.420858319	4.940683828	5.30E-16	1.79E-14
PARG0835	-2.245511915	7.662705228	5.48E-16	1.84E-14
PARG2744	-2.542638023	3.222412938	5.80E-16	1.95E-14
PARG2067	-5.871184847	0.327855097	6.97E-16	2.33E-14
PARG2373	-5.864201233	1.798890447	6.97E-16	2.33E-14
PARG1419	-7.56800783	0.165114621	7.85E-16	2.62E-14
PARG0024	-2.773361432	3.866405847	8.16E-16	2.71E-14
PARG1668	2.436284968	1.823506638	8.77E-16	2.91E-14
PARG2092	2.397676294	2.508895874	1.02E-15	3.37E-14
PARG0774	-2.227480034	5.710948452	1.10E-15	3.63E-14
PARG2561	2.864235495	2.822036882	1.12E-15	3.69E-14
PARG0987	-7.535147283	-0.123659399	1.15E-15	3.78E-14
PARG1764	-5.335015644	4.268922532	1.15E-15	3.79E-14
PARG1612	2.530917945	3.840800101	1.18E-15	3.88E-14
PARG0271	2.476527441	5.416226458	1.32E-15	4.33E-14
PARG1832	2.209353828	7.095085274	1.42E-15	4.63E-14
PARG0662	2.275843011	7.333019464	1.46E-15	4.76E-14
PARG1708	-2.593922575	1.641681323	1.68E-15	5.44E-14
PARG2724	-2.212603999	6.581682035	1.84E-15	5.98E-14
PARG2831	3.179595924	1.412377032	1.85E-15	5.98E-14
PARG2066	2.522114682	4.28584534	1.86E-15	6.00E-14
PARG1415	3.531369357	4.324102709	1.95E-15	6.28E-14
PARG1612	-2.321469484	4.402549817	2.02E-15	6.49E-14
PARG1684	2.57561084	3.128019394	2.46E-15	7.89E-14
PARG1985	2.717494047	4.837508826	2.53E-15	8.11E-14
PARG0719	2.587415776	1.83632637	2.67E-15	8.54E-14
PARG1736	-2.279885005	4.797598756	2.73E-15	8.70E-14
PARG1312	2.247512194	3.752673808	3.02E-15	9.62E-14
PARG0297	2.401739066	7.896442031	3.11E-15	9.90E-14
PARG1583	-2.763036462	2.271207578	3.13E-15	9.93E-14
PARG0483	-3.203790715	0.353911886	3.23E-15	1.02E-13
PARG2649	3.802738876	1.189569754	3.27E-15	1.03E-13
PARG0815	-2.340107724	4.405135038	3.53E-15	1.11E-13
PARG1231	2.28612951	5.602941072	3.55E-15	1.12E-13
PARG0901	-2.172696137	7.099883102	3.60E-15	1.13E-13
PARG1971	-2.354431377	4.806984887	3.74E-15	1.17E-13
PARG2241	-2.235103877	5.642097056	3.75E-15	1.17E-13
PARG2406	3.296298257	2.902996627	3.84E-15	1.20E-13

PARG1963	2.355088624	7.415529367	4.00E-15	1.25E-13
PARG2107	-2.273499299	6.129831039	4.02E-15	1.25E-13
PARG2462	-5.66949207	0.634666516	4.17E-15	1.29E-13
PARG2818	-4.315429765	0.158710357	4.26E-15	1.32E-13
PARG1097	2.926466127	0.876387755	4.29E-15	1.33E-13
PARG2062	2.750710366	5.116801211	4.75E-15	1.46E-13
PARG1452	-2.204426433	8.269519114	4.94E-15	1.52E-13
PARG0632	-2.433377043	4.806442228	5.11E-15	1.57E-13
PARG2837	-7.455808459	0.176774995	5.43E-15	1.66E-13
PARG0453	-7.452061392	1.710880415	5.43E-15	1.66E-13
PARG0459	7.449860596	1.421915038	5.43E-15	1.66E-13
PARG1294	-2.681959722	5.145672121	5.66E-15	1.73E-13
PARG0036	2.303499385	3.371507874	6.56E-15	2.00E-13
PARG2698	2.260935439	3.924844369	6.92E-15	2.10E-13
PARG1523	2.557358596	6.294050424	7.57E-15	2.29E-13
PARG0858	2.146986535	6.912156597	7.71E-15	2.33E-13
PARG2118	2.508199951	2.86725341	7.86E-15	2.37E-13
PARG1745	3.020037377	2.976463862	7.89E-15	2.38E-13
PARG1747	-2.537973973	2.551635129	7.96E-15	2.39E-13
PARG2700	3.623867673	5.272522663	8.74E-15	2.62E-13
PARG1240	-3.41914329	4.447372508	8.98E-15	2.69E-13
PARG1602	-3.807569438	0.345197933	9.31E-15	2.78E-13
PARG2134	2.301443482	3.732060883	9.55E-15	2.85E-13
PARG2624	-2.438229959	3.523529232	9.59E-15	2.86E-13
PARG1260	-2.13694729	7.498016262	1.00E-14	2.97E-13
PARG0996	-2.131575945	9.162283335	1.02E-14	3.03E-13
PARG1691	2.347413416	2.875078642	1.03E-14	3.06E-13
PARG1058	2.682277005	1.512178056	1.06E-14	3.13E-13
PARG1131	-7.422731259	-0.363655475	1.20E-14	3.53E-13
PARG1436	7.406425541	0.367116478	1.20E-14	3.53E-13
PARG2311	-2.289071794	4.859650602	1.20E-14	3.54E-13
PARG0431	-2.231250731	3.30301582	1.27E-14	3.74E-13
PARG2720	-2.480925836	2.468882976	1.30E-14	3.81E-13
PARG2328	-3.039315498	1.733999912	1.35E-14	3.94E-13
PARG0918	2.412469174	4.407941332	1.51E-14	4.42E-13
PARG1932	-2.136120918	5.0918006	1.54E-14	4.48E-13
PARG2519	-2.156501259	6.446915883	1.57E-14	4.58E-13
PARG2810	2.920673687	3.377534205	1.67E-14	4.83E-13
PARG0056	2.203401737	5.413547576	1.78E-14	5.14E-13
PARG2704	2.906144513	2.179980077	1.78E-14	5.14E-13
PARG2028	7.382852348	0.276263892	1.79E-14	5.15E-13
PARG2083	-7.37791415	-0.020205334	1.79E-14	5.15E-13
PARG1607	2.110930452	7.89754604	1.80E-14	5.17E-13
PARG0502	-4.643185954	-0.407340722	2.01E-14	5.78E-13
PARG2074	2.821527516	2.05138814	2.15E-14	6.15E-13
PARG2827	-2.147802176	6.20368314	2.49E-14	7.11E-13

PARG1134	2.319142202	4.470138519	2.64E-14	7.54E-13
PARG1677	-7.349967817	-0.601988891	2.68E-14	7.62E-13
PARG0366	2.859052828	3.492479814	2.68E-14	7.62E-13
PARG1096	3.170094915	2.341566041	2.80E-14	7.93E-13
PARG1800	-4.424060921	2.381868723	2.80E-14	7.93E-13
PARG2968	-4.424060921	2.381868723	2.80E-14	7.93E-13
PARG2440	2.887708211	0.649464119	2.82E-14	7.97E-13
PARG1627	-2.14141697	10.40824656	2.88E-14	8.12E-13
PARG2639	-3.092936824	1.128981226	2.96E-14	8.32E-13
PARG2764	-3.976791542	3.671098548	2.97E-14	8.35E-13
PARG1113	2.159730729	4.464986062	3.09E-14	8.67E-13
PARG1064	2.19597563	4.377645047	3.19E-14	8.93E-13
PARG2318	3.093360378	1.313192045	3.42E-14	9.54E-13
PARG1076	2.21812437	6.450185232	3.52E-14	9.83E-13
PARG2995	4.971120405	1.332738583	3.58E-14	9.96E-13
PARG2034	-2.078266033	8.978916733	3.97E-14	1.10E-12
PARG1986	7.342330992	-1.064477921	4.02E-14	1.11E-12
PARG0513	-7.340600599	-0.653496879	4.02E-14	1.11E-12
PARG0350	2.075227656	8.683270522	4.38E-14	1.21E-12
PARG0345	-2.511595256	3.95861426	4.42E-14	1.22E-12
PARG1907	2.080262629	6.766837901	4.50E-14	1.24E-12
PARG0166	2.626332697	2.459046526	4.53E-14	1.25E-12
PARG0233	-2.28662232	4.897128784	5.03E-14	1.38E-12
PARG0098	-2.604632553	2.62031017	5.18E-14	1.42E-12
PARG2348	4.286604089	1.355469634	5.23E-14	1.43E-12
PARG2755	2.08358816	8.628828149	5.79E-14	1.58E-12
PARG2483	-2.718544544	2.14952939	5.92E-14	1.61E-12
PARG0104	2.061047204	9.265096106	6.00E-14	1.63E-12
PARG3003	-4.895688037	-0.09829461	7.29E-14	1.98E-12
PARG2592	3.345173107	1.264286037	7.40E-14	2.01E-12
PARG0876	4.528272796	1.075012406	7.68E-14	2.08E-12
PARG1239	-2.328177796	7.09497188	7.91E-14	2.14E-12
PARG2831	2.172877439	4.265517783	8.36E-14	2.25E-12
PARG2793	-2.345894031	1.695405545	8.43E-14	2.27E-12
PARG0939	2.697654318	1.680604651	8.71E-14	2.34E-12
PARG2462	-2.12838357	4.785159086	9.02E-14	2.42E-12
PARG0053	-3.498866121	1.011874668	1.02E-13	2.74E-12
PARG2239	2.1437311	4.957006345	1.04E-13	2.78E-12
PARG0503	-3.525286704	1.550886814	1.04E-13	2.79E-12
PARG2694	-4.563494648	-0.234670678	1.08E-13	2.87E-12
PARG2631	-2.132416761	5.089242418	1.09E-13	2.89E-12
PARG2771	3.186787702	4.607525072	1.11E-13	2.95E-12
PARG1060	2.378166653	3.063434659	1.23E-13	3.27E-12
PARG1990	-2.176476096	3.826929939	1.23E-13	3.27E-12
PARG0848	2.126478255	4.554952954	1.27E-13	3.37E-12
PARG0043	2.492262113	1.29910794	1.30E-13	3.42E-12

PARG0182	2.222651415	2.579108973	1.37E-13	3.62E-12
PARG2456	2.071467881	5.189730931	1.49E-13	3.92E-12
PARG2070	-2.030311416	8.879939571	1.54E-13	4.05E-12
PARG0249	3.070313956	0.385973164	1.55E-13	4.05E-12
PARG2016	2.336875331	5.482875331	1.67E-13	4.38E-12
PARG1825	5.536006235	0.207928089	1.76E-13	4.61E-12
PARG2265	-2.06752681	8.056628324	1.83E-13	4.78E-12
PARG1552	2.131410811	4.227828533	1.89E-13	4.93E-12
PARG1308	2.034222485	6.339131142	1.91E-13	4.95E-12
PARG2226	-3.219451512	1.527578836	2.06E-13	5.35E-12
PARG3038	-7.255808086	1.721283562	2.09E-13	5.42E-12
PARG0103	2.0180891	7.342753394	2.10E-13	5.44E-12
PARG2087	-2.710650239	1.666182183	2.21E-13	5.71E-12
PARG0843	2.165413053	4.85660415	2.23E-13	5.75E-12
PARG0101	-2.010503845	9.332158332	2.51E-13	6.45E-12
PARG1266	4.202594218	0.107844169	2.64E-13	6.79E-12
PARG2163	-2.041393363	8.024846759	2.76E-13	7.07E-12
PARG2357	2.850255886	0.649223995	3.02E-13	7.75E-12
PARG0614	-2.040515435	6.544253337	3.03E-13	7.76E-12
PARG1377	3.047763392	1.782880953	3.18E-13	8.13E-12
PARG0012	2.585610271	7.800788745	3.26E-13	8.30E-12
PARG2540	-2.359980499	4.022306552	3.35E-13	8.53E-12
PARG2238	-2.501427653	1.289624856	3.46E-13	8.79E-12
PARG2281	-2.168288168	3.520780048	3.49E-13	8.86E-12
PARG0767	2.230527461	5.629482856	3.71E-13	9.38E-12
PARG2725	-1.996966473	6.64319216	3.82E-13	9.65E-12
PARG2928	5.480497035	1.815413937	3.84E-13	9.67E-12
PARG2473	2.05902946	6.914947693	3.84E-13	9.67E-12
PARG1024	2.743133735	2.617788479	4.05E-13	1.02E-11
PARG1324	2.2278772	4.794160457	4.18E-13	1.05E-11
PARG1141	-4.478511559	1.122746165	4.26E-13	1.07E-11
PARG1412	2.176304412	5.469574105	4.47E-13	1.12E-11
PARG0589	2.85739195	2.442012756	4.67E-13	1.17E-11
PARG1697	7.186015054	1.176323358	4.86E-13	1.21E-11
PARG0852	-2.001828581	6.209771419	4.91E-13	1.22E-11
PARG0307	-2.195366785	3.429927357	4.96E-13	1.23E-11
PARG1323	3.455637412	1.687394691	5.18E-13	1.29E-11
PARG1948	2.1114219	4.149175758	5.40E-13	1.34E-11
PARG1550	2.691729447	1.855730723	5.51E-13	1.37E-11
PARG0246	-1.993062417	6.654103016	5.58E-13	1.38E-11
PARG2062	5.471196665	1.799524493	5.68E-13	1.40E-11
PARG2734	-2.024254651	6.515689837	6.05E-13	1.49E-11
PARG1741	2.462212739	2.690198452	6.50E-13	1.60E-11
PARG0871	2.489677521	2.48531101	7.09E-13	1.74E-11
PARG0783	-2.458232836	2.677517999	7.17E-13	1.76E-11
PARG2237	7.166483874	0.521437434	7.44E-13	1.82E-11

PARG0784	-7.163688499	0.508465669	7.44E-13	1.82E-11
PARG2235	-7.143205087	0.392637404	7.44E-13	1.82E-11
PARG2712	-1.976453977	8.688777252	7.79E-13	1.90E-11
PARG0643	-1.981592272	5.871254531	7.88E-13	1.92E-11
PARG0906	-4.458538884	-0.217105175	8.58E-13	2.08E-11
PARG1453	2.448137703	1.522346512	8.99E-13	2.18E-11
PARG0811	2.733896217	0.364337579	9.19E-13	2.23E-11
PARG2297	-2.028436905	7.533114878	9.56E-13	2.31E-11
PARG0387	-2.186485834	3.179642028	9.83E-13	2.37E-11
PARG1939	2.517950419	5.81040785	1.03E-12	2.49E-11
PARG0562	-2.150251011	4.588069614	1.07E-12	2.57E-11
PARG0598	1.98523492	4.671222124	1.14E-12	2.73E-11
PARG2173	-7.141561737	1.047846825	1.14E-12	2.73E-11
PARG1653	-7.137669534	-0.423886084	1.14E-12	2.73E-11
PARG2562	7.121365457	-0.217125893	1.14E-12	2.73E-11
PARG2682	2.376665819	1.468452408	1.16E-12	2.78E-11
PARG0375	2.190048072	1.054102395	1.16E-12	2.78E-11
PARG2018	2.323396795	4.791291055	1.19E-12	2.84E-11
PARG0875	2.91026563	1.90614807	1.22E-12	2.91E-11
PARG0710	-2.210487845	1.685493989	1.25E-12	2.96E-11
PARG1148	1.945637475	7.81422376	1.25E-12	2.96E-11
PARG2733	1.980466286	6.034319959	1.30E-12	3.09E-11
PARG1030	-3.372036145	0.609364475	1.37E-12	3.25E-11
PARG0552	1.953012172	5.73677902	1.40E-12	3.30E-11
PARG1881	1.958133068	7.637751793	1.41E-12	3.32E-11
PARG2121	2.021475292	5.817017394	1.41E-12	3.33E-11
PARG2380	-3.093963564	-0.288202186	1.52E-12	3.57E-11
PARG0668	2.166202083	5.377153059	1.55E-12	3.64E-11
PARG2816	2.075891247	5.410272454	1.59E-12	3.74E-11
PARG1551	-2.304880822	3.243763101	1.68E-12	3.93E-11
PARG2709	1.950138454	4.828850311	1.72E-12	4.02E-11
PARG1529	-4.40489088	-0.251024956	1.74E-12	4.06E-11
PARG1396	7.106663054	-0.007103143	1.76E-12	4.10E-11
PARG0250	-2.074462742	5.357049249	1.91E-12	4.44E-11
PARG2761	-2.13226106	4.380568215	1.98E-12	4.60E-11
PARG1237	2.216668727	1.566649044	2.03E-12	4.71E-11
PARG1387	-1.96614781	5.726707311	2.08E-12	4.81E-11
PARG0862	-1.93475448	7.209089275	2.10E-12	4.86E-11
PARG1258	2.246011726	1.854205904	2.17E-12	5.00E-11
PARG0191	-2.019135573	4.283449931	2.32E-12	5.35E-11
PARG1853	-2.562897331	3.560782472	2.47E-12	5.68E-11
PARG1735	-7.082340819	-0.181186678	2.72E-12	6.25E-11
PARG0885	-1.974909169	5.081938052	2.86E-12	6.55E-11
PARG0209	2.47033972	5.315126319	2.92E-12	6.69E-11
PARG1512	-1.9777294	4.11541542	3.54E-12	8.10E-11
PARG1419	-3.586627075	0.659431697	3.67E-12	8.38E-11

PARG2279	1.92148382	5.150955164	3.76E-12	8.58E-11
PARG0291	2.558337679	2.826252709	3.89E-12	8.86E-11
PARG1943	2.191040552	3.377581544	4.25E-12	9.67E-11
PARG0624	-2.330620498	4.351272779	4.46E-12	1.01E-10
PARG1693	-1.97738718	4.252177784	4.63E-12	1.05E-10
PARG2997	-2.046928133	5.279373839	4.70E-12	1.06E-10
PARG1320	-1.892043274	7.650060021	4.84E-12	1.09E-10
PARG1127	-2.305606564	3.597524847	4.98E-12	1.12E-10
PARG1534	-2.605253284	1.406541618	4.98E-12	1.12E-10
PARG2469	1.905287322	5.4475888	5.06E-12	1.14E-10
PARG1607	-3.100658135	1.018891242	5.12E-12	1.15E-10
PARG2716	2.164916867	2.214163724	5.23E-12	1.17E-10
PARG0684	2.036002117	3.091637062	5.26E-12	1.18E-10
PARG0285	1.973684094	3.486654219	5.34E-12	1.20E-10
PARG0880	-1.991291029	4.573045496	5.38E-12	1.20E-10
PARG0896	1.985319857	4.439683422	5.45E-12	1.22E-10
PARG0229	-1.91649639	5.714865155	5.51E-12	1.23E-10
PARG0763	1.894134419	5.555366392	5.56E-12	1.24E-10
PARG2735	-1.914149927	5.958837796	5.76E-12	1.28E-10
PARG1991	-2.030391709	3.7368189	5.86E-12	1.30E-10
PARG2052	1.888484232	9.524743505	5.86E-12	1.30E-10
PARG1759	-1.885326486	6.768482337	6.26E-12	1.39E-10
PARG2088	-1.876429924	10.57501888	6.31E-12	1.39E-10
PARG0862	-1.933552485	6.144256064	6.34E-12	1.40E-10
PARG2350	-7.023609539	-0.121042232	6.56E-12	1.45E-10
PARG2380	-2.012891752	4.244058977	7.50E-12	1.65E-10
PARG2303	-2.154846307	2.035041331	7.72E-12	1.70E-10
PARG2063	2.198366219	6.631439541	7.87E-12	1.73E-10
PARG0200	2.045865876	8.186296047	8.17E-12	1.79E-10
PARG2086	-1.865955202	8.128813079	8.93E-12	1.95E-10
PARG0410	-2.006101044	5.567646126	8.93E-12	1.95E-10
PARG2078	4.642910061	0.929062306	9.10E-12	1.99E-10
PARG1742	5.299668112	0.399317016	9.53E-12	2.08E-10
PARG0796	1.882466006	4.954806014	9.58E-12	2.09E-10
PARG1397	3.214078173	0.168239733	9.71E-12	2.11E-10
PARG2173	2.472174752	6.386504356	1.04E-11	2.26E-10
PARG2146	1.853599471	9.905957584	1.06E-11	2.29E-10
PARG1580	-2.103349425	2.498582818	1.07E-11	2.32E-10
PARG0867	2.005719438	2.93112736	1.14E-11	2.47E-10
PARG0348	1.856300692	6.697717671	1.16E-11	2.50E-10
PARG2978	2.209462163	5.054221315	1.20E-11	2.58E-10
PARG2834	-2.468538435	0.076127742	1.21E-11	2.61E-10
PARG2223	1.863773153	6.552111573	1.22E-11	2.63E-10
PARG1052	-1.861079949	6.510675407	1.24E-11	2.67E-10
PARG0697	1.871724118	5.069858307	1.26E-11	2.69E-10
PARG0347	-1.853504277	6.853821275	1.35E-11	2.89E-10

PARG2454	1.984937972	5.411694553	1.36E-11	2.90E-10
PARG1527	5.271141239	1.704800403	1.44E-11	3.08E-10
PARG1585	-1.91118727	4.963822947	1.44E-11	3.08E-10
PARG1739	-6.94552826	-0.353269919	1.60E-11	3.41E-10
PARG2075	-2.028819673	2.723989734	1.70E-11	3.61E-10
PARG1900	-1.837741974	7.647234824	1.73E-11	3.68E-10
PARG1216	2.820530081	2.292527725	1.81E-11	3.83E-10
PARG0822	1.890156494	4.489024402	1.93E-11	4.09E-10
PARG2386	2.745229337	4.114756374	1.96E-11	4.14E-10
PARG0037	1.964455071	4.227423028	2.00E-11	4.22E-10
PARG1192	-3.801065584	0.812447686	2.01E-11	4.24E-10
PARG0388	2.181421975	2.383393098	2.11E-11	4.44E-10
PARG0253	-1.948713053	3.486236804	2.13E-11	4.49E-10
PARG2039	-2.982600174	0.088799946	2.29E-11	4.82E-10
PARG2025	6.905088219	-0.364828703	2.52E-11	5.29E-10
PARG0716	-2.482000233	2.73671264	2.57E-11	5.38E-10
PARG1854	2.221679953	2.28677446	2.58E-11	5.39E-10
PARG0840	1.882507888	6.650318574	2.58E-11	5.39E-10
PARG1118	-3.211878674	0.690441397	2.70E-11	5.63E-10
PARG1397	2.244287002	2.720863613	2.74E-11	5.72E-10
PARG0523	2.427264803	1.943438025	2.82E-11	5.87E-10
PARG1315	4.590449848	0.763909856	2.93E-11	6.09E-10
PARG1928	-2.116184847	3.797989534	3.06E-11	6.35E-10
PARG1309	1.902516318	5.521528382	3.35E-11	6.95E-10
PARG0768	-2.330341152	1.807846698	3.36E-11	6.96E-10
PARG0735	-2.999845978	0.074715757	3.75E-11	7.76E-10
PARG1205	2.140958325	4.391300952	3.83E-11	7.91E-10
PARG2823	-2.077676174	2.604924568	3.91E-11	8.07E-10
PARG1824	2.513472455	2.535977788	4.04E-11	8.33E-10
PARG1151	1.89576548	3.569487066	4.23E-11	8.70E-10
PARG0381	-4.587085934	0.265355596	4.34E-11	8.92E-10
PARG0781	-2.187547034	1.39189084	4.54E-11	9.31E-10
PARG0166	-4.216584552	0.961654356	4.62E-11	9.47E-10
PARG2290	1.796331161	7.352352949	4.72E-11	9.65E-10
PARG1528	-2.098623816	2.537971721	4.99E-11	1.02E-09
PARG1187	-2.194277851	4.144520596	5.25E-11	1.07E-09
PARG1231	1.962208128	4.949289513	5.32E-11	1.08E-09
PARG0968	1.795263752	6.363488512	5.38E-11	1.10E-09
PARG1819	3.994315291	2.136054108	5.52E-11	1.12E-09
PARG2945	2.034567652	4.208033329	5.61E-11	1.14E-09
PARG1708	-1.806333945	5.36498321	5.70E-11	1.16E-09
PARG1927	-2.016163149	4.31960871	5.87E-11	1.19E-09
PARG0715	1.793684606	5.189614086	5.87E-11	1.19E-09
PARG2646	1.780394491	10.07692286	5.90E-11	1.19E-09
PARG1418	3.89627132	0.239558377	6.28E-11	1.27E-09
PARG2942	-6.878204361	-1.112211087	6.29E-11	1.27E-09

PARG1448	6.846270775	0.090394426	6.29E-11	1.27E-09
PARG1119	4.532830222	2.050371818	6.46E-11	1.30E-09
PARG1411	2.843148437	3.876201311	6.68E-11	1.34E-09
PARG1122	2.489960641	4.75480434	6.69E-11	1.34E-09
PARG2259	-1.845733574	5.474936531	6.82E-11	1.37E-09
PARG0247	-1.836697256	6.039980386	6.91E-11	1.38E-09
PARG2760	-1.793782834	5.402707929	6.92E-11	1.38E-09
PARG0735	2.141929562	3.29120111	6.97E-11	1.39E-09
PARG0041	-2.543799241	1.068897436	7.25E-11	1.45E-09
PARG1058	1.956857111	3.826829124	7.66E-11	1.52E-09
PARG0362	-3.314721877	0.094844011	7.70E-11	1.53E-09
PARG0247	-5.180088204	0.109774345	7.76E-11	1.54E-09
PARG2181	-2.35780623	0.750349556	7.98E-11	1.58E-09
PARG0058	-1.835584663	5.422641511	8.04E-11	1.59E-09
PARG1386	3.102629665	3.10885432	8.21E-11	1.62E-09
PARG2347	2.022755183	3.251166313	8.51E-11	1.68E-09
PARG1022	-2.591184425	3.224836399	8.72E-11	1.72E-09
PARG0853	-1.992537111	2.677102966	8.86E-11	1.75E-09
PARG2496	3.860762445	3.073968283	8.98E-11	1.77E-09
PARG1890	1.888167318	3.867057382	8.99E-11	1.77E-09
PARG2992	1.800639428	6.363926469	9.05E-11	1.78E-09
PARG1217	-1.80976081	6.972564419	9.07E-11	1.78E-09
PARG2375	2.582051484	1.18869179	9.31E-11	1.82E-09
PARG2914	6.834854445	0.419855199	9.98E-11	1.95E-09
PARG1655	-1.795944359	4.985138101	1.02E-10	1.99E-09
PARG1504	2.668167233	3.067985221	1.07E-10	2.09E-09
PARG2497	1.792649181	6.527568838	1.08E-10	2.10E-09
PARG0491	1.884815101	4.640779582	1.11E-10	2.17E-09
PARG0797	-1.820593203	4.844946659	1.13E-10	2.19E-09
PARG2498	2.124404392	2.282410561	1.15E-10	2.24E-09
PARG0588	2.025109579	1.766114897	1.20E-10	2.32E-09
PARG0534	3.84584005	-0.199983338	1.29E-10	2.49E-09
PARG1039	2.544703474	2.03989261	1.32E-10	2.55E-09
PARG0888	-1.75995546	6.238093397	1.36E-10	2.63E-09
PARG2235	-3.291265562	1.367299484	1.41E-10	2.72E-09
PARG0578	1.756176521	6.402452981	1.43E-10	2.74E-09
PARG0829	1.97542023	4.594092101	1.43E-10	2.74E-09
PARG2847	4.817754799	0.023033957	1.44E-10	2.76E-09
PARG2940	-3.119861256	2.802023999	1.44E-10	2.77E-09
PARG0217	1.955244265	6.27359899	1.46E-10	2.81E-09
PARG1712	2.118856766	2.850922321	1.50E-10	2.87E-09
PARG1112	2.067221703	2.80647947	1.50E-10	2.87E-09
PARG2150	-1.950655213	4.772416065	1.51E-10	2.89E-09
PARG2561	1.814221436	4.530608726	1.57E-10	2.99E-09
PARG1573	2.373172014	2.719437512	1.57E-10	2.99E-09
PARG2690	1.839089104	5.274268814	1.63E-10	3.10E-09

PARG0460	3.711558825	1.679781095	1.72E-10	3.28E-09
PARG0413	-1.812827806	4.923310333	1.73E-10	3.29E-09
PARG1574	1.738621807	8.508613386	1.78E-10	3.38E-09
PARG1279	-1.872787373	3.629908371	1.82E-10	3.44E-09
PARG2942	1.806529115	4.903066322	1.82E-10	3.44E-09
PARG0019	-2.000687237	3.795790538	1.94E-10	3.66E-09
PARG2002	-1.807976689	4.195431315	2.05E-10	3.87E-09
PARG0653	-2.726523365	4.983662815	2.20E-10	4.14E-09
PARG0874	-1.766413158	5.853467179	2.21E-10	4.15E-09
PARG2010	2.270492746	1.077603114	2.34E-10	4.41E-09
PARG3035	-2.171903519	2.633639401	2.49E-10	4.67E-09
PARG1362	1.798592256	3.978130956	2.52E-10	4.72E-09
PARG0841	2.323612603	1.049550893	2.53E-10	4.75E-09
PARG0959	-6.7429208	-0.236798466	2.55E-10	4.76E-09
PARG0283	1.878856772	3.935522624	2.58E-10	4.83E-09
PARG0463	-1.84584013	3.391908429	2.67E-10	4.98E-09
PARG1166	-2.831883494	3.86229958	2.72E-10	5.07E-09
PARG0997	-1.833672083	3.983269254	2.83E-10	5.26E-09
PARG1921	2.064983478	5.373863946	2.87E-10	5.34E-09
PARG0782	2.002690653	2.861215552	3.02E-10	5.61E-09
PARG2231	2.604080503	2.725091835	3.11E-10	5.77E-09
PARG2641	-1.813416764	4.512835605	3.17E-10	5.87E-09
PARG0000	-4.662483513	1.227062684	3.24E-10	5.99E-09
PARG0997	-1.738453536	4.777664314	3.29E-10	6.08E-09
PARG1266	-1.922355227	4.204331668	3.30E-10	6.10E-09
PARG0341	-1.768438807	6.133090998	3.32E-10	6.12E-09
PARG2040	-1.999152563	5.047108376	3.34E-10	6.16E-09
PARG1907	-2.160906456	4.641988651	3.37E-10	6.21E-09
PARG2968	-2.682180816	0.252428962	3.49E-10	6.42E-09
PARG2225	1.71585107	7.077514118	3.72E-10	6.83E-09
PARG0419	-1.940063356	8.127885135	3.73E-10	6.85E-09
PARG0366	-3.827165432	1.64284285	3.82E-10	7.00E-09
PARG1709	1.79412534	3.675204966	3.86E-10	7.05E-09
PARG2273	-2.915106521	2.412694925	4.02E-10	7.35E-09
PARG0589	2.789475751	0.94178682	4.02E-10	7.35E-09
PARG1305	-1.781011035	4.741699531	4.03E-10	7.35E-09
PARG0934	6.721806887	-0.400055285	4.09E-10	7.45E-09
PARG0648	-1.975464278	3.00578317	4.12E-10	7.49E-09
PARG2339	1.719396153	4.840114892	4.18E-10	7.59E-09
PARG2439	1.803280128	5.630702885	4.21E-10	7.65E-09
PARG0858	2.395719466	0.629258212	4.22E-10	7.65E-09
PARG2294	-1.694037487	10.63971307	4.26E-10	7.71E-09
PARG0652	-1.699481628	8.537180342	4.32E-10	7.81E-09
PARG1449	2.187018104	0.951190589	4.33E-10	7.82E-09
PARG2656	1.705748971	6.434111935	4.41E-10	7.95E-09
PARG1267	-1.704348237	7.95339798	4.46E-10	8.04E-09

PARG0585	-4.07592271	1.009942589	4.52E-10	8.14E-09
PARG1282	-2.630351105	0.409929021	4.57E-10	8.21E-09
PARG2456	1.712871617	5.595983072	4.74E-10	8.51E-09
PARG1381	1.750236026	4.561444534	4.82E-10	8.65E-09
PARG2412	1.785177951	3.696060989	4.87E-10	8.73E-09
PARG1152	-1.756026004	3.832467824	5.02E-10	8.99E-09
PARG2219	2.461810099	3.559637494	5.04E-10	9.02E-09
PARG2523	1.698118601	7.66890272	5.20E-10	9.28E-09
PARG0337	-1.785991668	3.959787467	5.28E-10	9.42E-09
PARG2691	-1.695672892	5.89127024	5.43E-10	9.67E-09
PARG2099	-1.683412848	10.702974	5.56E-10	9.90E-09
PARG2456	1.89751769	2.678339263	5.87E-10	1.04E-08
PARG0903	-1.828817543	5.115569685	5.91E-10	1.05E-08
PARG0213	3.004528812	4.329989878	6.00E-10	1.06E-08
PARG2468	2.194585729	2.410364129	6.01E-10	1.06E-08
PARG2455	1.699910633	4.228174027	6.18E-10	1.09E-08
PARG2589	1.767123524	5.15047016	6.22E-10	1.10E-08
PARG2459	-6.698444398	-0.910279061	6.59E-10	1.16E-08
PARG1139	6.696520099	-0.498110254	6.59E-10	1.16E-08
PARG1519	2.236994025	5.562150308	6.63E-10	1.17E-08
PARG2138	3.99420478	-0.129097991	6.66E-10	1.17E-08
PARG2086	3.986318267	2.956353055	6.66E-10	1.17E-08
PARG0847	1.774882546	3.356677835	6.68E-10	1.17E-08
PARG1522	5.215379967	-0.072972125	6.79E-10	1.19E-08
PARG1901	4.985811044	1.672159602	6.79E-10	1.19E-08
PARG0790	4.982229797	0.476741143	6.79E-10	1.19E-08
PARG1941	1.797512517	2.808777481	6.92E-10	1.21E-08
PARG0065	1.716271925	5.20671158	7.06E-10	1.23E-08
PARG1351	2.52261534	5.477660361	7.09E-10	1.24E-08
PARG0271	1.693557087	6.897890452	7.14E-10	1.24E-08
PARG0686	-1.675986438	6.938807938	7.53E-10	1.31E-08
PARG2141	-1.843357041	3.983643828	7.56E-10	1.31E-08
PARG1667	2.188367198	3.149780491	7.57E-10	1.31E-08
PARG2037	-2.364573273	1.635458242	7.62E-10	1.32E-08
PARG0914	-2.683747203	2.827081238	7.82E-10	1.36E-08
PARG0343	3.734164217	3.315655296	7.98E-10	1.38E-08
PARG1232	3.722932474	0.005486159	7.98E-10	1.38E-08
PARG1832	2.979946159	0.478423383	8.01E-10	1.38E-08
PARG2415	2.973536064	1.7752943	8.01E-10	1.38E-08
PARG1858	-1.845578121	3.43447869	8.13E-10	1.40E-08
PARG1319	-1.759869542	4.669330347	8.50E-10	1.46E-08
PARG2515	-2.083889797	0.772143672	8.60E-10	1.48E-08
PARG2171	-2.214845711	1.039688313	8.62E-10	1.48E-08
PARG0741	-1.793634296	5.357629041	8.79E-10	1.51E-08
PARG2253	-1.737875344	4.014321535	8.88E-10	1.52E-08
PARG2819	-1.799066124	4.118315125	9.13E-10	1.56E-08

PARG0544	1.722760377	5.305046975	9.31E-10	1.59E-08
PARG0880	1.906707534	3.220674578	9.55E-10	1.63E-08
PARG2869	-1.749181097	3.585735177	9.67E-10	1.65E-08
PARG1902	-1.661331424	7.503667012	1.02E-09	1.73E-08
PARG0261	2.345738724	0.34836866	1.03E-09	1.75E-08
PARG1234	1.787702006	5.806527848	1.04E-09	1.78E-08
PARG0095	-1.834212894	2.100172598	1.06E-09	1.79E-08
PARG1661	-1.657606001	8.015657037	1.06E-09	1.80E-08
PARG1079	6.622854306	-0.166103099	1.07E-09	1.81E-08
PARG1219	-2.217695051	5.915539186	1.13E-09	1.91E-08
PARG2322	1.666820319	9.097944029	1.18E-09	2.00E-08
PARG0768	1.685025497	4.323953661	1.18E-09	2.00E-08
PARG1898	-1.997417037	3.161776565	1.21E-09	2.05E-08
PARG2302	2.324587052	3.076191633	1.23E-09	2.08E-08
PARG1646	1.877847651	3.326067319	1.23E-09	2.08E-08
PARG2132	1.837214433	3.705023318	1.25E-09	2.10E-08
PARG1826	-3.249418291	1.459111319	1.27E-09	2.13E-08
PARG1553	-3.24526894	5.450859691	1.27E-09	2.13E-08
PARG0042	2.627739923	1.746225504	1.27E-09	2.13E-08
PARG0227	1.680898817	5.099861097	1.30E-09	2.18E-08
PARG2351	-2.337194471	5.443572565	1.31E-09	2.19E-08
PARG1243	-1.791818499	2.727363893	1.39E-09	2.33E-08
PARG2049	-3.970023626	0.024202053	1.46E-09	2.43E-08
PARG2775	1.906339578	2.912354412	1.49E-09	2.48E-08
PARG2000	1.886599492	3.059255454	1.50E-09	2.51E-08
PARG0766	-1.783337985	2.69663995	1.61E-09	2.68E-08
PARG1276	2.174955628	0.341512897	1.62E-09	2.70E-08
PARG0148	-1.659447084	6.420572424	1.64E-09	2.72E-08
PARG1493	-4.977359316	-0.766747114	1.65E-09	2.74E-08
PARG1100	-4.030335406	0.338081323	1.69E-09	2.80E-08
PARG0013	-6.624295305	-0.351931672	1.73E-09	2.87E-08
PARG2726	6.589292155	0.13897947	1.73E-09	2.87E-08
PARG0899	-3.235064149	0.586975555	1.75E-09	2.90E-08
PARG0157	-1.632182714	8.332576239	1.77E-09	2.92E-08
PARG2376	-1.634153268	7.355522666	1.77E-09	2.92E-08
PARG0735	1.701337443	8.588210428	1.81E-09	2.98E-08
PARG2417	1.666694201	5.208993633	1.83E-09	3.01E-08
PARG0717	1.654690988	7.544425616	1.86E-09	3.06E-08
PARG2308	1.635822047	7.166166435	1.91E-09	3.13E-08
PARG0692	1.665213951	5.258659331	1.95E-09	3.20E-08
PARG0052	1.785393311	3.5226348	1.96E-09	3.22E-08
PARG2398	-1.684314205	4.606019981	1.97E-09	3.22E-08
PARG1902	-1.696018917	3.814266729	2.05E-09	3.36E-08
PARG0356	1.71478004	3.264483391	2.12E-09	3.46E-08
PARG2792	1.646967851	4.771818688	2.14E-09	3.50E-08
PARG0857	-1.91522174	2.97317956	2.31E-09	3.77E-08

PARG0913	-3.659698712	0.034425684	2.44E-09	3.97E-08
PARG0454	3.652759616	-0.130489347	2.44E-09	3.97E-08
PARG1062	-1.661053491	5.258292719	2.49E-09	4.05E-08
PARG0994	-1.620976827	7.275354885	2.50E-09	4.06E-08
PARG0282	-1.716502547	5.564770552	2.53E-09	4.10E-08
PARG1039	-3.191251127	0.614910157	2.53E-09	4.11E-08
PARG0776	2.66488027	2.928925279	2.63E-09	4.25E-08
PARG0949	2.651084748	0.168210196	2.63E-09	4.25E-08
PARG0775	1.610412264	8.753831743	2.74E-09	4.42E-08
PARG0669	-1.623327075	7.166142741	2.77E-09	4.48E-08
PARG0225	1.828079924	4.563724118	2.81E-09	4.53E-08
PARG0770	6.581033599	-0.210540401	2.83E-09	4.54E-08
PARG0791	-6.564667722	-0.448961805	2.83E-09	4.54E-08
PARG0072	-6.555100805	-0.114895832	2.83E-09	4.54E-08
PARG1056	-1.690707592	4.760258459	2.83E-09	4.54E-08
PARG2657	-1.798573758	3.453761677	2.84E-09	4.55E-08
PARG2001	1.945309932	4.309961999	2.84E-09	4.56E-08
PARG1802	-1.688889824	8.70631205	2.88E-09	4.61E-08
PARG1732	1.750491273	3.346076146	2.88E-09	4.61E-08
PARG2346	2.113938383	6.719844608	2.93E-09	4.68E-08
PARG2338	1.798648595	1.963405066	2.99E-09	4.77E-08
PARG2350	1.65651374	4.287026142	3.17E-09	5.06E-08
PARG1428	-1.799414952	4.51815243	3.40E-09	5.41E-08
PARG1119	2.050527824	0.827435176	3.41E-09	5.43E-08
PARG2706	1.616367314	5.604573577	3.63E-09	5.76E-08
PARG1656	-1.872149244	1.396470989	3.64E-09	5.77E-08
PARG0620	-1.761763907	4.332511564	3.64E-09	5.78E-08
PARG2777	-3.484734297	1.783829373	3.65E-09	5.79E-08
PARG0946	1.684468648	2.694560162	3.67E-09	5.80E-08
PARG0371	-1.604959683	6.166752152	3.67E-09	5.80E-08
PARG2313	1.803448323	3.90389686	3.85E-09	6.09E-08
PARG1251	-2.02364684	4.640462503	4.01E-09	6.32E-08
PARG1147	-1.598552324	7.28360286	4.08E-09	6.43E-08
PARG2426	-1.643117711	4.847945318	4.14E-09	6.51E-08
PARG2495	-1.596652297	7.348611087	4.15E-09	6.51E-08
PARG0182	1.644470564	5.660766128	4.15E-09	6.51E-08
PARG2087	-1.593924486	7.774944272	4.15E-09	6.51E-08
PARG2743	-1.812690905	2.719164219	4.27E-09	6.70E-08
PARG2916	1.592626035	7.109120699	4.31E-09	6.75E-08
PARG0159	1.634605061	4.228334286	4.47E-09	6.99E-08
PARG1879	1.606642805	6.117003348	4.55E-09	7.12E-08
PARG2170	-2.907262866	-0.686814319	4.63E-09	7.23E-08
PARG2688	6.49900394	-0.186763531	4.64E-09	7.23E-08
PARG1271	1.587868015	8.100914022	4.64E-09	7.23E-08
PARG0010	2.210192325	1.555565279	4.68E-09	7.29E-08
PARG2019	-1.618602543	5.763292863	4.98E-09	7.75E-08

PARG0477	2.026578481	0.073308805	5.03E-09	7.81E-08
PARG1894	-1.585243756	9.760034396	5.04E-09	7.83E-08
PARG0675	-1.687425879	2.909392396	5.06E-09	7.84E-08
PARG1719	-1.637465389	4.790231426	5.09E-09	7.89E-08
PARG1423	-2.001886924	2.546765919	5.20E-09	8.04E-08
PARG1113	1.686279135	3.850257389	5.28E-09	8.16E-08
PARG0030	1.769019605	1.670652442	5.33E-09	8.23E-08
PARG1947	1.75746226	3.538477955	5.36E-09	8.27E-08
PARG0459	1.758694096	7.368307661	5.36E-09	8.27E-08
PARG1245	-2.151853866	0.985908126	5.39E-09	8.30E-08
PARG2648	1.592451231	5.419400446	5.40E-09	8.30E-08
PARG2167	2.506217594	-0.260343651	5.41E-09	8.32E-08
PARG1756	1.694149915	3.783594879	5.46E-09	8.38E-08
PARG1232	2.540289884	1.099891314	5.49E-09	8.43E-08
PARG1414	1.619759416	4.633102251	5.55E-09	8.50E-08
PARG0865	1.705881263	4.138553274	5.76E-09	8.81E-08
PARG0578	1.679245995	3.35407958	5.81E-09	8.89E-08
PARG1831	2.093775973	1.336750043	6.02E-09	9.21E-08
PARG0726	-1.625767419	4.101873993	6.05E-09	9.24E-08
PARG0187	1.721061553	3.836480681	6.09E-09	9.29E-08
PARG1916	1.750045287	5.487798268	6.37E-09	9.71E-08
PARG1901	2.45789428	1.255682583	6.40E-09	9.74E-08
PARG0367	-1.603042988	5.314189004	6.71E-09	1.02E-07
PARG1041	1.576919417	6.627807344	6.75E-09	1.03E-07
PARG1219	-2.099142717	1.440400214	6.83E-09	1.04E-07
PARG1349	1.752228016	4.40480932	7.09E-09	1.08E-07
PARG0442	2.877201513	3.473599978	7.19E-09	1.09E-07
PARG1971	-1.901122536	2.078538104	7.20E-09	1.09E-07
PARG0121	-1.651217691	4.339651695	7.22E-09	1.09E-07
PARG1390	-2.141757942	2.590900814	7.54E-09	1.14E-07
PARG2647	1.603130454	5.605513958	7.84E-09	1.18E-07
PARG1535	-1.892575218	1.414865268	7.98E-09	1.20E-07
PARG0940	1.708234198	3.606074421	8.25E-09	1.24E-07
PARG0137	-2.844065096	0.664748677	8.37E-09	1.26E-07
PARG0962	-1.647365951	5.051338473	8.61E-09	1.29E-07
PARG2705	-1.969762175	2.350916448	8.80E-09	1.32E-07
PARG2392	2.466468824	2.635304304	9.41E-09	1.41E-07
PARG0678	-1.852403148	2.449989968	9.66E-09	1.45E-07
PARG0848	-1.867684819	2.679624497	9.71E-09	1.45E-07
PARG1243	1.925258601	2.021585119	9.94E-09	1.49E-07
PARG0598	1.588055521	4.335110303	1.03E-08	1.54E-07
PARG0835	1.672295064	2.609197989	1.03E-08	1.54E-07
PARG0005	-1.555208845	7.096240452	1.04E-08	1.55E-07
PARG1619	-1.567427569	8.360597192	1.04E-08	1.55E-07
PARG2135	1.56470899	7.350500216	1.07E-08	1.60E-07
PARG0568	-2.729864816	-0.129705869	1.08E-08	1.60E-07

PARG1876	-1.64882071	4.875905563	1.16E-08	1.73E-07
PARG2148	-1.656127791	3.728258856	1.17E-08	1.74E-07
PARG0447	1.546712628	6.364926654	1.17E-08	1.74E-07
PARG1614	1.656737823	2.557633171	1.21E-08	1.79E-07
PARG2498	-1.54008352	9.864101294	1.22E-08	1.80E-07
PARG1644	-1.59023971	4.704261184	1.24E-08	1.84E-07
PARG2257	-6.455558051	-0.570253479	1.26E-08	1.86E-07
PARG3016	-1.558568218	5.83548426	1.29E-08	1.91E-07
PARG1819	2.477258199	3.209859983	1.29E-08	1.91E-07
PARG1841	1.877264477	2.451145049	1.32E-08	1.95E-07
PARG0727	-1.538611553	7.213984715	1.33E-08	1.96E-07
PARG1901	1.544590151	6.610505277	1.39E-08	2.04E-07
PARG2417	1.933084121	2.527887221	1.40E-08	2.06E-07
PARG2952	-1.541155727	8.705177766	1.40E-08	2.06E-07
PARG1445	2.357289353	3.422429578	1.41E-08	2.07E-07
PARG1086	4.158215794	3.614944452	1.41E-08	2.07E-07
PARG1386	-1.573896427	4.84588886	1.47E-08	2.16E-07
PARG0739	1.621579815	3.836027445	1.50E-08	2.20E-07
PARG0353	2.784574041	-0.119383186	1.52E-08	2.22E-07
PARG2915	2.780962218	3.780226703	1.52E-08	2.22E-07
PARG1269	-1.711405305	3.432410885	1.55E-08	2.27E-07
PARG2573	1.551905506	5.688008721	1.56E-08	2.27E-07
PARG2225	2.513025605	1.930667406	1.56E-08	2.27E-07
PARG2024	-1.826548208	4.383740893	1.60E-08	2.33E-07
PARG2131	3.772957945	0.919585846	1.61E-08	2.34E-07
PARG1238	2.285163486	6.029414944	1.61E-08	2.34E-07
PARG1795	1.689862316	1.734581781	1.62E-08	2.35E-07
PARG0355	-1.720059453	3.144668906	1.66E-08	2.41E-07
PARG1196	-1.677299604	4.959923742	1.67E-08	2.42E-07
PARG1682	2.728885671	1.052136493	1.71E-08	2.47E-07
PARG1989	-1.569712093	5.155013879	1.71E-08	2.48E-07
PARG0770	1.521330965	10.70378157	1.74E-08	2.51E-07
PARG1917	-1.544778333	5.2088349	1.75E-08	2.52E-07
PARG1294	-1.733230253	3.387758816	1.76E-08	2.54E-07
PARG2940	1.543731241	5.117159889	1.77E-08	2.54E-07
PARG2439	1.541923492	5.386394024	1.81E-08	2.60E-07
PARG0759	1.521517126	7.829101663	1.85E-08	2.66E-07
PARG0629	-1.535739341	7.051417733	1.88E-08	2.70E-07
PARG2268	-1.712691965	2.751246227	1.88E-08	2.70E-07
PARG0400	1.531472749	6.746219566	1.90E-08	2.73E-07
PARG2748	-1.529232726	6.444565646	1.94E-08	2.78E-07
PARG1818	1.524053536	6.314702069	1.95E-08	2.78E-07
PARG1967	1.628872748	5.388723589	1.97E-08	2.82E-07
PARG2943	-1.580189987	4.873658492	1.98E-08	2.82E-07
PARG2641	-1.522437959	7.158748503	1.98E-08	2.83E-07
PARG2287	1.845127608	3.789827042	2.04E-08	2.91E-07

PARG2598	2.827278343	0.879735789	2.05E-08	2.92E-07
PARG2046	1.526294497	6.07076849	2.06E-08	2.94E-07
PARG0203	1.681975926	3.123114153	2.08E-08	2.95E-07
PARG1512	-6.392870751	-0.050633383	2.09E-08	2.97E-07
PARG2637	-1.803459971	3.723070408	2.12E-08	3.01E-07
PARG1359	-1.571641712	6.094371709	2.14E-08	3.03E-07
PARG2075	-4.142588556	0.099689513	2.18E-08	3.09E-07
PARG1944	-2.570648773	0.275958774	2.19E-08	3.10E-07
PARG0341	1.983404668	2.19312129	2.25E-08	3.18E-07
PARG2293	-1.875740746	1.193867593	2.25E-08	3.18E-07
PARG1983	1.806548745	1.360526968	2.28E-08	3.22E-07
PARG2922	-2.727312024	3.249996202	2.28E-08	3.22E-07
PARG1543	1.678191434	2.979276392	2.35E-08	3.32E-07
PARG2347	-1.777638483	5.303946111	2.36E-08	3.32E-07
PARG0406	-1.506943141	9.108664326	2.38E-08	3.35E-07
PARG2666	-1.814223888	2.287151371	2.42E-08	3.40E-07
PARG2545	-2.879094387	0.76728183	2.46E-08	3.46E-07
PARG1418	1.512391955	6.461311921	2.52E-08	3.53E-07
PARG0598	1.511801111	6.454095529	2.52E-08	3.53E-07
PARG1442	-1.516266718	6.72189438	2.55E-08	3.57E-07
PARG2171	-1.50618622	8.326996716	2.61E-08	3.65E-07
PARG0199	1.527779462	5.615802817	2.62E-08	3.66E-07
PARG2285	-1.624583491	3.796046895	2.67E-08	3.73E-07
PARG2082	-1.833137531	2.539708579	2.70E-08	3.76E-07
PARG1697	1.701249677	4.350150547	2.73E-08	3.81E-07
PARG2848	-1.752263794	1.787770322	2.77E-08	3.86E-07
PARG1729	-1.781546636	2.652494589	2.87E-08	3.99E-07
PARG0613	-2.215091015	-0.011398462	2.90E-08	4.04E-07
PARG0859	-1.498711068	9.291036395	2.92E-08	4.06E-07
PARG0611	1.565635471	3.578155021	2.93E-08	4.06E-07
PARG2688	-1.511650241	5.945371401	2.94E-08	4.07E-07
PARG2406	-1.494822754	8.998386352	3.06E-08	4.24E-07
PARG0659	1.872669607	3.216802231	3.08E-08	4.26E-07
PARG0727	2.008468787	5.900035483	3.14E-08	4.35E-07
PARG1791	-1.826153137	2.475962684	3.23E-08	4.46E-07
PARG2029	-1.531687709	4.269768763	3.23E-08	4.47E-07
PARG1586	1.685549218	7.08030409	3.30E-08	4.55E-07
PARG1551	2.617325238	1.252908741	3.31E-08	4.56E-07
PARG0580	1.810285952	2.942886988	3.48E-08	4.80E-07
PARG2475	-3.046744224	1.026868485	3.51E-08	4.82E-07
PARG0404	-2.232529375	2.563948569	3.56E-08	4.89E-07
PARG1039	2.230856485	1.570525185	3.59E-08	4.93E-07
PARG1960	-2.155118263	4.069731315	3.61E-08	4.96E-07
PARG1305	-3.728384354	2.015205065	3.65E-08	5.00E-07
PARG0715	1.700605539	3.361164096	3.66E-08	5.01E-07
PARG1861	1.487966568	6.241156788	3.67E-08	5.03E-07

PARG2203	-1.553851611	5.139823557	3.71E-08	5.08E-07
PARG0207	-1.591820314	4.018149672	3.72E-08	5.08E-07
PARG1376	1.483752063	9.94507246	3.75E-08	5.11E-07
PARG2484	-1.686468928	1.896951761	3.87E-08	5.28E-07
PARG1039	1.672182177	4.085180501	3.95E-08	5.39E-07
PARG0694	-2.924934915	1.696567375	4.08E-08	5.56E-07
PARG0364	1.57404391	4.501155708	4.11E-08	5.59E-07
PARG0120	-4.697007278	3.553698982	4.12E-08	5.59E-07
PARG1858	1.601722547	2.781602146	4.17E-08	5.67E-07
PARG2550	-1.513797384	4.533209065	4.32E-08	5.86E-07
PARG1221	-1.490986083	6.317377973	4.32E-08	5.86E-07
PARG1056	-1.987614766	1.979448439	4.43E-08	6.00E-07
PARG1803	2.250149703	1.163928369	4.43E-08	6.00E-07
PARG1808	1.677107849	3.21210253	4.45E-08	6.01E-07
PARG0519	1.846624122	2.070895513	4.45E-08	6.01E-07
PARG1162	-1.973822682	1.837720523	4.58E-08	6.18E-07
PARG0251	-1.702466393	2.826191924	4.63E-08	6.25E-07
PARG0135	1.483134461	5.771518924	4.67E-08	6.29E-07
PARG0955	-3.323578882	-0.852563175	4.73E-08	6.37E-07
PARG1263	-1.699106663	3.864182587	4.78E-08	6.43E-07
PARG0852	2.980417623	0.534921212	4.92E-08	6.62E-07
PARG1729	-1.486379216	5.035250279	4.98E-08	6.69E-07
PARG1873	-1.491141582	6.957884813	5.11E-08	6.86E-07
PARG1006	-1.573419111	4.880705781	5.21E-08	6.99E-07
PARG2883	-1.519495996	4.595381064	5.23E-08	6.99E-07
PARG2473	-1.684139389	3.182902306	5.23E-08	6.99E-07
PARG2817	-1.503549627	3.75000117	5.26E-08	7.03E-07
PARG1837	1.950980945	2.52305989	5.27E-08	7.05E-07
PARG1779	-1.736223963	2.98232457	5.28E-08	7.05E-07
PARG2727	-2.146328379	2.633617999	5.33E-08	7.11E-07
PARG2722	2.336692228	1.793847691	5.44E-08	7.24E-07
PARG1170	-1.60475219	4.395487561	5.56E-08	7.41E-07
PARG2016	-6.297059248	-0.320927285	5.83E-08	7.75E-07
PARG1056	-6.295171045	-0.536528914	5.83E-08	7.75E-07
PARG1087	-1.459139287	9.13356242	6.21E-08	8.24E-07
PARG0351	2.015634917	0.737704538	6.31E-08	8.37E-07
PARG0380	-2.802462578	2.264905076	6.32E-08	8.38E-07
PARG1179	-1.933916893	7.040665438	6.44E-08	8.53E-07
PARG1957	1.796866458	2.312713506	6.59E-08	8.72E-07
PARG0534	4.617987394	-0.799611943	6.60E-08	8.73E-07
PARG1798	2.153932005	1.776971358	6.65E-08	8.79E-07
PARG0301	-1.857675701	2.469653966	6.70E-08	8.85E-07
PARG2681	1.621128403	3.935303844	6.73E-08	8.88E-07
PARG0002	1.831965653	4.868051822	6.76E-08	8.90E-07
PARG2715	1.485741682	4.628440327	6.84E-08	9.01E-07
PARG1751	-1.891696867	0.922918262	6.93E-08	9.11E-07

PARG1568	1.797441322	1.436770679	7.08E-08	9.30E-07
PARG1652	1.585143397	3.290217953	7.22E-08	9.49E-07
PARG0849	1.52686246	3.993675017	7.64E-08	1.00E-06
PARG0286	-1.446893137	10.7121184	7.83E-08	1.03E-06
PARG0851	4.029469555	0.526301005	8.13E-08	1.07E-06
PARG1915	1.484626619	5.548313279	8.14E-08	1.07E-06
PARG2269	1.566975769	3.743201161	8.25E-08	1.08E-06
PARG2807	-1.491985522	5.24943402	8.48E-08	1.11E-06
PARG0865	-1.448394044	7.258375193	8.64E-08	1.13E-06
PARG1260	2.321820522	0.580852415	8.66E-08	1.13E-06
PARG2435	1.467314323	5.635816497	8.67E-08	1.13E-06
PARG2734	-1.455167499	6.233878812	8.87E-08	1.16E-06
PARG0275	1.523499102	2.538899807	8.92E-08	1.16E-06
PARG1472	-1.713377746	1.731863301	8.94E-08	1.16E-06
PARG0765	1.526545889	4.492737737	8.98E-08	1.17E-06
PARG2230	-1.496752029	5.391724431	9.01E-08	1.17E-06
PARG2806	1.460182509	5.574033798	9.16E-08	1.19E-06
PARG2408	-1.485600908	6.561051216	9.18E-08	1.19E-06
PARG2006	-1.440165841	8.087149472	9.32E-08	1.21E-06
PARG2034	-1.456711217	6.260654999	9.40E-08	1.22E-06
PARG1311	1.553684841	4.054528987	9.44E-08	1.22E-06
PARG0263	-1.832095982	0.940138305	9.57E-08	1.24E-06
PARG2268	-1.440412785	7.068440012	9.86E-08	1.27E-06
PARG2379	1.916645919	3.272229346	1.00E-07	1.29E-06
PARG1648	1.454790095	6.967355005	1.02E-07	1.32E-06
PARG0704	1.964671279	4.632348215	1.02E-07	1.32E-06
PARG0638	-1.542919595	3.351906149	1.05E-07	1.35E-06
PARG1121	2.233978699	4.378085393	1.06E-07	1.36E-06
PARG0038	-1.480670681	4.279315954	1.08E-07	1.39E-06
PARG0700	1.853727088	1.127929417	1.09E-07	1.39E-06
PARG2150	-1.460549106	5.460458158	1.14E-07	1.46E-06
PARG2172	-1.92170859	1.074293813	1.16E-07	1.49E-06
PARG0641	-1.495276031	4.895304038	1.17E-07	1.50E-06
PARG0887	-1.49996503	4.213801686	1.19E-07	1.52E-06
PARG1057	2.124134094	1.467920841	1.20E-07	1.53E-06
PARG1550	1.672415913	3.317178804	1.21E-07	1.54E-06
PARG2522	1.545137505	5.701956799	1.22E-07	1.56E-06
PARG0007	-1.993227794	4.09891424	1.22E-07	1.56E-06
PARG0601	-3.638500843	-0.382224652	1.27E-07	1.61E-06
PARG0204	1.443960166	5.255398337	1.29E-07	1.65E-06
PARG0274	1.638992425	1.280207443	1.31E-07	1.67E-06
PARG0165	-2.165621742	0.939750814	1.31E-07	1.67E-06
PARG2515	-1.604846376	3.177513083	1.34E-07	1.71E-06
PARG1837	2.881253933	0.124883981	1.37E-07	1.74E-06
PARG1066	-1.419248081	8.972258474	1.43E-07	1.82E-06
PARG2382	-1.447234292	5.493107804	1.45E-07	1.83E-06

PARG1080	1.432838811	4.711315943	1.46E-07	1.85E-06
PARG2454	-1.422306664	6.228670193	1.49E-07	1.88E-06
PARG2745	1.506766164	3.683800952	1.51E-07	1.91E-06
PARG1408	1.582343613	9.466798252	1.53E-07	1.93E-06
PARG0034	-1.41662668	7.452943096	1.55E-07	1.95E-06
PARG0696	2.157402303	1.425662951	1.55E-07	1.95E-06
PARG0440	-1.667742557	3.957880613	1.59E-07	2.00E-06
PARG1516	-1.516104791	4.333502162	1.61E-07	2.03E-06
PARG2291	-2.040446359	0.711946559	1.62E-07	2.03E-06
PARG2464	-1.434498217	6.321261486	1.62E-07	2.04E-06
PARG0645	1.630149129	3.944129086	1.63E-07	2.04E-06
PARG0866	1.528932202	3.451604155	1.64E-07	2.05E-06
PARG1406	1.78647421	3.706237232	1.65E-07	2.07E-06
PARG0365	6.218222106	-0.576947424	1.66E-07	2.07E-06
PARG1783	-6.20673598	1.321504846	1.66E-07	2.07E-06
PARG2277	6.175170987	1.622553487	1.66E-07	2.07E-06
PARG1102	-1.527516726	4.561406648	1.66E-07	2.08E-06
PARG1361	-1.616155476	1.743045126	1.70E-07	2.12E-06
PARG1559	-3.357740022	2.119055431	1.71E-07	2.14E-06
PARG0381	-3.019970616	-0.448255511	1.72E-07	2.14E-06
PARG0299	-2.653313139	-0.143616557	1.72E-07	2.15E-06
PARG1845	2.035005529	5.107540447	1.75E-07	2.18E-06
PARG0104	1.430126439	4.812522158	1.80E-07	2.24E-06
PARG1984	-1.406238972	9.740788531	1.81E-07	2.25E-06
PARG2160	-1.562670394	2.973427561	1.81E-07	2.25E-06
PARG1157	1.435797474	5.247249845	1.84E-07	2.29E-06
PARG1361	-1.929270746	0.639244953	1.86E-07	2.30E-06
PARG1552	1.426784971	4.677257571	1.86E-07	2.31E-06
PARG2278	1.446875944	5.390959264	1.89E-07	2.33E-06
PARG0197	-1.402401244	9.362569287	1.95E-07	2.41E-06
PARG2544	-1.471187782	4.287424128	1.96E-07	2.43E-06
PARG0859	3.9494344	0.114142644	1.98E-07	2.45E-06
PARG2645	1.934337545	0.729156791	2.03E-07	2.50E-06
PARG1109	-1.507068936	3.003827894	2.09E-07	2.58E-06
PARG1192	1.535136863	4.370255009	2.09E-07	2.58E-06
PARG2309	-1.478309981	4.485984527	2.10E-07	2.58E-06
PARG0034	-1.408829448	6.370498312	2.11E-07	2.60E-06
PARG2238	-1.446582492	5.099213655	2.15E-07	2.64E-06
PARG2255	-1.646884866	2.427074142	2.15E-07	2.65E-06
PARG1197	2.239757401	1.806834271	2.16E-07	2.66E-06
PARG0037	1.395182509	8.842831319	2.19E-07	2.69E-06
PARG2669	-1.477992975	4.518841983	2.20E-07	2.70E-06
PARG0569	1.438221323	4.509630557	2.21E-07	2.71E-06
PARG2366	1.406807472	5.557680256	2.28E-07	2.79E-06
PARG1513	-1.480341834	4.713006189	2.37E-07	2.89E-06
PARG0887	-1.486903084	3.62022096	2.39E-07	2.92E-06

PARG1066	2.526570202	1.324187587	2.41E-07	2.94E-06
PARG2959	1.508183106	3.575186215	2.41E-07	2.94E-06
PARG1071	-3.019163996	-0.457499757	2.47E-07	3.00E-06
PARG0285	2.965726439	2.86145986	2.47E-07	3.00E-06
PARG2759	-1.392643273	9.706554371	2.50E-07	3.04E-06
PARG2816	1.991618637	0.946201615	2.50E-07	3.04E-06
PARG2095	1.614806404	4.650615344	2.51E-07	3.05E-06
PARG1054	-3.373365413	-0.718897455	2.55E-07	3.10E-06
PARG1288	3.303371739	0.123604726	2.55E-07	3.10E-06
PARG1693	1.423573736	4.802405518	2.59E-07	3.14E-06
PARG2767	-1.609251145	4.60733211	2.60E-07	3.15E-06
PARG2474	-1.394128793	8.271552864	2.60E-07	3.15E-06
PARG0698	-1.385986837	9.010691114	2.63E-07	3.18E-06
PARG1663	1.600340708	0.997588642	2.65E-07	3.21E-06
PARG0296	-2.029236145	1.865079972	2.68E-07	3.23E-06
PARG1930	1.405973946	6.590698186	2.68E-07	3.24E-06
PARG1770	-1.407932084	6.512577678	2.70E-07	3.26E-06
PARG0327	1.419270931	5.737660924	2.73E-07	3.29E-06
PARG1178	-2.875444249	1.069963668	2.74E-07	3.30E-06
PARG1955	1.403765534	5.141100385	2.77E-07	3.33E-06
PARG1999	1.495475668	2.348215947	2.78E-07	3.34E-06
PARG2270	-6.141150869	-0.5124693	2.81E-07	3.38E-06
PARG0504	-6.133364121	-1.186552219	2.81E-07	3.38E-06
PARG2520	-1.476064562	3.618573399	2.87E-07	3.44E-06
PARG1061	1.427922903	3.692075207	2.87E-07	3.44E-06
PARG1494	1.499776324	2.647654739	2.89E-07	3.47E-06
PARG0631	2.290580415	4.498106315	2.91E-07	3.48E-06
PARG2079	3.506570577	0.883419628	2.94E-07	3.51E-06
PARG0175	1.395380324	5.45645993	3.00E-07	3.58E-06
PARG0040	1.621638087	1.971691247	3.03E-07	3.62E-06
PARG2623	-2.28337705	-0.427119988	3.07E-07	3.67E-06
PARG2010	1.406284471	6.484129748	3.09E-07	3.68E-06
PARG0978	-1.383543407	7.02382386	3.15E-07	3.75E-06
PARG2982	-1.393588798	5.746839774	3.17E-07	3.77E-06
PARG2537	2.158267851	2.072999017	3.17E-07	3.77E-06
PARG1639	-1.412428719	5.41632919	3.19E-07	3.79E-06
PARG2766	-1.388983544	6.007129951	3.23E-07	3.83E-06
PARG1138	-1.919294445	0.568825111	3.25E-07	3.85E-06
PARG2976	-2.483926573	2.64742808	3.26E-07	3.86E-06
PARG2453	-2.008252571	1.532342137	3.28E-07	3.88E-06
PARG3021	1.406766984	5.026913052	3.39E-07	4.01E-06
PARG2032	2.150948849	0.562711013	3.44E-07	4.06E-06
PARG2354	-1.615102534	2.629719502	3.46E-07	4.09E-06
PARG1997	1.645486938	0.806278649	3.49E-07	4.12E-06
PARG2944	-1.378119436	6.812604202	3.56E-07	4.21E-06
PARG0659	-1.399658616	5.769798307	3.59E-07	4.23E-06

PARG1256	1.409653727	4.004758258	3.69E-07	4.34E-06
PARG2454	-1.561440256	1.707685705	3.69E-07	4.35E-06
PARG2446	-1.439787351	4.273290764	3.70E-07	4.36E-06
PARG1426	1.39310411	6.452431416	3.73E-07	4.38E-06
PARG2833	-1.383766454	7.215069152	3.74E-07	4.39E-06
PARG0883	1.412898744	3.116340262	3.79E-07	4.45E-06
PARG0566	-1.372873379	6.49370336	3.82E-07	4.48E-06
PARG0309	1.372679148	6.395571675	3.84E-07	4.51E-06
PARG0597	1.46175212	6.062004319	3.88E-07	4.54E-06
PARG0145	-2.869425774	-0.041241449	3.88E-07	4.54E-06
PARG1535	-1.87022264	2.622011978	3.92E-07	4.59E-06
PARG0513	1.406049828	3.591859361	3.92E-07	4.59E-06
PARG2283	2.150757058	4.920380521	3.95E-07	4.61E-06
PARG0690	-1.374093643	5.429511437	3.96E-07	4.62E-06
PARG0764	-1.411644978	5.137323626	3.97E-07	4.62E-06
PARG0890	-1.369075045	8.142971262	4.01E-07	4.67E-06
PARG1885	-1.387244788	5.648664444	4.01E-07	4.67E-06
PARG1940	-1.764467892	1.777553663	4.10E-07	4.77E-06
PARG1717	1.546840767	2.464335362	4.11E-07	4.78E-06
PARG1292	-1.364723899	7.707965605	4.24E-07	4.93E-06
PARG0800	-2.415855466	0.136848753	4.29E-07	4.98E-06
PARG2123	1.713318832	3.008732309	4.33E-07	5.02E-06
PARG1339	-1.362623497	6.993960092	4.34E-07	5.04E-06
PARG1165	2.448139056	4.143957506	4.35E-07	5.04E-06
PARG1641	2.483579985	-0.239409972	4.38E-07	5.07E-06
PARG2738	-1.509035576	2.935916017	4.45E-07	5.14E-06
PARG2160	-1.463840434	3.6573427	4.51E-07	5.21E-06
PARG1074	-2.195894676	0.128967815	4.53E-07	5.23E-06
PARG0387	-4.505139021	-0.82524521	4.53E-07	5.23E-06
PARG0354	1.964821619	1.69777314	4.53E-07	5.23E-06
PARG2448	-1.406172145	4.969365347	4.57E-07	5.26E-06
PARG0210	-1.408490325	6.860275978	4.59E-07	5.29E-06
PARG1651	-2.093649188	0.894879316	4.68E-07	5.38E-06
PARG1590	-2.213520829	0.240512976	4.73E-07	5.44E-06
PARG0021	-1.359395308	8.175188723	4.74E-07	5.45E-06
PARG0388	1.48671634	2.671048088	4.76E-07	5.47E-06
PARG2974	-1.975092289	2.077102644	4.91E-07	5.64E-06
PARG0343	1.609666968	0.791094447	4.95E-07	5.68E-06
PARG1962	1.413833742	5.734305259	4.96E-07	5.69E-06
PARG2977	1.422555493	6.244297829	4.98E-07	5.71E-06
PARG0981	2.910786386	2.625871203	5.10E-07	5.83E-06
PARG2797	-1.354369966	7.274100799	5.12E-07	5.85E-06
PARG2460	-1.503427841	1.579099982	5.17E-07	5.91E-06
PARG1239	-1.372040212	4.89945417	5.18E-07	5.91E-06
PARG0310	-1.411134267	4.385657973	5.28E-07	6.03E-06
PARG0492	-1.418228549	4.004951822	5.36E-07	6.12E-06

PARG2148	2.969537616	0.478922029	5.50E-07	6.27E-06
PARG2959	1.516438877	3.750329221	5.65E-07	6.43E-06
PARG2321	1.455943625	4.064943396	5.75E-07	6.54E-06
PARG1288	1.372082816	5.024066616	5.82E-07	6.62E-06
PARG1945	1.387104232	4.196866241	5.83E-07	6.62E-06
PARG3035	1.719487262	3.174581885	5.98E-07	6.79E-06
PARG0805	-1.34839994	7.037605017	6.03E-07	6.83E-06
PARG1811	1.525672218	2.376348308	6.06E-07	6.87E-06
PARG1140	1.387146092	5.464868409	6.21E-07	7.04E-06
PARG2755	1.492519299	2.970397367	6.24E-07	7.06E-06
PARG0788	1.694875332	5.193284832	6.43E-07	7.27E-06
PARG2320	-1.403299115	4.934349661	6.44E-07	7.28E-06
PARG1369	1.416703602	5.962498157	6.55E-07	7.40E-06
PARG0831	-1.529148992	4.703946007	6.59E-07	7.44E-06
PARG1498	-1.368890814	5.916655728	6.73E-07	7.59E-06
PARG0391	1.356021655	5.198375726	6.82E-07	7.68E-06
PARG2429	3.437028314	2.368769421	6.88E-07	7.75E-06
PARG0306	2.098990111	1.763252194	6.89E-07	7.75E-06
PARG0212	1.34036115	6.245969829	7.14E-07	8.03E-06
PARG2131	1.873556026	2.202717931	7.16E-07	8.04E-06
PARG1993	-1.41435608	3.230395931	7.17E-07	8.05E-06
PARG2686	1.333711346	7.687556732	7.18E-07	8.05E-06
PARG2376	2.091758949	1.635213012	7.21E-07	8.09E-06
PARG0203	2.299782577	0.409030581	7.25E-07	8.13E-06
PARG2374	2.901150033	2.397598837	7.35E-07	8.23E-06
PARG0382	4.3864125	0.489692033	7.40E-07	8.26E-06
PARG0329	4.385135823	-0.381776263	7.40E-07	8.26E-06
PARG2063	4.38423526	1.64265532	7.40E-07	8.26E-06
PARG2074	1.681206681	2.170860876	7.49E-07	8.37E-06
PARG0217	1.463303964	3.158322048	7.58E-07	8.46E-06
PARG1909	1.879970176	3.86374875	7.61E-07	8.49E-06
PARG1824	1.344133033	5.961652845	7.71E-07	8.58E-06
PARG0126	-3.810925241	2.229493379	7.71E-07	8.58E-06
PARG1211	3.777009042	2.148006807	7.71E-07	8.58E-06
PARG1093	2.219353097	2.344758102	7.71E-07	8.58E-06
PARG1675	-1.969481049	0.898671483	7.86E-07	8.73E-06
PARG1151	-1.391639937	4.296991519	7.87E-07	8.74E-06
PARG0903	1.329103048	9.738854015	7.87E-07	8.74E-06
PARG1158	2.006771266	4.73917078	7.91E-07	8.77E-06
PARG1834	1.804747744	1.308405025	7.93E-07	8.79E-06
PARG2511	1.333087192	7.177334443	8.04E-07	8.90E-06
PARG2925	2.829549653	0.485861473	8.06E-07	8.92E-06
PARG2485	-1.358913316	7.329719991	8.08E-07	8.93E-06
PARG2511	1.370804711	3.959402317	8.22E-07	9.08E-06
PARG1581	-3.222811194	1.491059251	8.53E-07	9.41E-06
PARG2914	3.171391556	0.801628499	8.53E-07	9.41E-06

PARG0842	1.386846658	3.934184675	8.60E-07	9.48E-06
PARG1280	1.350342798	4.809222759	8.62E-07	9.50E-06
PARG0131	1.367998519	4.290587354	8.68E-07	9.56E-06
PARG2131	-1.552800431	4.080257695	8.88E-07	9.77E-06
PARG1852	-1.503809322	5.037128352	8.97E-07	9.86E-06
PARG1811	1.547646265	2.057765753	8.99E-07	9.88E-06
PARG2345	1.372650892	6.94086117	9.29E-07	1.02E-05
PARG1640	1.368720606	5.186829838	9.34E-07	1.02E-05
PARG2236	-1.322197076	7.778476485	9.59E-07	1.05E-05
PARG0036	1.319214486	7.34957655	9.61E-07	1.05E-05
PARG1088	1.507665787	2.060222266	9.63E-07	1.05E-05
PARG2758	1.639422649	4.266193104	9.68E-07	1.06E-05
PARG0229	-3.055791287	-0.048156889	9.77E-07	1.07E-05
PARG1544	-1.403302603	4.612323464	9.81E-07	1.07E-05
PARG2532	1.370540509	4.756482874	9.90E-07	1.08E-05
PARG0750	-1.3386469	5.979898124	1.00E-06	1.09E-05
PARG2400	-1.32245746	7.380498233	1.00E-06	1.09E-05
PARG0175	1.320822601	6.584421698	1.02E-06	1.11E-05
PARG1906	1.350140275	7.271683524	1.02E-06	1.11E-05
PARG1864	-1.39008147	4.020424438	1.04E-06	1.13E-05
PARG0612	-1.331804693	5.887695385	1.05E-06	1.14E-05
PARG2036	3.392297234	0.317389429	1.06E-06	1.15E-05
PARG0377	-1.441949463	3.386550172	1.07E-06	1.16E-05
PARG2311	2.537367345	5.028134087	1.08E-06	1.17E-05
PARG2753	2.433114645	-0.169242443	1.08E-06	1.17E-05
PARG2579	1.68060769	3.626623187	1.10E-06	1.19E-05
PARG2822	-1.349177391	4.673229854	1.10E-06	1.20E-05
PARG1785	-1.495636698	2.533133134	1.11E-06	1.20E-05
PARG1351	1.335667533	8.130579969	1.12E-06	1.21E-05
PARG0149	1.371708952	3.937303329	1.15E-06	1.24E-05
PARG2461	1.757449862	3.097665373	1.16E-06	1.25E-05
PARG1481	-1.810623544	0.825833893	1.17E-06	1.26E-05
PARG0619	1.398320672	4.932416978	1.17E-06	1.26E-05
PARG2435	1.454095318	5.022060584	1.17E-06	1.26E-05
PARG1573	-1.456707804	4.536232312	1.19E-06	1.28E-05
PARG0888	1.422097875	5.545223571	1.20E-06	1.29E-05
PARG1762	4.333115588	1.260551747	1.21E-06	1.30E-05
PARG1416	1.573040292	4.299767046	1.21E-06	1.30E-05
PARG2151	1.466861114	4.36186531	1.21E-06	1.30E-05
PARG2662	3.78312478	0.495434983	1.22E-06	1.31E-05
PARG1248	1.867702005	6.735443472	1.22E-06	1.31E-05
PARG0313	1.472332351	4.277497821	1.23E-06	1.32E-05
PARG2702	1.500406932	3.180748992	1.25E-06	1.34E-05
PARG0087	1.764318745	1.817931859	1.28E-06	1.37E-05
PARG0211	1.404797789	3.598687009	1.30E-06	1.39E-05
PARG1173	1.308532188	8.961552341	1.31E-06	1.40E-05

PARG2604	-1.529076733	1.891652247	1.32E-06	1.41E-05
PARG2576	-2.344866013	0.466181033	1.33E-06	1.41E-05
PARG1852	1.404549288	5.140086901	1.35E-06	1.43E-05
PARG2444	1.82452541	1.288030516	1.35E-06	1.43E-05
PARG0722	1.299664673	9.010669647	1.39E-06	1.48E-05
PARG1589	1.301597144	7.809947578	1.39E-06	1.48E-05
PARG0147	-1.372706426	4.19707454	1.40E-06	1.49E-05
PARG1433	1.359774876	4.294397685	1.41E-06	1.50E-05
PARG2179	5.97488453	-0.213377162	1.42E-06	1.51E-05
PARG0401	1.760809113	1.954204982	1.42E-06	1.51E-05
PARG0674	2.100028379	4.55346048	1.44E-06	1.53E-05
PARG1256	1.292690908	12.15531093	1.44E-06	1.53E-05
PARG2925	-1.352404259	4.454859744	1.46E-06	1.55E-05
PARG0760	2.0269454	1.999272494	1.47E-06	1.55E-05
PARG1772	1.982611142	1.959837098	1.47E-06	1.55E-05
PARG1110	1.344176898	5.182256923	1.47E-06	1.56E-05
PARG0922	-1.756422444	1.361301023	1.51E-06	1.59E-05
PARG1063	-1.45386153	6.158826819	1.56E-06	1.65E-05
PARG0899	-2.651574001	-0.192968149	1.58E-06	1.67E-05
PARG1424	-1.937016512	3.309163511	1.59E-06	1.67E-05
PARG1071	-1.661107298	0.85179064	1.59E-06	1.67E-05
PARG2248	-1.356418061	4.598244606	1.60E-06	1.68E-05
PARG2780	-1.486248092	1.105753459	1.61E-06	1.69E-05
PARG0935	-3.420196574	3.501397896	1.62E-06	1.71E-05
PARG0789	-1.334766847	5.609204248	1.63E-06	1.71E-05
PARG0544	1.35941273	3.316436006	1.64E-06	1.72E-05
PARG1273	1.30599998	4.45715904	1.65E-06	1.73E-05
PARG2461	-2.013992986	2.976709201	1.72E-06	1.80E-05
PARG2607	1.414762899	3.1120568	1.73E-06	1.82E-05
PARG1776	-1.394010256	3.979656802	1.76E-06	1.84E-05
PARG2319	-1.432703357	4.435714937	1.76E-06	1.85E-05
PARG2634	1.595617639	2.746197714	1.77E-06	1.85E-05
PARG2138	-1.300471913	6.253029221	1.77E-06	1.85E-05
PARG1789	-1.354162209	4.289854414	1.78E-06	1.86E-05
PARG0085	1.616016165	4.459604138	1.79E-06	1.87E-05
PARG0115	-1.705485531	1.254842624	1.81E-06	1.88E-05
PARG1381	1.319154865	4.579050638	1.85E-06	1.93E-05
PARG0750	1.944503432	1.916424667	1.87E-06	1.95E-05
PARG2454	-1.478927694	3.08421199	1.89E-06	1.96E-05
PARG2758	1.277862135	10.12594402	1.89E-06	1.97E-05
PARG0744	1.773249867	1.78035556	1.91E-06	1.99E-05
PARG0332	1.284060813	6.426663389	1.91E-06	1.99E-05
PARG2015	-1.279650339	7.966483506	1.92E-06	2.00E-05
PARG1667	3.106180772	0.354823782	1.92E-06	2.00E-05
PARG2861	1.799981939	1.696386644	1.93E-06	2.00E-05
PARG2393	3.688619136	2.419863414	1.93E-06	2.00E-05

PARG1646	3.684658225	0.585939696	1.93E-06	2.00E-05
PARG0617	1.455025273	4.881995302	1.93E-06	2.00E-05
PARG2769	-1.293884711	7.031894905	1.94E-06	2.00E-05
PARG0612	-1.835605544	4.411043279	1.94E-06	2.00E-05
PARG2982	-1.483547645	2.453547577	1.95E-06	2.01E-05
PARG1289	1.287364468	6.918471684	1.98E-06	2.05E-05
PARG1837	1.406306869	3.494558427	1.98E-06	2.05E-05
PARG2086	1.604171834	2.667985335	2.00E-06	2.06E-05
PARG0281	1.355338566	4.020311598	2.01E-06	2.07E-05
PARG1990	-1.429180878	2.554765279	2.03E-06	2.09E-05
PARG2930	-1.317542365	5.009577521	2.03E-06	2.09E-05
PARG0017	1.367635449	4.315496906	2.03E-06	2.09E-05
PARG1780	1.615831146	1.447802716	2.04E-06	2.09E-05
PARG1697	-1.812400523	0.20809962	2.05E-06	2.10E-05
PARG1091	1.493125448	3.094588606	2.05E-06	2.11E-05
PARG2078	1.411009232	4.202242354	2.06E-06	2.11E-05
PARG2018	-1.566621908	5.271388416	2.07E-06	2.12E-05
PARG1275	-2.462517495	0.383429296	2.07E-06	2.12E-05
PARG1845	-1.72004965	2.090888767	2.09E-06	2.14E-05
PARG2859	-1.462205166	3.361845297	2.11E-06	2.16E-05
PARG1074	2.941276603	0.327655671	2.12E-06	2.16E-05
PARG1113	1.947527024	-0.379518493	2.14E-06	2.19E-05
PARG1349	-1.445766524	4.071342345	2.15E-06	2.20E-05
PARG0156	-2.524837448	0.797450977	2.16E-06	2.20E-05
PARG1761	-2.438261355	-0.379814078	2.16E-06	2.20E-05
PARG2607	2.430775364	0.014359237	2.16E-06	2.20E-05
PARG1972	-1.371889822	3.686702527	2.16E-06	2.20E-05
PARG0304	-1.280270357	6.135065936	2.18E-06	2.22E-05
PARG0574	-1.284446114	5.444083195	2.21E-06	2.24E-05
PARG1863	-2.815173003	0.414845205	2.22E-06	2.25E-05
PARG0197	2.770710138	0.182704626	2.22E-06	2.25E-05
PARG1556	2.879469739	3.51024955	2.24E-06	2.28E-05
PARG0781	-1.525474285	4.85725264	2.26E-06	2.29E-05
PARG0585	-1.359332523	3.980212691	2.29E-06	2.32E-05
PARG1691	-1.492770974	2.626310327	2.34E-06	2.37E-05
PARG0741	1.477545602	3.784613887	2.34E-06	2.37E-05
PARG1407	1.404008942	4.691447897	2.36E-06	2.38E-05
PARG2268	-1.471626871	3.325596187	2.37E-06	2.39E-05
PARG2068	1.325066434	4.717577764	2.39E-06	2.42E-05
PARG2166	1.284065277	5.924235416	2.42E-06	2.44E-05
PARG1844	1.268581775	7.316946221	2.42E-06	2.44E-05
PARG2213	-2.171062153	1.169641078	2.43E-06	2.45E-05
PARG1138	1.39303081	3.46619774	2.46E-06	2.48E-05
PARG0919	-1.453237604	5.016134317	2.46E-06	2.48E-05
PARG2404	-1.278313099	5.506919954	2.54E-06	2.56E-05
PARG1983	-1.279712328	5.293697136	2.56E-06	2.57E-05

PARG1657	-1.660510329	1.554286917	2.56E-06	2.57E-05
PARG0679	1.431366899	4.033604756	2.57E-06	2.58E-05
PARG0215	2.247122151	3.976308616	2.64E-06	2.65E-05
PARG2731	1.757942948	5.216829071	2.65E-06	2.65E-05
PARG1277	-1.38619763	3.346669531	2.68E-06	2.68E-05
PARG0245	1.263575743	7.180812674	2.69E-06	2.69E-05
PARG2519	-1.347587442	4.802538929	2.70E-06	2.71E-05
PARG0059	1.39993512	2.946272287	2.73E-06	2.73E-05
PARG2282	-1.263432745	7.378235466	2.77E-06	2.77E-05
PARG2195	1.531315048	2.686778794	2.78E-06	2.78E-05
PARG1291	1.312578296	4.404881851	2.78E-06	2.78E-05
PARG0310	1.702407992	0.735656968	2.81E-06	2.81E-05
PARG0891	2.388105405	4.945133138	2.84E-06	2.83E-05
PARG1274	1.289984659	7.144148348	2.85E-06	2.84E-05
PARG0087	2.032762713	0.990257948	2.88E-06	2.87E-05
PARG1248	-1.317304604	4.866533758	2.91E-06	2.90E-05
PARG0833	1.593483911	1.664813907	2.91E-06	2.90E-05
PARG1948	1.313012113	3.913243885	3.02E-06	3.00E-05
PARG2494	-1.308582644	4.191094529	3.04E-06	3.02E-05
PARG0899	-2.056214659	-0.021200253	3.07E-06	3.05E-05
PARG0730	3.638480312	2.11535991	3.07E-06	3.05E-05
PARG1637	1.289486591	4.950144979	3.08E-06	3.05E-05
PARG1581	2.906888818	3.043896426	3.12E-06	3.09E-05
PARG0902	1.253045516	8.151938358	3.13E-06	3.10E-05
PARG2195	-1.272603597	5.505264097	3.15E-06	3.12E-05
PARG1331	1.263212537	5.217312681	3.16E-06	3.12E-05
PARG2323	-1.332188592	4.951087786	3.16E-06	3.13E-05
PARG0017	2.012571619	6.376466441	3.21E-06	3.17E-05
PARG1359	1.278270968	4.573005227	3.21E-06	3.17E-05
PARG2655	-1.409159053	5.831188478	3.24E-06	3.19E-05
PARG1844	1.729345622	1.22468238	3.30E-06	3.25E-05
PARG1390	1.702243102	1.835061924	3.30E-06	3.25E-05
PARG1087	1.618975881	6.035576856	3.33E-06	3.28E-05
PARG2216	1.570954546	1.923563596	3.38E-06	3.32E-05
PARG2786	-1.394989097	4.657248953	3.39E-06	3.33E-05
PARG1437	-1.341955125	3.39426826	3.40E-06	3.35E-05
PARG0524	2.144096099	4.456333064	3.45E-06	3.39E-05
PARG0756	-1.672905424	-0.305502385	3.59E-06	3.52E-05
PARG0555	-2.350940011	-0.005012514	3.63E-06	3.56E-05
PARG3039	1.263087628	5.042736226	3.64E-06	3.57E-05
PARG1275	1.709050254	3.580855558	3.64E-06	3.57E-05
PARG0588	1.445072781	2.25274883	3.77E-06	3.70E-05
PARG0367	1.452859519	2.367215567	3.80E-06	3.72E-05
PARG1121	2.516295361	4.586940012	3.90E-06	3.81E-05
PARG1201	-1.456426242	4.272847532	3.90E-06	3.81E-05
PARG0039	-1.461755877	1.665366331	3.93E-06	3.84E-05

PARG1243	1.341153411	3.807496343	3.96E-06	3.86E-05
PARG2842	1.438968928	3.258802984	3.99E-06	3.89E-05
PARG0278	-1.278992961	4.617675407	4.05E-06	3.95E-05
PARG2876	1.267830679	5.607130477	4.09E-06	3.99E-05
PARG1250	-1.277328159	6.199463476	4.10E-06	3.99E-05
PARG2924	-1.293957223	6.13684648	4.19E-06	4.08E-05
PARG0231	1.279579466	3.406811144	4.20E-06	4.08E-05
PARG0687	1.55608122	2.119164527	4.26E-06	4.14E-05
PARG0448	1.398960526	2.396441327	4.27E-06	4.14E-05
PARG1423	-5.859320129	0.126082247	4.27E-06	4.15E-05
PARG0326	-1.240245903	7.141177913	4.30E-06	4.17E-05
PARG1955	1.752514408	2.599165448	4.34E-06	4.21E-05
PARG0565	3.033994262	1.386200923	4.37E-06	4.23E-05
PARG2719	-2.563612928	3.207108436	4.38E-06	4.24E-05
PARG2719	2.381110122	2.969121562	4.38E-06	4.24E-05
PARG1663	-1.504487212	3.316452328	4.47E-06	4.32E-05
PARG0774	-1.246276575	4.934802396	4.47E-06	4.32E-05
PARG1835	2.126975063	3.054778299	4.51E-06	4.36E-05
PARG2976	-2.291014758	2.627835439	4.51E-06	4.36E-05
PARG3009	1.312974739	3.450145837	4.56E-06	4.40E-05
PARG2075	-2.652606131	0.382446407	4.56E-06	4.40E-05
PARG0010	1.970284884	2.775589162	4.57E-06	4.40E-05
PARG2489	2.648921583	0.303155162	4.61E-06	4.44E-05
PARG0047	1.264723397	5.773451143	4.62E-06	4.45E-05
PARG0872	-1.434080911	3.629511581	4.62E-06	4.45E-05
PARG1035	-2.65797542	-0.71179949	4.65E-06	4.47E-05
PARG0400	1.277495616	4.101545103	4.66E-06	4.47E-05
PARG0302	1.231878087	6.629514834	4.75E-06	4.56E-05
PARG0823	-1.709872968	1.8666367	4.76E-06	4.57E-05
PARG0514	-1.230125636	7.391549878	4.79E-06	4.60E-05
PARG2027	1.310588925	3.583679708	4.81E-06	4.61E-05
PARG1776	1.312822548	3.173857927	4.86E-06	4.66E-05
PARG1335	1.22678282	8.231980983	4.88E-06	4.67E-05
PARG1433	1.272922633	4.031804599	4.88E-06	4.67E-05
PARG0824	3.598041974	0.023052115	4.89E-06	4.68E-05
PARG0261	1.259138362	5.116501677	4.92E-06	4.70E-05
PARG0780	2.30452282	3.098489565	4.92E-06	4.70E-05
PARG2519	1.333259677	4.323698507	4.92E-06	4.70E-05
PARG0680	-1.251607264	5.531709076	4.95E-06	4.72E-05
PARG1435	1.226847576	10.50954852	4.96E-06	4.73E-05
PARG0675	1.981932087	2.701162583	4.97E-06	4.73E-05
PARG0191	1.257132184	4.590972072	4.99E-06	4.75E-05
PARG1382	1.786145654	0.099051186	5.08E-06	4.83E-05
PARG2296	-1.580784304	2.286577201	5.09E-06	4.84E-05
PARG1591	1.24425544	8.774559407	5.09E-06	4.84E-05
PARG3003	1.937312886	2.952789568	5.13E-06	4.88E-05

PARG0368	1.741582479	1.171105379	5.35E-06	5.08E-05
PARG1771	-1.241973658	5.836481046	5.39E-06	5.11E-05
PARG0859	-1.27773367	6.261324448	5.39E-06	5.11E-05
PARG0084	-4.430364624	-0.30986135	5.45E-06	5.16E-05
PARG0788	-1.264500681	4.685632671	5.50E-06	5.21E-05
PARG2360	1.30898106	4.071809363	5.60E-06	5.30E-05
PARG2111	-1.215889688	9.327280172	5.67E-06	5.36E-05
PARG2628	1.561842048	3.086993739	5.71E-06	5.39E-05
PARG1692	-1.311583009	3.64703864	5.76E-06	5.44E-05
PARG0016	2.429790849	0.826916297	5.77E-06	5.44E-05
PARG1260	-1.226196419	7.370600957	5.86E-06	5.53E-05
PARG1929	1.38803822	2.741376482	5.89E-06	5.55E-05
PARG1373	2.154425108	-0.306775465	5.90E-06	5.56E-05
PARG2123	1.264121715	4.565872658	5.91E-06	5.56E-05
PARG1931	1.755829567	2.752440184	6.04E-06	5.69E-05
PARG2780	1.577226077	3.142572715	6.08E-06	5.72E-05
PARG0183	-1.345508945	4.370939104	6.18E-06	5.81E-05
PARG2316	1.22991669	5.661382545	6.22E-06	5.84E-05
PARG0968	1.46059275	1.876275847	6.26E-06	5.87E-05
PARG2776	1.985079531	0.715287982	6.39E-06	5.99E-05
PARG2438	1.220740746	5.927407866	6.40E-06	6.00E-05
PARG2167	-1.278473738	2.886536029	6.45E-06	6.04E-05
PARG0702	-1.312299822	2.885107137	6.58E-06	6.16E-05
PARG2140	-1.251843401	5.032943829	6.59E-06	6.17E-05
PARG0271	1.262151869	5.804849644	6.63E-06	6.20E-05
PARG0216	-1.337985333	3.666374352	6.73E-06	6.29E-05
PARG2607	2.693736872	-0.364046901	6.76E-06	6.31E-05
PARG2823	2.659941247	5.52702782	6.76E-06	6.31E-05
PARG0778	-1.231158548	5.405927365	6.86E-06	6.40E-05
PARG1383	1.508983726	4.636037125	6.86E-06	6.40E-05
PARG1854	-1.287106851	4.905183079	7.07E-06	6.59E-05
PARG0433	-1.620171073	0.941733155	7.07E-06	6.59E-05
PARG1232	1.971096377	0.843859062	7.08E-06	6.59E-05
PARG2368	1.361406423	3.407087493	7.09E-06	6.60E-05
PARG0185	-1.225703076	6.565777966	7.10E-06	6.60E-05
PARG1198	-1.869190645	0.309580726	7.16E-06	6.65E-05
PARG0182	-1.860840858	2.195009237	7.16E-06	6.65E-05
PARG2691	1.266504512	4.639807128	7.20E-06	6.68E-05
PARG0889	-1.218003997	6.088510574	7.22E-06	6.70E-05
PARG2675	-1.332784171	5.650223982	7.31E-06	6.78E-05
PARG0784	-1.22119391	5.648724815	7.34E-06	6.80E-05
PARG2094	1.210834613	6.559574733	7.40E-06	6.85E-05
PARG0656	-1.239882667	5.000859963	7.41E-06	6.86E-05
PARG1098	-5.740681898	1.074831529	7.48E-06	6.91E-05
PARG0590	-5.733271842	-0.506840689	7.48E-06	6.91E-05
PARG0104	1.205899145	6.62931584	7.50E-06	6.93E-05

PARG0204	1.286567439	3.010881959	7.51E-06	6.94E-05
PARG0456	1.638984286	1.17899398	7.59E-06	7.01E-05
PARG2874	-1.27046276	3.84322781	7.65E-06	7.05E-05
PARG2267	1.821940704	1.60570626	7.66E-06	7.06E-05
PARG2182	-1.268976803	4.282690037	7.81E-06	7.19E-05
PARG1236	3.585129789	-0.303092957	7.82E-06	7.19E-05
PARG2453	-3.570046211	-0.592775341	7.82E-06	7.19E-05
PARG1945	3.5490234	0.576489829	7.82E-06	7.19E-05
PARG0285	-1.326805545	4.233027618	8.02E-06	7.37E-05
PARG1849	-1.240589767	6.785642811	8.06E-06	7.41E-05
PARG2729	1.319194722	6.258921555	8.19E-06	7.52E-05
PARG2470	-1.200448051	7.187895035	8.28E-06	7.60E-05
PARG0425	-1.76370028	2.137945896	8.36E-06	7.66E-05
PARG1909	1.489165019	6.001242263	8.55E-06	7.83E-05
PARG2618	1.283021082	3.083344672	8.56E-06	7.84E-05
PARG1997	-1.389656974	2.690028261	8.60E-06	7.87E-05
PARG1893	1.284324168	3.541412932	8.83E-06	8.07E-05
PARG2500	1.964055001	1.030262136	8.96E-06	8.18E-05
PARG1452	1.919388053	2.244110215	8.96E-06	8.18E-05
PARG1393	1.838222652	1.688844396	8.96E-06	8.18E-05
PARG0648	2.120093506	1.528678261	8.96E-06	8.18E-05
PARG0402	1.198874234	6.64790413	9.05E-06	8.26E-05
PARG2286	1.628799059	2.159765228	9.08E-06	8.27E-05
PARG0051	1.775405209	1.551415972	9.08E-06	8.27E-05
PARG2449	-1.189333905	8.899546456	9.08E-06	8.27E-05
PARG2393	-1.198194139	6.324320475	9.11E-06	8.29E-05
PARG0882	-1.280317052	4.216761908	9.23E-06	8.40E-05
PARG1654	1.299884086	5.408060772	9.35E-06	8.50E-05
PARG1036	1.19121976	7.198783377	9.52E-06	8.65E-05
PARG1356	-1.248023125	4.245647782	9.60E-06	8.72E-05
PARG1172	-1.192319783	6.149618844	9.73E-06	8.83E-05
PARG2639	2.942892821	0.0422501	9.99E-06	9.06E-05
PARG0957	1.451349345	4.525113381	1.00E-05	9.06E-05
PARG1446	1.251444924	4.869535102	1.00E-05	9.06E-05
PARG2673	-1.195011031	4.956207236	1.01E-05	9.11E-05
PARG2604	1.470129755	2.339537152	1.01E-05	9.12E-05
PARG2775	2.760070164	1.976316936	1.01E-05	9.13E-05
PARG1347	-1.371159756	2.334046101	1.01E-05	9.15E-05
PARG2107	1.32072643	2.734695346	1.02E-05	9.25E-05
PARG2847	-1.981329136	0.178478004	1.02E-05	9.25E-05
PARG1189	1.188740616	6.098953468	1.02E-05	9.25E-05
PARG0810	1.289585295	2.126137081	1.03E-05	9.28E-05
PARG2757	1.18551352	7.001201629	1.03E-05	9.29E-05
PARG2372	1.182852859	7.969787713	1.04E-05	9.39E-05
PARG1314	-1.195635969	5.615842509	1.05E-05	9.46E-05
PARG1606	-1.227656521	6.349493735	1.06E-05	9.56E-05

PARG2217	1.559286819	1.764668618	1.07E-05	9.58E-05
PARG0753	-1.252667702	4.54679119	1.07E-05	9.61E-05
PARG1572	-1.194194703	6.460612809	1.08E-05	9.67E-05
PARG2502	1.241536505	4.634970583	1.08E-05	9.70E-05
PARG2271	-1.810138432	1.593780954	1.09E-05	9.78E-05
PARG0371	2.185046588	2.893324981	1.10E-05	9.82E-05
PARG1851	-1.197861199	5.661068646	1.11E-05	9.92E-05
PARG0715	-1.205501011	4.521180217	1.11E-05	9.96E-05
PARG2794	-1.238441951	4.318825642	1.11E-05	9.96E-05
PARG2119	2.357810894	2.729302357	1.11E-05	9.96E-05
PARG2744	1.175654854	8.773176998	1.13E-05	0.00010136
PARG2000	1.177915578	7.155081849	1.16E-05	0.000103555
PARG2074	2.00245011	0.857763917	1.16E-05	0.000103941
PARG2353	1.313727524	2.835507192	1.17E-05	0.000104149
PARG2179	-1.173185343	7.924075106	1.19E-05	0.000106518
PARG0295	1.652830184	1.809985981	1.21E-05	0.000108032
PARG1645	-1.178158642	6.874520781	1.22E-05	0.000108879
PARG0197	2.590014701	0.013753721	1.22E-05	0.000108988
PARG0907	1.388336539	4.137551228	1.24E-05	0.00011065
PARG1230	3.493966122	2.427787654	1.25E-05	0.00011141
PARG0442	3.48996	1.746788444	1.25E-05	0.00011141
PARG2181	1.190327682	4.782586591	1.26E-05	0.000112107
PARG0757	-1.180170565	6.680677667	1.26E-05	0.00011219
PARG0630	1.17937181	6.999020423	1.27E-05	0.000112995
PARG1912	-1.187967365	5.696012304	1.29E-05	0.000114609
PARG2955	1.180030607	7.698583447	1.30E-05	0.000114922
PARG1822	-2.15241056	-0.043654154	1.30E-05	0.000114922
PARG2505	-1.24003828	3.742632706	1.31E-05	0.00011572
PARG1115	-1.173756569	7.937711852	1.31E-05	0.00011572
PARG0821	5.700603281	0.03577299	1.32E-05	0.000116323
PARG2665	5.670697928	0.365493808	1.32E-05	0.000116323
PARG0745	1.339987129	5.630375441	1.33E-05	0.000117356
PARG1806	-1.237917101	3.651741646	1.34E-05	0.000118184
PARG2302	-1.17768659	5.849478903	1.36E-05	0.000120271
PARG1403	1.166764645	8.493246106	1.36E-05	0.000120296
PARG1442	1.355520553	6.030457351	1.37E-05	0.000120598
PARG0122	1.30276624	6.161981339	1.37E-05	0.000120843
PARG1462	2.404698751	-0.25634687	1.38E-05	0.000121749
PARG1244	-1.17944968	5.640520453	1.39E-05	0.000122163
PARG0134	-1.227303019	3.909095067	1.40E-05	0.000123319
PARG1617	1.166433199	7.60648938	1.42E-05	0.000124996
PARG2843	1.174697436	5.472115126	1.45E-05	0.00012757
PARG1191	-1.587854767	4.639281226	1.45E-05	0.00012757
PARG2345	1.192604425	6.71898999	1.46E-05	0.000127758
PARG0400	1.22105129	4.70408808	1.47E-05	0.00012848
PARG2716	-1.177144359	5.559593859	1.47E-05	0.000128534

PARG2681	-1.752768122	1.622883446	1.47E-05	0.000128795
PARG2656	-1.311596822	2.134629261	1.48E-05	0.000129754
PARG0754	-1.170516916	5.450570546	1.48E-05	0.000129754
PARG2384	1.975675237	1.19372406	1.49E-05	0.000130329
PARG0891	1.165485177	6.61256699	1.50E-05	0.000131287
PARG0120	-1.229533534	4.100502715	1.51E-05	0.000131481
PARG0454	-4.084980294	-0.658443363	1.51E-05	0.00013184
PARG0590	-2.928893533	-0.195801175	1.51E-05	0.000132064
PARG1031	2.894530946	0.722177283	1.51E-05	0.000132064
PARG1608	1.807738246	3.86885929	1.54E-05	0.000134056
PARG2673	1.261964362	4.110669651	1.54E-05	0.000134437
PARG1008	-1.167621444	6.700836026	1.54E-05	0.000134487
PARG0752	1.157788638	9.220336985	1.55E-05	0.000134678
PARG1365	-1.217104594	4.195491194	1.55E-05	0.000135124
PARG1306	1.207644359	4.945934318	1.56E-05	0.000135293
PARG1505	1.208249779	4.890895363	1.56E-05	0.000135294
PARG0827	1.26922276	4.780087817	1.58E-05	0.000136794
PARG1533	-1.559837818	1.985901121	1.59E-05	0.000137763
PARG1837	-1.80077834	2.5265921	1.60E-05	0.000139044
PARG2510	-1.39294153	3.983112605	1.62E-05	0.000140037
PARG0541	-1.195711672	4.132840393	1.65E-05	0.000143052
PARG1723	1.182029105	5.382477558	1.66E-05	0.000143579
PARG2418	1.153298364	7.651244289	1.67E-05	0.00014453
PARG0389	-1.759479276	0.185102859	1.67E-05	0.000144861
PARG2628	1.154056451	9.020360342	1.68E-05	0.00014516
PARG2775	-1.986443697	-0.340957793	1.71E-05	0.000147617
PARG0109	-1.360951158	2.475694915	1.72E-05	0.000148707
PARG2571	-1.291477009	4.333193894	1.72E-05	0.000148839
PARG2796	1.619848615	0.426326415	1.73E-05	0.000148914
PARG1239	-2.158304927	0.876632294	1.73E-05	0.000148995
PARG1682	1.451921176	4.404977238	1.74E-05	0.000149806
PARG0765	1.327183283	4.517949943	1.75E-05	0.000150653
PARG2520	-1.465354577	5.157504887	1.76E-05	0.00015186
PARG0784	1.301119548	2.745929077	1.78E-05	0.000153354
PARG0164	-1.170056498	6.374521863	1.81E-05	0.000155243
PARG2516	-1.478314352	1.860814846	1.81E-05	0.000155479
PARG1659	-1.375609072	2.048109927	1.81E-05	0.00015567
PARG0811	-1.247628249	3.517960101	1.81E-05	0.00015567
PARG0559	1.201810987	2.728274933	1.82E-05	0.000156505
PARG0054	1.20253698	4.693319793	1.83E-05	0.000156694
PARG2581	-1.167585277	5.365987054	1.86E-05	0.000159073
PARG0299	-1.442427858	1.084773001	1.88E-05	0.000160772
PARG0076	-2.49634523	0.017998363	1.91E-05	0.000163275
PARG1715	-1.931571891	0.979563823	1.91E-05	0.00016343
PARG1902	1.205187818	5.938165481	1.94E-05	0.000165464
PARG1298	-1.202696745	4.290293343	1.94E-05	0.000166199

PARG0472	1.227489248	5.01094614	1.96E-05	0.000167058
PARG2507	-1.217069716	4.25850564	1.96E-05	0.000167459
PARG2166	-2.18844025	1.529834236	1.98E-05	0.000169029
PARG1620	-2.077715791	0.211180567	2.00E-05	0.000170178
PARG0036	1.141407282	8.2241675	2.01E-05	0.0001709
PARG1253	-1.154023436	6.984481391	2.01E-05	0.000171185
PARG0034	-3.48459082	-0.362970087	2.01E-05	0.000171297
PARG2535	3.476475486	-0.195625027	2.01E-05	0.000171297
PARG1624	1.144002452	7.104777085	2.04E-05	0.000173326
PARG0061	1.247118815	4.518857901	2.05E-05	0.00017405
PARG1670	1.461967291	1.783265122	2.05E-05	0.000174144
PARG0882	-1.161764133	5.363920784	2.05E-05	0.000174302
PARG2764	-1.3603568	3.157926484	2.07E-05	0.000175477
PARG0725	1.185794053	4.656494716	2.08E-05	0.000176363
PARG2728	2.552419914	0.437571465	2.08E-05	0.000176363
PARG0730	-1.305509484	4.469487283	2.09E-05	0.000177056
PARG0841	1.153208425	6.142700941	2.09E-05	0.000177158
PARG0559	-1.146203583	6.884903532	2.10E-05	0.000178025
PARG0323	1.933884449	-0.673262941	2.12E-05	0.000179063
PARG0579	-1.141192903	7.843569131	2.12E-05	0.000179553
PARG1208	1.597163988	1.527196803	2.15E-05	0.000181886
PARG2053	-1.137489393	8.378837143	2.16E-05	0.000182182
PARG2683	1.191523786	6.033112743	2.20E-05	0.00018592
PARG1666	1.143878783	6.880392587	2.21E-05	0.0001863
PARG1237	-2.722594558	0.69434419	2.22E-05	0.000187416
PARG0883	1.151094593	5.793190338	2.23E-05	0.000187951
PARG1384	-1.159468053	4.712562378	2.27E-05	0.000190864
PARG1241	1.380250986	2.422819607	2.29E-05	0.000192536
PARG0346	1.309240817	2.488931135	2.31E-05	0.000194343
PARG2045	1.134059081	7.279238205	2.32E-05	0.000194914
PARG0887	-1.830200216	-0.11220362	2.33E-05	0.000195456
PARG1232	-5.627144441	-0.60793461	2.33E-05	0.000195456
PARG0884	5.592015388	0.846449587	2.33E-05	0.000195456
PARG0732	-1.330861716	4.265863074	2.33E-05	0.000195456
PARG1903	1.160927777	5.2959517	2.33E-05	0.000195456
PARG1262	-1.159318467	6.310237923	2.34E-05	0.000196222
PARG0696	1.150602243	4.822448747	2.36E-05	0.00019734
PARG1395	-1.305184659	2.986421774	2.36E-05	0.00019734
PARG0434	-1.138181696	6.897781357	2.38E-05	0.000198678
PARG2930	-1.434309232	1.491583566	2.38E-05	0.000198678
PARG1988	-1.680265648	4.149798903	2.39E-05	0.000199606
PARG2182	1.228927095	3.564039163	2.39E-05	0.000199606
PARG1848	1.564325236	7.505257791	2.41E-05	0.000201194
PARG0801	1.181820442	4.828724625	2.42E-05	0.000201833
PARG1988	-1.141975433	5.644471229	2.43E-05	0.000202629
PARG0831	-1.136760458	7.003564976	2.44E-05	0.000203468

PARG0667	-1.840973583	0.668852055	2.45E-05	0.000204078
PARG1884	1.157160942	5.610782121	2.46E-05	0.000204978
PARG2710	-1.136705306	6.346056743	2.46E-05	0.000205036
PARG2793	1.243553558	3.264813274	2.47E-05	0.000205787
PARG0320	1.234058863	2.43124357	2.63E-05	0.000218828
PARG1021	-1.133540831	6.04070632	2.66E-05	0.000220757
PARG0184	-1.193357821	4.11862108	2.67E-05	0.000221528
PARG0612	1.225656533	2.680462515	2.69E-05	0.000222988
PARG0536	1.20467905	5.415144234	2.72E-05	0.000225394
PARG2241	1.40332591	2.641314734	2.74E-05	0.000227391
PARG0603	1.123411299	8.747027746	2.75E-05	0.000227548
PARG0847	1.613454768	0.618146473	2.75E-05	0.000227702
PARG2355	1.497714792	1.050294433	2.82E-05	0.000233382
PARG1493	-1.138080546	5.773264133	2.83E-05	0.000234405
PARG2344	-1.289240913	2.088938577	2.84E-05	0.000234519
PARG2113	1.325395142	3.197227349	2.85E-05	0.000235438
PARG1608	1.216034515	3.489894668	2.86E-05	0.00023643
PARG2255	-1.164326223	4.371621051	2.87E-05	0.000237389
PARG0843	-1.15706927	4.727083967	2.88E-05	0.000237547
PARG1959	-1.314945355	3.293353912	2.88E-05	0.000237731
PARG1869	1.121334617	7.184054886	2.90E-05	0.0002388
PARG2775	1.124512665	5.920677439	2.91E-05	0.000239702
PARG0503	1.148981252	4.329543725	2.93E-05	0.000241102
PARG1585	-1.174420365	3.86747141	2.93E-05	0.000241243
PARG1545	1.140294966	5.545448279	2.93E-05	0.000241355
PARG0120	-1.738829944	3.221642549	2.95E-05	0.000242762
PARG1407	1.366488298	3.71935222	2.96E-05	0.000243506
PARG2665	1.138347575	5.208313398	2.99E-05	0.000245369
PARG1857	-2.31109105	0.286922593	3.00E-05	0.000246307
PARG2512	-1.136759677	5.452735491	3.00E-05	0.000246307
PARG1045	-1.524228069	1.95090491	3.02E-05	0.000247382
PARG2740	-2.556985652	0.06458022	3.03E-05	0.000248452
PARG1141	2.519446379	1.271562367	3.03E-05	0.000248452
PARG2730	-1.18273218	5.440926645	3.04E-05	0.000249106
PARG0531	-1.123340006	5.557381466	3.05E-05	0.000249831
PARG2793	1.142644577	4.319590517	3.07E-05	0.000251293
PARG1225	-1.366289943	1.197720115	3.08E-05	0.000252217
PARG2995	1.175293793	6.196001655	3.11E-05	0.000254052
PARG0714	-1.133375365	6.120126411	3.13E-05	0.00025597
PARG1845	-1.246086042	3.185211603	3.15E-05	0.00025722
PARG1464	-1.117759196	7.26805591	3.17E-05	0.000258758
PARG1302	1.135705004	6.405171667	3.19E-05	0.000259962
PARG2027	-1.113713031	8.710497828	3.21E-05	0.000261327
PARG1551	-1.427003872	3.543575407	3.22E-05	0.000262471
PARG1031	3.387604037	1.649867606	3.24E-05	0.000263961
PARG1847	1.111030577	8.471933642	3.26E-05	0.000265261

PARG0908	1.190898716	3.290105418	3.27E-05	0.000265805
PARG1462	-1.560565869	1.673556887	3.27E-05	0.000265805
PARG2968	-1.560565869	1.673556887	3.27E-05	0.000265805
PARG2214	-1.232499852	3.391273099	3.30E-05	0.000267947
PARG0191	-1.122388755	4.794801611	3.32E-05	0.000269695
PARG1232	1.924067434	0.348872047	3.32E-05	0.000269736
PARG2047	1.168564014	4.88056598	3.36E-05	0.000272129
PARG2219	-1.688226547	0.006342512	3.41E-05	0.000276334
PARG2439	1.305053043	3.217324985	3.41E-05	0.000276371
PARG1248	1.619941982	2.586986685	3.42E-05	0.000276469
PARG0866	1.126425844	5.705068647	3.42E-05	0.000276469
PARG1061	1.135005115	5.975474699	3.43E-05	0.000277125
PARG0862	-2.37406521	0.698692171	3.44E-05	0.000277821
PARG1990	1.172500394	4.765212932	3.44E-05	0.000277821
PARG2682	1.97388654	-0.374345346	3.48E-05	0.00028131
PARG0295	1.148070927	5.094325125	3.49E-05	0.000281485
PARG0900	1.154333976	4.727562998	3.52E-05	0.000283898
PARG1884	1.113004843	5.345647396	3.53E-05	0.000284644
PARG2669	-1.132979419	5.377150018	3.63E-05	0.000292341
PARG0141	1.228769613	3.34103233	3.63E-05	0.000292581
PARG2504	1.186116335	3.645291917	3.64E-05	0.000292982
PARG0016	-1.126660842	3.958563399	3.66E-05	0.000294588
PARG0531	-1.115942978	6.034675194	3.67E-05	0.000295036
PARG2483	1.245090004	3.363653211	3.69E-05	0.000296548
PARG2703	1.858546294	3.64195293	3.73E-05	0.000299881
PARG0895	-1.143367452	4.354296476	3.79E-05	0.000304681
PARG2931	-1.249072147	3.166939892	3.81E-05	0.000305991
PARG1310	-1.169745021	4.140601887	3.82E-05	0.000306592
PARG1293	-1.099860223	11.59509161	3.84E-05	0.000307786
PARG0923	1.192003997	4.022356025	3.88E-05	0.000310843
PARG2834	1.598182666	0.414129804	3.89E-05	0.000311888
PARG2061	2.614332614	-0.015982991	3.92E-05	0.000313451
PARG0803	2.380023762	0.995853793	3.92E-05	0.000313451
PARG1299	-1.130817863	5.566378702	3.97E-05	0.000317266
PARG2643	1.111608738	6.562348786	3.97E-05	0.000317281
PARG2738	-1.101879652	6.812555639	3.97E-05	0.000317336
PARG0456	1.107778808	5.764707638	3.98E-05	0.000317474
PARG2715	1.167356732	4.968458017	3.99E-05	0.000318263
PARG1327	1.274546318	4.237314974	4.05E-05	0.000322696
PARG0775	1.110903672	5.906533545	4.07E-05	0.000324154
PARG2468	-1.119252585	6.761875207	4.08E-05	0.000325021
PARG2922	2.117006909	3.076707782	4.09E-05	0.000325851
PARG1415	2.022650557	3.645522869	4.09E-05	0.000325851
PARG1414	1.102666692	6.78440888	4.12E-05	0.000327893
PARG2103	-5.554411858	1.382371537	4.14E-05	0.000329105
PARG1655	5.520688627	0.413192231	4.14E-05	0.000329105

PARG2782	5.518409165	-0.109799925	4.14E-05	0.000329105
PARG2337	1.206130948	3.513283078	4.19E-05	0.000332442
PARG1186	-1.099459817	7.138212719	4.25E-05	0.000336524
PARG1832	-3.988928082	-0.378679244	4.25E-05	0.000336524
PARG2421	3.935587677	2.219418763	4.25E-05	0.000336524
PARG0713	3.9203856	-0.922671168	4.25E-05	0.000336524
PARG1206	1.326161221	2.018365829	4.30E-05	0.000340235
PARG0218	1.164358751	4.64409117	4.30E-05	0.000340235
PARG1979	1.259762433	3.06722351	4.30E-05	0.000340496
PARG0321	-1.780643065	0.482503861	4.32E-05	0.000341954
PARG1426	1.284881405	2.770336036	4.37E-05	0.000345579
PARG0047	1.344238107	3.181636186	4.38E-05	0.000345688
PARG1564	1.866249319	2.077537232	4.38E-05	0.000345688
PARG1707	-1.285891866	2.167284717	4.42E-05	0.000349086
PARG2789	-1.095520078	7.202907701	4.44E-05	0.000350334
PARG0241	1.352098179	2.539115526	4.46E-05	0.000351544
PARG0404	1.103280987	6.182003137	4.47E-05	0.000352203
PARG0945	1.653823234	2.384577196	4.48E-05	0.000353101
PARG0484	-1.140574693	4.645035645	4.51E-05	0.000355118
PARG1651	1.092002409	7.91626374	4.52E-05	0.00035535
PARG0248	1.219358327	2.008411468	4.61E-05	0.000362187
PARG2793	-1.65495022	-0.08201327	4.70E-05	0.000369607
PARG0745	-1.09106499	7.949539538	4.73E-05	0.000371876
PARG1668	1.169146059	4.201431085	4.75E-05	0.000373178
PARG0333	-1.415733394	3.977255087	4.80E-05	0.000377077
PARG0363	-1.283147479	2.922251531	4.90E-05	0.000384138
PARG2882	-1.385433169	1.728423757	4.90E-05	0.000384568
PARG1953	2.634571751	2.78237558	4.91E-05	0.000385146
PARG0510	1.208220464	3.281559253	4.94E-05	0.000386832
PARG1593	1.383540194	3.321004119	4.97E-05	0.000388816
PARG1595	1.089688019	7.817272105	4.97E-05	0.000388933
PARG1448	-1.372941607	1.675968434	5.00E-05	0.000390821
PARG1420	-1.265195531	1.174512118	5.04E-05	0.000393789
PARG2381	1.197666449	2.932731831	5.05E-05	0.000394227
PARG2754	-1.09415931	7.102365941	5.05E-05	0.000394451
PARG1189	1.313264801	3.413064476	5.08E-05	0.000396224
PARG0746	1.920872159	1.049827533	5.11E-05	0.000398831
PARG0272	1.920577029	0.519092826	5.11E-05	0.000398831
PARG1623	-1.132284315	4.735238835	5.15E-05	0.000401042
PARG0045	-1.778512516	1.415362499	5.16E-05	0.000402242
PARG1970	1.155675205	3.178115656	5.17E-05	0.000402455
PARG2499	-3.36152539	1.347401671	5.23E-05	0.000407058
PARG0577	3.320722325	1.877796404	5.23E-05	0.000407058
PARG0834	-1.083986708	7.420463232	5.29E-05	0.000411163
PARG0044	1.082686675	6.692869067	5.31E-05	0.000412679
PARG1708	2.779281847	-0.516898708	5.32E-05	0.000412688

PARG2634	2.766271893	2.981523704	5.32E-05	0.000412688
PARG1844	1.094419779	4.939367045	5.33E-05	0.000413317
PARG1512	1.097622112	6.089432188	5.34E-05	0.000413894
PARG2573	1.407825213	2.949827106	5.34E-05	0.000413894
PARG1264	-1.10801473	5.589871737	5.36E-05	0.000414993
PARG0013	-1.207915671	2.972487917	5.45E-05	0.000422234
PARG2051	-1.081672053	7.756310508	5.47E-05	0.000423463
PARG2750	1.184684739	3.190487279	5.49E-05	0.000424996
PARG0657	-1.216968789	2.549479027	5.50E-05	0.000425552
PARG0290	1.120493464	4.934326635	5.52E-05	0.000426494
PARG2758	-1.07730832	8.25738808	5.57E-05	0.000430331
PARG0277	-1.109086481	6.079804648	5.60E-05	0.000432331
PARG2774	1.24094851	2.506763686	5.61E-05	0.000433181
PARG1530	1.09620819	4.552271806	5.71E-05	0.000440104
PARG0716	-2.119445808	1.041880436	5.72E-05	0.000441294
PARG1620	1.24219952	3.053312384	5.78E-05	0.000445609
PARG0169	-2.251930072	-0.151186458	5.82E-05	0.000448354
PARG1372	2.197711796	-0.671752507	5.82E-05	0.000448354
PARG2021	-1.085000161	6.116827228	5.84E-05	0.000448906
PARG0833	-1.136029984	5.072911393	5.84E-05	0.000448906
PARG2768	1.567496035	1.570757196	5.88E-05	0.000452086
PARG1521	-1.502839899	1.775394938	5.89E-05	0.000452237
PARG1871	1.182818714	3.353331996	5.93E-05	0.00045516
PARG2905	1.106243365	6.055978885	6.00E-05	0.000460428
PARG2664	-1.179501082	4.444305886	6.01E-05	0.000461409
PARG1580	2.019137011	2.718165454	6.08E-05	0.000466527
PARG2314	-1.131758713	3.889759153	6.09E-05	0.000466746
PARG0268	-1.098163656	5.919604318	6.11E-05	0.000468428
PARG2116	1.898789222	3.528964651	6.13E-05	0.000469053
PARG1875	1.084205103	5.948663956	6.16E-05	0.000471156
PARG1276	1.106004239	5.347332706	6.23E-05	0.000476865
PARG1273	1.079829444	6.538376384	6.25E-05	0.000477834
PARG2559	-1.175483886	3.925392288	6.25E-05	0.000477834
PARG2765	1.121371219	4.663135964	6.27E-05	0.000479002
PARG0347	1.072214378	8.027189122	6.33E-05	0.000483104
PARG2298	1.321970555	3.191685269	6.34E-05	0.000483559
PARG1669	1.252680184	3.409695197	6.36E-05	0.000484928
PARG0869	1.188124764	3.0662015	6.45E-05	0.000491383
PARG1567	-1.07873575	6.534068468	6.46E-05	0.000492444
PARG2369	1.106409243	4.334346032	6.52E-05	0.00049672
PARG0968	1.125331032	4.845244167	6.60E-05	0.000502174
PARG0843	-1.070724204	6.764465626	6.66E-05	0.000507031
PARG2413	-1.23128226	3.727955626	6.67E-05	0.000507031
PARG2710	1.194840681	5.574000026	6.68E-05	0.000507571
PARG1290	-1.072281944	6.163616631	6.69E-05	0.000508484
PARG0076	1.111704772	5.451178849	6.70E-05	0.000508513

PARG2728	1.970498846	4.197876572	6.70E-05	0.000508642
PARG0778	1.178702178	3.027815714	6.70E-05	0.000508642
PARG1732	1.675268571	0.657552168	6.72E-05	0.000509982
PARG2957	-1.090878063	5.672073525	6.74E-05	0.000510538
PARG1295	1.064096337	10.59894961	6.74E-05	0.000510538
PARG3034	-1.107546017	4.392003264	6.74E-05	0.000510538
PARG1214	-2.144588359	-0.229201521	6.81E-05	0.000515133
PARG2297	-1.107273464	5.167138524	6.81E-05	0.000515549
PARG1048	1.336839566	1.559915913	6.84E-05	0.000517274
PARG1322	2.433352777	5.092055756	6.86E-05	0.000517986
PARG0914	-2.27462474	0.48715025	6.86E-05	0.000517986
PARG2745	1.356925517	3.526591479	6.86E-05	0.000517986
PARG1280	1.16359933	3.547638927	6.87E-05	0.000518208
PARG1913	1.145489561	4.01378364	6.88E-05	0.000519258
PARG2466	1.142464631	5.129292605	6.95E-05	0.000524065
PARG1806	1.430591688	1.654023825	6.96E-05	0.000524709
PARG1967	-1.152477785	4.184339927	6.97E-05	0.00052535
PARG0131	1.09338811	5.434079081	7.01E-05	0.000527946
PARG2093	1.067461189	6.733608458	7.06E-05	0.000531599
PARG1760	1.152069675	3.393020054	7.08E-05	0.000532192
PARG2691	-3.899292453	3.8338281	7.16E-05	0.000538227
PARG2953	1.687638791	1.198945545	7.16E-05	0.000538418
PARG2581	1.069697555	6.610235881	7.21E-05	0.000541656
PARG1407	-1.258983869	4.784244602	7.25E-05	0.000544513
PARG1642	-1.066385269	5.995352954	7.31E-05	0.000548322
PARG2815	2.56229062	4.264810704	7.32E-05	0.000548322
PARG1343	2.556722478	2.762159656	7.32E-05	0.000548322
PARG2828	-1.060210331	9.747171006	7.32E-05	0.000548322
PARG1982	1.062495116	7.554592279	7.33E-05	0.000549257
PARG1391	-1.097661763	4.607969519	7.39E-05	0.000553511
PARG0888	5.496784694	-0.708044898	7.41E-05	0.000553929
PARG0539	-5.461678446	-0.58967314	7.41E-05	0.000553929
PARG2907	5.429720899	-0.45446703	7.41E-05	0.000553929
PARG2014	1.175103982	3.852334062	7.42E-05	0.000553929
PARG2057	1.134529888	5.924986593	7.42E-05	0.000553929
PARG1426	1.077793705	5.334059432	7.53E-05	0.000561975
PARG1261	1.181078026	3.104820059	7.54E-05	0.000562307
PARG2722	-1.117808343	4.326980501	7.54E-05	0.000562307
PARG2496	1.087278573	4.634584195	7.58E-05	0.00056507
PARG0894	1.615085575	1.70940055	7.63E-05	0.000568775
PARG2824	1.067738918	6.981935819	7.64E-05	0.000568777
PARG0911	1.056886297	8.322715316	7.67E-05	0.000570597
PARG1885	1.059599021	7.18482342	7.69E-05	0.000572433
PARG2830	-1.062855517	7.159361711	7.72E-05	0.000574119
PARG2240	1.120286239	4.838533175	7.73E-05	0.000574337
PARG1977	1.088387925	5.366792931	7.75E-05	0.000575962

PARG1611	-1.350884246	1.614989864	7.80E-05	0.000579454
PARG2326	1.149548692	3.256933206	7.93E-05	0.000588524
PARG2938	1.073795043	6.343916127	7.95E-05	0.000590123
PARG2929	1.089678274	4.817287123	8.00E-05	0.000593216
PARG1942	-1.144102666	4.512954099	8.01E-05	0.000593332
PARG2737	-1.72822161	3.41196716	8.04E-05	0.000595708
PARG0918	-2.378270034	0.677476979	8.06E-05	0.00059706
PARG0601	1.072645681	4.873583631	8.07E-05	0.000597211
PARG0400	2.734085808	-0.226116914	8.10E-05	0.000599087
PARG1972	1.062009338	6.864888763	8.11E-05	0.000599646
PARG0489	-1.384739024	2.956656016	8.21E-05	0.000606908
PARG2239	-1.050699971	13.43458345	8.22E-05	0.000607285
PARG1811	1.203799221	8.23317952	8.40E-05	0.000620158
PARG2829	-1.086568772	5.48197224	8.43E-05	0.000622526
PARG0586	1.450303061	3.36224936	8.46E-05	0.000623751
PARG2066	-3.282981322	-0.550027853	8.47E-05	0.00062419
PARG0342	-1.716374014	1.659866641	8.49E-05	0.000625692
PARG2385	-1.21927352	3.860041065	8.55E-05	0.000629712
PARG0973	-1.151273833	3.142097176	8.58E-05	0.000631898
PARG2635	1.231905752	3.6671821	8.67E-05	0.000638332
PARG2930	-2.034030466	-0.104610621	8.76E-05	0.000643813
PARG2058	1.999058194	2.529675524	8.76E-05	0.000643813
PARG1937	-1.896982589	0.061919969	8.78E-05	0.000645245
PARG1888	-1.896475572	2.145380054	8.78E-05	0.000645245
PARG0270	-1.154151401	4.863477351	8.81E-05	0.000646639
PARG0748	1.179108649	3.0262273	8.85E-05	0.000649492
PARG2606	1.109924759	4.816554083	8.92E-05	0.000654651
PARG1214	1.111666417	4.003819829	9.15E-05	0.000670613
PARG2685	-1.241692913	3.352405666	9.25E-05	0.000677428
PARG0397	1.051476254	7.120881281	9.25E-05	0.000677428
PARG1847	1.125359395	2.428421327	9.27E-05	0.000678836
PARG1454	1.144535305	2.718546027	9.28E-05	0.000679302
PARG2809	1.207756159	1.860489308	9.29E-05	0.000679517
PARG0770	-1.04595735	7.396408848	9.30E-05	0.000679617
PARG1379	-1.10147888	3.951019364	9.30E-05	0.000679852
PARG1955	1.242822201	2.967551729	9.31E-05	0.000680021
PARG0813	1.686872313	3.434751264	9.35E-05	0.000682035
PARG1948	-1.647172759	0.119217406	9.35E-05	0.000682035
PARG0455	2.089225362	-0.159982054	9.37E-05	0.00068328
PARG0839	1.108998183	5.376494728	9.42E-05	0.000687039
PARG2014	-2.472830399	2.463101386	9.43E-05	0.000687039
PARG2725	1.086023878	4.290516812	9.44E-05	0.000687933
PARG2955	1.154310058	3.635555871	9.46E-05	0.000688902
PARG0554	-1.097805822	4.648925776	9.55E-05	0.00069483
PARG2450	1.421911236	2.890283443	9.55E-05	0.00069483
PARG0338	1.329254851	1.146938955	9.57E-05	0.000695541

PARG1845	-1.053963181	7.076794764	9.60E-05	0.000697782
PARG2356	1.051548232	5.297842486	9.69E-05	0.00070393
PARG0812	1.04102626	9.992019226	9.70E-05	0.000704252
PARG0725	2.014738203	0.094225271	9.70E-05	0.000704252
PARG1379	1.480137411	7.638701005	9.72E-05	0.000705302
PARG2167	-1.084292034	3.802504997	9.74E-05	0.000705903
PARG2326	1.114374294	3.308774633	9.80E-05	0.000710339
PARG0451	-1.340344965	2.395056625	9.84E-05	0.000712777
PARG1535	-1.057004019	5.833022227	9.85E-05	0.000713318
PARG1269	-1.043807879	7.515395961	9.87E-05	0.000714173
PARG1910	-1.110686262	4.035235137	9.93E-05	0.000718369
PARG1288	1.050425033	6.636798006	9.94E-05	0.00071846
PARG1756	1.243029553	2.216905941	9.98E-05	0.000721316
PARG1007	1.114535656	3.596441652	0.000100118	0.000723262
PARG1056	-1.044667153	6.873001254	0.000101055	0.000729699
PARG0020	1.091635994	4.493964127	0.000101331	0.000731353
PARG2465	-1.597346238	3.660451811	0.000101444	0.000731831
PARG2061	1.844785259	3.887019812	0.000102071	0.000736018
PARG1754	-1.046764583	6.579233404	0.000102512	0.000738862
PARG1857	-1.201894898	2.005115165	0.000102579	0.000739
PARG1417	1.297307353	1.444862651	0.000104515	0.000752605
PARG1584	1.059923549	8.71924524	0.000104582	0.000752742
PARG2720	-1.038944179	8.001822689	0.000105002	0.00075542
PARG1266	1.051374597	6.970981436	0.000105133	0.000756011
PARG2213	-1.2870016	1.714302945	0.000105463	0.000758037
PARG1801	1.714737754	0.800774148	0.000106285	0.000763597
PARG2031	1.060744487	5.336681959	0.000106487	0.000764365
PARG1966	-1.080479346	5.160737184	0.000106489	0.000764365
PARG2420	-1.087228936	5.690289932	0.000106703	0.000765549
PARG2148	-1.046390837	7.475755239	0.000107709	0.00077218
PARG1155	1.171776416	4.178617172	0.000107726	0.00077218
PARG1511	-1.124707456	6.00764475	0.000108185	0.000775117
PARG2631	1.222898038	2.935395133	0.000108666	0.000778211
PARG2159	-1.553890809	2.012153845	0.000108864	0.000779271
PARG0895	1.174455929	3.023231283	0.000109144	0.000780925
PARG0205	-1.127009824	3.146494738	0.000110256	0.000788516
PARG0860	1.34808003	3.853323602	0.000110476	0.000789729
PARG1870	-1.107917103	4.10437487	0.000110584	0.000790145
PARG1365	-1.065466119	6.671533431	0.000110693	0.000790564
PARG2322	-1.059951222	4.762499613	0.000111116	0.000793225
PARG1523	1.041419527	5.998852192	0.000112141	0.000800176
PARG0203	1.044064889	6.163171523	0.000113949	0.000812711
PARG0050	1.052221415	6.846842978	0.000115	0.000819829
PARG2346	-1.38833512	3.583565211	0.000115349	0.00082195
PARG2075	-2.33606773	0.239528543	0.000115743	0.000824007
PARG2477	2.266388368	-0.312473303	0.000115743	0.000824007

PARG2113	1.177679586	5.857641455	0.000116903	0.000831887
PARG1006	1.036674057	8.411019503	0.000117022	0.000832355
PARG0052	-1.060964506	4.599118146	0.00011712	0.000832648
PARG0208	1.471546601	3.334837659	0.000117275	0.000832648
PARG0455	1.465017347	0.947778759	0.000117275	0.000832648
PARG2275	1.452616208	3.038428293	0.000117275	0.000832648
PARG0242	-1.086559705	3.958178963	0.000117975	0.000837236
PARG0912	-1.029460307	8.714443127	0.000118583	0.000841007
PARG1777	1.041816353	5.344777572	0.000118613	0.000841007
PARG1947	1.123114221	3.964001283	0.00011874	0.000841523
PARG2117	-1.257082244	2.221887304	0.000118954	0.000841959
PARG2846	1.573722833	1.762601522	0.000118962	0.000841959
PARG0919	1.550904917	3.400504723	0.000118962	0.000841959
PARG0058	-1.062811937	4.705825551	0.000119139	0.00084283
PARG0467	-3.83388241	0.892262218	0.000121093	0.000856268
PARG2747	1.082307554	4.631434877	0.00012132	0.000857487
PARG2540	1.053827292	5.066311489	0.000122784	0.000867446
PARG1785	2.69800612	-0.284552047	0.000123497	0.000871699
PARG1289	2.659838705	0.323713678	0.000123497	0.000871699
PARG2780	1.787772658	4.389644001	0.00012361	0.0008721
PARG1745	1.329614081	1.839866759	0.000124031	0.00087468
PARG2102	-1.03473273	6.572739786	0.000125184	0.000882417
PARG2434	1.149823128	2.650535688	0.000125414	0.000883637
PARG0802	1.111460584	3.585951705	0.000125674	0.000884688
PARG1296	-1.08445243	2.977554313	0.000125675	0.000884688
PARG0518	1.182798948	2.9099351	0.000125894	0.000885827
PARG0229	1.050576267	5.481915784	0.000126046	0.0008865
PARG0320	1.181954777	2.905592112	0.000126308	0.000887944
PARG0615	1.028621035	6.933047894	0.000127131	0.000893333
PARG0281	1.195142418	3.431508602	0.000127192	0.000893359
PARG2970	-1.063325149	4.787696426	0.000127306	0.000893761
PARG1439	-1.100146878	3.472990206	0.000127447	0.000894352
PARG0600	1.60190752	5.881364148	0.000128656	0.000902433
PARG2224	1.285816005	1.602053726	0.000128927	0.000903925
PARG0447	-1.042096814	5.199660486	0.000129005	0.000903995
PARG2141	1.130349431	3.704492291	0.000129052	0.000903995
PARG2338	1.068140965	5.05031386	0.000130162	0.000911369
PARG1853	-1.025196357	7.306879285	0.00013025	0.000911574
PARG2284	-1.128671338	3.580641019	0.000130674	0.00091373
PARG2460	-1.033702427	4.934882515	0.00013072	0.00091373
PARG0624	-1.036707769	6.396338576	0.000130732	0.00091373
PARG2279	1.905672661	1.371013872	0.000131735	0.000919984
PARG1694	-1.038079861	7.557910268	0.000131744	0.000919984
PARG0856	1.103231442	4.517631721	0.000133307	0.000930483
PARG0180	-5.389582283	1.106178451	0.000133436	0.000930776
PARG1711	-1.019151956	11.1684675	0.000133498	0.000930776

PARG2005	1.051255883	4.915431514	0.000133527	0.000930776
PARG1521	-1.057927043	5.037428696	0.000133736	0.000931821
PARG0432	-1.171404054	2.989794131	0.000135581	0.000944255
PARG0588	1.485496901	2.201803798	0.000135787	0.000945271
PARG0534	2.156588376	-0.400775854	0.000137013	0.000953384
PARG1810	-3.23600687	-0.698021412	0.000137243	0.000954563
PARG0884	1.200136076	2.650750533	0.000137962	0.000959136
PARG1632	1.041643508	4.708963273	0.000138567	0.000962917
PARG0701	1.361859216	2.061330495	0.000141298	0.000981456
PARG1862	-1.029923185	5.961417986	0.000141856	0.0009849
PARG2123	-1.048764913	5.582164861	0.000143795	0.000997918
PARG1380	1.012647087	10.69184471	0.000146406	0.001015593
PARG2020	1.151089738	3.498812529	0.000146506	0.001015837
PARG0136	1.017981899	5.94419924	0.00014729	0.001020821
PARG1524	1.028402381	6.142441651	0.000148883	0.001031409
PARG2591	1.024217593	6.074425282	0.000149027	0.001031951
PARG0232	1.070351537	4.119298884	0.000152107	0.001052813
PARG1376	1.058761232	3.905519095	0.000152324	0.00105385
PARG0555	-1.233808786	1.814643127	0.000152518	0.001054728
PARG0458	-1.016156429	6.757187405	0.00015309	0.001058217
PARG2646	1.191816533	3.186419346	0.000154074	0.00106455
PARG2055	-1.455086465	4.875176285	0.000155255	0.001072236
PARG0777	1.226934634	2.868235982	0.000155358	0.001072473
PARG0075	-1.023965103	4.765212861	0.000155797	0.001075032
PARG1292	-1.025255719	3.897305928	0.000156714	0.001080884
PARG1861	1.008955158	8.238809664	0.000157607	0.001086566
PARG1650	-1.009314792	8.010268291	0.00015805	0.001089146
PARG2406	2.044782981	0.283277542	0.000158702	0.001093153
PARG0726	1.072033317	5.625981594	0.000160167	0.001102761
PARG1247	-1.200953032	3.505498228	0.000161572	0.001111952
PARG0610	1.109418601	3.261754834	0.000164928	0.001134546
PARG1759	1.030110622	4.184181299	0.000165457	0.001137673
PARG1943	-1.017061096	7.285988288	0.000165527	0.001137673
PARG2680	1.007479236	8.078043174	0.000165718	0.001138483
PARG0028	-1.004337808	9.191145857	0.000166122	0.001140762
PARG2806	1.010955467	5.847130876	0.00016796	0.001152883
PARG0306	-1.103404616	4.091994073	0.000169117	0.001160016
PARG1040	1.01338931	6.102110737	0.000169147	0.001160016
PARG0535	1.091892079	3.088513953	0.000170114	0.001166132
PARG0867	1.004954241	8.122651869	0.00017081	0.001170398
PARG2723	1.042554454	4.565859316	0.000170984	0.001171073
PARG0166	1.015517833	6.003112319	0.000172323	0.00117921
PARG1990	1.871587051	-0.148837149	0.000172471	0.00117921
PARG0074	1.862087914	0.224811621	0.000172471	0.00117921
PARG0109	-1.138235486	1.75974924	0.000172472	0.00117921
PARG0728	-1.202954902	5.485674749	0.00017286	0.001181348

PARG1741	-1.125887999	3.494385271	0.000173663	0.001186322
PARG1720	1.29618827	0.028435136	0.000173773	0.001186555
PARG1134	1.007592519	7.216695896	0.000174614	0.001191276
PARG2084	1.274596502	1.437057919	0.000174616	0.001191276
PARG1985	-1.106837658	2.986615857	0.000176142	0.001201162
PARG1963	2.162955741	1.009436009	0.000177431	0.001209427
PARG2798	1.026193565	5.874133079	0.000178065	0.001213222
PARG1439	-1.027532359	4.950151366	0.000178196	0.001213585
PARG2436	1.00372968	8.144093808	0.000178711	0.00121594
PARG1050	1.697291168	3.532198821	0.000178725	0.00121594
PARG1973	-1.046824818	4.306602896	0.000178829	0.00121594
PARG2940	1.046692322	4.903023825	0.000178851	0.00121594
PARG2498	-1.266456901	4.550307557	0.000179013	0.001216515
PARG0459	1.029151918	5.634842752	0.000180829	0.00122832
PARG2434	1.02252316	4.405325532	0.000184238	0.001250936
PARG1821	-1.002140543	7.169956463	0.000184657	0.001253241
PARG1303	1.843697085	0.871214045	0.000185422	0.001257889
PARG2713	1.57219354	5.117970021	0.000187715	0.001272512
PARG0406	1.033699935	4.349089277	0.00018774	0.001272512
PARG2841	1.197338309	4.795608201	0.00018948	0.001283553
PARG0375	-1.013436565	5.742915621	0.000189532	0.001283553
PARG2467	1.017979525	5.973577331	0.000192698	0.001304431
PARG0859	-1.047883789	5.449002369	0.000193149	0.001306358
PARG1991	-1.007131486	6.662426243	0.000194	0.00131155
PARG2455	-1.008000794	6.508708146	0.000195238	0.001319351
PARG0278	1.021193688	5.11381024	0.00019688	0.001329871
PARG2097	-1.028991033	5.924877875	0.000197058	0.001330498
PARG2124	1.257860006	2.06592652	0.000197495	0.001332882
PARG1067	-1.001274241	6.814671367	0.000200132	0.001350096
PARG0064	1.462002139	1.23128833	0.000200388	0.001351242
PARG2528	-1.088025354	3.569029967	0.000201363	0.00135665
PARG2664	1.669033518	3.938474266	0.000201673	0.001358158
PARG1453	1.25134979	2.861584008	0.000202545	0.001363446
PARG2005	-1.002796376	6.173203268	0.000204573	0.001376384
PARG1943	1.389798935	0.883382462	0.000204643	0.001376384
PARG1712	-3.773970032	-0.501776072	0.000205502	0.001380387
PARG0025	-3.760461159	-0.539114635	0.000205502	0.001380387
PARG0408	3.706322022	-0.385615368	0.000205502	0.001380387
PARG2756	1.186493627	2.784451047	0.000206085	0.001383369
PARG1816	-1.124174269	2.808736602	0.000206169	0.001383369
PARG0170	1.226226166	1.97978338	0.00020621	0.001383369
PARG1653	-1.022532164	5.099536989	0.000206398	0.001384039
PARG2490	1.143634482	3.18381484	0.000207254	0.001389185
PARG0519	-1.332815837	0.876006325	0.000207412	0.001389652
PARG1994	-1.007730797	6.237868296	0.000209278	0.001400956
PARG2349	-1.324332661	0.930960813	0.000209861	0.001404263

PARG0268	-2.907469903	1.32014359	0.000211598	0.001413471
PARG0097	2.830381835	-0.505517128	0.000211598	0.001413471
PARG0682	2.817687425	2.645402868	0.000211598	0.001413471
PARG2504	1.016498496	6.408006482	0.000213176	0.001423409
PARG2537	1.464418869	0.725627532	0.00021374	0.00142657
PARG1653	-1.003980307	6.725653605	0.000215386	0.001436942
PARG0898	-1.286859522	2.690197135	0.000217085	0.001447667
PARG2280	-1.020160551	4.373507784	0.000217524	0.001449977
PARG1207	1.000760897	5.557585151	0.000217767	0.001450362
PARG0151	1.0160638	4.251837636	0.000218214	0.001452725
PARG1233	-1.12359654	2.200012981	0.000218315	0.001452781
PARG1731	-1.14796957	3.10697111	0.000222885	0.001478511
PARG0981	-1.042699452	4.289313694	0.000222914	0.001478511
PARG2089	-3.220947265	1.653810249	0.000222945	0.001478511
PARG1944	3.147490724	1.180169778	0.000222945	0.001478511
PARG1994	1.021027982	5.462216445	0.000223043	0.001478511
PARG0005	1.157941947	2.605128315	0.000223124	0.001478511
PARG1315	-1.062381274	4.725977078	0.000223869	0.001482822
PARG1538	1.174082819	4.001832764	0.000224594	0.001486997
PARG0618	1.19586432	2.365119454	0.000225034	0.001489285
PARG0381	1.902673568	0.50886505	0.000229863	0.001519964
PARG2016	1.880022877	-0.476368561	0.000229863	0.001519964
PARG0759	1.072070089	4.398534012	0.000230007	0.001520271
PARG0199	-1.563520721	2.377874968	0.000230353	0.001521277
PARG0606	-1.084729752	3.0369428	0.000231883	0.00153074
PARG2116	1.008862066	5.727115467	0.000233114	0.001537571
PARG0794	-1.130881761	2.526254949	0.000236169	0.001555763
PARG0074	1.127246849	2.299568182	0.000237148	0.001561559
PARG0231	-1.069827549	3.232099298	0.000237837	0.001563849
PARG2942	1.711247788	4.109783592	0.000237895	0.001563849
PARG0904	-1.686718964	-0.623300038	0.000237895	0.001563849
PARG2993	-1.616111889	1.439089255	0.000237895	0.001563849
PARG1789	-2.181852706	-0.525242825	0.000238613	0.001567915
PARG1791	5.305611777	-0.782803224	0.000241628	0.001583089
PARG1368	-5.28442886	3.619196697	0.000241628	0.001583089
PARG2702	5.278150575	0.932235585	0.000241628	0.001583089
PARG1197	5.245271138	1.73577138	0.000241628	0.001583089
PARG0578	5.243107413	2.703955267	0.000241628	0.001583089
PARG1686	5.241638941	-0.652091446	0.000241628	0.001583089
PARG1811	-1.000190356	5.511361803	0.000242555	0.001587173
PARG1609	-1.025148818	4.827843021	0.000243586	0.001593257
PARG1872	-1.286773667	5.382941203	0.000245094	0.001599786
PARG1368	1.079284542	3.787815712	0.000248252	0.001619726
PARG1285	1.016607088	6.346687878	0.000249592	0.001627793
PARG2218	1.017899876	4.706089585	0.000256386	0.001668632
PARG1533	1.098969198	2.92435738	0.000257065	0.001672363

PARG1855	1.09406157	4.370403014	0.000258255	0.001679407
PARG1155	1.017072527	3.663098965	0.000260203	0.001689971
PARG1671	1.035574351	3.881373282	0.00026495	0.00171938
PARG2327	1.040073571	3.834922482	0.000267954	0.001737441
PARG1710	1.05967334	4.409081972	0.000269749	0.001748354
PARG2093	-1.191555433	0.694412401	0.000271511	0.001758052
PARG2695	-1.335104823	1.045485586	0.000271581	0.001758052
PARG0112	-1.584531117	0.797364167	0.000272597	0.001763906
PARG1417	1.122384043	2.269482907	0.000274746	0.001776342
PARG1354	-1.197121846	0.938107115	0.000274999	0.001777246
PARG1547	1.095974534	3.632205721	0.000276012	0.001781592
PARG0391	1.007881924	4.487640623	0.000276185	0.001781981
PARG1108	-1.077556059	4.235032047	0.000276512	0.001783355
PARG0737	1.298536151	2.197892928	0.000277998	0.001791467
PARG2369	-1.111813299	2.618791647	0.000279321	0.001798036
PARG0442	-1.031078047	5.669785086	0.000279361	0.001798036
PARG2583	-1.040850287	3.607735992	0.000281066	0.001807526
PARG0642	-1.732419699	-0.471039751	0.0002828	0.001817191
PARG0229	1.69331275	1.301484261	0.0002828	0.001817191
PARG1843	-1.007812474	5.135564295	0.000284098	0.001824335
PARG0091	-1.149561895	1.460193436	0.000284144	0.001824335
PARG0192	1.017002515	4.146476379	0.000286062	0.001835144
PARG0462	2.278496279	-0.261306699	0.000287883	0.001845319
PARG1781	2.305391905	3.275094782	0.0002942	0.001884408
PARG2855	-1.055150201	6.617118557	0.000297791	0.001905719
PARG2288	-1.002145973	4.62346786	0.000299889	0.001918362
PARG2142	-1.20208651	3.165863614	0.000306022	0.001955997
PARG0367	2.154448534	2.101069582	0.000306231	0.001956536
PARG2049	1.127801806	2.798011256	0.000306654	0.001958442
PARG0451	-1.029926562	3.687795868	0.000308281	0.001968034
PARG1509	1.405705321	0.43252355	0.000310646	0.001981518
PARG0641	-1.370088799	1.203073664	0.000310646	0.001981518
PARG2475	-1.616670141	0.878295061	0.000311218	0.001984365
PARG0963	1.368404425	0.51867483	0.000313242	0.001996455
PARG2439	-1.332542571	0.939650101	0.000315916	0.00201268
PARG1866	-1.336033283	4.329006805	0.000316532	0.00201579
PARG0809	-1.117773026	1.314742134	0.00031719	0.002019159
PARG2452	1.036699171	3.488289197	0.000317839	0.00202247
PARG1324	-1.123641821	1.898606107	0.000325098	0.002066149
PARG2236	1.177435493	2.369098564	0.000329722	0.002092994
PARG1313	-1.046971078	2.520930887	0.000330318	0.00209593
PARG0633	-1.179194717	2.369874834	0.000334069	0.002118874
PARG1856	-1.060475217	3.863478539	0.000334827	0.002122822
PARG1244	-1.091332207	5.48024165	0.000337027	0.002135913
PARG2286	1.705904461	0.445338492	0.000338621	0.00214487
PARG0860	-1.057126252	4.077013533	0.000339377	0.0021482

PARG0876	1.181055918	4.675093367	0.00034219	0.002164261
PARG2924	-2.167707571	0.94553771	0.000342601	0.002165988
PARG2914	-1.213842701	1.969539433	0.000348771	0.002204107
PARG0143	-3.751206	-0.391311101	0.000349856	0.002207413
PARG0042	-3.668237448	1.33717631	0.000349856	0.002207413
PARG1647	3.659299467	-0.74655814	0.000349856	0.002207413
PARG1854	3.628772835	1.015939317	0.000349856	0.002207413
PARG2130	1.535074203	1.603832939	0.000350369	0.002208874
PARG2965	1.511055005	1.155512722	0.000350369	0.002208874
PARG1103	-2.395072743	-0.28369278	0.000361579	0.00227589
PARG2852	3.068619097	1.489079424	0.000362814	0.002281835
PARG0459	3.06724062	1.854092794	0.000362814	0.002281835
PARG1874	1.160805089	2.153319309	0.000365851	0.002300014
PARG1860	-1.284976995	1.048989746	0.00036656	0.002303545
PARG0317	1.676833628	2.790522913	0.000375412	0.00235446
PARG0861	1.088677959	5.323173632	0.0003838	0.002401315
PARG0505	1.655486702	1.759261562	0.000384989	0.002407793
PARG0850	1.838588436	-0.059051511	0.000386604	0.002416926
PARG1100	-2.102122282	0.301724372	0.000386835	0.002417411
PARG1864	1.235119879	2.705874127	0.000388873	0.002429181
PARG1963	-1.055741884	3.78599935	0.000399518	0.002489733
PARG0903	1.128955216	2.073700324	0.000402604	0.002507967
PARG0365	-1.008134764	2.842824179	0.000409001	0.002542773
PARG0922	1.476415051	0.570975741	0.000412709	0.002564808
PARG1333	-1.010187758	2.733872648	0.000414502	0.002574255
PARG2681	1.109487848	3.594876676	0.000414777	0.002574601
PARG0690	-1.097210575	2.456467528	0.000416751	0.002585833
PARG1182	1.446039888	0.166433465	0.000421495	0.002613204
PARG1971	1.038973976	2.048681737	0.00042234	0.002617407
PARG2661	-1.449678006	0.117864998	0.000424893	0.002630113
PARG0874	-1.010894408	4.716519613	0.000425902	0.002634281
PARG0022	2.107184658	1.170856566	0.000426602	0.002637572
PARG1425	-2.228724032	2.636783595	0.000430007	0.002654446
PARG0081	2.199369241	-0.069929042	0.000430007	0.002654446
PARG1099	2.165455674	1.216481275	0.000430007	0.002654446
PARG2294	-1.328586811	1.863491224	0.000432015	0.00266474
PARG2028	1.328763151	0.577241065	0.000433001	0.002668728
PARG0593	1.318892391	0.570843137	0.000433001	0.002668728
PARG1378	-1.025494336	4.254936727	0.000439506	0.002705631
PARG2164	2.494158547	0.024644534	0.000439894	0.002706962
PARG2662	-5.168419811	0.033144792	0.000440227	0.002707947
PARG2792	1.191553107	2.114592277	0.000442566	0.002721212
PARG1976	-1.726666029	2.22660345	0.000443076	0.002722271
PARG0532	-1.01084696	3.617503984	0.000446472	0.002742062
PARG2711	1.150350627	1.442778383	0.00046108	0.002825153
PARG1148	2.051178043	3.077912066	0.000461886	0.002826779

PARG0067	1.909343028	2.391064797	0.000461886	0.002826779
PARG0026	1.898862221	2.572072674	0.000461886	0.002826779
PARG0437	1.183095302	2.179309319	0.000470182	0.002874188
PARG2058	1.293250837	1.417627362	0.000478214	0.002917609
PARG2485	1.26616482	2.050068612	0.000481054	0.002932657
PARG0352	1.056166147	4.118111659	0.000482424	0.002938724
PARG1810	1.265305461	1.975819113	0.000486717	0.002962579
PARG3039	1.250359468	1.599244443	0.000486717	0.002962579
PARG0298	-1.899180203	2.284246982	0.000492851	0.00299759
PARG0663	-1.11634327	3.038391352	0.000494152	0.003004337
PARG1183	1.054136458	3.37071284	0.000494775	0.00300669
PARG1068	-1.000875416	6.684421644	0.000505906	0.003065115
PARG1674	-1.209518505	1.78291099	0.000507719	0.003074913
PARG2424	1.388327891	1.517503201	0.00052195	0.003155014
PARG0789	2.691376235	1.933686945	0.000522899	0.003158315
PARG2028	-1.035955401	3.140162751	0.000526793	0.00317695
PARG2850	1.024832272	2.737091193	0.000528004	0.003183031
PARG2068	-1.07770131	2.308344217	0.000529917	0.003192106
PARG1282	1.274378965	1.915144965	0.000535161	0.003221223
PARG2065	-1.025060558	2.44646061	0.000535557	0.003222373
PARG0927	-1.253992206	2.81898495	0.000539296	0.003241145
PARG1483	1.071498524	2.967294755	0.00054802	0.00328728
PARG2322	1.123548665	1.596797356	0.000556605	0.003333685
PARG2564	-1.123495188	3.041909361	0.000560245	0.003352924
PARG0936	1.246487492	2.468644624	0.00056065	0.003354072
PARG0537	1.056205914	5.054807554	0.000562567	0.003362979
PARG1222	1.318176403	1.728580816	0.00056503	0.003376414
PARG1212	1.261733349	1.081366723	0.000569434	0.003400145
PARG0651	1.147490378	3.565424823	0.000574063	0.003423878
PARG1313	-1.02493329	2.804450784	0.000581552	0.003461968
PARG2465	1.0313471	5.320749816	0.000584249	0.003475387
PARG0471	1.330140737	2.187591572	0.000587143	0.003487312
PARG1179	2.99709794	3.845794816	0.00059137	0.00350844
PARG2742	2.988142399	4.012790367	0.00059137	0.00350844
PARG0112	1.648088026	0.044649505	0.000593439	0.003516726
PARG1950	1.635254334	1.079991751	0.000593439	0.003516726
PARG0792	1.139040297	1.400647699	0.000619379	0.003655268
PARG2924	-1.31844311	3.30026678	0.000620271	0.003659156
PARG1307	1.601606985	2.334195848	0.000621921	0.003667514
PARG0764	-1.574171527	2.245588243	0.000627766	0.003699202
PARG2211	-1.371381643	0.680388312	0.00063003	0.003711149
PARG2061	1.130454744	1.096962909	0.000633285	0.003727524
PARG1800	1.026035497	5.035863553	0.000641849	0.003773689
PARG1433	1.143751333	2.683902106	0.000645747	0.003793763
PARG0005	1.094368061	1.125830659	0.000659018	0.003870283
PARG0007	1.096475059	3.223924908	0.000667482	0.003915597

PARG1579	-1.084718379	3.336255862	0.000667482	0.003915597
PARG0241	-1.049713669	3.666302625	0.000670194	0.003930037
PARG0439	-1.266539801	1.528123828	0.000671612	0.003936547
PARG0603	2.180708889	-0.08404856	0.000672342	0.003937195
PARG0440	-1.140678049	2.455270734	0.000679638	0.003977985
PARG0739	1.690443906	0.843133262	0.000698378	0.004075517
PARG1513	-1.074814398	5.751032709	0.000704977	0.004110971
PARG0352	-1.0003511	3.492412601	0.000705871	0.004114653
PARG1376	1.034539702	2.699597702	0.000715783	0.004167788
PARG2423	-1.234494919	0.987220577	0.000717967	0.004178957
PARG2807	-1.739847503	-0.102994452	0.000722297	0.004201044
PARG2599	-1.735689377	1.041156446	0.000722297	0.004201044
PARG1791	1.333440272	0.261232107	0.000726349	0.004223046
PARG1611	1.072058799	2.042453202	0.000732414	0.004250439
PARG2393	-1.168319688	4.492843366	0.000743389	0.004302997
PARG2401	-1.263297892	1.029302391	0.000770381	0.004444483
PARG2636	-1.36122997	3.824815191	0.000770938	0.004444519
PARG2265	-2.021019551	0.508989939	0.00077155	0.004446327
PARG0059	1.525196908	1.552253651	0.000778702	0.004480962
PARG2400	1.018844611	3.001883881	0.000780026	0.004485294
PARG0616	1.056511481	3.381113962	0.000786502	0.004516086
PARG0740	-1.531667555	1.384426367	0.000792361	0.004542907
PARG1982	1.427179127	0.484576407	0.000792852	0.004543304
PARG2766	-1.4746073	-0.16803836	0.000793298	0.004543304
PARG0850	-1.434798852	3.254115997	0.000793298	0.004543304
PARG0364	-1.066861817	3.031882342	0.000795595	0.004551478
PARG0745	2.263354842	3.358348611	0.000804721	0.004593643
PARG1520	-5.074843496	2.881780891	0.000807082	0.004605446
PARG2045	-1.010611772	3.080337418	0.000811957	0.004628596
PARG2277	-1.000630855	6.071711867	0.000812024	0.004628596
PARG2777	1.136970637	2.555646976	0.000821529	0.004679377
PARG0203	2.62046882	4.171261163	0.000822852	0.004684985
PARG1747	1.23141231	1.968319803	0.0008327	0.004736131
PARG2698	1.071521389	3.190179706	0.000840706	0.004776476
PARG0688	1.130618546	2.242363068	0.000841716	0.004780486
PARG1767	-1.040401382	1.766914086	0.000849141	0.004815687
PARG0590	-1.221891443	1.676425712	0.000855573	0.004848661
PARG0867	1.211804462	7.872191368	0.000874621	0.004949462
PARG1420	-1.089299622	2.325390117	0.000880499	0.004975551
PARG2155	-1.052093591	1.589189431	0.00088885	0.005016093
PARG1653	-1.006424788	3.116007178	0.000889272	0.005016093
PARG1654	-1.006424788	3.116007178	0.000889272	0.005016093
PARG2009	1.110555352	2.559588921	0.000891118	0.005021408
PARG2734	-1.08944503	2.236157978	0.000891118	0.005021408
PARG0651	-1.038148477	1.854419408	0.000892153	0.00502512
PARG2327	1.298806689	3.512303021	0.000893103	0.005026864

PARG2789	-1.803460525	-0.272639888	0.000908636	0.005106442
PARG0688	1.156811978	2.346369466	0.000909518	0.005106442
PARG1733	1.311760934	-0.219095754	0.00091463	0.005129638
PARG2112	1.031038236	2.990246519	0.000917235	0.005140579
PARG2395	1.370645841	6.167025266	0.000925251	0.005176679
PARG2426	1.3859805	2.468739894	0.000935693	0.005219799
PARG2732	-1.031844675	3.176362418	0.000937041	0.005225458
PARG0600	1.704834416	6.004376269	0.00093771	0.005225752
PARG2648	-1.067800321	3.509930038	0.000939686	0.005232774
PARG0751	-1.362653803	2.742569177	0.000955707	0.005314447
PARG2147	-1.715297021	-0.110222411	0.000960216	0.00533574
PARG0509	-2.989077572	2.388416522	0.000965181	0.005359536
PARG1895	-1.444758303	0.785375667	0.000973848	0.005400021
PARG2945	-1.262389973	4.819510286	0.000974265	0.005400425
PARG1848	1.210136545	7.189478947	0.000976986	0.005410298
PARG2802	1.742722043	3.327969852	0.00097708	0.005410298
PARG1232	1.594175097	0.25536639	0.00097708	0.005410298
PARG0779	-1.087534003	2.457281602	0.000987433	0.005457999
PARG1533	-1.480429931	0.449420331	0.00098897	0.005462424
PARG1809	-1.676825037	2.535360113	0.000989129	0.005462424
PARG1343	1.526879659	0.566456672	0.000994924	0.005488953
PARG1065	1.662383268	0.153284647	0.00099709	0.00549397
PARG0779	1.564588414	0.572567065	0.001001596	0.005516862
PARG0292	1.205703068	2.655488517	0.001007711	0.005548599
PARG0312	-2.067289147	-0.259855771	0.001012039	0.005567023
PARG0030	1.252701339	1.163443708	0.001019758	0.005607061
PARG0239	-3.516499715	-0.447798367	0.001023013	0.005621022
PARG1061	3.44956594	1.147058165	0.001023013	0.005621022
PARG0222	2.395565112	0.988965224	0.001027615	0.005640382
PARG1181	2.383250986	2.002974281	0.001027615	0.005640382
PARG2390	2.376237135	-0.438606809	0.001027615	0.005640382
PARG1087	-1.089611283	1.842196321	0.001029019	0.00564612
PARG2693	1.189710594	0.336125711	0.001040466	0.005700957
PARG1810	1.059831369	2.007231567	0.001043221	0.005714056
PARG2423	1.09972679	1.927980606	0.001044582	0.005717519
PARG0039	-1.071233765	2.20525518	0.001083294	0.005908808
PARG1202	-1.239450591	1.588000305	0.001086025	0.005919588
PARG2201	-1.232032978	1.273853939	0.001086025	0.005919588
PARG0650	2.127048497	-0.447743063	0.0010886	0.00592745
PARG2575	-1.970260901	0.182834791	0.0010886	0.00592745
PARG1941	-1.056887122	3.555328166	0.001091125	0.005939139
PARG0642	1.082593786	2.429596612	0.001098537	0.005973271
PARG0439	-1.015823928	1.818822367	0.001109411	0.006026135
PARG1334	1.239093062	1.499908998	0.001152712	0.006229011
PARG2649	1.050771577	2.333375942	0.001191386	0.006424724
PARG1808	2.24385791	-0.393659514	0.001199951	0.006466471

PARG0181	-1.415117195	3.290295484	0.001201875	0.006474618
PARG1126	-1.054612958	3.495126704	0.001206938	0.006495205
PARG2436	1.077598237	3.092637013	0.001222797	0.006569293
PARG0434	-1.145155715	2.495194694	0.001252248	0.006711434
PARG2683	-1.143511477	1.092576668	0.001252248	0.006711434
PARG2125	1.186868634	0.652078982	0.00126539	0.006772619
PARG2108	1.126647576	2.123874595	0.001275018	0.0068195
PARG1373	-1.088025401	1.814279648	0.001279437	0.006831496
PARG1912	1.558429561	0.292129819	0.001290986	0.006890819
PARG0375	2.604615946	0.92407185	0.00129528	0.006906693
PARG1271	2.555732263	0.737493029	0.00129528	0.006906693
PARG0885	1.100681997	2.440575316	0.001297257	0.006914885
PARG1076	-1.221338259	1.203088716	0.001316227	0.007004113
PARG1927	1.019107586	4.460930975	0.00132184	0.007031596
PARG2058	1.358540781	0.975315673	0.001325197	0.007047068
PARG1939	2.067716672	4.149109822	0.001339905	0.007120456
PARG1276	1.195858944	3.02508448	0.001355076	0.007193772
PARG0792	1.039793003	1.658609489	0.001363721	0.007222568
PARG2740	-1.021925746	3.198468512	0.001363721	0.007222568
PARG1983	1.090430166	0.681639845	0.001391239	0.007363341
PARG2475	-1.042936131	2.94963899	0.001424148	0.007514714
PARG1198	1.048376516	2.224719139	0.001433107	0.007556909
PARG1457	1.190966869	5.781011327	0.001434957	0.007559043
PARG1576	1.017341003	2.348010066	0.00146408	0.007694384
PARG0013	1.172896527	1.940688156	0.001465282	0.007698125
PARG1421	1.079388391	2.593370902	0.001466846	0.007703763
PARG0854	1.044211377	1.923184846	0.001483519	0.007775718
PARG1926	-4.951111105	1.394276478	0.001489124	0.00778696
PARG1753	4.904864556	1.657955278	0.001489124	0.00778696
PARG3001	4.904767705	1.405761931	0.001489124	0.00778696
PARG0564	4.903844083	1.037587275	0.001489124	0.00778696
PARG0903	4.903445569	-0.582243797	0.001489124	0.00778696
PARG2296	4.902314289	2.087895129	0.001489124	0.00778696
PARG0498	1.292663922	3.233956679	0.001491767	0.007798121
PARG0222	1.223610568	4.300193414	0.001506452	0.007861798
PARG1423	-1.03610903	3.066980098	0.001508131	0.007867942
PARG2924	2.040542721	2.395422464	0.001534559	0.00797666
PARG2752	2.039297913	0.319548291	0.001534559	0.00797666
PARG0402	-1.950646991	1.743665262	0.001534559	0.00797666
PARG1855	1.888618082	1.916683821	0.001534559	0.00797666
PARG2630	-1.105887134	4.686195788	0.001537785	0.007990782
PARG1410	1.015204439	3.322389991	0.001557831	0.008081571
PARG2725	-1.295750232	0.693403257	0.001558693	0.008083372
PARG1931	1.184743072	2.841491321	0.00156387	0.008107542
PARG2726	2.848370644	0.184486645	0.001576871	0.008166848
PARG1292	2.829392321	-0.480441057	0.001576871	0.008166848

PARG1741	-1.009066658	1.935188309	0.001577799	0.008168961
PARG0497	1.080550049	1.902966256	0.001593935	0.008241628
PARG2415	1.949235638	1.327295515	0.001596885	0.008251731
PARG2492	-1.755095689	-0.278365868	0.001640825	0.00845068
PARG1494	1.274974816	0.750874451	0.001667386	0.008573412
PARG2515	-1.553490049	2.94063225	0.001670029	0.008581382
PARG2995	1.524919443	0.63510468	0.001670029	0.008581382
PARG1215	1.637250391	2.866468169	0.001683019	0.008637151
PARG2437	1.626548406	0.066971404	0.001683019	0.008637151
PARG1613	-1.710702442	0.081482569	0.001686068	0.008649642
PARG0190	-1.329038196	0.007849442	0.001705793	0.008736555
PARG0702	3.362859134	2.928478154	0.001756313	0.00895731
PARG0335	-3.332850049	-0.645962468	0.001756313	0.00895731
PARG0138	-1.369893222	1.075498092	0.001785625	0.009094987
PARG0803	-2.165805358	1.217671179	0.00178809	0.009104586
PARG0232	1.184432195	3.736401821	0.001801995	0.009160532
PARG1677	1.432787323	6.62937097	0.001826885	0.009269048
PARG1835	1.069844544	3.280743161	0.001838694	0.009322499
PARG1121	1.396402784	1.87453798	0.001868871	0.009454573
PARG2505	-1.324290209	1.123556428	0.001868871	0.009454573
PARG1739	1.082287364	4.696038336	0.001874552	0.009477202
PARG0579	1.081389424	1.238433486	0.001879977	0.009498512
PARG2538	-1.003367338	2.629028491	0.00189045	0.009548356
PARG1251	1.123116327	0.475646441	0.001910624	0.00963475
PARG0812	1.37041542	-0.177775754	0.001911403	0.009635583
PARG0060	2.29797458	1.951359713	0.001954202	0.009835547
PARG1212	2.015299476	1.252930894	0.001954202	0.009835547
PARG0263	1.047558815	1.525137862	0.001958447	0.009849187
PARG0247	1.466707577	3.031582355	0.001997065	0.010012754
PARG2128	1.1746481	4.965576133	0.002026849	0.010145878
PARG2629	-2.602433101	2.314869365	0.002038892	0.010189585
PARG1474	2.492128534	4.493037737	0.002038892	0.010189585
PARG1964	1.43046348	0.216104628	0.002039475	0.010189585
PARG1720	-1.403878724	0.171081886	0.002039475	0.010189585
PARG0262	-1.117884698	0.891955962	0.002043336	0.010205626
PARG1367	1.197011504	2.579204668	0.002050484	0.010234819
PARG0912	1.907094172	0.521752746	0.0020759	0.010348518
PARG2089	1.459722907	1.092345769	0.002080945	0.010364215
PARG2469	1.095501387	1.125613372	0.002150646	0.010673676
PARG1782	-1.88262958	4.13874477	0.002160809	0.01070052
PARG2598	-1.854854986	-0.32073533	0.002160809	0.01070052
PARG2729	1.844960573	0.985776595	0.002160809	0.01070052
PARG1261	1.020459241	3.462670007	0.00217203	0.010752612
PARG1309	1.022110027	2.592757908	0.002195056	0.010859749
PARG2117	1.162002991	4.312290126	0.002208144	0.010903883
PARG2012	1.050752516	3.948799177	0.002211124	0.010911736

PARG2727	1.531912111	0.245400241	0.002221118	0.010954165
PARG1353	-1.09001288	1.496182209	0.002245922	0.011062592
PARG0656	-1.714064648	1.773207742	0.002259548	0.011119243
PARG1583	-1.703073006	-0.070063638	0.002259548	0.011119243
PARG2550	-1.094680029	0.229768236	0.002267697	0.011150464
PARG0796	1.219681584	0.086395823	0.002301161	0.011302761
PARG1106	-1.358397125	1.057551608	0.002312519	0.011347896
PARG0228	-1.146363587	0.018285105	0.002331667	0.011431139
PARG0299	-1.063110855	0.567008474	0.002359151	0.011551453
PARG1952	1.349280776	0.28736159	0.002377084	0.011632004
PARG2251	2.258309749	1.35190091	0.002397611	0.01171419
PARG0917	2.247281953	-0.235794316	0.002397611	0.01171419
PARG2032	-2.225767726	1.209094375	0.002397611	0.01171419
PARG2947	-1.026103495	1.061337412	0.002506456	0.012188159
PARG1987	-1.172222287	1.315607764	0.002510026	0.012198882
PARG1062	1.39305636	-0.008760724	0.002510955	0.01219962
PARG1068	-2.876683981	2.037453098	0.002577856	0.012489872
PARG1824	2.774320714	-0.321427016	0.002577856	0.012489872
PARG1847	2.745701807	-0.369576237	0.002577856	0.012489872
PARG2957	-1.006461269	2.116449265	0.002600702	0.012587928
PARG0115	-1.164832666	2.429886988	0.002701203	0.013003214
PARG1652	1.500346193	0.783977124	0.002717973	0.013071918
PARG0915	-4.821681674	0.398518929	0.002765516	0.013264
PARG3000	-4.794319867	0.457140706	0.002765516	0.013264
PARG1951	4.772693938	2.674567484	0.002765516	0.013264
PARG0126	-1.484654892	0.668496029	0.002786049	0.013346169
PARG1391	1.202974574	3.589931347	0.002789655	0.013359368
PARG2707	1.084378451	3.345961817	0.002847718	0.013600086
PARG2512	-1.516492894	1.075556179	0.002851909	0.013615956
PARG1220	1.861512036	5.91854037	0.002967707	0.014121573
PARG1636	1.190659456	7.503314518	0.002981688	0.014179504
PARG2806	1.019497821	0.833163766	0.003000628	0.01426093
PARG1113	1.691177272	2.258196181	0.003018025	0.014330597
PARG0247	1.668762282	2.31574951	0.003018025	0.014330597
PARG0204	-1.068334972	3.047941756	0.003020488	0.01433217
PARG2941	-3.32118516	1.740831047	0.003022008	0.01433217
PARG0664	3.251507981	1.868870386	0.003022008	0.01433217
PARG1171	3.249811668	0.817183677	0.003022008	0.01433217
PARG1993	1.152047436	-0.072866412	0.00303571	0.014384117
PARG0103	-1.775769451	0.855844975	0.003070643	0.014531611
PARG1171	1.820891702	2.876214074	0.003073318	0.014531611
PARG2404	-1.72204502	-0.367462267	0.003073318	0.014531611
PARG1698	1.137944612	0.921997943	0.003179314	0.015001165
PARG0761	-1.100687546	0.644130272	0.003211911	0.015132228
PARG2669	-1.110967504	5.787191315	0.003243984	0.015265007
PARG1386	1.270904413	0.876820839	0.003281701	0.015414762

PARG2442	1.178994814	2.705150502	0.003292436	0.015460565
PARG1031	-1.146299832	1.28797129	0.003412336	0.015999659
PARG1111	-1.130152599	4.456940963	0.003477276	0.016265278
PARG1812	1.285764221	2.109976457	0.003531164	0.016502592
PARG2741	-1.461227248	3.090799168	0.003592109	0.016757484
PARG0556	1.013620194	3.121903717	0.003638324	0.016947894
PARG0130	1.141352202	0.488645409	0.003642912	0.016958606
PARG1353	1.125606597	0.278102489	0.003642912	0.016958606
PARG1884	1.034460882	0.847943429	0.003769151	0.017479506
PARG2375	-1.370479123	0.656058162	0.003807915	0.017633229
PARG2457	-1.187209562	2.015136156	0.00381822	0.017660112
PARG2751	-1.171144378	1.731935204	0.00381822	0.017660112
PARG2094	1.240109099	0.172182241	0.003855555	0.01782229
PARG1075	1.091539474	3.45223366	0.003925165	0.018085482
PARG2418	1.336794943	1.470871307	0.00395706	0.018216401
PARG0157	1.156239365	0.236131697	0.003971006	0.018272787
PARG2025	1.2744022	1.224511583	0.004012633	0.018428997
PARG2528	-1.02188876	3.66417025	0.004018878	0.01845228
PARG2069	1.250408465	3.52855533	0.004133733	0.018902224
PARG2245	-1.175760247	1.874235576	0.004133733	0.018902224
PARG2824	-2.004248338	-0.26713641	0.004140647	0.018927398
PARG2446	1.736448456	0.185705585	0.004196691	0.019139935
PARG1396	1.116430444	-0.074727354	0.004198696	0.019142035
PARG0195	2.659666082	0.990733872	0.004215024	0.01920123
PARG0608	1.110770777	3.084383374	0.004228033	0.019249323
PARG2186	1.081064546	1.278824631	0.004228033	0.019249323
PARG2046	1.803914316	3.820131848	0.004235879	0.019262696
PARG0731	1.799675676	0.783747861	0.004235879	0.019262696
PARG0041	1.815815169	2.720148587	0.004248307	0.019308025
PARG1625	-1.781494855	1.984482322	0.004265862	0.019370987
PARG0797	-1.761105175	1.390864777	0.004265862	0.019370987
PARG1262	-1.161634383	2.495874531	0.004307214	0.019519238
PARG1277	1.254000981	2.084055667	0.004356927	0.019707595
PARG2059	1.082524221	2.266601554	0.004370467	0.019748877
PARG1720	1.186476795	-0.863053512	0.004372193	0.019750988
PARG2030	1.151021318	-0.785004186	0.004392045	0.019834959
PARG0605	-1.069176521	2.286627561	0.004537482	0.020417194
PARG0462	1.021035269	2.034210528	0.004547215	0.020442074
PARG0721	1.169708657	5.443566521	0.004576802	0.020562863
PARG0703	1.012356305	1.827347282	0.004608072	0.02066786
PARG2247	1.395670758	0.226367805	0.004625119	0.020738394
PARG1844	1.183117702	1.314239554	0.004901458	0.021857507
PARG1254	-1.033229966	2.911894606	0.005034929	0.022352463
PARG1604	2.33507815	2.698666202	0.005043113	0.022382467
PARG1922	1.263418321	6.132473518	0.00512764	0.02273191
PARG2756	-4.652608736	-0.557633697	0.005170313	0.022875865

PARG2390	4.65125785	0.854472577	0.005170313	0.022875865
PARG1484	4.628762429	0.903458617	0.005170313	0.022875865
PARG0328	4.627185034	3.071782156	0.005170313	0.022875865
PARG1686	4.626450824	-0.766644075	0.005170313	0.022875865
PARG0873	4.598590635	-0.066185004	0.005170313	0.022875865
PARG0843	-1.21633606	2.735144522	0.005194266	0.022968897
PARG2377	-3.212545758	-0.862790987	0.005209351	0.023016152
PARG3038	3.13826577	1.565286054	0.005209351	0.023016152
PARG0146	-3.09898375	1.590646509	0.005209351	0.023016152
PARG2282	1.117093404	0.78422229	0.005237785	0.023115758
PARG1073	1.619603181	2.532217771	0.005370633	0.023607278
PARG2058	1.484979181	3.46212701	0.005370633	0.023607278
PARG1116	-1.069299237	1.478504187	0.005390216	0.023668702
PARG2916	1.377865066	2.415807358	0.005424611	0.023789821
PARG0255	-1.138246515	1.87348081	0.005437803	0.0238177
PARG2761	-1.585308713	2.252747543	0.005551881	0.02425645
PARG0658	1.553002317	0.570921605	0.005551881	0.02425645
PARG0092	2.108528786	1.651557613	0.005571048	0.024313123
PARG1041	1.299072817	0.151529648	0.005629371	0.02451314
PARG0311	-1.276120747	0.633819097	0.005629371	0.02451314
PARG2138	1.268923433	1.285655336	0.005629371	0.02451314
PARG2856	-1.262381227	-0.441029984	0.005629371	0.02451314
PARG0858	1.188353741	0.535454447	0.005689644	0.02474814
PARG0209	1.562772031	5.588016367	0.005720387	0.024851202
PARG1581	1.960817153	3.630568447	0.005872939	0.025432662
PARG1818	1.179014516	-0.642360663	0.005963274	0.025788286
PARG2004	1.017416849	0.693081701	0.005969808	0.02580943
PARG1145	-1.747522066	3.455246427	0.005977556	0.025814492
PARG1189	-1.727492917	0.27004038	0.005977556	0.025814492
PARG0084	1.662316458	0.619291951	0.005977556	0.025814492
PARG0726	2.07688534	3.345111705	0.006011354	0.025910555
PARG0750	1.844116224	3.00429027	0.006011354	0.025910555
PARG2157	1.844116224	3.65352095	0.006011354	0.025910555
PARG2842	1.329519942	2.007920794	0.006206097	0.026647514
PARG0322	-1.021052851	1.629645418	0.006209685	0.026648344
PARG2878	1.205256689	0.921278654	0.006283709	0.026921853
PARG0792	1.211450352	3.726125844	0.00661035	0.028152273
PARG0231	1.286844075	1.29757881	0.006869763	0.029114885
PARG2530	-1.024574949	6.180139941	0.006885669	0.029150934
PARG0804	2.938837021	-0.202807372	0.006889411	0.029150934
PARG1902	-2.620641985	2.501727181	0.006889411	0.029150934
PARG2056	2.563852861	2.933104569	0.006889411	0.029150934
PARG2466	2.560913189	0.250271959	0.006889411	0.029150934
PARG2398	-1.312723625	-0.468047977	0.007238025	0.030388479
PARG0213	1.002216225	2.048587727	0.007376775	0.030871933
PARG2441	-1.493099091	1.992789114	0.007464685	0.03114849

PARG1903	1.489773429	-0.118393149	0.007464685	0.03114849
PARG1950	-1.075360993	0.320344363	0.007554319	0.031463971
PARG2954	-1.212154747	0.395965215	0.007691098	0.031974273
PARG0581	1.065027432	0.088935236	0.007708837	0.032031054
PARG0653	1.516693817	1.74183158	0.007781299	0.032315033
PARG2995	2.245025574	3.323083703	0.007916445	0.032806853
PARG0873	-2.238925369	1.105009143	0.007916445	0.032806853
PARG2101	-1.039430479	2.422699276	0.007926949	0.032841713
PARG2390	1.712740133	5.026243499	0.008084257	0.033352605
PARG2686	-1.648361706	0.19223022	0.008084257	0.033352605
PARG0897	-1.628144916	-0.315840781	0.008084257	0.033352605
PARG0484	-1.620949074	-0.383173196	0.008084257	0.033352605
PARG0206	1.559930803	0.73519827	0.008084257	0.033352605
PARG1115	1.556643832	2.256872735	0.008084257	0.033352605
PARG1779	1.145593281	1.938744668	0.008127021	0.033502618
PARG2712	1.063904345	1.657372107	0.008245631	0.033938101
PARG1670	1.047288376	0.676152039	0.00838	0.034400954
PARG1424	-2.094827763	1.261228928	0.008469227	0.034631393
PARG1081	2.04598927	0.045123471	0.008469227	0.034631393
PARG2854	-2.039401772	2.850432838	0.008469227	0.034631393
PARG0212	2.028692448	0.729159097	0.008469227	0.034631393
PARG0437	2.024824146	-0.157956296	0.008469227	0.034631393
PARG2786	-1.374358714	5.298117805	0.00850406	0.034746678
PARG2764	-1.767636877	2.294274289	0.008576896	0.035007831
PARG2185	1.698997733	-0.492789703	0.008576896	0.035007831
PARG1984	1.337351983	1.193072891	0.008686128	0.03538008
PARG0154	-1.276112181	-0.074542392	0.008686128	0.03538008
PARG1742	1.865154395	-0.065522858	0.008696644	0.035404541
PARG0860	1.823305593	1.093749946	0.008707282	0.035420292
PARG2073	1.783676478	-0.408066321	0.008707282	0.035420292
PARG1413	1.782041564	2.824851659	0.008707282	0.035420292
PARG0377	3.084603544	2.478522529	0.008991581	0.036378825
PARG1388	-3.051523173	-0.095778216	0.008991581	0.036378825
PARG1406	3.022493129	1.799831587	0.008991581	0.036378825
PARG1188	3.019124074	3.375308237	0.008991581	0.036378825
PARG0435	3.018185696	1.550430624	0.008991581	0.036378825
PARG1762	3.013784772	2.453462159	0.008991581	0.036378825
PARG2771	3.012688798	0.678088319	0.008991581	0.036378825
PARG2816	-1.25195754	1.349600709	0.00911641	0.036770144
PARG1612	-1.023671216	0.535810996	0.009230922	0.037137955
PARG2092	1.042550821	0.639462515	0.009258332	0.037230444
PARG2361	1.002015619	0.943965767	0.009258992	0.037230444
PARG1989	-1.124401432	0.927750261	0.009401498	0.037700581
PARG1894	1.113614362	1.310422422	0.009401498	0.037700581
PARG2486	-1.361528579	0.449344966	0.009486347	0.037921233
PARG2687	-1.26590702	0.965381615	0.009678602	0.038573857

PARG0637	1.193891701	0.892805943	0.009678602	0.038573857
PARG0936	-4.498223397	0.186592623	0.009732353	0.038736537
PARG2050	4.465202812	0.613190988	0.009732353	0.038736537
PARG1494	4.462536755	0.831215072	0.009732353	0.038736537
PARG0028	-1.025713511	1.694341369	0.00973472	0.038736537
PARG0118	1.121477139	0.989168654	0.009977957	0.039564024
PARG0785	1.588001532	3.881042266	0.010014438	0.039678609
PARG1373	1.133456318	1.109725994	0.010253959	0.04047439
PARG1372	-1.085530479	0.875639029	0.010253959	0.04047439
PARG0515	-1.176181317	-0.15793357	0.010608352	0.041570122
PARG2398	1.170646572	3.142116895	0.010608352	0.041570122
PARG1237	1.165487846	3.261618279	0.010608352	0.041570122
PARG0925	1.166499972	0.11650781	0.010787353	0.042173747
PARG1692	-1.019111172	2.923961305	0.010862394	0.042406829
PARG0758	-2.54107694	0.988269739	0.011249028	0.043706579
PARG2056	2.46297462	2.340132712	0.011249028	0.043706579
PARG1995	2.462871389	1.047331195	0.011249028	0.043706579
PARG2116	-1.100912409	1.653448579	0.011476334	0.044406087
PARG2992	1.568037353	1.481940937	0.011654683	0.0449853
PARG1551	-1.413386843	0.739893135	0.011786292	0.045426274
PARG1845	-1.218259045	0.808520555	0.012057704	0.046272095
PARG3006	1.030383149	1.761027981	0.012058806	0.046272095
PARG0726	1.623273176	3.103268069	0.012157965	0.046584266
PARG0359	-1.150455648	0.972349394	0.012232431	0.046801049
PARG0079	-1.08263192	1.490970947	0.01225655	0.046861054
PARG2126	-2.233764339	0.731995485	0.01240086	0.047318596
PARG2453	-1.99088174	-0.402603844	0.01240086	0.047318596
PARG0056	1.338629285	0.746049578	0.012505193	0.047658779
PARG2780	1.259213157	1.225197391	0.012505193	0.047658779
PARG2139	2.012370962	-0.673613906	0.012842158	0.048732642
PARG2033	-1.960967304	0.358615825	0.012842158	0.048732642
PARG1268	-1.95736401	0.491906799	0.012842158	0.048732642
PARG2741	-1.885971601	-0.064218902	0.012842842	0.048732642
PARG2713	1.809334849	1.781868154	0.012842842	0.048732642
PARG2396	1.801714903	2.316396417	0.012842842	0.048732642
PARG2910	-1.040897511	1.294904171	0.012921318	0.048959414
PARG1190	-1.16179348	0.727247897	0.01306413	0.049345698
PARG2790	-1.156924751	-0.159716099	0.01306413	0.049345698
PARG2852	1.140374573	0.672862897	0.01306413	0.049345698
PARG2308	1.07237228	-0.06666023	0.01306413	0.049345698

Table S18. The list of High-quality annotation of *P. armeniaca*

PARG00003	PARG00006	PARG00007	PARG00008	PARG00009	PARG00010	PARG00012
PARG00013	PARG00014	PARG00015	PARG00016	PARG00019	PARG00020	PARG00021
PARG00022	PARG00023	PARG00024	PARG00025	PARG00026	PARG00027	PARG00028
PARG00029	PARG00030	PARG00031	PARG00033	PARG00034	PARG00035	PARG00036
PARG00037	PARG00038	PARG00039	PARG00040	PARG00041	PARG00042	PARG00043
PARG00044	PARG00045	PARG00046	PARG00048	PARG00049	PARG00050	PARG00051
PARG00052	PARG00054	PARG00056	PARG00057	PARG00058	PARG00059	PARG00060
PARG00061	PARG00063	PARG00064	PARG00065	PARG00066	PARG00073	PARG00075
PARG00076	PARG00077	PARG00078	PARG00079	PARG00080	PARG00081	PARG00082
PARG00087	PARG00088	PARG00090	PARG00092	PARG00093	PARG00094	PARG00095
PARG00096	PARG00097	PARG00098	PARG00099	PARG00100	PARG00101	PARG00103
PARG00104	PARG00105	PARG00106	PARG00107	PARG00112	PARG00114	PARG00115
PARG00116	PARG00117	PARG00118	PARG00119	PARG00120	PARG00122	PARG00123
PARG00124	PARG00125	PARG00126	PARG00127	PARG00128	PARG00129	PARG00130
PARG00131	PARG00132	PARG00133	PARG00134	PARG00136	PARG00137	PARG00138
PARG00141	PARG00142	PARG00143	PARG00144	PARG00152	PARG00153	PARG00161
PARG00162	PARG00165	PARG00167	PARG00168	PARG00169	PARG00170	PARG00171
PARG00172	PARG00173	PARG00174	PARG00175	PARG00176	PARG00178	PARG00179
PARG00180	PARG00182	PARG00183	PARG00184	PARG00185	PARG00186	PARG00188
PARG00190	PARG00191	PARG00192	PARG00194	PARG00195	PARG00196	PARG00197
PARG00198	PARG00199	PARG00200	PARG00201	PARG00204	PARG00205	PARG00206
PARG00207	PARG00208	PARG00209	PARG00211	PARG00213	PARG00214	PARG00215
PARG00216	PARG00217	PARG00218	PARG00219	PARG00220	PARG00221	PARG00222
PARG00223	PARG00224	PARG00225	PARG00226	PARG00227	PARG00228	PARG00229
PARG00230	PARG00233	PARG00234	PARG00235	PARG00236	PARG00237	PARG00238
PARG00239	PARG00242	PARG00243	PARG00244	PARG00246	PARG00247	PARG00248
PARG00249	PARG00250	PARG00251	PARG00252	PARG00253	PARG00255	PARG00256
PARG00258	PARG00259	PARG00260	PARG00263	PARG00264	PARG00265	PARG00266
PARG00267	PARG00268	PARG00269	PARG00272	PARG00273	PARG00279	PARG00280
PARG00281	PARG00282	PARG00283	PARG00284	PARG00285	PARG00286	PARG00287
PARG00288	PARG00289	PARG00290	PARG00291	PARG00293	PARG00294	PARG00296
PARG00297	PARG00298	PARG00299	PARG00300	PARG00301	PARG00302	PARG00303
PARG00304	PARG00305	PARG00306	PARG00307	PARG00308	PARG00309	PARG00310
PARG00311	PARG00312	PARG00313	PARG00315	PARG00316	PARG00317	PARG00318
PARG00319	PARG00321	PARG00322	PARG00323	PARG00325	PARG00326	PARG00327
PARG00328	PARG00329	PARG00330	PARG00333	PARG00334	PARG00335	PARG00336
PARG00337	PARG00338	PARG00339	PARG00340	PARG00341	PARG00342	PARG00343
PARG00344	PARG00345	PARG00346	PARG00347	PARG00348	PARG00349	PARG00350
PARG00351	PARG00354	PARG00355	PARG00356	PARG00357	PARG00358	PARG00359
PARG00360	PARG00361	PARG00362	PARG00363	PARG00364	PARG00365	PARG00366
PARG00367	PARG00368	PARG00369	PARG00370	PARG00371	PARG00372	PARG00373
PARG00374	PARG00375	PARG00376	PARG00377	PARG00378	PARG00379	PARG00380
PARG00381	PARG00383	PARG00384	PARG00385	PARG00386	PARG00387	PARG00388
PARG00389	PARG00390	PARG00391	PARG00392	PARG00393	PARG00394	PARG00395
PARG00396	PARG00397	PARG00398	PARG00400	PARG00401	PARG00402	PARG00403

PARG00404	PARG00405	PARG00406	PARG00410	PARG00411	PARG00412	PARG00413
PARG00414	PARG00415	PARG00416	PARG00417	PARG00418	PARG00419	PARG00420
PARG00421	PARG00422	PARG00423	PARG00424	PARG00425	PARG00426	PARG00427
PARG00428	PARG00429	PARG00430	PARG00431	PARG00432	PARG00433	PARG00434
PARG00435	PARG00436	PARG00437	PARG00438	PARG00439	PARG00440	PARG00441
PARG00443	PARG00445	PARG00446	PARG00448	PARG00449	PARG00450	PARG00451
PARG00452	PARG00453	PARG00454	PARG00455	PARG00456	PARG00457	PARG00458
PARG00460	PARG00461	PARG00462	PARG00464	PARG00465	PARG00466	PARG00467
PARG00468	PARG00469	PARG00470	PARG00471	PARG00472	PARG00473	PARG00474
PARG00475	PARG00476	PARG00477	PARG00478	PARG00480	PARG00481	PARG00482
PARG00483	PARG00485	PARG00486	PARG00487	PARG00488	PARG00489	PARG00490
PARG00491	PARG00492	PARG00493	PARG00496	PARG00497	PARG00498	PARG00499
PARG00500	PARG00501	PARG00502	PARG00503	PARG00504	PARG00505	PARG00506
PARG00507	PARG00508	PARG00509	PARG00510	PARG00511	PARG00512	PARG00513
PARG00514	PARG00515	PARG00516	PARG00517	PARG00518	PARG00519	PARG00520
PARG00521	PARG00522	PARG00524	PARG00525	PARG00526	PARG00528	PARG00529
PARG00530	PARG00531	PARG00532	PARG00535	PARG00538	PARG00539	PARG00540
PARG00541	PARG00542	PARG00543	PARG00544	PARG00545	PARG00546	PARG00547
PARG00548	PARG00549	PARG00550	PARG00551	PARG00552	PARG00553	PARG00554
PARG00555	PARG00556	PARG00557	PARG00558	PARG00559	PARG00560	PARG00561
PARG00562	PARG00563	PARG00564	PARG00565	PARG00566	PARG00567	PARG00568
PARG00570	PARG00571	PARG00572	PARG00573	PARG00574	PARG00575	PARG00578
PARG00579	PARG00580	PARG00581	PARG00582	PARG00583	PARG00584	PARG00585
PARG00586	PARG00587	PARG00588	PARG00590	PARG00591	PARG00593	PARG00594
PARG00595	PARG00596	PARG00597	PARG00598	PARG00599	PARG00600	PARG00602
PARG00603	PARG00604	PARG00605	PARG00606	PARG00607	PARG00608	PARG00609
PARG00610	PARG00611	PARG00612	PARG00613	PARG00614	PARG00615	PARG00616
PARG00618	PARG00619	PARG00620	PARG00622	PARG00623	PARG00624	PARG00625
PARG00626	PARG00627	PARG00628	PARG00629	PARG00630	PARG00631	PARG00632
PARG00633	PARG00634	PARG00635	PARG00636	PARG00637	PARG00638	PARG00639
PARG00640	PARG00641	PARG00642	PARG00643	PARG00644	PARG00645	PARG00646
PARG00647	PARG00648	PARG00651	PARG00652	PARG00653	PARG00654	PARG00655
PARG00656	PARG00657	PARG00658	PARG00659	PARG00660	PARG00661	PARG00662
PARG00663	PARG00664	PARG00665	PARG00666	PARG00667	PARG00668	PARG00669
PARG00670	PARG00671	PARG00672	PARG00673	PARG00677	PARG00678	PARG00679
PARG00682	PARG00683	PARG00684	PARG00685	PARG00686	PARG00687	PARG00688
PARG00693	PARG00694	PARG00695	PARG00696	PARG00697	PARG00698	PARG00699
PARG00700	PARG00701	PARG00703	PARG00704	PARG00705	PARG00706	PARG00707
PARG00708	PARG00709	PARG00710	PARG00711	PARG00712	PARG00713	PARG00714
PARG00715	PARG00716	PARG00717	PARG00718	PARG00719	PARG00721	PARG00722
PARG00723	PARG00724	PARG00725	PARG00726	PARG00727	PARG00728	PARG00729
PARG00730	PARG00731	PARG00732	PARG00733	PARG00734	PARG00735	PARG00736
PARG00737	PARG00738	PARG00739	PARG00740	PARG00742	PARG00743	PARG00744
PARG00745	PARG00746	PARG00747	PARG00748	PARG00749	PARG00750	PARG00751
PARG00753	PARG00754	PARG00755	PARG00757	PARG00758	PARG00759	PARG00761
PARG00762	PARG00763	PARG00764	PARG00765	PARG00766	PARG00767	PARG00768

PARG00769	PARG00770	PARG00771	PARG00772	PARG00773	PARG00774	PARG00775
PARG00776	PARG00777	PARG00778	PARG00780	PARG00781	PARG00782	PARG00783
PARG00784	PARG00785	PARG00788	PARG00789	PARG00790	PARG00791	PARG00793
PARG00795	PARG00796	PARG00797	PARG00798	PARG00799	PARG00800	PARG00801
PARG00802	PARG00803	PARG00808	PARG00809	PARG00810	PARG00811	PARG00812
PARG00813	PARG00815	PARG00817	PARG00818	PARG00819	PARG00820	PARG00821
PARG00822	PARG00823	PARG00824	PARG00825	PARG00826	PARG00827	PARG00828
PARG00829	PARG00830	PARG00831	PARG00832	PARG00833	PARG00834	PARG00836
PARG00837	PARG00838	PARG00839	PARG00840	PARG00841	PARG00842	PARG00843
PARG00844	PARG00845	PARG00846	PARG00847	PARG00849	PARG00850	PARG00851
PARG00852	PARG00853	PARG00854	PARG00857	PARG00858	PARG00859	PARG00860
PARG00861	PARG00862	PARG00863	PARG00864	PARG00865	PARG00869	PARG00870
PARG00874	PARG00875	PARG00876	PARG00878	PARG00879	PARG00880	PARG00881
PARG00882	PARG00883	PARG00884	PARG00885	PARG00886	PARG00887	PARG00888
PARG00889	PARG00890	PARG00891	PARG00892	PARG00893	PARG00894	PARG00895
PARG00896	PARG00899	PARG00901	PARG00903	PARG00904	PARG00905	PARG00906
PARG00907	PARG00908	PARG00909	PARG00910	PARG00911	PARG00912	PARG00913
PARG00914	PARG00915	PARG00916	PARG00917	PARG00918	PARG00919	PARG00920
PARG00921	PARG00922	PARG00923	PARG00924	PARG00925	PARG00926	PARG00927
PARG00928	PARG00929	PARG00930	PARG00931	PARG00936	PARG00937	PARG00938
PARG00939	PARG00940	PARG00941	PARG00942	PARG00943	PARG00944	PARG00945
PARG00946	PARG00947	PARG00948	PARG00949	PARG00950	PARG00952	PARG00953
PARG00954	PARG00955	PARG00956	PARG00957	PARG00958	PARG00960	PARG00962
PARG00963	PARG00964	PARG00965	PARG00966	PARG00967	PARG00968	PARG00969
PARG00970	PARG00971	PARG00972	PARG00973	PARG00974	PARG00975	PARG00976
PARG00978	PARG00979	PARG00981	PARG00982	PARG00983	PARG00984	PARG00985
PARG00986	PARG00987	PARG00988	PARG00989	PARG00990	PARG00991	PARG00992
PARG00994	PARG00995	PARG00996	PARG00997	PARG00998	PARG00999	PARG01000
PARG01001	PARG01002	PARG01003	PARG01004	PARG01006	PARG01007	PARG01008
PARG01010	PARG01011	PARG01013	PARG01014	PARG01015	PARG01016	PARG01021
PARG01022	PARG01023	PARG01024	PARG01028	PARG01029	PARG01030	PARG01031
PARG01032	PARG01033	PARG01041	PARG01042	PARG01043	PARG01044	PARG01046
PARG01047	PARG01049	PARG01050	PARG01053	PARG01054	PARG01056	PARG01059
PARG01060	PARG01061	PARG01062	PARG01063	PARG01064	PARG01065	PARG01066
PARG01067	PARG01068	PARG01070	PARG01071	PARG01072	PARG01073	PARG01074
PARG01075	PARG01077	PARG01078	PARG01079	PARG01080	PARG01082	PARG01083
PARG01084	PARG01085	PARG01086	PARG01087	PARG01088	PARG01089	PARG01090
PARG01091	PARG01092	PARG01093	PARG01095	PARG01096	PARG01097	PARG01098
PARG01099	PARG01101	PARG01102	PARG01103	PARG01104	PARG01107	PARG01108
PARG01109	PARG01110	PARG01111	PARG01112	PARG01113	PARG01114	PARG01116
PARG01117	PARG01118	PARG01120	PARG01121	PARG01122	PARG01123	PARG01124
PARG01126	PARG01127	PARG01128	PARG01134	PARG01135	PARG01136	PARG01138
PARG01139	PARG01140	PARG01141	PARG01144	PARG01145	PARG01146	PARG01147
PARG01148	PARG01149	PARG01150	PARG01151	PARG01152	PARG01155	PARG01156
PARG01157	PARG01158	PARG01159	PARG01163	PARG01165	PARG01167	PARG01168
PARG01173	PARG01174	PARG01175	PARG01176	PARG01177	PARG01178	PARG01179

PARG01181	PARG01190	PARG01192	PARG01193	PARG01195	PARG01196	PARG01197
PARG01198	PARG01199	PARG01200	PARG01201	PARG01202	PARG01203	PARG01204
PARG01205	PARG01206	PARG01207	PARG01208	PARG01209	PARG01210	PARG01213
PARG01214	PARG01215	PARG01221	PARG01222	PARG01223	PARG01226	PARG01228
PARG01233	PARG01234	PARG01235	PARG01236	PARG01237	PARG01238	PARG01239
PARG01242	PARG01243	PARG01244	PARG01245	PARG01247	PARG01248	PARG01249
PARG01250	PARG01251	PARG01252	PARG01253	PARG01254	PARG01255	PARG01256
PARG01257	PARG01258	PARG01260	PARG01261	PARG01263	PARG01264	PARG01265
PARG01266	PARG01267	PARG01268	PARG01269	PARG01270	PARG01271	PARG01272
PARG01275	PARG01277	PARG01278	PARG01280	PARG01282	PARG01285	PARG01286
PARG01289	PARG01290	PARG01291	PARG01294	PARG01298	PARG01299	PARG01300
PARG01301	PARG01303	PARG01306	PARG01310	PARG01311	PARG01312	PARG01316
PARG01317	PARG01318	PARG01319	PARG01320	PARG01324	PARG01329	PARG01330
PARG01332	PARG01333	PARG01334	PARG01335	PARG01336	PARG01337	PARG01338
PARG01339	PARG01341	PARG01344	PARG01345	PARG01346	PARG01347	PARG01348
PARG01350	PARG01355	PARG01356	PARG01357	PARG01358	PARG01359	PARG01361
PARG01363	PARG01364	PARG01365	PARG01366	PARG01369	PARG01370	PARG01371
PARG01372	PARG01373	PARG01374	PARG01375	PARG01376	PARG01377	PARG01378
PARG01379	PARG01380	PARG01381	PARG01382	PARG01383	PARG01384	PARG01385
PARG01389	PARG01390	PARG01391	PARG01392	PARG01393	PARG01394	PARG01402
PARG01404	PARG01406	PARG01412	PARG01414	PARG01416	PARG01417	PARG01418
PARG01419	PARG01420	PARG01421	PARG01423	PARG01424	PARG01425	PARG01427
PARG01429	PARG01432	PARG01433	PARG01434	PARG01435	PARG01436	PARG01437
PARG01438	PARG01439	PARG01443	PARG01445	PARG01446	PARG01448	PARG01449
PARG01450	PARG01451	PARG01452	PARG01453	PARG01455	PARG01456	PARG01457
PARG01458	PARG01460	PARG01463	PARG01464	PARG01465	PARG01468	PARG01470
PARG01473	PARG01475	PARG01484	PARG01486	PARG01487	PARG01488	PARG01490
PARG01491	PARG01494	PARG01495	PARG01496	PARG01497	PARG01499	PARG01501
PARG01503	PARG01504	PARG01505	PARG01507	PARG01510	PARG01511	PARG01512
PARG01513	PARG01514	PARG01515	PARG01516	PARG01521	PARG01524	PARG01525
PARG01526	PARG01533	PARG01548	PARG01549	PARG01550	PARG01552	PARG01553
PARG01554	PARG01555	PARG01559	PARG01560	PARG01562	PARG01564	PARG01565
PARG01566	PARG01568	PARG01570	PARG01571	PARG01572	PARG01573	PARG01575
PARG01576	PARG01578	PARG01579	PARG01581	PARG01583	PARG01584	PARG01590
PARG01591	PARG01593	PARG01594	PARG01595	PARG01597	PARG01598	PARG01600
PARG01601	PARG01602	PARG01605	PARG01606	PARG01611	PARG01613	PARG01615
PARG01616	PARG01617	PARG01618	PARG01622	PARG01636	PARG01638	PARG01639
PARG01641	PARG01642	PARG01643	PARG01644	PARG01645	PARG01647	PARG01652
PARG01655	PARG01659	PARG01663	PARG01665	PARG01666	PARG01667	PARG01669
PARG01670	PARG01672	PARG01673	PARG01674	PARG01676	PARG01677	PARG01681
PARG01683	PARG01687	PARG01689	PARG01694	PARG01695	PARG01696	PARG01699
PARG01700	PARG01701	PARG01702	PARG01703	PARG01704	PARG01715	PARG01716
PARG01728	PARG01729	PARG01733	PARG01736	PARG01740	PARG01741	PARG01742
PARG01745	PARG01746	PARG01747	PARG01748	PARG01749	PARG01750	PARG01752
PARG01753	PARG01757	PARG01758	PARG01759	PARG01760	PARG01762	PARG01763
PARG01764	PARG01776	PARG01777	PARG01778	PARG01780	PARG01781	PARG01782

PARG01783	PARG01784	PARG01786	PARG01789	PARG01790	PARG01791	PARG01792
PARG01793	PARG01794	PARG01796	PARG01802	PARG01803	PARG01808	PARG01809
PARG01811	PARG01820	PARG01821	PARG01822	PARG01823	PARG01824	PARG01826
PARG01827	PARG01828	PARG01829	PARG01830	PARG01832	PARG01833	PARG01834
PARG01835	PARG01836	PARG01837	PARG01838	PARG01839	PARG01840	PARG01841
PARG01842	PARG01843	PARG01844	PARG01845	PARG01846	PARG01847	PARG01849
PARG01850	PARG01851	PARG01854	PARG01855	PARG01856	PARG01857	PARG01858
PARG01859	PARG01860	PARG01861	PARG01862	PARG01863	PARG01864	PARG01865
PARG01869	PARG01870	PARG01871	PARG01874	PARG01875	PARG01876	PARG01877
PARG01878	PARG01879	PARG01880	PARG01881	PARG01882	PARG01883	PARG01884
PARG01885	PARG01886	PARG01888	PARG01889	PARG01892	PARG01893	PARG01894
PARG01895	PARG01896	PARG01897	PARG01900	PARG01901	PARG01903	PARG01904
PARG01905	PARG01906	PARG01907	PARG01909	PARG01910	PARG01911	PARG01912
PARG01914	PARG01915	PARG01916	PARG01917	PARG01918	PARG01919	PARG01920
PARG01921	PARG01922	PARG01923	PARG01924	PARG01925	PARG01927	PARG01930
PARG01934	PARG01938	PARG01939	PARG01942	PARG01950	PARG01951	PARG01953
PARG01954	PARG01955	PARG01956	PARG01957	PARG01958	PARG01959	PARG01960
PARG01962	PARG01963	PARG01964	PARG01965	PARG01966	PARG01967	PARG01968
PARG01969	PARG01970	PARG01971	PARG01972	PARG01973	PARG01974	PARG01976
PARG01977	PARG01978	PARG01979	PARG01980	PARG01984	PARG01985	PARG01987
PARG01988	PARG01989	PARG01990	PARG01991	PARG01992	PARG01993	PARG01994
PARG01996	PARG01997	PARG01998	PARG01999	PARG02000	PARG02002	PARG02003
PARG02005	PARG02006	PARG02007	PARG02008	PARG02009	PARG02010	PARG02011
PARG02012	PARG02013	PARG02014	PARG02015	PARG02016	PARG02018	PARG02019
PARG02020	PARG02021	PARG02024	PARG02025	PARG02026	PARG02027	PARG02032
PARG02033	PARG02034	PARG02035	PARG02036	PARG02038	PARG02039	PARG02040
PARG02041	PARG02042	PARG02044	PARG02045	PARG02046	PARG02047	PARG02048
PARG02049	PARG02050	PARG02052	PARG02053	PARG02054	PARG02055	PARG02056
PARG02057	PARG02058	PARG02059	PARG02060	PARG02061	PARG02062	PARG02063
PARG02064	PARG02065	PARG02066	PARG02067	PARG02068	PARG02069	PARG02070
PARG02071	PARG02072	PARG02073	PARG02075	PARG02076	PARG02077	PARG02078
PARG02079	PARG02080	PARG02081	PARG02082	PARG02083	PARG02084	PARG02085
PARG02086	PARG02087	PARG02088	PARG02089	PARG02090	PARG02091	PARG02092
PARG02093	PARG02094	PARG02095	PARG02096	PARG02097	PARG02098	PARG02099
PARG02100	PARG02101	PARG02102	PARG02103	PARG02104	PARG02105	PARG02106
PARG02107	PARG02108	PARG02109	PARG02110	PARG02112	PARG02113	PARG02114
PARG02115	PARG02116	PARG02117	PARG02119	PARG02120	PARG02121	PARG02122
PARG02123	PARG02124	PARG02125	PARG02126	PARG02127	PARG02129	PARG02130
PARG02131	PARG02132	PARG02133	PARG02134	PARG02135	PARG02136	PARG02137
PARG02138	PARG02139	PARG02141	PARG02142	PARG02143	PARG02144	PARG02145
PARG02146	PARG02147	PARG02148	PARG02149	PARG02150	PARG02151	PARG02152
PARG02153	PARG02154	PARG02155	PARG02157	PARG02159	PARG02160	PARG02162
PARG02163	PARG02164	PARG02165	PARG02166	PARG02167	PARG02168	PARG02169
PARG02170	PARG02171	PARG02172	PARG02173	PARG02174	PARG02175	PARG02176
PARG02177	PARG02178	PARG02179	PARG02180	PARG02181	PARG02182	PARG02183
PARG02184	PARG02185	PARG02186	PARG02187	PARG02188	PARG02189	PARG02190

PARG02191	PARG02192	PARG02193	PARG02194	PARG02195	PARG02196	PARG02198
PARG02199	PARG02200	PARG02201	PARG02202	PARG02203	PARG02204	PARG02208
PARG02209	PARG02210	PARG02211	PARG02212	PARG02213	PARG02214	PARG02215
PARG02216	PARG02217	PARG02218	PARG02220	PARG02221	PARG02222	PARG02223
PARG02224	PARG02225	PARG02226	PARG02228	PARG02229	PARG02230	PARG02231
PARG02232	PARG02233	PARG02234	PARG02235	PARG02236	PARG02237	PARG02238
PARG02239	PARG02240	PARG02241	PARG02242	PARG02243	PARG02244	PARG02245
PARG02248	PARG02249	PARG02250	PARG02251	PARG02252	PARG02253	PARG02254
PARG02255	PARG02256	PARG02257	PARG02258	PARG02259	PARG02260	PARG02261
PARG02262	PARG02263	PARG02264	PARG02267	PARG02268	PARG02269	PARG02270
PARG02271	PARG02272	PARG02273	PARG02274	PARG02275	PARG02276	PARG02277
PARG02278	PARG02279	PARG02280	PARG02281	PARG02282	PARG02283	PARG02284
PARG02285	PARG02286	PARG02287	PARG02288	PARG02289	PARG02290	PARG02291
PARG02292	PARG02293	PARG02294	PARG02295	PARG02297	PARG02298	PARG02299
PARG02300	PARG02302	PARG02303	PARG02304	PARG02306	PARG02307	PARG02308
PARG02310	PARG02311	PARG02312	PARG02314	PARG02315	PARG02316	PARG02317
PARG02318	PARG02319	PARG02320	PARG02321	PARG02322	PARG02323	PARG02324
PARG02326	PARG02327	PARG02328	PARG02329	PARG02330	PARG02331	PARG02332
PARG02333	PARG02334	PARG02335	PARG02336	PARG02337	PARG02338	PARG02339
PARG02340	PARG02342	PARG02343	PARG02345	PARG02346	PARG02347	PARG02348
PARG02349	PARG02351	PARG02352	PARG02353	PARG02354	PARG02355	PARG02356
PARG02357	PARG02358	PARG02360	PARG02361	PARG02362	PARG02363	PARG02364
PARG02365	PARG02366	PARG02367	PARG02368	PARG02369	PARG02370	PARG02371
PARG02372	PARG02373	PARG02374	PARG02375	PARG02376	PARG02377	PARG02378
PARG02380	PARG02381	PARG02382	PARG02383	PARG02384	PARG02394	PARG02396
PARG02397	PARG02398	PARG02399	PARG02400	PARG02401	PARG02402	PARG02404
PARG02405	PARG02407	PARG02408	PARG02409	PARG02410	PARG02411	PARG02412
PARG02413	PARG02414	PARG02416	PARG02417	PARG02418	PARG02419	PARG02420
PARG02421	PARG02422	PARG02423	PARG02424	PARG02425	PARG02426	PARG02427
PARG02428	PARG02429	PARG02430	PARG02431	PARG02432	PARG02437	PARG02438
PARG02441	PARG02443	PARG02444	PARG02445	PARG02446	PARG02447	PARG02448
PARG02449	PARG02450	PARG02451	PARG02452	PARG02453	PARG02454	PARG02455
PARG02456	PARG02457	PARG02458	PARG02459	PARG02460	PARG02464	PARG02465
PARG02466	PARG02467	PARG02468	PARG02469	PARG02470	PARG02471	PARG02472
PARG02473	PARG02474	PARG02475	PARG02476	PARG02477	PARG02478	PARG02479
PARG02480	PARG02481	PARG02482	PARG02483	PARG02484	PARG02485	PARG02486
PARG02489	PARG02490	PARG02491	PARG02493	PARG02494	PARG02495	PARG02496
PARG02497	PARG02498	PARG02501	PARG02502	PARG02503	PARG02504	PARG02505
PARG02506	PARG02507	PARG02508	PARG02509	PARG02510	PARG02511	PARG02515
PARG02517	PARG02518	PARG02519	PARG02520	PARG02521	PARG02522	PARG02523
PARG02526	PARG02527	PARG02531	PARG02532	PARG02535	PARG02536	PARG02537
PARG02538	PARG02539	PARG02543	PARG02544	PARG02546	PARG02547	PARG02548
PARG02549	PARG02550	PARG02551	PARG02552	PARG02553	PARG02554	PARG02555
PARG02556	PARG02557	PARG02566	PARG02578	PARG02579	PARG02580	PARG02584
PARG02588	PARG02591	PARG02593	PARG02594	PARG02595	PARG02596	PARG02598
PARG02600	PARG02602	PARG02603	PARG02605	PARG02606	PARG02607	PARG02612

PARG02613	PARG02614	PARG02615	PARG02617	PARG02619	PARG02620	PARG02623
PARG02626	PARG02627	PARG02629	PARG02630	PARG02632	PARG02637	PARG02638
PARG02640	PARG02641	PARG02642	PARG02649	PARG02650	PARG02651	PARG02652
PARG02655	PARG02656	PARG02657	PARG02658	PARG02659	PARG02660	PARG02661
PARG02662	PARG02663	PARG02664	PARG02665	PARG02666	PARG02667	PARG02668
PARG02669	PARG02670	PARG02671	PARG02673	PARG02674	PARG02675	PARG02676
PARG02677	PARG02678	PARG02680	PARG02681	PARG02682	PARG02683	PARG02684
PARG02685	PARG02686	PARG02687	PARG02688	PARG02689	PARG02691	PARG02692
PARG02693	PARG02694	PARG02695	PARG02696	PARG02700	PARG02701	PARG02702
PARG02703	PARG02704	PARG02706	PARG02708	PARG02709	PARG02710	PARG02711
PARG02712	PARG02713	PARG02714	PARG02715	PARG02716	PARG02717	PARG02718
PARG02720	PARG02721	PARG02722	PARG02723	PARG02724	PARG02725	PARG02726
PARG02727	PARG02730	PARG02731	PARG02732	PARG02733	PARG02734	PARG02735
PARG02736	PARG02737	PARG02739	PARG02740	PARG02741	PARG02742	PARG02743
PARG02744	PARG02745	PARG02746	PARG02747	PARG02748	PARG02749	PARG02750
PARG02751	PARG02752	PARG02753	PARG02754	PARG02755	PARG02756	PARG02757
PARG02759	PARG02761	PARG02765	PARG02766	PARG02771	PARG02772	PARG02773
PARG02774	PARG02775	PARG02776	PARG02777	PARG02778	PARG02779	PARG02780
PARG02781	PARG02782	PARG02783	PARG02784	PARG02785	PARG02786	PARG02787
PARG02788	PARG02789	PARG02797	PARG02798	PARG02800	PARG02802	PARG02803
PARG02804	PARG02806	PARG02807	PARG02808	PARG02810	PARG02811	PARG02812
PARG02813	PARG02814	PARG02815	PARG02816	PARG02817	PARG02818	PARG02819
PARG02820	PARG02822	PARG02823	PARG02824	PARG02825	PARG02826	PARG02827
PARG02828	PARG02829	PARG02830	PARG02831	PARG02832	PARG02833	PARG02834
PARG02835	PARG02836	PARG02837	PARG02838	PARG02839	PARG02840	PARG02841
PARG02842	PARG02843	PARG02844	PARG02845	PARG02846	PARG02848	PARG02849
PARG02850	PARG02851	PARG02852	PARG02853	PARG02854	PARG02855	PARG02856
PARG02857	PARG02858	PARG02859	PARG02860	PARG02861	PARG02863	PARG02865
PARG02866	PARG02867	PARG02868	PARG02869	PARG02870	PARG02871	PARG02872
PARG02873	PARG02874	PARG02875	PARG02876	PARG02877	PARG02878	PARG02879
PARG02881	PARG02882	PARG02883	PARG02884	PARG02885	PARG02886	PARG02887
PARG02888	PARG02889	PARG02890	PARG02891	PARG02892	PARG02893	PARG02894
PARG02895	PARG02896	PARG02897	PARG02898	PARG02899	PARG02900	PARG02901
PARG02902	PARG02903	PARG02904	PARG02905	PARG02906	PARG02907	PARG02908
PARG02909	PARG02910	PARG02911	PARG02912	PARG02913	PARG02914	PARG02915
PARG02916	PARG02917	PARG02918	PARG02919	PARG02920	PARG02921	PARG02922
PARG02923	PARG02924	PARG02925	PARG02926	PARG02927	PARG02928	PARG02929
PARG02930	PARG02931	PARG02932	PARG02933	PARG02934	PARG02935	PARG02936
PARG02937	PARG02938	PARG02939	PARG02940	PARG02941	PARG02942	PARG02943
PARG02944	PARG02945	PARG02946	PARG02947	PARG02948	PARG02949	PARG02950
PARG02951	PARG02952	PARG02953	PARG02954	PARG02955	PARG02956	PARG02957
PARG02958	PARG02959	PARG02960	PARG02961	PARG02962	PARG02963	PARG02964
PARG02965	PARG02966	PARG02967	PARG02968	PARG02975	PARG02976	PARG02977
PARG02978	PARG02979	PARG02980	PARG02981	PARG02982	PARG02983	PARG02984
PARG02985	PARG02986	PARG02987	PARG02988	PARG02989	PARG02990	PARG02991
PARG02992	PARG02993	PARG02994	PARG02995	PARG02996	PARG02997	PARG02998

PARG02999	PARG03000	PARG03001	PARG03003	PARG03004	PARG03005	PARG03006
PARG03007	PARG03008	PARG03009	PARG03010	PARG03011	PARG03012	PARG03015
PARG03016	PARG03017	PARG03018	PARG03019	PARG03020	PARG03021	PARG03022
PARG03023	PARG03024	PARG03025	PARG03026	PARG03027	PARG03028	PARG03029
PARG03031	PARG03032	PARG03034	PARG03035	PARG03036	PARG03037	PARG03038
PARG03039	PARG03040	PARG03041	PARG03042	PARG03043	PARG03044	PARG03045
PARG03046	PARG03047	PARG03048	PARG03049	PARG03050	PARG03051	PARG03052
PARG03053	PARG03055	PARG03056	PARG03057	PARG03058	PARG03059	PARG03060
PARG03061	PARG03062	PARG03063	PARG03064	PARG03065	PARG03067	PARG03068
PARG03069	PARG03070	PARG03071	PARG03072	PARG03073	PARG03075	PARG03076
PARG03077	PARG03078	PARG03079	PARG03080	PARG03081	PARG03082	PARG03083
PARG03084	PARG03085	PARG03086	PARG03087	PARG03088	PARG03089	PARG03090
PARG03091	PARG03092	PARG03093	PARG03094	PARG03095	PARG03096	PARG03097
PARG03100	PARG03101	PARG03102	PARG03103	PARG03104	PARG03105	PARG03106
PARG03107	PARG03108	PARG03109	PARG03110	PARG03111	PARG03112	PARG03113
PARG03114	PARG03115	PARG03116	PARG03117	PARG03118	PARG03121	PARG03122
PARG03123	PARG03124	PARG03125	PARG03126	PARG03127	PARG03128	PARG03129
PARG03131	PARG03132	PARG03133	PARG03134	PARG03135	PARG03136	PARG03137
PARG03138	PARG03139	PARG03140	PARG03141	PARG03142	PARG03143	PARG03144
PARG03145	PARG03146	PARG03147	PARG03148	PARG03149	PARG03150	PARG03151
PARG03152	PARG03153	PARG03154	PARG03155	PARG03156	PARG03157	PARG03158
PARG03159	PARG03160	PARG03161	PARG03162	PARG03163	PARG03164	PARG03165
PARG03166	PARG03167	PARG03168	PARG03173	PARG03176	PARG03177	PARG03178
PARG03179	PARG03180	PARG03181	PARG03182	PARG03183	PARG03184	PARG03185
PARG03186	PARG03188	PARG03189	PARG03190	PARG03191	PARG03192	PARG03194
PARG03195	PARG03196	PARG03197	PARG03200	PARG03201	PARG03202	PARG03203
PARG03204	PARG03205	PARG03206	PARG03207	PARG03208	PARG03209	PARG03210
PARG03212	PARG03213	PARG03214	PARG03215	PARG03216	PARG03217	PARG03218
PARG03219	PARG03220	PARG03221	PARG03223	PARG03224	PARG03225	PARG03226
PARG03227	PARG03229	PARG03230	PARG03232	PARG03233	PARG03234	PARG03235
PARG03236	PARG03237	PARG03238	PARG03239	PARG03240	PARG03241	PARG03242
PARG03243	PARG03245	PARG03246	PARG03247	PARG03248	PARG03249	PARG03250
PARG03251	PARG03252	PARG03253	PARG03254	PARG03255	PARG03256	PARG03258
PARG03259	PARG03260	PARG03261	PARG03262	PARG03263	PARG03264	PARG03265
PARG03266	PARG03267	PARG03268	PARG03270	PARG03271	PARG03272	PARG03273
PARG03274	PARG03275	PARG03276	PARG03277	PARG03278	PARG03279	PARG03280
PARG03281	PARG03282	PARG03283	PARG03284	PARG03285	PARG03286	PARG03287
PARG03288	PARG03289	PARG03290	PARG03291	PARG03294	PARG03295	PARG03296
PARG03297	PARG03298	PARG03299	PARG03300	PARG03301	PARG03302	PARG03303
PARG03304	PARG03305	PARG03306	PARG03307	PARG03311	PARG03312	PARG03316
PARG03318	PARG03319	PARG03320	PARG03321	PARG03322	PARG03323	PARG03324
PARG03325	PARG03326	PARG03327	PARG03328	PARG03329	PARG03330	PARG03334
PARG03335	PARG03336	PARG03338	PARG03339	PARG03340	PARG03341	PARG03342
PARG03343	PARG03345	PARG03347	PARG03348	PARG03351	PARG03352	PARG03353
PARG03354	PARG03355	PARG03356	PARG03357	PARG03358	PARG03359	PARG03360
PARG03361	PARG03362	PARG03363	PARG03364	PARG03365	PARG03366	PARG03367

PARG03368	PARG03369	PARG03370	PARG03371	PARG03372	PARG03373	PARG03375
PARG03379	PARG03380	PARG03381	PARG03382	PARG03383	PARG03384	PARG03385
PARG03386	PARG03387	PARG03388	PARG03389	PARG03390	PARG03392	PARG03393
PARG03395	PARG03396	PARG03397	PARG03398	PARG03399	PARG03400	PARG03401
PARG03402	PARG03403	PARG03405	PARG03406	PARG03407	PARG03408	PARG03409
PARG03410	PARG03411	PARG03412	PARG03414	PARG03415	PARG03416	PARG03417
PARG03418	PARG03419	PARG03420	PARG03421	PARG03422	PARG03423	PARG03424
PARG03425	PARG03426	PARG03427	PARG03428	PARG03429	PARG03430	PARG03431
PARG03432	PARG03433	PARG03434	PARG03435	PARG03436	PARG03437	PARG03438
PARG03439	PARG03440	PARG03442	PARG03443	PARG03444	PARG03445	PARG03446
PARG03447	PARG03448	PARG03450	PARG03451	PARG03453	PARG03454	PARG03456
PARG03457	PARG03458	PARG03459	PARG03460	PARG03461	PARG03462	PARG03463
PARG03464	PARG03465	PARG03466	PARG03467	PARG03468	PARG03469	PARG03470
PARG03471	PARG03472	PARG03473	PARG03475	PARG03476	PARG03477	PARG03478
PARG03479	PARG03480	PARG03481	PARG03482	PARG03483	PARG03484	PARG03485
PARG03486	PARG03487	PARG03488	PARG03489	PARG03490	PARG03491	PARG03492
PARG03494	PARG03495	PARG03496	PARG03497	PARG03498	PARG03499	PARG03500
PARG03501	PARG03502	PARG03503	PARG03504	PARG03505	PARG03506	PARG03507
PARG03508	PARG03511	PARG03512	PARG03514	PARG03515	PARG03516	PARG03517
PARG03518	PARG03519	PARG03520	PARG03521	PARG03524	PARG03526	PARG03527
PARG03528	PARG03529	PARG03531	PARG03532	PARG03534	PARG03536	PARG03539
PARG03540	PARG03541	PARG03542	PARG03543	PARG03544	PARG03545	PARG03546
PARG03547	PARG03548	PARG03549	PARG03550	PARG03551	PARG03552	PARG03553
PARG03554	PARG03555	PARG03556	PARG03557	PARG03558	PARG03559	PARG03560
PARG03561	PARG03562	PARG03563	PARG03565	PARG03566	PARG03567	PARG03568
PARG03576	PARG03578	PARG03582	PARG03583	PARG03584	PARG03585	PARG03586
PARG03588	PARG03589	PARG03590	PARG03591	PARG03592	PARG03593	PARG03594
PARG03595	PARG03596	PARG03597	PARG03598	PARG03601	PARG03602	PARG03603
PARG03604	PARG03606	PARG03607	PARG03609	PARG03610	PARG03611	PARG03612
PARG03613	PARG03614	PARG03615	PARG03616	PARG03617	PARG03618	PARG03619
PARG03620	PARG03621	PARG03623	PARG03624	PARG03626	PARG03627	PARG03628
PARG03629	PARG03630	PARG03631	PARG03632	PARG03633	PARG03634	PARG03635
PARG03636	PARG03637	PARG03638	PARG03639	PARG03640	PARG03641	PARG03642
PARG03643	PARG03644	PARG03645	PARG03646	PARG03647	PARG03648	PARG03649
PARG03650	PARG03652	PARG03653	PARG03654	PARG03655	PARG03656	PARG03657
PARG03658	PARG03659	PARG03660	PARG03661	PARG03662	PARG03663	PARG03665
PARG03666	PARG03672	PARG03673	PARG03674	PARG03675	PARG03676	PARG03677
PARG03679	PARG03681	PARG03682	PARG03683	PARG03684	PARG03685	PARG03686
PARG03687	PARG03688	PARG03689	PARG03690	PARG03692	PARG03693	PARG03694
PARG03696	PARG03697	PARG03698	PARG03699	PARG03700	PARG03701	PARG03702
PARG03703	PARG03705	PARG03709	PARG03710	PARG03711	PARG03712	PARG03713
PARG03714	PARG03715	PARG03716	PARG03718	PARG03720	PARG03722	PARG03724
PARG03725	PARG03727	PARG03728	PARG03729	PARG03730	PARG03733	PARG03734
PARG03736	PARG03737	PARG03738	PARG03739	PARG03740	PARG03741	PARG03742
PARG03743	PARG03744	PARG03745	PARG03746	PARG03747	PARG03748	PARG03749
PARG03750	PARG03751	PARG03752	PARG03753	PARG03755	PARG03756	PARG03758

PARG03759	PARG03760	PARG03761	PARG03764	PARG03765	PARG03766	PARG03767
PARG03768	PARG03769	PARG03770	PARG03771	PARG03772	PARG03773	PARG03774
PARG03775	PARG03776	PARG03777	PARG03779	PARG03780	PARG03782	PARG03783
PARG03786	PARG03787	PARG03791	PARG03793	PARG03795	PARG03797	PARG03798
PARG03799	PARG03800	PARG03801	PARG03802	PARG03804	PARG03807	PARG03808
PARG03809	PARG03810	PARG03811	PARG03812	PARG03813	PARG03814	PARG03815
PARG03816	PARG03817	PARG03819	PARG03820	PARG03822	PARG03823	PARG03824
PARG03825	PARG03826	PARG03827	PARG03828	PARG03831	PARG03832	PARG03833
PARG03834	PARG03835	PARG03836	PARG03837	PARG03838	PARG03839	PARG03840
PARG03841	PARG03844	PARG03845	PARG03847	PARG03850	PARG03851	PARG03852
PARG03853	PARG03854	PARG03857	PARG03859	PARG03860	PARG03863	PARG03864
PARG03865	PARG03866	PARG03867	PARG03868	PARG03869	PARG03871	PARG03872
PARG03873	PARG03874	PARG03879	PARG03881	PARG03883	PARG03884	PARG03885
PARG03886	PARG03889	PARG03890	PARG03891	PARG03892	PARG03893	PARG03894
PARG03895	PARG03897	PARG03899	PARG03900	PARG03901	PARG03902	PARG03903
PARG03904	PARG03905	PARG03906	PARG03908	PARG03909	PARG03910	PARG03911
PARG03912	PARG03913	PARG03914	PARG03915	PARG03917	PARG03918	PARG03919
PARG03920	PARG03922	PARG03923	PARG03924	PARG03925	PARG03926	PARG03927
PARG03929	PARG03930	PARG03933	PARG03934	PARG03935	PARG03936	PARG03937
PARG03938	PARG03939	PARG03942	PARG03949	PARG03950	PARG03951	PARG03952
PARG03953	PARG03954	PARG03955	PARG03956	PARG03957	PARG03958	PARG03959
PARG03960	PARG03961	PARG03962	PARG03963	PARG03964	PARG03965	PARG03966
PARG03967	PARG03969	PARG03971	PARG03972	PARG03973	PARG03974	PARG03975
PARG03976	PARG03977	PARG03978	PARG03979	PARG03981	PARG03982	PARG03983
PARG03984	PARG03985	PARG03987	PARG03988	PARG03989	PARG03992	PARG03993
PARG03994	PARG03995	PARG03996	PARG03997	PARG03998	PARG03999	PARG04000
PARG04001	PARG04002	PARG04003	PARG04005	PARG04006	PARG04007	PARG04009
PARG04011	PARG04013	PARG04015	PARG04016	PARG04017	PARG04020	PARG04021
PARG04022	PARG04023	PARG04024	PARG04025	PARG04026	PARG04028	PARG04029
PARG04031	PARG04033	PARG04034	PARG04035	PARG04036	PARG04037	PARG04038
PARG04039	PARG04040	PARG04041	PARG04042	PARG04043	PARG04046	PARG04047
PARG04049	PARG04053	PARG04055	PARG04056	PARG04057	PARG04058	PARG04059
PARG04060	PARG04061	PARG04063	PARG04069	PARG04070	PARG04071	PARG04072
PARG04074	PARG04075	PARG04076	PARG04077	PARG04078	PARG04079	PARG04080
PARG04081	PARG04082	PARG04083	PARG04085	PARG04086	PARG04087	PARG04088
PARG04089	PARG04091	PARG04092	PARG04097	PARG04098	PARG04099	PARG04100
PARG04101	PARG04102	PARG04103	PARG04104	PARG04105	PARG04109	PARG04111
PARG04112	PARG04113	PARG04114	PARG04115	PARG04116	PARG04118	PARG04119
PARG04120	PARG04121	PARG04123	PARG04124	PARG04125	PARG04126	PARG04127
PARG04129	PARG04132	PARG04133	PARG04134	PARG04135	PARG04136	PARG04139
PARG04140	PARG04141	PARG04142	PARG04143	PARG04144	PARG04145	PARG04146
PARG04147	PARG04148	PARG04149	PARG04150	PARG04151	PARG04152	PARG04153
PARG04154	PARG04155	PARG04156	PARG04157	PARG04158	PARG04159	PARG04160
PARG04167	PARG04168	PARG04170	PARG04172	PARG04173	PARG04174	PARG04177
PARG04178	PARG04179	PARG04180	PARG04181	PARG04182	PARG04183	PARG04184
PARG04186	PARG04187	PARG04188	PARG04189	PARG04190	PARG04192	PARG04193

PARG04194	PARG04195	PARG04196	PARG04197	PARG04198	PARG04201	PARG04202
PARG04205	PARG04210	PARG04211	PARG04212	PARG04213	PARG04216	PARG04218
PARG04219	PARG04225	PARG04226	PARG04229	PARG04235	PARG04237	PARG04238
PARG04247	PARG04248	PARG04254	PARG04255	PARG04256	PARG04257	PARG04260
PARG04266	PARG04282	PARG04283	PARG04293	PARG04295	PARG04296	PARG04297
PARG04298	PARG04299	PARG04304	PARG04305	PARG04306	PARG04308	PARG04309
PARG04311	PARG04312	PARG04313	PARG04314	PARG04316	PARG04317	PARG04319
PARG04322	PARG04323	PARG04324	PARG04325	PARG04326	PARG04327	PARG04328
PARG04329	PARG04331	PARG04332	PARG04334	PARG04335	PARG04338	PARG04339
PARG04340	PARG04341	PARG04342	PARG04343	PARG04344	PARG04345	PARG04346
PARG04347	PARG04348	PARG04349	PARG04350	PARG04353	PARG04354	PARG04355
PARG04356	PARG04358	PARG04359	PARG04360	PARG04361	PARG04362	PARG04363
PARG04364	PARG04365	PARG04366	PARG04367	PARG04368	PARG04369	PARG04370
PARG04371	PARG04372	PARG04373	PARG04375	PARG04376	PARG04378	PARG04379
PARG04380	PARG04381	PARG04382	PARG04383	PARG04384	PARG04385	PARG04386
PARG04387	PARG04388	PARG04389	PARG04390	PARG04391	PARG04392	PARG04393
PARG04394	PARG04395	PARG04398	PARG04400	PARG04401	PARG04402	PARG04403
PARG04404	PARG04405	PARG04406	PARG04407	PARG04408	PARG04409	PARG04411
PARG04413	PARG04414	PARG04415	PARG04416	PARG04417	PARG04419	PARG04420
PARG04421	PARG04423	PARG04424	PARG04425	PARG04426	PARG04427	PARG04428
PARG04429	PARG04430	PARG04434	PARG04435	PARG04436	PARG04437	PARG04438
PARG04439	PARG04441	PARG04442	PARG04443	PARG04444	PARG04445	PARG04446
PARG04447	PARG04448	PARG04449	PARG04452	PARG04455	PARG04456	PARG04457
PARG04458	PARG04459	PARG04460	PARG04461	PARG04462	PARG04463	PARG04464
PARG04465	PARG04466	PARG04468	PARG04469	PARG04470	PARG04471	PARG04472
PARG04473	PARG04474	PARG04475	PARG04476	PARG04477	PARG04478	PARG04479
PARG04480	PARG04481	PARG04482	PARG04483	PARG04484	PARG04485	PARG04487
PARG04488	PARG04489	PARG04490	PARG04491	PARG04492	PARG04493	PARG04494
PARG04495	PARG04496	PARG04498	PARG04499	PARG04500	PARG04501	PARG04502
PARG04503	PARG04506	PARG04507	PARG04508	PARG04509	PARG04510	PARG04511
PARG04512	PARG04513	PARG04514	PARG04515	PARG04516	PARG04517	PARG04518
PARG04520	PARG04521	PARG04522	PARG04523	PARG04524	PARG04525	PARG04526
PARG04528	PARG04529	PARG04530	PARG04535	PARG04537	PARG04540	PARG04541
PARG04542	PARG04543	PARG04547	PARG04548	PARG04549	PARG04550	PARG04551
PARG04554	PARG04555	PARG04556	PARG04557	PARG04558	PARG04559	PARG04560
PARG04561	PARG04562	PARG04563	PARG04564	PARG04565	PARG04566	PARG04567
PARG04568	PARG04569	PARG04570	PARG04571	PARG04572	PARG04573	PARG04574
PARG04575	PARG04576	PARG04577	PARG04578	PARG04579	PARG04580	PARG04581
PARG04582	PARG04584	PARG04585	PARG04586	PARG04587	PARG04588	PARG04589
PARG04590	PARG04591	PARG04592	PARG04593	PARG04594	PARG04595	PARG04596
PARG04597	PARG04598	PARG04599	PARG04601	PARG04603	PARG04612	PARG04613
PARG04623	PARG04624	PARG04625	PARG04626	PARG04634	PARG04635	PARG04636
PARG04642	PARG04643	PARG04646	PARG04647	PARG04648	PARG04649	PARG04650
PARG04651	PARG04652	PARG04653	PARG04654	PARG04655	PARG04656	PARG04658
PARG04661	PARG04662	PARG04663	PARG04664	PARG04668	PARG04672	PARG04673
PARG04674	PARG04675	PARG04676	PARG04680	PARG04692	PARG04693	PARG04694

PARG04695	PARG04696	PARG04697	PARG04698	PARG04699	PARG04702	PARG04705
PARG04706	PARG04708	PARG04710	PARG04711	PARG04712	PARG04713	PARG04716
PARG04718	PARG04719	PARG04720	PARG04721	PARG04722	PARG04723	PARG04726
PARG04732	PARG04733	PARG04734	PARG04735	PARG04741	PARG04743	PARG04744
PARG04745	PARG04746	PARG04748	PARG04749	PARG04750	PARG04752	PARG04753
PARG04754	PARG04755	PARG04756	PARG04757	PARG04765	PARG04766	PARG04767
PARG04768	PARG04771	PARG04772	PARG04778	PARG04795	PARG04809	PARG04811
PARG04830	PARG04832	PARG04833	PARG04834	PARG04835	PARG04838	PARG04839
PARG04840	PARG04841	PARG04842	PARG04843	PARG04844	PARG04845	PARG04850
PARG04877	PARG04883	PARG04884	PARG04885	PARG04886	PARG04887	PARG04888
PARG04889	PARG04890	PARG04891	PARG04892	PARG04893	PARG04897	PARG04898
PARG04899	PARG04900	PARG04901	PARG04902	PARG04904	PARG04905	PARG04906
PARG04907	PARG04908	PARG04909	PARG04912	PARG04913	PARG04914	PARG04915
PARG04916	PARG04918	PARG04919	PARG04920	PARG04921	PARG04922	PARG04923
PARG04926	PARG04927	PARG04928	PARG04929	PARG04930	PARG04931	PARG04932
PARG04933	PARG04934	PARG04935	PARG04937	PARG04938	PARG04940	PARG04941
PARG04942	PARG04943	PARG04944	PARG04945	PARG04946	PARG04947	PARG04948
PARG04949	PARG04962	PARG04963	PARG04967	PARG04968	PARG04969	PARG04970
PARG04972	PARG04973	PARG04975	PARG04976	PARG04985	PARG04987	PARG04989
PARG04990	PARG04992	PARG04998	PARG05001	PARG05002	PARG05006	PARG05007
PARG05008	PARG05012	PARG05014	PARG05022	PARG05025	PARG05026	PARG05030
PARG05032	PARG05033	PARG05037	PARG05038	PARG05039	PARG05041	PARG05042
PARG05044	PARG05048	PARG05051	PARG05052	PARG05055	PARG05058	PARG05059
PARG05066	PARG05067	PARG05071	PARG05072	PARG05073	PARG05074	PARG05075
PARG05076	PARG05077	PARG05080	PARG05083	PARG05088	PARG05089	PARG05090
PARG05092	PARG05095	PARG05096	PARG05097	PARG05098	PARG05099	PARG05103
PARG05104	PARG05106	PARG05108	PARG05109	PARG05110	PARG05111	PARG05112
PARG05115	PARG05116	PARG05118	PARG05120	PARG05121	PARG05122	PARG05124
PARG05125	PARG05126	PARG05127	PARG05129	PARG05133	PARG05134	PARG05135
PARG05137	PARG05139	PARG05140	PARG05144	PARG05146	PARG05147	PARG05149
PARG05150	PARG05151	PARG05152	PARG05153	PARG05155	PARG05156	PARG05167
PARG05168	PARG05170	PARG05171	PARG05178	PARG05179	PARG05180	PARG05181
PARG05183	PARG05184	PARG05189	PARG05190	PARG05198	PARG05199	PARG05200
PARG05207	PARG05208	PARG05209	PARG05210	PARG05211	PARG05212	PARG05213
PARG05214	PARG05217	PARG05218	PARG05219	PARG05220	PARG05221	PARG05222
PARG05223	PARG05227	PARG05231	PARG05235	PARG05236	PARG05237	PARG05243
PARG05244	PARG05245	PARG05246	PARG05249	PARG05250	PARG05251	PARG05252
PARG05253	PARG05256	PARG05257	PARG05258	PARG05259	PARG05260	PARG05261
PARG05266	PARG05267	PARG05268	PARG05269	PARG05271	PARG05272	PARG05273
PARG05274	PARG05276	PARG05283	PARG05284	PARG05286	PARG05287	PARG05288
PARG05289	PARG05293	PARG05294	PARG05295	PARG05299	PARG05302	PARG05303
PARG05309	PARG05311	PARG05314	PARG05316	PARG05317	PARG05318	PARG05319
PARG05320	PARG05323	PARG05324	PARG05325	PARG05330	PARG05331	PARG05332
PARG05333	PARG05334	PARG05336	PARG05339	PARG05340	PARG05343	PARG05344
PARG05345	PARG05348	PARG05350	PARG05351	PARG05352	PARG05353	PARG05354
PARG05355	PARG05356	PARG05357	PARG05368	PARG05370	PARG05371	PARG05374

PARG05381	PARG05382	PARG05383	PARG05387	PARG05388	PARG05389	PARG05390
PARG05391	PARG05392	PARG05397	PARG05401	PARG05407	PARG05411	PARG05416
PARG05417	PARG05419	PARG05420	PARG05421	PARG05425	PARG05426	PARG05427
PARG05428	PARG05429	PARG05430	PARG05431	PARG05432	PARG05433	PARG05435
PARG05436	PARG05437	PARG05440	PARG05441	PARG05442	PARG05443	PARG05444
PARG05445	PARG05446	PARG05447	PARG05448	PARG05449	PARG05450	PARG05451
PARG05452	PARG05453	PARG05457	PARG05458	PARG05459	PARG05460	PARG05461
PARG05462	PARG05468	PARG05469	PARG05471	PARG05472	PARG05473	PARG05474
PARG05475	PARG05476	PARG05477	PARG05478	PARG05479	PARG05480	PARG05481
PARG05483	PARG05484	PARG05485	PARG05487	PARG05488	PARG05489	PARG05490
PARG05491	PARG05492	PARG05494	PARG05495	PARG05496	PARG05497	PARG05499
PARG05500	PARG05501	PARG05503	PARG05504	PARG05505	PARG05506	PARG05507
PARG05508	PARG05509	PARG05510	PARG05511	PARG05512	PARG05513	PARG05514
PARG05515	PARG05516	PARG05519	PARG05520	PARG05521	PARG05523	PARG05524
PARG05525	PARG05526	PARG05527	PARG05528	PARG05531	PARG05532	PARG05533
PARG05534	PARG05535	PARG05536	PARG05537	PARG05538	PARG05539	PARG05540
PARG05541	PARG05542	PARG05543	PARG05544	PARG05545	PARG05546	PARG05547
PARG05548	PARG05549	PARG05552	PARG05553	PARG05554	PARG05555	PARG05556
PARG05557	PARG05558	PARG05559	PARG05560	PARG05561	PARG05562	PARG05563
PARG05565	PARG05566	PARG05567	PARG05568	PARG05569	PARG05570	PARG05572
PARG05573	PARG05574	PARG05575	PARG05576	PARG05577	PARG05578	PARG05579
PARG05580	PARG05581	PARG05582	PARG05585	PARG05587	PARG05588	PARG05589
PARG05590	PARG05592	PARG05593	PARG05594	PARG05595	PARG05596	PARG05604
PARG05605	PARG05607	PARG05608	PARG05609	PARG05610	PARG05611	PARG05616
PARG05618	PARG05619	PARG05620	PARG05621	PARG05622	PARG05623	PARG05624
PARG05626	PARG05628	PARG05629	PARG05630	PARG05631	PARG05632	PARG05633
PARG05634	PARG05635	PARG05636	PARG05637	PARG05638	PARG05640	PARG05641
PARG05642	PARG05643	PARG05645	PARG05646	PARG05647	PARG05651	PARG05652
PARG05654	PARG05658	PARG05659	PARG05660	PARG05661	PARG05662	PARG05663
PARG05664	PARG05665	PARG05666	PARG05667	PARG05669	PARG05670	PARG05671
PARG05672	PARG05675	PARG05678	PARG05679	PARG05680	PARG05681	PARG05682
PARG05683	PARG05684	PARG05686	PARG05688	PARG05690	PARG05692	PARG05693
PARG05694	PARG05695	PARG05696	PARG05697	PARG05698	PARG05699	PARG05700
PARG05701	PARG05702	PARG05709	PARG05710	PARG05711	PARG05712	PARG05715
PARG05716	PARG05718	PARG05719	PARG05720	PARG05721	PARG05722	PARG05723
PARG05724	PARG05725	PARG05726	PARG05727	PARG05728	PARG05729	PARG05735
PARG05736	PARG05738	PARG05739	PARG05740	PARG05741	PARG05743	PARG05744
PARG05745	PARG05746	PARG05747	PARG05748	PARG05749	PARG05751	PARG05752
PARG05753	PARG05754	PARG05755	PARG05756	PARG05757	PARG05758	PARG05759
PARG05760	PARG05762	PARG05763	PARG05765	PARG05766	PARG05767	PARG05768
PARG05769	PARG05770	PARG05771	PARG05772	PARG05773	PARG05774	PARG05775
PARG05776	PARG05777	PARG05778	PARG05779	PARG05780	PARG05781	PARG05782
PARG05783	PARG05784	PARG05785	PARG05786	PARG05788	PARG05789	PARG05791
PARG05792	PARG05793	PARG05794	PARG05795	PARG05798	PARG05799	PARG05800
PARG05801	PARG05802	PARG05803	PARG05805	PARG05806	PARG05807	PARG05808
PARG05809	PARG05810	PARG05811	PARG05813	PARG05814	PARG05815	PARG05816

PARG05817	PARG05818	PARG05819	PARG05820	PARG05821	PARG05822	PARG05823
PARG05824	PARG05825	PARG05826	PARG05827	PARG05828	PARG05829	PARG05830
PARG05831	PARG05832	PARG05841	PARG05842	PARG05843	PARG05845	PARG05846
PARG05847	PARG05848	PARG05849	PARG05850	PARG05851	PARG05852	PARG05854
PARG05856	PARG05857	PARG05858	PARG05861	PARG05863	PARG05864	PARG05865
PARG05866	PARG05871	PARG05874	PARG05875	PARG05876	PARG05878	PARG05879
PARG05880	PARG05881	PARG05882	PARG05883	PARG05884	PARG05885	PARG05886
PARG05887	PARG05888	PARG05889	PARG05890	PARG05891	PARG05892	PARG05893
PARG05894	PARG05895	PARG05897	PARG05898	PARG05899	PARG05900	PARG05902
PARG05903	PARG05904	PARG05905	PARG05906	PARG05907	PARG05908	PARG05909
PARG05910	PARG05911	PARG05912	PARG05913	PARG05914	PARG05915	PARG05925
PARG05927	PARG05928	PARG05929	PARG05937	PARG05938	PARG05939	PARG05942
PARG05945	PARG05947	PARG05948	PARG05950	PARG05952	PARG05959	PARG05960
PARG05961	PARG05963	PARG05964	PARG05965	PARG05967	PARG05969	PARG05973
PARG05975	PARG05976	PARG05977	PARG05978	PARG05980	PARG05981	PARG05982
PARG05983	PARG05984	PARG05985	PARG05986	PARG05987	PARG05988	PARG05989
PARG05990	PARG05991	PARG05992	PARG05993	PARG05994	PARG05995	PARG05996
PARG05997	PARG05998	PARG05999	PARG06002	PARG06003	PARG06004	PARG06005
PARG06006	PARG06007	PARG06008	PARG06009	PARG06010	PARG06011	PARG06012
PARG06013	PARG06015	PARG06016	PARG06017	PARG06018	PARG06019	PARG06020
PARG06021	PARG06022	PARG06025	PARG06027	PARG06028	PARG06029	PARG06031
PARG06032	PARG06033	PARG06034	PARG06036	PARG06037	PARG06038	PARG06039
PARG06040	PARG06041	PARG06042	PARG06045	PARG06046	PARG06047	PARG06049
PARG06050	PARG06051	PARG06052	PARG06053	PARG06054	PARG06055	PARG06056
PARG06057	PARG06058	PARG06059	PARG06060	PARG06061	PARG06062	PARG06063
PARG06064	PARG06065	PARG06066	PARG06067	PARG06068	PARG06069	PARG06070
PARG06071	PARG06072	PARG06073	PARG06074	PARG06076	PARG06077	PARG06078
PARG06079	PARG06080	PARG06081	PARG06082	PARG06083	PARG06084	PARG06085
PARG06086	PARG06087	PARG06088	PARG06089	PARG06090	PARG06092	PARG06093
PARG06094	PARG06095	PARG06096	PARG06098	PARG06099	PARG06100	PARG06101
PARG06102	PARG06104	PARG06105	PARG06106	PARG06107	PARG06108	PARG06109
PARG06110	PARG06111	PARG06112	PARG06113	PARG06114	PARG06115	PARG06116
PARG06118	PARG06119	PARG06120	PARG06121	PARG06122	PARG06123	PARG06124
PARG06125	PARG06126	PARG06127	PARG06128	PARG06129	PARG06130	PARG06131
PARG06132	PARG06133	PARG06134	PARG06135	PARG06139	PARG06140	PARG06141
PARG06142	PARG06143	PARG06145	PARG06146	PARG06147	PARG06148	PARG06149
PARG06150	PARG06151	PARG06152	PARG06153	PARG06154	PARG06155	PARG06156
PARG06157	PARG06158	PARG06160	PARG06161	PARG06162	PARG06163	PARG06164
PARG06165	PARG06166	PARG06169	PARG06171	PARG06172	PARG06173	PARG06174
PARG06175	PARG06177	PARG06178	PARG06179	PARG06182	PARG06183	PARG06184
PARG06186	PARG06190	PARG06193	PARG06194	PARG06195	PARG06196	PARG06197
PARG06198	PARG06199	PARG06200	PARG06202	PARG06203	PARG06204	PARG06205
PARG06206	PARG06207	PARG06208	PARG06209	PARG06210	PARG06211	PARG06212
PARG06213	PARG06215	PARG06216	PARG06217	PARG06219	PARG06220	PARG06222
PARG06225	PARG06226	PARG06227	PARG06228	PARG06229	PARG06230	PARG06231
PARG06232	PARG06233	PARG06234	PARG06235	PARG06236	PARG06237	PARG06238

PARG06239	PARG06240	PARG06241	PARG06242	PARG06243	PARG06244	PARG06245
PARG06246	PARG06247	PARG06248	PARG06249	PARG06250	PARG06251	PARG06254
PARG06257	PARG06262	PARG06263	PARG06264	PARG06265	PARG06266	PARG06267
PARG06269	PARG06270	PARG06271	PARG06272	PARG06274	PARG06275	PARG06281
PARG06282	PARG06283	PARG06284	PARG06285	PARG06286	PARG06287	PARG06288
PARG06289	PARG06290	PARG06291	PARG06292	PARG06294	PARG06296	PARG06297
PARG06301	PARG06303	PARG06304	PARG06305	PARG06306	PARG06307	PARG06308
PARG06309	PARG06310	PARG06311	PARG06312	PARG06313	PARG06315	PARG06316
PARG06317	PARG06318	PARG06319	PARG06320	PARG06321	PARG06322	PARG06323
PARG06324	PARG06325	PARG06326	PARG06327	PARG06328	PARG06329	PARG06330
PARG06331	PARG06332	PARG06333	PARG06337	PARG06338	PARG06339	PARG06340
PARG06341	PARG06342	PARG06343	PARG06344	PARG06345	PARG06346	PARG06347
PARG06348	PARG06349	PARG06350	PARG06351	PARG06352	PARG06353	PARG06354
PARG06357	PARG06358	PARG06360	PARG06361	PARG06362	PARG06363	PARG06365
PARG06366	PARG06367	PARG06368	PARG06370	PARG06371	PARG06372	PARG06374
PARG06375	PARG06376	PARG06378	PARG06379	PARG06380	PARG06381	PARG06382
PARG06383	PARG06384	PARG06386	PARG06387	PARG06388	PARG06389	PARG06390
PARG06391	PARG06393	PARG06394	PARG06395	PARG06396	PARG06397	PARG06398
PARG06399	PARG06400	PARG06401	PARG06403	PARG06404	PARG06405	PARG06406
PARG06407	PARG06408	PARG06409	PARG06410	PARG06413	PARG06414	PARG06415
PARG06416	PARG06417	PARG06418	PARG06419	PARG06420	PARG06422	PARG06423
PARG06424	PARG06425	PARG06426	PARG06427	PARG06428	PARG06429	PARG06432
PARG06433	PARG06434	PARG06435	PARG06436	PARG06441	PARG06442	PARG06443
PARG06444	PARG06449	PARG06450	PARG06453	PARG06454	PARG06455	PARG06456
PARG06458	PARG06459	PARG06460	PARG06461	PARG06462	PARG06463	PARG06464
PARG06465	PARG06466	PARG06467	PARG06468	PARG06469	PARG06470	PARG06471
PARG06472	PARG06473	PARG06474	PARG06475	PARG06476	PARG06477	PARG06478
PARG06479	PARG06480	PARG06481	PARG06483	PARG06484	PARG06485	PARG06486
PARG06487	PARG06488	PARG06489	PARG06490	PARG06491	PARG06492	PARG06495
PARG06496	PARG06497	PARG06498	PARG06499	PARG06500	PARG06501	PARG06502
PARG06503	PARG06504	PARG06506	PARG06507	PARG06508	PARG06509	PARG06510
PARG06511	PARG06512	PARG06513	PARG06514	PARG06515	PARG06516	PARG06517
PARG06519	PARG06520	PARG06521	PARG06522	PARG06523	PARG06524	PARG06525
PARG06526	PARG06527	PARG06528	PARG06529	PARG06530	PARG06531	PARG06532
PARG06533	PARG06534	PARG06535	PARG06537	PARG06538	PARG06539	PARG06540
PARG06541	PARG06542	PARG06543	PARG06544	PARG06545	PARG06546	PARG06547
PARG06549	PARG06550	PARG06551	PARG06555	PARG06556	PARG06557	PARG06558
PARG06560	PARG06561	PARG06562	PARG06563	PARG06564	PARG06565	PARG06566
PARG06567	PARG06568	PARG06569	PARG06570	PARG06571	PARG06572	PARG06573
PARG06574	PARG06575	PARG06576	PARG06578	PARG06579	PARG06580	PARG06581
PARG06582	PARG06583	PARG06584	PARG06585	PARG06586	PARG06587	PARG06588
PARG06589	PARG06590	PARG06591	PARG06592	PARG06593	PARG06594	PARG06596
PARG06597	PARG06598	PARG06599	PARG06600	PARG06606	PARG06607	PARG06609
PARG06610	PARG06611	PARG06612	PARG06613	PARG06614	PARG06615	PARG06621
PARG06622	PARG06623	PARG06624	PARG06625	PARG06627	PARG06628	PARG06629
PARG06630	PARG06631	PARG06632	PARG06633	PARG06634	PARG06636	PARG06637

PARG06638	PARG06639	PARG06640	PARG06642	PARG06643	PARG06644	PARG06645
PARG06646	PARG06647	PARG06648	PARG06649	PARG06650	PARG06651	PARG06652
PARG06653	PARG06654	PARG06655	PARG06656	PARG06657	PARG06658	PARG06659
PARG06660	PARG06661	PARG06662	PARG06663	PARG06664	PARG06667	PARG06669
PARG06670	PARG06671	PARG06672	PARG06673	PARG06675	PARG06676	PARG06677
PARG06678	PARG06679	PARG06680	PARG06681	PARG06682	PARG06683	PARG06684
PARG06685	PARG06686	PARG06687	PARG06688	PARG06689	PARG06690	PARG06691
PARG06692	PARG06693	PARG06694	PARG06695	PARG06696	PARG06697	PARG06698
PARG06699	PARG06700	PARG06701	PARG06702	PARG06703	PARG06704	PARG06705
PARG06706	PARG06707	PARG06708	PARG06711	PARG06712	PARG06713	PARG06714
PARG06715	PARG06716	PARG06717	PARG06719	PARG06720	PARG06721	PARG06722
PARG06724	PARG06725	PARG06726	PARG06727	PARG06728	PARG06729	PARG06730
PARG06731	PARG06732	PARG06737	PARG06739	PARG06740	PARG06741	PARG06742
PARG06744	PARG06745	PARG06746	PARG06747	PARG06748	PARG06749	PARG06750
PARG06751	PARG06752	PARG06755	PARG06756	PARG06757	PARG06758	PARG06759
PARG06760	PARG06761	PARG06764	PARG06765	PARG06766	PARG06767	PARG06769
PARG06770	PARG06771	PARG06772	PARG06773	PARG06774	PARG06775	PARG06776
PARG06777	PARG06778	PARG06779	PARG06780	PARG06781	PARG06782	PARG06783
PARG06785	PARG06786	PARG06788	PARG06789	PARG06790	PARG06791	PARG06792
PARG06793	PARG06794	PARG06795	PARG06796	PARG06797	PARG06798	PARG06799
PARG06800	PARG06801	PARG06802	PARG06803	PARG06804	PARG06807	PARG06808
PARG06809	PARG06810	PARG06811	PARG06812	PARG06813	PARG06814	PARG06816
PARG06817	PARG06818	PARG06819	PARG06820	PARG06821	PARG06822	PARG06824
PARG06825	PARG06826	PARG06827	PARG06828	PARG06831	PARG06832	PARG06833
PARG06834	PARG06835	PARG06836	PARG06837	PARG06838	PARG06839	PARG06840
PARG06841	PARG06842	PARG06843	PARG06844	PARG06845	PARG06846	PARG06847
PARG06848	PARG06849	PARG06850	PARG06851	PARG06852	PARG06854	PARG06855
PARG06856	PARG06857	PARG06858	PARG06859	PARG06860	PARG06861	PARG06862
PARG06863	PARG06864	PARG06865	PARG06866	PARG06867	PARG06868	PARG06869
PARG06870	PARG06871	PARG06872	PARG06873	PARG06874	PARG06875	PARG06876
PARG06877	PARG06878	PARG06879	PARG06880	PARG06881	PARG06882	PARG06883
PARG06884	PARG06885	PARG06886	PARG06887	PARG06888	PARG06889	PARG06890
PARG06891	PARG06892	PARG06893	PARG06895	PARG06896	PARG06897	PARG06899
PARG06900	PARG06901	PARG06902	PARG06903	PARG06905	PARG06906	PARG06907
PARG06908	PARG06909	PARG06910	PARG06911	PARG06912	PARG06913	PARG06914
PARG06915	PARG06916	PARG06917	PARG06918	PARG06919	PARG06920	PARG06922
PARG06923	PARG06924	PARG06925	PARG06927	PARG06928	PARG06929	PARG06930
PARG06931	PARG06932	PARG06933	PARG06934	PARG06935	PARG06936	PARG06937
PARG06938	PARG06939	PARG06940	PARG06941	PARG06942	PARG06943	PARG06944
PARG06945	PARG06946	PARG06947	PARG06948	PARG06949	PARG06950	PARG06951
PARG06952	PARG06953	PARG06954	PARG06955	PARG06956	PARG06957	PARG06958
PARG06959	PARG06961	PARG06962	PARG06963	PARG06964	PARG06965	PARG06966
PARG06967	PARG06968	PARG06969	PARG06970	PARG06971	PARG06972	PARG06973
PARG06974	PARG06975	PARG06976	PARG06977	PARG06978	PARG06979	PARG06980
PARG06981	PARG06982	PARG06983	PARG06984	PARG06985	PARG06986	PARG06987
PARG06988	PARG06989	PARG06990	PARG06991	PARG06992	PARG06993	PARG06994

PARG06995	PARG06996	PARG06997	PARG06998	PARG06999	PARG07000	PARG07001
PARG07002	PARG07003	PARG07004	PARG07005	PARG07006	PARG07007	PARG07008
PARG07009	PARG07010	PARG07011	PARG07012	PARG07013	PARG07014	PARG07015
PARG07016	PARG07017	PARG07018	PARG07019	PARG07020	PARG07021	PARG07022
PARG07023	PARG07024	PARG07025	PARG07026	PARG07027	PARG07028	PARG07029
PARG07030	PARG07031	PARG07032	PARG07033	PARG07034	PARG07035	PARG07036
PARG07037	PARG07038	PARG07039	PARG07040	PARG07041	PARG07042	PARG07043
PARG07044	PARG07046	PARG07047	PARG07048	PARG07049	PARG07050	PARG07051
PARG07052	PARG07053	PARG07054	PARG07055	PARG07056	PARG07057	PARG07058
PARG07059	PARG07060	PARG07061	PARG07062	PARG07063	PARG07064	PARG07065
PARG07066	PARG07067	PARG07068	PARG07069	PARG07071	PARG07072	PARG07073
PARG07074	PARG07076	PARG07077	PARG07078	PARG07079	PARG07080	PARG07081
PARG07082	PARG07083	PARG07084	PARG07085	PARG07086	PARG07087	PARG07088
PARG07089	PARG07092	PARG07093	PARG07094	PARG07095	PARG07096	PARG07097
PARG07098	PARG07099	PARG07100	PARG07101	PARG07102	PARG07103	PARG07104
PARG07105	PARG07106	PARG07107	PARG07108	PARG07109	PARG07110	PARG07111
PARG07112	PARG07113	PARG07114	PARG07115	PARG07116	PARG07117	PARG07118
PARG07119	PARG07120	PARG07121	PARG07122	PARG07123	PARG07124	PARG07125
PARG07126	PARG07128	PARG07130	PARG07131	PARG07132	PARG07134	PARG07135
PARG07136	PARG07137	PARG07138	PARG07139	PARG07140	PARG07141	PARG07142
PARG07143	PARG07144	PARG07145	PARG07146	PARG07147	PARG07149	PARG07150
PARG07151	PARG07152	PARG07153	PARG07154	PARG07155	PARG07156	PARG07157
PARG07158	PARG07159	PARG07160	PARG07161	PARG07162	PARG07163	PARG07164
PARG07165	PARG07166	PARG07167	PARG07168	PARG07169	PARG07170	PARG07171
PARG07172	PARG07173	PARG07174	PARG07175	PARG07176	PARG07177	PARG07178
PARG07179	PARG07180	PARG07181	PARG07182	PARG07183	PARG07184	PARG07185
PARG07186	PARG07187	PARG07188	PARG07189	PARG07190	PARG07191	PARG07196
PARG07197	PARG07198	PARG07199	PARG07200	PARG07201	PARG07202	PARG07204
PARG07205	PARG07206	PARG07207	PARG07208	PARG07209	PARG07210	PARG07211
PARG07212	PARG07213	PARG07214	PARG07215	PARG07216	PARG07217	PARG07218
PARG07219	PARG07220	PARG07221	PARG07226	PARG07227	PARG07228	PARG07229
PARG07231	PARG07232	PARG07233	PARG07234	PARG07235	PARG07236	PARG07238
PARG07240	PARG07241	PARG07242	PARG07243	PARG07244	PARG07246	PARG07247
PARG07248	PARG07249	PARG07250	PARG07251	PARG07252	PARG07253	PARG07254
PARG07256	PARG07257	PARG07258	PARG07259	PARG07260	PARG07261	PARG07262
PARG07263	PARG07264	PARG07265	PARG07266	PARG07267	PARG07270	PARG07271
PARG07272	PARG07273	PARG07274	PARG07275	PARG07276	PARG07277	PARG07278
PARG07279	PARG07280	PARG07281	PARG07282	PARG07283	PARG07284	PARG07286
PARG07287	PARG07288	PARG07289	PARG07290	PARG07291	PARG07292	PARG07293
PARG07294	PARG07295	PARG07296	PARG07297	PARG07298	PARG07299	PARG07300
PARG07301	PARG07302	PARG07303	PARG07304	PARG07305	PARG07306	PARG07307
PARG07308	PARG07309	PARG07310	PARG07311	PARG07312	PARG07313	PARG07314
PARG07315	PARG07316	PARG07317	PARG07318	PARG07319	PARG07320	PARG07321
PARG07322	PARG07323	PARG07325	PARG07326	PARG07327	PARG07328	PARG07329
PARG07330	PARG07331	PARG07332	PARG07333	PARG07334	PARG07335	PARG07336
PARG07337	PARG07338	PARG07339	PARG07340	PARG07341	PARG07343	PARG07344

PARG07345	PARG07346	PARG07347	PARG07351	PARG07352	PARG07353	PARG07354
PARG07355	PARG07356	PARG07357	PARG07358	PARG07359	PARG07360	PARG07365
PARG07366	PARG07367	PARG07368	PARG07369	PARG07370	PARG07371	PARG07372
PARG07373	PARG07374	PARG07375	PARG07376	PARG07377	PARG07378	PARG07380
PARG07381	PARG07383	PARG07384	PARG07385	PARG07386	PARG07387	PARG07388
PARG07389	PARG07390	PARG07392	PARG07393	PARG07395	PARG07396	PARG07397
PARG07398	PARG07399	PARG07400	PARG07401	PARG07402	PARG07403	PARG07404
PARG07405	PARG07406	PARG07407	PARG07408	PARG07409	PARG07410	PARG07411
PARG07412	PARG07413	PARG07414	PARG07415	PARG07416	PARG07417	PARG07418
PARG07419	PARG07420	PARG07421	PARG07422	PARG07423	PARG07424	PARG07425
PARG07427	PARG07429	PARG07430	PARG07431	PARG07432	PARG07433	PARG07436
PARG07437	PARG07438	PARG07439	PARG07440	PARG07441	PARG07442	PARG07443
PARG07444	PARG07445	PARG07446	PARG07447	PARG07448	PARG07449	PARG07450
PARG07451	PARG07452	PARG07453	PARG07454	PARG07455	PARG07456	PARG07457
PARG07458	PARG07459	PARG07461	PARG07462	PARG07463	PARG07465	PARG07466
PARG07467	PARG07468	PARG07469	PARG07471	PARG07472	PARG07475	PARG07476
PARG07477	PARG07478	PARG07479	PARG07480	PARG07481	PARG07482	PARG07483
PARG07484	PARG07485	PARG07486	PARG07487	PARG07490	PARG07491	PARG07492
PARG07493	PARG07494	PARG07496	PARG07497	PARG07498	PARG07499	PARG07500
PARG07501	PARG07502	PARG07503	PARG07504	PARG07505	PARG07506	PARG07507
PARG07508	PARG07510	PARG07512	PARG07513	PARG07514	PARG07516	PARG07517
PARG07518	PARG07519	PARG07520	PARG07521	PARG07522	PARG07523	PARG07524
PARG07525	PARG07526	PARG07527	PARG07528	PARG07529	PARG07531	PARG07534
PARG07535	PARG07536	PARG07537	PARG07538	PARG07540	PARG07541	PARG07542
PARG07543	PARG07544	PARG07545	PARG07546	PARG07547	PARG07548	PARG07549
PARG07550	PARG07551	PARG07552	PARG07553	PARG07554	PARG07555	PARG07556
PARG07558	PARG07559	PARG07560	PARG07562	PARG07563	PARG07565	PARG07566
PARG07567	PARG07568	PARG07569	PARG07570	PARG07571	PARG07572	PARG07573
PARG07574	PARG07575	PARG07577	PARG07578	PARG07579	PARG07580	PARG07581
PARG07582	PARG07583	PARG07585	PARG07586	PARG07587	PARG07588	PARG07589
PARG07590	PARG07591	PARG07592	PARG07593	PARG07594	PARG07596	PARG07597
PARG07598	PARG07599	PARG07600	PARG07601	PARG07602	PARG07603	PARG07604
PARG07605	PARG07606	PARG07607	PARG07608	PARG07609	PARG07610	PARG07611
PARG07612	PARG07613	PARG07614	PARG07615	PARG07616	PARG07617	PARG07618
PARG07619	PARG07620	PARG07623	PARG07624	PARG07625	PARG07626	PARG07627
PARG07628	PARG07629	PARG07630	PARG07631	PARG07632	PARG07634	PARG07635
PARG07636	PARG07637	PARG07638	PARG07639	PARG07640	PARG07641	PARG07642
PARG07643	PARG07644	PARG07645	PARG07646	PARG07647	PARG07649	PARG07650
PARG07651	PARG07652	PARG07653	PARG07654	PARG07655	PARG07657	PARG07658
PARG07659	PARG07660	PARG07661	PARG07662	PARG07663	PARG07664	PARG07665
PARG07669	PARG07670	PARG07671	PARG07672	PARG07673	PARG07675	PARG07676
PARG07677	PARG07678	PARG07679	PARG07680	PARG07681	PARG07682	PARG07683
PARG07684	PARG07685	PARG07686	PARG07687	PARG07689	PARG07690	PARG07691
PARG07692	PARG07693	PARG07694	PARG07696	PARG07697	PARG07698	PARG07699
PARG07700	PARG07701	PARG07702	PARG07703	PARG07704	PARG07705	PARG07706
PARG07707	PARG07708	PARG07709	PARG07710	PARG07711	PARG07712	PARG07713

PARG07714	PARG07715	PARG07718	PARG07719	PARG07720	PARG07721	PARG07724
PARG07725	PARG07726	PARG07727	PARG07728	PARG07729	PARG07731	PARG07732
PARG07734	PARG07737	PARG07739	PARG07740	PARG07741	PARG07742	PARG07743
PARG07744	PARG07745	PARG07746	PARG07748	PARG07749	PARG07750	PARG07751
PARG07752	PARG07753	PARG07754	PARG07755	PARG07757	PARG07758	PARG07759
PARG07760	PARG07761	PARG07762	PARG07763	PARG07764	PARG07765	PARG07766
PARG07767	PARG07768	PARG07769	PARG07770	PARG07771	PARG07772	PARG07773
PARG07774	PARG07776	PARG07778	PARG07779	PARG07780	PARG07781	PARG07782
PARG07783	PARG07784	PARG07785	PARG07786	PARG07787	PARG07788	PARG07789
PARG07790	PARG07793	PARG07794	PARG07795	PARG07796	PARG07797	PARG07798
PARG07799	PARG07800	PARG07801	PARG07802	PARG07803	PARG07804	PARG07805
PARG07806	PARG07808	PARG07809	PARG07810	PARG07812	PARG07813	PARG07814
PARG07815	PARG07816	PARG07817	PARG07818	PARG07819	PARG07820	PARG07821
PARG07822	PARG07823	PARG07824	PARG07825	PARG07826	PARG07827	PARG07828
PARG07829	PARG07830	PARG07831	PARG07832	PARG07833	PARG07834	PARG07835
PARG07836	PARG07837	PARG07838	PARG07839	PARG07840	PARG07841	PARG07842
PARG07843	PARG07844	PARG07845	PARG07847	PARG07848	PARG07849	PARG07850
PARG07851	PARG07852	PARG07853	PARG07854	PARG07855	PARG07856	PARG07857
PARG07858	PARG07860	PARG07863	PARG07864	PARG07865	PARG07866	PARG07867
PARG07868	PARG07869	PARG07870	PARG07872	PARG07873	PARG07874	PARG07876
PARG07877	PARG07878	PARG07880	PARG07881	PARG07882	PARG07883	PARG07884
PARG07885	PARG07886	PARG07887	PARG07888	PARG07889	PARG07890	PARG07891
PARG07894	PARG07895	PARG07896	PARG07898	PARG07899	PARG07900	PARG07901
PARG07902	PARG07903	PARG07904	PARG07905	PARG07906	PARG07907	PARG07908
PARG07909	PARG07910	PARG07912	PARG07913	PARG07914	PARG07915	PARG07917
PARG07918	PARG07919	PARG07920	PARG07921	PARG07922	PARG07923	PARG07924
PARG07925	PARG07926	PARG07927	PARG07928	PARG07929	PARG07930	PARG07931
PARG07932	PARG07933	PARG07934	PARG07935	PARG07936	PARG07937	PARG07938
PARG07939	PARG07940	PARG07942	PARG07943	PARG07944	PARG07945	PARG07946
PARG07947	PARG07948	PARG07949	PARG07950	PARG07951	PARG07952	PARG07953
PARG07954	PARG07955	PARG07956	PARG07957	PARG07958	PARG07959	PARG07960
PARG07962	PARG07963	PARG07964	PARG07965	PARG07966	PARG07967	PARG07968
PARG07969	PARG07970	PARG07971	PARG07972	PARG07973	PARG07974	PARG07975
PARG07976	PARG07977	PARG07978	PARG07979	PARG07980	PARG07981	PARG07982
PARG07983	PARG07987	PARG07989	PARG07990	PARG07991	PARG07992	PARG07993
PARG07994	PARG07996	PARG07997	PARG07998	PARG07999	PARG08000	PARG08001
PARG08002	PARG08003	PARG08004	PARG08005	PARG08006	PARG08007	PARG08008
PARG08009	PARG08010	PARG08011	PARG08013	PARG08014	PARG08015	PARG08016
PARG08017	PARG08019	PARG08020	PARG08021	PARG08022	PARG08023	PARG08024
PARG08025	PARG08026	PARG08027	PARG08028	PARG08029	PARG08030	PARG08031
PARG08032	PARG08033	PARG08034	PARG08035	PARG08036	PARG08037	PARG08038
PARG08039	PARG08040	PARG08041	PARG08042	PARG08043	PARG08044	PARG08045
PARG08046	PARG08047	PARG08048	PARG08049	PARG08050	PARG08051	PARG08052
PARG08053	PARG08054	PARG08055	PARG08057	PARG08058	PARG08059	PARG08060
PARG08061	PARG08062	PARG08063	PARG08064	PARG08065	PARG08066	PARG08067
PARG08070	PARG08071	PARG08072	PARG08073	PARG08077	PARG08078	PARG08080

PARG08081	PARG08082	PARG08087	PARG08088	PARG08089	PARG08090	PARG08092
PARG08093	PARG08094	PARG08096	PARG08101	PARG08102	PARG08103	PARG08104
PARG08105	PARG08106	PARG08107	PARG08108	PARG08109	PARG08110	PARG08111
PARG08112	PARG08113	PARG08115	PARG08116	PARG08117	PARG08118	PARG08119
PARG08120	PARG08121	PARG08122	PARG08123	PARG08124	PARG08125	PARG08126
PARG08127	PARG08128	PARG08129	PARG08130	PARG08131	PARG08132	PARG08135
PARG08137	PARG08138	PARG08140	PARG08143	PARG08146	PARG08147	PARG08148
PARG08151	PARG08154	PARG08156	PARG08157	PARG08158	PARG08159	PARG08160
PARG08162	PARG08163	PARG08165	PARG08166	PARG08167	PARG08168	PARG08170
PARG08172	PARG08173	PARG08176	PARG08177	PARG08179	PARG08181	PARG08184
PARG08187	PARG08188	PARG08193	PARG08194	PARG08195	PARG08196	PARG08197
PARG08200	PARG08204	PARG08205	PARG08206	PARG08207	PARG08210	PARG08215
PARG08216	PARG08218	PARG08219	PARG08220	PARG08221	PARG08222	PARG08223
PARG08224	PARG08225	PARG08226	PARG08227	PARG08229	PARG08230	PARG08231
PARG08232	PARG08233	PARG08234	PARG08235	PARG08236	PARG08237	PARG08238
PARG08239	PARG08240	PARG08242	PARG08243	PARG08244	PARG08245	PARG08246
PARG08248	PARG08249	PARG08250	PARG08251	PARG08252	PARG08253	PARG08254
PARG08255	PARG08256	PARG08257	PARG08258	PARG08259	PARG08260	PARG08261
PARG08262	PARG08263	PARG08264	PARG08266	PARG08268	PARG08269	PARG08271
PARG08272	PARG08274	PARG08275	PARG08276	PARG08277	PARG08278	PARG08279
PARG08280	PARG08281	PARG08282	PARG08283	PARG08284	PARG08285	PARG08286
PARG08287	PARG08288	PARG08289	PARG08290	PARG08291	PARG08292	PARG08293
PARG08294	PARG08298	PARG08299	PARG08300	PARG08301	PARG08302	PARG08303
PARG08304	PARG08306	PARG08307	PARG08308	PARG08309	PARG08310	PARG08311
PARG08312	PARG08313	PARG08314	PARG08315	PARG08316	PARG08317	PARG08318
PARG08319	PARG08320	PARG08321	PARG08323	PARG08324	PARG08325	PARG08326
PARG08327	PARG08328	PARG08329	PARG08330	PARG08331	PARG08332	PARG08333
PARG08334	PARG08335	PARG08337	PARG08338	PARG08339	PARG08340	PARG08341
PARG08342	PARG08343	PARG08344	PARG08345	PARG08346	PARG08347	PARG08348
PARG08349	PARG08350	PARG08351	PARG08352	PARG08353	PARG08354	PARG08355
PARG08356	PARG08357	PARG08358	PARG08359	PARG08360	PARG08361	PARG08362
PARG08363	PARG08364	PARG08365	PARG08366	PARG08367	PARG08368	PARG08369
PARG08370	PARG08371	PARG08372	PARG08373	PARG08374	PARG08376	PARG08377
PARG08378	PARG08379	PARG08380	PARG08381	PARG08382	PARG08384	PARG08385
PARG08386	PARG08387	PARG08388	PARG08389	PARG08390	PARG08392	PARG08393
PARG08394	PARG08395	PARG08396	PARG08397	PARG08398	PARG08400	PARG08401
PARG08402	PARG08403	PARG08405	PARG08406	PARG08407	PARG08408	PARG08409
PARG08410	PARG08411	PARG08412	PARG08413	PARG08415	PARG08416	PARG08417
PARG08418	PARG08419	PARG08420	PARG08421	PARG08422	PARG08424	PARG08425
PARG08426	PARG08427	PARG08428	PARG08429	PARG08430	PARG08431	PARG08432
PARG08433	PARG08434	PARG08435	PARG08436	PARG08437	PARG08438	PARG08439
PARG08440	PARG08441	PARG08444	PARG08445	PARG08446	PARG08447	PARG08448
PARG08449	PARG08450	PARG08451	PARG08454	PARG08455	PARG08456	PARG08457
PARG08458	PARG08459	PARG08460	PARG08461	PARG08462	PARG08463	PARG08465
PARG08466	PARG08467	PARG08468	PARG08469	PARG08470	PARG08471	PARG08472
PARG08473	PARG08474	PARG08475	PARG08476	PARG08477	PARG08478	PARG08479

PARG08480	PARG08481	PARG08482	PARG08483	PARG08484	PARG08485	PARG08486
PARG08487	PARG08488	PARG08489	PARG08491	PARG08497	PARG08498	PARG08499
PARG08500	PARG08501	PARG08502	PARG08503	PARG08504	PARG08505	PARG08506
PARG08507	PARG08509	PARG08510	PARG08511	PARG08512	PARG08513	PARG08514
PARG08515	PARG08516	PARG08520	PARG08521	PARG08522	PARG08523	PARG08524
PARG08525	PARG08526	PARG08527	PARG08528	PARG08529	PARG08530	PARG08531
PARG08533	PARG08534	PARG08535	PARG08536	PARG08537	PARG08538	PARG08539
PARG08540	PARG08541	PARG08542	PARG08543	PARG08544	PARG08545	PARG08546
PARG08547	PARG08548	PARG08551	PARG08552	PARG08553	PARG08554	PARG08555
PARG08556	PARG08557	PARG08558	PARG08559	PARG08560	PARG08561	PARG08563
PARG08564	PARG08565	PARG08566	PARG08567	PARG08569	PARG08570	PARG08571
PARG08572	PARG08573	PARG08574	PARG08575	PARG08576	PARG08577	PARG08578
PARG08579	PARG08580	PARG08581	PARG08582	PARG08583	PARG08584	PARG08585
PARG08586	PARG08588	PARG08589	PARG08590	PARG08591	PARG08592	PARG08593
PARG08594	PARG08595	PARG08596	PARG08597	PARG08598	PARG08599	PARG08600
PARG08601	PARG08602	PARG08603	PARG08604	PARG08605	PARG08606	PARG08607
PARG08608	PARG08610	PARG08611	PARG08612	PARG08613	PARG08614	PARG08615
PARG08617	PARG08618	PARG08619	PARG08620	PARG08621	PARG08622	PARG08623
PARG08624	PARG08625	PARG08626	PARG08627	PARG08628	PARG08629	PARG08630
PARG08631	PARG08632	PARG08633	PARG08634	PARG08635	PARG08636	PARG08637
PARG08638	PARG08639	PARG08640	PARG08641	PARG08645	PARG08646	PARG08647
PARG08648	PARG08649	PARG08650	PARG08651	PARG08652	PARG08653	PARG08655
PARG08656	PARG08658	PARG08659	PARG08660	PARG08661	PARG08662	PARG08663
PARG08664	PARG08665	PARG08666	PARG08667	PARG08668	PARG08669	PARG08670
PARG08672	PARG08673	PARG08674	PARG08676	PARG08677	PARG08678	PARG08679
PARG08680	PARG08683	PARG08684	PARG08685	PARG08686	PARG08687	PARG08688
PARG08689	PARG08690	PARG08691	PARG08692	PARG08694	PARG08695	PARG08696
PARG08697	PARG08698	PARG08699	PARG08700	PARG08701	PARG08702	PARG08703
PARG08704	PARG08705	PARG08706	PARG08707	PARG08708	PARG08709	PARG08710
PARG08711	PARG08712	PARG08713	PARG08714	PARG08715	PARG08716	PARG08717
PARG08718	PARG08719	PARG08720	PARG08721	PARG08722	PARG08723	PARG08724
PARG08725	PARG08726	PARG08728	PARG08729	PARG08730	PARG08732	PARG08733
PARG08734	PARG08735	PARG08736	PARG08737	PARG08738	PARG08739	PARG08740
PARG08741	PARG08742	PARG08743	PARG08744	PARG08746	PARG08747	PARG08748
PARG08749	PARG08750	PARG08751	PARG08752	PARG08754	PARG08755	PARG08756
PARG08757	PARG08758	PARG08760	PARG08764	PARG08765	PARG08766	PARG08767
PARG08768	PARG08769	PARG08770	PARG08771	PARG08772	PARG08773	PARG08774
PARG08775	PARG08776	PARG08777	PARG08780	PARG08781	PARG08782	PARG08783
PARG08785	PARG08786	PARG08787	PARG08788	PARG08789	PARG08790	PARG08793
PARG08794	PARG08795	PARG08796	PARG08797	PARG08798	PARG08799	PARG08800
PARG08801	PARG08803	PARG08804	PARG08805	PARG08807	PARG08808	PARG08809
PARG08810	PARG08812	PARG08813	PARG08814	PARG08815	PARG08816	PARG08817
PARG08818	PARG08819	PARG08820	PARG08821	PARG08822	PARG08823	PARG08824
PARG08826	PARG08827	PARG08828	PARG08830	PARG08833	PARG08834	PARG08835
PARG08836	PARG08837	PARG08838	PARG08839	PARG08840	PARG08841	PARG08842
PARG08843	PARG08845	PARG08846	PARG08847	PARG08848	PARG08849	PARG08850

PARG08851	PARG08852	PARG08853	PARG08854	PARG08855	PARG08856	PARG08857
PARG08858	PARG08861	PARG08862	PARG08863	PARG08864	PARG08865	PARG08866
PARG08867	PARG08868	PARG08869	PARG08870	PARG08871	PARG08872	PARG08873
PARG08874	PARG08875	PARG08876	PARG08878	PARG08879	PARG08880	PARG08881
PARG08882	PARG08883	PARG08884	PARG08885	PARG08886	PARG08887	PARG08888
PARG08889	PARG08890	PARG08891	PARG08895	PARG08896	PARG08897	PARG08901
PARG08902	PARG08903	PARG08904	PARG08905	PARG08906	PARG08908	PARG08909
PARG08910	PARG08911	PARG08912	PARG08913	PARG08914	PARG08915	PARG08916
PARG08918	PARG08919	PARG08920	PARG08921	PARG08922	PARG08923	PARG08924
PARG08925	PARG08926	PARG08927	PARG08928	PARG08929	PARG08930	PARG08931
PARG08933	PARG08935	PARG08936	PARG08937	PARG08939	PARG08941	PARG08942
PARG08943	PARG08944	PARG08946	PARG08947	PARG08948	PARG08949	PARG08951
PARG08953	PARG08954	PARG08955	PARG08956	PARG08957	PARG08958	PARG08959
PARG08961	PARG08962	PARG08963	PARG08964	PARG08965	PARG08966	PARG08967
PARG08968	PARG08969	PARG08970	PARG08971	PARG08973	PARG08974	PARG08976
PARG08977	PARG08978	PARG08980	PARG08983	PARG08986	PARG08987	PARG08988
PARG08989	PARG08990	PARG08991	PARG08993	PARG08994	PARG08996	PARG08997
PARG08998	PARG08999	PARG09000	PARG09002	PARG09003	PARG09004	PARG09005
PARG09006	PARG09007	PARG09008	PARG09009	PARG09010	PARG09011	PARG09013
PARG09014	PARG09015	PARG09016	PARG09017	PARG09018	PARG09019	PARG09020
PARG09021	PARG09022	PARG09023	PARG09024	PARG09025	PARG09026	PARG09027
PARG09028	PARG09030	PARG09031	PARG09032	PARG09033	PARG09034	PARG09035
PARG09036	PARG09037	PARG09038	PARG09039	PARG09040	PARG09041	PARG09044
PARG09046	PARG09047	PARG09048	PARG09049	PARG09050	PARG09053	PARG09054
PARG09055	PARG09056	PARG09058	PARG09059	PARG09062	PARG09063	PARG09066
PARG09067	PARG09068	PARG09069	PARG09070	PARG09071	PARG09072	PARG09074
PARG09075	PARG09076	PARG09077	PARG09078	PARG09079	PARG09080	PARG09081
PARG09082	PARG09084	PARG09086	PARG09087	PARG09088	PARG09089	PARG09091
PARG09092	PARG09094	PARG09097	PARG09098	PARG09100	PARG09101	PARG09102
PARG09103	PARG09104	PARG09105	PARG09107	PARG09108	PARG09109	PARG09110
PARG09112	PARG09113	PARG09114	PARG09115	PARG09116	PARG09117	PARG09118
PARG09119	PARG09120	PARG09121	PARG09122	PARG09123	PARG09124	PARG09126
PARG09127	PARG09128	PARG09131	PARG09133	PARG09134	PARG09135	PARG09138
PARG09139	PARG09140	PARG09141	PARG09142	PARG09143	PARG09144	PARG09145
PARG09146	PARG09147	PARG09148	PARG09149	PARG09150	PARG09151	PARG09152
PARG09153	PARG09154	PARG09155	PARG09156	PARG09157	PARG09158	PARG09160
PARG09161	PARG09162	PARG09163	PARG09165	PARG09166	PARG09167	PARG09168
PARG09169	PARG09170	PARG09171	PARG09172	PARG09174	PARG09175	PARG09178
PARG09179	PARG09180	PARG09181	PARG09182	PARG09183	PARG09184	PARG09185
PARG09186	PARG09187	PARG09188	PARG09189	PARG09190	PARG09191	PARG09192
PARG09193	PARG09194	PARG09195	PARG09196	PARG09197	PARG09198	PARG09199
PARG09200	PARG09201	PARG09202	PARG09203	PARG09204	PARG09206	PARG09208
PARG09209	PARG09210	PARG09211	PARG09212	PARG09213	PARG09215	PARG09216
PARG09217	PARG09218	PARG09220	PARG09221	PARG09222	PARG09223	PARG09226
PARG09228	PARG09229	PARG09230	PARG09232	PARG09233	PARG09234	PARG09235
PARG09236	PARG09237	PARG09238	PARG09239	PARG09240	PARG09241	PARG09242

PARG09243	PARG09244	PARG09245	PARG09246	PARG09247	PARG09248	PARG09249
PARG09250	PARG09251	PARG09252	PARG09253	PARG09254	PARG09255	PARG09256
PARG09257	PARG09258	PARG09259	PARG09261	PARG09262	PARG09263	PARG09264
PARG09265	PARG09266	PARG09268	PARG09269	PARG09270	PARG09271	PARG09272
PARG09273	PARG09274	PARG09275	PARG09276	PARG09277	PARG09279	PARG09283
PARG09284	PARG09296	PARG09297	PARG09298	PARG09299	PARG09305	PARG09308
PARG09309	PARG09324	PARG09328	PARG09329	PARG09330	PARG09331	PARG09333
PARG09334	PARG09339	PARG09341	PARG09343	PARG09344	PARG09345	PARG09346
PARG09349	PARG09351	PARG09353	PARG09355	PARG09356	PARG09358	PARG09359
PARG09360	PARG09361	PARG09362	PARG09363	PARG09364	PARG09365	PARG09366
PARG09367	PARG09368	PARG09370	PARG09371	PARG09372	PARG09374	PARG09375
PARG09376	PARG09386	PARG09390	PARG09392	PARG09393	PARG09394	PARG09395
PARG09397	PARG09398	PARG09400	PARG09407	PARG09409	PARG09412	PARG09413
PARG09417	PARG09419	PARG09420	PARG09421	PARG09424	PARG09425	PARG09428
PARG09429	PARG09431	PARG09432	PARG09433	PARG09434	PARG09435	PARG09436
PARG09437	PARG09441	PARG09445	PARG09446	PARG09447	PARG09449	PARG09450
PARG09451	PARG09452	PARG09453	PARG09454	PARG09456	PARG09457	PARG09458
PARG09459	PARG09460	PARG09461	PARG09462	PARG09463	PARG09464	PARG09465
PARG09466	PARG09468	PARG09469	PARG09470	PARG09472	PARG09483	PARG09484
PARG09486	PARG09487	PARG09490	PARG09491	PARG09492	PARG09493	PARG09494
PARG09495	PARG09497	PARG09502	PARG09503	PARG09504	PARG09505	PARG09506
PARG09509	PARG09524	PARG09531	PARG09532	PARG09534	PARG09537	PARG09539
PARG09540	PARG09541	PARG09542	PARG09543	PARG09544	PARG09549	PARG09551
PARG09552	PARG09553	PARG09555	PARG09557	PARG09561	PARG09562	PARG09570
PARG09571	PARG09573	PARG09576	PARG09577	PARG09579	PARG09586	PARG09593
PARG09594	PARG09597	PARG09604	PARG09606	PARG09607	PARG09608	PARG09609
PARG09620	PARG09621	PARG09622	PARG09623	PARG09624	PARG09625	PARG09626
PARG09627	PARG09632	PARG09644	PARG09645	PARG09646	PARG09647	PARG09649
PARG09651	PARG09652	PARG09653	PARG09657	PARG09658	PARG09661	PARG09662
PARG09663	PARG09664	PARG09665	PARG09666	PARG09669	PARG09670	PARG09672
PARG09673	PARG09676	PARG09680	PARG09681	PARG09682	PARG09683	PARG09684
PARG09685	PARG09687	PARG09689	PARG09691	PARG09692	PARG09698	PARG09699
PARG09702	PARG09705	PARG09712	PARG09713	PARG09714	PARG09715	PARG09720
PARG09725	PARG09726	PARG09727	PARG09728	PARG09729	PARG09730	PARG09733
PARG09734	PARG09739	PARG09740	PARG09744	PARG09745	PARG09746	PARG09750
PARG09751	PARG09752	PARG09754	PARG09755	PARG09756	PARG09757	PARG09759
PARG09760	PARG09761	PARG09762	PARG09763	PARG09765	PARG09768	PARG09783
PARG09788	PARG09789	PARG09792	PARG09795	PARG09798	PARG09799	PARG09800
PARG09807	PARG09810	PARG09811	PARG09815	PARG09816	PARG09817	PARG09818
PARG09819	PARG09824	PARG09829	PARG09830	PARG09838	PARG09840	PARG09841
PARG09844	PARG09859	PARG09870	PARG09871	PARG09872	PARG09873	PARG09874
PARG09876	PARG09880	PARG09884	PARG09885	PARG09887	PARG09888	PARG09891
PARG09893	PARG09895	PARG09897	PARG09900	PARG09910	PARG09913	PARG09917
PARG09920	PARG09927	PARG09933	PARG09935	PARG09938	PARG09941	PARG09942
PARG09947	PARG09948	PARG09950	PARG09951	PARG09955	PARG09956	PARG09958
PARG09966	PARG09968	PARG09969	PARG09970	PARG09971	PARG09972	PARG09973

PARG09974	PARG09976	PARG09977	PARG09978	PARG09979	PARG09983	PARG09988
PARG09989	PARG09990	PARG09992	PARG09994	PARG09995	PARG09996	PARG09997
PARG09998	PARG09999	PARG10000	PARG10001	PARG10002	PARG10003	PARG10004
PARG10010	PARG10011	PARG10013	PARG10014	PARG10015	PARG10016	PARG10017
PARG10019	PARG10023	PARG10024	PARG10032	PARG10037	PARG10039	PARG10040
PARG10041	PARG10043	PARG10045	PARG10046	PARG10048	PARG10054	PARG10055
PARG10059	PARG10062	PARG10063	PARG10064	PARG10069	PARG10074	PARG10075
PARG10077	PARG10078	PARG10079	PARG10080	PARG10081	PARG10083	PARG10086
PARG10088	PARG10089	PARG10098	PARG10099	PARG10100	PARG10102	PARG10106
PARG10107	PARG10108	PARG10115	PARG10116	PARG10119	PARG10120	PARG10121
PARG10122	PARG10129	PARG10134	PARG10135	PARG10138	PARG10142	PARG10143
PARG10144	PARG10145	PARG10146	PARG10149	PARG10150	PARG10153	PARG10158
PARG10159	PARG10161	PARG10164	PARG10165	PARG10166	PARG10167	PARG10173
PARG10184	PARG10185	PARG10186	PARG10189	PARG10190	PARG10191	PARG10193
PARG10195	PARG10196	PARG10197	PARG10199	PARG10201	PARG10202	PARG10203
PARG10204	PARG10206	PARG10207	PARG10208	PARG10209	PARG10210	PARG10215
PARG10216	PARG10219	PARG10221	PARG10222	PARG10223	PARG10224	PARG10225
PARG10229	PARG10233	PARG10235	PARG10239	PARG10243	PARG10244	PARG10246
PARG10247	PARG10248	PARG10249	PARG10250	PARG10252	PARG10254	PARG10256
PARG10257	PARG10258	PARG10259	PARG10260	PARG10261	PARG10262	PARG10263
PARG10266	PARG10267	PARG10268	PARG10269	PARG10270	PARG10273	PARG10276
PARG10277	PARG10279	PARG10280	PARG10281	PARG10282	PARG10284	PARG10285
PARG10286	PARG10287	PARG10289	PARG10290	PARG10291	PARG10292	PARG10293
PARG10294	PARG10295	PARG10297	PARG10301	PARG10302	PARG10303	PARG10304
PARG10305	PARG10306	PARG10307	PARG10308	PARG10309	PARG10310	PARG10311
PARG10312	PARG10313	PARG10314	PARG10316	PARG10317	PARG10318	PARG10319
PARG10320	PARG10322	PARG10324	PARG10325	PARG10327	PARG10328	PARG10329
PARG10331	PARG10333	PARG10335	PARG10336	PARG10337	PARG10339	PARG10340
PARG10342	PARG10343	PARG10344	PARG10345	PARG10347	PARG10349	PARG10350
PARG10351	PARG10352	PARG10353	PARG10354	PARG10355	PARG10356	PARG10357
PARG10358	PARG10359	PARG10360	PARG10361	PARG10362	PARG10363	PARG10364
PARG10365	PARG10366	PARG10367	PARG10368	PARG10369	PARG10370	PARG10371
PARG10373	PARG10374	PARG10375	PARG10376	PARG10380	PARG10382	PARG10384
PARG10385	PARG10386	PARG10387	PARG10388	PARG10390	PARG10391	PARG10392
PARG10393	PARG10394	PARG10395	PARG10396	PARG10397	PARG10398	PARG10399
PARG10400	PARG10401	PARG10402	PARG10403	PARG10404	PARG10405	PARG10406
PARG10407	PARG10408	PARG10409	PARG10410	PARG10411	PARG10412	PARG10415
PARG10416	PARG10417	PARG10418	PARG10419	PARG10420	PARG10422	PARG10424
PARG10425	PARG10426	PARG10427	PARG10428	PARG10429	PARG10430	PARG10431
PARG10432	PARG10435	PARG10436	PARG10437	PARG10438	PARG10439	PARG10440
PARG10442	PARG10444	PARG10445	PARG10446	PARG10449	PARG10450	PARG10451
PARG10452	PARG10453	PARG10454	PARG10455	PARG10456	PARG10457	PARG10458
PARG10459	PARG10461	PARG10462	PARG10463	PARG10464	PARG10465	PARG10466
PARG10468	PARG10469	PARG10470	PARG10471	PARG10472	PARG10473	PARG10474
PARG10475	PARG10476	PARG10479	PARG10480	PARG10483	PARG10486	PARG10488
PARG10489	PARG10490	PARG10493	PARG10497	PARG10500	PARG10501	PARG10503

PARG10505	PARG10506	PARG10507	PARG10508	PARG10509	PARG10510	PARG10511
PARG10512	PARG10513	PARG10514	PARG10515	PARG10516	PARG10517	PARG10518
PARG10519	PARG10521	PARG10523	PARG10524	PARG10525	PARG10526	PARG10528
PARG10530	PARG10533	PARG10534	PARG10536	PARG10537	PARG10538	PARG10540
PARG10542	PARG10543	PARG10545	PARG10546	PARG10547	PARG10548	PARG10549
PARG10550	PARG10551	PARG10552	PARG10553	PARG10554	PARG10555	PARG10556
PARG10557	PARG10558	PARG10559	PARG10561	PARG10562	PARG10563	PARG10564
PARG10565	PARG10566	PARG10569	PARG10571	PARG10572	PARG10573	PARG10574
PARG10575	PARG10577	PARG10578	PARG10580	PARG10582	PARG10583	PARG10584
PARG10585	PARG10586	PARG10587	PARG10588	PARG10589	PARG10590	PARG10591
PARG10592	PARG10593	PARG10595	PARG10596	PARG10597	PARG10601	PARG10602
PARG10603	PARG10605	PARG10607	PARG10608	PARG10609	PARG10610	PARG10612
PARG10613	PARG10614	PARG10615	PARG10616	PARG10617	PARG10618	PARG10619
PARG10620	PARG10622	PARG10623	PARG10624	PARG10625	PARG10626	PARG10627
PARG10628	PARG10629	PARG10630	PARG10632	PARG10634	PARG10637	PARG10638
PARG10639	PARG10640	PARG10641	PARG10646	PARG10647	PARG10648	PARG10649
PARG10650	PARG10651	PARG10652	PARG10653	PARG10654	PARG10655	PARG10656
PARG10657	PARG10658	PARG10659	PARG10660	PARG10661	PARG10662	PARG10663
PARG10664	PARG10665	PARG10666	PARG10667	PARG10668	PARG10669	PARG10671
PARG10672	PARG10673	PARG10674	PARG10677	PARG10678	PARG10679	PARG10680
PARG10681	PARG10682	PARG10683	PARG10684	PARG10686	PARG10687	PARG10688
PARG10691	PARG10692	PARG10693	PARG10694	PARG10696	PARG10697	PARG10698
PARG10699	PARG10700	PARG10702	PARG10703	PARG10704	PARG10705	PARG10706
PARG10707	PARG10708	PARG10709	PARG10710	PARG10714	PARG10715	PARG10716
PARG10718	PARG10719	PARG10720	PARG10721	PARG10722	PARG10723	PARG10724
PARG10725	PARG10726	PARG10728	PARG10729	PARG10730	PARG10731	PARG10732
PARG10733	PARG10734	PARG10735	PARG10736	PARG10737	PARG10738	PARG10739
PARG10740	PARG10741	PARG10742	PARG10743	PARG10745	PARG10746	PARG10747
PARG10748	PARG10749	PARG10750	PARG10751	PARG10752	PARG10753	PARG10754
PARG10755	PARG10756	PARG10758	PARG10759	PARG10760	PARG10761	PARG10762
PARG10763	PARG10764	PARG10765	PARG10766	PARG10767	PARG10768	PARG10769
PARG10772	PARG10773	PARG10774	PARG10775	PARG10776	PARG10778	PARG10779
PARG10780	PARG10781	PARG10782	PARG10784	PARG10785	PARG10786	PARG10787
PARG10788	PARG10789	PARG10790	PARG10791	PARG10792	PARG10793	PARG10794
PARG10795	PARG10796	PARG10801	PARG10803	PARG10804	PARG10805	PARG10806
PARG10807	PARG10808	PARG10809	PARG10810	PARG10811	PARG10812	PARG10813
PARG10814	PARG10815	PARG10816	PARG10817	PARG10818	PARG10821	PARG10822
PARG10823	PARG10826	PARG10828	PARG10830	PARG10832	PARG10840	PARG10841
PARG10842	PARG10843	PARG10844	PARG10845	PARG10846	PARG10847	PARG10849
PARG10850	PARG10851	PARG10852	PARG10854	PARG10855	PARG10856	PARG10857
PARG10858	PARG10859	PARG10860	PARG10861	PARG10862	PARG10863	PARG10864
PARG10865	PARG10871	PARG10872	PARG10873	PARG10874	PARG10875	PARG10876
PARG10879	PARG10880	PARG10881	PARG10882	PARG10883	PARG10884	PARG10885
PARG10888	PARG10889	PARG10890	PARG10892	PARG10895	PARG10909	PARG10911
PARG10913	PARG10918	PARG10922	PARG10925	PARG10926	PARG10927	PARG10928
PARG10929	PARG10930	PARG10932	PARG10933	PARG10934	PARG10935	PARG10936

PARG10938	PARG10939	PARG10941	PARG10942	PARG10943	PARG10944	PARG10945
PARG10946	PARG10947	PARG10948	PARG10949	PARG10950	PARG10951	PARG10952
PARG10953	PARG10954	PARG10955	PARG10956	PARG10957	PARG10958	PARG10960
PARG10961	PARG10962	PARG10963	PARG10964	PARG10965	PARG10966	PARG10967
PARG10970	PARG10971	PARG10973	PARG10974	PARG10975	PARG10976	PARG10977
PARG10978	PARG10979	PARG10981	PARG10982	PARG10984	PARG10985	PARG10986
PARG10987	PARG10988	PARG10993	PARG10994	PARG10995	PARG10998	PARG10999
PARG11000	PARG11002	PARG11004	PARG11005	PARG11006	PARG11007	PARG11008
PARG11010	PARG11011	PARG11012	PARG11013	PARG11014	PARG11015	PARG11016
PARG11019	PARG11021	PARG11022	PARG11023	PARG11024	PARG11025	PARG11026
PARG11027	PARG11028	PARG11032	PARG11036	PARG11037	PARG11038	PARG11039
PARG11040	PARG11041	PARG11042	PARG11047	PARG11048	PARG11049	PARG11050
PARG11051	PARG11052	PARG11054	PARG11056	PARG11057	PARG11059	PARG11064
PARG11065	PARG11066	PARG11068	PARG11070	PARG11072	PARG11073	PARG11075
PARG11077	PARG11078	PARG11079	PARG11080	PARG11081	PARG11082	PARG11083
PARG11084	PARG11085	PARG11086	PARG11087	PARG11088	PARG11089	PARG11090
PARG11091	PARG11092	PARG11093	PARG11094	PARG11095	PARG11096	PARG11097
PARG11098	PARG11099	PARG11100	PARG11101	PARG11102	PARG11103	PARG11104
PARG11105	PARG11106	PARG11107	PARG11108	PARG11109	PARG11110	PARG11111
PARG11112	PARG11113	PARG11114	PARG11116	PARG11117	PARG11118	PARG11119
PARG11124	PARG11126	PARG11127	PARG11128	PARG11129	PARG11130	PARG11131
PARG11132	PARG11133	PARG11134	PARG11135	PARG11137	PARG11138	PARG11139
PARG11140	PARG11144	PARG11146	PARG11147	PARG11150	PARG11151	PARG11152
PARG11153	PARG11154	PARG11155	PARG11156	PARG11157	PARG11158	PARG11159
PARG11160	PARG11161	PARG11162	PARG11163	PARG11164	PARG11165	PARG11166
PARG11168	PARG11170	PARG11171	PARG11173	PARG11174	PARG11175	PARG11183
PARG11184	PARG11185	PARG11187	PARG11188	PARG11189	PARG11191	PARG11192
PARG11193	PARG11194	PARG11196	PARG11199	PARG11200	PARG11208	PARG11209
PARG11210	PARG11211	PARG11212	PARG11213	PARG11214	PARG11216	PARG11217
PARG11222	PARG11223	PARG11224	PARG11225	PARG11226	PARG11227	PARG11228
PARG11229	PARG11230	PARG11231	PARG11232	PARG11233	PARG11234	PARG11235
PARG11236	PARG11237	PARG11238	PARG11239	PARG11240	PARG11241	PARG11242
PARG11243	PARG11244	PARG11245	PARG11246	PARG11247	PARG11248	PARG11249
PARG11251	PARG11252	PARG11254	PARG11255	PARG11256	PARG11257	PARG11258
PARG11260	PARG11261	PARG11262	PARG11263	PARG11267	PARG11268	PARG11269
PARG11270	PARG11271	PARG11272	PARG11273	PARG11274	PARG11275	PARG11276
PARG11277	PARG11278	PARG11279	PARG11280	PARG11281	PARG11282	PARG11283
PARG11284	PARG11285	PARG11287	PARG11288	PARG11289	PARG11291	PARG11292
PARG11293	PARG11294	PARG11295	PARG11296	PARG11297	PARG11298	PARG11299
PARG11301	PARG11304	PARG11305	PARG11306	PARG11308	PARG11310	PARG11311
PARG11312	PARG11314	PARG11317	PARG11318	PARG11326	PARG11329	PARG11331
PARG11332	PARG11333	PARG11336	PARG11337	PARG11338	PARG11339	PARG11340
PARG11341	PARG11342	PARG11343	PARG11344	PARG11345	PARG11346	PARG11347
PARG11348	PARG11349	PARG11350	PARG11351	PARG11352	PARG11353	PARG11354
PARG11360	PARG11361	PARG11362	PARG11363	PARG11365	PARG11366	PARG11367
PARG11368	PARG11369	PARG11370	PARG11371	PARG11372	PARG11373	PARG11374

PARG11375	PARG11376	PARG11377	PARG11378	PARG11379	PARG11380	PARG11381
PARG11382	PARG11385	PARG11386	PARG11388	PARG11389	PARG11390	PARG11391
PARG11392	PARG11393	PARG11394	PARG11395	PARG11397	PARG11398	PARG11399
PARG11400	PARG11401	PARG11402	PARG11403	PARG11404	PARG11405	PARG11406
PARG11407	PARG11408	PARG11409	PARG11410	PARG11411	PARG11412	PARG11413
PARG11414	PARG11415	PARG11416	PARG11417	PARG11422	PARG11423	PARG11424
PARG11425	PARG11426	PARG11427	PARG11428	PARG11429	PARG11430	PARG11432
PARG11439	PARG11442	PARG11443	PARG11444	PARG11445	PARG11447	PARG11448
PARG11449	PARG11450	PARG11451	PARG11452	PARG11453	PARG11454	PARG11455
PARG11456	PARG11457	PARG11458	PARG11462	PARG11463	PARG11464	PARG11465
PARG11468	PARG11470	PARG11471	PARG11472	PARG11473	PARG11474	PARG11476
PARG11477	PARG11478	PARG11480	PARG11481	PARG11482	PARG11487	PARG11488
PARG11489	PARG11490	PARG11491	PARG11492	PARG11493	PARG11494	PARG11495
PARG11496	PARG11497	PARG11498	PARG11499	PARG11500	PARG11501	PARG11502
PARG11503	PARG11511	PARG11512	PARG11513	PARG11514	PARG11515	PARG11516
PARG11517	PARG11518	PARG11519	PARG11520	PARG11521	PARG11524	PARG11525
PARG11526	PARG11528	PARG11529	PARG11530	PARG11531	PARG11532	PARG11533
PARG11536	PARG11537	PARG11538	PARG11539	PARG11540	PARG11541	PARG11542
PARG11543	PARG11544	PARG11545	PARG11546	PARG11547	PARG11548	PARG11549
PARG11550	PARG11551	PARG11552	PARG11553	PARG11554	PARG11558	PARG11559
PARG11560	PARG11561	PARG11564	PARG11568	PARG11570	PARG11571	PARG11572
PARG11575	PARG11576	PARG11577	PARG11578	PARG11579	PARG11580	PARG11581
PARG11582	PARG11583	PARG11584	PARG11585	PARG11586	PARG11587	PARG11588
PARG11589	PARG11591	PARG11592	PARG11593	PARG11595	PARG11596	PARG11604
PARG11613	PARG11614	PARG11616	PARG11622	PARG11623	PARG11624	PARG11625
PARG11626	PARG11627	PARG11630	PARG11631	PARG11632	PARG11633	PARG11634
PARG11635	PARG11636	PARG11637	PARG11638	PARG11639	PARG11644	PARG11645
PARG11646	PARG11647	PARG11648	PARG11649	PARG11650	PARG11651	PARG11652
PARG11653	PARG11654	PARG11656	PARG11657	PARG11658	PARG11659	PARG11660
PARG11661	PARG11662	PARG11663	PARG11665	PARG11666	PARG11667	PARG11668
PARG11669	PARG11670	PARG11671	PARG11672	PARG11673	PARG11674	PARG11675
PARG11676	PARG11677	PARG11678	PARG11679	PARG11680	PARG11681	PARG11682
PARG11684	PARG11685	PARG11686	PARG11688	PARG11689	PARG11690	PARG11691
PARG11692	PARG11693	PARG11694	PARG11695	PARG11697	PARG11698	PARG11699
PARG11700	PARG11701	PARG11702	PARG11703	PARG11704	PARG11706	PARG11707
PARG11708	PARG11709	PARG11712	PARG11714	PARG11715	PARG11718	PARG11719
PARG11720	PARG11721	PARG11722	PARG11723	PARG11724	PARG11725	PARG11727
PARG11728	PARG11729	PARG11730	PARG11732	PARG11733	PARG11734	PARG11736
PARG11737	PARG11738	PARG11739	PARG11740	PARG11741	PARG11742	PARG11743
PARG11745	PARG11746	PARG11748	PARG11749	PARG11750	PARG11751	PARG11754
PARG11755	PARG11760	PARG11766	PARG11773	PARG11774	PARG11775	PARG11779
PARG11781	PARG11782	PARG11787	PARG11789	PARG11790	PARG11791	PARG11792
PARG11796	PARG11797	PARG11799	PARG11802	PARG11803	PARG11804	PARG11805
PARG11806	PARG11808	PARG11809	PARG11810	PARG11811	PARG11812	PARG11813
PARG11814	PARG11815	PARG11816	PARG11817	PARG11818	PARG11819	PARG11820
PARG11821	PARG11822	PARG11823	PARG11824	PARG11825	PARG11826	PARG11827

PARG11828	PARG11829	PARG11830	PARG11831	PARG11832	PARG11833	PARG11834
PARG11835	PARG11836	PARG11839	PARG11840	PARG11841	PARG11842	PARG11843
PARG11844	PARG11845	PARG11846	PARG11847	PARG11848	PARG11849	PARG11850
PARG11851	PARG11852	PARG11855	PARG11856	PARG11857	PARG11858	PARG11859
PARG11860	PARG11861	PARG11862	PARG11863	PARG11864	PARG11865	PARG11866
PARG11867	PARG11868	PARG11869	PARG11870	PARG11871	PARG11872	PARG11873
PARG11876	PARG11877	PARG11878	PARG11879	PARG11880	PARG11881	PARG11882
PARG11883	PARG11884	PARG11885	PARG11886	PARG11887	PARG11888	PARG11889
PARG11890	PARG11891	PARG11892	PARG11893	PARG11894	PARG11895	PARG11896
PARG11897	PARG11898	PARG11900	PARG11901	PARG11902	PARG11903	PARG11904
PARG11905	PARG11906	PARG11908	PARG11909	PARG11910	PARG11911	PARG11912
PARG11913	PARG11914	PARG11915	PARG11916	PARG11917	PARG11918	PARG11919
PARG11920	PARG11921	PARG11922	PARG11923	PARG11924	PARG11925	PARG11926
PARG11927	PARG11928	PARG11929	PARG11931	PARG11932	PARG11935	PARG11937
PARG11938	PARG11940	PARG11943	PARG11944	PARG11945	PARG11946	PARG11947
PARG11948	PARG11949	PARG11950	PARG11951	PARG11952	PARG11954	PARG11955
PARG11956	PARG11957	PARG11958	PARG11959	PARG11960	PARG11961	PARG11962
PARG11963	PARG11964	PARG11965	PARG11966	PARG11967	PARG11969	PARG11970
PARG11971	PARG11972	PARG11973	PARG11975	PARG11976	PARG11977	PARG11978
PARG11979	PARG11980	PARG11981	PARG11982	PARG11983	PARG11984	PARG11985
PARG11986	PARG11987	PARG11988	PARG11989	PARG11990	PARG11991	PARG11992
PARG11993	PARG11994	PARG11995	PARG11996	PARG11997	PARG11998	PARG11999
PARG12001	PARG12002	PARG12003	PARG12004	PARG12005	PARG12006	PARG12008
PARG12009	PARG12010	PARG12011	PARG12012	PARG12013	PARG12014	PARG12015
PARG12016	PARG12017	PARG12018	PARG12019	PARG12020	PARG12021	PARG12022
PARG12023	PARG12024	PARG12025	PARG12026	PARG12027	PARG12028	PARG12030
PARG12039	PARG12040	PARG12041	PARG12043	PARG12044	PARG12045	PARG12046
PARG12047	PARG12048	PARG12053	PARG12054	PARG12055	PARG12056	PARG12057
PARG12061	PARG12062	PARG12063	PARG12064	PARG12065	PARG12066	PARG12067
PARG12068	PARG12069	PARG12070	PARG12071	PARG12072	PARG12073	PARG12074
PARG12075	PARG12076	PARG12077	PARG12079	PARG12080	PARG12081	PARG12082
PARG12083	PARG12084	PARG12085	PARG12086	PARG12089	PARG12090	PARG12091
PARG12092	PARG12093	PARG12094	PARG12095	PARG12096	PARG12097	PARG12098
PARG12100	PARG12104	PARG12105	PARG12106	PARG12107	PARG12108	PARG12109
PARG12110	PARG12111	PARG12112	PARG12115	PARG12116	PARG12117	PARG12118
PARG12119	PARG12120	PARG12121	PARG12122	PARG12123	PARG12124	PARG12125
PARG12126	PARG12127	PARG12128	PARG12129	PARG12130	PARG12131	PARG12132
PARG12133	PARG12134	PARG12136	PARG12137	PARG12138	PARG12139	PARG12140
PARG12141	PARG12142	PARG12143	PARG12144	PARG12145	PARG12146	PARG12147
PARG12148	PARG12151	PARG12152	PARG12153	PARG12154	PARG12155	PARG12156
PARG12157	PARG12158	PARG12159	PARG12160	PARG12163	PARG12164	PARG12166
PARG12167	PARG12168	PARG12169	PARG12170	PARG12171	PARG12172	PARG12173
PARG12174	PARG12175	PARG12176	PARG12177	PARG12178	PARG12179	PARG12180
PARG12181	PARG12182	PARG12183	PARG12184	PARG12185	PARG12186	PARG12187
PARG12188	PARG12189	PARG12190	PARG12191	PARG12192	PARG12194	PARG12195
PARG12196	PARG12197	PARG12198	PARG12199	PARG12200	PARG12201	PARG12203

PARG12204	PARG12205	PARG12206	PARG12207	PARG12208	PARG12209	PARG12210
PARG12211	PARG12212	PARG12213	PARG12214	PARG12215	PARG12216	PARG12217
PARG12218	PARG12219	PARG12220	PARG12221	PARG12222	PARG12223	PARG12224
PARG12225	PARG12226	PARG12227	PARG12228	PARG12229	PARG12230	PARG12231
PARG12232	PARG12234	PARG12235	PARG12236	PARG12237	PARG12238	PARG12239
PARG12240	PARG12241	PARG12242	PARG12243	PARG12244	PARG12245	PARG12246
PARG12247	PARG12248	PARG12249	PARG12250	PARG12251	PARG12252	PARG12253
PARG12254	PARG12255	PARG12257	PARG12258	PARG12259	PARG12261	PARG12262
PARG12264	PARG12265	PARG12266	PARG12267	PARG12268	PARG12269	PARG12270
PARG12273	PARG12274	PARG12276	PARG12280	PARG12281	PARG12282	PARG12283
PARG12284	PARG12285	PARG12286	PARG12287	PARG12288	PARG12289	PARG12290
PARG12291	PARG12292	PARG12293	PARG12294	PARG12295	PARG12296	PARG12297
PARG12298	PARG12299	PARG12300	PARG12302	PARG12303	PARG12304	PARG12305
PARG12306	PARG12307	PARG12308	PARG12309	PARG12311	PARG12312	PARG12313
PARG12314	PARG12315	PARG12316	PARG12317	PARG12318	PARG12319	PARG12320
PARG12321	PARG12322	PARG12323	PARG12324	PARG12325	PARG12326	PARG12327
PARG12328	PARG12329	PARG12330	PARG12331	PARG12332	PARG12333	PARG12334
PARG12335	PARG12336	PARG12337	PARG12338	PARG12339	PARG12340	PARG12341
PARG12342	PARG12343	PARG12344	PARG12345	PARG12346	PARG12347	PARG12348
PARG12349	PARG12350	PARG12351	PARG12352	PARG12353	PARG12354	PARG12355
PARG12356	PARG12357	PARG12358	PARG12359	PARG12360	PARG12361	PARG12362
PARG12363	PARG12364	PARG12365	PARG12366	PARG12367	PARG12368	PARG12369
PARG12370	PARG12372	PARG12373	PARG12374	PARG12375	PARG12376	PARG12377
PARG12378	PARG12379	PARG12380	PARG12381	PARG12382	PARG12383	PARG12384
PARG12385	PARG12386	PARG12387	PARG12388	PARG12389	PARG12390	PARG12391
PARG12392	PARG12393	PARG12394	PARG12397	PARG12398	PARG12399	PARG12400
PARG12401	PARG12402	PARG12403	PARG12404	PARG12409	PARG12410	PARG12411
PARG12413	PARG12414	PARG12416	PARG12417	PARG12418	PARG12419	PARG12421
PARG12422	PARG12426	PARG12427	PARG12428	PARG12429	PARG12430	PARG12431
PARG12432	PARG12433	PARG12434	PARG12436	PARG12438	PARG12439	PARG12440
PARG12441	PARG12442	PARG12443	PARG12444	PARG12448	PARG12449	PARG12450
PARG12451	PARG12452	PARG12453	PARG12454	PARG12455	PARG12457	PARG12458
PARG12459	PARG12460	PARG12461	PARG12462	PARG12463	PARG12464	PARG12465
PARG12466	PARG12467	PARG12468	PARG12469	PARG12470	PARG12471	PARG12472
PARG12473	PARG12474	PARG12475	PARG12476	PARG12477	PARG12478	PARG12479
PARG12480	PARG12481	PARG12482	PARG12484	PARG12485	PARG12486	PARG12487
PARG12488	PARG12489	PARG12490	PARG12491	PARG12492	PARG12493	PARG12494
PARG12495	PARG12496	PARG12497	PARG12498	PARG12499	PARG12501	PARG12502
PARG12503	PARG12504	PARG12506	PARG12507	PARG12508	PARG12510	PARG12511
PARG12513	PARG12515	PARG12517	PARG12518	PARG12519	PARG12520	PARG12521
PARG12523	PARG12524	PARG12526	PARG12527	PARG12528	PARG12529	PARG12530
PARG12531	PARG12532	PARG12533	PARG12534	PARG12535	PARG12536	PARG12537
PARG12538	PARG12539	PARG12540	PARG12541	PARG12542	PARG12543	PARG12544
PARG12545	PARG12546	PARG12547	PARG12548	PARG12549	PARG12550	PARG12551
PARG12552	PARG12553	PARG12556	PARG12557	PARG12558	PARG12559	PARG12561
PARG12562	PARG12563	PARG12564	PARG12565	PARG12566	PARG12567	PARG12568

PARG12569	PARG12570	PARG12571	PARG12572	PARG12574	PARG12575	PARG12576
PARG12577	PARG12578	PARG12580	PARG12581	PARG12582	PARG12583	PARG12584
PARG12585	PARG12586	PARG12587	PARG12588	PARG12589	PARG12590	PARG12591
PARG12592	PARG12597	PARG12598	PARG12599	PARG12600	PARG12601	PARG12602
PARG12603	PARG12604	PARG12605	PARG12606	PARG12607	PARG12608	PARG12609
PARG12610	PARG12611	PARG12612	PARG12613	PARG12614	PARG12615	PARG12616
PARG12617	PARG12618	PARG12619	PARG12620	PARG12621	PARG12622	PARG12623
PARG12624	PARG12626	PARG12627	PARG12628	PARG12629	PARG12630	PARG12631
PARG12632	PARG12633	PARG12634	PARG12635	PARG12636	PARG12637	PARG12638
PARG12639	PARG12640	PARG12641	PARG12642	PARG12643	PARG12644	PARG12645
PARG12646	PARG12647	PARG12648	PARG12649	PARG12650	PARG12651	PARG12652
PARG12653	PARG12654	PARG12655	PARG12657	PARG12658	PARG12659	PARG12660
PARG12661	PARG12662	PARG12663	PARG12664	PARG12665	PARG12666	PARG12667
PARG12670	PARG12671	PARG12672	PARG12673	PARG12674	PARG12675	PARG12676
PARG12677	PARG12678	PARG12679	PARG12680	PARG12681	PARG12682	PARG12683
PARG12684	PARG12685	PARG12686	PARG12687	PARG12688	PARG12689	PARG12690
PARG12691	PARG12692	PARG12693	PARG12694	PARG12695	PARG12696	PARG12697
PARG12698	PARG12699	PARG12700	PARG12701	PARG12702	PARG12703	PARG12704
PARG12705	PARG12706	PARG12707	PARG12708	PARG12709	PARG12710	PARG12712
PARG12713	PARG12714	PARG12715	PARG12716	PARG12717	PARG12718	PARG12719
PARG12720	PARG12721	PARG12722	PARG12723	PARG12724	PARG12725	PARG12726
PARG12727	PARG12728	PARG12729	PARG12730	PARG12731	PARG12732	PARG12733
PARG12734	PARG12735	PARG12736	PARG12737	PARG12738	PARG12739	PARG12740
PARG12741	PARG12743	PARG12745	PARG12746	PARG12747	PARG12748	PARG12749
PARG12750	PARG12751	PARG12753	PARG12754	PARG12755	PARG12756	PARG12757
PARG12759	PARG12760	PARG12761	PARG12762	PARG12763	PARG12764	PARG12765
PARG12766	PARG12767	PARG12768	PARG12771	PARG12772	PARG12773	PARG12774
PARG12775	PARG12776	PARG12777	PARG12778	PARG12779	PARG12780	PARG12781
PARG12782	PARG12783	PARG12784	PARG12785	PARG12786	PARG12787	PARG12788
PARG12789	PARG12790	PARG12791	PARG12792	PARG12793	PARG12794	PARG12795
PARG12797	PARG12798	PARG12799	PARG12800	PARG12801	PARG12802	PARG12803
PARG12804	PARG12805	PARG12806	PARG12807	PARG12808	PARG12809	PARG12810
PARG12811	PARG12812	PARG12813	PARG12814	PARG12815	PARG12816	PARG12817
PARG12818	PARG12819	PARG12820	PARG12821	PARG12822	PARG12823	PARG12824
PARG12825	PARG12826	PARG12827	PARG12828	PARG12829	PARG12830	PARG12831
PARG12832	PARG12833	PARG12834	PARG12835	PARG12837	PARG12838	PARG12839
PARG12840	PARG12841	PARG12842	PARG12843	PARG12844	PARG12845	PARG12846
PARG12847	PARG12848	PARG12849	PARG12850	PARG12851	PARG12852	PARG12853
PARG12854	PARG12855	PARG12856	PARG12857	PARG12858	PARG12859	PARG12860
PARG12861	PARG12862	PARG12863	PARG12864	PARG12865	PARG12866	PARG12867
PARG12868	PARG12870	PARG12871	PARG12872	PARG12873	PARG12874	PARG12875
PARG12876	PARG12877	PARG12878	PARG12879	PARG12880	PARG12881	PARG12882
PARG12883	PARG12884	PARG12885	PARG12886	PARG12887	PARG12888	PARG12889
PARG12890	PARG12891	PARG12893	PARG12894	PARG12895	PARG12896	PARG12897
PARG12898	PARG12900	PARG12901	PARG12902	PARG12903	PARG12904	PARG12905
PARG12906	PARG12907	PARG12908	PARG12910	PARG12911	PARG12912	PARG12913

PARG12914	PARG12916	PARG12917	PARG12918	PARG12919	PARG12920	PARG12921
PARG12922	PARG12923	PARG12924	PARG12925	PARG12926	PARG12927	PARG12928
PARG12929	PARG12930	PARG12931	PARG12932	PARG12933	PARG12934	PARG12935
PARG12936	PARG12937	PARG12938	PARG12939	PARG12940	PARG12941	PARG12942
PARG12943	PARG12944	PARG12945	PARG12946	PARG12947	PARG12948	PARG12949
PARG12950	PARG12951	PARG12952	PARG12953	PARG12954	PARG12955	PARG12956
PARG12957	PARG12958	PARG12959	PARG12960	PARG12961	PARG12962	PARG12963
PARG12964	PARG12965	PARG12966	PARG12968	PARG12969	PARG12970	PARG12971
PARG12972	PARG12973	PARG12974	PARG12975	PARG12976	PARG12977	PARG12978
PARG12979	PARG12980	PARG12981	PARG12983	PARG12985	PARG12986	PARG12987
PARG12988	PARG12989	PARG12990	PARG12991	PARG12992	PARG12993	PARG12995
PARG12996	PARG12997	PARG12998	PARG12999	PARG13000	PARG13001	PARG13002
PARG13003	PARG13004	PARG13005	PARG13006	PARG13009	PARG13011	PARG13012
PARG13016	PARG13018	PARG13021	PARG13022	PARG13023	PARG13024	PARG13025
PARG13026	PARG13027	PARG13028	PARG13029	PARG13031	PARG13032	PARG13033
PARG13034	PARG13035	PARG13036	PARG13037	PARG13038	PARG13039	PARG13040
PARG13041	PARG13042	PARG13043	PARG13044	PARG13046	PARG13047	PARG13048
PARG13049	PARG13050	PARG13051	PARG13052	PARG13053	PARG13054	PARG13055
PARG13056	PARG13057	PARG13058	PARG13059	PARG13060	PARG13061	PARG13062
PARG13063	PARG13064	PARG13065	PARG13066	PARG13067	PARG13068	PARG13069
PARG13070	PARG13071	PARG13072	PARG13073	PARG13074	PARG13076	PARG13077
PARG13079	PARG13080	PARG13081	PARG13082	PARG13083	PARG13084	PARG13085
PARG13086	PARG13087	PARG13088	PARG13089	PARG13090	PARG13091	PARG13092
PARG13093	PARG13094	PARG13095	PARG13096	PARG13097	PARG13098	PARG13099
PARG13100	PARG13102	PARG13103	PARG13104	PARG13105	PARG13106	PARG13107
PARG13108	PARG13109	PARG13110	PARG13111	PARG13112	PARG13113	PARG13114
PARG13115	PARG13116	PARG13117	PARG13118	PARG13120	PARG13121	PARG13123
PARG13125	PARG13126	PARG13127	PARG13128	PARG13130	PARG13131	PARG13132
PARG13133	PARG13134	PARG13135	PARG13136	PARG13137	PARG13138	PARG13139
PARG13140	PARG13141	PARG13142	PARG13144	PARG13145	PARG13146	PARG13147
PARG13149	PARG13150	PARG13151	PARG13152	PARG13153	PARG13154	PARG13155
PARG13156	PARG13157	PARG13158	PARG13159	PARG13162	PARG13163	PARG13166
PARG13167	PARG13169	PARG13174	PARG13175	PARG13177	PARG13178	PARG13181
PARG13185	PARG13186	PARG13187	PARG13188	PARG13191	PARG13192	PARG13193
PARG13194	PARG13196	PARG13197	PARG13198	PARG13199	PARG13200	PARG13201
PARG13202	PARG13203	PARG13204	PARG13205	PARG13206	PARG13207	PARG13208
PARG13209	PARG13210	PARG13211	PARG13212	PARG13213	PARG13214	PARG13216
PARG13217	PARG13218	PARG13219	PARG13220	PARG13221	PARG13222	PARG13223
PARG13224	PARG13225	PARG13226	PARG13228	PARG13229	PARG13230	PARG13231
PARG13232	PARG13233	PARG13234	PARG13235	PARG13236	PARG13237	PARG13239
PARG13240	PARG13241	PARG13242	PARG13243	PARG13244	PARG13245	PARG13246
PARG13247	PARG13248	PARG13249	PARG13253	PARG13254	PARG13255	PARG13256
PARG13257	PARG13258	PARG13259	PARG13260	PARG13261	PARG13262	PARG13263
PARG13264	PARG13265	PARG13266	PARG13267	PARG13268	PARG13269	PARG13270
PARG13271	PARG13272	PARG13273	PARG13275	PARG13279	PARG13280	PARG13281
PARG13283	PARG13284	PARG13285	PARG13286	PARG13287	PARG13288	PARG13291

PARG13294	PARG13295	PARG13296	PARG13297	PARG13298	PARG13299	PARG13301
PARG13303	PARG13304	PARG13306	PARG13307	PARG13309	PARG13310	PARG13313
PARG13314	PARG13315	PARG13316	PARG13317	PARG13318	PARG13319	PARG13320
PARG13322	PARG13323	PARG13324	PARG13325	PARG13326	PARG13327	PARG13328
PARG13329	PARG13330	PARG13331	PARG13332	PARG13334	PARG13335	PARG13336
PARG13337	PARG13338	PARG13339	PARG13340	PARG13341	PARG13342	PARG13343
PARG13344	PARG13345	PARG13346	PARG13349	PARG13350	PARG13351	PARG13352
PARG13353	PARG13355	PARG13356	PARG13357	PARG13358	PARG13359	PARG13360
PARG13361	PARG13362	PARG13363	PARG13364	PARG13375	PARG13376	PARG13377
PARG13378	PARG13379	PARG13380	PARG13381	PARG13382	PARG13383	PARG13384
PARG13386	PARG13387	PARG13388	PARG13389	PARG13390	PARG13392	PARG13393
PARG13394	PARG13395	PARG13396	PARG13397	PARG13398	PARG13399	PARG13400
PARG13401	PARG13402	PARG13403	PARG13405	PARG13406	PARG13407	PARG13408
PARG13409	PARG13410	PARG13412	PARG13413	PARG13414	PARG13415	PARG13416
PARG13417	PARG13418	PARG13419	PARG13420	PARG13422	PARG13423	PARG13424
PARG13425	PARG13426	PARG13427	PARG13428	PARG13429	PARG13430	PARG13431
PARG13432	PARG13433	PARG13434	PARG13435	PARG13436	PARG13437	PARG13438
PARG13439	PARG13440	PARG13441	PARG13443	PARG13444	PARG13445	PARG13447
PARG13448	PARG13449	PARG13453	PARG13454	PARG13457	PARG13458	PARG13459
PARG13460	PARG13462	PARG13464	PARG13465	PARG13466	PARG13467	PARG13468
PARG13470	PARG13474	PARG13475	PARG13476	PARG13478	PARG13479	PARG13480
PARG13481	PARG13482	PARG13483	PARG13484	PARG13485	PARG13486	PARG13490
PARG13491	PARG13492	PARG13493	PARG13495	PARG13496	PARG13497	PARG13498
PARG13499	PARG13500	PARG13501	PARG13502	PARG13503	PARG13504	PARG13505
PARG13506	PARG13507	PARG13510	PARG13511	PARG13512	PARG13513	PARG13514
PARG13515	PARG13516	PARG13517	PARG13518	PARG13519	PARG13520	PARG13521
PARG13522	PARG13523	PARG13524	PARG13525	PARG13526	PARG13528	PARG13529
PARG13530	PARG13531	PARG13532	PARG13533	PARG13534	PARG13535	PARG13536
PARG13537	PARG13538	PARG13542	PARG13543	PARG13544	PARG13546	PARG13547
PARG13548	PARG13549	PARG13550	PARG13551	PARG13552	PARG13553	PARG13559
PARG13560	PARG13562	PARG13563	PARG13565	PARG13566	PARG13567	PARG13568
PARG13571	PARG13572	PARG13573	PARG13574	PARG13575	PARG13578	PARG13579
PARG13580	PARG13581	PARG13582	PARG13583	PARG13584	PARG13585	PARG13586
PARG13587	PARG13588	PARG13589	PARG13590	PARG13591	PARG13592	PARG13593
PARG13594	PARG13595	PARG13596	PARG13598	PARG13599	PARG13600	PARG13601
PARG13602	PARG13603	PARG13604	PARG13605	PARG13606	PARG13609	PARG13610
PARG13612	PARG13613	PARG13614	PARG13615	PARG13616	PARG13618	PARG13619
PARG13620	PARG13621	PARG13622	PARG13623	PARG13624	PARG13625	PARG13626
PARG13627	PARG13628	PARG13629	PARG13630	PARG13632	PARG13633	PARG13634
PARG13635	PARG13636	PARG13637	PARG13638	PARG13639	PARG13640	PARG13641
PARG13642	PARG13643	PARG13644	PARG13645	PARG13646	PARG13647	PARG13649
PARG13650	PARG13652	PARG13653	PARG13654	PARG13655	PARG13656	PARG13660
PARG13661	PARG13662	PARG13663	PARG13670	PARG13671	PARG13672	PARG13673
PARG13674	PARG13675	PARG13677	PARG13679	PARG13680	PARG13681	PARG13682
PARG13683	PARG13684	PARG13685	PARG13687	PARG13688	PARG13689	PARG13690
PARG13691	PARG13692	PARG13693	PARG13695	PARG13697	PARG13698	PARG13700

PARG13701	PARG13702	PARG13703	PARG13708	PARG13709	PARG13711	PARG13713
PARG13714	PARG13715	PARG13716	PARG13717	PARG13718	PARG13719	PARG13720
PARG13721	PARG13722	PARG13723	PARG13724	PARG13726	PARG13727	PARG13729
PARG13730	PARG13731	PARG13732	PARG13733	PARG13734	PARG13735	PARG13736
PARG13737	PARG13738	PARG13739	PARG13741	PARG13742	PARG13743	PARG13744
PARG13745	PARG13746	PARG13747	PARG13748	PARG13749	PARG13750	PARG13752
PARG13753	PARG13754	PARG13755	PARG13756	PARG13757	PARG13758	PARG13760
PARG13761	PARG13764	PARG13765	PARG13766	PARG13767	PARG13768	PARG13769
PARG13770	PARG13771	PARG13772	PARG13773	PARG13774	PARG13775	PARG13776
PARG13777	PARG13778	PARG13779	PARG13780	PARG13781	PARG13782	PARG13783
PARG13784	PARG13785	PARG13786	PARG13787	PARG13788	PARG13789	PARG13790
PARG13791	PARG13792	PARG13793	PARG13794	PARG13795	PARG13796	PARG13797
PARG13798	PARG13800	PARG13803	PARG13804	PARG13805	PARG13806	PARG13807
PARG13808	PARG13809	PARG13810	PARG13811	PARG13812	PARG13813	PARG13815
PARG13816	PARG13818	PARG13819	PARG13820	PARG13821	PARG13822	PARG13823
PARG13824	PARG13825	PARG13826	PARG13827	PARG13828	PARG13830	PARG13831
PARG13832	PARG13833	PARG13834	PARG13835	PARG13836	PARG13837	PARG13838
PARG13839	PARG13840	PARG13841	PARG13842	PARG13843	PARG13844	PARG13845
PARG13846	PARG13847	PARG13849	PARG13852	PARG13853	PARG13854	PARG13855
PARG13856	PARG13857	PARG13858	PARG13859	PARG13860	PARG13861	PARG13862
PARG13863	PARG13864	PARG13865	PARG13866	PARG13867	PARG13868	PARG13869
PARG13870	PARG13871	PARG13872	PARG13873	PARG13874	PARG13875	PARG13876
PARG13877	PARG13878	PARG13879	PARG13880	PARG13881	PARG13882	PARG13883
PARG13884	PARG13885	PARG13886	PARG13887	PARG13888	PARG13889	PARG13890
PARG13892	PARG13893	PARG13894	PARG13895	PARG13897	PARG13898	PARG13899
PARG13900	PARG13901	PARG13902	PARG13903	PARG13904	PARG13905	PARG13907
PARG13908	PARG13909	PARG13910	PARG13911	PARG13912	PARG13913	PARG13914
PARG13915	PARG13916	PARG13917	PARG13918	PARG13919	PARG13920	PARG13921
PARG13922	PARG13923	PARG13924	PARG13925	PARG13926	PARG13927	PARG13929
PARG13930	PARG13931	PARG13932	PARG13933	PARG13934	PARG13936	PARG13937
PARG13938	PARG13939	PARG13940	PARG13941	PARG13942	PARG13943	PARG13944
PARG13945	PARG13946	PARG13947	PARG13948	PARG13949	PARG13950	PARG13951
PARG13952	PARG13953	PARG13954	PARG13955	PARG13956	PARG13957	PARG13958
PARG13959	PARG13960	PARG13962	PARG13963	PARG13964	PARG13965	PARG13966
PARG13967	PARG13968	PARG13969	PARG13970	PARG13971	PARG13972	PARG13973
PARG13974	PARG13975	PARG13976	PARG13977	PARG13978	PARG13979	PARG13980
PARG13981	PARG13982	PARG13986	PARG13987	PARG13990	PARG13991	PARG13992
PARG13993	PARG13994	PARG13995	PARG13996	PARG13997	PARG13998	PARG13999
PARG14000	PARG14001	PARG14002	PARG14003	PARG14005	PARG14008	PARG14010
PARG14011	PARG14012	PARG14013	PARG14014	PARG14015	PARG14016	PARG14017
PARG14019	PARG14020	PARG14024	PARG14025	PARG14026	PARG14027	PARG14028
PARG14029	PARG14030	PARG14032	PARG14033	PARG14034	PARG14035	PARG14036
PARG14037	PARG14038	PARG14039	PARG14040	PARG14041	PARG14042	PARG14043
PARG14044	PARG14045	PARG14046	PARG14047	PARG14049	PARG14050	PARG14051
PARG14052	PARG14053	PARG14056	PARG14057	PARG14058	PARG14059	PARG14061
PARG14062	PARG14063	PARG14064	PARG14065	PARG14066	PARG14067	PARG14068

PARG14070	PARG14071	PARG14073	PARG14075	PARG14077	PARG14078	PARG14079
PARG14082	PARG14083	PARG14084	PARG14085	PARG14086	PARG14087	PARG14089
PARG14090	PARG14091	PARG14092	PARG14093	PARG14094	PARG14100	PARG14101
PARG14102	PARG14103	PARG14104	PARG14105	PARG14106	PARG14107	PARG14108
PARG14109	PARG14110	PARG14112	PARG14114	PARG14117	PARG14118	PARG14119
PARG14120	PARG14121	PARG14122	PARG14123	PARG14124	PARG14125	PARG14126
PARG14127	PARG14128	PARG14129	PARG14130	PARG14131	PARG14132	PARG14133
PARG14134	PARG14135	PARG14136	PARG14137	PARG14138	PARG14139	PARG14140
PARG14141	PARG14142	PARG14143	PARG14144	PARG14145	PARG14146	PARG14147
PARG14148	PARG14149	PARG14150	PARG14151	PARG14152	PARG14153	PARG14154
PARG14155	PARG14157	PARG14158	PARG14159	PARG14160	PARG14161	PARG14162
PARG14163	PARG14167	PARG14168	PARG14169	PARG14170	PARG14171	PARG14173
PARG14174	PARG14175	PARG14177	PARG14178	PARG14179	PARG14180	PARG14182
PARG14183	PARG14184	PARG14185	PARG14186	PARG14187	PARG14188	PARG14189
PARG14190	PARG14192	PARG14193	PARG14194	PARG14196	PARG14197	PARG14200
PARG14202	PARG14203	PARG14204	PARG14206	PARG14207	PARG14208	PARG14209
PARG14210	PARG14211	PARG14212	PARG14213	PARG14214	PARG14217	PARG14218
PARG14219	PARG14221	PARG14224	PARG14229	PARG14231	PARG14232	PARG14233
PARG14234	PARG14237	PARG14238	PARG14239	PARG14240	PARG14241	PARG14246
PARG14247	PARG14248	PARG14249	PARG14250	PARG14251	PARG14252	PARG14253
PARG14254	PARG14255	PARG14256	PARG14257	PARG14258	PARG14259	PARG14263
PARG14264	PARG14265	PARG14267	PARG14268	PARG14269	PARG14270	PARG14273
PARG14274	PARG14275	PARG14276	PARG14277	PARG14278	PARG14279	PARG14280
PARG14281	PARG14282	PARG14283	PARG14285	PARG14286	PARG14290	PARG14291
PARG14292	PARG14293	PARG14295	PARG14296	PARG14297	PARG14299	PARG14302
PARG14303	PARG14306	PARG14308	PARG14310	PARG14312	PARG14313	PARG14314
PARG14315	PARG14316	PARG14317	PARG14320	PARG14321	PARG14322	PARG14323
PARG14324	PARG14325	PARG14328	PARG14329	PARG14330	PARG14331	PARG14332
PARG14333	PARG14334	PARG14335	PARG14336	PARG14338	PARG14339	PARG14340
PARG14341	PARG14342	PARG14344	PARG14345	PARG14346	PARG14347	PARG14350
PARG14356	PARG14357	PARG14362	PARG14366	PARG14374	PARG14377	PARG14378
PARG14379	PARG14380	PARG14381	PARG14382	PARG14383	PARG14384	PARG14385
PARG14387	PARG14388	PARG14389	PARG14390	PARG14392	PARG14393	PARG14394
PARG14395	PARG14396	PARG14397	PARG14398	PARG14400	PARG14401	PARG14402
PARG14403	PARG14404	PARG14409	PARG14411	PARG14413	PARG14414	PARG14415
PARG14416	PARG14417	PARG14419	PARG14420	PARG14421	PARG14422	PARG14423
PARG14424	PARG14425	PARG14426	PARG14427	PARG14428	PARG14429	PARG14430
PARG14432	PARG14434	PARG14436	PARG14437	PARG14438	PARG14440	PARG14441
PARG14442	PARG14443	PARG14444	PARG14446	PARG14447	PARG14448	PARG14449
PARG14450	PARG14452	PARG14453	PARG14454	PARG14455	PARG14456	PARG14460
PARG14461	PARG14462	PARG14463	PARG14466	PARG14467	PARG14469	PARG14471
PARG14472	PARG14473	PARG14474	PARG14475	PARG14476	PARG14480	PARG14482
PARG14483	PARG14484	PARG14486	PARG14488	PARG14490	PARG14492	PARG14493
PARG14494	PARG14495	PARG14496	PARG14498	PARG14502	PARG14503	PARG14504
PARG14506	PARG14508	PARG14509	PARG14510	PARG14511	PARG14512	PARG14514
PARG14516	PARG14519	PARG14520	PARG14522	PARG14523	PARG14524	PARG14525

PARG14526	PARG14527	PARG14528	PARG14530	PARG14533	PARG14534	PARG14535
PARG14536	PARG14539	PARG14540	PARG14541	PARG14543	PARG14545	PARG14546
PARG14548	PARG14558	PARG14559	PARG14560	PARG14573	PARG14577	PARG14587
PARG14588	PARG14590	PARG14597	PARG14598	PARG14600	PARG14601	PARG14603
PARG14604	PARG14605	PARG14606	PARG14611	PARG14614	PARG14617	PARG14618
PARG14619	PARG14621	PARG14626	PARG14628	PARG14629	PARG14630	PARG14631
PARG14632	PARG14634	PARG14635	PARG14636	PARG14638	PARG14639	PARG14640
PARG14641	PARG14642	PARG14643	PARG14644	PARG14645	PARG14646	PARG14647
PARG14648	PARG14649	PARG14651	PARG14652	PARG14653	PARG14654	PARG14655
PARG14656	PARG14657	PARG14658	PARG14659	PARG14674	PARG14675	PARG14678
PARG14686	PARG14702	PARG14705	PARG14706	PARG14709	PARG14710	PARG14720
PARG14721	PARG14722	PARG14727	PARG14731	PARG14732	PARG14734	PARG14736
PARG14737	PARG14742	PARG14743	PARG14744	PARG14745	PARG14746	PARG14749
PARG14751	PARG14758	PARG14762	PARG14763	PARG14764	PARG14765	PARG14766
PARG14769	PARG14770	PARG14774	PARG14775	PARG14777	PARG14778	PARG14779
PARG14780	PARG14787	PARG14793	PARG14794	PARG14796	PARG14797	PARG14802
PARG14806	PARG14807	PARG14808	PARG14810	PARG14811	PARG14812	PARG14816
PARG14819	PARG14820	PARG14821	PARG14822	PARG14823	PARG14827	PARG14828
PARG14830	PARG14831	PARG14832	PARG14834	PARG14835	PARG14837	PARG14838
PARG14839	PARG14843	PARG14848	PARG14852	PARG14857	PARG14858	PARG14860
PARG14870	PARG14872	PARG14874	PARG14877	PARG14878	PARG14883	PARG14885
PARG14886	PARG14903	PARG14904	PARG14907	PARG14912	PARG14913	PARG14917
PARG14918	PARG14919	PARG14920	PARG14921	PARG14923	PARG14925	PARG14926
PARG14930	PARG14931	PARG14932	PARG14933	PARG14936	PARG14940	PARG14941
PARG14942	PARG14943	PARG14945	PARG14953	PARG14954	PARG14955	PARG14956
PARG14960	PARG14963	PARG14964	PARG14965	PARG14967	PARG14970	PARG14975
PARG14976	PARG14977	PARG14978	PARG14979	PARG14980	PARG14981	PARG14983
PARG14984	PARG14985	PARG14986	PARG14987	PARG14988	PARG14991	PARG14996
PARG14998	PARG15000	PARG15007	PARG15009	PARG15010	PARG15011	PARG15013
PARG15014	PARG15015	PARG15019	PARG15022	PARG15025	PARG15030	PARG15031
PARG15032	PARG15033	PARG15038	PARG15039	PARG15040	PARG15042	PARG15043
PARG15044	PARG15056	PARG15057	PARG15058	PARG15059	PARG15060	PARG15066
PARG15072	PARG15076	PARG15079	PARG15082	PARG15083	PARG15085	PARG15086
PARG15087	PARG15088	PARG15089	PARG15090	PARG15091	PARG15093	PARG15094
PARG15096	PARG15097	PARG15098	PARG15100	PARG15101	PARG15102	PARG15104
PARG15105	PARG15106	PARG15107	PARG15108	PARG15112	PARG15113	PARG15114
PARG15115	PARG15116	PARG15117	PARG15119	PARG15120	PARG15121	PARG15122
PARG15123	PARG15124	PARG15127	PARG15129	PARG15131	PARG15133	PARG15134
PARG15135	PARG15137	PARG15138	PARG15139	PARG15140	PARG15141	PARG15142
PARG15143	PARG15144	PARG15145	PARG15147	PARG15148	PARG15150	PARG15151
PARG15152	PARG15155	PARG15156	PARG15157	PARG15158	PARG15159	PARG15160
PARG15162	PARG15163	PARG15164	PARG15165	PARG15166	PARG15167	PARG15169
PARG15170	PARG15173	PARG15174	PARG15175	PARG15176	PARG15178	PARG15180
PARG15181	PARG15182	PARG15185	PARG15189	PARG15190	PARG15191	PARG15192
PARG15193	PARG15195	PARG15198	PARG15199	PARG15201	PARG15203	PARG15204
PARG15205	PARG15206	PARG15207	PARG15209	PARG15210	PARG15211	PARG15212

PARG15214	PARG15215	PARG15216	PARG15217	PARG15218	PARG15219	PARG15220
PARG15221	PARG15223	PARG15225	PARG15227	PARG15229	PARG15231	PARG15232
PARG15233	PARG15234	PARG15235	PARG15239	PARG15245	PARG15248	PARG15249
PARG15250	PARG15251	PARG15252	PARG15256	PARG15257	PARG15258	PARG15259
PARG15260	PARG15261	PARG15262	PARG15263	PARG15264	PARG15266	PARG15267
PARG15269	PARG15272	PARG15273	PARG15274	PARG15275	PARG15276	PARG15279
PARG15280	PARG15282	PARG15283	PARG15284	PARG15285	PARG15286	PARG15288
PARG15289	PARG15291	PARG15292	PARG15294	PARG15296	PARG15303	PARG15304
PARG15305	PARG15306	PARG15307	PARG15308	PARG15309	PARG15310	PARG15311
PARG15312	PARG15313	PARG15314	PARG15315	PARG15316	PARG15317	PARG15318
PARG15319	PARG15320	PARG15321	PARG15322	PARG15324	PARG15325	PARG15327
PARG15328	PARG15329	PARG15330	PARG15331	PARG15332	PARG15333	PARG15334
PARG15335	PARG15336	PARG15337	PARG15338	PARG15339	PARG15340	PARG15341
PARG15342	PARG15343	PARG15344	PARG15345	PARG15346	PARG15347	PARG15348
PARG15349	PARG15350	PARG15351	PARG15352	PARG15355	PARG15357	PARG15358
PARG15359	PARG15360	PARG15362	PARG15363	PARG15364	PARG15366	PARG15367
PARG15368	PARG15371	PARG15372	PARG15375	PARG15376	PARG15377	PARG15378
PARG15381	PARG15382	PARG15383	PARG15384	PARG15385	PARG15386	PARG15387
PARG15388	PARG15391	PARG15392	PARG15393	PARG15394	PARG15395	PARG15396
PARG15397	PARG15398	PARG15399	PARG15400	PARG15401	PARG15402	PARG15404
PARG15405	PARG15406	PARG15407	PARG15408	PARG15409	PARG15410	PARG15411
PARG15413	PARG15414	PARG15415	PARG15418	PARG15422	PARG15423	PARG15424
PARG15425	PARG15426	PARG15427	PARG15428	PARG15430	PARG15431	PARG15433
PARG15434	PARG15435	PARG15436	PARG15437	PARG15438	PARG15439	PARG15440
PARG15442	PARG15443	PARG15444	PARG15445	PARG15446	PARG15447	PARG15448
PARG15449	PARG15450	PARG15451	PARG15452	PARG15454	PARG15455	PARG15456
PARG15457	PARG15458	PARG15460	PARG15461	PARG15462	PARG15463	PARG15464
PARG15468	PARG15469	PARG15470	PARG15473	PARG15476	PARG15477	PARG15478
PARG15480	PARG15483	PARG15484	PARG15485	PARG15489	PARG15491	PARG15492
PARG15493	PARG15494	PARG15495	PARG15496	PARG15497	PARG15498	PARG15499
PARG15501	PARG15502	PARG15503	PARG15504	PARG15505	PARG15506	PARG15508
PARG15509	PARG15510	PARG15511	PARG15512	PARG15513	PARG15514	PARG15515
PARG15516	PARG15517	PARG15518	PARG15519	PARG15520	PARG15521	PARG15522
PARG15523	PARG15524	PARG15525	PARG15526	PARG15527	PARG15530	PARG15531
PARG15532	PARG15533	PARG15534	PARG15535	PARG15536	PARG15538	PARG15539
PARG15540	PARG15544	PARG15546	PARG15547	PARG15548	PARG15549	PARG15550
PARG15551	PARG15552	PARG15553	PARG15554	PARG15555	PARG15556	PARG15557
PARG15558	PARG15559	PARG15562	PARG15564	PARG15565	PARG15566	PARG15567
PARG15568	PARG15569	PARG15571	PARG15573	PARG15575	PARG15576	PARG15577
PARG15578	PARG15579	PARG15580	PARG15581	PARG15588	PARG15589	PARG15590
PARG15591	PARG15592	PARG15595	PARG15596	PARG15598	PARG15599	PARG15600
PARG15601	PARG15603	PARG15604	PARG15605	PARG15606	PARG15607	PARG15608
PARG15610	PARG15611	PARG15612	PARG15613	PARG15616	PARG15618	PARG15619
PARG15620	PARG15621	PARG15622	PARG15623	PARG15624	PARG15625	PARG15626
PARG15628	PARG15629	PARG15631	PARG15634	PARG15636	PARG15637	PARG15638
PARG15639	PARG15640	PARG15641	PARG15643	PARG15644	PARG15645	PARG15646

PARG15647	PARG15648	PARG15649	PARG15650	PARG15653	PARG15655	PARG15656
PARG15657	PARG15658	PARG15659	PARG15660	PARG15661	PARG15662	PARG15663
PARG15664	PARG15665	PARG15666	PARG15673	PARG15674	PARG15675	PARG15676
PARG15677	PARG15678	PARG15679	PARG15680	PARG15681	PARG15682	PARG15683
PARG15684	PARG15685	PARG15686	PARG15687	PARG15688	PARG15689	PARG15691
PARG15692	PARG15697	PARG15699	PARG15700	PARG15701	PARG15708	PARG15709
PARG15710	PARG15713	PARG15714	PARG15715	PARG15716	PARG15717	PARG15718
PARG15719	PARG15720	PARG15721	PARG15722	PARG15723	PARG15724	PARG15725
PARG15726	PARG15727	PARG15728	PARG15729	PARG15730	PARG15731	PARG15732
PARG15733	PARG15734	PARG15735	PARG15736	PARG15737	PARG15738	PARG15739
PARG15740	PARG15741	PARG15742	PARG15743	PARG15744	PARG15745	PARG15746
PARG15747	PARG15748	PARG15749	PARG15751	PARG15752	PARG15754	PARG15755
PARG15756	PARG15757	PARG15758	PARG15759	PARG15760	PARG15761	PARG15762
PARG15763	PARG15764	PARG15765	PARG15766	PARG15767	PARG15769	PARG15770
PARG15771	PARG15772	PARG15773	PARG15774	PARG15775	PARG15776	PARG15777
PARG15778	PARG15779	PARG15780	PARG15781	PARG15782	PARG15783	PARG15784
PARG15785	PARG15786	PARG15787	PARG15788	PARG15789	PARG15790	PARG15791
PARG15792	PARG15793	PARG15794	PARG15795	PARG15796	PARG15797	PARG15798
PARG15799	PARG15800	PARG15802	PARG15803	PARG15804	PARG15805	PARG15806
PARG15807	PARG15808	PARG15809	PARG15810	PARG15811	PARG15812	PARG15813
PARG15814	PARG15815	PARG15816	PARG15817	PARG15818	PARG15819	PARG15820
PARG15821	PARG15822	PARG15825	PARG15826	PARG15829	PARG15832	PARG15833
PARG15834	PARG15835	PARG15836	PARG15837	PARG15838	PARG15839	PARG15840
PARG15841	PARG15842	PARG15843	PARG15844	PARG15845	PARG15846	PARG15847
PARG15848	PARG15849	PARG15850	PARG15851	PARG15852	PARG15855	PARG15856
PARG15857	PARG15858	PARG15859	PARG15860	PARG15861	PARG15862	PARG15863
PARG15864	PARG15865	PARG15866	PARG15867	PARG15868	PARG15869	PARG15870
PARG15871	PARG15872	PARG15873	PARG15874	PARG15875	PARG15876	PARG15877
PARG15878	PARG15879	PARG15880	PARG15883	PARG15884	PARG15885	PARG15887
PARG15888	PARG15889	PARG15890	PARG15891	PARG15892	PARG15893	PARG15895
PARG15897	PARG15898	PARG15900	PARG15902	PARG15903	PARG15904	PARG15907
PARG15909	PARG15910	PARG15911	PARG15912	PARG15913	PARG15915	PARG15917
PARG15918	PARG15919	PARG15921	PARG15922	PARG15923	PARG15924	PARG15925
PARG15926	PARG15927	PARG15929	PARG15930	PARG15931	PARG15932	PARG15933
PARG15934	PARG15935	PARG15936	PARG15937	PARG15938	PARG15939	PARG15941
PARG15942	PARG15943	PARG15944	PARG15946	PARG15947	PARG15948	PARG15949
PARG15950	PARG15951	PARG15952	PARG15953	PARG15954	PARG15955	PARG15956
PARG15957	PARG15958	PARG15959	PARG15960	PARG15961	PARG15962	PARG15963
PARG15964	PARG15965	PARG15972	PARG15973	PARG15974	PARG15975	PARG15976
PARG15977	PARG15978	PARG15979	PARG15980	PARG15981	PARG15982	PARG15983
PARG15985	PARG15986	PARG15987	PARG15990	PARG15991	PARG15992	PARG15993
PARG15994	PARG15995	PARG15996	PARG15997	PARG15998	PARG16000	PARG16001
PARG16004	PARG16005	PARG16007	PARG16008	PARG16009	PARG16010	PARG16011
PARG16012	PARG16013	PARG16014	PARG16017	PARG16019	PARG16020	PARG16021
PARG16022	PARG16023	PARG16024	PARG16025	PARG16026	PARG16029	PARG16031
PARG16032	PARG16033	PARG16034	PARG16035	PARG16036	PARG16037	PARG16038

PARG16039	PARG16041	PARG16043	PARG16044	PARG16045	PARG16046	PARG16047
PARG16048	PARG16049	PARG16053	PARG16054	PARG16055	PARG16056	PARG16057
PARG16058	PARG16059	PARG16060	PARG16061	PARG16062	PARG16064	PARG16065
PARG16066	PARG16067	PARG16068	PARG16069	PARG16070	PARG16071	PARG16072
PARG16073	PARG16074	PARG16075	PARG16077	PARG16078	PARG16079	PARG16080
PARG16081	PARG16082	PARG16083	PARG16084	PARG16085	PARG16086	PARG16087
PARG16088	PARG16089	PARG16090	PARG16091	PARG16092	PARG16093	PARG16094
PARG16098	PARG16099	PARG16100	PARG16101	PARG16102	PARG16103	PARG16104
PARG16105	PARG16106	PARG16108	PARG16109	PARG16110	PARG16111	PARG16112
PARG16113	PARG16114	PARG16116	PARG16117	PARG16118	PARG16119	PARG16120
PARG16121	PARG16122	PARG16123	PARG16124	PARG16125	PARG16126	PARG16127
PARG16128	PARG16129	PARG16132	PARG16133	PARG16134	PARG16136	PARG16138
PARG16139	PARG16140	PARG16142	PARG16143	PARG16144	PARG16145	PARG16148
PARG16151	PARG16152	PARG16153	PARG16154	PARG16155	PARG16156	PARG16160
PARG16161	PARG16162	PARG16163	PARG16164	PARG16166	PARG16167	PARG16168
PARG16169	PARG16170	PARG16171	PARG16172	PARG16173	PARG16174	PARG16175
PARG16176	PARG16177	PARG16178	PARG16179	PARG16180	PARG16181	PARG16182
PARG16183	PARG16184	PARG16185	PARG16188	PARG16189	PARG16190	PARG16191
PARG16192	PARG16193	PARG16194	PARG16195	PARG16197	PARG16198	PARG16199
PARG16200	PARG16201	PARG16202	PARG16203	PARG16204	PARG16205	PARG16206
PARG16207	PARG16208	PARG16209	PARG16210	PARG16211	PARG16212	PARG16213
PARG16214	PARG16215	PARG16216	PARG16217	PARG16218	PARG16219	PARG16220
PARG16221	PARG16222	PARG16223	PARG16224	PARG16225	PARG16226	PARG16227
PARG16228	PARG16229	PARG16230	PARG16231	PARG16232	PARG16233	PARG16235
PARG16236	PARG16237	PARG16238	PARG16240	PARG16241	PARG16243	PARG16244
PARG16245	PARG16246	PARG16247	PARG16248	PARG16249	PARG16254	PARG16255
PARG16256	PARG16257	PARG16258	PARG16259	PARG16260	PARG16261	PARG16262
PARG16264	PARG16266	PARG16269	PARG16271	PARG16272	PARG16274	PARG16276
PARG16278	PARG16279	PARG16280	PARG16284	PARG16285	PARG16287	PARG16288
PARG16289	PARG16291	PARG16292	PARG16293	PARG16294	PARG16296	PARG16298
PARG16299	PARG16300	PARG16301	PARG16302	PARG16303	PARG16304	PARG16305
PARG16306	PARG16307	PARG16308	PARG16309	PARG16310	PARG16311	PARG16312
PARG16314	PARG16315	PARG16316	PARG16319	PARG16321	PARG16322	PARG16323
PARG16325	PARG16326	PARG16330	PARG16332	PARG16337	PARG16338	PARG16339
PARG16342	PARG16343	PARG16344	PARG16345	PARG16346	PARG16347	PARG16348
PARG16349	PARG16351	PARG16352	PARG16353	PARG16354	PARG16355	PARG16357
PARG16358	PARG16359	PARG16361	PARG16362	PARG16363	PARG16364	PARG16365
PARG16366	PARG16367	PARG16369	PARG16371	PARG16374	PARG16377	PARG16379
PARG16380	PARG16381	PARG16382	PARG16383	PARG16384	PARG16385	PARG16386
PARG16387	PARG16388	PARG16389	PARG16392	PARG16393	PARG16395	PARG16396
PARG16397	PARG16398	PARG16399	PARG16401	PARG16402	PARG16403	PARG16404
PARG16405	PARG16406	PARG16412	PARG16413	PARG16414	PARG16415	PARG16416
PARG16418	PARG16419	PARG16420	PARG16421	PARG16423	PARG16424	PARG16425
PARG16426	PARG16428	PARG16429	PARG16431	PARG16432	PARG16433	PARG16434
PARG16435	PARG16436	PARG16437	PARG16438	PARG16439	PARG16440	PARG16441
PARG16442	PARG16443	PARG16446	PARG16447	PARG16449	PARG16450	PARG16451

PARG16453	PARG16454	PARG16455	PARG16456	PARG16457	PARG16460	PARG16461
PARG16462	PARG16465	PARG16466	PARG16467	PARG16468	PARG16471	PARG16472
PARG16474	PARG16475	PARG16476	PARG16478	PARG16479	PARG16480	PARG16482
PARG16483	PARG16484	PARG16485	PARG16486	PARG16490	PARG16491	PARG16492
PARG16493	PARG16494	PARG16495	PARG16496	PARG16497	PARG16499	PARG16500
PARG16501	PARG16502	PARG16504	PARG16505	PARG16507	PARG16508	PARG16509
PARG16511	PARG16512	PARG16514	PARG16515	PARG16516	PARG16518	PARG16521
PARG16523	PARG16524	PARG16525	PARG16526	PARG16529	PARG16530	PARG16531
PARG16532	PARG16533	PARG16534	PARG16535	PARG16536	PARG16537	PARG16538
PARG16539	PARG16540	PARG16541	PARG16546	PARG16547	PARG16548	PARG16553
PARG16554	PARG16555	PARG16557	PARG16558	PARG16560	PARG16561	PARG16562
PARG16563	PARG16564	PARG16565	PARG16566	PARG16567	PARG16568	PARG16569
PARG16571	PARG16572	PARG16573	PARG16574	PARG16575	PARG16576	PARG16577
PARG16582	PARG16584	PARG16586	PARG16587	PARG16588	PARG16590	PARG16592
PARG16593	PARG16597	PARG16606	PARG16607	PARG16608	PARG16611	PARG16612
PARG16613	PARG16614	PARG16615	PARG16616	PARG16618	PARG16619	PARG16620
PARG16621	PARG16622	PARG16623	PARG16624	PARG16625	PARG16628	PARG16629
PARG16630	PARG16632	PARG16634	PARG16635	PARG16636	PARG16637	PARG16638
PARG16640	PARG16641	PARG16642	PARG16643	PARG16645	PARG16646	PARG16647
PARG16648	PARG16649	PARG16651	PARG16652	PARG16653	PARG16655	PARG16656
PARG16657	PARG16658	PARG16659	PARG16661	PARG16662	PARG16664	PARG16665
PARG16666	PARG16668	PARG16669	PARG16670	PARG16671	PARG16674	PARG16675
PARG16676	PARG16677	PARG16678	PARG16679	PARG16680	PARG16681	PARG16683
PARG16685	PARG16686	PARG16687	PARG16688	PARG16689	PARG16690	PARG16693
PARG16694	PARG16695	PARG16696	PARG16697	PARG16699	PARG16700	PARG16701
PARG16702	PARG16703	PARG16704	PARG16712	PARG16713	PARG16714	PARG16716
PARG16717	PARG16718	PARG16720	PARG16721	PARG16726	PARG16727	PARG16732
PARG16734	PARG16735	PARG16739	PARG16740	PARG16742	PARG16745	PARG16746
PARG16747	PARG16748	PARG16749	PARG16752	PARG16753	PARG16754	PARG16755
PARG16757	PARG16759	PARG16760	PARG16762	PARG16763	PARG16764	PARG16766
PARG16767	PARG16768	PARG16769	PARG16770	PARG16776	PARG16777	PARG16779
PARG16780	PARG16781	PARG16782	PARG16784	PARG16785	PARG16786	PARG16787
PARG16788	PARG16789	PARG16791	PARG16792	PARG16793	PARG16794	PARG16795
PARG16796	PARG16805	PARG16809	PARG16811	PARG16817	PARG16820	PARG16821
PARG16822	PARG16825	PARG16828	PARG16831	PARG16834	PARG16835	PARG16836
PARG16841	PARG16843	PARG16845	PARG16846	PARG16848	PARG16849	PARG16850
PARG16852	PARG16854	PARG16855	PARG16857	PARG16859	PARG16860	PARG16869
PARG16871	PARG16872	PARG16873	PARG16874	PARG16875	PARG16878	PARG16879
PARG16880	PARG16881	PARG16884	PARG16887	PARG16888	PARG16889	PARG16890
PARG16892	PARG16895	PARG16896	PARG16901	PARG16908	PARG16909	PARG16911
PARG16915	PARG16917	PARG16918	PARG16920	PARG16921	PARG16923	PARG16924
PARG16931	PARG16933	PARG16936	PARG16937	PARG16941	PARG16942	PARG16943
PARG16944	PARG16950	PARG16951	PARG16952	PARG16953	PARG16954	PARG16958
PARG16960	PARG16961	PARG16962	PARG16964	PARG16967	PARG16970	PARG16975
PARG16977	PARG16980	PARG16981	PARG16982	PARG16985	PARG16986	PARG16987
PARG16993	PARG16994	PARG16996	PARG17003	PARG17013	PARG17018	PARG17019

PARG17020	PARG17026	PARG17030	PARG17031	PARG17044	PARG17049	PARG17050
PARG17051	PARG17052	PARG17053	PARG17055	PARG17056	PARG17058	PARG17060
PARG17075	PARG17076	PARG17078	PARG17087	PARG17088	PARG17092	PARG17094
PARG17095	PARG17096	PARG17097	PARG17098	PARG17099	PARG17100	PARG17102
PARG17103	PARG17104	PARG17108	PARG17109	PARG17110	PARG17114	PARG17115
PARG17120	PARG17121	PARG17123	PARG17125	PARG17127	PARG17128	PARG17129
PARG17130	PARG17138	PARG17139	PARG17142	PARG17143	PARG17146	PARG17148
PARG17149	PARG17150	PARG17151	PARG17152	PARG17154	PARG17155	PARG17157
PARG17158	PARG17159	PARG17160	PARG17161	PARG17162	PARG17163	PARG17167
PARG17168	PARG17170	PARG17173	PARG17174	PARG17175	PARG17176	PARG17177
PARG17178	PARG17182	PARG17183	PARG17184	PARG17186	PARG17187	PARG17188
PARG17189	PARG17190	PARG17191	PARG17192	PARG17193	PARG17195	PARG17196
PARG17197	PARG17198	PARG17203	PARG17206	PARG17207	PARG17208	PARG17211
PARG17221	PARG17225	PARG17232	PARG17237	PARG17245	PARG17246	PARG17247
PARG17248	PARG17249	PARG17250	PARG17251	PARG17253	PARG17254	PARG17255
PARG17260	PARG17268	PARG17269	PARG17270	PARG17272	PARG17279	PARG17289
PARG17292	PARG17293	PARG17295	PARG17296	PARG17297	PARG17298	PARG17302
PARG17306	PARG17307	PARG17314	PARG17315	PARG17316	PARG17321	PARG17322
PARG17323	PARG17324	PARG17326	PARG17327	PARG17328	PARG17329	PARG17330
PARG17331	PARG17332	PARG17336	PARG17338	PARG17339	PARG17340	PARG17346
PARG17347	PARG17348	PARG17349	PARG17350	PARG17351	PARG17352	PARG17353
PARG17356	PARG17357	PARG17360	PARG17364	PARG17365	PARG17370	PARG17375
PARG17379	PARG17383	PARG17384	PARG17390	PARG17393	PARG17394	PARG17395
PARG17396	PARG17399	PARG17404	PARG17407	PARG17408	PARG17411	PARG17413
PARG17414	PARG17415	PARG17416	PARG17418	PARG17420	PARG17421	PARG17422
PARG17425	PARG17426	PARG17427	PARG17428	PARG17429	PARG17433	PARG17434
PARG17439	PARG17441	PARG17442	PARG17443	PARG17444	PARG17448	PARG17450
PARG17452	PARG17454	PARG17455	PARG17457	PARG17458	PARG17459	PARG17463
PARG17464	PARG17470	PARG17471	PARG17472	PARG17473	PARG17474	PARG17475
PARG17481	PARG17482	PARG17483	PARG17485	PARG17487	PARG17488	PARG17491
PARG17493	PARG17494	PARG17495	PARG17496	PARG17497	PARG17498	PARG17500
PARG17501	PARG17503	PARG17509	PARG17510	PARG17511	PARG17512	PARG17513
PARG17514	PARG17515	PARG17516	PARG17517	PARG17519	PARG17522	PARG17523
PARG17526	PARG17528	PARG17529	PARG17530	PARG17531	PARG17532	PARG17533
PARG17535	PARG17536	PARG17537	PARG17538	PARG17539	PARG17541	PARG17542
PARG17543	PARG17544	PARG17545	PARG17548	PARG17550	PARG17551	PARG17555
PARG17557	PARG17558	PARG17560	PARG17561	PARG17562	PARG17563	PARG17564
PARG17565	PARG17571	PARG17573	PARG17574	PARG17575	PARG17576	PARG17577
PARG17587	PARG17592	PARG17593	PARG17594	PARG17595	PARG17597	PARG17598
PARG17599	PARG17600	PARG17601	PARG17602	PARG17603	PARG17604	PARG17605
PARG17606	PARG17607	PARG17608	PARG17609	PARG17610	PARG17611	PARG17612
PARG17614	PARG17615	PARG17616	PARG17617	PARG17620	PARG17621	PARG17623
PARG17624	PARG17625	PARG17626	PARG17627	PARG17628	PARG17629	PARG17630
PARG17631	PARG17632	PARG17636	PARG17637	PARG17638	PARG17639	PARG17640
PARG17642	PARG17643	PARG17644	PARG17645	PARG17647	PARG17648	PARG17651
PARG17652	PARG17653	PARG17654	PARG17655	PARG17656	PARG17658	PARG17661

PARG17662	PARG17663	PARG17667	PARG17671	PARG17673	PARG17675	PARG17676
PARG17677	PARG17681	PARG17682	PARG17683	PARG17684	PARG17685	PARG17686
PARG17688	PARG17690	PARG17691	PARG17693	PARG17694	PARG17696	PARG17697
PARG17698	PARG17699	PARG17701	PARG17702	PARG17703	PARG17704	PARG17705
PARG17706	PARG17707	PARG17708	PARG17710	PARG17711	PARG17712	PARG17713
PARG17716	PARG17717	PARG17718	PARG17719	PARG17720	PARG17721	PARG17723
PARG17724	PARG17725	PARG17726	PARG17727	PARG17728	PARG17729	PARG17730
PARG17731	PARG17732	PARG17733	PARG17734	PARG17735	PARG17736	PARG17737
PARG17738	PARG17739	PARG17740	PARG17741	PARG17743	PARG17744	PARG17745
PARG17746	PARG17748	PARG17749	PARG17750	PARG17752	PARG17753	PARG17754
PARG17756	PARG17757	PARG17758	PARG17759	PARG17760	PARG17761	PARG17762
PARG17763	PARG17764	PARG17765	PARG17768	PARG17769	PARG17770	PARG17771
PARG17772	PARG17774	PARG17775	PARG17776	PARG17777	PARG17778	PARG17779
PARG17780	PARG17781	PARG17783	PARG17787	PARG17788	PARG17789	PARG17790
PARG17792	PARG17793	PARG17794	PARG17795	PARG17796	PARG17797	PARG17799
PARG17802	PARG17804	PARG17806	PARG17807	PARG17808	PARG17809	PARG17810
PARG17811	PARG17812	PARG17814	PARG17815	PARG17816	PARG17817	PARG17818
PARG17819	PARG17820	PARG17821	PARG17823	PARG17824	PARG17825	PARG17826
PARG17827	PARG17829	PARG17830	PARG17831	PARG17832	PARG17834	PARG17835
PARG17837	PARG17838	PARG17840	PARG17841	PARG17842	PARG17848	PARG17856
PARG17857	PARG17858	PARG17859	PARG17860	PARG17861	PARG17862	PARG17863
PARG17866	PARG17871	PARG17876	PARG17877	PARG17878	PARG17881	PARG17882
PARG17883	PARG17885	PARG17886	PARG17887	PARG17888	PARG17889	PARG17890
PARG17891	PARG17892	PARG17893	PARG17894	PARG17895	PARG17896	PARG17897
PARG17898	PARG17899	PARG17901	PARG17902	PARG17903	PARG17904	PARG17905
PARG17906	PARG17907	PARG17908	PARG17909	PARG17910	PARG17911	PARG17912
PARG17913	PARG17914	PARG17915	PARG17916	PARG17917	PARG17918	PARG17919
PARG17922	PARG17925	PARG17928	PARG17929	PARG17932	PARG17933	PARG17934
PARG17935	PARG17936	PARG17937	PARG17938	PARG17939	PARG17940	PARG17941
PARG17942	PARG17943	PARG17944	PARG17945	PARG17946	PARG17947	PARG17948
PARG17949	PARG17950	PARG17951	PARG17952	PARG17953	PARG17954	PARG17955
PARG17956	PARG17957	PARG17958	PARG17959	PARG17960	PARG17961	PARG17962
PARG17963	PARG17964	PARG17966	PARG17967	PARG17968	PARG17969	PARG17970
PARG17972	PARG17973	PARG17974	PARG17975	PARG17976	PARG17977	PARG17979
PARG17980	PARG17982	PARG17983	PARG17984	PARG17985	PARG17986	PARG17987
PARG17989	PARG17991	PARG17992	PARG17993	PARG17994	PARG17995	PARG17996
PARG17998	PARG17999	PARG18000	PARG18001	PARG18004	PARG18005	PARG18006
PARG18007	PARG18008	PARG18009	PARG18010	PARG18012	PARG18013	PARG18014
PARG18017	PARG18018	PARG18019	PARG18020	PARG18021	PARG18022	PARG18023
PARG18025	PARG18026	PARG18027	PARG18030	PARG18033	PARG18034	PARG18038
PARG18039	PARG18045	PARG18046	PARG18047	PARG18048	PARG18049	PARG18051
PARG18052	PARG18053	PARG18054	PARG18055	PARG18056	PARG18057	PARG18058
PARG18059	PARG18060	PARG18061	PARG18062	PARG18063	PARG18064	PARG18065
PARG18066	PARG18067	PARG18068	PARG18069	PARG18071	PARG18072	PARG18074
PARG18075	PARG18076	PARG18077	PARG18078	PARG18079	PARG18080	PARG18081
PARG18082	PARG18083	PARG18084	PARG18086	PARG18088	PARG18089	PARG18090

PARG18091	PARG18092	PARG18093	PARG18094	PARG18095	PARG18096	PARG18097
PARG18098	PARG18099	PARG18100	PARG18101	PARG18102	PARG18103	PARG18104
PARG18105	PARG18106	PARG18107	PARG18108	PARG18109	PARG18110	PARG18111
PARG18112	PARG18113	PARG18114	PARG18115	PARG18116	PARG18117	PARG18118
PARG18119	PARG18120	PARG18122	PARG18125	PARG18128	PARG18129	PARG18130
PARG18132	PARG18133	PARG18134	PARG18135	PARG18137	PARG18138	PARG18139
PARG18140	PARG18141	PARG18142	PARG18143	PARG18144	PARG18145	PARG18146
PARG18147	PARG18148	PARG18149	PARG18150	PARG18151	PARG18152	PARG18153
PARG18154	PARG18155	PARG18156	PARG18157	PARG18158	PARG18159	PARG18160
PARG18161	PARG18162	PARG18166	PARG18167	PARG18168	PARG18171	PARG18172
PARG18173	PARG18174	PARG18175	PARG18177	PARG18178	PARG18179	PARG18180
PARG18181	PARG18183	PARG18184	PARG18185	PARG18186	PARG18187	PARG18188
PARG18189	PARG18190	PARG18191	PARG18192	PARG18193	PARG18194	PARG18195
PARG18199	PARG18200	PARG18202	PARG18204	PARG18205	PARG18206	PARG18207
PARG18208	PARG18209	PARG18210	PARG18212	PARG18213	PARG18214	PARG18215
PARG18216	PARG18217	PARG18218	PARG18219	PARG18220	PARG18221	PARG18222
PARG18223	PARG18225	PARG18226	PARG18227	PARG18228	PARG18229	PARG18230
PARG18231	PARG18233	PARG18235	PARG18238	PARG18239	PARG18240	PARG18241
PARG18242	PARG18243	PARG18244	PARG18245	PARG18246	PARG18247	PARG18248
PARG18249	PARG18250	PARG18251	PARG18252	PARG18258	PARG18259	PARG18260
PARG18261	PARG18262	PARG18263	PARG18264	PARG18265	PARG18266	PARG18267
PARG18268	PARG18269	PARG18270	PARG18271	PARG18272	PARG18273	PARG18274
PARG18275	PARG18278	PARG18279	PARG18283	PARG18287	PARG18290	PARG18291
PARG18292	PARG18297	PARG18298	PARG18299	PARG18300	PARG18301	PARG18303
PARG18304	PARG18305	PARG18306	PARG18308	PARG18309	PARG18310	PARG18311
PARG18312	PARG18314	PARG18316	PARG18317	PARG18319	PARG18320	PARG18321
PARG18322	PARG18323	PARG18324	PARG18325	PARG18326	PARG18327	PARG18328
PARG18329	PARG18330	PARG18331	PARG18332	PARG18333	PARG18334	PARG18335
PARG18336	PARG18337	PARG18338	PARG18339	PARG18340	PARG18342	PARG18343
PARG18344	PARG18345	PARG18346	PARG18348	PARG18349	PARG18350	PARG18351
PARG18352	PARG18353	PARG18354	PARG18355	PARG18356	PARG18357	PARG18358
PARG18359	PARG18360	PARG18361	PARG18362	PARG18363	PARG18364	PARG18365
PARG18366	PARG18367	PARG18368	PARG18369	PARG18370	PARG18371	PARG18372
PARG18373	PARG18374	PARG18375	PARG18376	PARG18378	PARG18379	PARG18380
PARG18381	PARG18382	PARG18383	PARG18384	PARG18386	PARG18387	PARG18388
PARG18389	PARG18390	PARG18391	PARG18392	PARG18393	PARG18394	PARG18395
PARG18400	PARG18401	PARG18402	PARG18404	PARG18405	PARG18406	PARG18407
PARG18408	PARG18409	PARG18410	PARG18411	PARG18414	PARG18415	PARG18416
PARG18417	PARG18419	PARG18420	PARG18421	PARG18422	PARG18423	PARG18424
PARG18425	PARG18426	PARG18427	PARG18428	PARG18429	PARG18430	PARG18431
PARG18432	PARG18433	PARG18434	PARG18436	PARG18437	PARG18438	PARG18439
PARG18440	PARG18441	PARG18442	PARG18443	PARG18444	PARG18445	PARG18446
PARG18447	PARG18448	PARG18449	PARG18450	PARG18451	PARG18452	PARG18453
PARG18454	PARG18455	PARG18456	PARG18457	PARG18458	PARG18459	PARG18460
PARG18461	PARG18462	PARG18463	PARG18465	PARG18466	PARG18467	PARG18469
PARG18470	PARG18471	PARG18472	PARG18473	PARG18474	PARG18475	PARG18476

PARG18477	PARG18478	PARG18479	PARG18480	PARG18481	PARG18482	PARG18483
PARG18485	PARG18486	PARG18487	PARG18488	PARG18489	PARG18490	PARG18491
PARG18492	PARG18493	PARG18494	PARG18495	PARG18496	PARG18499	PARG18500
PARG18501	PARG18502	PARG18503	PARG18504	PARG18506	PARG18507	PARG18508
PARG18509	PARG18510	PARG18512	PARG18513	PARG18514	PARG18515	PARG18516
PARG18517	PARG18518	PARG18519	PARG18520	PARG18521	PARG18522	PARG18523
PARG18524	PARG18525	PARG18526	PARG18527	PARG18528	PARG18531	PARG18532
PARG18533	PARG18534	PARG18536	PARG18537	PARG18538	PARG18539	PARG18540
PARG18541	PARG18542	PARG18543	PARG18544	PARG18545	PARG18546	PARG18547
PARG18549	PARG18550	PARG18551	PARG18552	PARG18553	PARG18556	PARG18557
PARG18558	PARG18559	PARG18560	PARG18561	PARG18562	PARG18564	PARG18565
PARG18566	PARG18567	PARG18568	PARG18569	PARG18572	PARG18576	PARG18577
PARG18578	PARG18579	PARG18580	PARG18581	PARG18582	PARG18583	PARG18584
PARG18585	PARG18586	PARG18588	PARG18589	PARG18590	PARG18592	PARG18593
PARG18594	PARG18596	PARG18597	PARG18598	PARG18599	PARG18600	PARG18601
PARG18602	PARG18603	PARG18604	PARG18605	PARG18606	PARG18607	PARG18608
PARG18610	PARG18611	PARG18612	PARG18613	PARG18614	PARG18615	PARG18616
PARG18617	PARG18618	PARG18619	PARG18620	PARG18622	PARG18623	PARG18624
PARG18625	PARG18626	PARG18627	PARG18628	PARG18629	PARG18630	PARG18631
PARG18632	PARG18633	PARG18634	PARG18635	PARG18636	PARG18637	PARG18641
PARG18642	PARG18643	PARG18644	PARG18645	PARG18647	PARG18648	PARG18649
PARG18650	PARG18651	PARG18652	PARG18653	PARG18654	PARG18655	PARG18656
PARG18658	PARG18662	PARG18663	PARG18665	PARG18666	PARG18667	PARG18668
PARG18669	PARG18670	PARG18671	PARG18672	PARG18675	PARG18676	PARG18677
PARG18678	PARG18679	PARG18683	PARG18684	PARG18685	PARG18686	PARG18689
PARG18690	PARG18691	PARG18694	PARG18695	PARG18696	PARG18697	PARG18699
PARG18700	PARG18701	PARG18702	PARG18703	PARG18704	PARG18706	PARG18707
PARG18711	PARG18712	PARG18713	PARG18714	PARG18715	PARG18716	PARG18717
PARG18718	PARG18721	PARG18722	PARG18723	PARG18724	PARG18725	PARG18726
PARG18727	PARG18728	PARG18729	PARG18730	PARG18731	PARG18733	PARG18734
PARG18735	PARG18736	PARG18737	PARG18739	PARG18740	PARG18741	PARG18742
PARG18743	PARG18744	PARG18745	PARG18746	PARG18747	PARG18750	PARG18751
PARG18752	PARG18753	PARG18754	PARG18755	PARG18756	PARG18758	PARG18759
PARG18760	PARG18761	PARG18762	PARG18763	PARG18764	PARG18765	PARG18766
PARG18767	PARG18768	PARG18771	PARG18772	PARG18774	PARG18775	PARG18777
PARG18779	PARG18780	PARG18781	PARG18782	PARG18783	PARG18784	PARG18786
PARG18787	PARG18788	PARG18791	PARG18792	PARG18793	PARG18794	PARG18795
PARG18796	PARG18797	PARG18798	PARG18799	PARG18800	PARG18801	PARG18802
PARG18803	PARG18804	PARG18805	PARG18806	PARG18807	PARG18808	PARG18809
PARG18810	PARG18813	PARG18817	PARG18818	PARG18819	PARG18820	PARG18821
PARG18823	PARG18824	PARG18825	PARG18826	PARG18828	PARG18829	PARG18830
PARG18831	PARG18832	PARG18833	PARG18834	PARG18836	PARG18837	PARG18838
PARG18839	PARG18840	PARG18841	PARG18842	PARG18843	PARG18844	PARG18845
PARG18846	PARG18847	PARG18848	PARG18849	PARG18850	PARG18852	PARG18853
PARG18854	PARG18855	PARG18858	PARG18859	PARG18860	PARG18863	PARG18864
PARG18865	PARG18873	PARG18875	PARG18877	PARG18879	PARG18880	PARG18882

PARG18884	PARG18885	PARG18886	PARG18887	PARG18888	PARG18889	PARG18890
PARG18891	PARG18892	PARG18893	PARG18894	PARG18895	PARG18896	PARG18897
PARG18898	PARG18899	PARG18901	PARG18902	PARG18903	PARG18904	PARG18905
PARG18906	PARG18907	PARG18908	PARG18909	PARG18910	PARG18911	PARG18913
PARG18915	PARG18917	PARG18918	PARG18919	PARG18920	PARG18921	PARG18922
PARG18923	PARG18924	PARG18925	PARG18926	PARG18928	PARG18929	PARG18930
PARG18932	PARG18933	PARG18935	PARG18936	PARG18937	PARG18938	PARG18939
PARG18940	PARG18941	PARG18942	PARG18943	PARG18944	PARG18945	PARG18947
PARG18948	PARG18951	PARG18952	PARG18954	PARG18955	PARG18956	PARG18958
PARG18959	PARG18960	PARG18961	PARG18962	PARG18963	PARG18964	PARG18965
PARG18966	PARG18967	PARG18968	PARG18971	PARG18972	PARG18973	PARG18974
PARG18975	PARG18976	PARG18977	PARG18978	PARG18979	PARG18980	PARG18981
PARG18982	PARG18983	PARG18984	PARG18985	PARG18986	PARG18987	PARG18988
PARG18989	PARG18990	PARG18991	PARG18992	PARG18995	PARG18996	PARG18997
PARG18998	PARG18999	PARG19000	PARG19001	PARG19002	PARG19003	PARG19004
PARG19005	PARG19007	PARG19008	PARG19009	PARG19010	PARG19011	PARG19012
PARG19014	PARG19015	PARG19016	PARG19017	PARG19018	PARG19019	PARG19020
PARG19021	PARG19022	PARG19023	PARG19024	PARG19025	PARG19026	PARG19028
PARG19029	PARG19030	PARG19031	PARG19032	PARG19033	PARG19034	PARG19035
PARG19036	PARG19037	PARG19038	PARG19039	PARG19040	PARG19041	PARG19042
PARG19043	PARG19045	PARG19046	PARG19047	PARG19048	PARG19049	PARG19050
PARG19051	PARG19052	PARG19053	PARG19054	PARG19055	PARG19056	PARG19057
PARG19058	PARG19059	PARG19060	PARG19061	PARG19062	PARG19063	PARG19064
PARG19065	PARG19066	PARG19067	PARG19070	PARG19071	PARG19072	PARG19073
PARG19074	PARG19075	PARG19076	PARG19077	PARG19078	PARG19079	PARG19080
PARG19081	PARG19083	PARG19084	PARG19085	PARG19086	PARG19087	PARG19088
PARG19089	PARG19090	PARG19091	PARG19092	PARG19093	PARG19094	PARG19095
PARG19096	PARG19097	PARG19098	PARG19099	PARG19100	PARG19101	PARG19102
PARG19103	PARG19104	PARG19105	PARG19106	PARG19107	PARG19108	PARG19109
PARG19110	PARG19111	PARG19112	PARG19113	PARG19114	PARG19117	PARG19118
PARG19119	PARG19120	PARG19121	PARG19122	PARG19123	PARG19124	PARG19125
PARG19126	PARG19127	PARG19128	PARG19129	PARG19130	PARG19131	PARG19132
PARG19133	PARG19134	PARG19135	PARG19136	PARG19137	PARG19138	PARG19139
PARG19143	PARG19144	PARG19145	PARG19146	PARG19147	PARG19148	PARG19149
PARG19150	PARG19151	PARG19152	PARG19153	PARG19154	PARG19155	PARG19156
PARG19157	PARG19158	PARG19159	PARG19160	PARG19161	PARG19162	PARG19163
PARG19164	PARG19165	PARG19166	PARG19167	PARG19168	PARG19169	PARG19170
PARG19171	PARG19172	PARG19173	PARG19174	PARG19175	PARG19176	PARG19177
PARG19178	PARG19179	PARG19180	PARG19181	PARG19182	PARG19183	PARG19184
PARG19185	PARG19186	PARG19187	PARG19188	PARG19189	PARG19190	PARG19191
PARG19192	PARG19193	PARG19194	PARG19196	PARG19197	PARG19198	PARG19199
PARG19200	PARG19201	PARG19202	PARG19203	PARG19204	PARG19205	PARG19206
PARG19207	PARG19208	PARG19209	PARG19210	PARG19211	PARG19212	PARG19213
PARG19214	PARG19216	PARG19217	PARG19218	PARG19219	PARG19220	PARG19221
PARG19222	PARG19223	PARG19224	PARG19225	PARG19226	PARG19227	PARG19228
PARG19229	PARG19230	PARG19232	PARG19234	PARG19235	PARG19236	PARG19237

PARG19239	PARG19240	PARG19241	PARG19242	PARG19243	PARG19244	PARG19245
PARG19246	PARG19247	PARG19248	PARG19249	PARG19250	PARG19251	PARG19252
PARG19253	PARG19255	PARG19258	PARG19259	PARG19260	PARG19261	PARG19262
PARG19263	PARG19264	PARG19266	PARG19267	PARG19268	PARG19269	PARG19270
PARG19271	PARG19272	PARG19273	PARG19274	PARG19275	PARG19276	PARG19277
PARG19278	PARG19279	PARG19280	PARG19281	PARG19282	PARG19283	PARG19284
PARG19285	PARG19286	PARG19287	PARG19288	PARG19289	PARG19291	PARG19292
PARG19293	PARG19294	PARG19295	PARG19296	PARG19297	PARG19299	PARG19300
PARG19301	PARG19302	PARG19303	PARG19304	PARG19305	PARG19306	PARG19307
PARG19309	PARG19310	PARG19311	PARG19312	PARG19314	PARG19315	PARG19316
PARG19317	PARG19318	PARG19319	PARG19320	PARG19321	PARG19323	PARG19325
PARG19326	PARG19327	PARG19328	PARG19329	PARG19330	PARG19331	PARG19332
PARG19334	PARG19335	PARG19336	PARG19337	PARG19338	PARG19339	PARG19341
PARG19342	PARG19344	PARG19345	PARG19346	PARG19347	PARG19348	PARG19349
PARG19350	PARG19351	PARG19352	PARG19353	PARG19354	PARG19355	PARG19356
PARG19357	PARG19358	PARG19359	PARG19360	PARG19361	PARG19363	PARG19364
PARG19365	PARG19366	PARG19367	PARG19368	PARG19369	PARG19370	PARG19371
PARG19372	PARG19373	PARG19374	PARG19375	PARG19376	PARG19377	PARG19379
PARG19380	PARG19381	PARG19382	PARG19383	PARG19384	PARG19385	PARG19386
PARG19387	PARG19388	PARG19389	PARG19392	PARG19393	PARG19394	PARG19395
PARG19396	PARG19397	PARG19398	PARG19400	PARG19401	PARG19402	PARG19403
PARG19404	PARG19405	PARG19406	PARG19407	PARG19408	PARG19409	PARG19410
PARG19411	PARG19412	PARG19413	PARG19414	PARG19415	PARG19416	PARG19417
PARG19418	PARG19419	PARG19420	PARG19421	PARG19422	PARG19423	PARG19424
PARG19425	PARG19426	PARG19427	PARG19428	PARG19429	PARG19430	PARG19431
PARG19432	PARG19433	PARG19434	PARG19436	PARG19437	PARG19438	PARG19439
PARG19440	PARG19441	PARG19442	PARG19443	PARG19444	PARG19445	PARG19446
PARG19447	PARG19448	PARG19449	PARG19450	PARG19451	PARG19452	PARG19453
PARG19454	PARG19455	PARG19456	PARG19457	PARG19458	PARG19459	PARG19461
PARG19462	PARG19463	PARG19464	PARG19465	PARG19466	PARG19467	PARG19468
PARG19469	PARG19470	PARG19471	PARG19472	PARG19473	PARG19474	PARG19475
PARG19476	PARG19477	PARG19478	PARG19479	PARG19480	PARG19481	PARG19482
PARG19483	PARG19484	PARG19485	PARG19486	PARG19487	PARG19488	PARG19489
PARG19490	PARG19491	PARG19492	PARG19493	PARG19494	PARG19495	PARG19496
PARG19497	PARG19498	PARG19499	PARG19501	PARG19502	PARG19503	PARG19504
PARG19505	PARG19506	PARG19507	PARG19508	PARG19509	PARG19510	PARG19511
PARG19512	PARG19513	PARG19514	PARG19515	PARG19516	PARG19517	PARG19518
PARG19519	PARG19520	PARG19522	PARG19523	PARG19524	PARG19525	PARG19526
PARG19527	PARG19528	PARG19529	PARG19530	PARG19531	PARG19532	PARG19533
PARG19534	PARG19535	PARG19536	PARG19539	PARG19542	PARG19543	PARG19544
PARG19545	PARG19546	PARG19547	PARG19548	PARG19549	PARG19550	PARG19551
PARG19552	PARG19553	PARG19554	PARG19555	PARG19556	PARG19557	PARG19558
PARG19559	PARG19561	PARG19562	PARG19563	PARG19566	PARG19567	PARG19569
PARG19570	PARG19571	PARG19572	PARG19573	PARG19574	PARG19575	PARG19576
PARG19577	PARG19578	PARG19579	PARG19580	PARG19581	PARG19582	PARG19583
PARG19584	PARG19585	PARG19586	PARG19587	PARG19588	PARG19589	PARG19590

PARG19591	PARG19592	PARG19593	PARG19594	PARG19595	PARG19596	PARG19597
PARG19598	PARG19599	PARG19601	PARG19602	PARG19603	PARG19604	PARG19605
PARG19606	PARG19607	PARG19608	PARG19609	PARG19611	PARG19612	PARG19613
PARG19614	PARG19615	PARG19616	PARG19617	PARG19618	PARG19619	PARG19620
PARG19621	PARG19622	PARG19623	PARG19624	PARG19625	PARG19626	PARG19628
PARG19629	PARG19630	PARG19631	PARG19632	PARG19633	PARG19634	PARG19635
PARG19636	PARG19637	PARG19638	PARG19639	PARG19640	PARG19641	PARG19642
PARG19643	PARG19644	PARG19645	PARG19646	PARG19647	PARG19648	PARG19649
PARG19653	PARG19654	PARG19655	PARG19656	PARG19657	PARG19658	PARG19659
PARG19660	PARG19661	PARG19663	PARG19664	PARG19665	PARG19666	PARG19667
PARG19668	PARG19669	PARG19670	PARG19671	PARG19672	PARG19673	PARG19674
PARG19675	PARG19676	PARG19677	PARG19678	PARG19679	PARG19680	PARG19681
PARG19682	PARG19683	PARG19684	PARG19686	PARG19687	PARG19688	PARG19689
PARG19690	PARG19691	PARG19692	PARG19693	PARG19694	PARG19695	PARG19696
PARG19697	PARG19698	PARG19699	PARG19700	PARG19701	PARG19702	PARG19703
PARG19704	PARG19705	PARG19706	PARG19707	PARG19708	PARG19709	PARG19711
PARG19712	PARG19713	PARG19714	PARG19715	PARG19716	PARG19717	PARG19718
PARG19719	PARG19720	PARG19721	PARG19722	PARG19723	PARG19724	PARG19725
PARG19726	PARG19727	PARG19728	PARG19729	PARG19730	PARG19731	PARG19732
PARG19733	PARG19734	PARG19735	PARG19736	PARG19737	PARG19738	PARG19739
PARG19741	PARG19742	PARG19743	PARG19744	PARG19745	PARG19748	PARG19749
PARG19750	PARG19751	PARG19752	PARG19753	PARG19754	PARG19755	PARG19756
PARG19757	PARG19758	PARG19759	PARG19760	PARG19761	PARG19762	PARG19763
PARG19764	PARG19765	PARG19766	PARG19767	PARG19768	PARG19770	PARG19771
PARG19773	PARG19774	PARG19775	PARG19776	PARG19777	PARG19778	PARG19779
PARG19780	PARG19781	PARG19782	PARG19783	PARG19784	PARG19785	PARG19786
PARG19787	PARG19788	PARG19789	PARG19790	PARG19791	PARG19792	PARG19793
PARG19794	PARG19795	PARG19796	PARG19797	PARG19798	PARG19801	PARG19802
PARG19803	PARG19804	PARG19805	PARG19807	PARG19808	PARG19809	PARG19810
PARG19811	PARG19812	PARG19813	PARG19815	PARG19816	PARG19817	PARG19818
PARG19819	PARG19820	PARG19821	PARG19822	PARG19823	PARG19824	PARG19825
PARG19826	PARG19827	PARG19829	PARG19830	PARG19831	PARG19832	PARG19833
PARG19834	PARG19835	PARG19836	PARG19837	PARG19838	PARG19839	PARG19840
PARG19841	PARG19842	PARG19843	PARG19844	PARG19845	PARG19847	PARG19851
PARG19852	PARG19853	PARG19854	PARG19855	PARG19856	PARG19857	PARG19858
PARG19859	PARG19860	PARG19861	PARG19862	PARG19863	PARG19864	PARG19865
PARG19866	PARG19867	PARG19868	PARG19869	PARG19870	PARG19871	PARG19872
PARG19873	PARG19874	PARG19875	PARG19876	PARG19877	PARG19879	PARG19880
PARG19881	PARG19882	PARG19883	PARG19884	PARG19885	PARG19886	PARG19887
PARG19888	PARG19889	PARG19890	PARG19891	PARG19892	PARG19893	PARG19894
PARG19895	PARG19896	PARG19897	PARG19898	PARG19899	PARG19900	PARG19901
PARG19902	PARG19903	PARG19904	PARG19905	PARG19906	PARG19907	PARG19908
PARG19909	PARG19910	PARG19911	PARG19913	PARG19914	PARG19915	PARG19916
PARG19917	PARG19920	PARG19921	PARG19923	PARG19924	PARG19925	PARG19926
PARG19927	PARG19928	PARG19929	PARG19930	PARG19931	PARG19932	PARG19933
PARG19934	PARG19935	PARG19936	PARG19937	PARG19938	PARG19939	PARG19940

PARG19942	PARG19943	PARG19944	PARG19945	PARG19946	PARG19947	PARG19948
PARG19949	PARG19950	PARG19951	PARG19952	PARG19953	PARG19954	PARG19955
PARG19957	PARG19958	PARG19959	PARG19960	PARG19961	PARG19962	PARG19963
PARG19964	PARG19965	PARG19966	PARG19967	PARG19968	PARG19969	PARG19970
PARG19971	PARG19972	PARG19973	PARG19974	PARG19975	PARG19979	PARG19980
PARG19981	PARG19982	PARG19983	PARG19984	PARG19985	PARG19986	PARG19987
PARG19988	PARG19989	PARG19990	PARG19991	PARG19992	PARG19993	PARG19994
PARG19995	PARG19996	PARG19997	PARG19998	PARG19999	PARG20000	PARG20001
PARG20002	PARG20003	PARG20004	PARG20005	PARG20006	PARG20007	PARG20008
PARG20009	PARG20010	PARG20011	PARG20013	PARG20014	PARG20015	PARG20016
PARG20017	PARG20018	PARG20019	PARG20021	PARG20022	PARG20023	PARG20024
PARG20025	PARG20026	PARG20027	PARG20028	PARG20029	PARG20030	PARG20031
PARG20032	PARG20033	PARG20034	PARG20035	PARG20036	PARG20037	PARG20038
PARG20039	PARG20040	PARG20041	PARG20042	PARG20043	PARG20044	PARG20045
PARG20047	PARG20048	PARG20049	PARG20050	PARG20051	PARG20052	PARG20053
PARG20054	PARG20055	PARG20056	PARG20058	PARG20059	PARG20060	PARG20061
PARG20062	PARG20063	PARG20065	PARG20066	PARG20067	PARG20069	PARG20070
PARG20071	PARG20072	PARG20073	PARG20076	PARG20077	PARG20078	PARG20079
PARG20080	PARG20081	PARG20082	PARG20084	PARG20085	PARG20086	PARG20087
PARG20088	PARG20089	PARG20090	PARG20091	PARG20092	PARG20093	PARG20094
PARG20095	PARG20096	PARG20097	PARG20099	PARG20100	PARG20101	PARG20102
PARG20103	PARG20104	PARG20105	PARG20106	PARG20107	PARG20108	PARG20109
PARG20110	PARG20111	PARG20112	PARG20113	PARG20114	PARG20115	PARG20117
PARG20118	PARG20119	PARG20120	PARG20121	PARG20122	PARG20124	PARG20125
PARG20126	PARG20127	PARG20128	PARG20129	PARG20130	PARG20131	PARG20132
PARG20133	PARG20134	PARG20135	PARG20136	PARG20137	PARG20138	PARG20139
PARG20140	PARG20141	PARG20142	PARG20143	PARG20144	PARG20145	PARG20146
PARG20147	PARG20148	PARG20150	PARG20151	PARG20152	PARG20153	PARG20154
PARG20155	PARG20156	PARG20157	PARG20158	PARG20159	PARG20160	PARG20161
PARG20162	PARG20163	PARG20164	PARG20165	PARG20166	PARG20167	PARG20168
PARG20169	PARG20171	PARG20172	PARG20173	PARG20174	PARG20175	PARG20176
PARG20177	PARG20178	PARG20179	PARG20180	PARG20181	PARG20182	PARG20183
PARG20184	PARG20185	PARG20186	PARG20187	PARG20188	PARG20192	PARG20193
PARG20194	PARG20195	PARG20196	PARG20197	PARG20198	PARG20199	PARG20200
PARG20201	PARG20202	PARG20203	PARG20204	PARG20206	PARG20208	PARG20209
PARG20210	PARG20212	PARG20214	PARG20215	PARG20216	PARG20217	PARG20218
PARG20219	PARG20220	PARG20221	PARG20222	PARG20223	PARG20224	PARG20225
PARG20226	PARG20228	PARG20229	PARG20230	PARG20231	PARG20232	PARG20233
PARG20234	PARG20235	PARG20237	PARG20238	PARG20239	PARG20240	PARG20242
PARG20243	PARG20244	PARG20245	PARG20246	PARG20247	PARG20248	PARG20249
PARG20250	PARG20251	PARG20252	PARG20253	PARG20254	PARG20255	PARG20256
PARG20257	PARG20258	PARG20259	PARG20260	PARG20261	PARG20262	PARG20263
PARG20264	PARG20265	PARG20266	PARG20267	PARG20268	PARG20269	PARG20270
PARG20271	PARG20273	PARG20274	PARG20275	PARG20276	PARG20277	PARG20278
PARG20279	PARG20280	PARG20281	PARG20282	PARG20283	PARG20284	PARG20285
PARG20286	PARG20287	PARG20288	PARG20289	PARG20290	PARG20291	PARG20292

PARG20293	PARG20294	PARG20296	PARG20299	PARG20300	PARG20303	PARG20304
PARG20305	PARG20307	PARG20308	PARG20309	PARG20311	PARG20312	PARG20313
PARG20314	PARG20315	PARG20316	PARG20317	PARG20318	PARG20320	PARG20321
PARG20322	PARG20323	PARG20325	PARG20326	PARG20329	PARG20330	PARG20331
PARG20332	PARG20333	PARG20334	PARG20335	PARG20336	PARG20337	PARG20338
PARG20339	PARG20340	PARG20341	PARG20342	PARG20343	PARG20345	PARG20346
PARG20347	PARG20348	PARG20349	PARG20350	PARG20351	PARG20352	PARG20353
PARG20354	PARG20355	PARG20357	PARG20358	PARG20359	PARG20362	PARG20363
PARG20364	PARG20365	PARG20366	PARG20367	PARG20368	PARG20372	PARG20376
PARG20377	PARG20378	PARG20379	PARG20380	PARG20381	PARG20382	PARG20384
PARG20385	PARG20386	PARG20387	PARG20388	PARG20391	PARG20392	PARG20393
PARG20394	PARG20395	PARG20396	PARG20397	PARG20398	PARG20399	PARG20400
PARG20401	PARG20402	PARG20403	PARG20404	PARG20405	PARG20406	PARG20407
PARG20408	PARG20409	PARG20410	PARG20411	PARG20422	PARG20423	PARG20424
PARG20425	PARG20426	PARG20427	PARG20428	PARG20429	PARG20430	PARG20431
PARG20432	PARG20433	PARG20434	PARG20435	PARG20439	PARG20441	PARG20442
PARG20443	PARG20444	PARG20450	PARG20451	PARG20452	PARG20453	PARG20454
PARG20455	PARG20456	PARG20457	PARG20458	PARG20459	PARG20460	PARG20461
PARG20463	PARG20464	PARG20465	PARG20466	PARG20467	PARG20468	PARG20470
PARG20471	PARG20472	PARG20473	PARG20474	PARG20475	PARG20476	PARG20477
PARG20478	PARG20479	PARG20480	PARG20481	PARG20482	PARG20484	PARG20485
PARG20486	PARG20487	PARG20489	PARG20490	PARG20491	PARG20492	PARG20493
PARG20494	PARG20496	PARG20497	PARG20498	PARG20499	PARG20500	PARG20501
PARG20502	PARG20503	PARG20504	PARG20505	PARG20506	PARG20507	PARG20508
PARG20509	PARG20510	PARG20511	PARG20513	PARG20514	PARG20515	PARG20516
PARG20517	PARG20518	PARG20519	PARG20520	PARG20521	PARG20522	PARG20524
PARG20527	PARG20528	PARG20529	PARG20530	PARG20531	PARG20532	PARG20533
PARG20534	PARG20535	PARG20536	PARG20537	PARG20538	PARG20539	PARG20540
PARG20543	PARG20544	PARG20545	PARG20546	PARG20547	PARG20548	PARG20549
PARG20550	PARG20551	PARG20552	PARG20553	PARG20554	PARG20555	PARG20556
PARG20557	PARG20559	PARG20560	PARG20561	PARG20562	PARG20563	PARG20564
PARG20565	PARG20566	PARG20567	PARG20568	PARG20569	PARG20570	PARG20573
PARG20574	PARG20575	PARG20576	PARG20577	PARG20580	PARG20581	PARG20582
PARG20583	PARG20584	PARG20585	PARG20586	PARG20587	PARG20588	PARG20589
PARG20590	PARG20591	PARG20592	PARG20593	PARG20594	PARG20595	PARG20596
PARG20597	PARG20598	PARG20599	PARG20600	PARG20601	PARG20602	PARG20603
PARG20604	PARG20605	PARG20606	PARG20607	PARG20608	PARG20609	PARG20610
PARG20611	PARG20612	PARG20613	PARG20614	PARG20615	PARG20616	PARG20618
PARG20619	PARG20620	PARG20621	PARG20625	PARG20626	PARG20629	PARG20630
PARG20631	PARG20633	PARG20634	PARG20635	PARG20636	PARG20637	PARG20638
PARG20639	PARG20640	PARG20641	PARG20642	PARG20643	PARG20644	PARG20645
PARG20646	PARG20647	PARG20648	PARG20649	PARG20650	PARG20651	PARG20652
PARG20653	PARG20654	PARG20655	PARG20656	PARG20657	PARG20658	PARG20659
PARG20660	PARG20661	PARG20662	PARG20663	PARG20665	PARG20666	PARG20667
PARG20668	PARG20669	PARG20670	PARG20671	PARG20672	PARG20673	PARG20674
PARG20675	PARG20676	PARG20677	PARG20681	PARG20682	PARG20683	PARG20684

PARG20685	PARG20686	PARG20687	PARG20688	PARG20691	PARG20692	PARG20693
PARG20694	PARG20695	PARG20696	PARG20697	PARG20698	PARG20699	PARG20700
PARG20701	PARG20702	PARG20703	PARG20704	PARG20705	PARG20706	PARG20707
PARG20708	PARG20709	PARG20710	PARG20711	PARG20713	PARG20714	PARG20715
PARG20716	PARG20717	PARG20718	PARG20719	PARG20720	PARG20721	PARG20722
PARG20726	PARG20731	PARG20732	PARG20733	PARG20734	PARG20735	PARG20736
PARG20737	PARG20738	PARG20739	PARG20740	PARG20742	PARG20743	PARG20744
PARG20745	PARG20746	PARG20747	PARG20748	PARG20749	PARG20751	PARG20753
PARG20757	PARG20758	PARG20764	PARG20768	PARG20772	PARG20779	PARG20780
PARG20781	PARG20782	PARG20783	PARG20784	PARG20785	PARG20786	PARG20787
PARG20788	PARG20789	PARG20790	PARG20791	PARG20792	PARG20793	PARG20794
PARG20795	PARG20796	PARG20797	PARG20798	PARG20799	PARG20800	PARG20801
PARG20802	PARG20804	PARG20805	PARG20806	PARG20807	PARG20808	PARG20809
PARG20810	PARG20811	PARG20812	PARG20813	PARG20814	PARG20815	PARG20816
PARG20817	PARG20818	PARG20819	PARG20820	PARG20821	PARG20822	PARG20823
PARG20824	PARG20825	PARG20826	PARG20828	PARG20829	PARG20830	PARG20831
PARG20832	PARG20833	PARG20834	PARG20835	PARG20836	PARG20837	PARG20838
PARG20839	PARG20840	PARG20841	PARG20843	PARG20846	PARG20847	PARG20848
PARG20849	PARG20850	PARG20851	PARG20852	PARG20853	PARG20854	PARG20855
PARG20856	PARG20857	PARG20858	PARG20859	PARG20862	PARG20863	PARG20864
PARG20865	PARG20866	PARG20867	PARG20868	PARG20870	PARG20871	PARG20872
PARG20873	PARG20874	PARG20875	PARG20876	PARG20877	PARG20878	PARG20879
PARG20880	PARG20881	PARG20882	PARG20883	PARG20884	PARG20885	PARG20886
PARG20887	PARG20888	PARG20889	PARG20890	PARG20891	PARG20892	PARG20893
PARG20894	PARG20895	PARG20896	PARG20898	PARG20900	PARG20901	PARG20902
PARG20903	PARG20904	PARG20905	PARG20906	PARG20907	PARG20908	PARG20909
PARG20910	PARG20911	PARG20912	PARG20913	PARG20914	PARG20915	PARG20916
PARG20917	PARG20918	PARG20919	PARG20920	PARG20921	PARG20922	PARG20923
PARG20924	PARG20925	PARG20926	PARG20927	PARG20928	PARG20930	PARG20931
PARG20932	PARG20933	PARG20934	PARG20935	PARG20936	PARG20937	PARG20938
PARG20939	PARG20940	PARG20941	PARG20942	PARG20943	PARG20944	PARG20945
PARG20947	PARG20948	PARG20949	PARG20950	PARG20951	PARG20952	PARG20953
PARG20954	PARG20955	PARG20959	PARG20961	PARG20962	PARG20967	PARG20970
PARG20971	PARG20972	PARG20973	PARG20974	PARG20975	PARG20976	PARG20977
PARG20978	PARG20979	PARG20980	PARG20981	PARG20982	PARG20983	PARG20984
PARG20992	PARG20993	PARG20994	PARG20995	PARG20996	PARG20997	PARG20999
PARG21000	PARG21002	PARG21003	PARG21004	PARG21005	PARG21007	PARG21008
PARG21009	PARG21011	PARG21013	PARG21014	PARG21015	PARG21017	PARG21019
PARG21020	PARG21022	PARG21023	PARG21024	PARG21025	PARG21026	PARG21027
PARG21028	PARG21029	PARG21030	PARG21031	PARG21032	PARG21033	PARG21037
PARG21038	PARG21039	PARG21040	PARG21041	PARG21042	PARG21048	PARG21049
PARG21050	PARG21051	PARG21052	PARG21053	PARG21054	PARG21055	PARG21056
PARG21057	PARG21059	PARG21060	PARG21062	PARG21063	PARG21064	PARG21065
PARG21066	PARG21067	PARG21068	PARG21069	PARG21070	PARG21071	PARG21072
PARG21073	PARG21074	PARG21075	PARG21076	PARG21077	PARG21078	PARG21079
PARG21080	PARG21081	PARG21082	PARG21083	PARG21084	PARG21085	PARG21086

PARG21087	PARG21088	PARG21089	PARG21090	PARG21091	PARG21092	PARG21093
PARG21094	PARG21099	PARG21100	PARG21101	PARG21102	PARG21103	PARG21104
PARG21105	PARG21106	PARG21107	PARG21108	PARG21109	PARG21110	PARG21111
PARG21113	PARG21114	PARG21115	PARG21116	PARG21117	PARG21118	PARG21119
PARG21120	PARG21121	PARG21122	PARG21123	PARG21124	PARG21125	PARG21126
PARG21128	PARG21129	PARG21130	PARG21131	PARG21132	PARG21133	PARG21135
PARG21136	PARG21137	PARG21138	PARG21139	PARG21140	PARG21141	PARG21142
PARG21143	PARG21144	PARG21145	PARG21150	PARG21151	PARG21153	PARG21154
PARG21155	PARG21156	PARG21159	PARG21161	PARG21162	PARG21164	PARG21165
PARG21166	PARG21167	PARG21168	PARG21169	PARG21170	PARG21172	PARG21173
PARG21174	PARG21175	PARG21176	PARG21177	PARG21178	PARG21179	PARG21180
PARG21181	PARG21182	PARG21183	PARG21184	PARG21185	PARG21186	PARG21188
PARG21192	PARG21193	PARG21194	PARG21200	PARG21201	PARG21202	PARG21203
PARG21204	PARG21205	PARG21206	PARG21210	PARG21211	PARG21212	PARG21213
PARG21214	PARG21215	PARG21216	PARG21217	PARG21218	PARG21220	PARG21221
PARG21222	PARG21223	PARG21224	PARG21225	PARG21227	PARG21228	PARG21229
PARG21230	PARG21231	PARG21232	PARG21233	PARG21234	PARG21235	PARG21236
PARG21240	PARG21241	PARG21242	PARG21243	PARG21245	PARG21246	PARG21247
PARG21248	PARG21249	PARG21250	PARG21251	PARG21252	PARG21253	PARG21254
PARG21256	PARG21257	PARG21259	PARG21260	PARG21261	PARG21262	PARG21263
PARG21264	PARG21265	PARG21266	PARG21267	PARG21269	PARG21270	PARG21271
PARG21272	PARG21273	PARG21274	PARG21275	PARG21276	PARG21277	PARG21280
PARG21281	PARG21282	PARG21283	PARG21285	PARG21286	PARG21287	PARG21288
PARG21289	PARG21290	PARG21296	PARG21300	PARG21305	PARG21306	PARG21307
PARG21310	PARG21312	PARG21313	PARG21314	PARG21315	PARG21316	PARG21317
PARG21318	PARG21319	PARG21320	PARG21321	PARG21322	PARG21323	PARG21324
PARG21326	PARG21327	PARG21328	PARG21329	PARG21332	PARG21333	PARG21334
PARG21335	PARG21336	PARG21337	PARG21338	PARG21339	PARG21340	PARG21341
PARG21342	PARG21344	PARG21345	PARG21346	PARG21347	PARG21348	PARG21349
PARG21350	PARG21351	PARG21352	PARG21355	PARG21366	PARG21368	PARG21371
PARG21372	PARG21374	PARG21379	PARG21380	PARG21382	PARG21383	PARG21387
PARG21388	PARG21389	PARG21390	PARG21391	PARG21392	PARG21395	PARG21396
PARG21397	PARG21401	PARG21402	PARG21403	PARG21404	PARG21405	PARG21406
PARG21407	PARG21408	PARG21409	PARG21411	PARG21412	PARG21413	PARG21414
PARG21415	PARG21416	PARG21417	PARG21418	PARG21419	PARG21420	PARG21421
PARG21423	PARG21424	PARG21425	PARG21426	PARG21427	PARG21428	PARG21429
PARG21430	PARG21432	PARG21433	PARG21434	PARG21435	PARG21436	PARG21437
PARG21438	PARG21441	PARG21442	PARG21444	PARG21445	PARG21448	PARG21449
PARG21450	PARG21453	PARG21454	PARG21455	PARG21456	PARG21457	PARG21458
PARG21459	PARG21460	PARG21461	PARG21462	PARG21463	PARG21465	PARG21466
PARG21468	PARG21469	PARG21470	PARG21472	PARG21473	PARG21474	PARG21476
PARG21480	PARG21481	PARG21482	PARG21483	PARG21484	PARG21485	PARG21488
PARG21489	PARG21491	PARG21495	PARG21496	PARG21497	PARG21498	PARG21499
PARG21500	PARG21501	PARG21503	PARG21504	PARG21507	PARG21508	PARG21509
PARG21510	PARG21511	PARG21512	PARG21513	PARG21514	PARG21515	PARG21516
PARG21518	PARG21519	PARG21520	PARG21521	PARG21523	PARG21524	PARG21527

PARG21528	PARG21529	PARG21530	PARG21531	PARG21532	PARG21533	PARG21534
PARG21535	PARG21536	PARG21537	PARG21538	PARG21539	PARG21540	PARG21541
PARG21542	PARG21543	PARG21544	PARG21545	PARG21551	PARG21552	PARG21553
PARG21554	PARG21555	PARG21556	PARG21557	PARG21558	PARG21559	PARG21560
PARG21562	PARG21563	PARG21564	PARG21565	PARG21566	PARG21567	PARG21568
PARG21571	PARG21572	PARG21573	PARG21574	PARG21575	PARG21576	PARG21577
PARG21578	PARG21579	PARG21580	PARG21581	PARG21582	PARG21584	PARG21585
PARG21586	PARG21587	PARG21588	PARG21589	PARG21590	PARG21591	PARG21592
PARG21593	PARG21594	PARG21596	PARG21597	PARG21598	PARG21599	PARG21600
PARG21601	PARG21602	PARG21603	PARG21604	PARG21605	PARG21606	PARG21607
PARG21609	PARG21611	PARG21612	PARG21619	PARG21620	PARG21622	PARG21623
PARG21624	PARG21625	PARG21626	PARG21627	PARG21628	PARG21630	PARG21631
PARG21632	PARG21633	PARG21634	PARG21635	PARG21639	PARG21642	PARG21644
PARG21647	PARG21648	PARG21649	PARG21650	PARG21651	PARG21652	PARG21653
PARG21654	PARG21655	PARG21658	PARG21659	PARG21660	PARG21661	PARG21662
PARG21663	PARG21664	PARG21665	PARG21666	PARG21668	PARG21669	PARG21670
PARG21671	PARG21672	PARG21673	PARG21674	PARG21675	PARG21676	PARG21677
PARG21681	PARG21682	PARG21683	PARG21687	PARG21688	PARG21689	PARG21690
PARG21693	PARG21695	PARG21698	PARG21699	PARG21700	PARG21701	PARG21702
PARG21703	PARG21705	PARG21709	PARG21710	PARG21711	PARG21712	PARG21713
PARG21714	PARG21717	PARG21718	PARG21719	PARG21720	PARG21721	PARG21722
PARG21723	PARG21724	PARG21726	PARG21728	PARG21729	PARG21730	PARG21731
PARG21732	PARG21733	PARG21734	PARG21735	PARG21736	PARG21737	PARG21738
PARG21740	PARG21741	PARG21747	PARG21748	PARG21749	PARG21750	PARG21752
PARG21753	PARG21755	PARG21758	PARG21772	PARG21773	PARG21774	PARG21776
PARG21779	PARG21785	PARG21790	PARG21791	PARG21793	PARG21794	PARG21795
PARG21796	PARG21800	PARG21801	PARG21802	PARG21803	PARG21804	PARG21805
PARG21807	PARG21808	PARG21809	PARG21810	PARG21811	PARG21812	PARG21814
PARG21816	PARG21817	PARG21818	PARG21822	PARG21823	PARG21824	PARG21831
PARG21832	PARG21833	PARG21834	PARG21837	PARG21838	PARG21839	PARG21840
PARG21841	PARG21842	PARG21846	PARG21847	PARG21848	PARG21853	PARG21854
PARG21857	PARG21862	PARG21863	PARG21864	PARG21865	PARG21866	PARG21867
PARG21868	PARG21870	PARG21871	PARG21872	PARG21873	PARG21874	PARG21876
PARG21877	PARG21878	PARG21881	PARG21889	PARG21895	PARG21905	PARG21907
PARG21914	PARG21915	PARG21916	PARG21928	PARG21930	PARG21932	PARG21934
PARG21935	PARG21936	PARG21938	PARG21943	PARG21945	PARG21946	PARG21947
PARG21950	PARG21954	PARG21957	PARG21960	PARG21966	PARG21968	PARG21969
PARG21970	PARG21974	PARG21978	PARG21981	PARG21984	PARG21990	PARG21993
PARG21994	PARG21997	PARG21998	PARG22002	PARG22004	PARG22005	PARG22006
PARG22007	PARG22009	PARG22011	PARG22012	PARG22013	PARG22014	PARG22015
PARG22018	PARG22019	PARG22020	PARG22021	PARG22022	PARG22028	PARG22029
PARG22030	PARG22031	PARG22033	PARG22034	PARG22035	PARG22036	PARG22041
PARG22048	PARG22053	PARG22055	PARG22058	PARG22059	PARG22062	PARG22064
PARG22066	PARG22070	PARG22074	PARG22076	PARG22077	PARG22081	PARG22083
PARG22086	PARG22087	PARG22088	PARG22091	PARG22095	PARG22096	PARG22097
PARG22098	PARG22099	PARG22100	PARG22103	PARG22104	PARG22105	PARG22106

PARG22107	PARG22108	PARG22110	PARG22115	PARG22116	PARG22117	PARG22118
PARG22119	PARG22123	PARG22127	PARG22129	PARG22130	PARG22133	PARG22134
PARG22136	PARG22138	PARG22141	PARG22142	PARG22150	PARG22151	PARG22152
PARG22160	PARG22161	PARG22162	PARG22163	PARG22164	PARG22165	PARG22166
PARG22167	PARG22168	PARG22170	PARG22171	PARG22172	PARG22174	PARG22175
PARG22177	PARG22178	PARG22181	PARG22183	PARG22184	PARG22185	PARG22186
PARG22187	PARG22189	PARG22193	PARG22194	PARG22196	PARG22197	PARG22198
PARG22199	PARG22200	PARG22201	PARG22202	PARG22203	PARG22204	PARG22205
PARG22206	PARG22207	PARG22208	PARG22209	PARG22210	PARG22211	PARG22212
PARG22213	PARG22216	PARG22217	PARG22218	PARG22220	PARG22222	PARG22223
PARG22224	PARG22226	PARG22227	PARG22231	PARG22233	PARG22234	PARG22239
PARG22240	PARG22244	PARG22245	PARG22246	PARG22247	PARG22248	PARG22249
PARG22250	PARG22251	PARG22252	PARG22253	PARG22254	PARG22255	PARG22256
PARG22257	PARG22258	PARG22259	PARG22260	PARG22263	PARG22264	PARG22265
PARG22266	PARG22267	PARG22268	PARG22269	PARG22270	PARG22271	PARG22272
PARG22274	PARG22275	PARG22276	PARG22277	PARG22280	PARG22282	PARG22285
PARG22286	PARG22287	PARG22288	PARG22293	PARG22294	PARG22296	PARG22297
PARG22298	PARG22299	PARG22300	PARG22301	PARG22306	PARG22307	PARG22308
PARG22309	PARG22310	PARG22311	PARG22313	PARG22314	PARG22315	PARG22317
PARG22318	PARG22319	PARG22322	PARG22323	PARG22324	PARG22325	PARG22326
PARG22327	PARG22331	PARG22332	PARG22333	PARG22334	PARG22335	PARG22336
PARG22338	PARG22343	PARG22345	PARG22346	PARG22347	PARG22348	PARG22351
PARG22353	PARG22355	PARG22364	PARG22365	PARG22366	PARG22367	PARG22368
PARG22369	PARG22372	PARG22374	PARG22375	PARG22378	PARG22379	PARG22380
PARG22381	PARG22382	PARG22383	PARG22384	PARG22385	PARG22386	PARG22387
PARG22388	PARG22392	PARG22393	PARG22394	PARG22395	PARG22396	PARG22397
PARG22398	PARG22399	PARG22400	PARG22403	PARG22405	PARG22406	PARG22408
PARG22410	PARG22413	PARG22414	PARG22415	PARG22416	PARG22417	PARG22418
PARG22419	PARG22421	PARG22423	PARG22424	PARG22425	PARG22428	PARG22429
PARG22432	PARG22433	PARG22435	PARG22437	PARG22443	PARG22444	PARG22445
PARG22446	PARG22449	PARG22450	PARG22452	PARG22455	PARG22460	PARG22462
PARG22464	PARG22465	PARG22466	PARG22468	PARG22469	PARG22470	PARG22471
PARG22472	PARG22473	PARG22476	PARG22478	PARG22480	PARG22481	PARG22482
PARG22483	PARG22488	PARG22489	PARG22490	PARG22491	PARG22492	PARG22494
PARG22495	PARG22499	PARG22500	PARG22501	PARG22502	PARG22503	PARG22507
PARG22508	PARG22509	PARG22510	PARG22511	PARG22512	PARG22513	PARG22514
PARG22515	PARG22519	PARG22520	PARG22521	PARG22522	PARG22524	PARG22525
PARG22526	PARG22529	PARG22531	PARG22532	PARG22534	PARG22535	PARG22536
PARG22537	PARG22538	PARG22539	PARG22540	PARG22541	PARG22542	PARG22543
PARG22544	PARG22545	PARG22546	PARG22547	PARG22548	PARG22549	PARG22550
PARG22551	PARG22552	PARG22553	PARG22555	PARG22556	PARG22557	PARG22558
PARG22559	PARG22560	PARG22562	PARG22563	PARG22564	PARG22566	PARG22569
PARG22570	PARG22572	PARG22573	PARG22574	PARG22575	PARG22579	PARG22580
PARG22582	PARG22585	PARG22587	PARG22588	PARG22596	PARG22597	PARG22599
PARG22600	PARG22601	PARG22602	PARG22603	PARG22604	PARG22609	PARG22615
PARG22619	PARG22620	PARG22621	PARG22622	PARG22623	PARG22627	PARG22628

PARG22629	PARG22630	PARG22631	PARG22632	PARG22634	PARG22636	PARG22637
PARG22638	PARG22639	PARG22641	PARG22644	PARG22646	PARG22649	PARG22652
PARG22653	PARG22655	PARG22656	PARG22657	PARG22658	PARG22659	PARG22660
PARG22661	PARG22662	PARG22663	PARG22664	PARG22665	PARG22666	PARG22667
PARG22668	PARG22669	PARG22670	PARG22671	PARG22672	PARG22673	PARG22674
PARG22675	PARG22676	PARG22677	PARG22678	PARG22679	PARG22681	PARG22682
PARG22684	PARG22685	PARG22686	PARG22688	PARG22689	PARG22692	PARG22693
PARG22697	PARG22702	PARG22703	PARG22704	PARG22705	PARG22706	PARG22707
PARG22712	PARG22713	PARG22715	PARG22716	PARG22717	PARG22718	PARG22719
PARG22720	PARG22721	PARG22722	PARG22723	PARG22724	PARG22725	PARG22726
PARG22728	PARG22729	PARG22730	PARG22731	PARG22732	PARG22733	PARG22734
PARG22735	PARG22737	PARG22741	PARG22743	PARG22745	PARG22746	PARG22747
PARG22748	PARG22749	PARG22750	PARG22751	PARG22752	PARG22753	PARG22754
PARG22755	PARG22756	PARG22757	PARG22759	PARG22760	PARG22761	PARG22766
PARG22767	PARG22768	PARG22769	PARG22770	PARG22771	PARG22772	PARG22773
PARG22774	PARG22775	PARG22776	PARG22777	PARG22778	PARG22779	PARG22780
PARG22781	PARG22782	PARG22787	PARG22788	PARG22789	PARG22790	PARG22791
PARG22792	PARG22793	PARG22794	PARG22795	PARG22796	PARG22797	PARG22798
PARG22799	PARG22800	PARG22801	PARG22802	PARG22803	PARG22804	PARG22805
PARG22806	PARG22807	PARG22808	PARG22810	PARG22812	PARG22813	PARG22814
PARG22815	PARG22816	PARG22817	PARG22818	PARG22819	PARG22820	PARG22821
PARG22822	PARG22823	PARG22824	PARG22825	PARG22826	PARG22827	PARG22830
PARG22831	PARG22832	PARG22833	PARG22834	PARG22835	PARG22836	PARG22838
PARG22839	PARG22840	PARG22841	PARG22842	PARG22843	PARG22844	PARG22847
PARG22850	PARG22852	PARG22854	PARG22856	PARG22859	PARG22860	PARG22862
PARG22863	PARG22864	PARG22865	PARG22867	PARG22868	PARG22869	PARG22870
PARG22872	PARG22873	PARG22874	PARG22875	PARG22876	PARG22878	PARG22879
PARG22880	PARG22881	PARG22882	PARG22883	PARG22884	PARG22886	PARG22888
PARG22889	PARG22892	PARG22893	PARG22894	PARG22895	PARG22896	PARG22897
PARG22899	PARG22900	PARG22901	PARG22902	PARG22903	PARG22907	PARG22908
PARG22909	PARG22910	PARG22911	PARG22912	PARG22913	PARG22914	PARG22915
PARG22918	PARG22921	PARG22922	PARG22923	PARG22924	PARG22925	PARG22926
PARG22927	PARG22929	PARG22931	PARG22932	PARG22933	PARG22934	PARG22935
PARG22936	PARG22937	PARG22938	PARG22939	PARG22940	PARG22941	PARG22942
PARG22943	PARG22944	PARG22945	PARG22946	PARG22948	PARG22949	PARG22950
PARG22951	PARG22952	PARG22953	PARG22955	PARG22956	PARG22957	PARG22961
PARG22962	PARG22963	PARG22964	PARG22967	PARG22968	PARG22969	PARG22970
PARG22971	PARG22972	PARG22974	PARG22975	PARG22976	PARG22977	PARG22978
PARG22979	PARG22982	PARG22986	PARG22987	PARG22988	PARG22989	PARG22990
PARG22991	PARG22993	PARG22994	PARG22997	PARG22998	PARG22999	PARG23001
PARG23002	PARG23004	PARG23005	PARG23006	PARG23007	PARG23012	PARG23015
PARG23017	PARG23019	PARG23020	PARG23022	PARG23025	PARG23026	PARG23027
PARG23029	PARG23030	PARG23031	PARG23034	PARG23035	PARG23036	PARG23037
PARG23038	PARG23039	PARG23041	PARG23043	PARG23045	PARG23047	PARG23050
PARG23051	PARG23052	PARG23053	PARG23054	PARG23055	PARG23059	PARG23060
PARG23062	PARG23063	PARG23064	PARG23065	PARG23066	PARG23067	PARG23068

PARG23069	PARG23070	PARG23071	PARG23072	PARG23073	PARG23074	PARG23075
PARG23076	PARG23078	PARG23079	PARG23080	PARG23082	PARG23083	PARG23085
PARG23088	PARG23093	PARG23094	PARG23095	PARG23096	PARG23097	PARG23099
PARG23100	PARG23101	PARG23103	PARG23104	PARG23105	PARG23106	PARG23107
PARG23108	PARG23109	PARG23110	PARG23111	PARG23112	PARG23113	PARG23116
PARG23117	PARG23118	PARG23119	PARG23120	PARG23122	PARG23123	PARG23124
PARG23125	PARG23128	PARG23129	PARG23130	PARG23131	PARG23132	PARG23133
PARG23134	PARG23136	PARG23137	PARG23138	PARG23139	PARG23140	PARG23142
PARG23143	PARG23144	PARG23145	PARG23146	PARG23147	PARG23148	PARG23149
PARG23150	PARG23151	PARG23152	PARG23153	PARG23154	PARG23155	PARG23156
PARG23157	PARG23158	PARG23159	PARG23160	PARG23163	PARG23164	PARG23165
PARG23166	PARG23167	PARG23168	PARG23169	PARG23170	PARG23172	PARG23173
PARG23176	PARG23177	PARG23178	PARG23179	PARG23180	PARG23182	PARG23183
PARG23184	PARG23185	PARG23186	PARG23188	PARG23189	PARG23191	PARG23192
PARG23193	PARG23194	PARG23196	PARG23197	PARG23198	PARG23199	PARG23200
PARG23206	PARG23207	PARG23208	PARG23209	PARG23210	PARG23211	PARG23213
PARG23215	PARG23216	PARG23217	PARG23218	PARG23219	PARG23220	PARG23221
PARG23222	PARG23223	PARG23224	PARG23225	PARG23227	PARG23228	PARG23229
PARG23233	PARG23234	PARG23235	PARG23236	PARG23237	PARG23238	PARG23239
PARG23240	PARG23241	PARG23242	PARG23243	PARG23244	PARG23245	PARG23246
PARG23247	PARG23248	PARG23249	PARG23254	PARG23255	PARG23256	PARG23261
PARG23262	PARG23263	PARG23264	PARG23265	PARG23266	PARG23267	PARG23268
PARG23269	PARG23270	PARG23271	PARG23272	PARG23273	PARG23274	PARG23275
PARG23276	PARG23277	PARG23278	PARG23279	PARG23280	PARG23281	PARG23283
PARG23284	PARG23285	PARG23286	PARG23287	PARG23288	PARG23289	PARG23304
PARG23305	PARG23306	PARG23319	PARG23320	PARG23321	PARG23332	PARG23334
PARG23335	PARG23336	PARG23338	PARG23339	PARG23340	PARG23341	PARG23342
PARG23343	PARG23344	PARG23345	PARG23349	PARG23350	PARG23351	PARG23353
PARG23354	PARG23355	PARG23356	PARG23357	PARG23358	PARG23359	PARG23365
PARG23366	PARG23370	PARG23371	PARG23372	PARG23373	PARG23374	PARG23377
PARG23379	PARG23383	PARG23384	PARG23385	PARG23386	PARG23387	PARG23388
PARG23389	PARG23390	PARG23392	PARG23393	PARG23394	PARG23395	PARG23396
PARG23397	PARG23398	PARG23400	PARG23402	PARG23403	PARG23405	PARG23406
PARG23407	PARG23408	PARG23409	PARG23411	PARG23412	PARG23413	PARG23414
PARG23415	PARG23416	PARG23417	PARG23418	PARG23419	PARG23420	PARG23421
PARG23423	PARG23424	PARG23426	PARG23429	PARG23430	PARG23431	PARG23432
PARG23433	PARG23434	PARG23435	PARG23436	PARG23437	PARG23439	PARG23440
PARG23441	PARG23442	PARG23446	PARG23447	PARG23449	PARG23450	PARG23452
PARG23453	PARG23454	PARG23455	PARG23456	PARG23457	PARG23458	PARG23459
PARG23460	PARG23461	PARG23463	PARG23464	PARG23465	PARG23466	PARG23467
PARG23468	PARG23469	PARG23470	PARG23471	PARG23473	PARG23474	PARG23476
PARG23478	PARG23479	PARG23480	PARG23481	PARG23484	PARG23485	PARG23486
PARG23487	PARG23488	PARG23489	PARG23490	PARG23491	PARG23496	PARG23498
PARG23499	PARG23500	PARG23501	PARG23502	PARG23503	PARG23504	PARG23505
PARG23506	PARG23507	PARG23508	PARG23509	PARG23515	PARG23516	PARG23518
PARG23520	PARG23521	PARG23522	PARG23524	PARG23525	PARG23526	PARG23527

PARG23531	PARG23532	PARG23533	PARG23534	PARG23535	PARG23536	PARG23537
PARG23538	PARG23539	PARG23540	PARG23541	PARG23542	PARG23543	PARG23545
PARG23546	PARG23547	PARG23548	PARG23549	PARG23550	PARG23551	PARG23552
PARG23553	PARG23554	PARG23555	PARG23556	PARG23557	PARG23558	PARG23559
PARG23560	PARG23561	PARG23562	PARG23563	PARG23564	PARG23565	PARG23566
PARG23567	PARG23568	PARG23569	PARG23572	PARG23575	PARG23576	PARG23577
PARG23578	PARG23579	PARG23580	PARG23581	PARG23582	PARG23583	PARG23584
PARG23587	PARG23588	PARG23589	PARG23592	PARG23593	PARG23594	PARG23595
PARG23596	PARG23597	PARG23598	PARG23599	PARG23600	PARG23603	PARG23604
PARG23606	PARG23607	PARG23608	PARG23609	PARG23611	PARG23612	PARG23613
PARG23614	PARG23615	PARG23616	PARG23618	PARG23622	PARG23626	PARG23627
PARG23628	PARG23629	PARG23635	PARG23636	PARG23637	PARG23638	PARG23639
PARG23645	PARG23649	PARG23650	PARG23651	PARG23655	PARG23656	PARG23657
PARG23658	PARG23661	PARG23662	PARG23673	PARG23674	PARG23675	PARG23676
PARG23677	PARG23678	PARG23679	PARG23681	PARG23689	PARG23690	PARG23693
PARG23698	PARG23699	PARG23700	PARG23701	PARG23702	PARG23703	PARG23704
PARG23705	PARG23706	PARG23707	PARG23708	PARG23709	PARG23716	PARG23717
PARG23718	PARG23719	PARG23720	PARG23721	PARG23723	PARG23724	PARG23725
PARG23726	PARG23727	PARG23728	PARG23729	PARG23730	PARG23731	PARG23732
PARG23733	PARG23734	PARG23735	PARG23736	PARG23739	PARG23740	PARG23742
PARG23743	PARG23745	PARG23746	PARG23747	PARG23748	PARG23752	PARG23753
PARG23754	PARG23755	PARG23756	PARG23758	PARG23759	PARG23760	PARG23761
PARG23762	PARG23763	PARG23764	PARG23765	PARG23766	PARG23767	PARG23768
PARG23769	PARG23770	PARG23774	PARG23775	PARG23777	PARG23779	PARG23780
PARG23783	PARG23784	PARG23785	PARG23786	PARG23787	PARG23789	PARG23790
PARG23791	PARG23792	PARG23793	PARG23794	PARG23795	PARG23796	PARG23797
PARG23798	PARG23799	PARG23800	PARG23801	PARG23802	PARG23805	PARG23809
PARG23810	PARG23811	PARG23812	PARG23814	PARG23815	PARG23817	PARG23818
PARG23819	PARG23820	PARG23822	PARG23823	PARG23824	PARG23825	PARG23826
PARG23827	PARG23829	PARG23830	PARG23831	PARG23832	PARG23833	PARG23834
PARG23835	PARG23836	PARG23837	PARG23838	PARG23839	PARG23840	PARG23841
PARG23842	PARG23843	PARG23844	PARG23845	PARG23846	PARG23847	PARG23848
PARG23849	PARG23853	PARG23854	PARG23855	PARG23856	PARG23857	PARG23858
PARG23859	PARG23861	PARG23862	PARG23863	PARG23865	PARG23866	PARG23867
PARG23868	PARG23869	PARG23870	PARG23871	PARG23872	PARG23873	PARG23874
PARG23875	PARG23876	PARG23877	PARG23878	PARG23879	PARG23880	PARG23881
PARG23882	PARG23883	PARG23884	PARG23885	PARG23886	PARG23889	PARG23890
PARG23891	PARG23892	PARG23893	PARG23894	PARG23895	PARG23896	PARG23897
PARG23899	PARG23900	PARG23901	PARG23904	PARG23905	PARG23906	PARG23912
PARG23915	PARG23916	PARG23917	PARG23918	PARG23919	PARG23920	PARG23921
PARG23925	PARG23926	PARG23927	PARG23930	PARG23932	PARG23933	PARG23934
PARG23936	PARG23937	PARG23938	PARG23939	PARG23940	PARG23941	PARG23942
PARG23943	PARG23944	PARG23945	PARG23946	PARG23947	PARG23948	PARG23949
PARG23950	PARG23951	PARG23952	PARG23953	PARG23954	PARG23955	PARG23956
PARG23957	PARG23958	PARG23961	PARG23962	PARG23963	PARG23964	PARG23966
PARG23967	PARG23968	PARG23969	PARG23970	PARG23971	PARG23972	PARG23973

PARG23974	PARG23975	PARG23976	PARG23979	PARG23980	PARG23981	PARG23982
PARG23983	PARG23984	PARG23985	PARG23986	PARG23987	PARG23988	PARG23989
PARG23990	PARG23991	PARG23994	PARG23995	PARG23996	PARG23997	PARG23998
PARG23999	PARG24000	PARG24001	PARG24002	PARG24003	PARG24004	PARG24005
PARG24006	PARG24007	PARG24008	PARG24009	PARG24010	PARG24011	PARG24012
PARG24013	PARG24014	PARG24015	PARG24016	PARG24019	PARG24021	PARG24026
PARG24027	PARG24028	PARG24029	PARG24030	PARG24031	PARG24032	PARG24033
PARG24034	PARG24036	PARG24037	PARG24038	PARG24039	PARG24040	PARG24041
PARG24042	PARG24043	PARG24044	PARG24045	PARG24046	PARG24047	PARG24048
PARG24049	PARG24050	PARG24052	PARG24054	PARG24055	PARG24056	PARG24057
PARG24058	PARG24062	PARG24063	PARG24064	PARG24065	PARG24066	PARG24067
PARG24068	PARG24069	PARG24070	PARG24071	PARG24072	PARG24073	PARG24074
PARG24075	PARG24076	PARG24077	PARG24078	PARG24080	PARG24081	PARG24082
PARG24083	PARG24084	PARG24085	PARG24086	PARG24087	PARG24088	PARG24089
PARG24090	PARG24091	PARG24092	PARG24093	PARG24094	PARG24096	PARG24097
PARG24098	PARG24099	PARG24100	PARG24101	PARG24102	PARG24103	PARG24104
PARG24105	PARG24106	PARG24107	PARG24108	PARG24109	PARG24110	PARG24111
PARG24112	PARG24114	PARG24117	PARG24119	PARG24120	PARG24124	PARG24125
PARG24126	PARG24127	PARG24128	PARG24129	PARG24130	PARG24131	PARG24132
PARG24133	PARG24134	PARG24135	PARG24136	PARG24137	PARG24138	PARG24139
PARG24140	PARG24141	PARG24142	PARG24143	PARG24144	PARG24146	PARG24147
PARG24149	PARG24150	PARG24151	PARG24152	PARG24153	PARG24154	PARG24155
PARG24156	PARG24157	PARG24158	PARG24159	PARG24165	PARG24166	PARG24167
PARG24168	PARG24169	PARG24170	PARG24171	PARG24172	PARG24173	PARG24174
PARG24175	PARG24176	PARG24178	PARG24179	PARG24180	PARG24181	PARG24182
PARG24183	PARG24184	PARG24185	PARG24186	PARG24187	PARG24188	PARG24189
PARG24190	PARG24191	PARG24192	PARG24193	PARG24194	PARG24195	PARG24196
PARG24197	PARG24198	PARG24199	PARG24200	PARG24201	PARG24202	PARG24203
PARG24204	PARG24205	PARG24206	PARG24207	PARG24208	PARG24209	PARG24211
PARG24212	PARG24213	PARG24214	PARG24216	PARG24217	PARG24218	PARG24219
PARG24220	PARG24221	PARG24222	PARG24223	PARG24224	PARG24225	PARG24226
PARG24227	PARG24228	PARG24229	PARG24230	PARG24231	PARG24232	PARG24233
PARG24234	PARG24235	PARG24236	PARG24237	PARG24238	PARG24239	PARG24240
PARG24241	PARG24242	PARG24243	PARG24244	PARG24245	PARG24246	PARG24247
PARG24248	PARG24249	PARG24250	PARG24251	PARG24252	PARG24253	PARG24255
PARG24257	PARG24258	PARG24260	PARG24261	PARG24262	PARG24263	PARG24264
PARG24265	PARG24266	PARG24267	PARG24268	PARG24269	PARG24270	PARG24271
PARG24272	PARG24273	PARG24274	PARG24275	PARG24276	PARG24277	PARG24278
PARG24279	PARG24280	PARG24281	PARG24284	PARG24285	PARG24286	PARG24287
PARG24288	PARG24289	PARG24290	PARG24292	PARG24293	PARG24294	PARG24297
PARG24299	PARG24300	PARG24301	PARG24302	PARG24303	PARG24304	PARG24305
PARG24306	PARG24307	PARG24308	PARG24312	PARG24314	PARG24315	PARG24320
PARG24324	PARG24325	PARG24327	PARG24328	PARG24329	PARG24331	PARG24332
PARG24333	PARG24334	PARG24335	PARG24336	PARG24337	PARG24338	PARG24340
PARG24341	PARG24342	PARG24343	PARG24344	PARG24345	PARG24346	PARG24347
PARG24348	PARG24349	PARG24350	PARG24351	PARG24356	PARG24357	PARG24358

PARG24359	PARG24360	PARG24361	PARG24362	PARG24363	PARG24365	PARG24366
PARG24367	PARG24368	PARG24369	PARG24370	PARG24371	PARG24372	PARG24373
PARG24374	PARG24376	PARG24377	PARG24378	PARG24379	PARG24380	PARG24381
PARG24382	PARG24383	PARG24384	PARG24385	PARG24386	PARG24387	PARG24388
PARG24389	PARG24390	PARG24391	PARG24392	PARG24393	PARG24395	PARG24396
PARG24397	PARG24399	PARG24400	PARG24402	PARG24403	PARG24404	PARG24405
PARG24406	PARG24407	PARG24408	PARG24409	PARG24410	PARG24411	PARG24412
PARG24413	PARG24414	PARG24415	PARG24416	PARG24417	PARG24418	PARG24419
PARG24420	PARG24421	PARG24422	PARG24423	PARG24425	PARG24426	PARG24427
PARG24428	PARG24429	PARG24430	PARG24431	PARG24432	PARG24433	PARG24434
PARG24435	PARG24436	PARG24437	PARG24438	PARG24440	PARG24441	PARG24442
PARG24443	PARG24444	PARG24445	PARG24446	PARG24447	PARG24449	PARG24450
PARG24451	PARG24453	PARG24454	PARG24455	PARG24456	PARG24457	PARG24458
PARG24459	PARG24460	PARG24461	PARG24462	PARG24463	PARG24465	PARG24466
PARG24467	PARG24468	PARG24469	PARG24470	PARG24471	PARG24472	PARG24473
PARG24475	PARG24477	PARG24479	PARG24480	PARG24481	PARG24482	PARG24487
PARG24488	PARG24489	PARG24490	PARG24491	PARG24492	PARG24493	PARG24494
PARG24495	PARG24496	PARG24497	PARG24498	PARG24499	PARG24500	PARG24501
PARG24502	PARG24504	PARG24505	PARG24507	PARG24508	PARG24509	PARG24510
PARG24511	PARG24512	PARG24513	PARG24514	PARG24515	PARG24516	PARG24519
PARG24520	PARG24521	PARG24522	PARG24523	PARG24524	PARG24525	PARG24528
PARG24530	PARG24531	PARG24532	PARG24533	PARG24534	PARG24536	PARG24538
PARG24539	PARG24540	PARG24541	PARG24542	PARG24543	PARG24544	PARG24545
PARG24546	PARG24548	PARG24549	PARG24550	PARG24551	PARG24552	PARG24553
PARG24554	PARG24555	PARG24556	PARG24557	PARG24558	PARG24559	PARG24560
PARG24561	PARG24562	PARG24564	PARG24566	PARG24567	PARG24568	PARG24569
PARG24570	PARG24573	PARG24574	PARG24575	PARG24576	PARG24577	PARG24578
PARG24579	PARG24582	PARG24584	PARG24585	PARG24586	PARG24587	PARG24589
PARG24590	PARG24591	PARG24592	PARG24594	PARG24595	PARG24597	PARG24598
PARG24599	PARG24600	PARG24601	PARG24602	PARG24603	PARG24604	PARG24605
PARG24607	PARG24608	PARG24609	PARG24610	PARG24611	PARG24612	PARG24613
PARG24614	PARG24615	PARG24616	PARG24617	PARG24618	PARG24619	PARG24620
PARG24621	PARG24622	PARG24623	PARG24624	PARG24625	PARG24626	PARG24627
PARG24628	PARG24629	PARG24630	PARG24631	PARG24632	PARG24633	PARG24634
PARG24635	PARG24636	PARG24637	PARG24638	PARG24640	PARG24641	PARG24642
PARG24643	PARG24644	PARG24645	PARG24646	PARG24647	PARG24648	PARG24649
PARG24652	PARG24653	PARG24654	PARG24655	PARG24656	PARG24657	PARG24658
PARG24659	PARG24660	PARG24661	PARG24662	PARG24663	PARG24664	PARG24665
PARG24666	PARG24667	PARG24668	PARG24669	PARG24670	PARG24671	PARG24672
PARG24673	PARG24674	PARG24675	PARG24676	PARG24677	PARG24678	PARG24679
PARG24680	PARG24681	PARG24682	PARG24683	PARG24684	PARG24685	PARG24686
PARG24687	PARG24688	PARG24689	PARG24690	PARG24691	PARG24692	PARG24693
PARG24696	PARG24697	PARG24698	PARG24699	PARG24701	PARG24702	PARG24703
PARG24704	PARG24705	PARG24706	PARG24707	PARG24709	PARG24710	PARG24711
PARG24712	PARG24713	PARG24714	PARG24716	PARG24717	PARG24718	PARG24719
PARG24720	PARG24721	PARG24722	PARG24723	PARG24724	PARG24725	PARG24726

PARG24727	PARG24728	PARG24729	PARG24730	PARG24731	PARG24732	PARG24733
PARG24734	PARG24735	PARG24736	PARG24737	PARG24738	PARG24739	PARG24740
PARG24741	PARG24742	PARG24743	PARG24744	PARG24745	PARG24746	PARG24747
PARG24748	PARG24749	PARG24750	PARG24751	PARG24752	PARG24753	PARG24754
PARG24755	PARG24756	PARG24757	PARG24758	PARG24759	PARG24760	PARG24761
PARG24762	PARG24763	PARG24764	PARG24765	PARG24766	PARG24767	PARG24768
PARG24769	PARG24770	PARG24771	PARG24772	PARG24773	PARG24774	PARG24775
PARG24776	PARG24777	PARG24778	PARG24779	PARG24780	PARG24781	PARG24782
PARG24783	PARG24784	PARG24785	PARG24786	PARG24787	PARG24788	PARG24793
PARG24794	PARG24803	PARG24811	PARG24813	PARG24814	PARG24815	PARG24816
PARG24817	PARG24818	PARG24820	PARG24821	PARG24822	PARG24823	PARG24824
PARG24825	PARG24826	PARG24827	PARG24828	PARG24829	PARG24830	PARG24831
PARG24832	PARG24833	PARG24834	PARG24835	PARG24836	PARG24837	PARG24838
PARG24839	PARG24840	PARG24841	PARG24842	PARG24843	PARG24844	PARG24845
PARG24846	PARG24847	PARG24848	PARG24849	PARG24850	PARG24851	PARG24852
PARG24853	PARG24854	PARG24855	PARG24856	PARG24857	PARG24858	PARG24859
PARG24862	PARG24864	PARG24865	PARG24890	PARG24891	PARG24892	PARG24893
PARG24895	PARG24896	PARG24897	PARG24898	PARG24899	PARG24900	PARG24901
PARG24902	PARG24903	PARG24904	PARG24905	PARG24906	PARG24907	PARG24908
PARG24909	PARG24910	PARG24911	PARG24912	PARG24913	PARG24914	PARG24915
PARG24916	PARG24917	PARG24918	PARG24919	PARG24920	PARG24921	PARG24922
PARG24923	PARG24924	PARG24925	PARG24926	PARG24928	PARG24929	PARG24930
PARG24931	PARG24932	PARG24935	PARG24936	PARG24939	PARG24941	PARG24942
PARG24943	PARG24944	PARG24945	PARG24946	PARG24947	PARG24950	PARG24951
PARG24952	PARG24953	PARG24954	PARG24955	PARG24956	PARG24957	PARG24958
PARG24959	PARG24960	PARG24961	PARG24962	PARG24963	PARG24964	PARG24965
PARG24966	PARG24967	PARG24968	PARG24969	PARG24970	PARG24971	PARG24972
PARG24973	PARG24974	PARG24975	PARG24976	PARG24977	PARG24978	PARG24979
PARG24980	PARG24981	PARG24982	PARG24983	PARG24984	PARG24985	PARG24986
PARG24987	PARG24988	PARG24989	PARG24991	PARG24992	PARG24993	PARG24995
PARG24996	PARG24997	PARG24998	PARG25001	PARG25002	PARG25003	PARG25004
PARG25006	PARG25007	PARG25008	PARG25009	PARG25010	PARG25011	PARG25012
PARG25013	PARG25014	PARG25016	PARG25017	PARG25018	PARG25019	PARG25020
PARG25021	PARG25022	PARG25023	PARG25026	PARG25028	PARG25029	PARG25030
PARG25032	PARG25033	PARG25035	PARG25036	PARG25037	PARG25038	PARG25039
PARG25040	PARG25041	PARG25042	PARG25043	PARG25044	PARG25045	PARG25046
PARG25047	PARG25048	PARG25049	PARG25050	PARG25051	PARG25052	PARG25053
PARG25055	PARG25056	PARG25057	PARG25058	PARG25059	PARG25061	PARG25063
PARG25065	PARG25066	PARG25067	PARG25068	PARG25069	PARG25070	PARG25071
PARG25072	PARG25073	PARG25074	PARG25075	PARG25076	PARG25077	PARG25078
PARG25079	PARG25080	PARG25081	PARG25083	PARG25084	PARG25085	PARG25086
PARG25087	PARG25088	PARG25089	PARG25090	PARG25091	PARG25092	PARG25093
PARG25096	PARG25097	PARG25098	PARG25099	PARG25100	PARG25101	PARG25102
PARG25103	PARG25104	PARG25105	PARG25106	PARG25107	PARG25108	PARG25109
PARG25110	PARG25111	PARG25112	PARG25113	PARG25114	PARG25115	PARG25116
PARG25117	PARG25118	PARG25119	PARG25120	PARG25121	PARG25122	PARG25123

PARG25124	PARG25126	PARG25127	PARG25128	PARG25129	PARG25130	PARG25131
PARG25132	PARG25133	PARG25134	PARG25135	PARG25136	PARG25137	PARG25138
PARG25139	PARG25141	PARG25143	PARG25144	PARG25145	PARG25146	PARG25147
PARG25148	PARG25149	PARG25152	PARG25153	PARG25154	PARG25155	PARG25156
PARG25157	PARG25158	PARG25159	PARG25161	PARG25163	PARG25164	PARG25165
PARG25166	PARG25167	PARG25168	PARG25170	PARG25171	PARG25172	PARG25173
PARG25174	PARG25175	PARG25176	PARG25177	PARG25179	PARG25180	PARG25181
PARG25182	PARG25183	PARG25184	PARG25185	PARG25186	PARG25187	PARG25188
PARG25189	PARG25190	PARG25191	PARG25192	PARG25193	PARG25194	PARG25195
PARG25196	PARG25197	PARG25198	PARG25199	PARG25200	PARG25201	PARG25202
PARG25203	PARG25204	PARG25205	PARG25206	PARG25207	PARG25208	PARG25209
PARG25210	PARG25211	PARG25213	PARG25214	PARG25215	PARG25216	PARG25217
PARG25218	PARG25219	PARG25220	PARG25221	PARG25222	PARG25223	PARG25224
PARG25225	PARG25226	PARG25227	PARG25228	PARG25229	PARG25230	PARG25231
PARG25232	PARG25233	PARG25234	PARG25235	PARG25236	PARG25237	PARG25238
PARG25239	PARG25240	PARG25241	PARG25242	PARG25243	PARG25244	PARG25245
PARG25246	PARG25248	PARG25249	PARG25252	PARG25253	PARG25255	PARG25259
PARG25260	PARG25261	PARG25262	PARG25263	PARG25266	PARG25267	PARG25268
PARG25270	PARG25271	PARG25272	PARG25273	PARG25276	PARG25277	PARG25278
PARG25279	PARG25280	PARG25281	PARG25282	PARG25283	PARG25284	PARG25285
PARG25286	PARG25287	PARG25288	PARG25289	PARG25290	PARG25291	PARG25292
PARG25293	PARG25294	PARG25295	PARG25296	PARG25297	PARG25298	PARG25299
PARG25300	PARG25301	PARG25302	PARG25304	PARG25305	PARG25306	PARG25307
PARG25308	PARG25309	PARG25310	PARG25311	PARG25312	PARG25313	PARG25314
PARG25315	PARG25316	PARG25317	PARG25318	PARG25319	PARG25320	PARG25321
PARG25322	PARG25323	PARG25324	PARG25325	PARG25326	PARG25327	PARG25328
PARG25330	PARG25331	PARG25332	PARG25333	PARG25334	PARG25335	PARG25336
PARG25337	PARG25338	PARG25339	PARG25340	PARG25341	PARG25343	PARG25344
PARG25345	PARG25346	PARG25349	PARG25350	PARG25353	PARG25354	PARG25356
PARG25359	PARG25361	PARG25362	PARG25363	PARG25364	PARG25365	PARG25371
PARG25372	PARG25373	PARG25375	PARG25376	PARG25377	PARG25378	PARG25379
PARG25381	PARG25382	PARG25383	PARG25384	PARG25385	PARG25386	PARG25387
PARG25388	PARG25389	PARG25390	PARG25395	PARG25396	PARG25397	PARG25398
PARG25399	PARG25400	PARG25401	PARG25402	PARG25403	PARG25404	PARG25405
PARG25406	PARG25407	PARG25408	PARG25409	PARG25410	PARG25411	PARG25414
PARG25415	PARG25417	PARG25421	PARG25422	PARG25424	PARG25425	PARG25434
PARG25435	PARG25438	PARG25440	PARG25441	PARG25442	PARG25444	PARG25446
PARG25448	PARG25449	PARG25451	PARG25452	PARG25453	PARG25454	PARG25455
PARG25456	PARG25457	PARG25458	PARG25463	PARG25472	PARG25479	PARG25480
PARG25481	PARG25482	PARG25491	PARG25501	PARG25507	PARG25508	PARG25509
PARG25518	PARG25522	PARG25523	PARG25532	PARG25536	PARG25537	PARG25538
PARG25539	PARG25540	PARG25547	PARG25548	PARG25549	PARG25550	PARG25554
PARG25555	PARG25556	PARG25557	PARG25558	PARG25560	PARG25561	PARG25563
PARG25564	PARG25565	PARG25570	PARG25572	PARG25573	PARG25576	PARG25577
PARG25579	PARG25580	PARG25581	PARG25582	PARG25584	PARG25585	PARG25589
PARG25590	PARG25591	PARG25592	PARG25594	PARG25595	PARG25596	PARG25597

PARG25602	PARG25603	PARG25604	PARG25605	PARG25606	PARG25607	PARG25608
PARG25611	PARG25612	PARG25613	PARG25616	PARG25617	PARG25621	PARG25626
PARG25627	PARG25629	PARG25630	PARG25631	PARG25632	PARG25633	PARG25635
PARG25637	PARG25640	PARG25644	PARG25645	PARG25646	PARG25647	PARG25648
PARG25649	PARG25652	PARG25653	PARG25658	PARG25659	PARG25660	PARG25664
PARG25665	PARG25668	PARG25669	PARG25672	PARG25673	PARG25677	PARG25678
PARG25679	PARG25680	PARG25682	PARG25683	PARG25684	PARG25688	PARG25689
PARG25690	PARG25702	PARG25703	PARG25704	PARG25705	PARG25706	PARG25707
PARG25708	PARG25713	PARG25714	PARG25715	PARG25720	PARG25721	PARG25727
PARG25728	PARG25731	PARG25734	PARG25735	PARG25736	PARG25737	PARG25738
PARG25739	PARG25740	PARG25741	PARG25743	PARG25744	PARG25749	PARG25750
PARG25751	PARG25752	PARG25753	PARG25754	PARG25755	PARG25756	PARG25757
PARG25758	PARG25759	PARG25761	PARG25762	PARG25763	PARG25764	PARG25765
PARG25767	PARG25768	PARG25769	PARG25770	PARG25787	PARG25790	PARG25791
PARG25798	PARG25800	PARG25801	PARG25804	PARG25808	PARG25809	PARG25811
PARG25812	PARG25814	PARG25815	PARG25817	PARG25818	PARG25821	PARG25822
PARG25832	PARG25841	PARG25843	PARG25845	PARG25850	PARG25851	PARG25856
PARG25858	PARG25863	PARG25864	PARG25865	PARG25878	PARG25879	PARG25881
PARG25890	PARG25895	PARG25896	PARG25899	PARG25900	PARG25907	PARG25908
PARG25909	PARG25910	PARG25911	PARG25913	PARG25916	PARG25919	PARG25920
PARG25924	PARG25925	PARG25926	PARG25930	PARG25931	PARG25932	PARG25933
PARG25934	PARG25936	PARG25937	PARG25938	PARG25940	PARG25941	PARG25942
PARG25944	PARG25945	PARG25946	PARG25948	PARG25949	PARG25950	PARG25952
PARG25953	PARG25954	PARG25955	PARG25958	PARG25960	PARG25972	PARG25975
PARG25976	PARG25977	PARG25979	PARG25980	PARG25981	PARG25983	PARG25984
PARG25985	PARG25986	PARG25987	PARG25988	PARG25994	PARG25998	PARG25999
PARG26000	PARG26002	PARG26003	PARG26008	PARG26018	PARG26019	PARG26020
PARG26025	PARG26029	PARG26030	PARG26031	PARG26032	PARG26033	PARG26038
PARG26039	PARG26040	PARG26041	PARG26042	PARG26043	PARG26044	PARG26046
PARG26047	PARG26049	PARG26050	PARG26054	PARG26056	PARG26057	PARG26058
PARG26061	PARG26062	PARG26063	PARG26064	PARG26066	PARG26067	PARG26069
PARG26070	PARG26071	PARG26075	PARG26076	PARG26077	PARG26078	PARG26080
PARG26085	PARG26086	PARG26092	PARG26094	PARG26098	PARG26100	PARG26101
PARG26110	PARG26112	PARG26113	PARG26114	PARG26122	PARG26135	PARG26138
PARG26139	PARG26143	PARG26144	PARG26145	PARG26146	PARG26148	PARG26149
PARG26153	PARG26154	PARG26156	PARG26159	PARG26160	PARG26162	PARG26168
PARG26173	PARG26175	PARG26177	PARG26181	PARG26182	PARG26183	PARG26184
PARG26185	PARG26186	PARG26189	PARG26193	PARG26195	PARG26203	PARG26204
PARG26205	PARG26208	PARG26211	PARG26212	PARG26215	PARG26216	PARG26217
PARG26218	PARG26221	PARG26222	PARG26223	PARG26226	PARG26232	PARG26233
PARG26238	PARG26242	PARG26245	PARG26246	PARG26247	PARG26248	PARG26249
PARG26250	PARG26252	PARG26253	PARG26259	PARG26261	PARG26264	PARG26265
PARG26273	PARG26274	PARG26280	PARG26284	PARG26285	PARG26289	PARG26290
PARG26293	PARG26294	PARG26295	PARG26296	PARG26299	PARG26300	PARG26302
PARG26305	PARG26306	PARG26307	PARG26308	PARG26309	PARG26313	PARG26317
PARG26318	PARG26319	PARG26325	PARG26326	PARG26327	PARG26329	PARG26330

PARG26331	PARG26332	PARG26335	PARG26336	PARG26339	PARG26340	PARG26341
PARG26342	PARG26343	PARG26344	PARG26345	PARG26346	PARG26347	PARG26348
PARG26349	PARG26350	PARG26351	PARG26355	PARG26356	PARG26357	PARG26358
PARG26359	PARG26360	PARG26361	PARG26362	PARG26363	PARG26366	PARG26367
PARG26368	PARG26369	PARG26372	PARG26373	PARG26374	PARG26375	PARG26376
PARG26377	PARG26378	PARG26379	PARG26381	PARG26382	PARG26385	PARG26386
PARG26387	PARG26388	PARG26389	PARG26390	PARG26393	PARG26397	PARG26398
PARG26399	PARG26400	PARG26402	PARG26404	PARG26405	PARG26406	PARG26407
PARG26408	PARG26409	PARG26410	PARG26411	PARG26412	PARG26413	PARG26414
PARG26415	PARG26416	PARG26417	PARG26418	PARG26419	PARG26420	PARG26423
PARG26424	PARG26425	PARG26426	PARG26427	PARG26428	PARG26429	PARG26430
PARG26431	PARG26432	PARG26434	PARG26435	PARG26436	PARG26437	PARG26441
PARG26442	PARG26443	PARG26444	PARG26445	PARG26446	PARG26447	PARG26448
PARG26449	PARG26450	PARG26451	PARG26452	PARG26453	PARG26454	PARG26455
PARG26456	PARG26457	PARG26459	PARG26460	PARG26461	PARG26462	PARG26463
PARG26464	PARG26465	PARG26466	PARG26468	PARG26469	PARG26470	PARG26471
PARG26472	PARG26473	PARG26474	PARG26475	PARG26477	PARG26478	PARG26479
PARG26480	PARG26481	PARG26482	PARG26484	PARG26485	PARG26486	PARG26487
PARG26488	PARG26489	PARG26490	PARG26491	PARG26492	PARG26493	PARG26494
PARG26496	PARG26497	PARG26498	PARG26499	PARG26500	PARG26501	PARG26503
PARG26505	PARG26506	PARG26507	PARG26509	PARG26510	PARG26512	PARG26513
PARG26514	PARG26515	PARG26516	PARG26517	PARG26520	PARG26521	PARG26522
PARG26523	PARG26527	PARG26528	PARG26529	PARG26530	PARG26531	PARG26532
PARG26533	PARG26534	PARG26535	PARG26536	PARG26537	PARG26539	PARG26540
PARG26541	PARG26542	PARG26543	PARG26544	PARG26545	PARG26546	PARG26547
PARG26548	PARG26549	PARG26551	PARG26552	PARG26553	PARG26554	PARG26555
PARG26556	PARG26557	PARG26558	PARG26560	PARG26561	PARG26562	PARG26563
PARG26564	PARG26565	PARG26566	PARG26567	PARG26569	PARG26570	PARG26571
PARG26572	PARG26573	PARG26574	PARG26575	PARG26576	PARG26577	PARG26578
PARG26579	PARG26581	PARG26582	PARG26583	PARG26586	PARG26587	PARG26588
PARG26589	PARG26590	PARG26591	PARG26592	PARG26593	PARG26594	PARG26595
PARG26596	PARG26598	PARG26599	PARG26600	PARG26601	PARG26603	PARG26604
PARG26605	PARG26606	PARG26611	PARG26612	PARG26613	PARG26614	PARG26615
PARG26616	PARG26617	PARG26618	PARG26619	PARG26620	PARG26621	PARG26622
PARG26623	PARG26624	PARG26627	PARG26632	PARG26633	PARG26634	PARG26639
PARG26640	PARG26641	PARG26642	PARG26644	PARG26645	PARG26646	PARG26648
PARG26649	PARG26650	PARG26651	PARG26652	PARG26653	PARG26654	PARG26655
PARG26656	PARG26657	PARG26658	PARG26659	PARG26660	PARG26661	PARG26662
PARG26663	PARG26664	PARG26665	PARG26666	PARG26667	PARG26668	PARG26669
PARG26670	PARG26671	PARG26672	PARG26673	PARG26674	PARG26675	PARG26676
PARG26677	PARG26678	PARG26679	PARG26680	PARG26681	PARG26682	PARG26683
PARG26684	PARG26686	PARG26687	PARG26688	PARG26689	PARG26690	PARG26691
PARG26692	PARG26693	PARG26694	PARG26695	PARG26696	PARG26697	PARG26698
PARG26699	PARG26700	PARG26701	PARG26702	PARG26703	PARG26704	PARG26705
PARG26706	PARG26707	PARG26708	PARG26709	PARG26710	PARG26711	PARG26712
PARG26713	PARG26714	PARG26715	PARG26716	PARG26717	PARG26718	PARG26719

PARG26721	PARG26723	PARG26724	PARG26725	PARG26727	PARG26728	PARG26729
PARG26730	PARG26732	PARG26733	PARG26734	PARG26735	PARG26736	PARG26737
PARG26738	PARG26739	PARG26740	PARG26742	PARG26743	PARG26747	PARG26750
PARG26751	PARG26752	PARG26757	PARG26763	PARG26770	PARG26773	PARG26776
PARG26777	PARG26778	PARG26779	PARG26780	PARG26781	PARG26782	PARG26784
PARG26785	PARG26786	PARG26787	PARG26789	PARG26790	PARG26791	PARG26792
PARG26793	PARG26794	PARG26795	PARG26796	PARG26797	PARG26798	PARG26799
PARG26800	PARG26801	PARG26802	PARG26803	PARG26804	PARG26805	PARG26806
PARG26807	PARG26808	PARG26809	PARG26810	PARG26811	PARG26812	PARG26813
PARG26814	PARG26815	PARG26816	PARG26818	PARG26819	PARG26820	PARG26821
PARG26822	PARG26824	PARG26825	PARG26827	PARG26829	PARG26830	PARG26831
PARG26832	PARG26833	PARG26834	PARG26835	PARG26836	PARG26837	PARG26838
PARG26839	PARG26840	PARG26841	PARG26842	PARG26843	PARG26844	PARG26845
PARG26846	PARG26848	PARG26849	PARG26850	PARG26852	PARG26853	PARG26854
PARG26855	PARG26856	PARG26857	PARG26858	PARG26859	PARG26860	PARG26861
PARG26862	PARG26864	PARG26865	PARG26866	PARG26867	PARG26868	PARG26869
PARG26870	PARG26871	PARG26872	PARG26873	PARG26874	PARG26875	PARG26876
PARG26877	PARG26880	PARG26881	PARG26882	PARG26883	PARG26884	PARG26885
PARG26888	PARG26890	PARG26892	PARG26894	PARG26895	PARG26896	PARG26897
PARG26898	PARG26902	PARG26903	PARG26904	PARG26906	PARG26907	PARG26908
PARG26909	PARG26910	PARG26911	PARG26912	PARG26913	PARG26914	PARG26915
PARG26916	PARG26917	PARG26918	PARG26919	PARG26920	PARG26921	PARG26922
PARG26923	PARG26924	PARG26925	PARG26926	PARG26927	PARG26928	PARG26929
PARG26930	PARG26931	PARG26932	PARG26933	PARG26934	PARG26935	PARG26936
PARG26937	PARG26938	PARG26939	PARG26940	PARG26941	PARG26942	PARG26943
PARG26944	PARG26945	PARG26946	PARG26950	PARG26955	PARG26959	PARG26960
PARG26961	PARG26962	PARG26963	PARG26964	PARG26965	PARG26969	PARG26970
PARG26971	PARG26973	PARG26974	PARG26975	PARG26979	PARG26980	PARG26981
PARG26983	PARG26984	PARG26985	PARG26986	PARG26987	PARG26988	PARG26989
PARG26991	PARG26993	PARG26994	PARG26995	PARG26997	PARG27000	PARG27001
PARG27002	PARG27004	PARG27013	PARG27015	PARG27016	PARG27017	PARG27018
PARG27019	PARG27020	PARG27021	PARG27022	PARG27023	PARG27024	PARG27025
PARG27026	PARG27027	PARG27028	PARG27031	PARG27032	PARG27033	PARG27034
PARG27035	PARG27036	PARG27037	PARG27038	PARG27039	PARG27040	PARG27041
PARG27042	PARG27043	PARG27044	PARG27045	PARG27046	PARG27047	PARG27048
PARG27049	PARG27050	PARG27051	PARG27052	PARG27053	PARG27054	PARG27055
PARG27056	PARG27057	PARG27059	PARG27061	PARG27062	PARG27063	PARG27064
PARG27065	PARG27066	PARG27068	PARG27069	PARG27070	PARG27071	PARG27072
PARG27073	PARG27074	PARG27075	PARG27076	PARG27077	PARG27078	PARG27079
PARG27080	PARG27082	PARG27083	PARG27084	PARG27085	PARG27086	PARG27087
PARG27089	PARG27091	PARG27093	PARG27094	PARG27095	PARG27096	PARG27098
PARG27099	PARG27100	PARG27101	PARG27102	PARG27103	PARG27104	PARG27107
PARG27108	PARG27109	PARG27110	PARG27111	PARG27113	PARG27115	PARG27116
PARG27117	PARG27118	PARG27119	PARG27122	PARG27123	PARG27124	PARG27125
PARG27126	PARG27128	PARG27129	PARG27130	PARG27131	PARG27133	PARG27135
PARG27136	PARG27137	PARG27138	PARG27139	PARG27140	PARG27141	PARG27142

PARG27144	PARG27145	PARG27146	PARG27147	PARG27148	PARG27149	PARG27150
PARG27151	PARG27152	PARG27153	PARG27154	PARG27155	PARG27156	PARG27157
PARG27158	PARG27159	PARG27160	PARG27161	PARG27162	PARG27163	PARG27164
PARG27165	PARG27166	PARG27167	PARG27170	PARG27171	PARG27173	PARG27174
PARG27175	PARG27177	PARG27178	PARG27179	PARG27180	PARG27181	PARG27182
PARG27183	PARG27184	PARG27185	PARG27186	PARG27187	PARG27188	PARG27189
PARG27190	PARG27191	PARG27192	PARG27193	PARG27194	PARG27195	PARG27196
PARG27197	PARG27198	PARG27199	PARG27200	PARG27201	PARG27202	PARG27203
PARG27204	PARG27205	PARG27206	PARG27207	PARG27208	PARG27209	PARG27210
PARG27211	PARG27212	PARG27213	PARG27214	PARG27215	PARG27216	PARG27217
PARG27218	PARG27219	PARG27220	PARG27221	PARG27222	PARG27223	PARG27224
PARG27225	PARG27226	PARG27227	PARG27228	PARG27229	PARG27230	PARG27231
PARG27232	PARG27233	PARG27235	PARG27236	PARG27237	PARG27238	PARG27239
PARG27240	PARG27241	PARG27242	PARG27243	PARG27244	PARG27245	PARG27246
PARG27248	PARG27249	PARG27250	PARG27251	PARG27252	PARG27253	PARG27254
PARG27255	PARG27256	PARG27257	PARG27258	PARG27259	PARG27260	PARG27261
PARG27262	PARG27263	PARG27264	PARG27265	PARG27266	PARG27267	PARG27268
PARG27269	PARG27270	PARG27271	PARG27272	PARG27273	PARG27274	PARG27275
PARG27276	PARG27277	PARG27278	PARG27279	PARG27281	PARG27282	PARG27283
PARG27284	PARG27285	PARG27286	PARG27287	PARG27288	PARG27289	PARG27291
PARG27292	PARG27293	PARG27294	PARG27295	PARG27296	PARG27297	PARG27298
PARG27299	PARG27300	PARG27301	PARG27302	PARG27303	PARG27304	PARG27305
PARG27306	PARG27307	PARG27308	PARG27309	PARG27310	PARG27311	PARG27312
PARG27313	PARG27314	PARG27315	PARG27316	PARG27317	PARG27318	PARG27319
PARG27320	PARG27321	PARG27322	PARG27323	PARG27324	PARG27325	PARG27326
PARG27327	PARG27328	PARG27329	PARG27330	PARG27331	PARG27333	PARG27334
PARG27335	PARG27336	PARG27337	PARG27338	PARG27339	PARG27340	PARG27341
PARG27342	PARG27343	PARG27344	PARG27345	PARG27346	PARG27347	PARG27348
PARG27350	PARG27352	PARG27353	PARG27354	PARG27355	PARG27356	PARG27357
PARG27358	PARG27359	PARG27360	PARG27361	PARG27362	PARG27363	PARG27364
PARG27365	PARG27366	PARG27367	PARG27369	PARG27370	PARG27371	PARG27372
PARG27373	PARG27374	PARG27375	PARG27376	PARG27377	PARG27378	PARG27379
PARG27380	PARG27381	PARG27382	PARG27383	PARG27384	PARG27385	PARG27386
PARG27387	PARG27388	PARG27389	PARG27390	PARG27391	PARG27392	PARG27393
PARG27394	PARG27395	PARG27396	PARG27397	PARG27398	PARG27399	PARG27401
PARG27402	PARG27403	PARG27404	PARG27405	PARG27409	PARG27410	PARG27411
PARG27412	PARG27414	PARG27415	PARG27416	PARG27417	PARG27418	PARG27419
PARG27420	PARG27421	PARG27423	PARG27424	PARG27425	PARG27426	PARG27427
PARG27428	PARG27429	PARG27430	PARG27432	PARG27433	PARG27434	PARG27435
PARG27437	PARG27438	PARG27439	PARG27440	PARG27441	PARG27442	PARG27443
PARG27445	PARG27446	PARG27449	PARG27450	PARG27451	PARG27452	PARG27453
PARG27454	PARG27455	PARG27456	PARG27457	PARG27458	PARG27459	PARG27460
PARG27461	PARG27462	PARG27464	PARG27465	PARG27466	PARG27468	PARG27470
PARG27471	PARG27472	PARG27473	PARG27474	PARG27475	PARG27476	PARG27477
PARG27478	PARG27479	PARG27480	PARG27481	PARG27483	PARG27485	PARG27486
PARG27487	PARG27489	PARG27490	PARG27491	PARG27494	PARG27495	PARG27496

PARG27497	PARG27498	PARG27499	PARG27500	PARG27501	PARG27502	PARG27503
PARG27504	PARG27507	PARG27508	PARG27509	PARG27510	PARG27511	PARG27512
PARG27513	PARG27514	PARG27516	PARG27517	PARG27518	PARG27519	PARG27520
PARG27521	PARG27524	PARG27525	PARG27526	PARG27527	PARG27528	PARG27529
PARG27530	PARG27531	PARG27532	PARG27533	PARG27534	PARG27535	PARG27538
PARG27539	PARG27540	PARG27541	PARG27542	PARG27543	PARG27544	PARG27545
PARG27546	PARG27547	PARG27550	PARG27551	PARG27557	PARG27558	PARG27559
PARG27560	PARG27562	PARG27563	PARG27564	PARG27565	PARG27566	PARG27567
PARG27568	PARG27570	PARG27574	PARG27576	PARG27577	PARG27578	PARG27579
PARG27580	PARG27581	PARG27582	PARG27583	PARG27584	PARG27585	PARG27586
PARG27587	PARG27588	PARG27589	PARG27590	PARG27591	PARG27592	PARG27593
PARG27594	PARG27595	PARG27596	PARG27597	PARG27598	PARG27599	PARG27600
PARG27603	PARG27604	PARG27605	PARG27606	PARG27607	PARG27608	PARG27609
PARG27610	PARG27611	PARG27612	PARG27613	PARG27614	PARG27615	PARG27616
PARG27617	PARG27618	PARG27620	PARG27623	PARG27624	PARG27625	PARG27626
PARG27627	PARG27628	PARG27629	PARG27630	PARG27631	PARG27632	PARG27633
PARG27634	PARG27635	PARG27636	PARG27637	PARG27638	PARG27639	PARG27640
PARG27641	PARG27642	PARG27643	PARG27644	PARG27645	PARG27646	PARG27647
PARG27648	PARG27649	PARG27650	PARG27651	PARG27652	PARG27653	PARG27654
PARG27655	PARG27656	PARG27657	PARG27658	PARG27659	PARG27660	PARG27661
PARG27662	PARG27663	PARG27664	PARG27665	PARG27666	PARG27667	PARG27668
PARG27669	PARG27671	PARG27673	PARG27674	PARG27675	PARG27676	PARG27677
PARG27678	PARG27679	PARG27680	PARG27681	PARG27682	PARG27684	PARG27685
PARG27686	PARG27687	PARG27688	PARG27689	PARG27690	PARG27691	PARG27692
PARG27693	PARG27695	PARG27696	PARG27697	PARG27698	PARG27699	PARG27700
PARG27701	PARG27702	PARG27703	PARG27704	PARG27706	PARG27707	PARG27708
PARG27709	PARG27710	PARG27711	PARG27712	PARG27713	PARG27716	PARG27717
PARG27718	PARG27719	PARG27720	PARG27721	PARG27722	PARG27723	PARG27724
PARG27725	PARG27726	PARG27727	PARG27728	PARG27729	PARG27730	PARG27731
PARG27732	PARG27733	PARG27734	PARG27735	PARG27736	PARG27737	PARG27738
PARG27739	PARG27740	PARG27741	PARG27742	PARG27743	PARG27744	PARG27745
PARG27746	PARG27747	PARG27748	PARG27749	PARG27750	PARG27751	PARG27752
PARG27753	PARG27754	PARG27755	PARG27756	PARG27757	PARG27758	PARG27759
PARG27761	PARG27763	PARG27764	PARG27765	PARG27766	PARG27769	PARG27770
PARG27771	PARG27772	PARG27773	PARG27774	PARG27775	PARG27776	PARG27777
PARG27778	PARG27779	PARG27780	PARG27781	PARG27782	PARG27785	PARG27786
PARG27787	PARG27789	PARG27791	PARG27794	PARG27796	PARG27798	PARG27799
PARG27800	PARG27801	PARG27802	PARG27803	PARG27804	PARG27805	PARG27806
PARG27807	PARG27808	PARG27809	PARG27810	PARG27811	PARG27812	PARG27813
PARG27814	PARG27815	PARG27816	PARG27817	PARG27818	PARG27819	PARG27820
PARG27821	PARG27822	PARG27823	PARG27824	PARG27825	PARG27826	PARG27827
PARG27828	PARG27829	PARG27830	PARG27831	PARG27832	PARG27835	PARG27836
PARG27838	PARG27840	PARG27841	PARG27842	PARG27843	PARG27844	PARG27845
PARG27846	PARG27847	PARG27849	PARG27851	PARG27852	PARG27853	PARG27854
PARG27855	PARG27856	PARG27857	PARG27858	PARG27859	PARG27860	PARG27861
PARG27862	PARG27863	PARG27864	PARG27865	PARG27866	PARG27867	PARG27868

PARG27869	PARG27870	PARG27871	PARG27872	PARG27873	PARG27874	PARG27875
PARG27880	PARG27882	PARG27883	PARG27884	PARG27885	PARG27886	PARG27887
PARG27888	PARG27889	PARG27890	PARG27891	PARG27892	PARG27893	PARG27894
PARG27895	PARG27896	PARG27897	PARG27899	PARG27900	PARG27901	PARG27902
PARG27903	PARG27904	PARG27905	PARG27906	PARG27907	PARG27911	PARG27914
PARG27915	PARG27916	PARG27917	PARG27918	PARG27919	PARG27920	PARG27921
PARG27922	PARG27923	PARG27924	PARG27926	PARG27927	PARG27928	PARG27929
PARG27931	PARG27932	PARG27933	PARG27934	PARG27935	PARG27936	PARG27937
PARG27938	PARG27939	PARG27940	PARG27941	PARG27942	PARG27943	PARG27944
PARG27945	PARG27946	PARG27947	PARG27948	PARG27949	PARG27950	PARG27951
PARG27952	PARG27953	PARG27954	PARG27955	PARG27956	PARG27957	PARG27958
PARG27959	PARG27960	PARG27961	PARG27962	PARG27963	PARG27964	PARG27965
PARG27966	PARG27967	PARG27968	PARG27969	PARG27970	PARG27971	PARG27972
PARG27973	PARG27974	PARG27975	PARG27976	PARG27977	PARG27978	PARG27979
PARG27980	PARG27981	PARG27982	PARG27983	PARG27984	PARG27985	PARG27986
PARG27987	PARG27989	PARG27990	PARG27991	PARG27992	PARG27993	PARG27994
PARG27995	PARG27996	PARG27997	PARG27998	PARG28000	PARG28001	PARG28002
PARG28003	PARG28004	PARG28005	PARG28006	PARG28007	PARG28008	PARG28009
PARG28010	PARG28011	PARG28012	PARG28013	PARG28014	PARG28015	PARG28017
PARG28018	PARG28020	PARG28021	PARG28022	PARG28023	PARG28024	PARG28025
PARG28026	PARG28028	PARG28029	PARG28030	PARG28031	PARG28032	PARG28033
PARG28034	PARG28035	PARG28037	PARG28038	PARG28040	PARG28041	PARG28042
PARG28044	PARG28045	PARG28046	PARG28047	PARG28048	PARG28049	PARG28050
PARG28051	PARG28052	PARG28053	PARG28054	PARG28055	PARG28056	PARG28057
PARG28058	PARG28059	PARG28060	PARG28061	PARG28062	PARG28063	PARG28064
PARG28067	PARG28068	PARG28070	PARG28071	PARG28072	PARG28074	PARG28075
PARG28076	PARG28077	PARG28078	PARG28079	PARG28080	PARG28087	PARG28088
PARG28089	PARG28090	PARG28091	PARG28092	PARG28093	PARG28094	PARG28095
PARG28096	PARG28097	PARG28098	PARG28099	PARG28100	PARG28101	PARG28102
PARG28103	PARG28104	PARG28105	PARG28106	PARG28107	PARG28108	PARG28109
PARG28110	PARG28111	PARG28112	PARG28113	PARG28114	PARG28115	PARG28116
PARG28117	PARG28118	PARG28119	PARG28120	PARG28121	PARG28122	PARG28123
PARG28124	PARG28125	PARG28126	PARG28127	PARG28128	PARG28129	PARG28130
PARG28131	PARG28132	PARG28133	PARG28134	PARG28135	PARG28136	PARG28138
PARG28139	PARG28140	PARG28141	PARG28144	PARG28146	PARG28147	PARG28149
PARG28150	PARG28151	PARG28152	PARG28153	PARG28154	PARG28155	PARG28156
PARG28157	PARG28158	PARG28159	PARG28160	PARG28161	PARG28162	PARG28163
PARG28164	PARG28165	PARG28166	PARG28167	PARG28168	PARG28169	PARG28170
PARG28171	PARG28172	PARG28173	PARG28174	PARG28175	PARG28176	PARG28177
PARG28178	PARG28179	PARG28180	PARG28181	PARG28182	PARG28183	PARG28184
PARG28185	PARG28186	PARG28187	PARG28188	PARG28189	PARG28190	PARG28191
PARG28192	PARG28193	PARG28194	PARG28195	PARG28196	PARG28198	PARG28199
PARG28201	PARG28202	PARG28203	PARG28204	PARG28205	PARG28206	PARG28207
PARG28208	PARG28210	PARG28212	PARG28213	PARG28214	PARG28216	PARG28222
PARG28223	PARG28225	PARG28226	PARG28227	PARG28228	PARG28229	PARG28230
PARG28231	PARG28232	PARG28233	PARG28234	PARG28235	PARG28236	PARG28237

PARG28238	PARG28239	PARG28240	PARG28241	PARG28242	PARG28243	PARG28244
PARG28245	PARG28246	PARG28247	PARG28248	PARG28249	PARG28250	PARG28251
PARG28252	PARG28253	PARG28254	PARG28255	PARG28256	PARG28257	PARG28258
PARG28259	PARG28260	PARG28261	PARG28262	PARG28263	PARG28264	PARG28265
PARG28266	PARG28267	PARG28268	PARG28269	PARG28270	PARG28271	PARG28272
PARG28273	PARG28274	PARG28275	PARG28276	PARG28277	PARG28278	PARG28279
PARG28280	PARG28281	PARG28282	PARG28283	PARG28284	PARG28285	PARG28286
PARG28287	PARG28288	PARG28289	PARG28290	PARG28291	PARG28292	PARG28293
PARG28294	PARG28295	PARG28296	PARG28297	PARG28298	PARG28299	PARG28300
PARG28301	PARG28302	PARG28303	PARG28304	PARG28305	PARG28306	PARG28307
PARG28308	PARG28309	PARG28310	PARG28311	PARG28312	PARG28313	PARG28314
PARG28315	PARG28316	PARG28317	PARG28318	PARG28319	PARG28320	PARG28322
PARG28323	PARG28324	PARG28325	PARG28326	PARG28327	PARG28328	PARG28329
PARG28330	PARG28331	PARG28332	PARG28333	PARG28334	PARG28335	PARG28336
PARG28337	PARG28338	PARG28339	PARG28340	PARG28341	PARG28342	PARG28343
PARG28344	PARG28346	PARG28347	PARG28348	PARG28349	PARG28351	PARG28355
PARG28358	PARG28359	PARG28360	PARG28373	PARG28375	PARG28376	PARG28377
PARG28378	PARG28379	PARG28383	PARG28391	PARG28393	PARG28394	PARG28399
PARG28406	PARG28407	PARG28409	PARG28410	PARG28411	PARG28412	PARG28413
PARG28414	PARG28415	PARG28416	PARG28417	PARG28418	PARG28424	PARG28425
PARG28426	PARG28427	PARG28428	PARG28429	PARG28430	PARG28431	PARG28432
PARG28433	PARG28434	PARG28435	PARG28436	PARG28437	PARG28439	PARG28440
PARG28441	PARG28443	PARG28444	PARG28445	PARG28446	PARG28447	PARG28448
PARG28451	PARG28452	PARG28453	PARG28454	PARG28459	PARG28460	PARG28470
PARG28471	PARG28475	PARG28476	PARG28478	PARG28479	PARG28480	PARG28482
PARG28483	PARG28484	PARG28485	PARG28486	PARG28488	PARG28489	PARG28491
PARG28492	PARG28493	PARG28494	PARG28495	PARG28497	PARG28500	PARG28504
PARG28507	PARG28508	PARG28512	PARG28513	PARG28514	PARG28515	PARG28516
PARG28517	PARG28520	PARG28523	PARG28524	PARG28525	PARG28526	PARG28527
PARG28528	PARG28529	PARG28530	PARG28531	PARG28532	PARG28540	PARG28541
PARG28546	PARG28547	PARG28549	PARG28551	PARG28553	PARG28555	PARG28557
PARG28561	PARG28562	PARG28563	PARG28564	PARG28565	PARG28567	PARG28568
PARG28569	PARG28570	PARG28573	PARG28574	PARG28575	PARG28577	PARG28578
PARG28579	PARG28580	PARG28581	PARG28583	PARG28584	PARG28587	PARG28590
PARG28591	PARG28593	PARG28595	PARG28596	PARG28597	PARG28599	PARG28600
PARG28601	PARG28604	PARG28605	PARG28606	PARG28609	PARG28613	PARG28616
PARG28618	PARG28619	PARG28622	PARG28625	PARG28626	PARG28627	PARG28628
PARG28629	PARG28630	PARG28631	PARG28632	PARG28633	PARG28636	PARG28637
PARG28638	PARG28640	PARG28643	PARG28644	PARG28645	PARG28646	PARG28647
PARG28648	PARG28649	PARG28650	PARG28651	PARG28652	PARG28657	PARG28660
PARG28661	PARG28663	PARG28664	PARG28665	PARG28666	PARG28667	PARG28668
PARG28669	PARG28671	PARG28672	PARG28676	PARG28677	PARG28682	PARG28683
PARG28685	PARG28686	PARG28687	PARG28689	PARG28690	PARG28691	PARG28692
PARG28693	PARG28696	PARG28697	PARG28701	PARG28707	PARG28708	PARG28709
PARG28712	PARG28714	PARG28715	PARG28716	PARG28717	PARG28718	PARG28720
PARG28727	PARG28730	PARG28732	PARG28734	PARG28735	PARG28736	PARG28737

PARG28738	PARG28739	PARG28740	PARG28741	PARG28742	PARG28743	PARG28744
PARG28745	PARG28750	PARG28751	PARG28752	PARG28753	PARG28755	PARG28756
PARG28761	PARG28763	PARG28777	PARG28779	PARG28780	PARG28781	PARG28786
PARG28787	PARG28790	PARG28796	PARG28797	PARG28798	PARG28800	PARG28802
PARG28808	PARG28809	PARG28810	PARG28816	PARG28818	PARG28819	PARG28823
PARG28824	PARG28825	PARG28826	PARG28827	PARG28830	PARG28831	PARG28832
PARG28833	PARG28835	PARG28843	PARG28845	PARG28846	PARG28852	PARG28855
PARG28856	PARG28868	PARG28869	PARG28871	PARG28872	PARG28875	PARG28876
PARG28879	PARG28880	PARG28883	PARG28884	PARG28887	PARG28888	PARG28890
PARG28891	PARG28894	PARG28895	PARG28897	PARG28898	PARG28901	PARG28904
PARG28905	PARG28907	PARG28912	PARG28918	PARG28919	PARG28922	PARG28923
PARG28927	PARG28928	PARG28932	PARG28933	PARG28935	PARG28936	PARG28941
PARG28942	PARG28944	PARG28945	PARG28947	PARG28951	PARG28952	PARG28953
PARG28954	PARG28955	PARG28956	PARG28957	PARG28961	PARG28962	PARG28964
PARG28965	PARG28968	PARG28969	PARG28970	PARG28975	PARG28976	PARG28980
PARG28981	PARG28984	PARG28985	PARG28989	PARG28990	PARG28992	PARG28995
PARG28996	PARG29000	PARG29001	PARG29005	PARG29006	PARG29010	PARG29011
PARG29014	PARG29019	PARG29021	PARG29022	PARG29023	PARG29024	PARG29025
PARG29026	PARG29027	PARG29028	PARG29032	PARG29033	PARG29036	PARG29037
PARG29039	PARG29041	PARG29045	PARG29046	PARG29049	PARG29053	PARG29056
PARG29057	PARG29058	PARG29061	PARG29062	PARG29067	PARG29068	PARG29072
PARG29073	PARG29076	PARG29078	PARG29080	PARG29083	PARG29084	PARG29089
PARG29090	PARG29095	PARG29096	PARG29101	PARG29103	PARG29105	PARG29106
PARG29107	PARG29108	PARG29109	PARG29110	PARG29111	PARG29113	PARG29114
PARG29115	PARG29116	PARG29120	PARG29121	PARG29124	PARG29125	PARG29127
PARG29130	PARG29133	PARG29138	PARG29139	PARG29140	PARG29141	PARG29142
PARG29143	PARG29144	PARG29145	PARG29146	PARG29147	PARG29148	PARG29149
PARG29150	PARG29151	PARG29152	PARG29153	PARG29154	PARG29155	PARG29156
PARG29160	PARG29161	PARG29162	PARG29163	PARG29164	PARG29166	PARG29167
PARG29169	PARG29171	PARG29172	PARG29174	PARG29175	PARG29178	PARG29179
PARG29180	PARG29181	PARG29182	PARG29183	PARG29184	PARG29185	PARG29186
PARG29187	PARG29188	PARG29189	PARG29192	PARG29193	PARG29194	PARG29195
PARG29196	PARG29197	PARG29198	PARG29199	PARG29201	PARG29202	PARG29205
PARG29206	PARG29207	PARG29208	PARG29211	PARG29212	PARG29213	PARG29214
PARG29215	PARG29216	PARG29220	PARG29221	PARG29222	PARG29223	PARG29225
PARG29226	PARG29227	PARG29228	PARG29229	PARG29230	PARG29231	PARG29232
PARG29233	PARG29234	PARG29235	PARG29236	PARG29237	PARG29240	PARG29241
PARG29242	PARG29245	PARG29246	PARG29247	PARG29248	PARG29249	PARG29250
PARG29251	PARG29252	PARG29253	PARG29254	PARG29255	PARG29256	PARG29257
PARG29258	PARG29259	PARG29260	PARG29261	PARG29262	PARG29263	PARG29264
PARG29265	PARG29267	PARG29268	PARG29269	PARG29270	PARG29271	PARG29272
PARG29273	PARG29274	PARG29275	PARG29276	PARG29277	PARG29278	PARG29279
PARG29280	PARG29281	PARG29282	PARG29283	PARG29284	PARG29285	PARG29286
PARG29287	PARG29288	PARG29289	PARG29291	PARG29292	PARG29293	PARG29295
PARG29296	PARG29297	PARG29298	PARG29299	PARG29300	PARG29301	PARG29303
PARG29304	PARG29305	PARG29306	PARG29308	PARG29309	PARG29310	PARG29311

PARG29314	PARG29315	PARG29319	PARG29320	PARG29321	PARG29322	PARG29323
PARG29324	PARG29325	PARG29326	PARG29327	PARG29328	PARG29329	PARG29330
PARG29331	PARG29333	PARG29334	PARG29336	PARG29337	PARG29338	PARG29339
PARG29341	PARG29343	PARG29344	PARG29345	PARG29346	PARG29347	PARG29348
PARG29349	PARG29350	PARG29352	PARG29353	PARG29354	PARG29355	PARG29356
PARG29357	PARG29358	PARG29359	PARG29360	PARG29361	PARG29363	PARG29366
PARG29367	PARG29368	PARG29369	PARG29370	PARG29371	PARG29372	PARG29374
PARG29377	PARG29378	PARG29379	PARG29382	PARG29383	PARG29384	PARG29385
PARG29386	PARG29387	PARG29388	PARG29389	PARG29390	PARG29391	PARG29392
PARG29395	PARG29396	PARG29399	PARG29400	PARG29401	PARG29402	PARG29403
PARG29404	PARG29405	PARG29406	PARG29407	PARG29408	PARG29409	PARG29410
PARG29411	PARG29412	PARG29413	PARG29414	PARG29415	PARG29416	PARG29417
PARG29419	PARG29421	PARG29422	PARG29423	PARG29425	PARG29426	PARG29427
PARG29428	PARG29429	PARG29430	PARG29431	PARG29432	PARG29433	PARG29434
PARG29435	PARG29436	PARG29437	PARG29438	PARG29440	PARG29442	PARG29447
PARG29448	PARG29449	PARG29450	PARG29451	PARG29452	PARG29454	PARG29455
PARG29456	PARG29457	PARG29458	PARG29459	PARG29460	PARG29461	PARG29462
PARG29463	PARG29465	PARG29466	PARG29467	PARG29468	PARG29469	PARG29470
PARG29471	PARG29472	PARG29473	PARG29474	PARG29475	PARG29478	PARG29479
PARG29480	PARG29481	PARG29482	PARG29483	PARG29484	PARG29485	PARG29486
PARG29487	PARG29488	PARG29489	PARG29490	PARG29491	PARG29492	PARG29493
PARG29494	PARG29495	PARG29496	PARG29500	PARG29501	PARG29502	PARG29505
PARG29513	PARG29514	PARG29515	PARG29516	PARG29517	PARG29518	PARG29520
PARG29522	PARG29523	PARG29524	PARG29525	PARG29526	PARG29527	PARG29528
PARG29529	PARG29530	PARG29531	PARG29532	PARG29533	PARG29534	PARG29535
PARG29536	PARG29537	PARG29538	PARG29539	PARG29540	PARG29541	PARG29542
PARG29543	PARG29544	PARG29545	PARG29546	PARG29547	PARG29548	PARG29549
PARG29550	PARG29551	PARG29552	PARG29554	PARG29555	PARG29556	PARG29557
PARG29558	PARG29559	PARG29560	PARG29561	PARG29562	PARG29563	PARG29564
PARG29565	PARG29566	PARG29567	PARG29568	PARG29569	PARG29570	PARG29571
PARG29572	PARG29573	PARG29574	PARG29575	PARG29576	PARG29577	PARG29578
PARG29579	PARG29580	PARG29581	PARG29582	PARG29585	PARG29588	PARG29589
PARG29590	PARG29591	PARG29593	PARG29594	PARG29595	PARG29596	PARG29597
PARG29598	PARG29601	PARG29605	PARG29607	PARG29610	PARG29636	PARG29638
PARG29639	PARG29640	PARG29641	PARG29642	PARG29643	PARG29647	PARG29649
PARG29653	PARG29654	PARG29655	PARG29656	PARG29657	PARG29658	PARG29659
PARG29660	PARG29661	PARG29662	PARG29665	PARG29668	PARG29670	PARG29673
PARG29676	PARG29677	PARG29679	PARG29680	PARG29682	PARG29683	PARG29684
PARG29686	PARG29689	PARG29690	PARG29691	PARG29700	PARG29701	PARG29703
PARG29704	PARG29705	PARG29706	PARG29707	PARG29709	PARG29713	PARG29714
PARG29715	PARG29716	PARG29718	PARG29720	PARG29721	PARG29722	PARG29723
PARG29725	PARG29726	PARG29727	PARG29728	PARG29729	PARG29732	PARG29733
PARG29734	PARG29735	PARG29736	PARG29737	PARG29738	PARG29739	PARG29740
PARG29741	PARG29742	PARG29743	PARG29744	PARG29745	PARG29746	PARG29747
PARG29748	PARG29749	PARG29750	PARG29751	PARG29752	PARG29753	PARG29754
PARG29757	PARG29758	PARG29759	PARG29760	PARG29761	PARG29762	PARG29763

PARG29764	PARG29765	PARG29766	PARG29767	PARG29768	PARG29769	PARG29770
PARG29772	PARG29774	PARG29783	PARG29787	PARG29788	PARG29789	PARG29790
PARG29792	PARG29794	PARG29795	PARG29797	PARG29798	PARG29799	PARG29800
PARG29801	PARG29802	PARG29803	PARG29804	PARG29805	PARG29806	PARG29807
PARG29808	PARG29809	PARG29810	PARG29811	PARG29812	PARG29821	PARG29823
PARG29824	PARG29825	PARG29827	PARG29829	PARG29830	PARG29831	PARG29832
PARG29835	PARG29838	PARG29846	PARG29848	PARG29849	PARG29853	PARG29857
PARG29858	PARG29861	PARG29862	PARG29866	PARG29867	PARG29870	PARG29871
PARG29873	PARG29875	PARG29876	PARG29879	PARG29880	PARG29883	PARG29884
PARG29887	PARG29888	PARG29890	PARG29892	PARG29893	PARG29895	PARG29901
PARG29902	PARG29904	PARG29906	PARG29907	PARG29921	PARG29922	PARG29923
PARG29924	PARG29927	PARG29929	PARG29932	PARG29935	PARG29938	PARG29939
PARG29941	PARG29942	PARG29945	PARG29948	PARG29949	PARG29950	PARG29951
PARG29952	PARG29953	PARG29954	PARG29956	PARG29957	PARG29958	PARG29961
PARG29962	PARG29963	PARG29964	PARG29966	PARG29967	PARG29968	PARG29969
PARG29970	PARG29971	PARG29972	PARG29973	PARG29974	PARG29976	PARG29977
PARG29978	PARG29979	PARG29980	PARG29981	PARG29982	PARG29983	PARG29985
PARG29986	PARG29988	PARG29989	PARG29990	PARG29991	PARG29992	PARG29993
PARG29994	PARG29996	PARG29997	PARG29998	PARG30001	PARG30002	PARG30004
PARG30007	PARG30008	PARG30009	PARG30010	PARG30011	PARG30012	PARG30013
PARG30014	PARG30015	PARG30016	PARG30017	PARG30019	PARG30020	PARG30021
PARG30022	PARG30023	PARG30030	PARG30033	PARG30035	PARG30036	PARG30037
PARG30038	PARG30039	PARG30040	PARG30041	PARG30044	PARG30047	PARG30048
PARG30049	PARG30050	PARG30053	PARG30055	PARG30057	PARG30058	PARG30059
PARG30062	PARG30065	PARG30067	PARG30068	PARG30069	PARG30070	PARG30071
PARG30073	PARG30076	PARG30078	PARG30084	PARG30085	PARG30088	PARG30089
PARG30090	PARG30091	PARG30092	PARG30093	PARG30094	PARG30095	PARG30096
PARG30097	PARG30098	PARG30099	PARG30100	PARG30101	PARG30102	PARG30103
PARG30104	PARG30105	PARG30106	PARG30107	PARG30108	PARG30109	PARG30110
PARG30111	PARG30112	PARG30113	PARG30115	PARG30116	PARG30117	PARG30118
PARG30119	PARG30121	PARG30156	PARG30161	PARG30165	PARG30166	PARG30167
PARG30169	PARG30173	PARG30174	PARG30179	PARG30180	PARG30184	PARG30185
PARG30189	PARG30190	PARG30193	PARG30196	PARG30197	PARG30199	PARG30200
PARG30210	PARG30211	PARG30212	PARG30213	PARG30214	PARG30215	PARG30216
PARG30219	PARG30220	PARG30221	PARG30222	PARG30223	PARG30226	PARG30227
PARG30228	PARG30229	PARG30233	PARG30234	PARG30238	PARG30239	PARG30243
PARG30244	PARG30247	PARG30249	PARG30250	PARG30251	PARG30252	PARG30253
PARG30254	PARG30255	PARG30256	PARG30257	PARG30258	PARG30259	PARG30260
PARG30264	PARG30265	PARG30269	PARG30270	PARG30272	PARG30275	PARG30276
PARG30277	PARG30278	PARG30281	PARG30282	PARG30283	PARG30284	PARG30285
PARG30290	PARG30291	PARG30294	PARG30295	PARG30298	PARG30299	PARG30304
PARG30307	PARG30308	PARG30311	PARG30312	PARG30315	PARG30316	PARG30318
PARG30320	PARG30321	PARG30322	PARG30323	PARG30326	PARG30329	PARG30330
PARG30331	PARG30333	PARG30335	PARG30336	PARG30339	PARG30340	PARG30341
PARG30343	PARG30344	PARG30345	PARG30347	PARG30348	PARG30349	PARG30350
PARG30351	PARG30352	PARG30353	PARG30354	PARG30355	PARG30357	PARG30359

PARG30360	PARG30364	PARG30365	PARG30366	PARG30370	PARG30371	PARG30374
PARG30377	PARG30379	PARG30380	PARG30381	PARG30382	PARG30383	PARG30384
PARG30385	PARG30386	PARG30388	PARG30389	PARG30390	PARG30391	PARG30392
PARG30393	PARG30394	PARG30399	PARG30400	PARG30401	PARG30402	PARG30405
PARG30406	PARG30407	PARG30408	PARG30409	PARG30410	PARG30411	PARG30418
PARG30419	PARG30423	PARG30424	PARG30425	PARG30426	PARG30427	PARG30428
PARG30429	PARG30431	PARG30432	PARG30434	PARG30435	PARG30437	PARG30438
